# CRISPR-based dissection of microRNA-23a ~ 27a ~ 24-2 cluster functionality in hepatocellular carcinoma

**DOI:** 10.1038/s41388-024-03115-z

**Published:** 2024-08-07

**Authors:** Mengying Cui, Zhichao Liu, Shuaibin Wang, Sejong Bae, Hua Guo, Jiangbing Zhou, Runhua Liu, Lizhong Wang

**Affiliations:** 1 Department of Genetics, University of Alabama at Birmingham, Birmingham, AL USA; 2 Department of O’Neal Comprehensive Cancer Center, University of Alabama at Birmingham, Birmingham, AL USA; 3 Department of Medicine, University of Alabama at Birmingham, Birmingham, AL USA; 4 Department of Pathology, University of Alabama at Birmingham, Birmingham, AL USA; 5 Department of Neurosurgery, Yale University, New Haven, CT USA; 6 Department of Biomedical Engineering, Yale University, New Haven, CT USA

**Keywords:** Liver cancer, Non-coding RNAs

## Abstract

The miR-23a ~ 27a ~ 24-2 cluster, commonly upregulated in diverse cancers, including hepatocellular carcinoma (HCC), raises questions about the specific functions of its three mature miRNAs and their integrated function. Utilizing CRISPR knockout (KO), CRISPR interference (CRISPRi), and CRISPR activation (CRISPRa) technologies, we established controlled endogenous miR-23a ~ 27 ~ a24-2 cell models to unravel their roles and signaling pathways in HCC. Both miR-23a KO and miR-27a KO displayed reduced cell growth in vitro and in vivo, revealing an integrated oncogenic function. Functional analysis indicated cell cycle arrest, particularly at the G2/M phase, through the downregulation of CDK1/cyclin B activation. High-throughput RNA-seq, combined with miRNA target prediction, unveiled the miR-23a/miR-27a-regulated gene network, validated through diverse technologies. While miR-23a and miR-27a exhibited opposing roles in cell migration and mesenchymal-epithelial transition, an integrated CRISPRi/a analysis suggested an oncogenic role of the miR-23a ~ 27a ~ 24-2 cluster in cell migration. This involvement potentially encompasses two signaling axes: miR-23a-BMPR2 and miR-27a-TMEM170B in HCC cells. In conclusion, our CRISPRi/a study provides a valuable tool for comprehending the integrated roles and underlying mechanisms of endogenous miRNA clusters, paving the way for promising directions in miRNA-targeted therapy interventions.

## Introduction

MicroRNAs (miRNAs or miRs) are endogenous, small non-coding RNAs involved in post-transcriptional regulation, leading to mRNA cleavage or translation repression of target genes. This regulatory process influences various biological phenomena such as cell differentiation, proliferation, apoptosis, and metabolism [1]. Remarkably, a single microRNA can regulate multiple genes, and even subtle changes in miRNA expression levels can have profound effects on biological functions [2]. Of particular importance are clustered miRNAs, which are located at the same locus in the genome and transcribed as a single primary miRNA (pri-miRNA). Unfortunately, the significance of these clustered miRNAs has often been overlooked [3]. Given the interactive and complex nature of the regulatory network between miRNAs and mRNAs, it becomes essential to comprehensively understand the regulation, properties, and biological functions of miRNAs when they are organized into clusters.

The miR‐23a‐27a‐24‐2 cluster located on chromosome 9q22 encodes a pri-miRNA that consists of three individual miRNAs: miR-23a, miR-27a, and miR-24 [4, 5]. These three miRNAs function independently, as they regulate distinct target genes [4, 5]. Consequently, the miR-23a ~ 27a ~ 24-2 cluster plays diverse roles in processes such as development, tumorigenesis, invasion, metastasis, vascular remodeling, tumor immunity, and drug resistance [5]. However, despite its significance, studies on the individual mature miRNAs (miR-23a, miR-27a, miR-24) within this cluster have yielded inconclusive results. Addressing these inconsistencies requires an integrated functional analysis of the endogenous miR-23a ~ 27a ~ 24-2 cluster alongside the individual mature miRNAs. In human cancers, including hepatocellular carcinoma (HCC), the expression of miR‐23a‐27a‐24‐2 pri-miRNA is upregulated compared to normal tissues [6]. Nevertheless, investigations into the three mature miRNAs within the miR-23a ~ 27a ~ 24-2 cluster in HCC have reported inconsistent results for miR-23a [7–15], miR-27a [16–25], and miR-24 [26–29]. This inconsistency suggests their potential involvement in distinct cell signaling pathways, contributing to self‐regulation and feedback loops under diverse circumstances [30, 31].

Traditional methods involving overexpression through transfection with plasmids or RNA interference techniques may introduce exogenous variables, potentially leading to spurious effects and not accurately reflecting endogenous miRNA dynamics. Moreover, these approaches may not capture the comprehensive expression profile governing clustered miRNAs. In the present study, we addressed these challenges by establishing an endogenous miR-23a ~ 27a ~ 24-2 controllable system using CRISPR technologies. Leveraging CRISPR genomic editing, we individually knocked out each endogenous mature miRNA within the miR-23a ~ 27a ~ 24-2 cluster in HCC cells to discern the functional roles of individual miRNAs. Furthermore, employing CRISPR epigenomic editing through CRISPR interference (CRISPRi) and CRISPR activation (CRISPRa), we precisely modulated the expression of pri-miR-23a ~ 27a ~ 24-2 and simultaneously controlled the co-expression of its three mature miRNAs, providing insights into the overall regulation of clustered miRNAs. In addition, we investigated the functional roles of both the miR-23a ~ 27a ~ 24-2 cluster and its individual mature miRNAs in HCC cell proliferation, apoptosis, and migration. Utilizing this novel platform, we conducted a comprehensive analysis to dissect the functional roles and identify underlying targets and signaling pathways associated with the microRNA-23a-27a-24-2 cluster in HCC cells.

## Results

### Characterization of miR-23a ~ 27a ~ 24-2 expression and its survival outcomes in HCC tissues and cell lines

The miR-23a, miR-27a, and miR-24 are transcribed from the miR-23a ~ 27a ~ 24-2 cluster (Supplementary Fig. S1A). The Cancer Genome Atlas (TCGA) dataset analysis showed no significant difference in the expression of miR-23a (*p* = 0.980) and miR-27a (*p* = 0.129), with only a significant difference in the expression of miR-24-2 (*p* = 0.04) between HCC and normal liver tissues (Supplementary Fig. S1B). Additionally, in the Kaplan–Meier Plotter RNA-sequencing (RNA-seq) dataset, Cox regression analysis revealed poor overall survival for high expression of miR-23a (hazard ratio (HR) = 1.67, *p* = 0.0052), miR-27a (HR = 1.65, *p* = 0.0057), and miR-24 (HR = 1.77, *p* = 0.0021) compared to low expression of these miRNAs in HCC patients (Supplementary Fig. S1C–E).

**Fig. 1:**
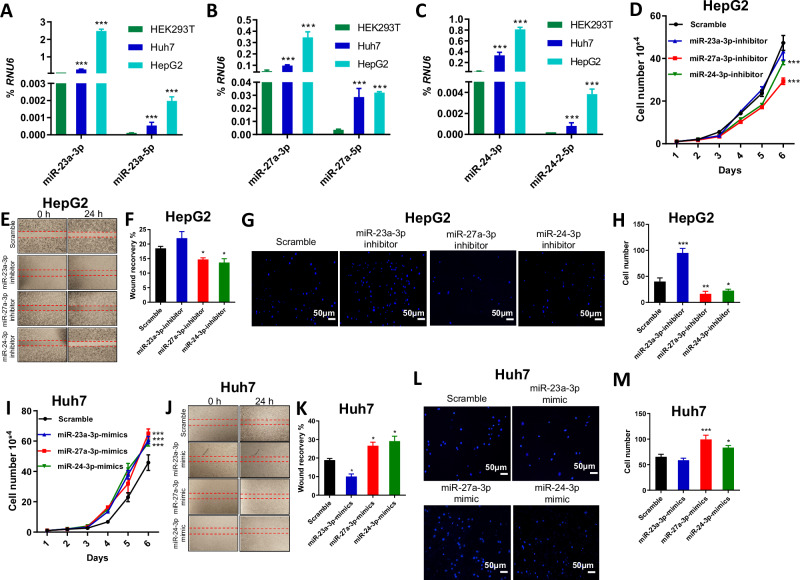
Effect of miRNAs in the miR-23a ~ 27a ~ 24-2 cluster on cell proliferation and migration of HCC cells. **A**–**C** Quantification of miR-23a-3p/5p, miR-27a-3p/5p, and miR-24-3p/5p expression by qPCR as a percentage of *RNU6* expression in HEK293T, Huh7, and HepG2 cells. Data are presented as means ± standard deviation (SD). ***p < 0.001 by one-way ANOVA Tukey’s multiple comparisons test for HCC cells *vs*. HEK293T cells. **D**, **I** Cell growth curve in HepG2 and Huh7 cells treated with scramble (Scr), miRNA inhibitor, and miRNA mimic for 6 days. Data are presented as means ± SD. ***p < 0.001 by two-way ANOVA test *vs*. Scr group. **E**, **F**, **J**, **K** Scratch migration assay and quantitative analysis in HepG2 and Huh7 cells. Data are presented as means ± SD. *p < 0.05 by one-way ANOVA Tukey’s multiple comparisons test vs. Scr group. **G**, **H**, **L**, **M** Transwell migration assay and quantitative analysis in HepG2 and Huh7 cells. Data are presented as means ± SD. *p < 0.05 and **p < 0.01 by one-way ANOVA Tukey’s multiple comparisons test vs. Scr group. All experiments were repeated at least two times.

However, the expression of these miRNAs in these datasets is likely from pri-mRNAs of the miR-23a ~ 27a ~ 24-2 cluster. Next, we characterized the expression level of mature miRNAs in the miR-23a ~ 27a ~ 24-2 cluster in HEK293T, Huh7, and HepG2 cell lines using quantitative real-time PCR (qPCR). The expression of mature miR-23a-3p/5p, miR-27a-3p/5p, and miR-24-3p/5p was higher in HepG2 and Huh7 cells than in HEK293T cells, with the highest expression evident in HepG2 cells (Fig. 1A–C). In particular, the mature 3p miRNAs (miR-23a-3p, miR-27a-3p, and miR-24-3p) were consistently expressed more than 10-fold higher in cells compared to the mature 5p miRNAs (miR-23a-5p, miR-27a-5p, miR-24-2-5p), indicating a predominant expression of 3p miRNAs in the miR-23a ~ 27a ~ 24-2 cluster. Thus, using the HepG2 cell line, we established miRNA KO HCC cell models and focused on the expression of 3p’ miRNAs as the main factors when choosing miRNA KO cell colonies. In addition, miR-24 is also expressed in the miR-23b ~ 27b ~ 24-1 cluster at 9q22.32. To reduce the complexity of our analysis, in the present study, we only focused on miR-23a and miR-27a.

### Establishment of miR-23a/miR-27a KO HepG2 cell models

Using CRISPR/Cas9 genomic editing, we generated scramble and miR-23a/miR-27a KO HepG2 cell models. The miRNA expression in selected cell colonies was assessed by qPCR. As shown in Supplementary Fig. S2A, miR-23a KO markedly decreased the expression of miR-23a-3p in two clones, while varied expressions of miR-23a-5p were observed. Notably, miR-23a KO also led to decreased expressions of miR-27a-3p and miR-24-3p, but not miR-27a-5p and miR-24-5p in the two miR-23a KO clones. Furthermore, miR-27a KO reduced the expression of miR-27a-3p/5p and miR-24-3p/5p in two miR-27a KO clones (Supplementary Fig. S2B). However, miR-27a KO slightly increased the expression of miR-23a-3p while decreasing the expression of miR-23a-5p in the two miR-27a KO clones (Supplementary Fig. S2B). Next, we selected miR-23a KO clones 1 and 2 (miR-23a KO1 and KO2) and miR-27a KO clones 1 and 2 (miR-27a KO1 and KO2) for Sanger DNA sequencing. Supplementary Fig. S2C and S2D show a 55 bp homozygous deletion in miR-23a KO1 and KO2, a 53 bp homozygous deletion in miR-27a KO1, and a heterozygous 53 bp deletion with a 40 bp insertion in miR-27a KO2.

### Identification of the functional role of miR-23a and miR-27a in HepG2 cells

Initially, we transfected HepG2 and Huh7 cells with miRNA mimics or inhibitors to elucidate the individual roles of miRNAs within the miR-23a ~ 27a ~ 24-2 cluster in the proliferation and migration of HCC cells. In HepG2 cells, the miR-27a-3p and miR-24-3p inhibitors notably reduced both cell proliferation and migration (Fig. 1D–H), while the miR-27a-3p but not miR-24-3p mimics seemed to induce cell proliferation and migration (Supplementary Fig. 3A–E). Surprisingly, the miR-23a-3p inhibitor did not influence cell proliferation but appeared to induce cell migration (Fig. 1D–H), while the miR-23a-3p mimic seemed to induce cell proliferation but inhibited cell migration in HepG2 cells (Supplementary Fig. 3A–E). In Huh7 cells, the miR-23a-3p, miR-27a-3p, and miR-24-3p mimics induced cell proliferation (Fig. 1I), while the miR-23a-3p, miR-27a-3p, and miR-24-3p inhibitors reduced cell proliferation (Supplementary Fig. 3F). However, while the miR-27a-3p and miR-24-3p mimics promoted cell migration, the miR-23a-3p mimic seemed to inhibit cell migration (Fig. 1J–M). Also, the miR-27a-3p and miR-24-3p inhibitors reduced cell migration, while the miR-23a-3p mimic induced cell migration (Supplementary Fig. 3G–J).

**Fig. 2:**
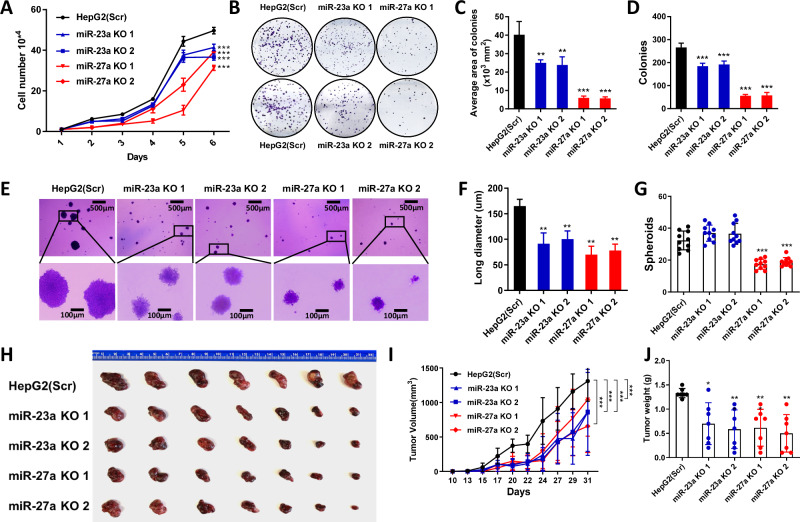
Effect of miR-23a/miR-27a knockout on cell proliferation and xenograft tumor growth of HCC cells. **A** Cell growth curve in Scr, miR-23a knockout (KO), and miR-27a KO HepG2 cells for 6 days. Data are presented as means ± SD. ***p < 0.001 by two-way ANOVA test *vs*. Scr group. **B**–**D** Colony formation assay and quantitative analysis of area and number of colonies in Scr, miR-23a KO, and miR-27a KO HepG2 cells. Data are presented as means ± SD. *p < 0.05, **p < 0.01, and ***p < 0.001 by one-way ANOVA Tukey’s multiple comparisons test vs. Scr group. **E**–**G** Soft agar assay and quantitative analysis of size and number of spheroids at 4 weeks after implantation. Data are presented as means ± SD. *p < 0.05, **p < 0.01, ***p < 0.001 by one-way ANOVA Tukey’s multi*p*le comparisons test vs. Scr group. Scale bars = 500 μm/100 μm. **H**–**J** Representative images of xenograft tumors at day 31 after injection and tumor growth and weights in NSG mice subcutaneously injected with Scr (n = 7), miR-23a KO1 (n = 7), miR-23a KO2 (n = 7), miR-27a KO1 (n = 7), or miR-27a KO2 (n = 7) HepG2 cells. Data are presented as means ± SD of the tumor volumes. **p < 0.01 and ***p < 0.001 by two-way ANOVA test or one-way ANOVA Tukey’s multiple comparisons test vs. Scr group. All experiments were repeated three times.

Subsequently, using our established KO cell models, we conducted various cell proliferation assays to assess the impact of miRNAs in the miR-23a ~ 27a ~ 24-2 cluster on HepG2 cell growth. Cell growth was notably slower in miR-23a KO and miR-27a KO cells compared to scrambled cells (Fig. 2A). The colony formation assay revealed reduced number and area of the colonies in miR-23a KO or miR-27a KO cells compared with scrambled cells (Fig. 2B–D). Remarkably, miR-27a played a dominant role in HepG2 cells compared to miR-23a, evident in the suppression of cell growth and colony formation.

Similarly, in three-dimensional cell culture models, average spheroid size was diminished in miR-23a KO or miR-27a KO cells compared with scrambled cells (Fig. 2E, F). However, only miR-27a KO resulted in a decrease in colony number, not observed in miR-23a KO compared with scrambled cells (Fig. 2E, G). To corroborate these in vitro findings, we subcutaneously injected scramble, miR-23a KO, and miR-27a KO HepG2 cells into NGS mice. Xenograft tumor growth and weights were notably reduced in mice with miR-23a KO or miR-27a KO cells compared to those with scrambled cells (Fig. 2H–J).

### miR-23a KO and miR-27a KO inhibits cell proliferation through the cell cycle arrest in HepG2 cells

**Fig. 3:**
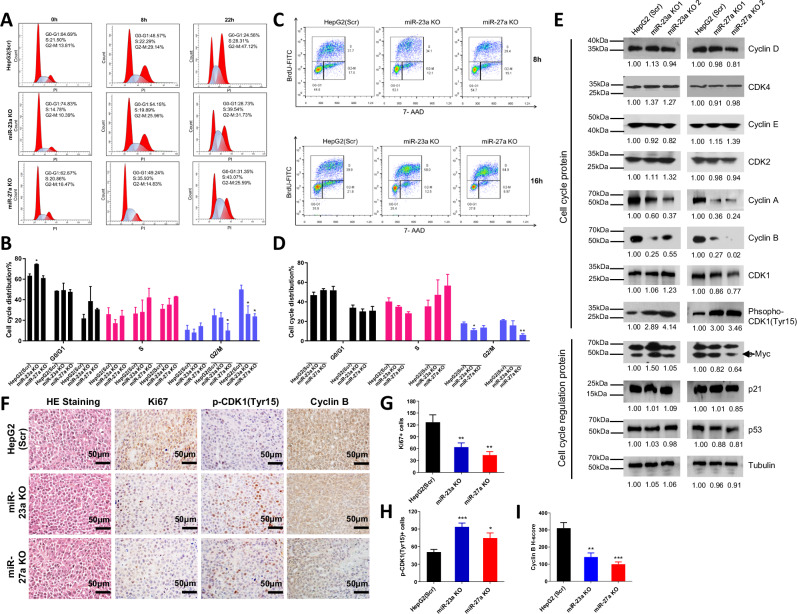
Effect of miR-23a/miR-27a knockout on cell cycle progression of HCC cells. **A**, **B** Cell cycle progression and quantitative analysis in Scr, miR-23a KO, and miR-27a KO HepG2 cells after starvation for 48 h monitored by PI staining and flow cytometry at 0, 8, and 22 h after serum stimulation. Data presented as means ± SD of triplicates. *p < 0.05 by one-way ANOVA Tukey’s multiple comparisons test *vs*. Scr group. **C**, **D** Cell cycle progression and quantitative analysis in Scr, miR-23a KO, and miR-27a KO HepG2 cells after starvation for 48 h monitored by BrdU staining and flow cytometry at 8 and 16 h after serum stimulation. Data presented as means ± SD of triplicates. *p < 0.05 and **p < 0.01 by one-way ANOVA Tukey’s multiple comparisons test *vs*. Scr group. **E** Protein expression by Western blot in Scr, miR-23a KO or miR-27a KO HepG2 cells. **F** Protein expression of Ki67, phospho (p)-CDK1(Tyr15), and Cyclin B by IHC staining in xenograft tumors injected with Scr, miR-23a KO, and miR-27a KO HepG2 cells in NSG mice. Scale bars = 50 μm. **G**–**I** Quantitative IHC analysis of Ki67+ and p-CDK1(Tyr15)+ cells and IHC H-score analysis of Cyclin B staining. *p < 0.05, **p < 0.01, and ***p < 0.001 by one-way ANOVA Tukey’s multiple comparisons test vs. Scr group. All experiments were repeated three times.

To examine whether the impact of miR-23a and miR-27a on cell proliferation is mediated through apoptosis, we treated scramble, miR-23a KO, and miR-27a KO HepG2 cells with H_2_O_2_ to induce apoptosis. However, both miR-23a KO and miR-27a KO did not appear to affect cell apoptosis before and after H_2_O_2_ stimulation when compared to scrambled cells (Supplementary Fig. S4A–D). This suggests that miR-23a KO or miR-27a KO may not influence the apoptosis of HepG2 cells. Subsequently, to investigate whether the effect of miR-23a and miR-27a on cell proliferation is related to the regulation of cell cycle progression, we arrested cells at the G0-G1 phase by serum deprivation for 48 h in scramble, miR-23a KO, and miR-27a KO HepG2 cells. After 8 h of serum stimulation, no significant differences were observed among the three groups in the S phase. However, cells entering the G2/M phase were likely decreased in the miR-27a KO group (Fig. 3A, B). After 22 h of serum stimulation, fewer cells entered the G2/M phase in both miR-23a KO and miR-27a KO groups (Fig. 3A, B). To corroborate the cell cycle results and further assess the impact of miR-23a/miR-27a on cell proliferation, BrdU staining was performed. At 8 h after serum stimulation, 37.7% of scrambled cells, 34.1% of miR-23a KO cells, and 29.4% of miR-27a KO cells were in the S phase, with no significant differences observed between scrambled and miRNA KO cells (Fig. 3C, D). After 16 h of serum stimulation, 39.9%, 58%, and 64.9% of cells entered the S phase, respectively, with no significant difference among the groups (Fig. 3C, D). However, fewer miR-23a KO cells at 8 h and miR-27a KO cells at 16 h entered the G2/M phase compared to scrambled cells (Fig. 3C, D).

To unravel the molecular mechanism underlying the growth suppression caused by miR-23a/miR-27a KO, we assessed the expression of cell cycle regulatory proteins through Western blot analysis. As depicted in Fig. 3E, both miR-23a KO and miR-27a KO led to a reduction in the expression of cyclin A and cyclin B, while cyclin D and cyclin E remained unchanged in the cells. Moreover, phospho-CDK1, essential for G2/M phase transitions, exhibited an increase in miR-23a KO or miR-27a KO cells (Fig. 3E). This suggests that the elevated phospho-CDK1 in miRNA KO cells may contribute to cell cycle arrest at the G2/M phase. Notably, the expressions of p53, p21 (essential for G1 arrest), and c-Myc (critical in G1/S progression) [32] remained unaltered among the experimental groups (Fig. 3E). Furthermore, decreased expression of Ki67 and cyclin B and increased expression of phospho-CDK1 were also validated by immunohistochemical staining (IHC) in miR-23a KO or miR-27a KO xenograft tumors compared to scrambled xenograft tumors (Fig. 3F–I). These findings suggest that miR-23a KO and miR-27a KO may promote cell proliferation through CDK1-dependent cell cycle arrest at the G2/M phase.

### Regulation of cell cycle progression and associated gene network by miR-23a and miR-27a in HepG2 Cells

**Fig. 4:**
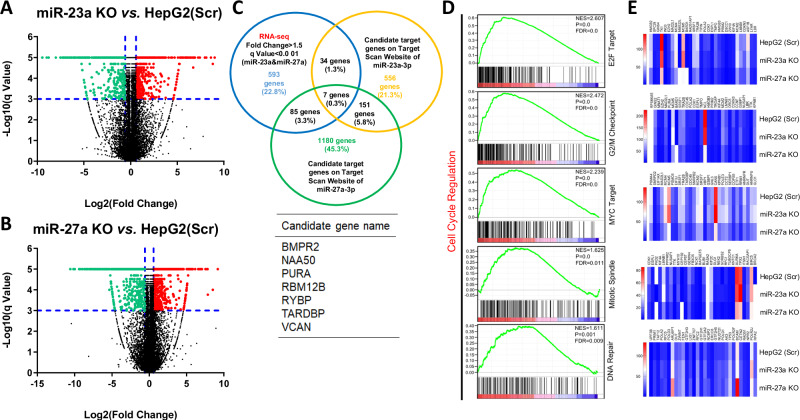
Bioinformatics analysis of RNA-seq data in miR-23a/miR-27a KO HCC cells. **A**, **B** Gene expression profiling data showing up-regulated (red) and downregulated (green) genes in miR-23a KO or miR-27a KO HepG2 cells relative to Scr HepG2 cells by RNA-seq analysis. Blue vertical line represents log_2_ (Fold Change) = 0.58, Blue horizon line represents −log_10_ (*q* value) = 3. **C** Venn diagram showing candidate target genes selected by an overlap of three gene sets: 1, Genes up-regulated both in miR-23a KO group and miR-27a KO group (Fold change >1.5, *q* < 0.001) by RNA-seq analysis (593 genes); 2, Genes that miR-23a-3p binds to 3’-untranslated region (UTR) of target gene predicted by an online website (http://www.targetscan/) (556 genes); 3, Genes that miR-27a-3p binds to 3’-UTR of target gene predicted by an online website (http://www.targetscan/) (1180 genes). The bottom table indicates a list of 7 candidate target genes by an overlap of three gene sets. **D**, **E** Gene set enrich analysis after miR-23a/miR-27a KO. **D** Top five cell signaling pathways by normalized enrichment score (NES) score ranking; **E** Heat map of differential gene expression of top 30 genes in each gene set by Rank Metric Score ranking.

To unravel the regulatory gene network influenced by miR-23a and miR-27a, we conducted RNA-seq using scramble HepG2 cells as the control group and miR-23a KO/miR-27a KO HepG2 cells as the case groups. As depicted in Fig. 4A and detailed in Supplementary Table S1A–B, miR-23a KO led to the up-regulation of 2599 genes and the down-regulation of 795 genes (>1.5-fold change, *q* < 0.001) compared to control cells. Similarly, miR-27a KO resulted in the up-regulation of 2194 genes and the down-regulation of 1147 genes (>1.5-fold change, *q* < 0.001) compared to control cells (Fig. 4B and Supplementary Table S1C, D). Furthermore, potential target genes of miR-23a and miR-27a were predicted using the public miRNA TargetScan database (Supplementary Table S2). Seven candidate genes were selected based on the criteria of (1) Fold change >1.5, *q* < 0.001 in both miR-23a KO and miR-27a KO groups, and (2) Genes binding to the sequences of both miR-23a-3p and miR-27a-3p predicted by TargetScan (Fig. 4C and Supplementary Table S3A–C). Next, we employed Gene Set Enrichment Analysis (GSEA) to identify enriched gene sets in miR-23a KO or miR-27a KO cells. As illustrated in Fig. 4D and detailed in Supplementary Table S4, the up-regulated genes were predominantly associated with cell cycle regulation, encompassing E2F target, G2/M checkpoint, MYC target, mitotic spindle, and DNA repair. Additionally, the heat map of the top 30 core enrichment genes with a high “Rank Metric Score” in each gene set is presented in Fig. 4E. Finally, among the seven-candidate target genes, *PURA* was identified as enriched in the G2/M checkpoint gene set (Fig. 4C and Supplementary Table S5) and co-regulated by miR-23a and miR-27a (Supplementary Fig. S5).

### Identification of the miR-23a ~ 27a ~ 24-2 cluster regulated gene network and its integrative function in HCC cells

**Fig. 5:**
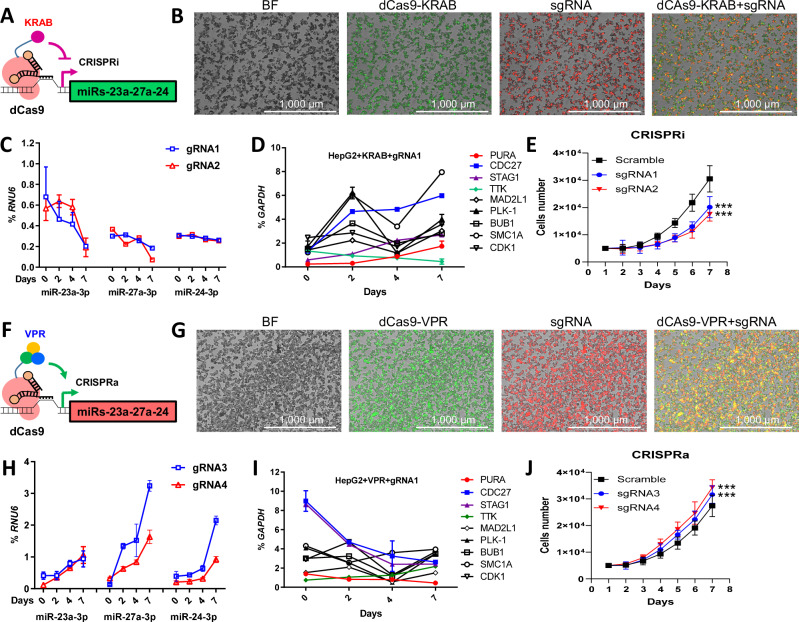
Establishment of CRISPRi/a cell models and validation of miR-23a ~ 27a ~ 24-2 cluster target genes in HCC cells. **A**, **F** Diagram of CRISPR interference (CRISPRi) and activation (CRISPRa) system. **B**, **G** Efficacy of transfection of dCas9 (GFP) and transduction of sgRNA (mCherry) into CRISPRi/a HepG2 cells at day 4 as determined by fluorescence microscopy. Scale bar = 1000 μm. **C**, **H** Dynamic changes of miR-23a-3p, miR-27a-3p, and miR-24-3p expression in CRISPRi/a HepG2 cells detected by qPCR. **D**, **I** Dynamic changes of candidate target gene expression in CRISPRi/a HepG2 cells detected by qPCR. **E**, **J** Cell growth curve in CRISPRi/a HepG2 cells for 96 h. Data are presented as means ± SD. ***p < 0.001 by two-way ANOVA test vs. Scr group. All experiments were repeated three times.

To elucidate the collaborative function of miRNAs within the miR-23a ~ 27a ~ 24-2 cluster, we established endogenous miR-23a ~ 27a ~ 24-2 CRISPR/dCas9 controllable HepG2 cell models. Utilizing the doxycycline (Dox)-inducible dCas9-VP64-p65-Rta (dCas9-VPR) for CRISPRa and Dox-inducible dCas9-KRAB for CRISPRi, we aimed to regulate the transcriptional activity of the miR-23a ~ 27a ~ 24-2 cluster in HepG2 cells (Fig. 5A, F). Different sequence-specific single guide RNAs (sgRNAs) in the promoter region of miR-23a ~ 27a ~ 24-2 were designed, and the transduced efficiency of these sgRNAs was confirmed through immunofluorescence and qPCR (Fig. 5B, G). Following Dox addition, the CRISPR/dCas9 system was induced for seven days. In the CRISPRi system, the expression of miR-23a-3p, miR-27a-3p, and miR-24-3p gradually decreased in cells transfected with sgRNA1 and sgRNA2 (Fig. 5C). Conversely, in the CRISPRa system, the expression of miR-23a-3p, miR-27a-3p, and miR-24-3p progressively increased in cells transfected with sgRNA3 and sgRNA4 (Fig. 5H).

To validate the gene network and signaling pathway involved in the miR-23a ~ 27a ~ 24-2 cluster, we identified genes enriched in the G2/M phase using KEGG mapper-cell cycle and marked the enriched genes in red (Supplementary Fig. S5). Most “red” genes were associated with the G2/M phase, consistent with the in vitro and in vivo results. Subsequently, genes enriched in the G2/M phase of the KEGG mapper-cell cycle were further investigated using our established endogenous miR-23a ~ 27a ~ 24-2 controllable HepG2 cell model. Nine genes were examined in the CRISPRi system with sgRNAs (*PURA*, *CDC27*, *STAG1*, *TTK*, *MAD2L1*, *PLK1*, *BUB1*, *SMC1A*, and *CDK1*) (Supplementary Fig. S5). The expression of *PURA*, *CDC27*, *STAG1*, and *TTK* dynamically changed under the condition of Dox induction (Fig. 5D). These four genes were validated in the CRISPRa system with sgRNAs, showing dynamic changes in expression after Dox induction (Fig. 5I). Likewise, we established endogenous miR-23a ~ 27a ~ 24-2 CRISPRi/a controllable cell models in Huh7 cells (Supplementary Fig. S6A–C and S6F–H). These four genes were further confirmed in the CRISPRi/a system in Huh7 cells, showing dynamic changes in expression after Dox induction (Supplementary Fig. S6D and S6I).

Next, we examined the functional implications of manipulating the miR-23a ~ 27a ~ 24-2 cluster in HepG2 and Huh7 cells. The introduction of Dox induced a decrease in cell proliferation in CRISPRi HepG2 and Huh7 cells (Fig. 5E and Supplementary Fig. S6E), while CRISPRa HepG2 and Huh7 cells exhibited an increase in cell proliferation following Dox induction (Fig. 5J and Supplementary Fig. S6J). These findings strongly suggest an oncogenic role of the miR-23a ~ 27a ~ 24-2 cluster in HCC cells. To gain deeper insights into the regulatory mechanisms of this miRNA cluster, we investigated the impact of 3p’ miR-23a/27a/24 mimics and inhibitors on cell proliferation in miR-23a ~ 27a ~ 24-2 CRISPRi/a HepG2 and Huh7 cells. Upon Dox induction, the miR-23a-3p and miR-27a-3p mimics enhanced cell proliferation in miR-23a ~ 27a ~ 24-2 CRISPRi cells, while the miR-23a-3p and miR-27a-3p inhibitors reduced cell proliferation in miR-23a ~ 27a ~ 24-2 CRISPRa cells (Supplementary Fig. S7A–D). However, miR-24-3p mimic and inhibitor did not exhibit effects on cell proliferation. These results suggest that miR-23a and miR-27a contributes to the integrative oncogenic function of the miR-23a ~ 27a ~ 24-2 cluster in HCC cells.

### miR-23a/miR-27a co-target *PURA* directly to promote cell proliferation in HepG2 cells

**Fig. 6:**
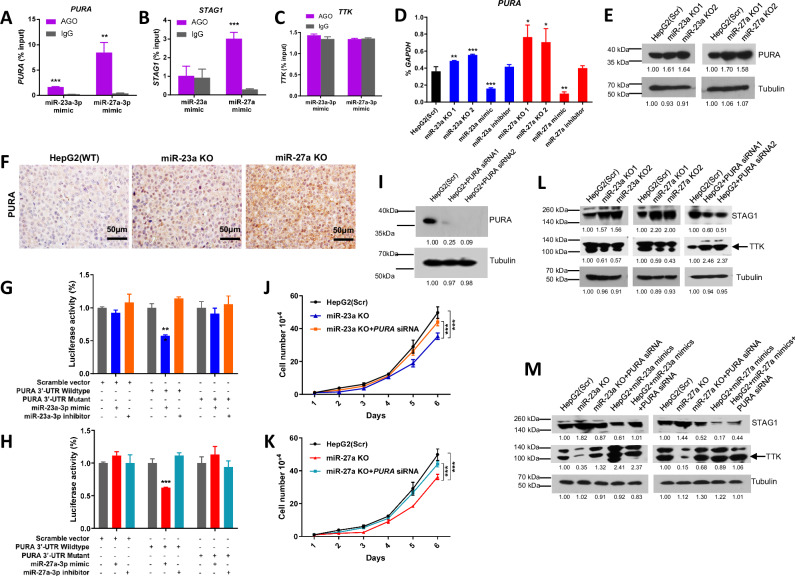
miR-23a/miR-27a-target gene network in HCC cells. **A**–**C** Interaction analysis of miR-23a-5p or miR-27a-5p mimic with the 3′-UTRs of *PURA, STAG1*, and *TTK* mRNA by miRNA/mRNA IP assay of HepG2 cells. Data are presented as means ± SD. ***p < 0.001 by two-tailed *t*-test vs. Scr group. **D** mRNA expression of *PURA* measured by qPCR in Scr, miR-23a KO, or miR-27a KO HepG2 cells treated with or without miRNA mimic or inhibitor. **E** Protein expression of PURA measured by Western blot in Scr, miR-23a KO, or miR-27a KO HepG2 cells. **F** Protein expression of PURA by IHC staining in xenograft tumors injected with Scr, miR-23a KO, and miR-27a KO HepG2 cells in NSG mice. Scale bars=50μm. **G**, **H** Luciferase reporter activity in HepG2 cells co-transfected with Scr vector, wild-type or mutant 3’-UTR *PURA* constructs with mimics or inhibitor for miR-23a-3p or miR-27a-3p. ***p < 0.001 by one-way ANOVA Tukey’s multiple comparisons test *vs*. Scr group. **I** Protein expression of PURA measured by Western blot in Scr cells or cells transfection with *PURA* siRNA. **J**, **K** Cell growth curve in Scr, miR-23a KO, miR-23a KO, miR-27a KO, and miR-27a KO cells transfected with *PURA* siRNA for 6 days. Data are presented as means ± SD. *p < 0.05 and ***p < 0.001 by two-way ANOVA test *vs*. Scr or *PURA* siRNA group. **L** Protein expression of STAG1 and TTK measured by Western blot in Scr, miR-23a KO or miR-27a KO transfected with *PURA* siRNA after 48 h, respectively. **M** Protein expression of STAG1 and TTK measured by Western blot in Scr, miR-23a KO, miR-23a KO + *PURA* siRNA, Scr+miR-23a-3p mimic, Scr+miR-23a-3p mimic+*PURA* siRNA, miR-27a KO, miR-27a KO + *PURA* siRNA, Scr+miR-27a-3p mimic, and Scr+miR-27a-3p mimic+*PURA* siRNA cells, respectively. All experiments were repeated three times.

To investigate the interaction between miR-23a-3p/miR-27a-3p and its target mRNAs, we conducted a miRNA-mRNA interaction analysis using miRNA target immunoprecipitation (IP) with Argonaute proteins (Ago1/2/3) following transfection with the miR-23a-3p/miR-27a-3p mimic in HepG2 cells (Supplementary Fig. S8A). The Ago IP analysis revealed a direct binding of *PURA* mRNA with Ago1/2/3 proteins in the presence of miR-23a/miR-27a compared to the scrambled miRNA control (Fig. 6A), supporting the direct interaction of miR-23a/miR-27a with *PURA* mRNA in HepG2 cells. However, Fig. 6B, C demonstrated that *STAG1* mRNA bound to the sequence of miR-27a but not the sequence of miR-23a, and TTK did not bind to either miR-23a or miR-27a.

To delve deeper into the mechanism of miR-23a/miR-27a-mediated transcriptional regulation of *PURA*, we evaluated the impact of miR-23a/miR-27a KO, mimic, and inhibitor on *PURA* transcription in HepG2 cells. As depicted in Fig. 6D, compared to the scrambled cells, *PURA* expression was higher in miR-23 KO cells, lower in cells transfected with miR-23a-3p mimic, and unchanged in cells transfected with miR-23a-3p inhibitor. Similar results were observed in miR-27a KO cells or cells transfected with mimic or inhibitor (Fig. 6D). Western blot analysis also revealed elevated *PURA* expression in miR-23a KO and miR-27a KO cells compared to scrambled cells (Fig. 6E). Consistent results were confirmed by IHC in miR-23a KO and miR-27a KO xenograft tumors relative to scrambled xenograft tumors (Fig. 6F).

Subsequent sequence alignment analysis identified potential miR-23a-3p/miR-27a-3p targeting sites in the *PURA* 3’- untranslated region (UTR) (Supplementary Fig. S8B). Using a pmiR-luciferase reporting system, we elucidated the post-transcriptional regulation mechanism of *PURA* by miRNAs. Transfection of miR-23a-3p/miR-27a-3p mimics reduced the luciferase activity of the wild-type PURA 3’-UTR (Fig. 6G, H). However, deletion of the miR-23a-3p-targeting sequence (AAUGUGA)/miR-27a-3p-targeting sequence (ACUGUGA) in the *PURA* 3’-UTR rescued the luciferase activity of *PURA* 3’-UTR in HepG2 cells transfected with miR-23a-3p/miR-27a-3p mimics (Fig. 6G, H). Confirming the impact of *PURA* on cell proliferation, we assessed cell growth in miR-23a KO/miR-27a KO cells transfected with *PURA* siRNA (Fig. 6I). As depicted in Fig. 6J, K, the suppression of cell growth in miR-23a/miR-27a KO HepG2 cells was partly rescued after *PURA* silencing.

### Validation of miR-23a/miR-27a-regulated gene network in HepG2 cells

To confirm the regulatory impact of miR-23a/miR-27a on gene expression, Western blot analysis revealed that STAG1 was up-regulated, while TTK was down-regulated in miR-23a KO and miR-27a KO cells, respectively, compared to scrambled control cells (Fig. 6L). Subsequently, we introduced *PURA* siRNAs into scrambled HepG2 cells to investigate whether the regulation of STAG1 and TTK was PURA-dependent. The expression of STAG1 decreased, whereas TTK increased after *PURA* siRNA silencing (Fig. 6L), indicating a PURA-dependent expression pattern for STAG1 and TTK. Further investigation into the miR-23a/miR-27a KO effects on the PURA-STAG1/TTK axes and their regulation in both scramble and miR-23a/miR-27a KO HepG2 cells revealed intriguing insights. For STAG1 expression analysis, miR-23a KO increased the expression of STAG1, but this effect was diminished by *PURA* siRNA in miR-23a KO cells (Fig. 6M). Conversely, miR-23a-3p mimic decreased STAG1 expression, and this effect was rescued by *PURA* siRNA in miR-23a-3p mimic-transfected HepG2 cells (Fig. 6M), indicating a miR-23a-PURA-STAG1 axis. Likewise, miR-27a KO increased STAG1 expression, but this increase was reversed by *PURA* siRNA in miR-27a KO cells (Fig. 6M). However, miR-27a-3p mimic decreased STAG1 expression, and *PURA* siRNA did not rescue this decrease in miR-27a-3p mimic-transfected HepG2 cells (Fig. 6M), suggesting a PURA-independent miR-27a-STAG1 axis. Notably, miR-27a may directly target *STAG1* in the miRNA target IP assay (Fig. 6B), further supporting a PURA-independent miR-27a-STAG1 axis.

### Effect of miR-23a/miR-27 and its cluster in cell migration and epithelial-mesenchymal transition (EMT) in HCC cells

**Fig. 7:**
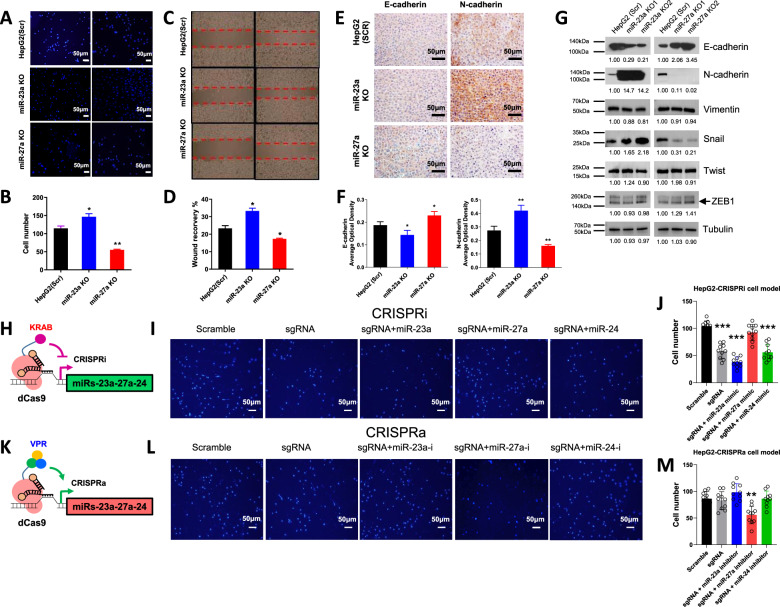
Effect of miR-23a/miR-27a on cell migration and EMT in HCC cells. **A**, **B** Transwell migration assay and quantitative analysis in HepG2 cells. Data are presented as means ± SD. *p < 0.05 and **p < 0.01 by one-way ANOVA Tukey’s multiple comparisons test *vs*. Scr group. **C**, **D** Scratch migration assay and quantitative analysis in HepG2 cells. Data are presented as means ± SD. *p < 0.05 by one-way ANOVA Tukey’s multiple comparisons test *vs*. Scr group. **E**, **F** Protein expression and quantitative analysis of E-cadherin and N-cadherin by IHC staining in xenograft tumors injected with Scr, miR-23a KO, and miR-27a KO HepG2 cells in NSG mice. Scale bars = 50 μm. **G** Protein expression of E-cadherin, N-cadherin, Vimentin, Snail, Twist, ZEB1 measured by Western blot in Scr, miR-23a KO or miR-27a KO HepG2 cells. **H**, **K** Diagram of CRISPRi and CRISPRa system. **I**, **J**, **L**, **M** Transwell migration assay and quantitative analysis in CRISPRi/a HepG2 cells with miRNA mimic or inhibitor. Data are presented as means ± SD. **p < 0.01 and ***p < 0.001 by one-way ANOVA Tukey’s multiple comparisons test *vs*. Scr group. All experiments were repeated three times.

Given the reported role of the miR-23a ~ 27a ~ 24-2 cluster in promoting cell migration [33] and the observed regulation of cell migration by miR-23a-3p/miR-27a-3p mimics (Fig. 1E–H), we investigated the impact of miR-23a/miR-27a KO on cell migration in HepG2 cells. Utilizing scratch assays and Transwell assays, we found that cell migration was accelerated in miR-23a KO cells and decelerated in miR-27a KO cells compared to scrambled control cells (Fig. 7A–D). Additionally, IHC analysis revealed that, in miR-23a KO xenograft tumors, E-cadherin expression decreased while N-cadherin increased (Fig. 7E, F), indicating a role for miR-23a in promoting cell migration and EMT. Conversely, miR-27a KO xenograft tumors exhibited an opposite trend, with increased E-cadherin and decreased N-cadherin expression (Fig. 7E, F), suggesting a distinct role for miR-27a in these processes. Furthermore, to understand the molecular mechanisms underlying the miR-23a ~ 27a ~ 24-2 cluster-regulated migration of HCC cells, we investigated proteins involved in EMT and relevant regulatory molecules. In HepG2 cells, miR-23a KO decreased E-cadherin but increased N-cadherin expression, while miR-27a had the opposite effect (Fig. 7G). Among EMT-related or upstream regulatory molecules, only Snail exhibited inter-group differences, indicating that the miR-23a ~ 27a ~ 24-2 cluster appear to influence EMT through the regulation of Snail (Fig. 7G).

Next, we investigated the impact of manipulating the miR-23a ~ 27a ~ 24-2 cluster on cell migration in both HepG2 and Huh7 cells. Upon Dox induction, CRISPRi inhibited cell migration in HepG2 and Huh7 cells. The introduction of miR-23a-3p mimic further enhanced cell migration, whereas miR-27a-3p mimic reduced cell migration (Fig. 7H–J and Supplementary Figs. S9A–D and S10A–C). Interestingly, miR-24-3p mimic did not significantly alter cell migration. Conversely, CRISPRa did not enhance cell migration. However, miR-27a-3p inhibitor, but not miR-23a-3p and miR-24-3p inhibitors, reduced cell migration (Fig. 7K–M and Supplementary Fig. S9E–H and S10D–F). This differential impact of miR-23a, miR-27a, and miR-24 on cell migration suggests distinct roles for each miRNA within the miR-23a ~ 27a ~ 24-2 cluster, further underscoring the integrative oncogenic function of this cluster in HCC cells.

### Identification of miR-23a/miR-27 signaling axes in HCC cell migration

**Fig. 8:**
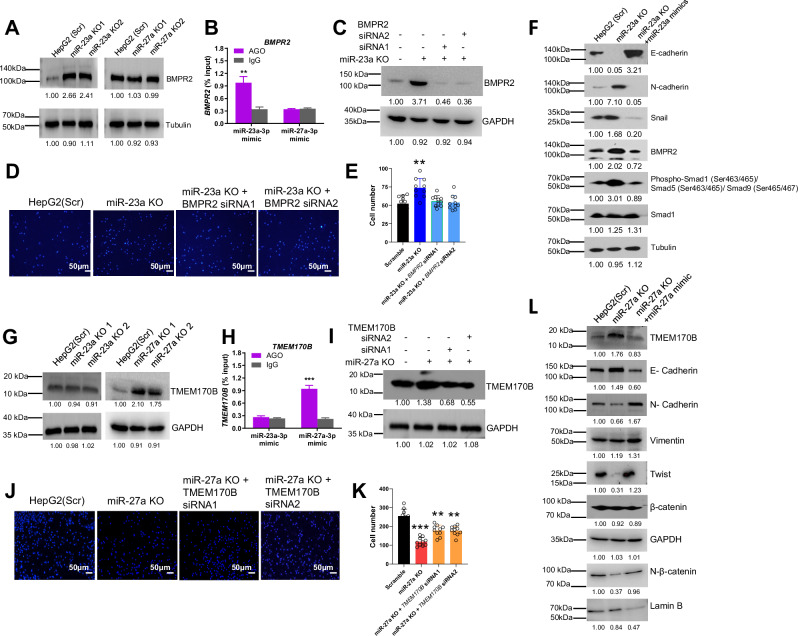
miR-23a/miR-27a-regulated signaling pathways on cell migration and EMT in HCC cells. **A**, **G** Protein expression of BMPR2 and TMEM170B measured by Western blot in Scr, miR-23a KO, or miR-27a KO HepG2 cells. **B**, **H** Interaction analysis of miR-23a-5p or miR-27a-5p mimic with the 3′-UTRs of *BMPR2* and *TMEM170B* mRNA by miRNA/mRNA IP assay of HepG2 cells. Data are presented as means ± SD. ***p < 0.001 by two-tailed *t*-test vs. Scr group. **C**, **I** Protein expression of BMPR2 and TMEM170B measured by Western blot in miR-23a KO or miR-27a KO HepG2 cells treated with Scr or siRNA. **D**, **E**, **J**, **K** Transwell migration assay and quantitative analysis in in miR-23a KO or miR-27a KO HepG2 cells treated with Scr or siRNA. Data are presented as means ± SD. **p < 0.01 and ***p < 0.001 by one-way ANOVA Tukey’s multiple comparisons test vs. Scr group. **F** Protein expression of BMPR2, E-cadherin, N-cadherin, Snail, p-Smad1, Smad1 measured by Western blot in Scr, miR-23a KO, and miR-23a KO+miR-23a-3p mimic cells. **L** Protein expression of TMEM170B, E-cadherin, N-cadherin, Vimentin, Twist, β-catenin, and nclear (N)-β-catenin measured by Western blot in Scr, miR-27a KO, and miR-27a KO+miR-27a-3p mimic cells. All experiments were repeated three times.

Upon re-analyzing the candidate target genes (Fig. 4C), we identified *BMPR2* as a target gene of miR-23a that regulates EMT through the BMP-Smad-Snail signaling pathway [34]. In HepG2 cells, we observed up-regulation of BMPR2 in miR-23a KO cells, while no significant difference in BMPR2 was observed in miR-27a KO cells compared to scrambled cells (Fig. 8A). miRNA target IP further confirmed the direct binding of miR-23a, but not miR-27a, to the *BMPR2* 3’-UTR (Fig. 8B). Silencing *BMPR2* using siRNAs rescued the enhanced cell migration observed in miR-23a KO HepG2 cells (Fig. 8C–E). Transfecting miR-23a-3p mimics into miR-23a KO cells rescued the expression of E-cadherin, reduced the expression of N-cadherin, and led to decreases in BMPR2, Snail, and phospho-Smad1 (Fig. 8F). These findings suggest a miR-23a-BMPR2-Smad-Snail axis in the regulation of EMT signaling.

Likewise, we identified *TMEM170B* as a potential target gene of miR-27a that regulates EMT via the TMEM170B-Twist-β-catenin signaling pathway [35]. Testing the effect of miR-23a/miR-27a on this EMT signaling in HepG2 cells, we found that TMEM170B was up-regulated in miR-27a KO cells but not in miR-23a KO cells compared to scrambled cells (Fig. 8G). miRNA target IP validated the direct binding of miR-27a, but not miR-23a, to the *TMEM170B* 3’-UTR (Fig. 8H). Silencing *TMEM170B* using siRNAs rescued the reduced cell migration observed in miR-27a KO HepG2 cells (Fig. 8I–K). Transfecting miR-27a-3p mimics into miR-27a KO cells rescued the expression of TMEM170B and E-cadherin, induced the expression of N-cadherin and vimentin, and led to increases in Twist and nuclear β-catenin (Fig. 8L). These results suggest a miR-27a-TMEM170B-Twist-β-catenin axis in the regulation of EMT signaling.

## Discussion

The utilization of CRISPR/Cas9 for genetic and epigenetic editing holds promise as a tool for investigating the regulation and function of clustered miRNAs [36]. In this study, we employed CRISPR KO, CRISPRi, and CRISPRa approaches to dissect the functional role and underlying signaling of the miR-23a ~ 27a ~ 24-2 cluster in HCC cells. Either miR-23a KO or miR-27a KO resulted in reduced cell growth both in vitro and in vivo. Moreover, the endogenous miRNAs within the miR-23a ~ 27a ~ 24-2 cluster were effectively regulated by CRISPRi/a. Notably, using the CRISPRi/a approach, we identified an integrated oncogenic role of the miR-23a ~ 27a ~ 24-2 cluster in the proliferation of HCC cells. Functional analysis revealed that miR-23a KO and miR-27a KO induced cell cycle arrest at the G2/M phase by reducing CDK1/cyclin B activation in HCC cells. Furthermore, employing a high-throughput RNA-seq approach with miRNA target prediction, we identified the miR-23a/miR-27a-regulated gene network. Various analyses validated that miR-23a/miR-27a co-target *PURA* directly to promote cell proliferation in HepG2 cells. In addition, miR-23a and miR-27a exhibited opposite roles in cell migration and EMT. However, an integrated analysis by CRISPRi/a suggested an oncogenic role of the miR-23a ~ 27a ~ 24-2 cluster in cell migration may occur through an interaction of miR-23a-BMPR2-Smad-Snail axis and miR-27a-TMEM170B-Twist-β-catenin axis in HCC cells (Supplementary Fig. S11).

Given the involvement of miRNAs in the miR-23a ~ 27a ~ 24-2 cluster in various functions and signaling pathways in HCC [5], depicting their integrative role and complete regulatory network is challenging. However, our established endogenous miRNA controllable system offers a comprehensive platform for identifying the functional role and underlying signaling of clustered miRNAs. In this study, we used CRISPR KO to assess the potential role and gene network of individual mature miR-23a/miR-27a in HCC cells. However, due to the joint transcription of miRNAs in the miR-23a ~ 27a ~ 24-2 cluster, miR-23a KO disrupted the expression of miR-27 and miR-24, while miR-27a KO affected the expression of miR-24 but unlikely changed the expression of miR-23a. Thus, the CRISPR KO approach is not ideal for defining the role of individual miRNAs from the miRNA cluster. Recognizing the complexity and interplay among these miRNAs, we subsequently employed CRISPR epigenetic editing by CRISPRi/a systems to dissect their functional roles and interactions more comprehensively. Using CRISPRi/a, we addressed the role and gene network of the miR-23a ~ 27a ~ 24-2 cluster in HCC cells. Combining CRISPRi/a-mediated endogenous miRNA regulation with exogenous miRNA mimic and inhibitor intervention, we validated the role and gene network of individual mature miR-23a/miR-27a in HCC cells. Additionally, both the miR-23a ~ 27a ~ 24-2 and miR-23b ~ 27b ~ 24-1 clusters include miR-24 and encode the pri-miRNA transcript that composes miR-24 [4]. To address the role of miR-24, CRISPR KO or CRISPRi/a should target both clusters for transcriptional regulation of miR-24. However, this approach may introduce more complexity to the CRISPR system, off-target effects, or complicate functional analysis between the two clusters. Recently, we have developed a flexible CRISPR/dCas9-based platform for complex gene regulation, allowing independent control of the expression of different genes (repression and activation) within the same cell, using two different S. pyogenes (Sp)-dCas9 and S. aureus (Sa)-dCas9 [37]. This dual CRISPR platform for repression and activation may serve as an ideal tool for our future studies to distinguish the roles of the two miRNA clusters, including miR-24, within the same HCC cell.

Our gene network analysis revealed that the enriched gene set in both miR-23a KO and miR-27a KO groups was predominantly associated with the cell cycle signaling network. Further analysis of the enriched genes in the KEGG map showed that the majority of these genes were concentrated in the G2/M phase of the cell cycle. This finding was consistent with the observed changes in cell cycle regulatory proteins at the G2/M phase, such as CDK1/cyclin B [38]. Using endogenous miR-23a ~ 27a ~ 24-2 controllable CRISPRi/a cell models, we confirmed dynamic changes in genes enriched in the G2/M phase based on miR-23a ~ 27a ~ 24-2 up-/down-regulation. The miR-23a ~ 27a ~ 24-2 cluster directly/indirectly regulated the gene network, including *PURA*, *STAG1*, and *TTK*, in HCC cells. In HepG2 cells, miR-23a and miR-27a induced cell cycle progression by directly targeting *PURA*, implicating cancer cell proliferation by inhibiting cell cycle progression at G1-S or G2-M checkpoints [39], resulting in increased HCC cell growth. MiR-23a and miR-27a targeted specific sites in the 3’-UTR of *PURA* to regulate post-transcription of *PURA*. Notably, the miR-23a and miR-27a-mediated proliferation phenotype was partly retrieved by *PURA* siRNA. However, another study also suggested that miR-23a may promote G1/S cell cycle transition in HCC [8]. Although in our study, a few relevant genes of the G1/S phase were enriched in the miR-23a KO group, we did not observe an apparent change in cell cycle progression and proteins of the G1/S phase. Furthermore, our data support the notion that miR-27a may play a dominant role in the regulation of G2/M cell cycle transition. Additionally, other studies suggest that miR-23a/miR-27a regulate apoptosis in HCC cells [6, 40], but our analysis showed no significant change in apoptosis after miR-23a KO or miR-27a KO. Thus, our data suggest that miR-23a/miR-27a may synergistically induce cell cycle progression, promoting cell proliferation in HCC cells.

The members of the miR-23a ~ 27a ~ 24-2 cluster integrally inhibit the aggressiveness of breast cancer cells by targeting *NCOA1*, *NLK*, and *RAP1B* [41]. Additionally, the c-MYC-regulated miR-23a ~ 27a ~ 24-2 cluster promotes cell invasion and hepatic metastasis of breast cancer by targeting *SPRY2* [33]. Our CRISPR analysis is the first to provide an integrated and dynamic analysis supporting the idea that the miR-23a ~ 27a ~ 24-2 cluster promotes cell migration and EMT through a complex gene network in HCC cells. However, individual miR-23a/miR-27a have been reported to be associated with both tumor-promoting [42–49] and tumor-suppressing [18, 50–52] activities, depending on the specific context and cancer type. In this study, there are opposing effects between miR-23a (repression) and miR-27a (promotion) on cell migration and EMT in HCC cells, suggesting that miR-23a/miR-27a may play distinctive roles in tumor progression and metastasis through two different signaling axes, such as the miR-23a-BMPR2-Smad-Snail axis and the miR-27a-TMEM170B-Twist-β-catenin. Given an oncogenic role of the miR-23a ~ 27a ~ 24-2 cluster in cell migration and EMT, miR-27a may play a predominant role in cell migration and EMT of HCC cells. In addition, an early study has reported that, through miRNA mimic and inhibitor, miR-27a-3p inhibits the growth and metastasis of HCC cells [18]. Specifically, miR-27a-3p inhibits the cell migration, invasion, and EMT of HCC cells. Conversely, through miRNA KO with mimic and inhibitor, we identified an oncogenic role of miR-27a-3p in cell migration and EMT of HCC cells. The different results may be attributed to the potential effect of CRISPR KO on the transcription of the miR-23a ~ 27a ~ 24-2 cluster and its individual members, including miR-24. However, through CRISPRi/a with miRNA mimic and inhibitor, we validated an oncogenic role of miR-27a-3p in HCC cell migration. In addition, there is a significant impact in survival outcomes associated with miR-27a and miR-24 but not miR-23a, despite being regulated by the same promoter. However, these results are consistent with our experimental data that miR-23a and miR-27a play contrasting roles in cell migration. While miR-23a inhibits migration, miR-27a promotes it. This differential effect on cell migration may potentially explain the disparate survival outcomes observed between miR-23a and miR-27a.

In conclusion, we developed an integrated CRISPR genetic and epigenetic approach to investigate the functional role and underlying signaling of the miR-23a ~ 27a ~ 24-2 cluster in HCC cells. Our CRISPR analysis offers a valuable research tool for dissecting the complex functions and underlying gene network of an endogenous miRNA cluster. Furthermore, this CRISPR approach provides new avenues for intervening in miRNA clusters, enabling an integrated analysis to determine the specific roles of individual miRNAs within the cluster.

## Materials and methods

### Cell lines

The hepatocellular carcinoma cell lines HepG2 and Huh7, as well as the human embryonic kidney cell line HEK293T, were procured from the American Type Culture Collection (ATCC, Rockville, Maryland). These cell lines were cultured for less than 6 months, authenticated through examination of morphology and growth characteristics, and confirmed to be mycoplasma-free. Short tandem-repeat analysis for DNA fingerprinting was also employed to verify the cell lines. All cell lines were cultured in Dulbecco’s Modified Eagle’s medium (DMEM) supplemented with 10% fetal bovine serum (FBS) under standard conditions of 37 °C and 5% CO_2_.

### Antibodies

Antibodies specific for the following proteins were used as primary antibodies for Western blot or IHC: Beta catenin (ab32572, 1:5000, Abcam, Cambridge, MA), beta IV Tubulin (ab179509, 1:5000, Abcam), CDC27 (A1954, 1:1000, Abclonal, Woburn, MA), BMPR2 (A16778, 1:1000, Abclonal), CDK1 (626901, 1:1000, Biolegend, San Diego, CA), CDK2 (643901, 1:1000, Biolegend), CDK4 (2906, 1:1000, Cell Signaling, Danvers, MA), c-Myc (5605, 1:1000, Cell Signaling), Cyclin A (644001, 1:1000, Biolegend), PURA (A9296, 1:1000 for Western blot and 1:100 for IHC, Abclonal), Cyclin B (647902, 1:1000 for Western blot and 1:100 for IHC, Biolegend), Cyclin D1 (ab134175, 1:5000, Abcam), Cyclin E (sc-247, 1:1000, Santa Cruz Biotechnology, Dallas, TX), E-cadherin (3195, 1:1000 for Western blot and 1:100 for IHC, Cell Signaling), GAPDH (2118, 1:5000, Cell Signaling), Ki67 (ab15580, 1:5000 for Western blot and 1:200 for IHC, Abcam), Lamin B (sc-374015, 1:5000, Santa Cruz Biotechnology), N-cadherin (844702, 1:1000 for Western blot and 1:100 for IHC, Biolegend), p21 (2947, 1:1000, Cell Signaling), p53 (sc-126, 1:1000, Santa Cruz Biotechnology), phospho-cdc2 (Tyr15) (4539, 1:1000 for Western blot and 1:100 for IHC, Cell Signaling), phospho-Smad1/Smad5/Smad9 (13820, 1:1000, Cell Signaling), Smad1 (6944, 1:1000, Cell Signaling), Snail (ab167609, 1:5000, Abcam), STAG1 (A13715, 1:1000, Abclonal), TMEM170B (NBP2-33739, 1:1000 NOVUS Biologicals, Centennial CO), TTK (A2500, 1:1000, Abclonal), Twist (ab175430, 1:5000, Abcam), Vimentin (sc-6260, 1:1000, Santa Cruz Biotechnology), and ZEB1 (3396, 1:1000, Cell Signaling).

### Establishment of CRISPR KO and CRISPRi/a cell models

The Benchling CRISPR design tool (San Francisco, CA, https://benchling.com) was employed for designing sgRNAs. The CRISPR/Cas9 editing targeted the pri-miR-23a ~ 27a ~ 24-2 region. Pair sgRNAs with high specificity and efficiency scores were chosen at two flanking sites of the mature miR-23a or miR-27a sequence. All sgRNAs were assessed using the Cas-OFFinder off-target searching tool (South Korea, http://www.rgenome.net/cas-offinder). To mitigate off-target effects, potential off-target regions underwent PCR and Sanger sequence analysis (Supplementary Table S6). The sequences of the pair sgRNAs targeting miR-23a or miR-27a are listed in Supplementary Table S7.

The pair sgRNA oligos were annealed by slow cooling from 95 °C down to 10 °C and then ligated to BbsI-digested pSpCas9(BB)-2A-GFP (PX458) vector (Addgene, Cambridge, MA). The integrity of the miR-23a- and miR-27a-targeted-Cas9 constructed vectors was confirmed by DNA sequencing. The constructed vector with the miR-23a or miR-27a targeting sequence, or the PX458 empty vector, was transfected into cells using Lipofectamine 3000 (Thermo Fisher Scientific). miR-23a or miR-27a KO colonies were confirmed by qPCR and Sanger sequencing after single-cell sorting (BD FACSMelody™ Cell Sorter) with GFP. All selected colonies of miRNA scramble and KO cells were validated by PCR and Sanger sequence analysis to exclude off-target effects in potential off-target regions of sgRNAs, as described previously [53, 54].

CRISPRi/a epigenetic editing technology was used to establish the endogenous miR-23a ~ 27a ~ 24-2 controllable cell models. The carrier of sgRNAs for the CRISPR nuclease-dead Cas9 (dCas9) system is pSLQ2837 pLenti U6-spsgTRE3G CMV-mIFP (a gift from Stanley Qi lab, Stanford University). pSLQ1922-dCas9-GFP-KRAB-Zeocin and pSLQ1932-dCas9-GFP-VPR-Zeocin (gift from Stanley Qi lab) are piggyBac (PB)-based DNA constructs with dCas9 elements (Supplementary Table S8). The promoter region of pri-miR-23a ~ 27a ~ 24-2 was the candidate target region for CRISPRi and CRISPRa, respectively.

Four sgRNAs (Supplementary Table S7) were designed around the −50 to +300 bp of the transcription start site (TSS) of the miR-23a ~ 27a ~ 24-2 cluster for CRISPRi (CRISPR/dCas9-KRAB-mediated transcriptional repression). Similarly, four sgRNAs were designed around the −400 to −50 bp of the TSS for CRISPRa (CRISPR/dCas9-VPR-mediated transcriptional activation). Then, we established various cell models as follows: (1) Vectors with CRISPRi or CRISPRa system were transfected with PB transposase vector into cells, as previously described [55]. (2) sgRNA vectors were transduced by lentivirus-mediated transduction into CRISPRi or CRISPRa transfected cells, followed by single-cell sorting with GFP (for dCas9) and mIFP (for sgRNA). (3) A stable cell line was established after zeocin (100 μg/ml, Invitrogen) selection for two weeks.

### Cell transfection for virus production

A total of 6.0 × 10^6^ HEK293T cells were seeded onto a 10 cm dish. A transgene (21 μg), pCMV-Gag-Pol vector (21 μg), and pCMV-VSV-G-poly A vector (10.5 μg) were mixed with ddH_2_O to a final volume of 945 μl (DNA mix). To this mix, 105 μl of CaCl_2_ (2.5 M) and 1050 μl of 2 × HBSS were added, and the solution was incubated for 3 min. The 2100 μl solution was then added to the cells and incubated at 37 °C with 5% CO_2_ for 8–10 h. Following incubation, the media were removed, and the cells were cultured in 5% FBS + DMEM for at least 48 h. The culture containing the virus was then collected for further experiments.

### Analyses of cell proliferation, cell cycle progression, and apoptosis

miR-23a/miR-27a KO cells or scrambled control cells were seeded onto a 6-well plate with a density of 5 × 10^4^. Cell morphology, viability, and numbers were monitored microscopically over 6 days. Additionally, the optical density of MTT (3-[4,5-dimethylthiazol-2-yl]-2,5 diphenyl tetrazolium bromide, Sigma-Aldrich, St. Louis, MO) was measured daily. For the colony formation assay, cells were seeded at 5 × 10^3^ cells/ml in triplicate in a 6-well plate. After three weeks, cells were stained with 0.125% crystal violet, and colonies (>20 cells) were photographed and counted under the microscope. In the soft agar assay, a 3.2% sterile stock agarose (Sigma-Aldrich) solution was prepared with ddH_2_O, and a 0.8% base agarose layer was created using cell culture medium. Cells (2 × 10^3^ cells per 6-well plate) were trypsinized, counted, and used to prepare a 0.48% upper agarose layer. Each well received 1 ml of cell culture medium. After a 14-day incubation, colonies were stained with 4% formaldehyde and 0.005% crystal violet. Colonies (>50 cells) were then photographed and counted under the microscope.

Cell-cycle progression was determined by flow cytometry using propidium iodide (PI) staining (50 μg/ml, BD Biosciences, Franklin Lakes, NJ). This analysis was conducted after a 48-h starvation of cells, followed by serum stimulation at 0, 8, and 22 h. Additionally, cell-cycle progression was assessed using flow cytometry with BrdU antibody and 7AAD at 6 and 14 h after starvation, following the manufacturer’s protocol (Phase-Flow BrdU Kit, Biolegend).

Apoptosis analysis was performed by flow cytometry 24 h after seeding, utilizing the Apoptosis Detection Kit with Annexin V and 7AAD from Biolegend. In the case of apoptosis induction by H_2_O_2_, cells were treated with 0.1 mM H_2_O_2_ for 15 min, and the samples were subsequently subjected to flow analysis.

### Soft agar assay

To determine anchorage-independent cell growth, a 6-well plate was prepared with a solidified 0.6% agarose bottom layer. A 0.3% agarose solution containing 1000 cells was then added as the top layer. Following a 2–4 week incubation period in a standard cell culture incubator, allowing for the formation of colonies within the three-dimensional agarose matrix, the resulting colonies were stained using crystal violet. Quantitative analysis involved colony counting and size measurement using microscopy with ImageJ analysis software.

### Cell migration assays

A total of 4 × 10^4^ cells were cultured in 0.2% FBS + DMEM and seeded onto transwell inserts (pore size, 8 μm, CORNING, Corning, NY). Next, 600–800 μL of 10% FBS + DMEM was added to the lower well of the 24‐transwell plate. To mitigate the effects of cell proliferation on migration analysis, cells were pre-treated with 10 μg/ml mitomycin C (Sigma-Aldrich) for 2 h prior to the Transwell migration assay. The transwell insert was then placed into the lower well and incubated for 18–22 h. Following the incubation, the transwell polycarbonate membrane was fixed in 4% formaldehyde for 15 min. Cells that did not migrate across the membrane were gently removed with a cotton swab. The membrane was carefully cut with a scalpel and stained with DAPI for 10 min. For the scratch assay, 1 × 10^6^ cells were seeded onto a 6-well plate. Pipette tips were used to create a wound by scratching the cells. The wound was photographed at 0 and 24 h, and the wound-healing rates were calculated using software.

### qPCR

Total RNA was extracted from cultured cells using Trizol reagent (Thermo Fisher Scientific) as directed by the manufacturer. For miRNA expression profiling, a 20-μl reverse transcription reaction, utilizing 5 μl of RNA and miScript II RT Kits (QIAGEN, Germantown, MD), adhered to the manufacturer’s protocol. Subsequently, 2 μl of cDNA acted as the template for real-time PCR, executed on a LightCycler 480 Real-Time PCR System (Roche Applied Sciences, Indianapolis, IN) with miScript SYBR Green PCR kits (QIAGEN). Incubation of the reaction mixtures occurred in 96-well optical plates at 95 °C for 10 min, succeeded by 40 cycles of 95 °C for 15 s and 60 °C for 1 min. Post-reaction, cycle threshold (Ct) data were determined using fixed threshold settings, and mean Ct values were derived from triplicate PCRs. Employing the 2^(-ΔCt) method for quantification, *GAPDH* or *U6* were selected as a reference gene for mRNA or miRNA, respectively. The 2^(-ΔCt) method is a relative quantification that compares the Ct values of a target gene to those of a reference gene. In this method, the difference in Ct values between the target gene (ΔCt) and the reference gene is calculated to assess the relative expression level of the target gene. The qPCR primer sequences are listed in Supplementary Table S7.

### Western blots

Western blotting was performed as previously described [56, 57]. Briefly, cell lysates containing 50–100 μg of protein were prepared and subjected to 10% SDS-polyacrylamide gels. Proteins were transferred to PVDF membranes, which were incubated in 5% non-fat milk for 1 h and overnight at 4 °C in 0.25% non-fat milk containing specific primary antibodies. The membranes were subsequently incubated at room temperature for 1 h in 0.25% non-fat milk with anti-rabbit/mouse IgG HRP-linked secondary antibody (1:5000, Cell Signaling). Enhanced chemiluminescence reagents were utilized, and the membranes were then exposed to X-films for 1–5 min.

### Luciferase assay

The pmirGLO luciferase reporter vector (Promega) was utilized to construct DNA fragments from the 3’-UTR of *PURA* (Transcript: ENST00000331327.3) by NotI and XbaI (New England Biolabs, Ipswich, MA) digestion, following the manufacturer’s protocol. The primers for the constructed vectors and mutagenesis of *PURA* are listed in Supplementary Table S7. In brief, 1 × 10^4^ cells were seeded onto a 96-well plate, cultured with DMEM + 10% FBS, incubated at 37 °C and 5% CO_2_ overnight, and transiently co-transfected with constructed vectors (pmirGLO-PURA-3’ UTR, pmirGLO-PURA-3’UTR-Mut) or empty pmirGLO vector and miR-23a-3p/miR-27a-3p mimics (50 nmol/L) or inhibitor (100 nmol/L) using Lipofectamine 3000. After 48 h of transfection, substrate reagents (Promega) were added to the wells and incubated for 10 min. Subsequently, the luciferase activity was assessed with a Veritas Microplate Luminometer (Turner BioSystems, Sunnyvale, CA). Following this, stop reagents were added, and the luciferase activity was re-evaluated.

### miRNA/mRNA IP assay

miRNA/mRNA immunoprecipitation was performed using miRNA Target IP kits (Active Motif, Carlsbad, CA). Briefly, cells (1 × 10^7^) were seeded onto a 10 cm dish and transfected with scrambled control or miRNA mimic (50 nmol/L) or inhibitor (100 nmol/L) using Lipofectamine 3000 for 24 h. Cell lysates were collected from each sample using 150 μL complete lysis buffer, and 10 μL of cell lysate was marked as the input. Samples and inputs were incubated on ice for 10 min and then at −80 °C for 2 h. Protein G magnetic beads (50 μL) were blocked with 200 μL BSA for 1 h and then placed on a magnet to pellet the beads. The beads were washed twice with wash buffer. Ago1/2/3 antibody (2.5 μL) or a negative control anti-IgG antibody (12.5 μL) was added to each tube and incubated for 30 min at room temperature. Subsequently, samples and inputs were added to the protein G magnetic beads-antibody complex and incubated overnight at 4 °C. After proteinase K digestion (55 °C for 30 min) and RNA precipitation, RT-qPCR was used to analyze if the candidate gene target could bind to miRNA. The results of Ago-IP were normalized to that of the negative control IgG-IP.

### IHC staining

Dako retrieval buffer (Agilent, Santa Clara, CA) was employed for antigen retrieval, and the ABC detection system (Vector Laboratories, Burlingame, CA) was utilized for immunostaining. Specific primary antibodies were incubated with tissues overnight at 4 °C. The secondary antibody used was goat anti-mouse/rabbit IgG (Invitrogen, 1:200). The staining process was completed following the manufacturer’s protocol of VECTASTAIN ABC Kits (Vector Laboratories). Hematoxylin was applied last for the staining of the nucleus. Protein expression of Ki67 and p-CDK1 (Tyr15) in the nuclei were classified as negative or positive and quantitated by positive cell number. Protein expression of E-cadherin and N-cadherin in the plasma membrane or cytoplasm were quantitated by average optical density. Protein expression of cyclin B in the cytoplasm and nuclei were quantitated by H-score, which was classified as negative if <10% of cells within tumor areas were stained or positive if 10%–100% were stained. Then, the percentage of positive tumor cells per slide (10% to 100%) was multiplied by the dominant intensity pattern of staining (1, weak; 2, moderate; 3, intense); the overall scores ranged up to 300 H-scores. All slides were examined by pathologists in a blinded fashion.

### In vivo tumor xenograft model

Mice (n = 7/group) were randomly grouped for treatment. Scramble, miR-23a KO, and miR-27a KO HepG2 cells (100 μl, 5 × 10^7^/ml) were subcutaneously injected into the left flanks of 6-week-old NSG mice (Jackson Laboratories, Bar Harbor, ME). Tumor growth was monitored every two or three days. At 31 days after injection, NSG mice were sacrificed, and tumor volume and weights were measured. All experiments were conducted in accordance with accepted standards of animal care and approved by the Institutional Animal Care and Use Committee (IACUC) of The University of Alabama at Birmingham.

### RNA-seq

Utilizing the TruSeq Stranded mRNA Library Prep Kit (Illumina, San Diego, CA), RNA libraries were prepared in accordance with the manufacturer’s protocol. The integrity check employed an Agilent 2200 Tapestation instrument. First-strand cDNA synthesis utilized random hexamers and ProtoScript II Reverse Transcriptase (New England Biolabs, Ipswich, MA). Subsequently, libraries underwent normalization, pooling, and cluster and pair read sequencing on a HiSeqX10 instrument (Illumina) for 150 cycles, following manufacturer instructions. The generated RNA-seq data have been deposited in NCBI GEO under Accession no. GSE199332.

### Bioinformatic analysis

Differential expression analysis of genes (DEGs) was conducted utilizing fold change and q-value. miRNA expression data from cancer and normal tissue samples were sourced from Oncomir (www.oncomir.umn.edu) and TCGA. To generate and visualize a functionally grouped network of terms and pathways for extensive gene clusters, the ClueGO Cytoscape plug-in (apps.cytoscape.org/apps/cluego) was employed. Gene clusters were imported from a text file or interactively from the Cytoscape network. For in-depth analysis, KEGG (www.genome.jp/kegg/) analysis of the identified DEGs was executed to retrieve interacting genes and proteins (string-db.org/). The Cytoscape software (cytoscape.org/) facilitated the construction of an interaction network diagram for the DEGs.

### Statistical analysis

Continuous variables were summarized using mean, standard deviation (SD), and median values. The distribution of samples was assessed using the one-sample Kolmogorov-Smirnov test. For samples with normal distributions, a two-tailed *t*-test compared means between two groups. In cases of non-normal distributions, the Mann–Whitney *U* test was employed to compare median variation between two groups. One-way analysis of variance (ANOVA) Tukey’s multiple comparisons test was tested for differences among at least three groups, while two-way ANOVA was used to assess the influence of two categorically independent variables on one dependent variable. Survival curves were estimated using the Kaplan–Meier method and compared statistically using the log rank test. Seven animals per group were used to achieve a minimum of 80% power at 0.05 significance level to detect 2-fold changes in tumor growth for 5 groups assuming the coefficient of variation on the original scale is 0.5 by Student’s 2-tailed t-tests. SAS (Version 9.4) and GraphPad Prism (Version 10) software were utilized for data analysis.

### Supplementary information

Supplementary Figures

Supplementary Tables

## Data Availability

The RNA-seq data discussed in this publication has been deposited into the Gene Expression Omnibus (GSE199332). All study data are included in the article and Supplementary Materials.
