# Supplementary material for: CRISPR-based dissection of microRNA-23a ~ 27a ~ 24-2 cluster functionality in hepatocellular carcinoma
Source: Oncogene. 2024 Aug 7;43(36):2708–21. doi: 10.1038/s41388-024-03115-z (PMC11364504; doi:10.1038/s41388-024-03115-z)
Supplement: Supplementary file 1 — Supplementary Figures [file 41388_2024_3115_MOESM1_ESM.pdf]

## Supplementary Figures

# CRISPR-Based Dissection of microRNA-23a~27a~24-2 Cluster Functionality in Hepatocellular Carcinoma

Mengying Cui, Zhichao Liu, Shuaibin Wang, Sejong Bae, Hua Guo, Jiangbing Zhou, Runhua Liu, and Lizhong Wang

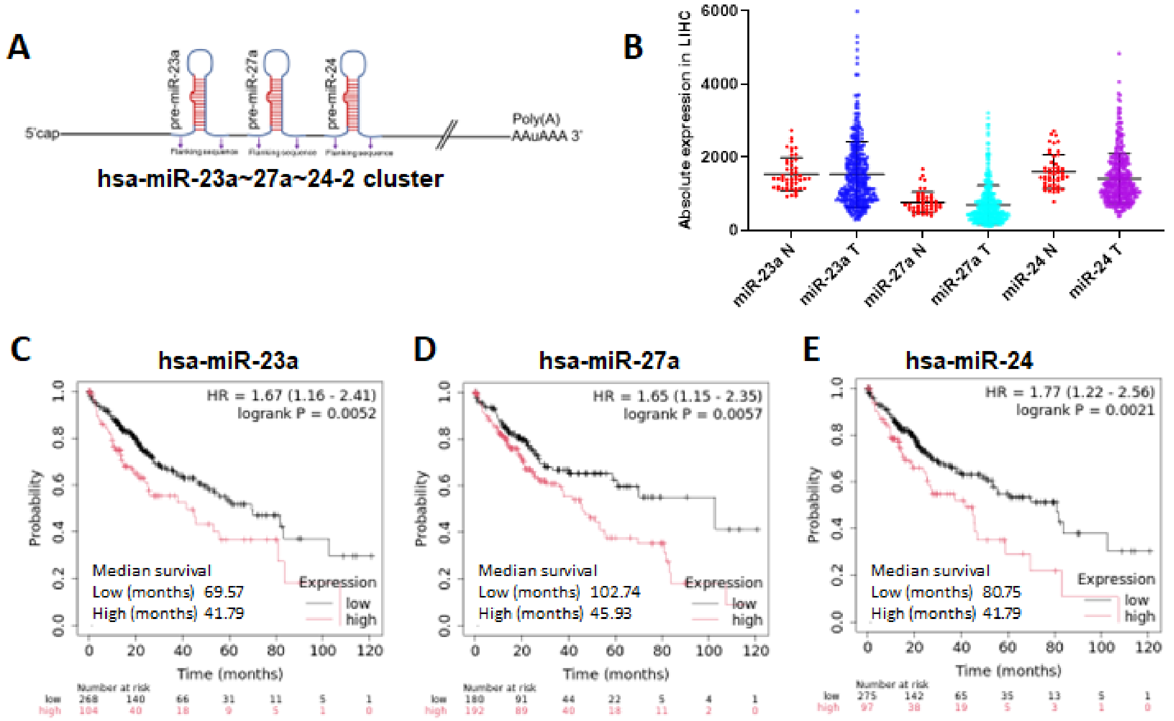


**Figure S1. A,** Schematic representation of endogenous has-miR-23a~27a~24-2 cluster. **B,** Expression of miR-23a, miR-27a, and miR-24 in human primary liver hepatocellular carcinoma (LIHC) using TCGA dataset. Data are presented as means ± SD. Normal tissues (N) n=51; tumor tissues (T) n=375. **C-E,** cKaplan–Meier survival curves for miR-23a, miR-27a, and miR-24 expression with low and high levels, according to the median of the miRNA expression, in in the KM-plotter analysis. The RNA-seq data from 372 liver cancer patients have been evaluated in KM-plotter overall survival (OS) analysis. For has-miR-23a, the median OS times were 69.57 months for the low expression group (n=268) and 41.79 months for the high expression group (n=104). For has-miR-27a, the median OS times were 102.74 months for the low expression group (n=180) and 45.93 months for the high expression group (n=192). For has-miR-24, the median OS times were 80.75 months for the low expression group (n=275) and 41.79 months for the high expression group (n=97). Based on Sci Rep. 2018, 8: 9227 (PMID: 29907753), the RNA-seq data from 372 liver cancer patients in the KM-plotter analysis are from the TCGA Illumina miRNA-Seq data. *p* value by log rank test.

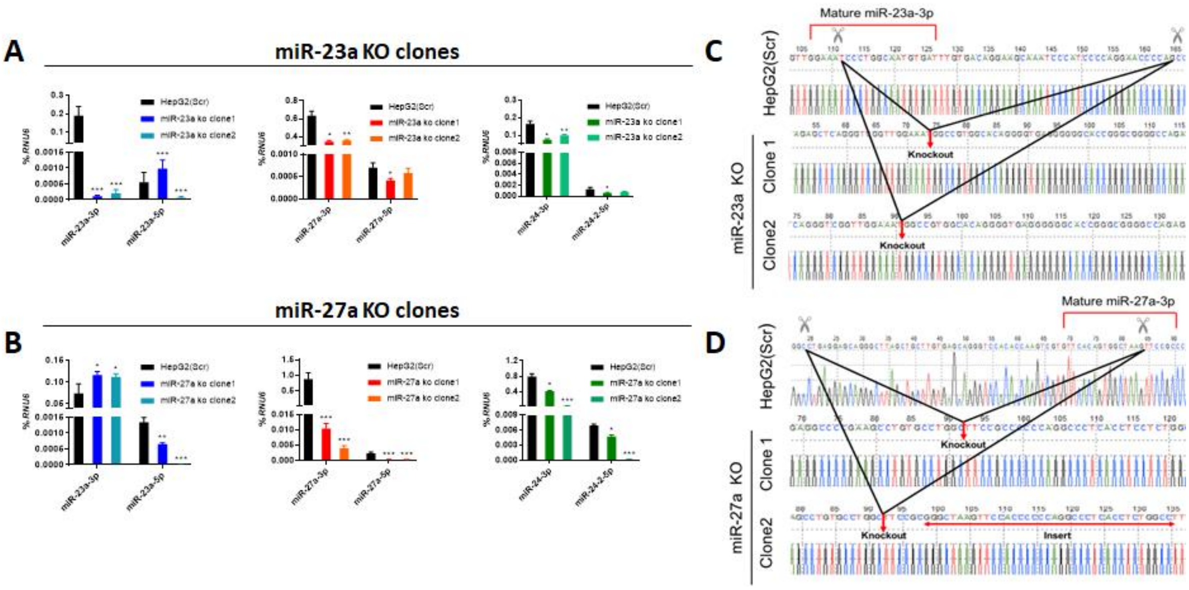


**Figure S2. Establishment of miR-23a/miR-27a knockout (KO) HepG2 cell models. A-B,** Expression of miR-23a-3p/5p, miR-27a-3p/5p, miR-24-3p/5p in miR-23a/miR-27a KO HepG2 cells detected by qPCR. Data are presented as means ± SD. * $p < 0.05$, ** $p < 0.01$, and *** $p < 0.001$ by one-way ANOVA followed by protected least-significant difference test *vs*. Scr group. **C-D,** Sanger sequencing for miR-23a/miR-27a KO HepG2 cell clones. The red line indicates DNA deletion within miR-23a/miR-27a. Scr, scramble control; KO, knockout. All experiments were repeated three times.

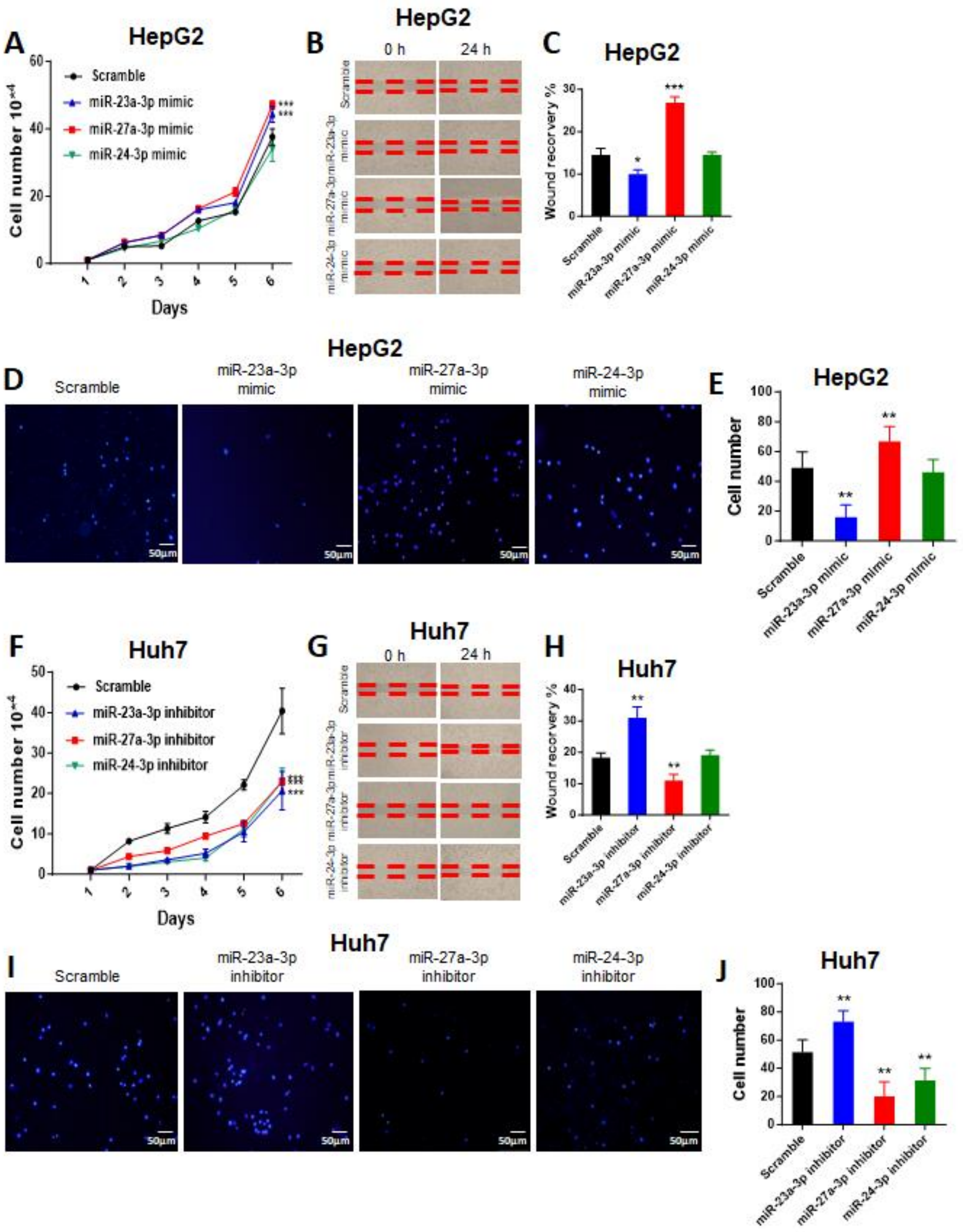


**Figure S3. Effect of miRNAs in the miR-23a~27a~24-2 cluster on cell proliferation and migration of HCC cells. A** and **F,** Cell growth curve in HepG2 and Huh7 cells treated with scramble (Scr), miRNA mimic, and miRNA inhibitor for 6 days. Data are presented as means ± SD. *** $p < 0.001$ by two-way ANOVA test *vs*. Scr group. **B-C** and **G-H,** Scratch migration assay and quantitative analysis in HepG2 and Huh7 cells. Data are presented as means ± SD. * $p < 0.05$ by one-way ANOVA Tukey's multiple comparisons test *vs*. Scr group. **D-E** and **I-J,** Transwell migration assay and quantitative analysis in HepG2 and Huh7 cells. Data are presented as means ± SD. *

$p < 0.05$ and ** $p < 0.01$ by one-way ANOVA Tukey's multiple comparisons test *vs*. Scr group. All experiments were repeated at least two times.

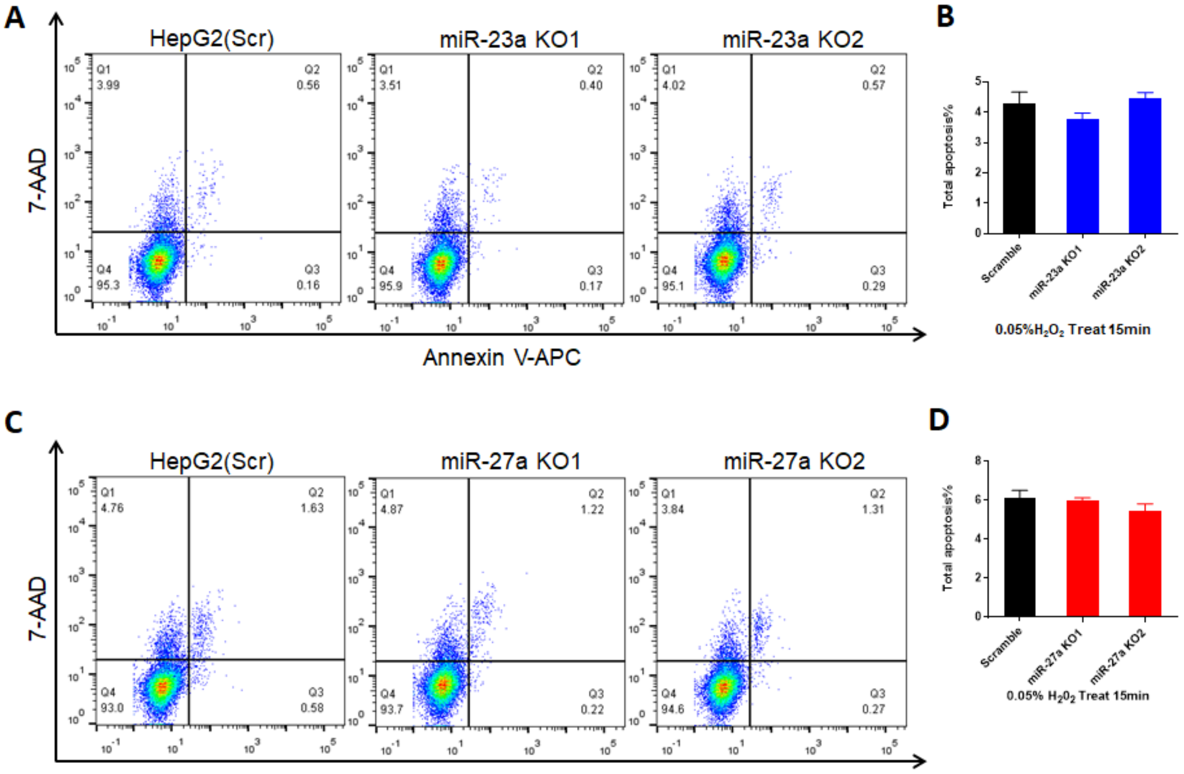


**Figure S4. Effect of the miR-23a/miR-27a KO on apoptosis in HcpG2 cells. A-B,** Cell apoptosis and quantitative analysis of Scr, miR-23a KO1, and miR-23a KO2 cells by flow cytometry with 7-AAD and Annexin V. **C-D,** Cell apoptosis and quantitative analysis of Scr, miR-27a KO1, and miR-27a KO2 cells by flow cytometry with 7-AAD and Annexin V. Scr, scramble; KO, knockout. All experiments were repeated three times.

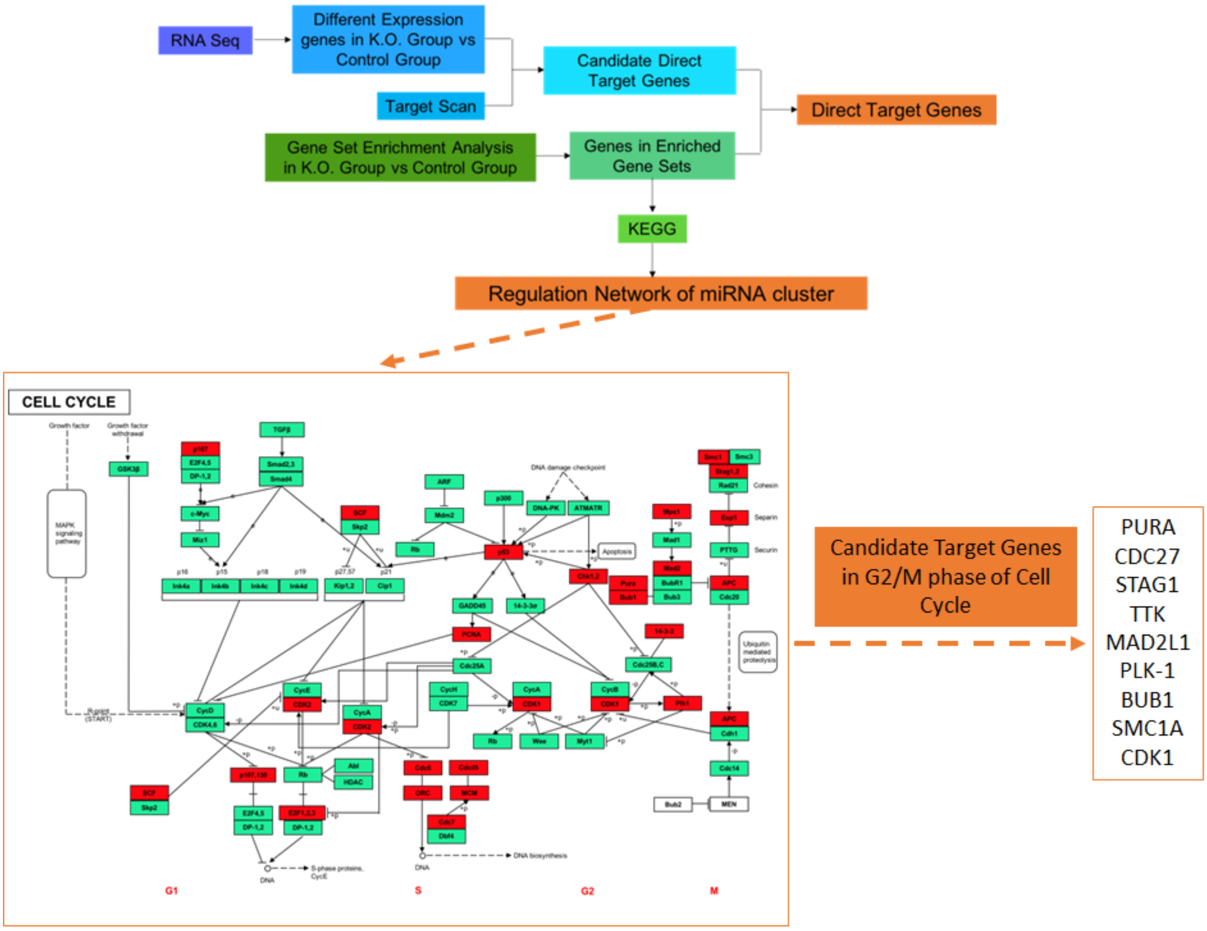


**Figure S5. Identification of miR-23a/miR-27a target genes and significantly enriched pathways by bioinformatics analysis using RNA-seq data.**

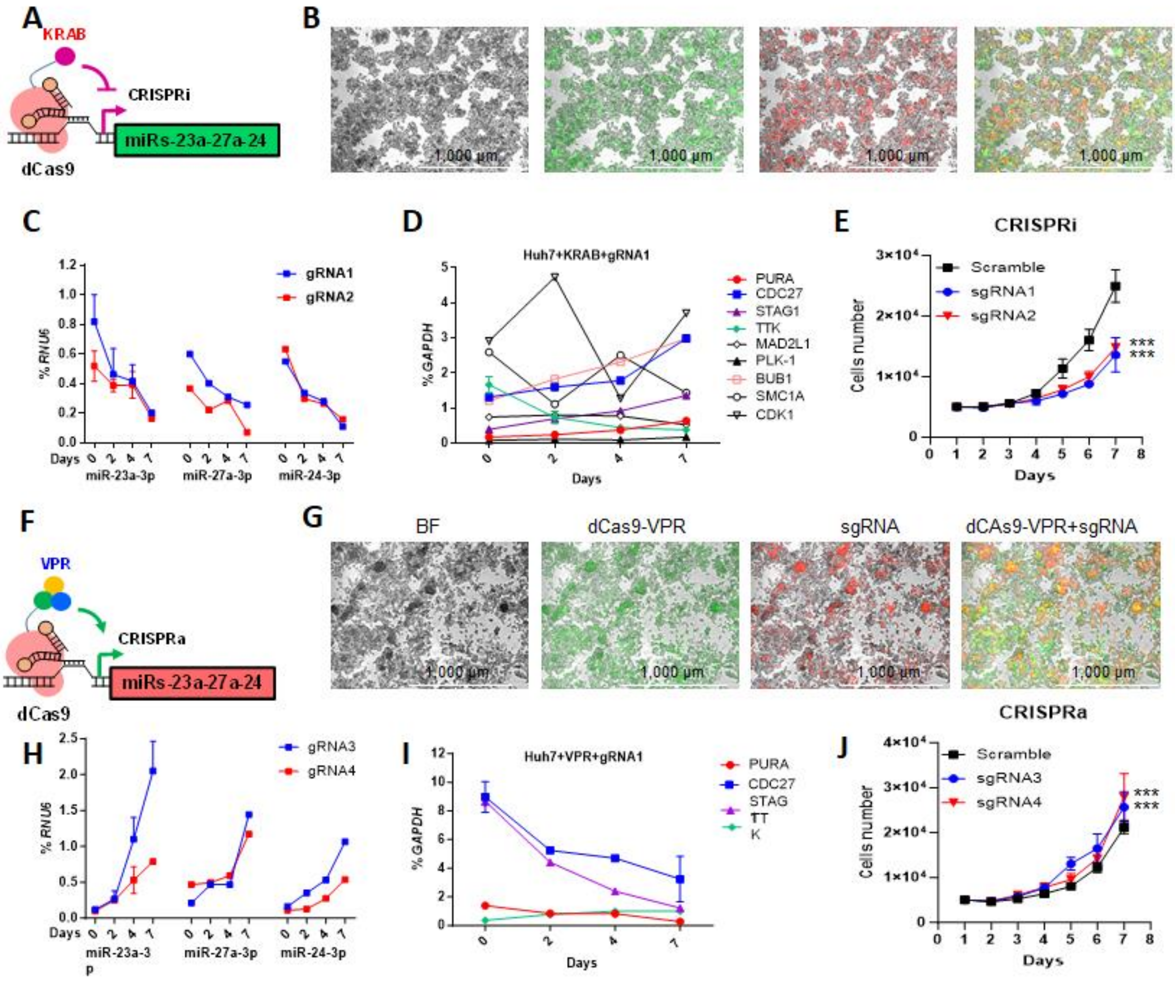


**Figure S6. Establishment of CRISPRi/a cell models and validation of miR-23a~27a~24-2 cluster target genes in Huh7 cells. A** and **F,** Diagram of CRISPR interference (CRISPRi) and activation (CRISPRa) system. **B** and **G,** Efficacy of transfection of dCas9 (GFP) and transduction of sgRNA (mCherry) into CRISPRi/a Huh7 cells at day 4 as determined by fluorescence microscopy. Scale bar =1,000 µm. **C** and **H,** Dynamic changes of miR-23a-3p, miR-27a-3p, and miR-24-3p expression in CRISPRi/a Huh7 cells detected by qPCR. **D** and **I,** Dynamic changes of candidate target gene expression in CRISPRi/a Huh7 cells detected by qPCR. **E** and **J,** Cell growth curve in CRISPRi/a Huh7 cells for 96 hours. Data are presented as means ± SD. *** $p < 0.001$ by two-way ANOVA test *vs*. Scr group. All experiments were repeated three times.

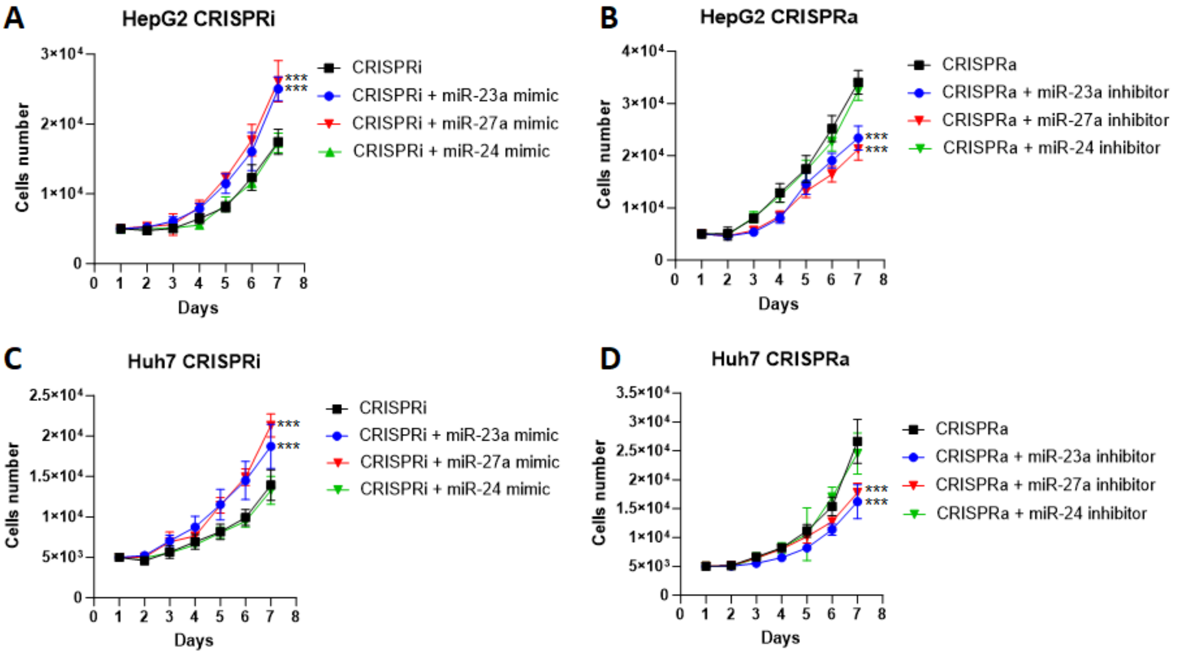


**Figure S7. Effect of miR-23a/miR-27a/miR-24 mimic and inhibitor on cell proliferation in CRISPRi/a HCC cells. A,** Cell growth curve in CRISPRi HepG2 cells treated with miR-23a-3p, miR-27a-3p, and miR-24-3p mimic, respectively. **B,** Cell growth curve in CRISPRa HepG2 cells treated with miR-23a-3p, miR-27a-3p, and miR-24-3p inhibitor, respectively. **C,** Cell growth curve in CRISPRi Huh7 cells treated with miR-23a-3p, miR-27a-3p, and miR-24-3p mimic, respectively. **D,** Cell growth curve in CRISPRa Huh7 cells treated with miR-23a-3p, miR-27a-3p, and miR-24-3p inhibitor, respectively. Data are presented as means ± SD. * $p < 0.05$ and ** $p < 0.01$ by two-way ANOVA test *vs*. Scr group.. All experiments were repeated three times.

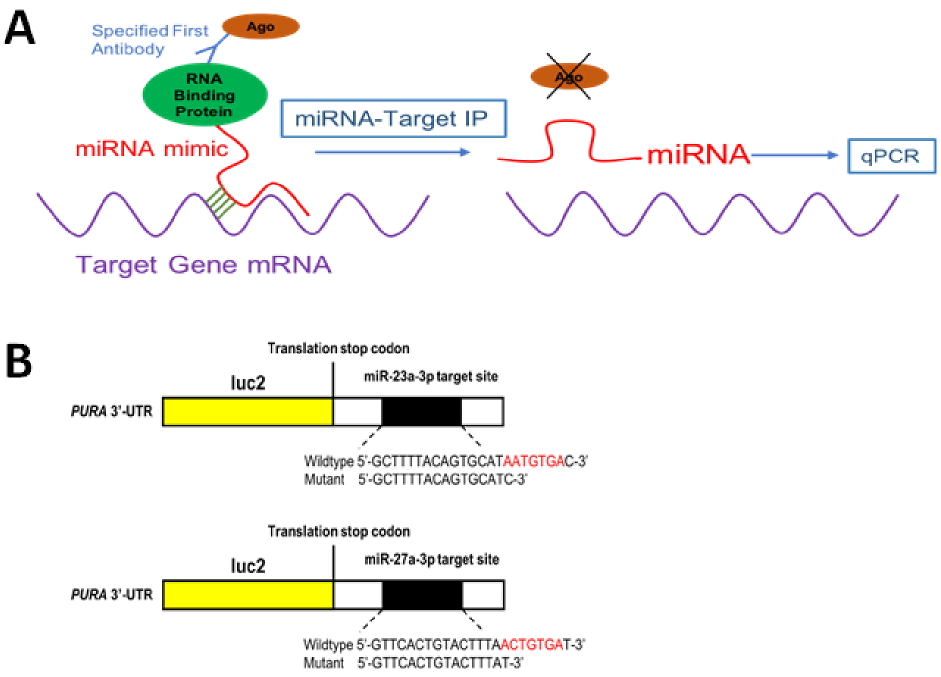

**Figure S8. A.** Schematic representation of miRNA interaction with the 3′-UTR of its corresponding targets, miRNA target immunoprecipitation (IP) with Ago proteins, miRNA-induced mRNA silencing complex, and qPCR analysis for mRNA expression from IP. **B,** Schematic of luciferase reporter constructs with Scr or mutant (deletion of residues in red) *PURA* 3'-UTR downstream of the Firefly luciferase reporter gene.

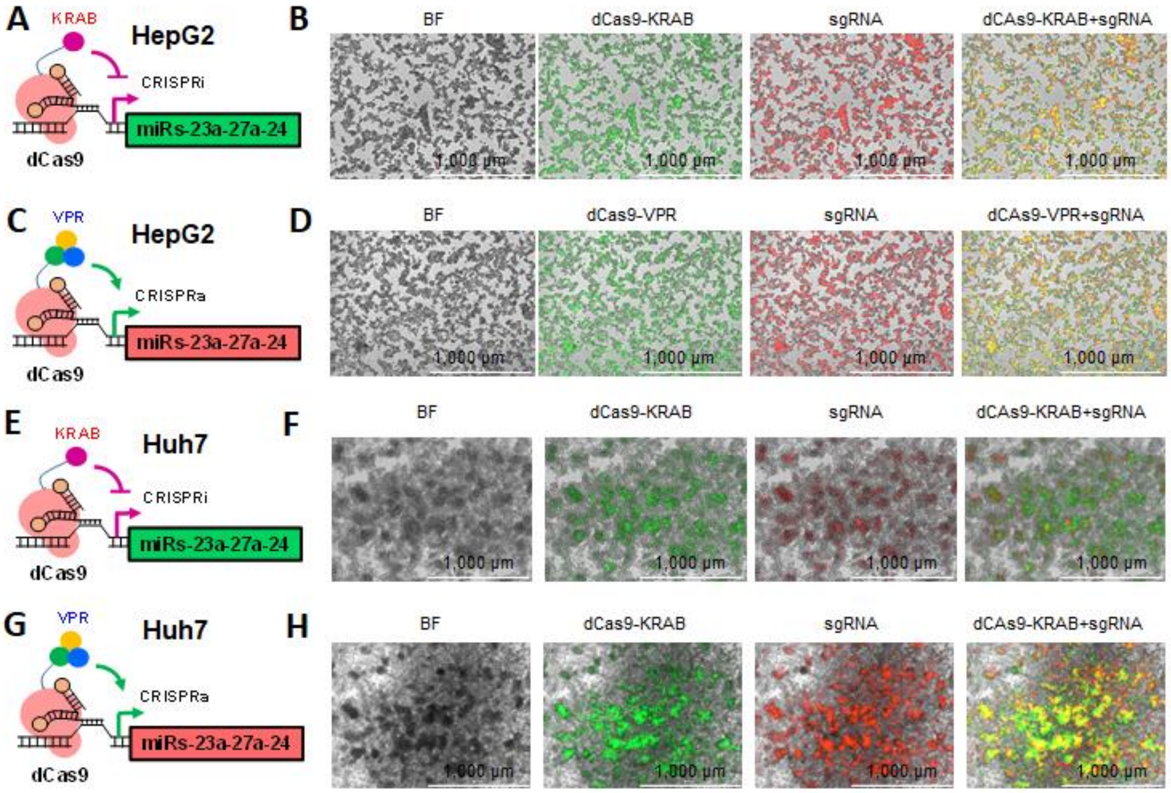


**Figure S9. A-D,** Efficacy of transfection of dCas9 (GFP) and transduction of sgRNA (mCherry) into CRISPRi/a HepG2 cells at day 4 as determined by fluorescence microscopy. Scale bar =1,000 μm. **E-H,** Efficacy of transfection of dCas9 (GFP) and transduction of sgRNA (mCherry) into CRISPRi/a Huh7 cells at day 4 as determined by fluorescence microscopy. Scale bar =1,000 μm.

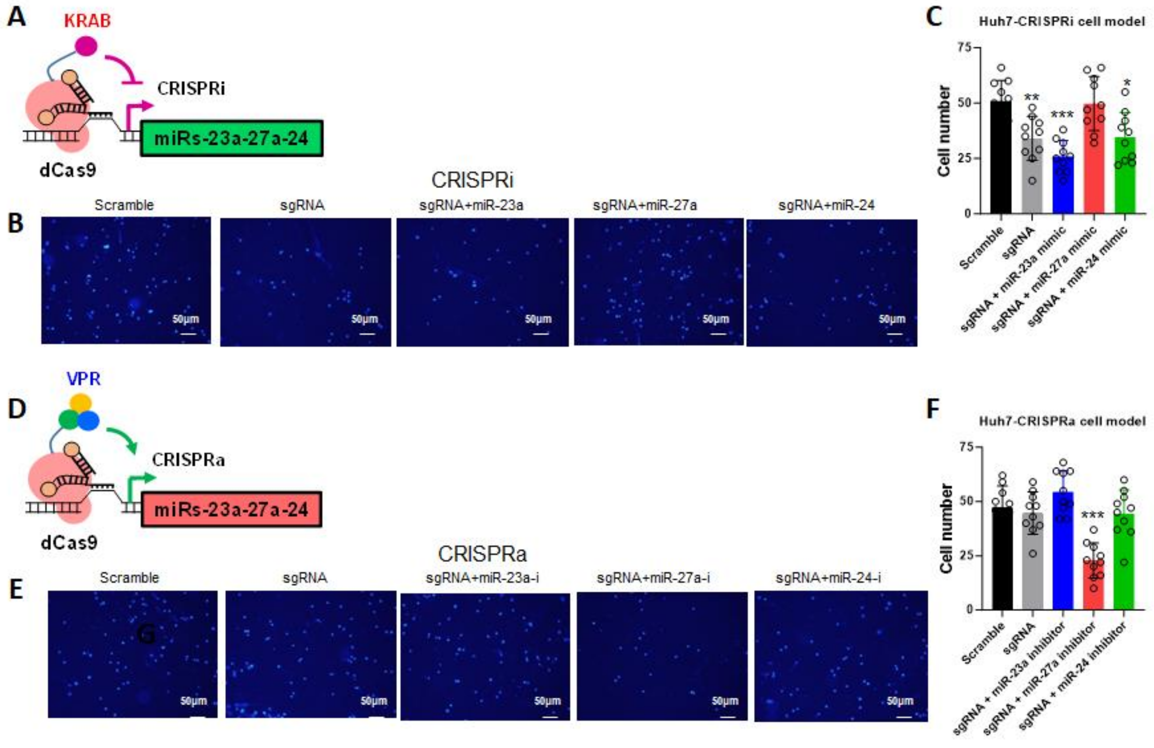


**Figure S10. Effect of miR-23a/miR-27a on cell migration and EMT in Huh7 cells.** Transwell migration assay and quantitative analysis in CRISPRi/a Huh7 cells with miRNA mimic (**A-C**) or inhibitor (**D-E**). Data are presented as means ± SD. ** $p < 0.01$ and *** $p < 0.001$ by one-way ANOVA followed by protected least-significant difference test *vs*. Scr group. All experiments were repeated three times.

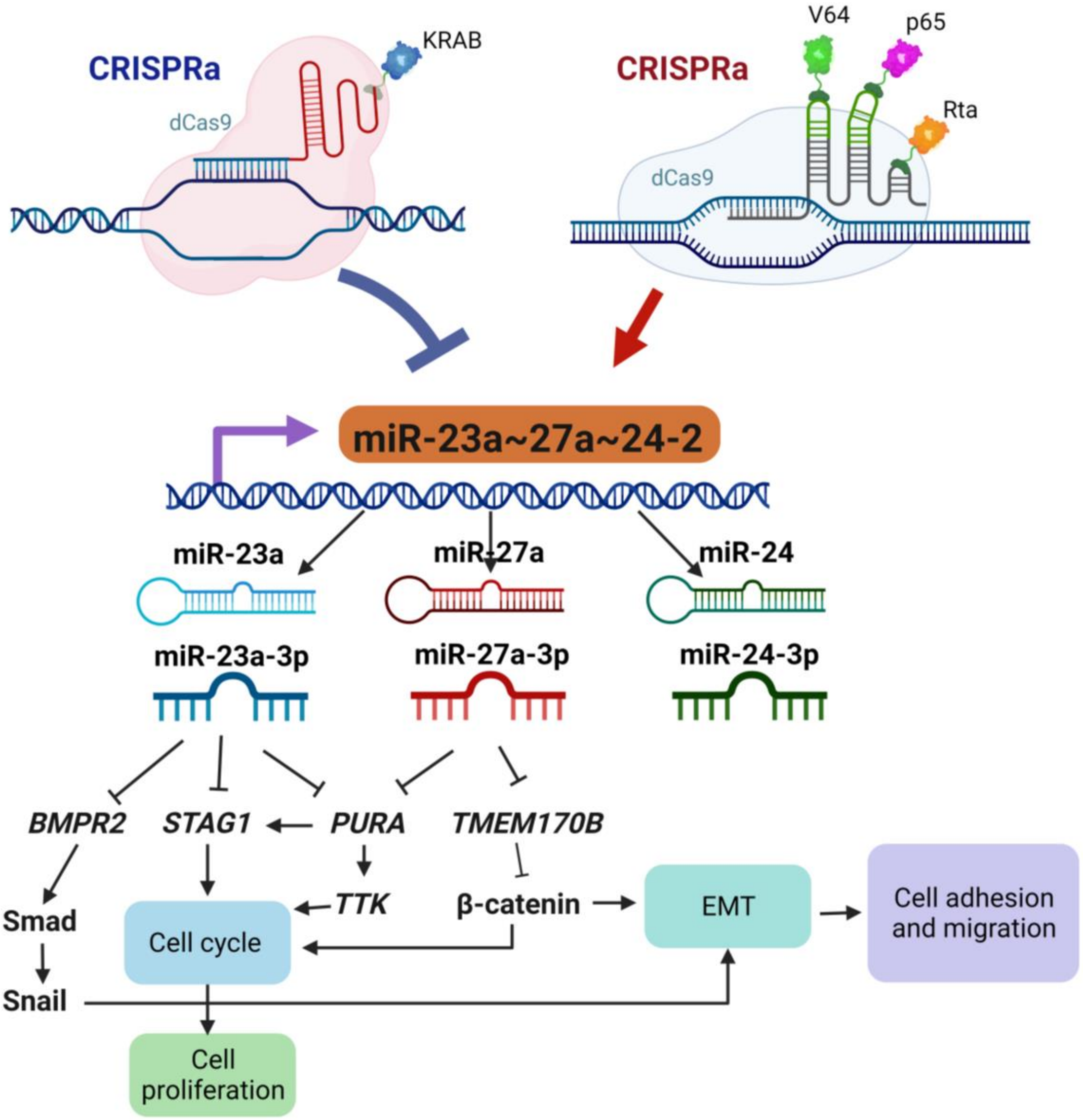


**Figure S11. Schematic representation of CRISPRi/a-mediated regulation of the miR-23a-27a-24-2 cluster and its regulated downstream gene network and signaling pathways in human HCC cells.**

Figure 3E

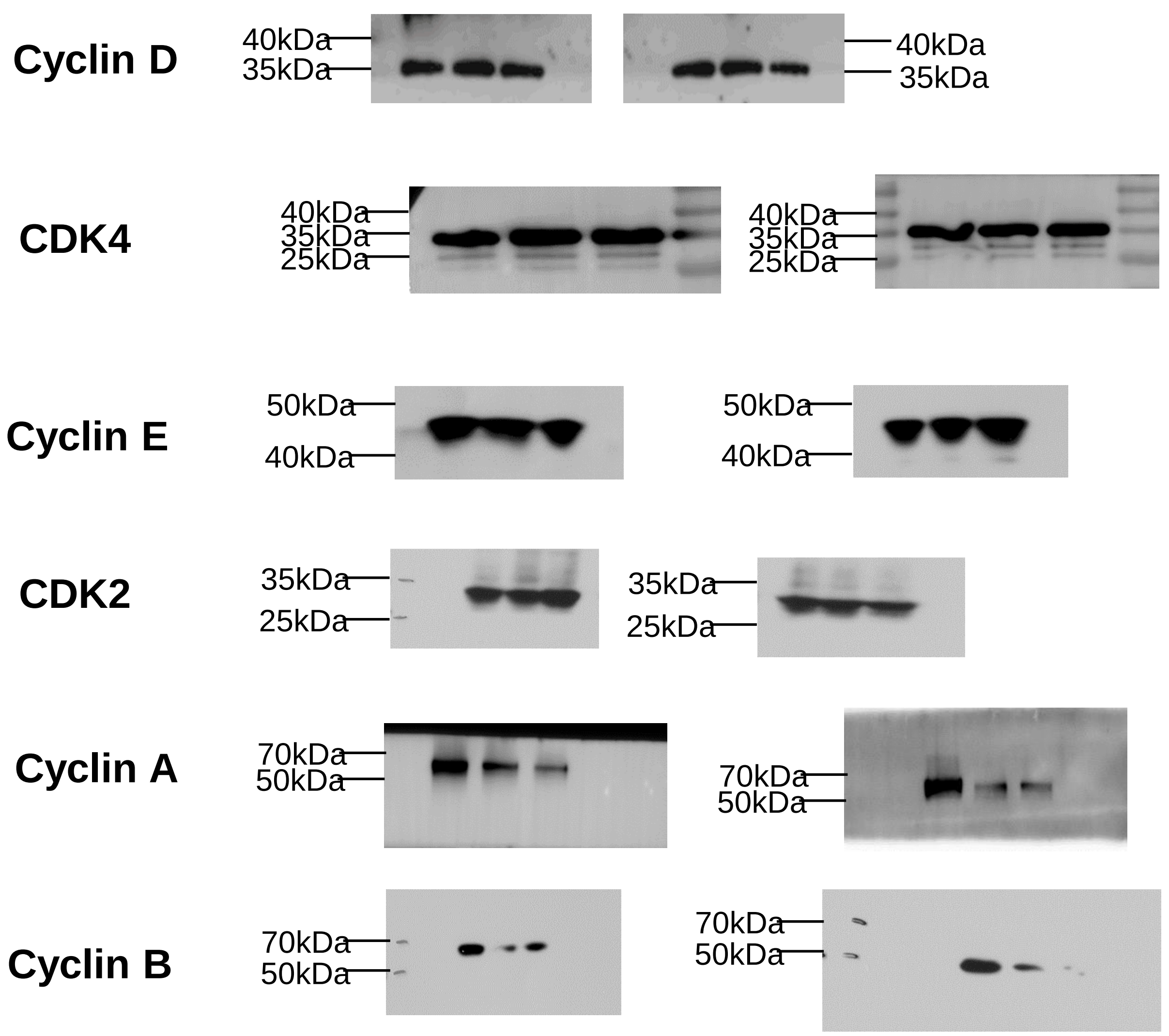

Cyclin D
40kDa
35kDa
40kDa
35kDa
CDK4
40kDa
35kDa
25kDa
40kDa
35kDa
25kDa
Cyclin E
50kDa
40kDa
50kDa
40kDa
CDK2
35kDa
25kDa
35kDa
25kDa
Cyclin A
70kDa
50kDa
70kDa
50kDa
Cyclin B
70kDa
50kDa
70kDa
50kDa

# Figure 3E

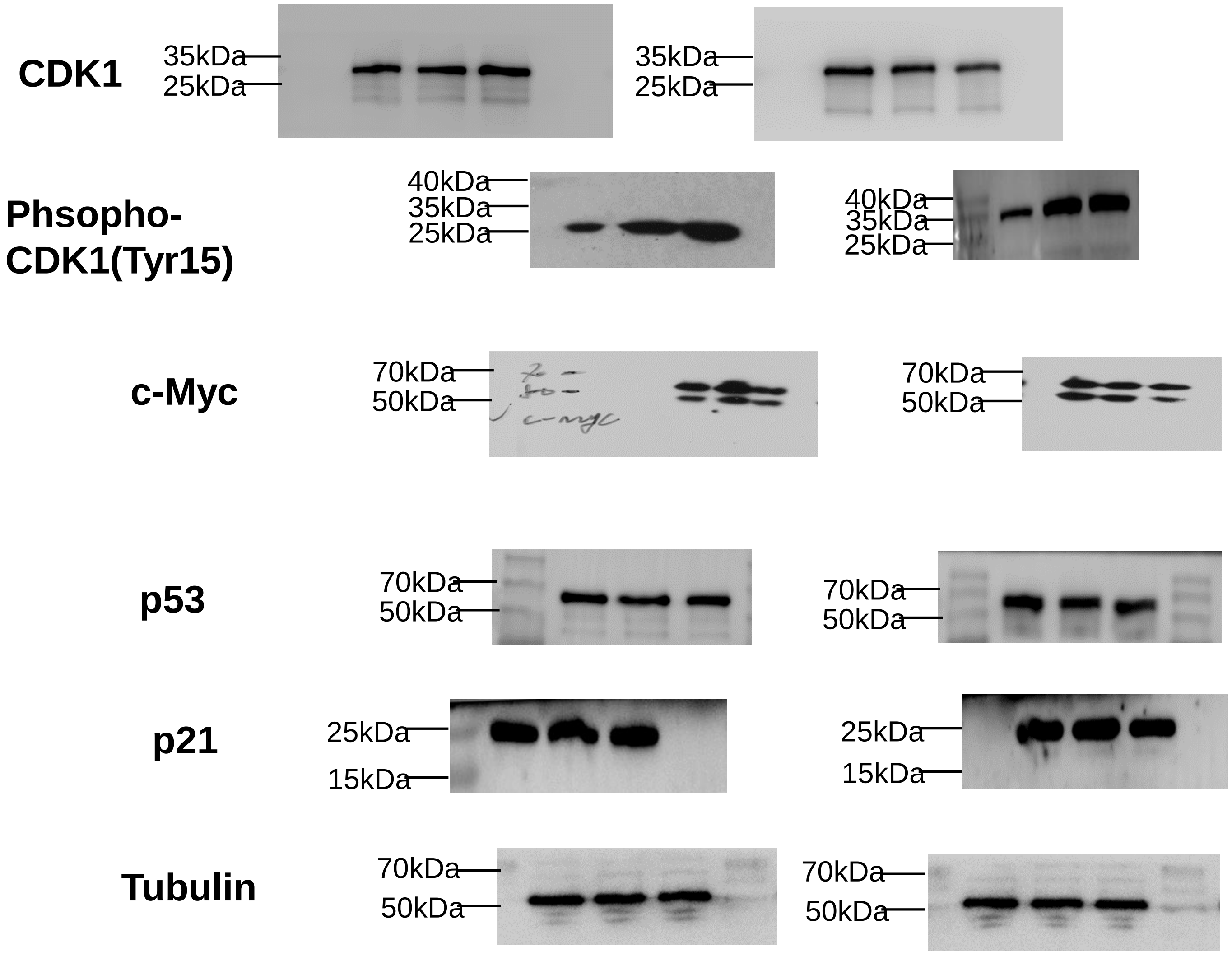
CDK1
35kDa
25kDa
35kDa
25kDa
Phsopho-CDK1(Tyr15)
40kDa
35kDa
25kDa
40kDa
35kDa
25kDa
c-Myc
70kDa
50kDa
70kDa
50kDa
p53
70kDa
50kDa
70kDa
50kDa
p21
25kDa
15kDa
25kDa
15kDa
Tubulin
70kDa
50kDa
70kDa
50kDa

# Figure 6F

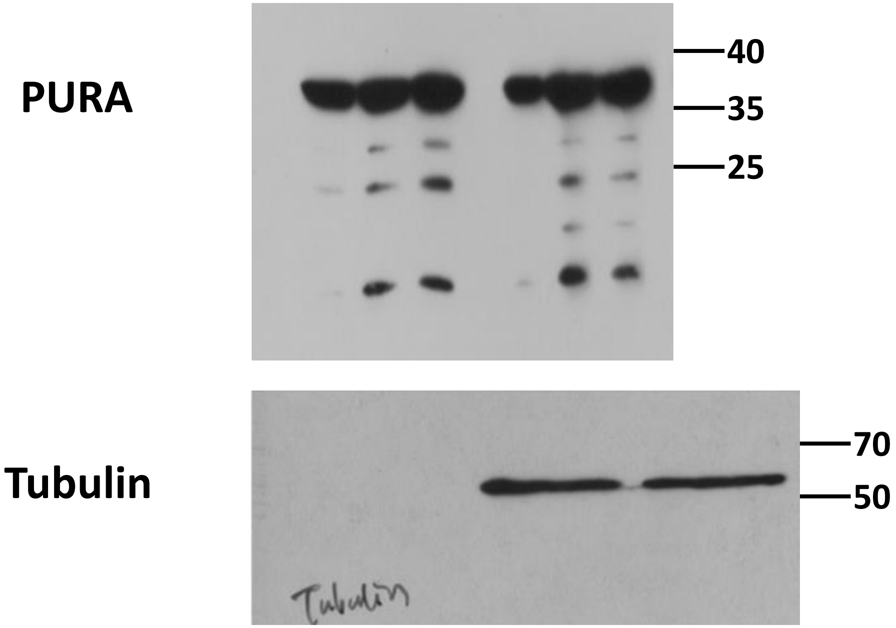


# Figure 6K

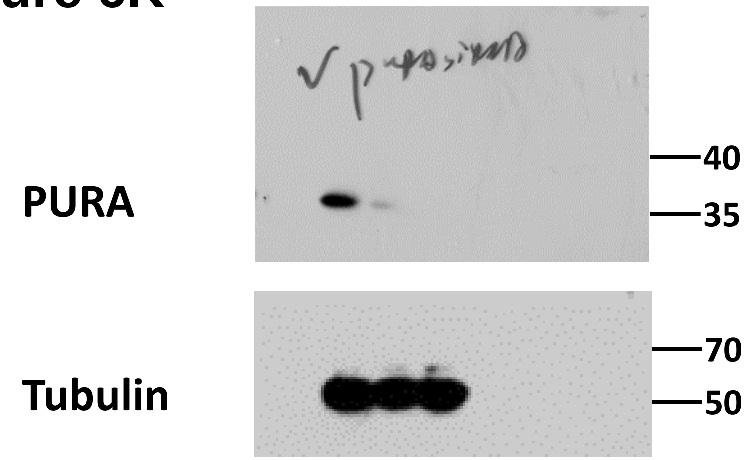

# Figure 6N

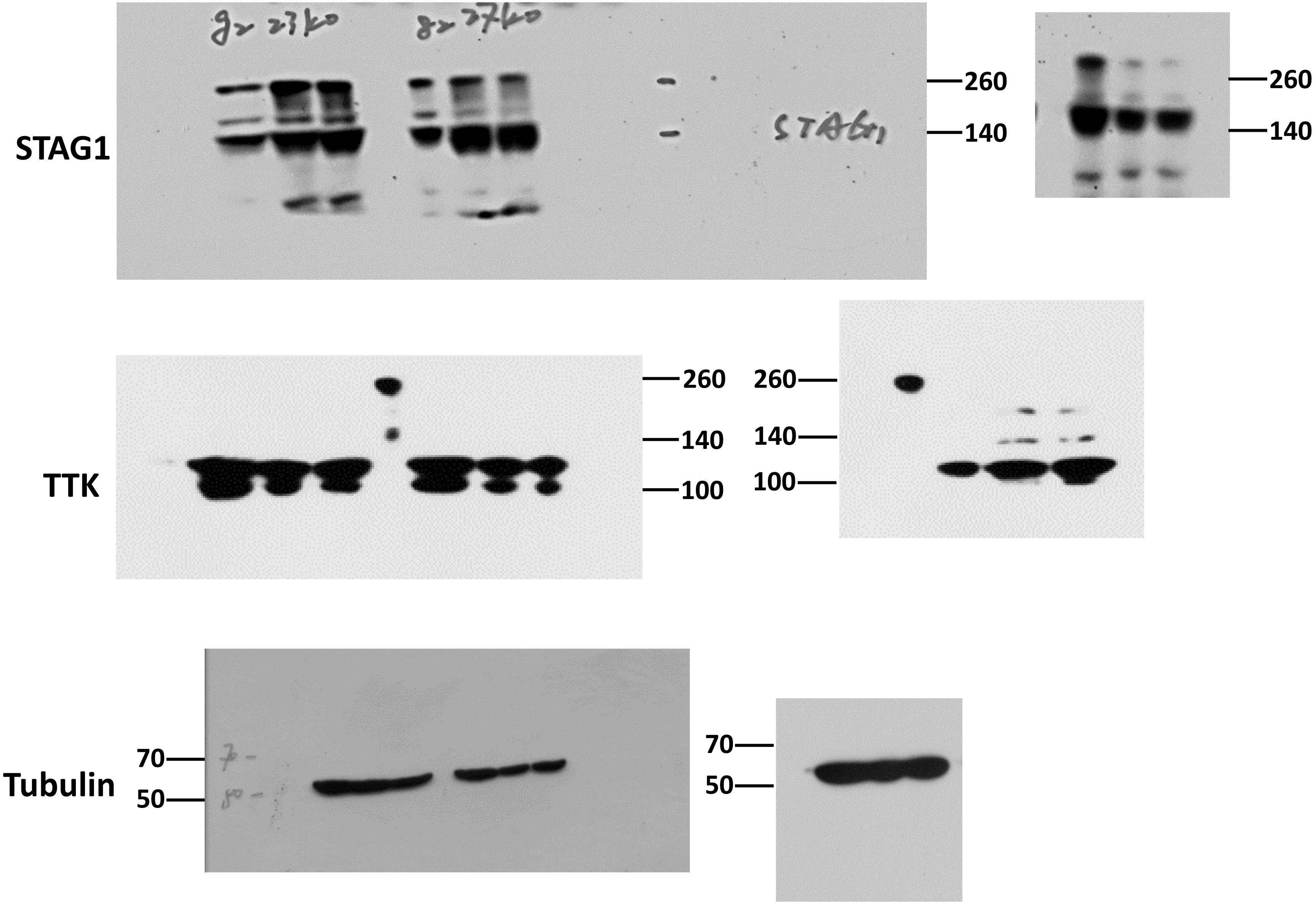
STAG1
260
140
260
140
TTK
260
140
100
260
140
100
Tubulin
70
50
70
50

# Figure 6O

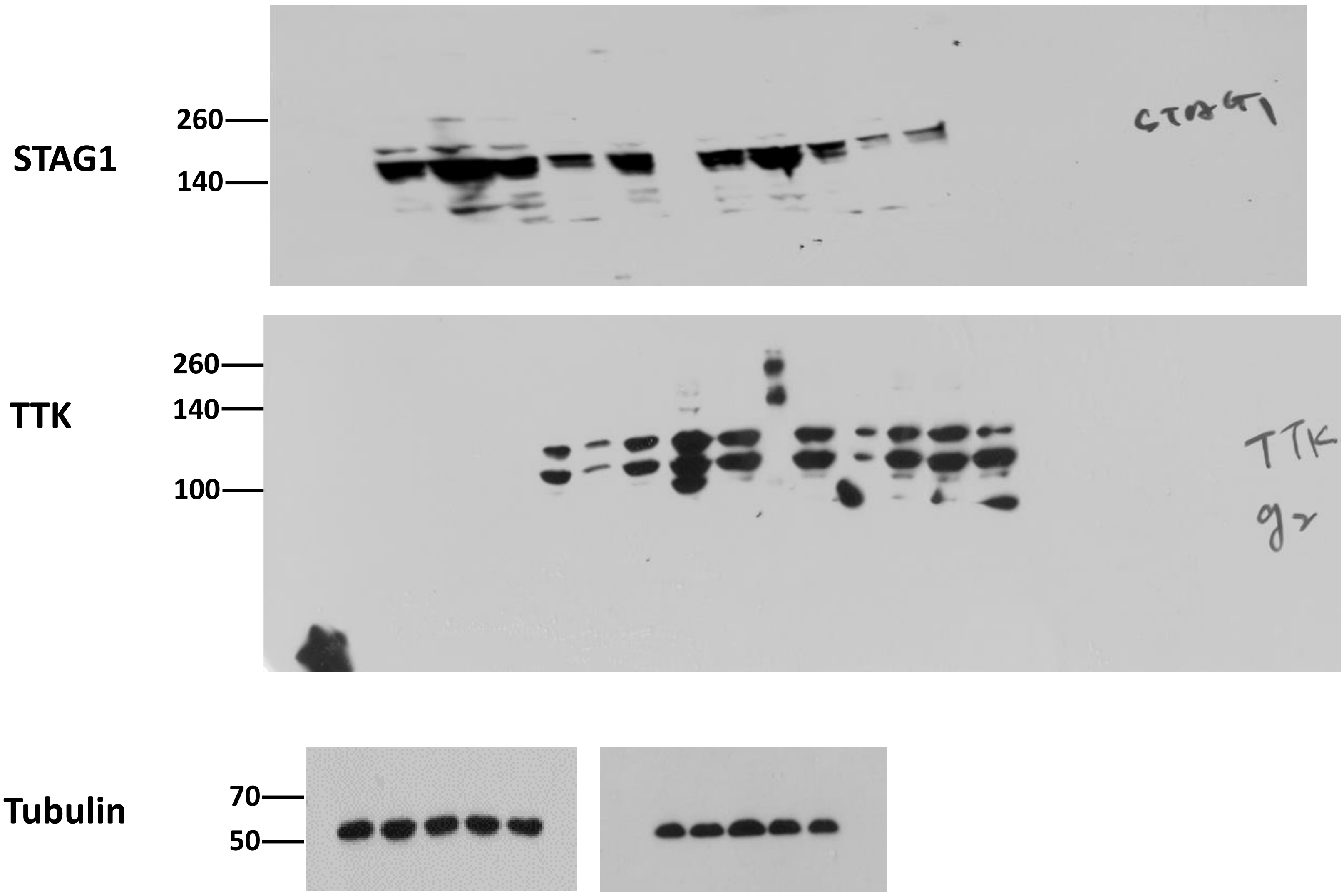

# Figure 7F

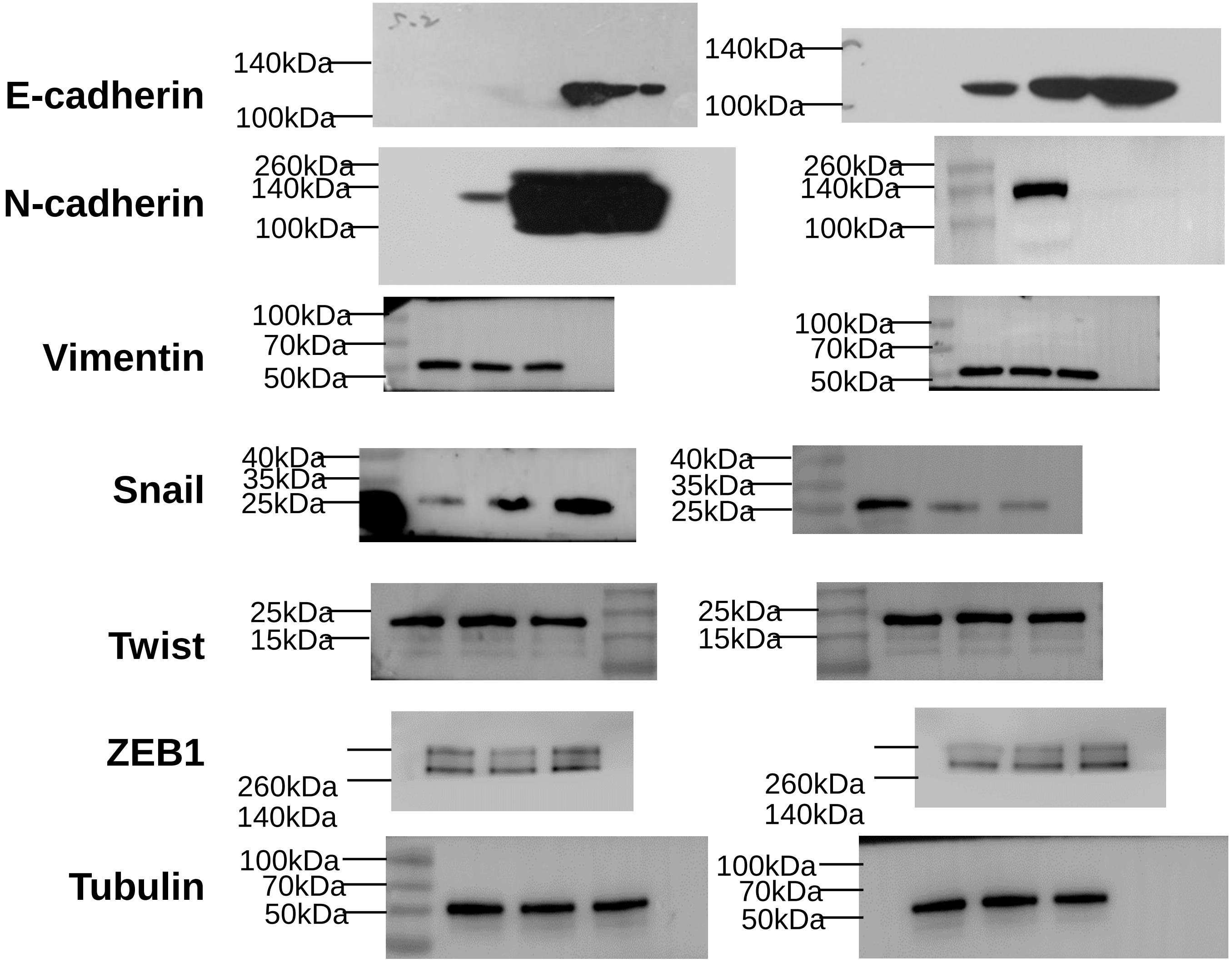
E-cadherin
140kDa
100kDa
140kDa
100kDa
N-cadherin
260kDa
140kDa
100kDa
260kDa
140kDa
100kDa
Vimentin
100kDa
70kDa
50kDa
100kDa
70kDa
50kDa
Snail
40kDa
35kDa
25kDa
40kDa
35kDa
25kDa
Twist
25kDa
15kDa
25kDa
15kDa
ZEB1
260kDa
140kDa
260kDa
140kDa
Tubulin
100kDa
70kDa
50kDa
100kDa
70kDa
50kDa

Figure 8A

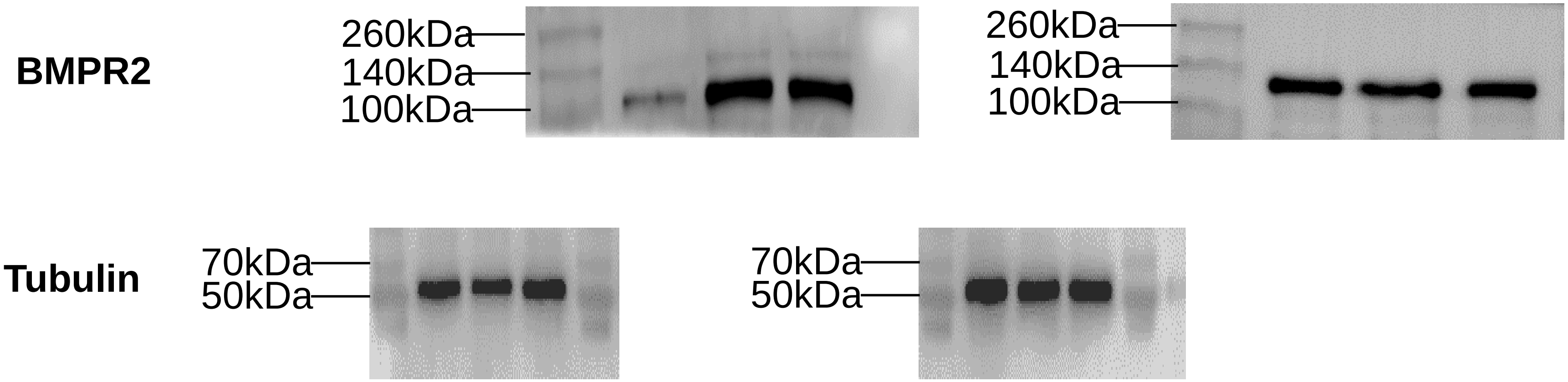


Figure 8C

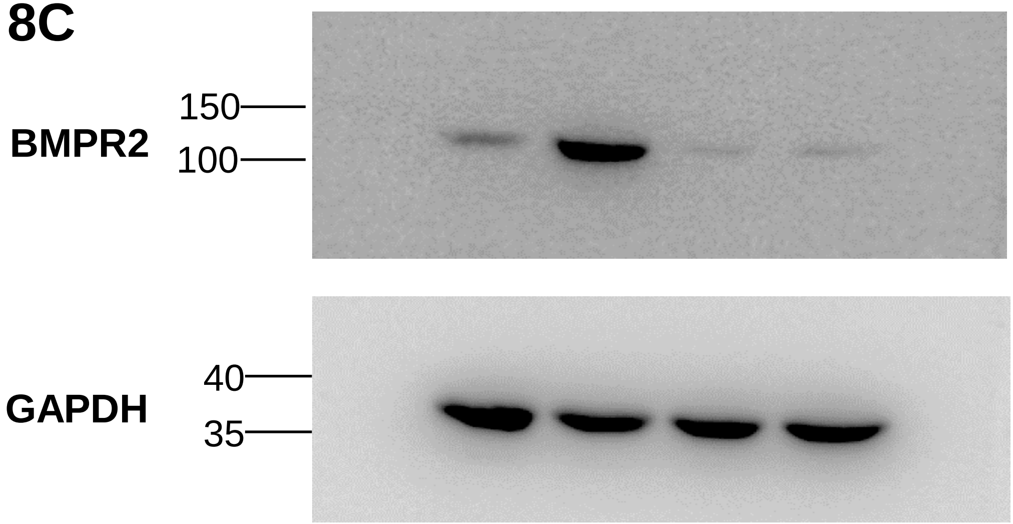

# Figure 8F

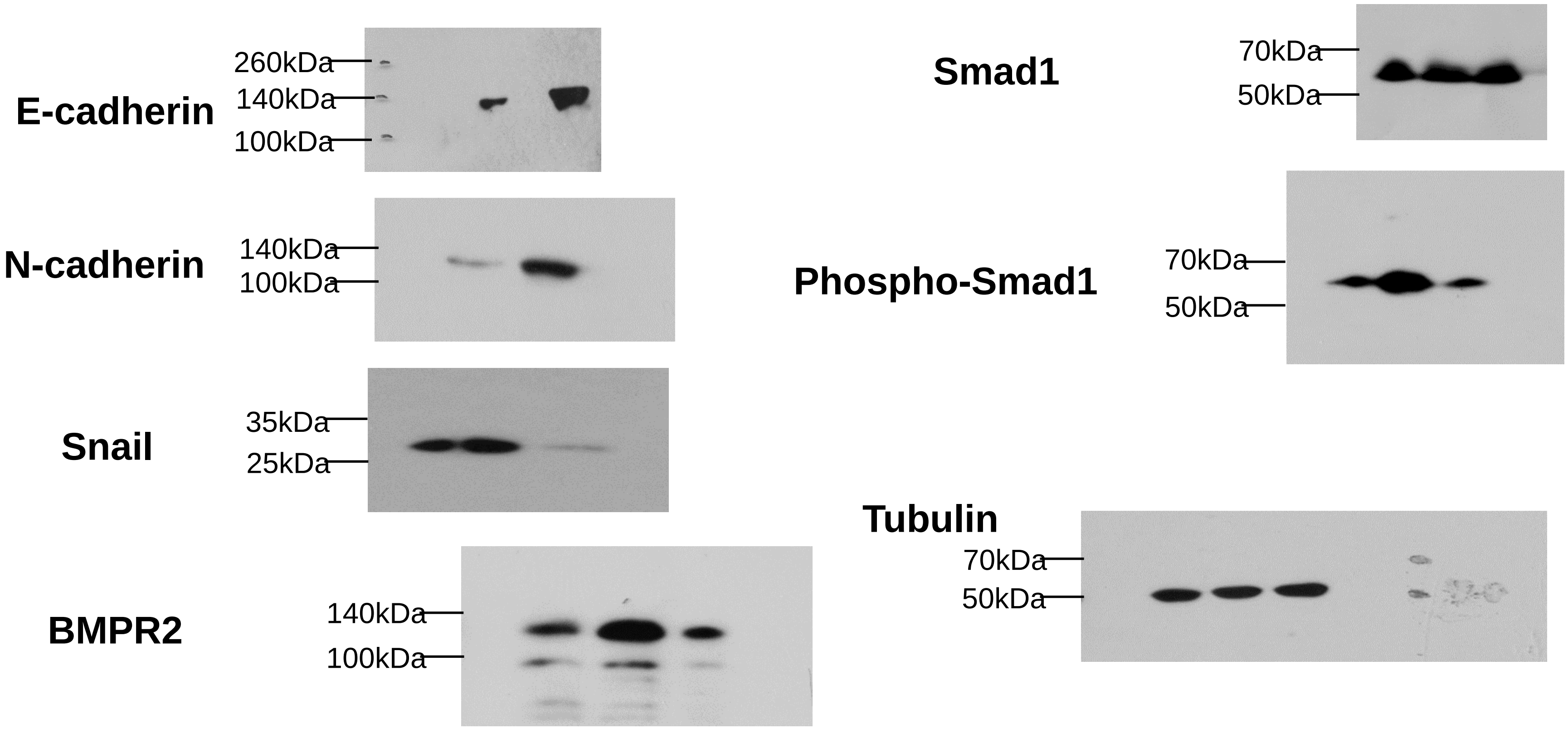
E-cadherin
260kDa
140kDa
100kDa
N-cadherin
140kDa
100kDa
Snail
35kDa
25kDa
BMPR2
140kDa
100kDa
Smad1
70kDa
50kDa
Phospho-Smad1
70kDa
50kDa
Tubulin
70kDa
50kDa

# Figure 8G

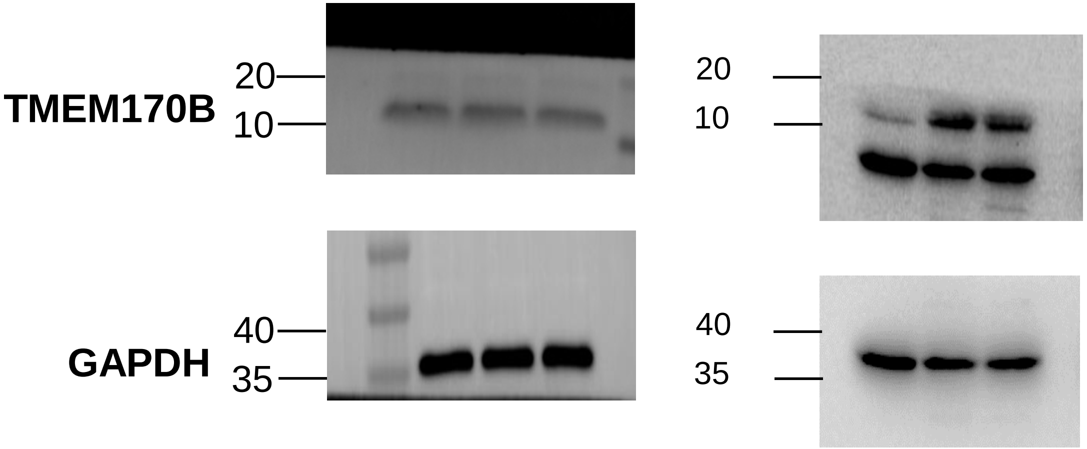


# Figure 8I

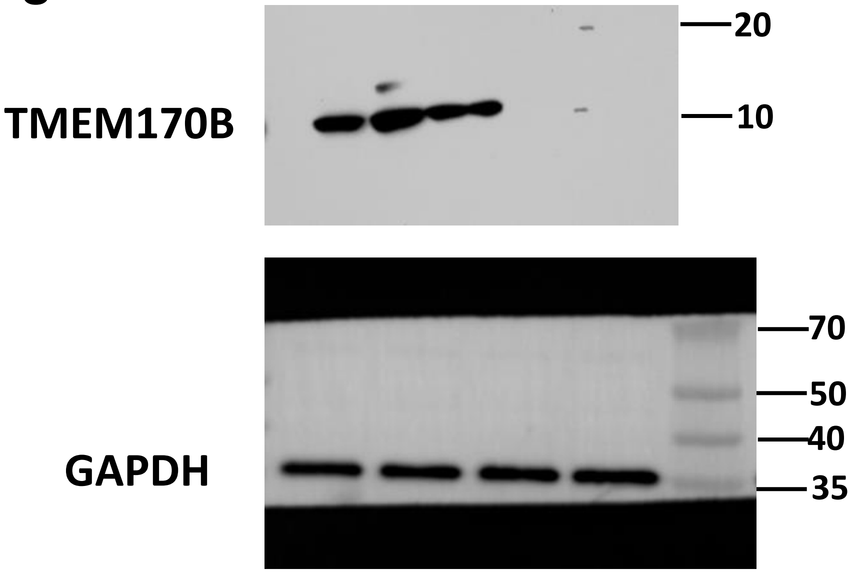

# Figure 8L

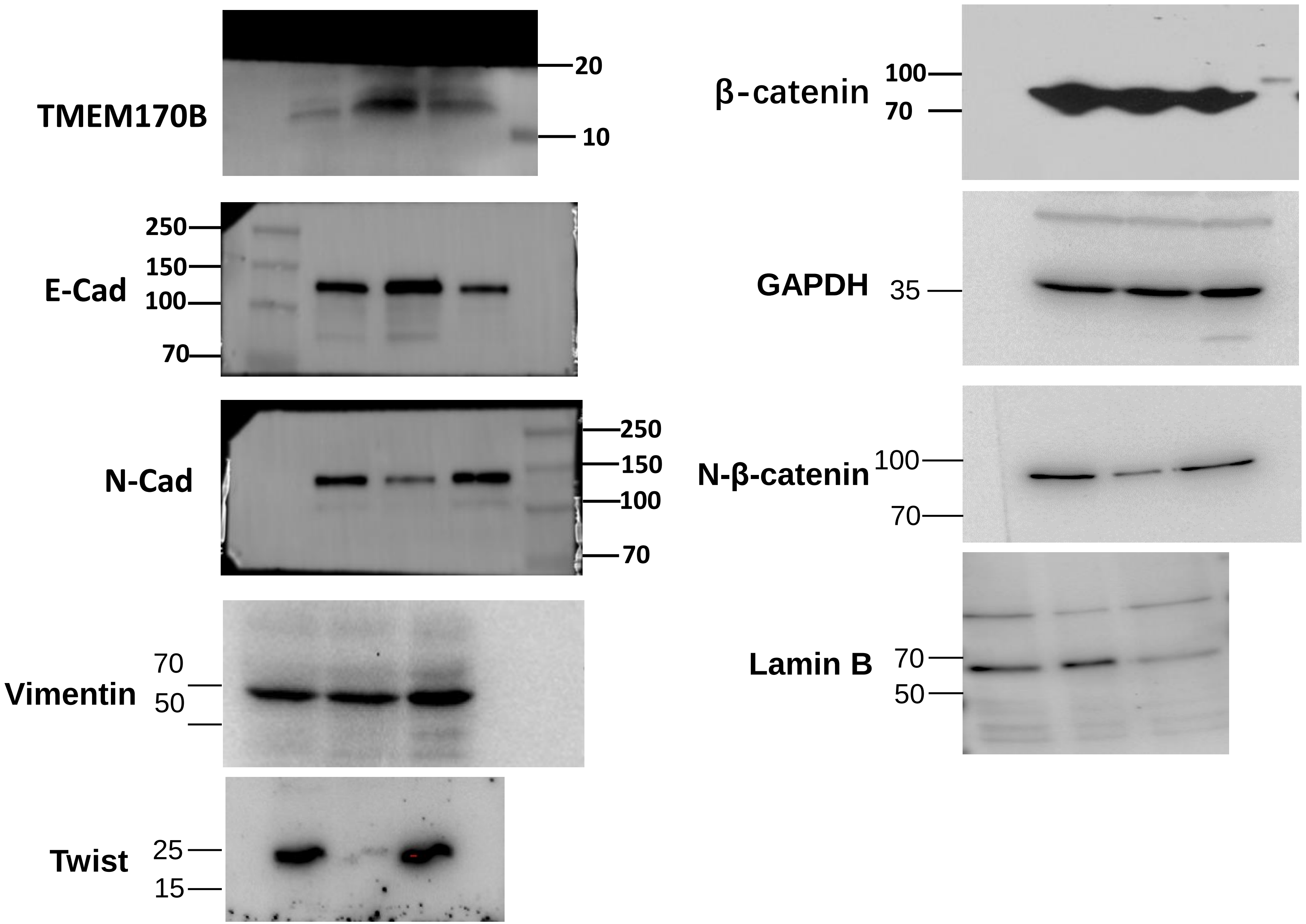
TMEM170B
20
10
E-Cad
250
150
100
70
N-Cad
250
150
100
70
Vimentin
70
50
Twist
25
15
β-catenin
100
70
GAPDH
35
N-β-catenin
100
70
Lamin B
70
50
