# Supplementary material for: CRISPR-based dissection of microRNA-23a ~ 27a ~ 24-2 cluster functionality in hepatocellular carcinoma
Source: Oncogene. 2024 Aug 7;43(36):2708–21. doi: 10.1038/s41388-024-03115-z (PMC11364504; doi:10.1038/s41388-024-03115-z)
Supplement: Supplementary file 2 — Supplementary Tables [file 41388_2024_3115_MOESM2_ESM.pdf]

Table S1a. Genes up-regulated (>1.5-fold change, q-value < 0.001) after miR-23a knock out

Symobol	GeneID	23aKO1 FPKM	23aKO2 FPKM	Scr1 FPKM	Scr2 FPKM	log2Ratio 23aKO/Scr	q-value	p-value
AASDH	132949	0.8	2.12	1.23	1.25	0.70	0.00	0.00
AASDHPPT	60496	5.41	12.22	4.6	6.63	0.65	0.00	0.00
AASS	10157	0.34	1.54	0.48	0.54	0.88	0.00	0.00
ABCA10	10349	0.02	0.04	0	0.02	1.99	0.00	0.00
ABCB10	23456	1.87	5.21	1.5	2.89	0.65	0.00	0.00
ABCD1	215	38.78	16.66	20.07	26.52	0.76	0.00	0.00
ABCD3	5825	18.09	36.11	10.91	16.21	1.21	0.00	0.00
ABCE1	6059	6.08	25.86	6.6	11.27	0.82	0.00	0.00
ABHD10	55347	7.7	12.64	5.79	6.13	0.73	0.00	0.00
ABHD13	84945	0.96	2.48	1.07	1.02	0.71	0.00	0.00
ABHD14A-ACY1	100526760	2.1	0.72	0.32	0.78	1.37	0.00	0.00
ABHD17A	81926	143.3	54.8	67.32	54.57	0.71	0.00	0.00
ABHD18	80167	0.77	2.56	0.9	1.47	0.67	0.00	0.00
ABHD8	79575	39.61	18.03	17.53	15.55	0.81	0.00	0.00
ABRAXAS2	23172	1.16	3.3	0.87	1.53	0.88	0.00	0.00
ACADM	34	5.8	17.98	3.31	6.68	1.22	0.00	0.00
ACADSB	36	3.53	7.26	5.94	4.4	0.76	0.00	0.00
ACAP2	23527	0.31	1.98	0.4	0.94	0.89	0.00	0.00
ACBD3	64746	2.56	8.72	2.08	2.57	1.25	0.00	0.00
ACBD5	91452	0	0	0	0	0.77	0.00	0.00
ACE2	59272	0.62	1.79	0.5	0.99	0.77	0.00	0.00
ACSL1	2180	0.16	0.57	0.22	0.35	0.85	0.00	0.00
ACSM1	116285	0.67	0.27	0.05	0.29	2.10	0.00	0.00
ACSM2A	123876	1.1	1.07	0.8	0.56	0.66	0.00	0.00
ACSM5	54988	3.8	3.58	2.29	2.15	0.74	0.00	0.00
ACTL8	81569	2.3	0.98	0.88	0.7	1.05	0.00	0.00
ACTR2	10097	13.96	52.69	17.68	25.31	0.63	0.00	0.00
ACTR6	64431	1.68	2.6	0.66	0.9	1.46	0.00	0.00
ADAL	161823	0.81	2.99	1.1	1.11	0.59	0.00	0.00

ADAM10	102	2.43	14.46	4.26	6.52	0.65	0.00	0.00
ADAM11	4185	14.19	2.46	2.85	4.2	1.24	0.00	0.00
ADAM19	8728	0.69	0.85	0.47	0.33	0.96	0.00	0.00
ADAM22	53616	0	0	0	0	0.64	0.00	0.00
ADAM9	8754	5.23	26.97	7.53	13.87	0.69	0.00	0.00
ADAMTS6	11174	0.03	0.74	0.14	0.06	1.48	0.00	0.00
ADAT3	113179	19.51	7.43	6.61	7.62	0.84	0.00	0.00
ADCY10P1	221442	0.11	0.33	0.15	0.09	0.96	0.00	0.00
ADCY3	109	2.15	2.35	1.3	1.34	0.75	0.00	0.00
ADD3	120	2.58	10.36	2.31	4.11	0.95	0.00	0.00
ADGRF3	165082	1.39	0.42	0.27	0.56	1.27	0.00	0.00
ADGRG6	57211	3.24	26.44	7.61	9.53	0.78	0.00	0.00
ADGRL2	23266	0.64	4.3	0.82	1.68	1.00	0.00	0.00
ADORA1	134	0	0.02	0	0	2.62	0.00	0.00
ADORA2A	135	15.82	6.9	6.31	5.11	0.99	0.00	0.00
ADORA2A-AS1	646023	1.14	0.3	0	0.23	2.62	0.00	0.00
ADSS	159	3.46	12.54	4.45	4.55	0.82	0.00	0.00
AFAP1	60312	0.72	1.09	0.48	0.6	0.72	0.00	0.00
AFF4	27125	0.6	7.78	2.09	3.32	0.68	0.00	0.00
AFP	174	1319.2	3289.64	1030.99	1594.7	0.80	0.00	0.00
AFTPH	54812	1.28	6.71	2.14	2.82	0.62	0.00	0.00
AGGF1	55109	0.69	2.84	0.55	1.19	1.01	0.00	0.00
AGL	178	0	0	0	0	0.85	0.00	0.00
AGPS	8540	2.64	14.08	3.34	5.17	0.97	0.00	0.00
AGTPBP1	23287	1.94	7.72	1.61	1.92	1.10	0.00	0.00
AHCTF1	25909	0.84	5.11	0.81	1.61	1.27	0.00	0.00
AHI1	54806	0.61	2.27	1.31	0.92	1.15	0.00	0.00
AIMP2	7965	63.29	20.04	26.13	22.65	0.70	0.00	0.00
AIRN	100271873	16.85	20.07	16.76	26.22	1.89	0.00	0.00
AJM1	389813	4.17	1.01	0.33	2.11	1.10	0.00	0.00
AK6	102157402	14.2	13.87	4.88	4.95	1.43	0.00	0.00
AK9	221264	0.29	1.13	0.31	0.4	1.03	0.00	0.00
AKAP11	11215	0.21	1.58	0.38	0.58	0.86	0.00	0.00

AKAP6	9472	0.03	0.73	0.17	0.33	1.00	0.00	0.00
AKAP7	9465	1.38	4.56	1.28	2.36	0.66	0.00	0.00
AKAP9	10142	0.26	0.63	0.2	0.42	0.63	0.00	0.00
AKR1D1	6718	3.2	4.89	1.17	2.03	1.33	0.00	0.00
AKR1E2	83592	0.52	6.18	2.59	1.65	0.59	0.00	0.00
AKT1	207	105.24	45.86	49.07	47.91	0.66	0.00	0.00
AKT1S1	84335	211.72	74.55	81.69	92.43	0.71	0.00	0.00
AKT2	208	0	0	0	0	0.59	0.00	0.00
ALDOC	230	57.31	33.31	35.63	25.02	0.58	0.00	0.00
ALG1	56052	51.46	26.34	21.04	27.73	0.61	0.00	0.00
ALG10	84920	0.52	1.15	0.23	0.57	1.04	0.00	0.00
ALG11	440138	0.76	2.42	0.98	0.89	1.15	0.00	0.00
ALKBH8	91801	0.15	1.22	0.31	0.27	1.34	0.00	0.00
ALMS1	7840	0.39	2.22	0.46	0.75	1.30	0.00	0.00
ALPK2	115701	0.16	4.26	1.75	0.78	0.87	0.00	0.00
AMD1	262	11.48	30.42	9.21	14.84	0.80	0.00	0.00
AMMECR1	9949	0.63	1.75	0.65	0.65	0.79	0.00	0.00
ANAPC4	29945	0.91	6	1.66	2.72	0.65	0.00	0.00
ANGEL2	90806	1.06	2.51	1.13	1.17	0.68	0.00	0.00
ANGPT2	285	0.52	0	0	0	6.13	0.00	0.00
ANGPTL3	27329	0.79	0.28	0	0.22	2.25	0.00	0.00
ANGPTL4	51129	0.98	4.63	2.65	0.93	0.62	0.00	0.00
ANKIB1	54467	0.7	3.37	0.8	1.15	1.00	0.00	0.00
ANKMY2	57037	0	0	0	0	0.64	0.00	0.00
ANKRD13C	81573	2.3	3.74	1.64	2.39	0.60	0.00	0.00
ANKRD17	26057	3.92	19.61	6.07	8.25	0.69	0.00	0.00
ANKRD18A	253650	0.48	1.1	0.55	0.55	0.66	0.00	0.00
ANKRD23	200539	1.64	1.28	0.44	0.71	1.26	0.00	0.00
ANKRD26	22852	0.27	0.75	0.25	0.38	0.90	0.00	0.00
ANKRD28	23243	2.18	7.84	3.46	6.98	0.82	0.00	0.00
ANKRD36	375248	0.2	0.7	0.37	0.31	0.86	0.00	0.00
ANKRD36B	57730	0.01	0	0	0	1.05	0.00	0.00
ANKRD49	54851	1.65	1.85	0.57	0.76	1.16	0.00	0.00

ANKRD50	57182	0.27	1.21	0.29	0.48	0.94	0.00	0.00
ANKRD52	283373	14	15.51	9.73	9.52	0.61	0.00	0.00
ANKRD55	79722	2.2	1.88	1.31	0.67	1.40	0.00	0.00
ANLN	54443	2.43	11.62	1.51	2.65	1.73	0.00	0.00
ANP32E	81611	3.91	15.42	3.45	4.85	1.23	0.00	0.00
AP1AR	55435	4.43	9.89	3.46	5.32	0.76	0.00	0.00
AP1M2	10053	33.13	6.72	7.07	11.37	1.12	0.00	0.00
AP1S2	8905	0	0	0	0	0.65	0.00	0.00
AP3B1	8546	0	0	0	0	0.66	0.00	0.00
AP4E1	23431	0.37	2.02	0.56	0.91	0.63	0.00	0.00
APAF1	317	0.66	2.91	0.57	0.91	1.28	0.00	0.00
APBA3	9546	30.16	15.21	15.79	10.69	0.76	0.00	0.00
APC	324	0.09	1	0.18	0.42	0.79	0.00	0.00
APC2	10297	15.51	6.36	8.1	6.52	0.58	0.00	0.00
API5	8539	3.07	7.43	1.46	0.43	2.33	0.00	0.00
APOA1	335	0.07	0.18	0	0	0.77	0.00	0.00
APOA4	337	65.77	18.61	22.07	32.52	0.64	0.00	0.00
APOC3	345	880.47	212.46	314.41	341.12	0.76	0.00	0.00
APOOL	139322	0.93	3.43	0.96	1.34	0.94	0.00	0.00
APPL1	26060	1.84	6.01	1	2.02	1.14	0.00	0.00
APTR	100505854	1.96	2.55	0.58	1.08	0.90	0.00	0.00
AQP4	361	0.15	0.12	0.02	0.01	2.76	0.00	0.00
AQP6	363	0.86	0.62	0.29	0.46	1.03	0.00	0.00
ARFGEF1	10565	0.41	3.22	0.55	1.02	1.09	0.00	0.00
ARFIP1	27236	1.5	5.05	1.55	2.24	0.74	0.00	0.00
ARGLU1	55082	6.97	25.83	8.72	10.23	0.81	0.00	0.00
ARHGAP10	79658	1.12	2.13	0.6	0.27	1.95	0.00	0.00
ARHGAP11A	9824	0.76	6.51	0.92	2.06	1.17	0.00	0.00
ARHGAP12	94134	1.62	11.09	2.32	3.2	1.17	0.00	0.00
ARHGAP18	93663	5.4	31.4	6.1	10.83	1.10	0.00	0.00
ARHGAP29	9411	1.42	7.06	1.5	1.84	1.39	0.00	0.00
ARHGAP5	394	1.58	6.31	0.96	1.7	1.44	0.00	0.00
ARHGEF12	23365	1.33	12.37	3.45	5.16	0.66	0.00	0.00

ARHGEF26	26084	0.6	1.78	0.4	0.65	1.11	0.00	0.00
ARHGEF38	54848	0.02	0.07	0.02	0	4.00	0.00	0.00
ARL13B	200894	0.55	1.44	0.53	0.59	0.88	0.00	0.00
ARL14EP	120534	0.75	2.42	0.89	1.01	0.80	0.00	0.00
ARL15	54622	0.4	4.4	1.26	1.12	0.90	0.00	0.00
ARL4A	10124	2.28	9.14	2.28	3.41	1.01	0.00	0.00
ARL5A	26225	7.73	13.48	3.4	5.88	1.10	0.00	0.00
ARL5B	221079	2.91	11.27	1.82	3.76	1.33	0.00	0.00
ARL6	84100	0.17	0.48	0.1	0.13	1.57	0.00	0.00
ARMC1	55156	5.31	9.53	3.1	5.08	0.87	0.00	0.00
ARMC5	79798	28.22	7.79	10.03	11.53	0.76	0.00	0.00
ARMC6	93436	102.51	41.89	39.82	40.87	0.81	0.00	0.00
ARMCX3	51566	3.14	7.08	3.22	3.21	0.64	0.00	0.00
ARMCX5	64860	1.51	4.85	1.28	1.81	1.03	0.00	0.00
ARMT1	79624	6.49	14.75	2.94	6.41	1.14	0.00	0.00
ARNTL2-AS1	101928646	0.44	2.21	0.53	0.23	1.79	0.00	0.00
ARRDC3	57561	12.08	14.53	16.48	14.48	0.93	0.00	0.00
ARSK	153642	0.77	2.18	0.71	0.77	0.93	0.00	0.00
ARV1	64801	8.29	10.87	5.09	6.29	0.72	0.00	0.00
AS3MT	57412	0	0.03	0	0	0.63	0.00	0.00
ASB4	51666	2.34	7.14	3.77	2.51	0.81	0.00	0.00
ASCC3	10973	1.15	7.17	1.5	2.85	1.14	0.00	0.00
ASDURF	110599588	4.78	4.28	0.37	2.1	1.89	0.00	0.00
ASF1A	25842	10.13	15.99	5.99	6.77	1.03	0.00	0.00
ASF1B	55723	90.11	67.68	50.94	46.85	0.69	0.00	0.00
ASH1L	55870	2.14	2.08	0.63	0.8	0.99	0.00	0.00
ASNSD1	54529	5.76	18.14	4.62	6.48	1.10	0.00	0.00
ASPM	259266	0.34	1.35	0.18	0.6	1.19	0.00	0.00
ATAD1	84896	2.09	6.09	1.99	3.1	0.75	0.00	0.00
ATAD2	29028	2.8	14.14	1.85	4.71	1.32	0.00	0.00
ATAD2B	54454	0.28	1.14	0.24	0.5	0.90	0.00	0.00
ATAD3A	55210	0	0.05	0	0	0.77	0.00	0.00
ATAD3B	83858	79.06	34.08	26.3	33.14	0.86	0.00	0.00

ATAD5	79915	0.11	0.58	0.32	0.29	1.01	0.00	0.00
ATE1	11101	1.79	7.22	1.82	3.51	0.76	0.00	0.00
ATF1	466	3.99	5.91	2.24	2.58	0.78	0.00	0.00
ATF2	1386	62.7	40.13	27.19	33.79	0.73	0.00	0.00
ATG12	9140	3.34	6.49	2.59	3.11	0.77	0.00	0.00
ATG2B	55102	0.17	1.81	0.31	0.57	0.99	0.00	0.00
ATG5	9474	3.78	8.88	3.58	4.94	0.72	0.00	0.00
ATL2	64225	4.85	15.98	5.47	7.53	0.67	0.00	0.00
ATM	472	0	0	0	0	1.50	0.00	0.00
ATP10D	57205	0.19	0.41	0.11	0.14	1.33	0.00	0.00
ATP11B	23200	0.44	1.41	0.64	0.79	0.72	0.00	0.00
ATP11C	286410	0.69	2.79	0.53	1.02	1.16	0.00	0.00
ATP13A3	79572	1.05	6.67	1.24	2.41	1.07	0.00	0.00
ATP2A3	489	0.73	0.47	0.28	0.16	1.40	0.00	0.00
ATP2B1	490	2.06	5.46	3.82	2.95	0.72	0.00	0.00
ATP5F1D	513	398.37	125.57	177.61	163.22	0.65	0.00	0.00
ATP6V0B	533	176.68	50.58	68.69	70.93	0.73	0.00	0.00
ATP6V1C1	528	3.4	10.97	2.49	4.69	0.94	0.00	0.00
ATP7A	538	0.14	0.74	0.22	0.21	0.91	0.00	0.00
ATP8A1	10396	0.18	1.04	0.22	0.4	0.97	0.00	0.00
ATP8A2	51761	0.03	0.36	0.07	0.07	3.03	0.00	0.00
ATR	545	0.37	1.68	0.39	0.5	1.39	0.00	0.00
AZI2	64343	4.08	7.06	4	5.01	0.64	0.00	0.00
B3GLCT	145173	0.57	1.96	0.79	0.82	0.65	0.00	0.00
B4GALNT3	283358	9.22	5.9	4.7	4.8	0.62	0.00	0.00
B4GALT1-AS1	101929639	0	0.02	0	0	1.36	0.00	0.00
B4GALT2	8704	102.52	40.84	50.52	42.54	0.63	0.00	0.00
BAAT	570	1.06	3.38	0.86	1.42	1.10	0.00	0.00
BACH1	571	0.41	3.3	0.85	1.22	0.71	0.00	0.00
BAD	572	57.86	19.57	19.14	21.11	0.97	0.00	0.00
BAG2	9532	12.57	21.36	6.84	10.48	0.96	0.00	0.00
BAK1	578	57.99	23.89	25.75	23.1	0.76	0.00	0.00
BARD1	580	0.49	2.24	0.57	0.64	1.32	0.00	0.00

BAZ1A	11177	1.14	2.42	1.09	1.5	0.69	0.00	0.00
BAZ1B	9031	3.7	17.96	5.21	6.18	0.91	0.00	0.00
BBIP1	92482	1.93	3.38	1.27	2.05	0.64	0.00	0.00
BBS10	79738	0	1.61	0.18	0.46	1.29	0.00	0.00
BBS7	55212	2.44	2.84	1	1.35	1.14	0.00	0.00
BBX	56987	0.45	2.79	0.51	0.72	1.39	0.00	0.00
BCAP29	55973	2.81	3.74	1.45	2.53	0.73	0.00	0.00
BCAS2	10286	9.14	14.62	5.96	8.69	0.69	0.00	0.00
BCAT1	586	2.07	9.27	2.47	2.93	1.07	0.00	0.00
BCCIP	56647	13.05	32.9	8.65	15.12	0.95	0.00	0.00
BCL2	596	0.49	0.86	0.4	0.56	0.77	0.00	0.00
BCLAF1	9774	4.86	20.14	2.96	5.92	1.46	0.00	0.00
BDP1	55814	17.22	31.73	13.14	14.45	0.71	0.00	0.00
BHLHA15	168620	14.51	5.87	5.82	6.68	0.73	0.00	0.00
BIRC2	329	11.09	56.67	8.68	13.65	1.62	0.00	0.00
BIRC3	330	0.13	0.51	0.06	0.07	1.62	0.00	0.00
BIRC6	57448	1.57	4.91	1.15	1.75	1.04	0.00	0.00
BIVM	54841	0.16	4.39	0.83	0.91	1.36	0.00	0.00
BIVM-ERCC5	100533467	1.37	2.21	1.17	0.94	0.73	0.00	0.00
BLM	641	0.79	2.93	0.6	0.82	1.53	0.00	0.00
BLOC1S5	63915	2.79	8.49	1.8	3.49	1.08	0.00	0.00
BLOC1S6	26258	1.56	5.87	1.67	3.17	0.61	0.00	0.00
BMI1	648	6.18	10.9	2.58	7.78	0.71	0.00	0.00
BMPR1A	657	1.71	9	2.74	3.22	0.75	0.00	0.00
BMPR2	659	0.63	2.76	1.05	1.12	0.60	0.00	0.00
BMS1	9790	3.12	12.02	3.46	4.19	1.16	0.00	0.00
BNIP2	663	2.27	7.17	2.75	3.48	0.67	0.00	0.00
BNIP3	664	0	0	0	0.04	0.59	0.00	0.00
BNIP3L	665	2.82	14.01	5.58	4.41	0.74	0.00	0.00
BOC	91653	6.15	5.38	3.95	3.87	0.63	0.00	0.00
BORA	79866	2.5	6.96	1.55	1.7	1.40	0.00	0.00
BPGM	669	1.06	2.91	1.36	0.72	0.94	0.00	0.00
BRCA1	672	0.16	0.29	0.67	0.63	1.65	0.00	0.00

BRCA2	675	0.2	1.24	0.29	0.37	1.46	0.00	0.00
BRCC3	79184	0	0.04	0	0	0.78	0.00	0.00
BRICD5	283870	19.55	5.32	7.23	8.46	0.67	0.00	0.00
BRIP1	83990	0.7	2.29	0.35	0.67	1.50	0.00	0.00
BRIX1	55299	10.95	23.89	6.4	11.88	0.78	0.00	0.00
BRMS1	25855	45.54	26.76	16.07	10.6	1.45	0.00	0.00
BROX	148362	1.33	3.39	0.94	1.91	0.73	0.00	0.00
BRWD3	254065	0.12	0.92	0.16	0.27	1.02	0.00	0.00
BST1	683	1.41	1.15	0.32	0.53	1.62	0.00	0.00
BTAF1	9044	0.45	5.17	0.97	1.38	1.23	0.00	0.00
BTBD1	53339	6.45	13.62	4.2	6.43	0.91	0.00	0.00
BUB1	699	5.7	22.87	5.18	6.79	1.23	0.00	0.00
BUB1B	701	4.24	18.03	4.28	5.77	1.14	0.00	0.00
BZW1	9689	31.4	107.18	27.64	46.17	0.88	0.00	0.00
C10orf91	170393	20.21	11.04	6.68	9.06	1.00	0.00	0.00
C11orf74	119710	6.13	9.65	5.53	5.11	0.63	0.00	0.00
C12orf29	91298	2.28	2.57	1.21	1.64	0.79	0.00	0.00
C12orf4	57102	1.16	2.86	0.73	0.79	1.31	0.00	0.00
C12orf60	144608	0.59	1.06	0.39	0.68	0.85	0.00	0.00
C14orf178	283579	0	0	0	0	1.43	0.00	0.00
C15orf39	56905	38.73	23.99	21.85	18.68	0.63	0.00	0.00
C16orf87	388272	1.4	4.38	1.69	0.72	1.15	0.00	0.00
C16orf89	146556	3.47	2.34	1.64	0.67	1.34	0.00	0.00
C17orf82	388407	2.08	1.41	0.98	0.45	1.29	0.00	0.00
C18orf54	162681	0.09	0.84	0.07	0.17	2.12	0.00	0.00
C19orf24	55009	0.02	0	0	0	1.01	0.00	0.00
C19orf25	148223	75.28	24.19	19.65	20.19	1.03	0.00	0.00
C19orf47	126526	19.52	9.12	8.46	7.8	0.81	0.00	0.00
C19orf48	84798	178.81	62.87	65.31	67.98	0.90	0.00	0.00
C19orf70	125988	91.58	39.22	46.56	39.05	0.63	0.00	0.00
C1D	10438	12.02	15.93	7.79	8.77	0.71	0.00	0.00
C1GALT1	56913	2.67	6.75	2.12	3.47	0.63	0.00	0.00
C1orf112	55732	0	0.08	0.06	0.04	0.69	0.00	0.00

C1orf122	127687	122.98	24.22	24.09	37.42	1.16	0.00	0.00
C1orf159	54991	21.78	8.11	9.36	8.88	0.68	0.00	0.00
C1orf52	148423	2.16	3.61	2.06	1.92	0.61	0.00	0.00
C1QTNF6	114904	16.94	8.84	6.02	6.89	1.02	0.00	0.00
C20orf27	54976	258.7	91.65	116.73	112.11	0.63	0.00	0.00
C21orf91	54149	0.4	0.78	0.23	0.18	1.34	0.00	0.00
C2CD4A	145741	1.95	1.42	0.76	1.32	0.70	0.00	0.00
C2CD5	9847	0.82	4.23	0.84	1.18	1.39	0.00	0.00
C2orf69	205327	1.77	4.97	0.91	1.91	1.27	0.00	0.00
C2orf74	339804	1.22	4.23	0.76	1.81	1.15	0.00	0.00
C3orf38	285237	0	0.02	0	0.01	0.90	0.00	0.00
C3orf84	646498	0.88	0.03	0.06	0.12	2.67	0.00	0.00
C4orf46	201725	2.48	7.7	2.89	3.43	0.68	0.00	0.00
C4orf48	401115	57.76	26.55	32.96	21.4	0.71	0.00	0.00
C5	727	4.53	6.7	6.58	7.6	0.59	0.00	0.00
C6	729	0.31	0.73	0.19	0.22	1.40	0.00	0.00
C7orf50	84310	116.69	57.11	52.46	51.07	0.68	0.00	0.00
C8orf58	541565	2.23	1.86	1.37	0.67	1.02	0.00	0.00
C8orf59	401466	24.94	10.97	4.17	6.89	1.31	0.00	0.00
C8orf82	414919	3.23	6.16	4.31	3.65	0.71	0.00	0.00
C9orf116	138162	27.88	14.94	10.92	12.75	0.71	0.00	0.00
C9orf147	100133204	0.04	0.1	0.08	0.07	0.85	0.00	0.00
C9orf24	84688	4.87	2.25	1.84	1.19	1.05	0.00	0.00
CACFD1	11094	1.31	0.9	0.89	0.35	0.82	0.00	0.00
CACNB4	785	0.28	1.32	0.25	0.5	1.06	0.00	0.00
CACYBP	27101	40.22	65.13	29.39	35.28	0.70	0.00	0.00
CALD1	800	5.75	24.26	7.4	11.75	0.64	0.00	0.00
CAMSAP2	23271	0.21	1.63	0.33	0.51	1.17	0.00	0.00
CAND1	55832	2.82	13.12	3.07	5.02	0.95	0.00	0.00
CAPN15	6650	39.93	17.39	16.87	16.35	0.81	0.00	0.00
CAPN7	23473	2.01	6.66	1.63	2.11	1.16	0.00	0.00
CAPZA1	829	17.34	37.85	13.71	22.02	0.63	0.00	0.00
CAPZA2	830	51.43	61.72	56.13	56.97	0.93	0.00	0.00

CARD6	84674	0	0	0	0	0.66	0.00	0.00
CARD8	22900	1.05	3.08	1.81	1.74	0.64	0.00	0.00
CARNMT1	138199	0.74	3.3	0.42	1.15	1.40	0.00	0.00
CASC10	399726	9.66	6.3	4.52	4.97	0.68	0.00	0.00
CASC4	113201	4.58	9.53	6.31	6.33	0.66	0.00	0.00
CASC9	101805492	0.02	0.04	0.01	0.02	1.09	0.00	0.00
CASD1	64921	4.44	4.6	4.46	3.7	0.71	0.00	0.00
CASK	8573	1.76	9.15	2.72	3.08	0.68	0.00	0.00
CASP3	836	7.2	13.63	2.58	6.37	1.21	0.00	0.00
CASP7	840	3.1	8.01	2.98	3.69	0.71	0.00	0.00
CASP8AP2	9994	0.18	0.97	0.14	0.22	1.67	0.00	0.00
CAVIN2	8436	0	2.18	0	0.16	3.46	0.00	0.00
CBARP	255057	8.53	5.01	4.69	3.73	0.65	0.00	0.00
CBR4	84869	1.81	5.67	1.8	2.5	0.84	0.00	0.00
CBX2	84733	11.51	13.26	8.54	5.59	0.80	0.00	0.00
CBX3	11335	15.54	40.66	13.43	19.19	0.78	0.00	0.00
CBX6	23466	6.38	11.72	7.04	4.4	0.66	0.00	0.00
CCAR1	55749	2.19	10.61	1.82	5.23	0.72	0.00	0.00
CCDC126	90693	1.11	1.22	0.74	0.71	1.12	0.00	0.00
CCDC138	165055	1.02	2.92	0.56	1.31	1.17	0.00	0.00
CCDC14	64770	0.48	3.41	0.46	1.07	1.19	0.00	0.00
CCDC150	284992	0.3	1.6	0.49	0.57	1.04	0.00	0.00
CCDC152	100129792	0.22	1.09	0.23	0.41	1.02	0.00	0.00
CCDC167	154467	0.02	0.2	0.04	0.04	0.94	0.00	0.00
CCDC173	129881	0.25	0.51	0.17	0.22	1.32	0.00	0.00
CCDC18	343099	0.52	0.95	0.3	0.61	0.61	0.00	0.00
CCDC183-AS1	100131193	4.11	2.52	1.74	1.8	0.68	0.00	0.00
CCDC191	57577	0.03	0.4	0.14	0.04	2.39	0.00	0.00
CCDC30	728621	0.22	0.9	0.28	0.12	1.39	0.00	0.00
CCDC34	91057	0	0	0	0	0.96	0.00	0.00
CCDC47	57003	6.24	29.54	5.97	10.62	0.89	0.00	0.00
CCDC59	29080	5.22	11.15	4.32	6.14	0.64	0.00	0.00
CCDC66	285331	0	0.01	0	0	1.10	0.00	0.00

CCDC68	80323	0.51	1.61	0.38	0.38	1.25	0.00	0.00
CCDC7	79741	19.77	99.73	21.22	27.82	1.26	0.00	0.00
CCDC77	84318	84.81	37.24	39.71	41.35	1.80	0.00	0.00
CCDC82	79780	1.29	2.49	0.75	1.32	0.94	0.00	0.00
CCDC85B	11007	118.18	38.86	50.64	46.86	0.69	0.00	0.00
CCDC88A	55704	0.84	3.34	0.9	1.42	1.08	0.00	0.00
CCHCR1	54535	9.82	15.54	11.36	12.2	1.13	0.00	0.00
CCNA2	890	25.3	40.36	15.4	22.69	0.80	0.00	0.00
CCNC	892	14.9	23.05	8.31	10.41	1.03	0.00	0.00
CCNE2	9134	0.32	1.25	0.07	0.34	1.96	0.00	0.00
CCNH	902	15.79	19.97	9.56	13.72	0.66	0.00	0.00
CCNO	10309	4.15	1.52	1.02	1.25	1.29	0.00	0.00
CCNT1	904	0.56	3.22	0.96	1.21	0.83	0.00	0.00
CCNT2	905	0.6	3.05	0.78	1.35	0.88	0.00	0.00
CCP110	9738	0.21	1.9	0.32	0.35	1.52	0.00	0.00
CCT6A	908	41.77	105.59	40.26	50.3	0.69	0.00	0.00
CD248	57124	3.96	2.46	2.29	1.99	0.59	0.00	0.00
CD2AP	23607	1.31	6.96	1.57	2.83	1.09	0.00	0.00
CD300C	10871	0.31	0.57	0.11	0.07	2.22	0.00	0.00
CD320	51293	417.14	142.05	169.99	152.38	0.81	0.00	0.00
CD36	948	4.66	11.73	2.15	2.6	1.62	0.00	0.00
CD8A	925	2.12	0.36	0.24	0.12	3.74	0.00	0.00
CD8B	926	0.77	1.94	0.38	0.16	2.40	0.00	0.00
CDC27	996	3.64	16.06	3.43	6.19	1.00	0.00	0.00
CDC37L1	55664	1.22	2.96	0.66	0.88	1.25	0.00	0.00
CDC40	51362	0.43	0.26	0.19	0.06	1.11	0.00	0.00
CDC42BPA	8476	0.34	2.33	0.62	0.91	0.74	0.00	0.00
CDC5L	988	30.74	14.76	10.82	11.45	1.63	0.00	0.00
CDC7	8317	0.36	2.55	0.39	0.96	1.10	0.00	0.00
CDCA2	157313	1.6	5.23	1	1.6	1.33	0.00	0.00
CDCA3	83461	32.82	21.76	16.33	18.46	0.64	0.00	0.00
CDCA7	83879	9.62	27.39	13.1	11.03	0.61	0.00	0.00
CDH16	1014	15.35	11.05	9.16	7.3	0.67	0.00	0.00

CDH2	1000	4.68	23.82	2.98	2.25	2.41	0.00	0.00
CDIP1	29965	176.06	69.76	73.26	83.13	0.66	0.00	0.00
CDK1	983	16.02	24.87	7.33	9.95	1.23	0.00	0.00
CDK14	5218	0.6	2.25	0.54	0.45	1.52	0.00	0.00
CDK17	5128	1.02	3.39	0.92	1.49	0.97	0.00	0.00
CDK3	1018	109.44	24.48	38.54	40.34	6.02	0.00	0.00
CDK6	1021	0.31	7.29	0.93	2.43	1.22	0.00	0.00
CDK8	1024	1.57	7.84	2.34	2.8	0.87	0.00	0.00
CDKN2AIP	55602	1.54	4.34	1.13	1.81	0.99	0.00	0.00
CDKN3	1033	29.74	46.31	20.93	21.54	0.82	0.00	0.00
CDX2	1045	2.84	2.09	1.23	1.24	1.00	0.00	0.00
CEBPA	1050	280.84	80.33	97.74	136.88	0.63	0.00	0.00
CEBPZ	10153	2.6	9.11	1.18	3.17	1.58	0.00	0.00
CENPC	1060	0.36	0.95	0.18	0.5	1.04	0.00	0.00
CENPE	1062	0.12	0.56	0.11	0.26	0.86	0.00	0.00
CENPF	1063	0.43	3.59	0.72	1.2	1.03	0.00	0.00
CENPI	2491	0.72	2.14	0.33	0.48	1.64	0.00	0.00
CENPJ	55835	0.27	2.24	0.46	0.37	1.53	0.00	0.00
CENPK	64105	4.23	6.22	0.87	1.91	1.80	0.00	0.00
CENPL	91687	0.89	2.43	1.26	1.13	0.75	0.00	0.00
CENPM	79019	72.46	32.5	27.66	33.41	0.76	0.00	0.00
CENPO	79172	19.59	16.8	9.42	11.38	0.72	0.00	0.00
CENPQ	55166	2.81	2.19	0.5	0.78	1.93	0.00	0.00
CENPU	79682	1.18	2.43	0.6	1.12	1.07	0.00	0.00
CEP120	153241	0.52	1.97	0.36	0.75	1.12	0.00	0.00
CEP128	145508	0.07	0.63	0.05	0.11	2.15	0.00	0.00
CEP135	9662	0.13	0.49	0.08	0.21	2.08	0.00	0.00
CEP152	22995	0.09	0.76	0.02	0.23	1.70	0.00	0.00
CEP162	22832	0.07	0.42	0.03	0.1	2.02	0.00	0.00
CEP170	9859	0.27	1.87	0.49	0.49	1.12	0.00	0.00
CEP170B	283638	18.06	8.1	8.62	9.13	0.72	0.00	0.00
CEP192	55125	0.19	1.61	0.5	0.7	0.59	0.00	0.00
CEP290	80184	0.02	0.31	0.09	0.12	1.37	0.00	0.00

CEP350	9857	0.14	0.88	0.11	0.26	1.40	0.00	0.00
CEP55	55165	1.95	6.77	1.16	1.95	1.47	0.00	0.00
CEP57	9702	2.06	7.03	2.36	2.42	0.82	0.00	0.00
CEP57L1	285753	0.71	1.99	0.16	0.61	1.66	0.00	0.00
CEP70	80321	1.09	2.74	0.88	0.93	1.05	0.00	0.00
CEP76	79959	1.56	4.22	1.5	2.15	0.66	0.00	0.00
CEP78	84131	1.18	4.81	1.14	1.95	0.94	0.00	0.00
CEP83	51134	0.32	0.99	0.26	0.34	1.04	0.00	0.00
CEP85L	387119	0.1	0.55	0.18	0.1	1.33	0.00	0.00
CEP97	79598	0.03	0.33	0.01	0.05	2.49	0.00	0.00
CERS6	253782	5.7	8.92	5.47	5.83	0.74	0.00	0.00
CETN3	1070	4.48	6.58	1.64	1.91	1.63	0.00	0.00
CFAP410	755	22.03	7.75	9.62	8.6	0.65	0.00	0.00
CFAP44	55779	0.06	0.57	0.08	0.21	1.52	0.00	0.00
CFAP97	57587	1.56	6.62	2.07	1.99	0.77	0.00	0.00
CGRRF1	10668	2.96	3.26	1.74	1.92	0.82	0.00	0.00
CHAC2	494143	0.16	0.08	0	0	0.68	0.00	0.00
CHCHD5	84269	69.63	27.95	36.57	26.12	0.60	0.00	0.00
CHD1	1105	0.89	3.1	0.93	1.45	0.89	0.00	0.00
CHD9	80205	1.01	6.48	1.57	2.86	1.10	0.00	0.00
CHEK1	1111	3.16	11.13	3.16	4.41	0.96	0.00	0.00
CHM	1121	0.57	2.47	0.52	0.93	1.04	0.00	0.00
CHMP1A	5119	175.24	65.45	61.95	61.37	0.97	0.00	0.00
CHMP2B	25978	4.77	9.42	3	6.31	0.59	0.00	0.00
CHMP5	51510	17.55	22.65	13.45	11.19	0.65	0.00	0.00
CHMP6	79643	64.27	27.18	28.58	26.31	0.71	0.00	0.00
CHORDC1	26973	2.07	6.53	0.87	1.55	1.76	0.00	0.00
CHRNA5	1138	1.09	2.27	1.17	0.95	0.67	0.00	0.00
CHST10	9486	5.62	6.63	4.75	3.3	0.59	0.00	0.00
CHST9	83539	2.49	5.37	0.61	1.67	1.46	0.00	0.00
CHSY3	337876	0.17	0.8	0.34	0.16	1.22	0.00	0.00
CHUK	1147	4.14	12.54	6.64	5.67	1.04	0.00	0.00
CIAO3	64428	34.34	12.91	14.09	14.61	0.71	0.00	0.00

CIB2	10518	4.21	2.92	3.18	1.47	0.62	0.00	0.00
CIP2A	57650	0.78	2.1	0.36	0.78	1.34	0.00	0.00
CIR1	9541	2.77	5.07	1.26	2.16	1.26	0.00	0.00
CKAP2	26586	1.59	6.82	0.44	3.08	1.28	0.00	0.00
CKAP2L	150468	0.01	0.04	0	0.02	1.46	0.00	0.00
CKAP5	9793	5.67	33.4	15.66	15.76	0.62	0.00	0.00
CLASP2	23122	0.54	4.05	1.05	1.52	0.92	0.00	0.00
CLCN3	1182	0.99	6.26	1.68	2.15	0.77	0.00	0.00
CLGN	1047	0.87	3.63	0.57	1.1	1.40	0.00	0.00
CLINT1	9685	6.16	20.3	5.98	8.18	0.65	0.00	0.00
CLK4	57396	0.3	1.64	0.42	0.85	0.90	0.00	0.00
CLMAT3	101927096	2.46	4.39	2.39	2.1	0.60	0.00	0.00
CLOCK	9575	0.31	1.42	0.51	0.57	0.70	0.00	0.00
CLPP	8192	187.27	79.37	94	83.76	0.60	0.00	0.00
CLPX	10845	5.31	13.08	5.11	2.03	1.09	0.00	0.00
CLSPN	63967	0	0	0	0.03	1.16	0.00	0.00
CLTC	1213	10.81	68.43	16.89	29.9	0.72	0.00	0.00
CLUAP1	23059	23.06	28.7	27.51	29.35	0.84	0.00	0.00
CLUH	23277	92.86	57.57	45.18	44.13	0.77	0.00	0.00
CMPK1	51727	16.72	36.49	12.73	21.49	0.62	0.00	0.00
CMSS1	84319	11.38	20.4	11.28	9.35	0.63	0.00	0.00
CMTR2	55783	0.39	1.82	0.46	0.82	0.80	0.00	0.00
CNOT6L	246175	0.46	2.79	0.92	1.16	0.64	0.00	0.00
CNPY3-GNMT	107080644	0.52	0.14	0	0	4.97	0.00	0.00
CNTLN	54875	0.21	0.86	0.08	0.29	2.09	0.00	0.00
COBLL1	22837	3.58	17.54	3.43	9.66	0.58	0.00	0.00
COG3	83548	5.94	7.79	4.26	5.94	0.63	0.00	0.00
COG6	57511	0.97	4.44	1.19	1.4	1.00	0.00	0.00
COL2A1	1280	1.54	0.7	0.57	0.4	1.23	0.00	0.00
COL4A3BP	10087	0.52	3.98	1.36	1.7	0.62	0.00	0.00
COMMD3-BMI1	100532731	161.77	110.54	94.01	105.01	1.39	0.00	0.00
COMMD4	54939	0.85	2.92	0.43	0.59	0.60	0.00	0.00
COMMD5	28991	39.62	15.5	17.17	18.01	0.64	0.00	0.00

COMMD8	54951	2.8	2.44	1.01	1.62	1.00	0.00	0.00
COPB1	1315	10.58	36.62	9.95	17.59	0.76	0.00	0.00
COPB2	9276	0	0	0.15	0.15	0.82	0.00	0.00
COPS2	9318	7.36	14.74	2.8	5.91	1.26	0.00	0.00
COPS4	51138	12.02	13.3	6.74	8.93	0.69	0.00	0.00
COPS5	10987	11.49	20.89	10.2	11.14	0.60	0.00	0.00
COPZ2	51226	3.06	1.4	0.96	0.79	1.32	0.00	0.00
COQ2	27235	8.81	10.21	5.42	7.15	0.63	0.00	0.00
CPD	1362	3.23	25.58	6.37	9.26	0.75	0.00	0.00
CPLANE1	65250	0.03	0	0	0.01	1.06	0.00	0.00
CPNE3	8895	3.92	10.64	2.92	5.18	0.84	0.00	0.00
CPO	130749	2.06	3.07	0.81	1.53	1.02	0.00	0.00
CPSF2	53981	2.54	10.25	2.7	3.49	0.94	0.00	0.00
CPTP	80772	69.84	24.72	29.01	34.46	0.59	0.00	0.00
CPZ	8532	2.5	1.52	1.34	0.69	1.02	0.00	0.00
CRBN	51185	3.72	4.81	2.11	2.75	0.86	0.00	0.00
CREB1	1385	1.18	5.11	2.06	1.83	1.01	0.00	0.00
CREBZF	58487	0.04	0.05	0	0.17	0.77	0.00	0.00
CREM	1390	8.23	10.52	5.66	6.68	0.60	0.00	0.00
CRK	1398	5.75	22.98	7.93	9.15	0.69	0.00	0.00
CRNKL1	51340	3.05	12.53	2.79	4.45	1.00	0.00	0.00
CROCCP3	114819	0.16	0.42	0.17	0.15	0.68	0.00	0.00
CRYBB2	1415	0.16	0.25	0.06	0.03	2.35	0.00	0.00
CRYBG3	131544	0.04	0.45	0.03	0.14	1.58	0.00	0.00
CRYZL1	9946	1.89	4.62	2.83	2.96	1.00	0.00	0.00
CSDE1	7812	20.15	85.76	27.25	35.61	0.89	0.00	0.00
CSE1L	1434	15.42	80.77	14.84	28.86	1.12	0.00	0.00
CSGALNACT2	55454	0.44	2.08	0.59	0.81	0.83	0.00	0.00
CSNK1G3	1456	0.75	3.46	0.97	1.61	0.67	0.00	0.00
CSPP1	79848	0.4	1.69	0.35	0.78	0.81	0.00	0.00
CSRNP1	64651	14.74	7.57	6.82	8.24	0.61	0.00	0.00
CSTF3	1479	12.31	19.8	11.66	12.15	0.64	0.00	0.00
CTAGE15	441294	0.21	0.5	0.19	0.27	0.80	0.00	0.00

CTDSP1	58190	201.05	79.38	75.83	90.73	0.78	0.00	0.00
CTDSPL2	51496	1.47	4.54	0.99	1.35	1.09	0.00	0.00
CTNNAL1	8727	8.93	40.78	12.7	20.23	0.58	0.00	0.00
CTR9	9646	1.46	6.79	1.52	2.39	1.05	0.00	0.00
CTRC	11330	0.07	0.06	0	0.02	1.95	0.00	0.00
CUL1	8454	6.86	20.98	7.44	10.11	0.68	0.00	0.00
CUL2	8453	1.51	8.37	1.5	3.08	1.09	0.00	0.00
CUL3	8452	7.04	24.87	7.57	10.09	0.80	0.00	0.00
CUL4B	8450	1.97	7.78	2.03	3.77	0.83	0.00	0.00
CUL5	8065	1.79	2.97	1.07	1.78	0.89	0.00	0.00
CWC22	57703	1.03	3.31	0.7	1.54	0.94	0.00	0.00
CWC27	10283	3.43	7.1	1.85	2	1.26	0.00	0.00
CX3CL1	6376	0	0.09	0	0	0.63	0.00	0.00
CXADR	1525	6.78	23.67	9.49	12.38	0.63	0.00	0.00
CYB561	1534	6.59	5.34	4.33	3.66	0.60	0.00	0.00
CYB5R4	51167	3.37	4.49	1.68	2.5	0.88	0.00	0.00
CYC1	1537	421.1	135.49	182.42	159.28	0.71	0.00	0.00
CYGB	114757	26.13	1.14	1.62	1.49	3.15	0.00	0.00
CYP4F30P	100132708	0.03	0.09	0.01	0.01	3.38	0.00	0.00
CYP4F35P	284233	0	0.21	0.01	0.01	1.95	0.00	0.00
CYP8B1	1582	1.27	0.98	0.54	0.9	0.64	0.00	0.00
CYSRT1	375791	26.13	6.52	8.97	8.75	0.86	0.00	0.00
DAB2	1601	0.89	1.36	0.22	0.68	1.16	0.00	0.00
DAPK3	1613	46.67	23.36	25.18	21.88	0.59	0.00	0.00
DBF4	10926	3.25	7.69	2.13	3.48	1.08	0.00	0.00
DBF4B	80174	8.24	9.96	5.39	5.16	0.82	0.00	0.00
DCAF13	25879	8.18	15.81	5.03	9.03	0.80	0.00	0.00
DCAF17	80067	1.35	5.57	1.81	2.34	0.79	0.00	0.00
DCDC1	341019	0.29	1.77	0.25	0.28	1.84	0.00	0.00
DCDC2	51473	1.46	8.66	2.7	4.03	0.64	0.00	0.00
DCLRE1A	9937	0.86	3.8	0.75	1.08	0.89	0.00	0.00
DCLRE1C	64421	0.56	2.18	0.82	1.02	0.79	0.00	0.00
DCP2	167227	3.78	5.6	2.49	2.73	1.00	0.00	0.00

DCUN1D1	54165	1.88	3.09	0.45	1.3	1.31	0.00	0.00
DCUN1D4	23142	0.98	3.92	0.93	1.33	1.15	0.00	0.00
DDI2	84301	1.83	12.36	3.38	5.15	0.66	0.00	0.00
DDIAS	220042	0.44	2.34	0.41	0.94	1.09	0.00	0.00
DDN	23109	5.76	0.74	0.29	1.61	1.78	0.00	0.00
DDX1	1653	12.59	47.46	11.96	16.41	1.07	0.00	0.00
DDX10	1662	1.87	7.3	1.92	2.91	0.91	0.00	0.00
DDX18	8886	5.63	22.5	5.1	7.78	1.10	0.00	0.00
DDX21	9188	0.24	0.16	0.72	0.48	0.82	0.00	0.00
DDX3X	1654	3.59	15.21	7	7.97	0.77	0.00	0.00
DDX3Y	8653	1.79	16.1	2.2	3.61	1.63	0.00	0.00
DDX46	9879	3.78	14.41	3.01	5.35	1.01	0.00	0.00
DDX49	54555	91.63	38.89	37.17	41.18	0.75	0.00	0.00
DDX50	79009	48.04	31.44	35.02	36.89	0.83	0.00	0.00
DDX52	11056	0.63	0.98	0.33	0.29	1.22	0.00	0.00
DDX58	23586	4.95	6.7	7.36	3.04	0.64	0.00	0.00
DEF8	54849	102.63	43.08	39.15	41.77	0.72	0.00	0.00
DEK	7913	18.5	42.76	7.75	16.62	1.32	0.00	0.00
DENND2D	79961	0.03	0.25	0.03	0.08	1.19	0.00	0.00
DENND4A	10260	0.39	3.96	0.82	1.19	0.85	0.00	0.00
DENND4C	55667	2.7	5.05	2.9	3.16	0.75	0.00	0.00
DENND5B	160518	0.68	2.13	0.86	0.88	0.60	0.00	0.00
DENR	8562	8.23	20.96	7.67	10	0.72	0.00	0.00
DEPDC1	55635	0.84	2.86	0.43	0.88	1.49	0.00	0.00
DEPDC1B	55789	9.58	17.78	6.11	9.4	0.82	0.00	0.00
DEPDC4	120863	0.75	1.06	0.44	0.23	1.62	0.00	0.00
DGCR6L	85359	85.02	22.91	26.87	22.74	1.15	0.00	0.00
DGKI	9162	19.34	16.59	13.11	10.9	0.64	0.00	0.00
DHFR	1719	12.23	27.3	8.06	10.86	1.05	0.00	0.00
DHFR2	200895	0.26	1.11	0.33	0.32	0.96	0.00	0.00
DHX15	1665	12.8	41.11	14.51	17.86	0.73	0.00	0.00
DHX29	54505	0.97	4.07	0.67	1.87	1.06	0.00	0.00
DHX34	9704	13.64	7.69	6.9	6.26	0.68	0.00	0.00

DHX40	79665	2.09	7.25	1.87	2.17	1.21	0.00	0.00
DHX57	90957	1.71	6.77	2.06	2.84	0.79	0.00	0.00
DHX9	1660	14.49	55.3	15	19.05	1.01	0.00	0.00
DIAPH3	81624	0.53	2.76	0.55	1.05	1.06	0.00	0.00
DICER1	23405	0.62	4.96	1.09	1.6	0.95	0.00	0.00
DIS3	22894	1.9	8.72	1.17	2.09	1.58	0.00	0.00
DIXDC1	85458	2.48	12.63	3.03	3.33	1.34	0.00	0.00
DLD	1738	2.35	9.79	2.09	3.16	1.14	0.00	0.00
DLEU2	8847	1.31	1.31	0.43	0.8	1.00	0.00	0.00
DLG1	1739	1.97	9.59	2.31	3.26	1.04	0.00	0.00
DLGAP5	9787	1.7	8.17	1.19	2.6	1.36	0.00	0.00
DLX4	1748	0.01	0	0	0	0.71	0.00	0.00
DMAC1	90871	0	0.1	0	0	0.80	0.00	0.00
DMTF1	9988	0.44	3.39	0.8	0.77	1.09	0.00	0.00
DMWD	1762	6.38	3.53	3.83	2.84	0.65	0.00	0.00
DMXL1	1657	0.21	1.12	0.27	0.5	0.72	0.00	0.00
DMXL2	23312	0.11	1.29	0.17	0.16	2.13	0.00	0.00
DNA2	1763	0.88	3.2	0.88	1.07	0.92	0.00	0.00
DNAAF2	55172	3.35	5.49	2.3	3.28	0.66	0.00	0.00
DNAAF4-CCPG1	100533483	1.03	1.97	0.47	0.46	1.03	0.00	0.00
DNAJA1	3301	30.9	89.55	28.46	30.54	1.04	0.00	0.00
DNAJB14	79982	0.57	1.76	0.44	1.1	0.69	0.00	0.00
DNAJB4	11080	0.62	2.9	0.48	0.56	1.72	0.00	0.00
DNAJB9	4189	2.24	5.43	1.91	2.29	0.87	0.00	0.00
DNAJC1	64215	7.86	10.97	4.22	5.48	0.81	0.00	0.00
DNAJC10	54431	0	0.01	0	0	1.18	0.00	0.00
DNAJC13	23317	1.14	3.79	1.05	1.66	1.11	0.00	0.00
DNAJC21	134218	1.97	10.82	3.3	5.06	0.59	0.00	0.00
DNAJC25	548645	1.49	3.62	1.08	1.16	1.24	0.00	0.00
DNAJC3	5611	2.17	10.06	2.49	4.25	0.85	0.00	0.00
DNAJC7	7266	13.15	27.4	9.28	14.69	0.72	0.00	0.00
DND1	373863	0.17	0.64	0.23	0.17	1.21	0.00	0.00
DNM1L	10059	2.54	10.28	3.59	4.49	0.76	0.00	0.00

DNTTIP2	30836	1.52	7.07	1.15	2.59	1.19	0.00	0.00
DOC2B	8447	5.81	4.31	1.74	1.66	1.61	0.00	0.00
DOCK4	9732	0.53	6.84	2.07	2.28	0.72	0.00	0.00
DOCK7	85440	3.1	8.45	2.72	4.57	0.98	0.00	0.00
DOP1A	23033	0.16	1.15	0.31	0.49	0.63	0.00	0.00
DPH1	1801	61.61	31.56	28.28	29.06	0.71	0.00	0.00
DPH2	1802	70.62	26.28	27.3	35.25	0.64	0.00	0.00
DPH3	285381	7.04	7.16	3.81	5.63	0.59	0.00	0.00
DPM1	8813	40.05	68.84	26.48	33.78	0.82	0.00	0.00
DPP4	1803	15.54	55.37	14.5	24.96	0.81	0.00	0.00
DPP8	54878	0	0	0	0	0.65	0.00	0.00
DPY19L1P1	100129460	0.43	0.85	0.25	0.37	1.10	0.00	0.00
DPY19L3	147991	0.43	3.97	1.25	1.73	0.60	0.00	0.00
DPY19L4	286148	0.74	3.26	0.82	1.23	1.23	0.00	0.00
DPYSL4	10570	7.99	6.17	3.63	3.21	1.05	0.00	0.00
DSCC1	79075	1.9	5.79	1.16	1.69	1.43	0.00	0.00
DSE	29940	0.56	1.85	0.44	0.67	0.89	0.00	0.00
DSN1	79980	5.78	14.52	4.96	6.24	0.82	0.00	0.00
DTL	51514	4.07	13.4	4.28	6.13	0.83	0.00	0.00
DTWD1	56986	1.92	2.98	1.2	1.48	1.04	0.00	0.00
DTX4	23220	5.02	4.83	2.33	4.19	0.61	0.00	0.00
DUSP19	142679	0.18	0.65	0.25	0.1	1.05	0.00	0.00
DUSP9	1852	811.57	237.03	260.67	315.67	0.88	0.00	0.00
DYNC1I2	1781	9.55	28.09	9.96	11.9	0.69	0.00	0.00
DYNC1LI1	51143	8.93	11.41	5.74	5.67	0.80	0.00	0.00
DYNC2LI1	51626	4.22	7.06	1.92	2.74	1.27	0.00	0.00
DYNLT3	6990	1.35	2.31	0.96	0.88	0.95	0.00	0.00
DYRK3	8444	0.75	1.86	0.76	0.75	0.79	0.00	0.00
DZIP1	22873	1.29	4.58	0.95	0.95	1.43	0.00	0.00
DZIP1L	199221	0.21	0.52	0.09	0.05	2.60	0.00	0.00
DZIP3	9666	0.54	2.58	0.4	1.04	1.06	0.00	0.00
E2F1	1869	73.8	31.08	25.7	30.07	0.92	0.00	0.00
E2F7	144455	0.58	2.85	0.93	1.08	0.76	0.00	0.00

E2F8	79733	1.44	3.17	1.03	1.64	0.79	0.00	0.00
EAF2	55840	1.77	3.98	1.05	1.84	0.99	0.00	0.00
EBAG9	9166	5.16	8.21	4.43	4.83	0.62	0.00	0.00
ECHDC1	55862	7.34	14.76	4.19	8.13	0.82	0.00	0.00
ECSIT	51295	131.81	44.88	51.23	47.15	0.86	0.00	0.00
ECT2	1894	1.77	8.3	1.16	2.4	1.41	0.00	0.00
EDA2R	60401	1.57	4.11	1.75	1.5	0.62	0.00	0.00
EDEM3	80267	0.5	2.9	0.65	1.15	1.01	0.00	0.00
EDRF1	26098	0.46	1.84	0.53	0.81	0.73	0.00	0.00
EED	8726	2.82	7.44	3.63	3.29	0.60	0.00	0.00
EEF1AKMT4	110599564	62.73	26.1	25.2	26.14	0.80	0.00	0.00
EEF1E1	9521	20.87	26.17	11.51	12.89	0.98	0.00	0.00
EEF1E1-BLOC1S5	100526837	35.55	46.35	20.75	24.32	0.85	0.00	0.00
EFCAB11	90141	1.63	3.32	1.24	1.4	0.95	0.00	0.00
EFCAB7	84455	0.42	0.91	0.21	0.31	1.56	0.00	0.00
EFHD2	79180	117.03	87.15	75.56	59.45	0.60	0.00	0.00
EFL1	79631	1.07	3.45	0.95	1.5	1.05	0.00	0.00
EFL1P1	648809	0.01	0.08	0	0.01	3.21	0.00	0.00
EFNA4	1945	18.8	10.09	9.99	9.89	0.59	0.00	0.00
EFNB1	1947	2.95	3.05	1.67	1.48	0.93	0.00	0.00
EGFR	1956	0.93	2.04	0.91	0.86	0.75	0.00	0.00
EGLN2	112398	37.45	16.37	15.36	16.42	0.77	0.00	0.00
EIF1AX	1964	15.19	21.75	5.01	10.85	0.78	0.00	0.00
EIF1AY	9086	9.68	10.89	2.95	4.61	1.45	0.00	0.00
EIF2AK2	5610	1.26	5.48	1.48	2.25	0.86	0.00	0.00
EIF3A	8661	5.71	27.95	5.26	9.16	1.11	0.00	0.00
EIF3E	3646	0	0.02	0	0	1.10	0.00	0.00
EIF3J	8669	12.2	22.88	8.1	11.63	0.83	0.00	0.00
EIF4E	1977	15.52	30.15	10.49	15.72	0.76	0.00	0.00
EIF5	1983	11.8	38.77	11.86	13.93	0.82	0.00	0.00
EIF6	3692	903.03	319.68	391.13	369.53	0.70	0.00	0.00
ELF2	1998	1.02	3.77	0.99	1.48	0.81	0.00	0.00
ELK3	2004	0	0	0	0	0.91	0.00	0.00

ELMOD2	255520	1.62	5.34	1.06	2.65	0.97	0.00	0.00
ELOF1	84337	122.17	62.08	57.35	52.21	0.76	0.00	0.00
EMC2	9694	2.92	5.78	1.68	1.73	1.28	0.00	0.00
EMC6	83460	213.61	102.34	90.49	100.66	0.73	0.00	0.00
EML4	27436	16.18	100.28	35.88	38.11	0.63	0.00	0.00
EML5	161436	0.12	0.26	0.03	0.07	2.01	0.00	0.00
EML6	400954	0.31	2.24	0.65	0.64	1.18	0.00	0.00
ENAH	55740	0.76	7.36	2.4	2.41	0.72	0.00	0.00
ENDOG	2021	50.79	15.67	21.71	23.7	0.60	0.00	0.00
ENHO	375704	5.53	2.34	1.44	1.65	1.36	0.00	0.00
ENTPD7	57089	0.99	0.99	0.41	0.46	1.14	0.00	0.00
EP300-AS1	101927279	0.26	0.77	0.15	0.15	1.27	0.00	0.00
EPB41L2	2037	7.18	24.41	9.67	10.61	0.67	0.00	0.00
EPB41L5	57669	1.98	7.22	2.65	2.92	0.71	0.00	0.00
EPN3	55040	6.69	3.79	2.46	3.51	0.67	0.00	0.00
EPO	2056	13.18	3.95	3.76	4.96	1.03	0.00	0.00
EPOR	2057	0	0	0	0	2.06	0.00	0.00
EPRS	2058	6.05	28.78	7.75	8.89	1.22	0.00	0.00
EPS15	2060	1.02	5.53	2.03	2.15	0.59	0.00	0.00
ERAP2	64167	0.55	2.82	0.77	1.43	0.64	0.00	0.00
ERBIN	55914	0.67	3.82	0.81	1.57	0.91	0.00	0.00
ERCC4	2072	0.42	1.52	0.47	0.73	0.65	0.00	0.00
ERCC5	2073	0.78	4.25	0.89	1.94	0.82	0.00	0.00
ERCC6	2074	0.28	1.38	0.49	0.4	0.71	0.00	0.00
ERCC6L	54821	0.39	2.32	0.21	0.38	2.10	0.00	0.00
EREG	2069	0.4	3.77	0.69	0.28	2.09	0.00	0.00
ERGIC2	51290	3.92	5.88	1.39	2.4	1.31	0.00	0.00
ERI1	90459	1.94	4.36	1.61	2.01	0.89	0.00	0.00
ERI2	112479	1.52	3.79	1.15	1.45	0.97	0.00	0.00
ESCO1	114799	0.77	2.58	0.62	0.82	0.85	0.00	0.00
ESCO2	157570	0.7	2.56	0.42	0.76	1.44	0.00	0.00
ESF1	51575	2.95	3.55	0.98	2.36	0.99	0.00	0.00
ETAA1	54465	1.6	2.52	0.55	1.3	1.23	0.00	0.00

EVI5	7813	0.32	1.25	0.35	0.63	0.76	0.00	0.00
EVL	51466	3.62	1.21	1.13	1.15	1.16	0.00	0.00
EXO1	9156	2.24	7.97	1.55	2.48	1.45	0.00	0.00
EXOC1	55763	0.67	2.68	0.8	1.2	0.75	0.00	0.00
EXOC5	10640	0.81	3.57	0.43	0.75	1.73	0.00	0.00
EXOC6	54536	0.83	2.75	0.76	1.14	0.88	0.00	0.00
EXOSC4	54512	98.07	39.19	46.63	43.79	0.59	0.00	0.00
EXOSC9	5393	8.39	18.82	6.29	8.4	1.00	0.00	0.00
EXTL2	2135	2.37	5.65	1.73	2.16	1.08	0.00	0.00
EZH2	2146	6.42	14.98	4.81	5.86	0.92	0.00	0.00
FAAP100	80233	0.02	0.02	0.05	0	0.92	0.00	0.00
FADS2	9415	1.04	3.44	1.41	1.64	0.90	0.00	0.00
FAIM	55179	8.93	8.01	2.58	4.81	1.16	0.00	0.00
FAM107B	83641	8.43	24.74	8.75	12.67	0.62	0.00	0.00
FAM110A	83541	13.33	7.31	6.62	4.67	0.92	0.00	0.00
FAM122C	159091	0.57	2.03	0.66	0.74	0.79	0.00	0.00
FAM126A	84668	1.83	3.01	0.9	0.99	1.18	0.00	0.00
FAM133B	257415	3.69	8.23	3.71	4.07	0.58	0.00	0.00
FAM135A	57579	0.74	2.74	0.74	0.94	0.96	0.00	0.00
FAM13A	10144	0.67	2.73	0.61	0.73	1.24	0.00	0.00
FAM13B	51306	0.36	2.2	0.55	0.84	0.81	0.00	0.00
FAM161A	84140	0.3	1.27	0.3	0.3	1.29	0.00	0.00
FAM198B	51313	3.39	21.85	4.61	6.49	1.11	0.00	0.00
FAM199X	139231	1.1	5.53	1.53	1.53	1.06	0.00	0.00
FAM206A	54942	5.93	9.14	3.86	5.06	0.79	0.00	0.00
FAM208A	23272	1.05	3.63	0.93	1.5	1.04	0.00	0.00
FAM208B	54906	0.8	5.43	1.38	2.45	0.74	0.00	0.00
FAM210A	125228	0	0	0	0	0.73	0.00	0.00
FAM217B	63939	1.26	3.44	1.17	1.44	1.00	0.00	0.00
FAM229B	619208	15.01	10.39	6.92	3.7	1.44	0.00	0.00
FAM43B	163933	0.01	0.01	0	0	0.62	0.00	0.00
FAM47E	100129583	0	0.03	0	0	0.83	0.00	0.00
FAM69A	388650	1.04	4.46	1.25	2.28	0.60	0.00	0.00

FAM72B	653820	1.64	7.2	3.14	2.7	0.69	0.00	0.00
FAM72D	728833	0.91	4.31	1.81	1.58	0.63	0.00	0.00
FAM76B	143684	0.44	1.61	0.38	0.68	0.82	0.00	0.00
FAM78A	286336	1.5	2.39	1.24	0.93	0.81	0.00	0.00
FAM83H-AS1	100128338	1.74	1.14	0.37	0.66	1.46	0.00	0.00
FAM91A1	157769	1.65	6.9	1.76	3.32	0.73	0.00	0.00
FAM92A	137392	2.81	5.23	1.62	1.75	1.21	0.00	0.00
FAM98A	25940	9.13	30.65	11.69	13.82	0.63	0.00	0.00
FAM98B	283742	1.93	5.55	1.89	2.33	0.82	0.00	0.00
FAN1	22909	1.08	3.62	1.36	1.38	0.75	0.00	0.00
FANCB	2187	0.32	0.55	0.11	0.32	1.04	0.00	0.00
FANCG	2189	14.3	8.55	6.77	7.26	0.74	0.00	0.00
FANCI	55215	2.17	11.66	3.74	3.79	0.85	0.00	0.00
FANCL	55120	2.08	8.49	3.21	3.62	0.64	0.00	0.00
FANCM	57697	0.23	0.41	0.05	0.12	1.62	0.00	0.00
FARSA	2193	37.03	10.86	5.81	8.83	1.72	0.00	0.00
FASTKD1	79675	1.29	4.99	1.28	1.26	1.15	0.00	0.00
FASTKD2	22868	1.7	5.83	1.44	2.37	1.03	0.00	0.00
FASTKD3	79072	0.79	3.67	1.18	1.66	0.61	0.00	0.00
FBN1	2200	0.16	1.14	0.3	0.19	1.38	0.00	0.00
FBXL3	26224	1.72	4.28	1.1	1.82	0.99	0.00	0.00
FBXO11	80204	1.39	11.51	3.52	4.44	0.69	0.00	0.00
FBXO30	84085	0.59	3.13	0.74	1.22	0.88	0.00	0.00
FBXO5	26271	7.96	15.28	2.87	5.11	1.54	0.00	0.00
FBXW5	54461	0	0	0.28	0	0.72	0.00	0.00
FBXW7	55294	0.41	2.53	0.84	0.92	0.74	0.00	0.00
FBXW9	84261	21.72	8.68	11.2	8.83	0.61	0.00	0.00
FCGBP	8857	6.35	4.66	3.32	4.34	0.65	0.00	0.00
FDXR	2232	285.25	150.06	149.83	137.82	0.60	0.00	0.00
FEM1B	10116	1.07	3.19	0.96	1.51	0.72	0.00	0.00
FER	2241	0.38	1.72	0.48	0.53	1.05	0.00	0.00
FERMT2	10979	2.61	4.14	0.28	0.33	3.61	0.00	0.00
FGD6	55785	0.08	0.69	0.19	0.18	0.98	0.00	0.00

FGFR1OP	11116	3.54	9.94	3.72	5.25	0.65	0.00	0.00
FIGN	55137	0.15	1.08	0.34	0.49	0.60	0.00	0.00
FIGNL1	63979	0.81	1.99	0.33	0.44	1.53	0.00	0.00
FITM1	161247	51.24	15.13	15.56	15.91	1.09	0.00	0.00
FKBP14	55033	0.69	5.71	1.08	1.55	1.26	0.00	0.00
FKBP3	2287	15.86	33.79	11.9	16.39	0.73	0.00	0.00
FKTN	2218	0.27	1.88	0.41	0.63	1.02	0.00	0.00
FMR1	2332	0.81	3.86	1.01	1.76	0.75	0.00	0.00
FNBP1L	54874	1.44	6.59	2.62	3.75	0.81	0.00	0.00
FNDC10	643988	6.73	1.85	2.54	2.5	0.78	0.00	0.00
FNDC3A	22862	114.53	125.88	152.43	115.94	0.58	0.00	0.00
FNTB	2342	19.3	13.75	7.85	10.41	0.86	0.00	0.00
FOXN2	3344	0.72	2.87	0.75	1.52	0.71	0.00	0.00
FPGT-TNNI3K	100526835	0.15	1.19	0.23	0.72	1.04	0.00	0.00
FRA10AC1	118924	0	0	0	0.08	0.64	0.00	0.00
FRG1	2483	8.35	12.91	5.83	7.43	0.67	0.00	0.00
FRK	2444	0.61	2.32	0.6	0.53	1.25	0.00	0.00
FRMD8	83786	16.98	7.07	6.5	7.76	0.80	0.00	0.00
FRYL	285527	0.08	1.23	0.17	0.3	1.60	0.00	0.00
FUNDC1	139341	5.14	7.48	3.87	4.07	0.65	0.00	0.00
FUT10	84750	1.23	3.1	1.23	1.63	0.59	0.00	0.00
FXR1	8087	3.83	24.53	5.34	7.07	1.17	0.00	0.00
FYTTD1	84248	1.48	6.97	1.81	3.11	0.75	0.00	0.00
FZD1	8321	0.62	1.49	0.53	0.6	0.84	0.00	0.00
FZD6	8323	0.2	2.38	0.66	0.82	0.75	0.00	0.00
FZD9	8326	2.39	0.63	0.67	0.88	0.98	0.00	0.00
FZR1	51343	56.12	29.03	25.86	21.92	0.72	0.00	0.00
G2E3	55632	0.35	1.88	0.28	0.38	1.56	0.00	0.00
G3BP2	9908	11.36	42.36	14.98	18.43	0.68	0.00	0.00
GADD45GIP1	90480	271.24	105.29	109.73	96.59	0.87	0.00	0.00
GALNT1	2589	2.25	12.47	3.55	5.22	0.74	0.00	0.00
GALNT16	57452	4.43	4.11	1.63	1.1	1.64	0.00	0.00
GAMT	2593	451.8	158.44	187.8	167	0.79	0.00	0.00

GAN	8139	0.15	1.06	0.39	0.36	0.62	0.00	0.00
GAS2L1	10634	17.41	5.51	5.52	5.99	0.98	0.00	0.00
GAS2L3	283431	0.39	2.16	0.47	0.92	0.65	0.00	0.00
GAS6	2621	3.02	1.81	1.35	0.79	1.07	0.00	0.00
GAS6-AS1	650669	0.12	0.13	0.05	0.03	1.76	0.00	0.00
GATA2-AS1	101927167	3.92	3.61	2.12	2.73	0.60	0.00	0.00
GATC	283459	16.46	11.05	8	9.79	0.62	0.00	0.00
GATD1	347862	84.66	36.23	31.74	32.07	0.60	0.00	0.00
GCC2	9648	0.24	2.26	0.43	0.53	1.00	0.00	0.00
GCHFR	2644	170.71	47.5	50.03	64.17	0.96	0.00	0.00
GCLC	2729	6.12	27.93	11	11.45	0.63	0.00	0.00
GCNT2	2651	8.44	23.38	8.6	10.07	0.74	0.00	0.00
GCSH	2653	36.03	36.66	19.8	22.15	0.80	0.00	0.00
GDAP1	54332	0.18	1.6	0.44	0.56	0.93	0.00	0.00
GDPD1	284161	1.33	2.87	1.22	0.95	0.90	0.00	0.00
GDPD3	79153	47.73	16.42	20.05	22.97	0.59	0.00	0.00
GEMIN2	8487	5.26	4.33	2.8	3.17	0.66	0.00	0.00
GEMIN4	50628	22.16	20.32	13.76	13.45	0.65	0.00	0.00
GEN1	348654	0.43	1.55	0.18	0.7	1.29	0.00	0.00
GFER	2671	52.22	16.45	19.31	21.02	0.78	0.00	0.00
GIN1	54826	0.35	0.81	0.12	0.29	1.75	0.00	0.00
GINS2	51659	63.23	49.15	29.69	33.47	0.84	0.00	0.00
GK5	256356	0.36	1.47	0.44	0.63	1.07	0.00	0.00
GLCCI1	113263	0.2	1.68	0.51	0.67	0.69	0.00	0.00
GLCE	26035	0.76	3.04	0.9	1.26	0.75	0.00	0.00
GLIS1	148979	1.85	1.68	0.45	0.34	1.95	0.00	0.00
GLMN	11146	1.45	2.47	0.65	1	1.02	0.00	0.00
GLTPD2	388323	0	0	0	0	0.89	0.00	0.00
GLYCTK	132158	68.94	18.86	19.44	26.66	0.96	0.00	0.00
GMCL1	64395	2.24	9.87	1.83	4.42	0.96	0.00	0.00
GMFB	2764	7.79	11.86	2.19	4.3	1.57	0.00	0.00
GMNN	51053	34.13	61.28	22.76	37.02	0.67	0.00	0.00
GMPPB	29925	16.93	11.2	9.16	10.84	0.61	0.00	0.00

GNA13	10672	2.08	11.79	3.15	5.06	0.74	0.00	0.00
GNAI3	2773	0.25	1.77	0.43	0.55	0.81	0.00	0.00
GNAT2	2780	5.76	12.98	4.35	5.79	0.89	0.00	0.00
GNB1L	54584	16.72	8.68	6.71	7.51	0.85	0.00	0.00
GNG10	2790	8.49	13.88	6.01	2.29	1.43	0.00	0.00
GNL2	29889	4.3	14.05	3.64	4.99	0.99	0.00	0.00
GNL3	26354	0.21	0.27	0.06	0.11	0.94	0.00	0.00
GNMT	27232	7.2	1.82	0.87	4.1	0.87	0.00	0.00
GNPDA2	132789	1.68	1.9	0.72	1.85	0.73	0.00	0.00
GNPTAB	79158	0.58	4.98	1.48	1.81	0.84	0.00	0.00
GOLGA5	9950	3.46	12.94	3.82	6.01	0.73	0.00	0.00
GOLGA6L9	440295	0.06	0.39	0.08	0.05	1.50	0.00	0.00
GOLGA8A	23015	0.11	1.92	0.41	0.45	1.45	0.00	0.00
GOLGA8B	440270	0.26	2.8	1	0.73	0.89	0.00	0.00
GOLIM4	27333	1.26	8.98	2.33	4.13	0.62	0.00	0.00
GOPC	57120	1.56	8.86	2.13	3.16	0.93	0.00	0.00
GORAB	92344	0.14	1.03	0.18	0.29	1.45	0.00	0.00
GP1BB	2812	3.29	1.05	0.65	0.95	1.45	0.00	0.00
GPALPP1	55425	0	0	0.1	0	0.81	0.00	0.00
GPAM	57678	5.77	31.63	7.66	9.74	0.99	0.00	0.00
GPATCH11	253635	0.52	2.45	0.6	0.87	1.03	0.00	0.00
GPATCH2	55105	0.4	1.67	0.54	0.57	0.60	0.00	0.00
GPATCH2L	55668	0.6	1.9	1.07	0.99	0.75	0.00	0.00
GPATCH4	54865	6.36	13.96	4.12	5.64	1.00	0.00	0.00
GPBP1	65056	1.99	8.45	2.03	2.65	1.15	0.00	0.00
GPC6	10082	2.73	14.16	3.89	3.87	1.09	0.00	0.00
GPCPD1	56261	0.64	3.54	1.02	1.04	0.73	0.00	0.00
GPHA2	170589	70.8	13.09	13.58	19.28	1.37	0.00	0.00
GPN3	51184	4.18	10.6	4.59	4.7	0.63	0.00	0.00
GPR137	56834	29.33	12.26	11.9	14.46	0.63	0.00	0.00
GPSM2	29899	0.62	4.52	1.31	1.58	0.81	0.00	0.00
GPX4	2879	1411.67	458.82	642.37	539.72	0.68	0.00	0.00
GPX8	493869	0.23	1.66	0.03	0.05	3.74	0.00	0.00

GRK3	157	1.27	3.05	1.38	1.21	0.65	0.00	0.00
GRPEL2	134266	0.62	2.48	0.94	0.96	0.70	0.00	0.00
GRWD1	83743	53.92	23.21	21.16	23.57	0.72	0.00	0.00
GSKIP	51527	4.38	5.19	1.81	2.71	1.07	0.00	0.00
GSTA1	2938	94.28	25.6	7.88	18	2.25	0.00	0.00
GSTA2	2939	12.79	3.99	1.42	4.98	1.41	0.00	0.00
GSTCD	79807	1.27	4.7	1.35	1.24	1.08	0.00	0.00
GSTM2	2946	4.05	3.13	2.22	1.17	1.09	0.00	0.00
GTF2A1	2957	0.54	3.13	0.77	1.57	0.65	0.00	0.00
GTF2F2	2963	6.54	14.55	5.4	8.45	0.64	0.00	0.00
GTF2H3	2967	4.35	11.38	5.06	4.93	0.58	0.00	0.00
GTF3C3	9330	2.13	11.95	5.01	4.26	0.58	0.00	0.00
GTPBP10	85865	3.46	6.95	2.59	3.05	1.19	0.00	0.00
GTPBP4	23560	15.93	43.65	15.51	14.28	0.96	0.00	0.00
GTPBP6	8225	84.66	26.33	35.08	33.82	0.67	0.00	0.00
GUSBP1	728411	0.74	1.23	0.59	0.72	0.78	0.00	0.00
GXYLT1	283464	1.36	2.49	1.05	1.09	0.78	0.00	0.00
GYS1	2997	30.13	25.57	17.73	19.45	0.59	0.00	0.00
H2AFX	3014	240.32	101.39	85.64	92.43	0.95	0.00	0.00
HACE1	57531	0.18	2.05	0.54	0.69	0.90	0.00	0.00
HASPIN	83903	1.51	4.63	1.71	2.03	0.71	0.00	0.00
HAT1	8520	31.1	36.77	5.27	11.67	1.82	0.00	0.00
HAUS1	115106	11.42	18.19	5.45	9.27	1.00	0.00	0.00
HAUS3	79441	0.1	0.04	0.13	0	1.60	0.00	0.00
HAUS6	54801	0.05	0	0	0	1.16	0.00	0.00
HBA2	3040	22.13	8	6.9	4.81	1.01	0.00	0.00
HBQ1	3049	41.52	15.62	19.81	12.69	0.83	0.00	0.00
HBS1L	10767	7.52	27.66	6.63	9.72	1.07	0.00	0.00
HDAC2	3066	23.31	65.06	18.27	22.05	1.11	0.00	0.00
HDAC2-AS2	101927768	0.46	0.92	0.1	0.42	1.49	0.00	0.00
HEATR1	55127	3.04	16.96	7.74	7.86	0.61	0.00	0.00
HEATR5A	25938	0.57	2.58	0.85	1.12	0.63	0.00	0.00
HECTD1	25831	1.14	8.19	2.02	3.19	0.91	0.00	0.00

HECTD2	143279	0.06	0.69	0	0.11	1.08	0.00	0.00
HELLS	3070	1.18	5.64	0.97	1.8	1.05	0.00	0.00
HELZ	9931	0.82	3.52	0.89	1.72	0.58	0.00	0.00
HEPACAM	220296	19.26	19.7	10.79	11.91	0.74	0.00	0.00
HERC1	8925	0.24	2.13	0.7	0.84	0.59	0.00	0.00
HERC2P2	400322	0.09	1.02	0.25	0.37	0.83	0.00	0.00
HERC2P3	283755	0.11	0.32	0.09	0.15	1.21	0.00	0.00
HERC4	26091	0	0	0	0	0.78	0.00	0.00
HIBCH	26275	4.89	15.6	3.94	6.78	1.03	0.00	0.00
HIC1	3090	0.72	0.22	0.15	0.11	1.94	0.00	0.00
HIF1A	3091	4.65	21.64	5.41	9.86	0.75	0.00	0.00
HINT3	135114	1.89	4.55	1.54	2.37	0.73	0.00	0.00
HIPK2	28996	0.5	3.52	0.98	1.17	0.74	0.00	0.00
HIPK3	10114	0.66	3.58	0.89	1.47	0.85	0.00	0.00
HIST1H2BC	8347	1.44	1.54	0.37	0.98	2.50	0.00	0.00
HIST1H2BK	85236	13.51	7	6.63	5.77	0.90	0.00	0.00
HIVEP2	3097	0.1	1.77	0.45	0.43	1.09	0.00	0.00
HJURP	55355	10.04	22.84	10.08	9.92	0.74	0.00	0.00
HLTF-AS1	100873945	0.43	0	0	0	6.45	0.00	0.00
HLX-AS1	100873924	0.3	0.2	0.02	0.02	3.35	0.00	0.00
HMGB1	3146	2.99	4.6	1.55	1.82	1.14	0.00	0.00
HMGB2	3148	37.85	74.14	33.78	30.79	0.79	0.00	0.00
HMGN2P46	283651	0	0.24	0.01	0	5.08	0.00	0.00
HMGN3	9324	63.18	76.13	34.99	55.35	0.60	0.00	0.00
HMMR	3161	3.08	6.96	0.97	2.5	1.61	0.00	0.00
HNRNPA2B1	3181	75.65	253.94	77.77	118.52	0.70	0.00	0.00
HNRNPA3	220988	27.35	98.73	34.23	43.41	0.71	0.00	0.00
HNRNPDL	9987	16.23	42.44	10.28	14.43	1.24	0.00	0.00
HNRNPH2	3188	2.02	15.12	4.28	5.48	0.80	0.00	0.00
HNRNPR	10236	30.85	83.74	24.53	36.21	0.92	0.00	0.00
HNRNPU	3192	23.93	78.81	29.18	35.97	0.61	0.00	0.00
HOOK1	51361	0.47	2.37	0.69	1.19	0.60	0.00	0.00
HOOK3	84376	0.3	2.19	0.63	0.85	0.76	0.00	0.00

HOXA10	3206	0	0	0	0	2.76	0.00	0.00
HPF1	54969	2.99	8.38	3.58	3.37	0.70	0.00	0.00
HPX	3263	16.2	4.13	4.59	6.71	0.86	0.00	0.00
HS2ST1	9653	5.75	22.94	7.11	9.85	0.67	0.00	0.00
HS3ST4	9951	4.79	3.53	1.99	2.66	0.83	0.00	0.00
HSDL2	84263	6.28	23.39	5.72	10.77	0.75	0.00	0.00
HSF2BP	11077	1.24	1.38	1.04	0.94	0.68	0.00	0.00
HSP90AA1	3320	59.99	273.94	40.06	61.42	1.70	0.00	0.00
HSP90B1	7184	66.36	244.79	78.06	98.58	0.95	0.00	0.00
HSPA13	6782	3.14	10.86	3.24	3.45	1.05	0.00	0.00
HSPA14	51182	13.89	18.75	6.7	8.75	0.86	0.00	0.00
HSPA4	3308	13.79	45.72	13.7	19.25	0.69	0.00	0.00
HSPA4L	22824	1.31	6.06	0.99	1.64	1.36	0.00	0.00
HSPA8	3312	450.4	505.6	256.18	359.05	0.67	0.00	0.00
HSPBP1	23640	72.29	28.26	32.03	34.1	0.61	0.00	0.00
HSPD1	3329	155.96	512.5	169.87	236.93	0.71	0.00	0.00
HSPE1	3336	356.48	329.82	203.94	250.38	0.60	0.00	0.00
HSPH1	10808	4.51	26.79	3.03	6.32	1.70	0.00	0.00
HTATSF1	27336	0	0	0	0	0.87	0.00	0.00
HTT-AS	100750326	3.18	4.05	0.28	1.48	2.03	0.00	0.00
HYLS1	219844	1.65	1.72	0.5	1.52	0.92	0.00	0.00
IBTK	25998	1.36	6.07	1.52	2.64	0.80	0.00	0.00
ICAM3	3385	36.87	12.56	12.37	16.33	0.71	0.00	0.00
ICE1	23379	0.6	3.54	0.62	1.1	1.23	0.00	0.00
ICE2	79664	1.15	4.65	1.37	2.46	0.60	0.00	0.00
ICK	22858	1.61	3.74	1.61	2.05	0.61	0.00	0.00
ICOSLG	23308	0.86	0.49	0	0.12	2.13	0.00	0.00
IDH3A	3419	6.47	15.01	5.85	6.82	0.74	0.00	0.00
IER3IP1	51124	15.56	26.3	11.02	14.74	0.69	0.00	0.00
IFIT5	24138	0.09	0.39	0.04	0.06	2.19	0.00	0.00
IFT20	90410	5.97	8.77	5.57	5.03	0.72	0.00	0.00
IFT22	64792	7.63	5.81	4.7	4.97	0.69	0.00	0.00
IFT57	55081	3.03	5.37	0.84	0.85	2.23	0.00	0.00

IFT74	80173	0.75	2.56	0.5	0.44	1.58	0.00	0.00
IFT81	28981	0.21	0.3	0.44	0.14	1.53	0.00	0.00
IFT88	8100	0.72	2.23	0.53	0.9	0.98	0.00	0.00
IKBIP	121457	7.14	11.29	3.1	5.71	1.00	0.00	0.00
IL12RB2	3595	15.1	49.06	20.73	20.64	0.64	0.00	0.00
IL17RC	84818	27.63	8.58	11.2	12.32	0.62	0.00	0.00
IL1RAP	3556	2.02	5	1.37	2.36	0.87	0.00	0.00
IL6ST	3572	1.92	6.21	1.75	3.34	0.64	0.00	0.00
ILVBL	10994	94.54	36.16	37.93	32.55	0.91	0.00	0.00
IMPA1	3612	2.12	5.34	2.08	2.58	0.74	0.00	0.00
IMPACT	55364	0.53	1.58	0.5	0.48	1.12	0.00	0.00
ING2	3622	3.66	5.27	2.49	2.73	0.82	0.00	0.00
INPP5F	22876	1.22	3.94	1.08	2.3	0.67	0.00	0.00
INPP5K	51763	11.76	6.12	6.33	5.77	0.59	0.00	0.00
INSIG2	51141	3.37	13.26	6.06	5.55	0.61	0.00	0.00
INTS13	55726	3.63	11.59	2.06	5.3	1.08	0.00	0.00
INTS2	57508	0.49	2.99	0.49	1.05	1.15	0.00	0.00
INTS6	26512	1.67	5.38	1.87	2.73	0.62	0.00	0.00
INTS8	55656	0.7	2.87	0.62	0.92	1.26	0.00	0.00
INTU	27152	0.25	0.85	0.14	0.36	0.84	0.00	0.00
IP6K1	9807	22.63	13.1	11.56	11.2	0.64	0.00	0.00
IPMK	253430	0.34	1.05	0.3	0.43	0.89	0.00	0.00
IPO11	51194	2.28	7.65	1.84	2.31	1.28	0.00	0.00
IPO7	10527	4.46	27.83	7.39	11.22	0.77	0.00	0.00
IQCG	84223	2.27	3.3	1.96	1.3	0.62	0.00	0.00
IQGAP2	10788	4.37	21.1	4.82	12.64	0.69	0.00	0.00
IRAK1BP1	134728	0.71	1.63	0.94	0.46	1.28	0.00	0.00
IREB2	3658	1.07	7.21	1.72	2.68	0.88	0.00	0.00
ISM1	140862	2.77	6.09	1.54	2.27	1.20	0.00	0.00
ISX	91464	4.35	3.54	2.42	2.81	0.62	0.00	0.00
ITGA2	3673	0.79	7.5	2.3	2.43	0.80	0.00	0.00
ITGA6-AS1	101929947	0.49	1.08	0.07	0.15	3.36	0.00	0.00
ITGAV	3685	1.23	10.62	2.54	3.09	1.05	0.00	0.00

ITGB3BP	23421	14.4	13.17	3.88	4.02	1.65	0.00	0.00
ITPR2	3709	0.64	5.71	1.14	2.04	1.12	0.00	0.00
ITPRID2	6744	3.73	9.21	3.05	4.86	0.77	0.00	0.00
ITSN2	50618	0	0	0	0	0.62	0.00	0.00
IWS1	55677	6.22	21.03	7.01	9.36	0.62	0.00	0.00
JMJD1C	221037	0.28	1.47	0.27	0.65	1.01	0.00	0.00
JMJD4	65094	43.59	13.37	16.97	13.84	0.89	0.00	0.00
JOSD2	126119	10.23	3.88	4.79	1.73	1.16	0.00	0.00
JRK	8629	1.57	2.17	1.45	1.07	0.62	0.00	0.00
KAT6B	23522	0.19	1.52	0.39	0.7	0.69	0.00	0.00
KATNA1	11104	2.37	10.1	3.43	4.32	0.64	0.00	0.00
KATNAL1	84056	0.37	1.07	0.62	0.62	0.58	0.00	0.00
KATNBL1	79768	2.68	3.07	1.63	1.67	0.75	0.00	0.00
KBTBD6	89890	0.53	2.09	0.86	0.87	0.59	0.00	0.00
KBTBD8	84541	0.18	0.66	0.08	0.13	2.00	0.00	0.00
KCNG3	170850	0	0.04	0	0	1.20	0.00	0.00
KCTD3	51133	2.5	14.3	3.35	5.53	0.91	0.00	0.00
KCTD9	54793	1.51	8.29	2.35	3.2	0.84	0.00	0.00
KDELC2	143888	2.26	12.05	3.04	4.08	1.00	0.00	0.00
KDM3A	55818	1.87	11.19	3.3	3.4	1.02	0.00	0.00
KDM5A	5927	0.44	3.84	1.51	1.48	0.65	0.00	0.00
KIAA0232	9778	0.49	4.17	1.23	1.49	0.69	0.00	0.00
KIAA0513	9764	118.03	47.71	71.96	64.87	1.07	0.00	0.00
KIAA0753	9851	1.2	4.55	1.69	2.04	0.96	0.00	0.00
KIAA0895	23366	0.28	1.91	0.4	0.43	1.13	0.00	0.00
KIAA0895L	653319	1.65	3.2	1.39	1.63	0.68	0.00	0.00
KIAA1109	84162	0.08	0.82	0.14	0.27	1.19	0.00	0.00
KIAA1143	57456	5.22	7.01	1.87	2.65	1.10	0.00	0.00
KIAA1324L	222223	0.52	1.26	0.55	0.49	0.76	0.00	0.00
KIAA1551	55196	0.13	0.47	0.07	0.19	1.10	0.00	0.00
KIAA1586	57691	3.07	2.64	0.46	1.51	1.54	0.00	0.00
KIAA1841	84542	78.61	55.42	55.43	46.34	0.66	0.00	0.00
KIDINS220	57498	0.88	4.03	1.1	1.59	0.84	0.00	0.00

KIF11	3832	1.48	7.78	0.97	1.98	1.64	0.00	0.00
KIF14	9928	0.13	0.72	0.08	0.15	1.81	0.00	0.00
KIF15	56992	0.54	3.22	0.25	0.73	2.06	0.00	0.00
KIF16B	55614	1.09	5.51	1.05	1.97	1.13	0.00	0.00
KIF18A	81930	0.52	1.06	0.14	0.59	1.32	0.00	0.00
KIF20B	9585	42.22	34.02	34.94	40.67	0.63	0.00	0.00
KIF21A	55605	0.75	4.42	0.98	1.41	1.11	0.00	0.00
KIF22	3835	87.33	55.43	49.02	45.25	0.61	0.00	0.00
KIF23	9493	2.85	9.85	1.82	3.74	1.27	0.00	0.00
KIF2A	3796	0.93	6.95	1.52	2.65	1.00	0.00	0.00
KIF4A	24137	3.84	12.42	4	5.05	0.82	0.00	0.00
KIF5B	3799	2.9	17.88	2.48	5.03	1.41	0.00	0.00
KIFC3	3801	37.65	28.1	21.91	17.77	0.70	0.00	0.00
KIN	22944	1.99	4.9	1.32	2.79	0.63	0.00	0.00
KISS1R	84634	12.08	6.28	4.75	4.09	0.93	0.00	0.00
KITLG	4254	0.35	2.2	0.46	0.73	1.11	0.00	0.00
KIZ	55857	8.61	49.1	9.43	8.2	1.64	0.00	0.00
KLF12	11278	0.68	1.76	0.71	0.67	0.68	0.00	0.00
KLHL15	80311	0.3	1.12	0.24	0.37	1.05	0.00	0.00
KLHL20	27252	0.71	1.74	0.62	0.75	0.70	0.00	0.00
KLHL8	57563	0.51	2.7	0.58	0.79	1.25	0.00	0.00
KLHL9	55958	1.01	2.61	0.86	1.18	0.83	0.00	0.00
KLRA1P	10748	0.05	0.93	0.09	0.11	1.26	0.00	0.00
KMT2E	55904	0.6	3.37	0.9	1.4	0.68	0.00	0.00
KNL1	57082	0.42	1.38	0.35	0.52	1.02	0.00	0.00
KNTC1	9735	0.46	4.55	0.88	0.94	1.49	0.00	0.00
KPNA3	3839	4.23	21	6.72	8.85	0.66	0.00	0.00
KPNB1	3837	20.18	79.74	24.78	32.45	0.81	0.00	0.00
KRAS	3845	0.17	2.39	0.61	0.53	1.14	0.00	0.00
KRCC1	51315	1.24	2.66	0.62	0.97	1.27	0.00	0.00
KRIT1	889	0.4	2.29	0.53	0.93	0.88	0.00	0.00
KRR1	11103	2.72	4.68	0.98	1.7	1.19	0.00	0.00
KTN1	3895	2.22	8.67	2.2	4.28	0.77	0.00	0.00

L2HGDH	79944	0.34	2.03	0.56	0.79	0.68	0.00	0.00
LACTB2	51110	4.62	9.76	3.41	5.31	0.70	0.00	0.00
LAMB1	3912	9.52	54.02	22.48	19.26	0.65	0.00	0.00
LAMC1-AS1	110841583	0	1.08	0	0.58	0.88	0.00	0.00
LAMTOR1	55004	182.41	63.62	71.17	84.03	0.68	0.00	0.00
LAMTOR3	8649	3.95	6.96	2.56	4.18	0.74	0.00	0.00
LAMTOR4	389541	93.08	39.52	43.52	39.25	0.67	0.00	0.00
LANCL1	10314	2.57	12.59	3.61	5.87	0.67	0.00	0.00
LARP4	113251	2.23	6.79	1.79	3.13	0.75	0.00	0.00
LARP7	51574	5.04	5.82	1.08	2.91	1.40	0.00	0.00
LBR	3930	6.16	19.51	6.97	8.47	0.73	0.00	0.00
LCA5	167691	0.04	0.37	0.08	0.09	1.79	0.00	0.00
LEO1	123169	4.73	15.51	5.03	7.31	0.70	0.00	0.00
LEPROT	54741	2.15	5.08	1.88	2	0.84	0.00	0.00
LGALS2	3957	61.25	15.62	16.84	22.35	1.00	0.00	0.00
LGI1	9211	10.71	20.72	5.35	4.23	1.85	0.00	0.00
LGR4	55366	0.2	0.14	0.16	0.32	0.73	0.00	0.00
LHFPL5	222662	0	0.3	0.06	0.03	1.69	0.00	0.00
LHX1	3975	0.4	0.72	0.02	0.08	3.54	0.00	0.00
LIFR	3977	0.16	0.66	0.17	0.3	0.70	0.00	0.00
LIME1	54923	97.45	25.37	27.54	36.19	0.92	0.00	0.00
LIMK1	3984	47.89	25.75	26.99	21.32	0.60	0.00	0.00
LIMS1	3987	3.95	13.64	4.85	6.51	0.64	0.00	0.00
LIN28B	389421	4.72	21.23	2.27	5.13	1.80	0.00	0.00
LIN54	132660	0.88	3.47	0.8	1.99	0.66	0.00	0.00
LIN7A	8825	2.68	4.26	1	2.62	0.64	0.00	0.00
LIN7C	55327	0.55	2.56	0.6	0.96	0.97	0.00	0.00
LIN9	286826	0.85	1.72	0.54	0.8	1.12	0.00	0.00
LINC00272	388719	0.36	0.28	0.11	0	2.55	0.00	0.00
LINC00426	100188949	30.67	62	23.81	26.35	0.88	0.00	0.00
LINC00641	283624	0.04	0.66	0.12	0.29	0.77	0.00	0.00
LINC00862	554279	0.29	1.07	0.04	0.15	1.67	0.00	0.00
LINC00888	100505687	3.16	7.52	2.75	4.06	0.67	0.00	0.00

LINC01336	104326191	0.03	0.89	0	0.1	4.06	0.00	0.00
LINC01814	101929567	0.04	0.23	0.1	0.06	0.61	0.00	0.00
LINC02008	105377180	0.15	0.72	0.14	0.31	1.06	0.00	0.00
LINC02313	105370439	0	0	0	0	0.99	0.00	0.00
LINC02561	110806285	0.02	0.05	0	0	4.07	0.00	0.00
LLPH	84298	7.7	11.61	3.85	5.46	1.12	0.00	0.00
LMAN1	3998	6.76	44.93	10.57	15.86	0.95	0.00	0.00
LMBRD2	92255	0.31	0.91	0.18	0.36	1.21	0.00	0.00
LMCD1	29995	0.04	0	0	0.14	0.72	0.00	0.00
LMLN	89782	0.15	0.48	0.1	0.18	1.34	0.00	0.00
LNCOC1	100288181	0.06	0.04	0	0	5.36	0.00	0.00
LNPK	80856	1.16	4.56	1.02	1.2	1.27	0.00	0.00
LOC100130691	100130691	0.18	1.26	0.24	0.26	0.81	0.00	0.00
LOC100287896	100287896	1.13	0.72	0.09	0.32	2.25	0.00	0.00
LOC100507600	100507600	4.28	12.55	4.35	5.04	0.83	0.00	0.00
LOC100996447	100996447	0	0.3	0	0	4.66	0.00	0.00
LOC101927013	101927013	0.42	1.26	0.17	0.4	1.54	0.00	0.00
LOC101927039	101927039	105.26	78.83	51.82	53.55	0.81	0.00	0.00
LOC101927283	101927283	1.92	3.31	0.87	1.47	1.15	0.00	0.00
LOC101927345	101927345	0.69	3.3	0.41	0.07	2.45	0.00	0.00
LOC101927482	101927482	1.79	2.12	0.09	0.26	3.51	0.00	0.00
LOC101927497	101927497	0.91	3.91	1.65	0.42	1.21	0.00	0.00
LOC101927978	101927978	0.32	1.92	1.01	0.68	1.01	0.00	0.00
LOC101928351	101928351	0.01	0	0	0	6.00	0.00	0.00
LOC101928731	101928731	0.51	0	0	0	4.87	0.00	0.00
LOC101928953	101928953	1.86	0.63	0.64	0.69	0.92	0.00	0.00
LOC101929204	101929204	0	0.16	0	0	5.64	0.00	0.00
LOC101929231	101929231	0.28	0.58	0.09	0.12	1.99	0.00	0.00
LOC101929356	101929356	0.45	2.57	0.71	1.18	0.90	0.00	0.00
LOC102723468	102723468	0	1.23	0	0	7.24	0.00	0.00
LOC102723757	102723757	4.14	36.93	4.65	10.4	1.43	0.00	0.00
LOC102723824	102723824	0.03	0.35	0.06	0.07	1.60	0.00	0.00
LOC102724322	102724322	0.15	0.32	0.15	0.16	1.12	0.00	0.00

LOC102724438	102724438	7.92	2.59	1.25	2.8	1.37	0.00	0.00
LOC102724528	102724528	0.16	0.17	0	0	5.44	0.00	0.00
LOC102724701	102724701	0.34	1.2	0.49	0.58	0.60	0.00	0.00
LOC102724843	102724843	1.05	3.61	1.09	1.39	0.91	0.00	0.00
LOC102724951	102724951	0.24	0.91	0.19	0.21	0.83	0.00	0.00
LOC105369225	105369225	0.51	3.91	0.69	0.9	1.02	0.00	0.00
LOC105369420	105369420	0.69	1.34	0.13	0.08	3.26	0.00	0.00
LOC105369519	105369519	0.16	0.18	0.04	0.04	2.02	0.00	0.00
LOC105369748	105369748	0.37	0.95	0.15	0.38	1.32	0.00	0.00
LOC105369844	105369844	0.01	0.16	0	0	4.81	0.00	0.00
LOC105370560	105370560	0.4	0.58	0.13	0.27	1.27	0.00	0.00
LOC105370687	105370687	2.04	2.36	1.13	0.49	1.51	0.00	0.00
LOC105370775	105370775	0.64	2.3	0.38	0.67	1.74	0.00	0.00
LOC105370784	105370784	0	0	0	0	3.13	0.00	0.00
LOC105370947	105370947	0.8	0.94	0.03	0.14	2.53	0.00	0.00
LOC105371425	105371425	0.79	1.19	0.53	0.43	1.04	0.00	0.00
LOC105371505	105371505	0	3.3	0	0	8.61	0.00	0.00
LOC105371755	105371755	1.04	2.37	1.01	1.13	0.70	0.00	0.00
LOC105371762	105371762	1.27	0.22	0	0.15	3.32	0.00	0.00
LOC105372279	105372279	2909.94	902.11	960.09	1304.72	5.12	0.00	0.00
LOC105372589	105372589	53.33	44.04	30.53	32.88	0.62	0.00	0.00
LOC105372653	105372653	3.92	2.02	0.12	0.76	2.98	0.00	0.00
LOC105373047	105373047	0.7	1.14	0.29	0	2.69	0.00	0.00
LOC105373184	105373184	0.12	1.11	0.19	0.44	0.92	0.00	0.00
LOC105373255	105373255	0.12	1.15	0.17	0.17	1.25	0.00	0.00
LOC105373283	105373283	0	0.13	0	0	5.97	0.00	0.00
LOC105373786	105373786	1.62	1.4	0.37	0.67	1.53	0.00	0.00
LOC105374640	105374640	0.54	9.82	1.05	1.14	2.23	0.00	0.00
LOC105375022	105375022	0.47	0.73	0.35	0.12	1.36	0.00	0.00
LOC105375116	105375116	0.65	0	0	0	6.85	0.00	0.00
LOC105375228	105375228	0.2	1.15	0.29	0.19	1.28	0.00	0.00
LOC105375799	105375799	1.32	7.41	3.08	3.69	0.93	0.00	0.00
LOC105376216	105376216	0.04	0.08	0	0.03	2.20	0.00	0.00

LOC105376217	105376217	0.16	0.61	0	0.2	1.94	0.00	0.00
LOC105376392	105376392	7.09	2.14	0	2.6	1.84	0.00	0.00
LOC105376644	105376644	0.58	1.18	0.14	0.48	1.49	0.00	0.00
LOC105377015	105377015	0.13	0	0	0	4.57	0.00	0.00
LOC105377063	105377063	0.13	0	0	0	5.53	0.00	0.00
LOC105377517	105377517	0	1.25	0	0	8.12	0.00	0.00
LOC105378083	105378083	0.11	0.1	0.07	0	2.59	0.00	0.00
LOC105378511	105378511	0.04	0.01	0	0	1.18	0.00	0.00
LOC105379154	105379154	1.55	6.41	1.87	2.48	0.86	0.00	0.00
LOC105379525	105379525	0.01	0.04	0	0	3.47	0.00	0.00
LOC105379599	105379599	3.02	4.96	2.54	2.29	0.66	0.00	0.00
LOC107984034	107984034	7.15	6.83	2.87	3.4	1.17	0.00	0.00
LOC107984109	107984109	0.39	0.47	0	0.39	1.14	0.00	0.00
LOC107984396	107984396	4.08	3.17	2.33	2.2	0.79	0.00	0.00
LOC107984546	107984546	0	0.26	0	0	5.65	0.00	0.00
LOC107984663	107984663	0.17	2.15	0.57	0.45	1.17	0.00	0.00
LOC107985027	107985027	0.33	1.14	0.3	0.37	0.81	0.00	0.00
LOC107985381	107985381	0.61	4.58	0.19	0.51	3.61	0.00	0.00
LOC107985410	107985410	0	0.29	0.01	0	4.72	0.00	0.00
LOC107985414	107985414	0.28	0.19	0.11	0	2.16	0.00	0.00
LOC107985506	107985506	0.01	0.22	0.07	0.02	1.21	0.00	0.00
LOC107986282	107986282	0	0.16	0	0.03	2.44	0.00	0.00
LOC107986303	107986303	0	0.15	0	0	4.60	0.00	0.00
LOC107986430	107986430	1	1.18	0.68	0.46	0.96	0.00	0.00
LOC107986837	107986837	1.19	1.24	0	0	6.46	0.00	0.00
LOC107986852	107986852	0.78	0.34	0.35	0.08	1.42	0.00	0.00
LOC107987043	107987043	0.18	4.16	0.2	1.63	1.21	0.00	0.00
LOC107987225	107987225	0.16	0.02	0.01	0.01	3.45	0.00	0.00
LOC107987238	107987238	3.75	1.25	0.84	1.34	1.15	0.00	0.00
LOC107987397	107987397	0.16	0.62	0.06	0.13	2.03	0.00	0.00
LOC107987408	107987408	0.23	0.08	0.02	0	3.30	0.00	0.00
LOC107987420	107987420	0.03	0.18	0	0	6.30	0.00	0.00
LOC112267869	112267869	0.2	0.16	0.09	0.02	1.61	0.00	0.00

LOC112267885	112267885	0.13	0.94	0.25	0.03	1.91	0.00	0.00
LOC112267993	112267993	2.53	0.44	0	0.44	2.76	0.00	0.00
LOC112268149	112268149	0.07	0.1	0	0	5.34	0.00	0.00
LOC112268150	112268150	0	0.57	0	0.17	1.69	0.00	0.00
LOC112268153	112268153	0.02	0.06	0.01	0.02	1.41	0.00	0.00
LOC112268246	112268246	0	0	0	0.02	0.63	0.00	0.00
LOC112268259	112268259	0.14	0.74	0.22	0.22	0.97	0.00	0.00
LOC112268321	112268321	13.85	1.9	2.29	1.52	1.26	0.00	0.00
LOC112268322	112268322	0.05	0.26	0.03	0.01	2.25	0.00	0.00
LOC112268430	112268430	0.09	1.1	0.28	0.33	0.94	0.00	0.00
LOC389831	389831	4.2	7.19	3.07	4.75	0.66	0.00	0.00
LOC400710	400710	4.96	1.9	0.77	0.63	2.31	0.00	0.00
LOC440300	440300	0.55	0.12	0.11	0.09	1.54	0.00	0.00
LPAR6	10161	2.94	3.05	0.84	1.24	1.55	0.00	0.00
LPGAT1	9926	0.3	2.81	0.84	0.43	1.41	0.00	0.00
LRBA	987	0.95	7.27	2.8	4.37	0.61	0.00	0.00
LRFN4	78999	16.64	4.62	6.37	5.09	0.91	0.00	0.00
LRFN5	145581	0.02	0	0	0	2.28	0.00	0.00
LRIF1	55791	2.64	6	1.64	2.53	1.06	0.00	0.00
LRP3	4037	122.13	30.14	36.48	35.76	1.09	0.00	0.00
LRP6	4040	0.52	2.71	0.71	1.18	0.73	0.00	0.00
LRPPRC	10128	12.26	70.47	16.6	26.19	0.88	0.00	0.00
LRRC14	9684	8.46	9.54	6.81	7.76	0.85	0.00	0.00
LRRC20	55222	76.69	33.24	28.65	39.46	0.70	0.00	0.00
LRRC37A2	474170	0.05	1.83	0.2	0.27	2.07	0.00	0.00
LRRC40	55631	2.65	4.97	0.62	2.21	1.44	0.00	0.00
LRRC58	116064	0.9	4.11	0.95	1.75	0.86	0.00	0.00
LSM10	84967	73.05	30.6	30.74	35.92	0.63	0.00	0.00
LSM5	23658	26.36	20.24	15.54	16.43	0.61	0.00	0.00
LTN1	26046	9.89	10.93	7.95	6.82	1.86	0.00	0.00
LTV1	84946	0	0	0	0	1.10	0.00	0.00
LUC7L3	51747	2.7	17.78	4.22	5.53	1.00	0.00	0.00
LUZP1	7798	3.01	12.36	4.92	4.88	0.62	0.00	0.00

LY6E	4061	72.21	38.28	46.95	21.44	0.70	0.00	0.00
LYAR	55646	9.15	20.98	7.91	10.56	0.70	0.00	0.00
LYRM2	57226	1.78	2.6	1.2	0.31	1.47	0.00	0.00
LYRM7	90624	0.4	1.76	0.58	0.98	0.62	0.00	0.00
LYSMD3	116068	0.55	1.93	0.33	0.75	1.19	0.00	0.00
LZTFL1	54585	1.26	1.9	0.83	0.63	1.08	0.00	0.00
MACF1	23499	0.83	7.59	2.31	2.99	0.65	0.00	0.00
MAD2L1	4085	36.5	49.23	23.1	28.01	0.77	0.00	0.00
MAF1	84232	150.24	53.5	68.72	66.04	0.61	0.00	0.00
MAGI2	9863	1.23	5.44	1.8	2.39	0.65	0.00	0.00
MAGI3	260425	0.39	1.66	0.48	0.55	0.60	0.00	0.00
MAGOHB	55110	2.56	3.51	1.84	1.96	0.78	0.00	0.00
MALRD1	340895	0	0	0.03	0.03	1.61	0.00	0.00
MALT1	10892	2.49	17.55	3.97	4.7	1.20	0.00	0.00
MAML2	84441	0.5	4.79	1.57	1.59	0.67	0.00	0.00
MAN1A1	4121	22.22	108.64	41.34	46.41	0.59	0.00	0.00
MAN1A2	10905	0.59	3.06	0.39	1.17	1.22	0.00	0.00
MAN2A1	4124	1.24	9.78	2.29	3.31	0.97	0.00	0.00
MANEA	79694	0.81	1.28	0.36	0.97	0.69	0.00	0.00
MAP1A	4130	27.21	53.82	31.34	24.77	3.76	0.00	0.00
MAP2K4	6416	3.63	11.55	3.67	4.95	0.84	0.00	0.00
MAP2K6	5608	0.26	0.84	0.25	0.12	1.63	0.00	0.00
MAP3K11	4296	125.09	36.12	46.33	54.82	0.73	0.00	0.00
MAP3K2	10746	0.03	0.98	0.08	0.02	3.35	0.00	0.00
MAP3K20	51776	0	0.01	0	0	0.78	0.00	0.00
MAP3K4	4216	0.94	6.54	1.76	1.98	0.96	0.00	0.00
MAP3K8	1326	0.01	0.07	0.12	0.08	0.84	0.00	0.00
MAP4K3	8491	0	0	0	0	0.80	0.00	0.00
MAP4K4	9448	25.21	110.6	48.35	41.19	0.59	0.00	0.00
MAP4K5	11183	0.7	6.08	1.19	2.19	1.00	0.00	0.00
MAPK6	5597	4.99	22.5	6.35	10.67	0.69	0.00	0.00
MAPK8IP1	9479	19.02	53.94	25.99	28.85	0.83	0.00	0.00
MASTL	84930	1.47	5.95	1.44	1.85	0.98	0.00	0.00

MATR3	9782	15.31	48.39	15.09	20.63	0.72	0.00	0.00
MAZ	4150	471.08	156.05	182.53	179.1	0.78	0.00	0.00
MBD3	53615	306.11	105.13	102	105.13	0.99	0.00	0.00
MBD4	8930	2.14	1.77	2.06	2.38	1.01	0.00	0.00
MBD5	55777	0.19	0.51	0.13	0.18	0.68	0.00	0.00
MBIP	51562	7.64	8.32	4.95	7.04	0.83	0.00	0.00
MBNL1	4154	1.84	9.5	2.45	4.29	0.76	0.00	0.00
MBNL2	10150	2.44	15.07	2.89	4.61	1.05	0.00	0.00
MBNL3	55796	6.93	38.21	10.82	16.51	0.69	0.00	0.00
MBTD1	54799	0.57	3.87	1.2	1.67	0.59	0.00	0.00
MCFD2	90411	47.29	106.22	46.55	51	0.64	0.00	0.00
MCM10	55388	2.73	11.22	1.91	2.92	1.50	0.00	0.00
MCM8	84515	1.6	9.53	1.43	2.49	1.48	0.00	0.00
MCPH1	79648	2.85	8.14	2.8	3.49	0.68	0.00	0.00
MCRIP1	348262	242.28	66.06	90.28	90.42	0.78	0.00	0.00
MCRIP2	84331	1.16	0	0	0	0.75	0.00	0.00
MCRS1	10445	139.74	49.25	48.36	50.81	0.94	0.00	0.00
MDM1	56890	0.38	1.83	0.2	0.34	1.79	0.00	0.00
MDN1	23195	0.27	2.94	0.43	0.93	1.59	0.00	0.00
ME2	4200	5.24	14.53	5.68	6.33	0.70	0.00	0.00
MED13	9969	0.61	3.02	0.89	1.31	0.68	0.00	0.00
MED16	10025	52.92	20.01	23.05	21.32	0.67	0.00	0.00
MED22	6837	14.26	8.04	5.75	6.27	0.89	0.00	0.00
MED31	51003	2.07	1.75	0.32	0.74	1.85	0.00	0.00
MED7	9443	2.54	3.49	1.22	1.77	1.17	0.00	0.00
MELK	9833	5.37	18.24	5.15	6.83	0.91	0.00	0.00
MET	4233	2.35	13.04	4.56	5.88	0.62	0.00	0.00
METAP2	10988	6.29	21.85	5.08	7.6	1.11	0.00	0.00
METRN	79006	0.02	0.11	0.04	0.04	0.63	0.00	0.00
METTL14	57721	0.86	2.82	0.52	1.02	1.35	0.00	0.00
METTL15	196074	1.62	2.43	1.16	1.27	0.73	0.00	0.00
METTL5	29081	0	0	0.2	0.2	0.68	0.00	0.00
METTL8	79828	18.2	13.36	14.14	15.36	0.60	0.00	0.00

MFAP1	4236	3.96	10.95	2.73	3.13	1.34	0.00	0.00
MFN1	55669	1.33	6.12	1.09	1.91	0.99	0.00	0.00
MFSD2B	388931	1.73	0.32	0.55	1.07	0.74	0.00	0.00
MFSD8	256471	0.83	2.79	0.83	1.25	1.13	0.00	0.00
MGA	23269	0.21	2.92	0.73	0.8	1.17	0.00	0.00
MGAT4A	11320	1.16	7.03	1.53	2.39	1.04	0.00	0.00
MIA3	375056	1.12	4.99	2.1	2.69	0.75	0.00	0.00
MIB1	57534	0.75	4.05	0.77	1.31	1.20	0.00	0.00
MICU2	221154	6.39	12.72	4.04	5.52	0.98	0.00	0.00
MIER1	57708	1.65	5.95	1.77	2.56	0.76	0.00	0.00
MIER3	166968	0.46	2.07	0.45	0.78	0.94	0.00	0.00
MIGA1	374986	0.35	1.46	0.43	0.59	0.77	0.00	0.00
MIGA2	84895	17.86	5.7	4.95	7.76	0.90	0.00	0.00
MINDY2	54629	0.35	1.57	0.38	0.75	0.72	0.00	0.00
MIOS	54468	0.99	2.88	0.75	1.3	0.92	0.00	0.00
MIPOL1	145282	0.21	1.31	0.34	0.38	1.31	0.00	0.00
MIR22HG	84981	10.91	6.91	5.03	4.88	0.73	0.00	0.00
MIR4453HG	54553	0.13	1.03	0.2	0.28	1.36	0.00	0.00
MIR497HG	100506755	13.57	5.05	5.08	5.45	0.74	0.00	0.00
MIS12	79003	2.51	8.3	3.18	2.89	0.96	0.00	0.00
MIS18A	54069	5.37	10.46	4.72	5.24	0.67	0.00	0.00
MIS18BP1	55320	1.12	2.12	0.3	0.64	1.88	0.00	0.00
MITD1	129531	4.69	11.96	3.46	5.11	0.96	0.00	0.00
MKI67	4288	3.55	5.28	1.18	1.66	0.89	0.00	0.00
MKLN1	4289	2.63	7.64	2.65	4.09	0.96	0.00	0.00
MLH3	27030	0.28	1.16	0.48	0.43	0.62	0.00	0.00
MLLT10	8028	15.88	17.96	9.69	9.82	0.77	0.00	0.00
MLLT3	4300	0.46	2.26	0.42	0.63	1.49	0.00	0.00
MLST8	64223	146.26	42.99	62.55	58.35	0.68	0.00	0.00
MMADHC	27249	63.27	67.64	37.01	44.45	0.68	0.00	0.00
MMP25-AS1	100507419	0.67	0.32	0.4	0.2	0.72	0.00	0.00
MMS22L	253714	0.49	1.56	0.52	0.48	1.55	0.00	0.00
MNAT1	4331	10.54	14.72	4.77	5.24	1.32	0.00	0.00

MND1	84057	10.2	12.81	3.99	4.63	1.29	0.00	0.00
MOB4	25843	3.46	8.45	2.68	4.08	0.81	0.00	0.00
MON1A	84315	28.78	9.64	12.25	13.33	0.64	0.00	0.00
MON2	23041	0.51	3.03	0.84	1.52	0.67	0.00	0.00
MORC3	23515	0.77	2.45	0.67	0.88	1.10	0.00	0.00
MORC4	79710	2.53	11.22	3.46	4.98	0.72	0.00	0.00
MORF4L2	9643	81.94	171.93	85.54	76.29	0.66	0.00	0.00
MOSPD1	56180	4.34	10.86	4.09	5.77	0.61	0.00	0.00
MPDU1	9526	194.61	68.62	73.55	83.26	0.72	0.00	0.00
MPHOSPH10	10199	7.76	19.18	3.77	13.3	0.68	0.00	0.00
MPHOSPH6	10200	10.21	13.84	5.74	10.59	0.60	0.00	0.00
MPHOSPH8	54737	1.19	5.71	1.58	2.88	0.66	0.00	0.00
MPP5	64398	0.65	3.82	1.19	1.62	0.68	0.00	0.00
MPP6	51678	0.42	2.18	0.42	0.5	0.94	0.00	0.00
MRE11	4361	0.55	3.26	0.73	0.86	1.14	0.00	0.00
MREG	55686	2.78	3.95	4.03	4.2	0.72	0.00	0.00
MRPL1	65008	9.06	9.8	3	4.4	1.35	0.00	0.00
MRPL17	63875	53.79	35.23	29.08	29.52	0.61	0.00	0.00
MRPL19	9801	12.32	19.79	8.14	10.04	0.76	0.00	0.00
MRPL23	6150	334.82	117.93	125.27	133.54	0.73	0.00	0.00
MRPL28	10573	189.14	94.15	94.42	89.15	0.64	0.00	0.00
MRPL3	11222	53.56	71.5	35.74	39.79	0.72	0.00	0.00
MRPL35	51318	26.59	28.6	21.83	18.82	0.62	0.00	0.00
MRPL42	28977	0	0.01	0	0	0.64	0.00	0.00
MRPL50	54534	0.02	0	0	0	1.18	0.00	0.00
MRPL54	116541	114.31	41.22	43.04	44.26	0.85	0.00	0.00
MRPS12	6183	155.97	47.46	56.96	59.09	0.82	0.00	0.00
MRPS18A	55168	172.99	77.91	79.18	70.7	0.76	0.00	0.00
MRPS30-DT	100506674	0.04	0.2	0.06	0.1	0.59	0.00	0.00
MRPS31	10240	5.76	10.48	4.67	5.73	0.61	0.00	0.00
MRPS34	65993	198.35	84.43	91.4	99.1	0.58	0.00	0.00
MSANTD4	84437	0.54	2.57	0.56	0.85	1.20	0.00	0.00
MSH2	4436	3.49	15.29	2.38	4.15	1.51	0.00	0.00

MSH3	4437	0.54	3.12	0.8	1.43	0.67	0.00	0.00
MSH6	2956	5.36	25.25	7.16	9.09	0.95	0.00	0.00
MSRB1	51734	166.15	57.83	76.64	72.98	0.59	0.00	0.00
MTBP	27085	0.47	1.06	0.28	0.39	0.86	0.00	0.00
MTERF1	7978	0.47	1.47	0.28	0.41	1.22	0.00	0.00
MTERF2	80298	1.22	2.86	0.94	1.38	0.68	0.00	0.00
MTERF3	51001	0	0.05	0	0	0.70	0.00	0.00
MTF1	4520	0	0.8	0.13	0.23	1.11	0.00	0.00
MTF2	22823	1.27	6.33	0.95	1.68	1.20	0.00	0.00
MTFP1	51537	91.02	30.73	27.21	27.41	1.15	0.00	0.00
MTIF2	4528	3.71	17.06	2.93	5.24	1.30	0.00	0.00
MTIF3	219402	9.56	14.95	4.55	8.2	0.81	0.00	0.00
MTM1	4534	1.64	4.5	1.31	2.38	0.77	0.00	0.00
MTMR6	9107	1.24	3.92	0.96	1.66	1.00	0.00	0.00
MTPAP	55149	0	0	0	0	0.60	0.00	0.00
MTR	4548	0.41	3.45	0.97	1.25	0.91	0.00	0.00
MTREX	23517	1.84	8.86	1.32	3.07	1.26	0.00	0.00
MTRF1	9617	1.14	2.7	0.78	1.7	0.73	0.00	0.00
MTRF1L	54516	3.07	7.52	2.8	3.51	0.65	0.00	0.00
MTX1	4580	77.5	32.11	38.03	34.82	0.58	0.00	0.00
MTX3	345778	0.12	0.87	0.07	0.41	1.04	0.00	0.00
MVB12A	93343	80.65	32.69	40.29	34.19	0.62	0.00	0.00
MVD	4597	130.61	21.68	45.57	40.39	0.84	0.00	0.00
MVK	4598	134.26	197.25	198.04	190.72	0.74	0.00	0.00
MYBL1	4603	0.13	0.97	0.09	0.28	0.99	0.00	0.00
MYBL2	4605	315.32	148.02	117.43	115.61	1.00	0.00	0.00
MYBPH	4608	0.42	0.99	0.19	0.06	2.52	0.00	0.00
MYCBP2	23077	0.3	4.03	0.72	1.03	1.30	0.00	0.00
MYH10	4628	3.87	20.79	6.99	7.2	0.78	0.00	0.00
MYH15	22989	0.23	1.81	0.03	0.12	2.55	0.00	0.00
MYNN	55892	0.79	2.42	0.58	0.92	0.92	0.00	0.00
MYO1B	4430	4.57	20.62	5.38	8.33	1.04	0.00	0.00
MYO5A	4644	0.26	1.05	0.28	0.59	0.61	0.00	0.00

MYO5C	55930	0.48	3.66	0.73	1.72	0.74	0.00	0.00
MYO9A	4649	0.05	0.84	0.27	0.38	0.62	0.00	0.00
MYSM1	114803	0.03	0.03	0	0	0.73	0.00	0.00
MZT1	440145	1.57	1.61	1.11	0.91	0.64	0.00	0.00
N4BP2	55728	0.13	0.34	0.09	0.12	1.58	0.00	0.00
N4BP2L2	10443	1.57	7.8	2.24	2.78	1.03	0.00	0.00
NAA15	80155	3.03	9.6	2.1	2.97	1.19	0.00	0.00
NAA16	79612	0.53	2.94	1.05	1.05	0.87	0.00	0.00
NAA25	80018	0.89	5.65	1.37	1.87	0.94	0.00	0.00
NAA30	122830	0.55	2.4	0.74	1.1	0.66	0.00	0.00
NAA35	60560	2.95	7.61	3.19	4.4	0.61	0.00	0.00
NAA38	84316	64.65	21.9	26.44	30.08	0.65	0.00	0.00
NAA50	80218	0.02	0.04	0.02	0	1.49	0.00	0.00
NAALAD2	10003	0.13	0.79	0.13	0.11	2.39	0.00	0.00
NAB1	4664	2	8.24	2.02	3.44	0.92	0.00	0.00
NACC1	112939	90.02	49.62	47.34	45.57	0.59	0.00	0.00
NAE1	8883	14.56	43.87	11.99	18.08	0.93	0.00	0.00
NAMPT	10135	2.47	8.44	2.95	3.25	0.80	0.00	0.00
NANP	140838	1.04	2.45	1.39	1.77	0.66	0.00	0.00
NAP1L5	266812	0.71	1.56	0.34	0.53	1.39	0.00	0.00
NARS	4677	12.75	45.07	16.17	17.88	0.77	0.00	0.00
NAT8	9027	13.05	3.83	2.97	8.35	0.59	0.00	0.00
NAT8L	339983	16.55	10.2	7.15	3.95	1.27	0.00	0.00
NBEAL1	65065	0.6	1.29	0.89	0.52	1.13	0.00	0.00
NBN	4683	0.95	4.69	0.75	1.56	0.91	0.00	0.00
NCAPG	64151	2.08	8.68	1.3	2.54	1.46	0.00	0.00
NCAPG2	54892	3.07	14.33	5.58	6.4	0.60	0.00	0.00
NCAPH2	29781	66.29	30.7	25.96	25.82	0.86	0.00	0.00
NCBP1	4686	3.55	14.4	3.41	5.71	0.87	0.00	0.00
NCK1	4690	1.68	3.38	1.41	2.14	0.65	0.00	0.00
NCKAP1	10787	7.85	30.49	6.69	14.32	0.85	0.00	0.00
NCL	4691	112.84	337.49	100.32	136.16	0.85	0.00	0.00
NCLN	56926	152.66	54.53	52.91	51.97	0.99	0.00	0.00

NDC80	10403	2.95	7.09	0.98	2.59	1.52	0.00	0.00
NDOR1	27158	31.81	12.26	12.29	16.24	0.63	0.00	0.00
NDUFA11	126328	479.71	182.27	212.9	185.33	0.75	0.00	0.00
NDUFA13	51079	504.05	192.62	236.46	189.57	0.73	0.00	0.00
NDUFA5	4698	12.12	13.19	3.58	8.19	1.10	0.00	0.00
NDUFAF8	284184	120.47	55.29	50.92	57.4	0.59	0.00	0.00
NDUFB7	4713	609.44	191.95	217.28	201.74	0.96	0.00	0.00
NDUFS7	374291	290.13	88.66	121.45	103.66	0.76	0.00	0.00
NEDD1	121441	0.55	2.72	0.5	0.77	1.46	0.00	0.00
NEDD4	4734	0.9	2.97	0.55	1.41	0.91	0.00	0.00
NEIL3	55247	0.58	2.11	0.43	0.73	1.16	0.00	0.00
NEK1	4750	0.22	1.17	0.17	0.4	1.08	0.00	0.00
NEK2	4751	2.91	10.51	3.23	4.25	0.85	0.00	0.00
NEK4	6787	1.12	3.93	1	1.35	0.74	0.00	0.00
NEK7	140609	0.76	2.82	0.43	1.04	1.24	0.00	0.00
NELFB	25920	83.42	42.39	43.43	41.41	0.58	0.00	0.00
NEMP1	23306	0.83	4.24	1.29	1.93	0.67	0.00	0.00
NEU4	129807	235	67.98	68.63	85.69	0.96	0.00	0.00
NFATC3	4775	4.54	16.82	3.93	5.62	1.09	0.00	0.00
NFKBIE	4794	21.67	9.43	6.87	7.61	1.06	0.00	0.00
NFKBIZ	64332	0.34	6.25	3.32	1.17	0.58	0.00	0.00
NFXL1	152518	0.59	2.67	0.41	1.21	0.91	0.00	0.00
NFYB	4801	1.85	4.44	1.08	1.38	1.29	0.00	0.00
NGLY1	55768	4.16	10.51	4.03	5.14	0.61	0.00	0.00
NHLRC2	374354	0.73	1.72	0.84	0.39	0.96	0.00	0.00
NIFK	84365	12.6	19.73	7.03	10.12	0.91	0.00	0.00
NIPBL	25836	0.26	1.9	0.32	0.86	0.81	0.00	0.00
NKAP	79576	3.83	7.83	2.91	3.57	0.82	0.00	0.00
NKAPD1	55216	0.48	1.02	0.44	0.22	0.94	0.00	0.00
NKTR	4820	0.15	2.53	0.73	0.97	0.67	0.00	0.00
NKX2-5	1482	4.35	2.48	2.75	1.5	0.72	0.00	0.00
NLGN4Y	22829	0.77	2.04	0.53	0.71	1.12	0.00	0.00
NLN	57486	4.43	14.03	4.41	5.89	0.83	0.00	0.00

NLRP12	91662	0	0	0	0.07	2.34	0.00	0.00
NMD3	51068	3.23	11.54	2.63	4	1.09	0.00	0.00
NME7	29922	1.59	4.21	1.58	1.3	1.15	0.00	0.00
NOC3L	64318	1.68	4.61	0.92	1.03	1.68	0.00	0.00
NOL10	79954	7.68	18.34	6.33	8.06	0.89	0.00	0.00
NOL11	25926	12.14	32.65	9.61	15.68	0.81	0.00	0.00
NOL8	55035	1.17	5.12	0.71	1.57	1.45	0.00	0.00
NOP58	51602	9.6	26.86	5.61	10.55	1.05	0.00	0.00
NORAD	647979	5.71	31.56	8.52	11.42	0.85	0.00	0.00
NOSIP	51070	143.37	47.42	46.63	43.32	1.10	0.00	0.00
NPAT	4863	0.2	0.84	0.16	0.28	1.23	0.00	0.00
NPHP1	4867	0.22	1.14	0.33	0.18	1.35	0.00	0.00
NPHP3-ACAD11	100532724	0	0	0	0	0.65	0.00	0.00
NPM1	4869	382.47	623.28	269.62	309.05	0.79	0.00	0.00
NR2C1	7181	3.83	3.46	3.93	2.13	0.66	0.00	0.00
NR3C1	2908	0.51	4.44	1.02	1.33	1.08	0.00	0.00
NR5A2	2494	0.56	1.42	0.26	0.73	0.93	0.00	0.00
NRBF2	29982	5.91	14.76	7.04	6.88	0.59	0.00	0.00
NRCAM	4897	0.28	1.74	0.55	0.42	1.11	0.00	0.00
NRG4	145957	1.06	1.73	0.68	0.9	0.74	0.00	0.00
NRGN	4900	15.38	9.67	7.68	6.7	0.74	0.00	0.00
NRIP1	8204	1.08	5.79	1.04	1.69	1.27	0.00	0.00
NRP2	8828	2.34	11.18	5.19	2.73	0.78	0.00	0.00
NSA2	10412	26.86	39.88	15.77	16.51	1.02	0.00	0.00
NSD3	54904	0.4	3.4	1.05	1.36	0.85	0.00	0.00
NSL1	25936	10.19	14.79	6.46	7.83	0.71	0.00	0.00
NSMF	26012	192.05	87.11	110.71	71.88	0.70	0.00	0.00
NSRP1	84081	0.44	2.93	1	1.16	0.66	0.00	0.00
NSUN3	63899	0.45	1.32	0.49	0.45	0.84	0.00	0.00
NSUN6	221078	1.55	6.72	1.56	2.2	0.87	0.00	0.00
NT5C1B-RDH14	100526794	1.36	3.52	1.57	0.51	1.22	0.00	0.00
NT5C2	22978	9.15	20	7.98	10.88	0.63	0.00	0.00
NT5C3A	51251	2.27	8.18	2.56	4.18	0.61	0.00	0.00

NT5DC2	64943	444.34	173.48	182.45	168.12	0.82	0.00	0.00
NTMT1	28989	72.72	29.98	30.24	28.83	0.77	0.00	0.00
NUBP2	10101	117.99	34.87	49.12	43.73	0.73	0.00	0.00
NUBPL	80224	0.79	3.07	1.12	1.59	0.70	0.00	0.00
NUCB2	4925	4.81	7.73	1.81	3.66	1.15	0.00	0.00
NUCKS1	64710	9.93	40.81	12.25	14.9	0.88	0.00	0.00
NUDCD1	84955	2.3	6.44	1.57	2.34	1.12	0.00	0.00
NUDT1	4521	29.15	13.53	12.59	15.77	0.60	0.00	0.00
NUDT16L1	84309	71.49	23.62	26.11	31.24	0.73	0.00	0.00
NUDT21	11051	9.12	38.03	13.94	16.69	0.78	0.00	0.00
NUDT4B	440672	0.44	3.21	0.93	1.38	0.65	0.00	0.00
NUDT4P2	170688	2.21	4.16	1.96	1.53	0.86	0.00	0.00
NUF2	83540	3	18.22	4.2	8.2	1.65	0.00	0.00
NUFIP2	57532	1.21	6.16	1.21	1.73	0.95	0.00	0.00
NUP107	57122	1.9	9.7	1.56	2.51	1.42	0.00	0.00
NUP155	9631	2.4	12.25	3.7	4.33	0.80	0.00	0.00
NUP35	129401	6.1	16.54	5.36	6.87	0.90	0.00	0.00
NUP50	10762	2.96	12.85	2.85	4.19	1.21	0.00	0.00
NUP54	53371	4.5	11.94	3.85	5.44	0.80	0.00	0.00
NUP58	9818	3.5	17.36	4.91	5.12	1.04	0.00	0.00
NUP88	4927	0.91	0	0.81	0.71	0.77	0.00	0.00
NUSAP1	51203	9.41	31.88	8.94	11.19	1.04	0.00	0.00
NXT2	55916	2.35	3.17	0.86	1.99	0.96	0.00	0.00
ODAM	54959	41.64	131.87	43.75	23.42	1.36	0.00	0.00
ODR4	54953	0.72	2.35	0.43	0.86	1.23	0.00	0.00
OIP5-AS1	729082	1.38	6.65	2.49	2.88	0.65	0.00	0.00
OLFML1	283298	0.31	0.23	0	0.08	2.90	0.00	0.00
OLFML3	56944	8.33	6.41	2.9	3.29	1.26	0.00	0.00
OMA1	115209	1.23	4.38	1.54	1.66	0.77	0.00	0.00
OPA1	4976	1.6	7.81	1.61	2.81	1.07	0.00	0.00
ORAI1	84876	42.51	18.39	18.82	20.89	0.63	0.00	0.00
ORC3	23595	3.33	8.2	2.13	2.74	1.14	0.00	0.00
ORC4	5000	6.89	11.6	3.22	4.73	0.82	0.00	0.00

ORC5	5001	3.63	10.18	3.56	4.99	0.69	0.00	0.00
OSBP2	23762	0	0.27	0	0	5.07	0.00	0.00
OSBPL8	114882	1.08	3.52	0.76	1.4	1.14	0.00	0.00
OSTM1	28962	2.79	5.05	1.09	2.12	1.20	0.00	0.00
OTUD3	23252	0.41	4.22	1.71	1.24	0.68	0.00	0.00
OTUD4	54726	1.02	6.5	1.91	2.43	0.79	0.00	0.00
OVCA2	124641	101.67	33.99	35.1	35.7	0.95	0.00	0.00
OXR1	55074	1.05	2.97	0.42	0.8	1.78	0.00	0.00
P4HA1	5033	4.32	32.01	12.67	7.57	0.83	0.00	0.00
PAFAH1B1	5048	14.57	14.19	11.79	12.38	0.63	0.00	0.00
PAK1IP1	55003	13.61	27.05	9.13	10.65	1.09	0.00	0.00
PALB2	79728	1.85	6.5	1.72	2.35	1.05	0.00	0.00
PALM2-AKAP2	445815	0.06	0.61	0	0.14	2.19	0.00	0.00
PANK3	79646	1.89	4.19	2.32	3.37	0.62	0.00	0.00
PAPOLA	10914	0	0	0	0	0.93	0.00	0.00
PAQR3	152559	0.65	4.05	1.32	1.61	0.83	0.00	0.00
PAQR4	124222	46.6	22.89	18.25	21.94	0.83	0.00	0.00
PARG	8505	0	0	0	0	1.07	0.00	0.00
PARGP1	728407	0.34	1.38	0.43	0.49	0.62	0.00	0.00
PARN	5073	7.01	19.17	2.69	10.25	1.00	0.00	0.00
PARP2	10038	6.93	19.41	7.82	9.21	0.61	0.00	0.00
PARPBP	55010	1.94	4.49	0.94	2.02	1.14	0.00	0.00
PAWR	5074	1.57	6	2.65	3.04	0.60	0.00	0.00
PAXBP1	94104	1.29	5.96	1.76	1.72	1.01	0.00	0.00
PBDC1	51260	7.12	11.69	4.27	6.01	0.98	0.00	0.00
PBK	55872	3.87	13.64	3.56	4.58	1.09	0.00	0.00
PBRM1	55193	0.71	3.88	0.93	1.76	0.63	0.00	0.00
PBX3	5090	0.65	2.02	0.53	0.83	0.75	0.00	0.00
PCDHB19P	84054	0.05	0.21	0.04	0.03	1.79	0.00	0.00
PCDHGA1	56114	0.09	0.13	0	0.03	3.04	0.00	0.00
PCED1B	91523	2.79	1.99	1.61	1.1	0.81	0.00	0.00
PCLAF	9768	20.51	27.33	13.68	17.06	0.74	0.00	0.00
PCM1	5108	0.98	4.98	0.92	2.02	0.94	0.00	0.00

PCNX4	64430	1.81	7.56	1.48	3.15	0.83	0.00	0.00
PCYT2	5833	285.61	75.53	84.24	121.83	0.69	0.00	0.00
PDCD10	11235	10.56	14.59	5.18	7.23	1.00	0.00	0.00
PDCL	5082	1.93	5.24	2.66	2.06	0.63	0.00	0.00
PDE11A	50940	0	0	0	0	0.91	0.00	0.00
PDE3B	5140	1.12	4.26	1.04	2.26	0.60	0.00	0.00
PDE4D	5144	1.35	6.31	1.37	1.72	1.20	0.00	0.00
PDIK1L	149420	0.35	0.45	0.16	0.16	0.87	0.00	0.00
PDK1	5163	3.33	10.76	4.26	3.78	0.83	0.00	0.00
PDS5A	23244	1.85	12.21	2.36	5.1	1.01	0.00	0.00
PDS5B	23047	0.53	3.56	0.67	1.25	1.01	0.00	0.00
PDXP	57026	132.92	36.23	44.09	49.81	0.86	0.00	0.00
PDZK1	5174	8.15	19.97	5.61	10.89	0.66	0.00	0.00
PDZK1IP1	10158	2.8	0.27	0.34	0.55	1.81	0.00	0.00
PEAK1	79834	0.34	2.23	0.82	0.96	0.66	0.00	0.00
PEG10	23089	64.46	386.25	102.31	188.35	0.62	0.00	0.00
PEG3	5178	7.69	73.44	13.38	15.81	1.46	0.00	0.00
PELI1	57162	1.17	5.49	1.69	1.8	0.91	0.00	0.00
PELI3	246330	3.04	1.89	1.61	1.01	0.91	0.00	0.00
PET100	100131801	84.24	42.02	41.03	37.25	0.65	0.00	0.00
PEX10	5192	63.87	24.37	26.34	29.39	0.64	0.00	0.00
PEX3	8504	6.58	13.39	4.71	7.04	0.75	0.00	0.00
PFDN4	5203	37.25	40.73	23.91	19.35	0.83	0.00	0.00
PFKFB4	5210	0	0.02	0.02	0	1.04	0.00	0.00
PFN1	5216	1776.81	826.19	859.21	797.89	0.66	0.00	0.00
PGAP1	80055	0.07	0.82	0.18	0.19	1.18	0.00	0.00
PGF	5228	35.2	13.75	14.08	13.78	0.82	0.00	0.00
PGLYRP2	114770	5.14	0.8	0.45	2.04	1.26	0.00	0.00
PGM2	55276	2.27	8.79	3.37	4.35	0.59	0.00	0.00
PGM2L1	283209	0.01	0.2	0.03	0.01	2.46	0.00	0.00
PGM3	5238	6.14	21.28	6.25	7.22	0.94	0.00	0.00
PHACTR2	9749	0.4	1.66	0.38	0.68	0.97	0.00	0.00
PHAX	51808	0.93	3.14	0.9	1.29	0.90	0.00	0.00

PHETA1	144717	35.4	13.7	14.58	18.17	0.60	0.00	0.00
PHF14	9678	2.46	5.84	1.2	2.37	1.07	0.00	0.00
PHF3	23469	0.76	4.6	1.21	1.97	0.87	0.00	0.00
PHF6	84295	0.64	3.37	0.63	0.93	1.22	0.00	0.00
PHKG2	5261	7.41	1.82	1.03	3.26	1.12	0.00	0.00
PHLDB2	90102	0.85	2.48	0.57	0.99	1.23	0.00	0.00
PHOSPHO2-KLHL23	100526832	2.41	7.76	2.32	3.29	0.76	0.00	0.00
PHTF1	10745	1.1	3.43	1.14	1.59	0.60	0.00	0.00
PHTF2	57157	0.59	1.74	0.57	0.83	0.61	0.00	0.00
PI4K2B	55300	2.81	8.07	2.24	3.51	0.77	0.00	0.00
PIAS1	8554	0.72	4.09	0.71	1.44	1.02	0.00	0.00
PIBF1	10464	0.97	1.6	0.22	0.45	2.09	0.00	0.00
PIGA	5277	0.46	2.4	0.53	0.55	1.46	0.00	0.00
PIGK	10026	3.29	5.31	1.99	2.64	1.09	0.00	0.00
PIGQ	9091	29.62	12.49	12.53	9.61	0.94	0.00	0.00
PIH1D2	120379	0.18	0.68	0.11	0.09	3.07	0.00	0.00
PIK3C2A	5286	1.03	3.88	0.86	1.8	0.97	0.00	0.00
PIK3C3	5289	1.85	7.82	1.92	3.01	0.96	0.00	0.00
PIK3CA	5290	0.23	1.57	0.2	0.33	1.27	0.00	0.00
PIK3R3	8503	0.79	1.3	0.46	0.77	0.84	0.00	0.00
PIK3R4	30849	0.83	4.75	1.53	2.2	0.58	0.00	0.00
PIKFYVE	200576	0.35	2.43	0.61	0.73	1.19	0.00	0.00
PIMREG	54478	41.35	32.54	18.67	16.83	1.06	0.00	0.00
PITX1	5307	92.08	25.38	33.91	36.19	0.76	0.00	0.00
PJA2	9867	1.37	7.86	1.72	2.65	1.09	0.00	0.00
PKD1P4-NPIPA8	110006323	2.04	2.11	1.19	1.17	0.78	0.00	0.00
PKIB	5570	16.45	13.15	6.52	10.49	0.90	0.00	0.00
PKMYT1	9088	6.96	9.11	10.09	8.62	0.72	0.00	0.00
PKN2	5586	0.89	4	1.03	1.38	1.03	0.00	0.00
PLAG1	5324	0.07	0.6	0.12	0.24	0.89	0.00	0.00
PLBD1	79887	0.05	0.19	0	0	8.82	0.00	0.00
PLCB1	23236	1.13	5.01	1.35	2.28	0.86	0.00	0.00
PLEKHA8	84725	0.83	4.06	1.13	1.86	0.87	0.00	0.00

PLEKHA8P1	51054	0.06	0.93	0.22	0.15	1.20	0.00	0.00
PLEKHF2	79666	1.4	3.13	0.77	1.32	1.06	0.00	0.00
PLEKHJ1	55111	224.58	79.09	97.59	100.01	0.64	0.00	0.00
PLEKHO2	80301	4.43	3.67	2.94	2.25	0.66	0.00	0.00
PLK1	5347	181.86	123.25	101.93	96.88	0.59	0.00	0.00
PLK4	10733	0.74	4.09	0.59	1.15	1.48	0.00	0.00
PLOD2	5352	5.23	46.48	10.24	12.01	1.19	0.00	0.00
PLP1	5354	0.97	4.64	0.9	0.21	2.32	0.00	0.00
PLPP7	84814	16.55	6.62	5.81	5.01	0.85	0.00	0.00
PLS1	5357	0	0.01	0	0	0.96	0.00	0.00
PLS3	5358	9.39	32.71	7.98	8.6	1.34	0.00	0.00
PLXDC2	84898	0.01	0.08	0.04	0	1.84	0.00	0.00
PM20D2	135293	1.39	4.3	1.7	1.85	0.65	0.00	0.00
PMAIP1	5366	0.68	1.46	0.35	0.29	1.76	0.00	0.00
PMP22	5376	3.56	6.47	2.39	1.14	1.52	0.00	0.00
PMS1	5378	1.87	4.74	1.37	2.23	0.73	0.00	0.00
PNLIPRP2	5408	3.71	2.49	1.4	2.17	0.80	0.00	0.00
PNN	5411	4.35	13.35	3.43	5.28	0.87	0.00	0.00
PNPLA8	50640	0.55	1.88	0.39	0.75	1.12	0.00	0.00
PNPT1	87178	4.38	16.52	5.69	6.51	0.86	0.00	0.00
PNRC2	55629	19.19	58.71	14.04	26.31	0.94	0.00	0.00
POC1B-AS1	109729146	0	0	0	0	0.64	0.00	0.00
POC5	134359	1.07	2.47	0.94	1.03	0.80	0.00	0.00
PODN	127435	0.28	0.41	0.21	0.19	0.80	0.00	0.00
POLA1	5422	0.88	5.41	1.46	2.5	0.94	0.00	0.00
POLA2	23649	20.87	18.31	11.42	11.7	0.65	0.00	0.00
POLD2	5425	201.79	75.25	92.7	82.51	0.67	0.00	0.00
POLD3	10714	2.12	7.2	2.22	2.08	1.03	0.00	0.00
POLE4	56655	130.73	57.28	58.26	62.96	0.65	0.00	0.00
POLI	11201	0.5	2.54	0.72	0.9	0.75	0.00	0.00
POLQ	10721	1.31	2.27	1.8	1.11	1.22	0.00	0.00
POLR2B	5431	6.09	26.1	9.1	12.12	0.59	0.00	0.00
POLR2E	5434	486.17	202.54	224.79	226.76	0.61	0.00	0.00

POLR2M	81488	1.66	4.15	1.68	2.09	0.62	0.00	0.00
POLR3B	55703	1.35	4.11	1.71	1.66	0.82	0.00	0.00
POLR3F	10621	1.98	5.73	2.22	2.67	0.64	0.00	0.00
POLR3G	10622	1.21	2.6	1.05	0.77	1.34	0.00	0.00
POMGNT2	84892	21.47	10.99	9.48	9.58	0.77	0.00	0.00
POP1	10940	1.71	4.95	1.93	1.7	0.63	0.00	0.00
POP7	10248	135.44	70.24	61.14	63.39	0.73	0.00	0.00
POT1	25913	0.52	3.54	0.66	0.89	1.42	0.00	0.00
POU4F1	5457	4.01	5.59	4	5.22	0.78	0.00	0.00
PPAT	5471	1.51	9.43	2.16	3.41	0.97	0.00	0.00
PPIG	9360	3.57	8.27	2.67	5.03	0.66	0.00	0.00
PPIL3	53938	20.52	24.98	12.68	14.87	0.73	0.00	0.00
PPIL4	85313	3.02	7.52	2.42	3.54	0.82	0.00	0.00
PPIP5K2	23262	1.2	3.81	0.44	1.11	1.59	0.00	0.00
PPM1K	152926	0.61	2.32	0.7	0.95	0.92	0.00	0.00
PPP1CB	5500	6.19	15.81	5.14	7.66	0.77	0.00	0.00
PPP1R12A	4659	0.73	3.78	1.09	1.42	0.85	0.00	0.00
PPP1R14B	26472	256.63	89.32	110.18	111.96	0.65	0.00	0.00
PPP1R16A	84988	0	0.02	0	0	0.68	0.00	0.00
PPP1R1C	151242	4.02	2.6	2.25	2.3	0.84	0.00	0.00
PPP1R3G	648791	1.91	1.11	0.71	0.92	0.89	0.00	0.00
PPP1R9A	55607	0.2	1.76	0.4	0.53	1.09	0.00	0.00
PPP2R2C	5522	0.06	0.1	0.06	0.01	1.43	0.00	0.00
PPP2R5D	5528	2.06	7.72	2.75	3.19	0.83	0.00	0.00
PPP4R2	151987	4.43	13.16	3.06	5.74	0.90	0.00	0.00
PPP4R3A	55671	2.82	10.13	3.03	5.08	0.64	0.00	0.00
PPP4R3B	57223	2.78	10.67	2.66	4.24	0.91	0.00	0.00
PPP6R3	55291	3.6	13.82	3.65	5.42	0.89	0.00	0.00
PPWD1	23398	5.63	4.72	6.18	6.82	1.23	0.00	0.00
PQLC1	80148	83.25	24.45	31.91	33.95	0.73	0.00	0.00
PRADC1	84279	57.58	16.87	14.18	20.79	1.11	0.00	0.00
PRAF2	11230	0.73	0	0	0	5.06	0.00	0.00
PRC1	9055	9.9	37.4	15.53	15.36	0.58	0.00	0.00

PREB	10113	200.82	103.9	106.46	90.94	0.63	0.00	0.00
PREPL	9581	2.25	11.1	2.53	3.9	1.04	0.00	0.00
PRIM1	5557	8.71	20.17	7.45	8.97	0.88	0.00	0.00
PRIMPOL	201973	0	0	0.14	0	1.28	0.00	0.00
PRKAA1	5562	1.8	6.68	2.15	3.33	0.68	0.00	0.00
PRKAB2	5565	2.09	10.19	4.2	3.71	0.62	0.00	0.00
PRKCI	5584	1.04	8.08	1.66	2.91	1.01	0.00	0.00
PRKD3	23683	0.94	6.3	0.95	1.61	1.30	0.00	0.00
PRLR	5618	0.16	0.48	0.06	0.22	1.20	0.00	0.00
PRMT3	10196	3.12	6.7	2.24	3.48	0.76	0.00	0.00
PRMT9	90826	36.24	30.93	27.4	27.69	1.35	0.00	0.00
PROM1	8842	4.02	15.4	3.33	4.49	1.31	0.00	0.00
PRPF18	8559	1.4	4.42	0.96	1.34	1.14	0.00	0.00
PRPF38B	55119	1.79	5.73	1.25	1.66	1.29	0.00	0.00
PRPF39	55015	0.78	3.5	0.79	1.01	1.07	0.00	0.00
PRPF40A	55660	7.78	18.55	3.97	7.19	1.13	0.00	0.00
PRPF4B	8899	2.38	11.6	2.13	4.06	1.07	0.00	0.00
PRR5	55615	42.81	13.08	17.35	19.54	0.63	0.00	0.00
PRRG1	5638	0.39	1.5	0.59	0.57	0.71	0.00	0.00
PRTG	283659	0.34	2.32	0.57	0.58	1.49	0.00	0.00
PSD3	23362	0.24	1.62	0.49	0.67	0.90	0.00	0.00
PSIP1	11168	3.26	15.02	3.54	4.1	1.28	0.00	0.00
PSKH1	5681	84.82	26.78	36.04	34.75	0.67	0.00	0.00
PSMA3	5684	52.5	69.74	33.16	45.56	0.63	0.00	0.00
PSMA4	5685	50.89	75.44	35.38	35.62	0.81	0.00	0.00
PSMC2	5701	0.37	3.68	0.25	0.41	2.96	0.00	0.00
PSMC6	5706	17.94	36.09	8.6	13.69	1.26	0.00	0.00
PSMD1	5707	15.07	47.19	15.73	21.12	0.74	0.00	0.00
PSMD12	5718	6.06	13.7	5.87	7.06	0.61	0.00	0.00
PSME4	23198	3.89	7.63	2.74	2.81	0.80	0.00	0.00
PSMG3	84262	38	18.55	19.35	18.5	0.60	0.00	0.00
PSORS1C3	100130889	0.01	0.24	0.03	0.05	1.68	0.00	0.00
PTAR1	375743	0.5	1.27	0.38	0.64	0.73	0.00	0.00

PTBP2	58155	0.24	1.83	0.4	0.57	1.13	0.00	0.00
PTBP3	9991	3.06	14.71	4.77	6.99	0.62	0.00	0.00
PTCD2	79810	1.64	3.25	1.46	1.35	0.62	0.00	0.00
PTER	9317	1.77	5.05	1.55	2.13	0.88	0.00	0.00
PTGR2	145482	0.69	1.39	0.33	0.47	1.36	0.00	0.00
PTPA	5524	150.57	66.2	71.35	73.03	0.60	0.00	0.00
PTPN11	5781	2.51	14.59	3.67	5.08	0.95	0.00	0.00
PTPRG	5793	1.15	5.87	1.88	2.09	0.87	0.00	0.00
PTRHD1	391356	142.41	51.99	56.1	65.69	0.69	0.00	0.00
PUM2	23369	2.93	16.67	4.78	7.53	0.68	0.00	0.00
PUM3	9933	2.55	9.26	2.63	3.6	0.87	0.00	0.00
PURA	5813	0.57	0.57	0.48	0.59	0.65	0.00	0.00
PUS3	83480	3.06	5.99	2.44	3.52	0.65	0.00	0.00
PUS7L	83448	0.65	1.47	0.25	0.62	1.11	0.00	0.00
PUSL1	126789	53.31	21.82	25.65	24.76	0.61	0.00	0.00
PXYLP1	92370	0.58	1.53	0.82	0.44	0.75	0.00	0.00
PYCR3	65263	5.09	1.04	0.86	1.94	1.14	0.00	0.00
QKI	9444	6.35	22.59	5.96	8.53	0.68	0.00	0.00
QSER1	79832	0.49	3.83	0.71	1.48	0.95	0.00	0.00
R3HCC1L	27291	0.33	3.37	1.16	1.21	0.87	0.00	0.00
RAB18	22931	4.95	14.18	3.89	5.97	1.01	0.00	0.00
RAB23	51715	0.79	3.84	0.58	1.02	1.50	0.00	0.00
RAB30	27314	0.17	0.62	0.29	0.16	0.83	0.00	0.00
RAB33B	83452	0.47	1.54	0.57	0.65	0.69	0.00	0.00
RAB34	83871	89.52	39.58	41.37	39.15	0.68	0.00	0.00
RAB3GAP2	25782	0.67	3.73	1.09	1.42	1.00	0.00	0.00
RAB8B	51762	0.48	2.02	0.6	0.51	1.16	0.00	0.00
RABEP1	9135	5.77	8.14	3	3.68	1.07	0.00	0.00
RABGGTB	5876	12.76	41.54	17.25	20.48	0.61	0.00	0.00
RAD18	56852	1	4.09	0.78	1.21	1.13	0.00	0.00
RAD21	5885	9.06	42.11	9.78	12.73	1.17	0.00	0.00
RAD50	10111	0.67	2.72	0.63	1.52	0.69	0.00	0.00
RAD51AP1	10635	4.54	5.9	1.05	2.46	1.59	0.00	0.00

RAD51C	5889	19.33	34.45	15.73	18.25	0.76	0.00	0.00
RAD54B	25788	0.45	3.25	0.45	0.67	1.69	0.00	0.00
RAI14	26064	1.02	6.32	1.44	2.05	1.05	0.00	0.00
RALGAPA1	253959	0.33	1.91	0.49	0.87	0.82	0.00	0.00
RALGPS2	55103	0.79	3.21	0.75	0.98	0.93	0.00	0.00
RANBP2	5903	1.06	6.67	0.8	2.14	1.50	0.00	0.00
RANBP6	26953	1.48	0.87	1.77	1.19	1.22	0.00	0.00
RAP2A	5911	1.97	5.8	1.55	2.52	0.90	0.00	0.00
RAPGEF2	9693	0.59	3.8	0.89	1.65	0.80	0.00	0.00
RAPGEF5	9771	0.69	2.31	0.96	0.87	0.66	0.00	0.00
RAPGEF6	51735	0.28	1.61	0.2	0.65	1.18	0.00	0.00
RARS	5917	10.96	47.13	14.61	17.16	0.86	0.00	0.00
RASA2	5922	0.19	1.14	0.32	0.28	0.98	0.00	0.00
RASGEF1B	153020	0.45	1.52	0.37	0.44	1.33	0.00	0.00
RASGRF2	5924	1.81	6.43	2.54	2.52	0.63	0.00	0.00
RASL10B	91608	63.14	18	16.72	16.37	1.30	0.00	0.00
RASSF7	8045	65.89	123.87	96.69	96.88	0.60	0.00	0.00
RAVER2	55225	0.32	1.95	0.66	0.7	0.88	0.00	0.00
RB1	5925	1.46	7.9	1.6	3.42	0.89	0.00	0.00
RB1CC1	9821	0.62	2.25	0.51	0.85	1.03	0.00	0.00
RBBP8	5932	2.73	7.94	1.62	3.34	1.11	0.00	0.00
RBBP9	10741	8.12	19.07	5.87	8.56	0.90	0.00	0.00
RBL1	5933	1.36	7.18	1.38	2.23	1.12	0.00	0.00
RBM12B	389677	0.41	1.9	0.42	0.51	0.95	0.00	0.00
RBM14	10432	18.27	13.46	10.49	10.16	0.62	0.00	0.00
RBM25	58517	4.12	12.57	3.34	6.13	0.77	0.00	0.00
RBM26	64062	0	0.07	0.07	0	0.59	0.00	0.00
RBM27	54439	0.82	3.88	1.11	1.32	0.91	0.00	0.00
RBM34	23029	5.15	9.82	2.93	4.14	1.01	0.00	0.00
RBM39	9584	105.02	58.3	69.72	66.58	0.92	0.00	0.00
RBM7	10179	1.2	4.39	1.37	2.04	0.73	0.00	0.00
RBMS1	5937	2.23	11.06	3.79	3.97	0.74	0.00	0.00
RBMXL1	494115	1.36	3.3	1.33	1.74	0.60	0.00	0.00

RBPJ	3516	0.77	6.18	1.54	2.18	0.86	0.00	0.00
RC3H1	149041	0.2	1.22	0.16	0.74	0.63	0.00	0.00
RCE1	9986	0.02	0	0	0	0.65	0.00	0.00
RCHY1	25898	6.67	6.84	2.96	5.36	0.64	0.00	0.00
RCOR2	283248	11.84	11.78	8.2	6.51	0.71	0.00	0.00
RDX	5962	3.64	18.96	5.19	7.36	0.75	0.00	0.00
RECQL	5965	0.9	4.76	0.43	1.47	1.72	0.00	0.00
RECQL4	9401	31.49	16.12	13.35	15.57	0.76	0.00	0.00
RELB	5971	9.47	2.64	4.24	3.66	0.63	0.00	0.00
RELCH	57614	0.6	3.8	0.96	1.84	0.63	0.00	0.00
RELT	84957	3.23	2.67	2.13	1.96	0.59	0.00	0.00
REST	5978	0.31	1.75	0.59	0.75	0.60	0.00	0.00
REV1	51455	0.91	5.32	1.76	1.96	0.74	0.00	0.00
REX1BD	55049	29	13.82	12.74	13.41	0.65	0.00	0.00
RFC1	5981	2.44	11.5	2.09	3.52	1.20	0.00	0.00
RFC3	5983	7.55	20.38	7.92	10.85	0.61	0.00	0.00
RFK	55312	6.08	12.98	4.89	6.35	0.79	0.00	0.00
RFX7	64864	0.7	3.59	1.05	1.68	0.59	0.00	0.00
RGP1	9827	1.87	0	0	0	6.84	0.00	0.00
RGPD5	84220	0.09	1.37	0.5	0.23	0.88	0.00	0.00
RGPD6	729540	0.24	2.15	0.23	0.65	1.43	0.00	0.00
RGPD8	727851	0.1	0.81	0.2	0.28	0.86	0.00	0.00
RHOBTB1	9886	6.74	43.45	15.71	13.18	0.78	0.00	0.00
RHOT1	55288	0	0	0	0	0.66	0.00	0.00
RIBC1	158787	1.33	1.55	0.74	0.93	0.88	0.00	0.00
RIC1	57589	1.17	6.42	1.64	2.67	0.85	0.00	0.00
RICTOR	253260	0.15	0.98	0.23	0.25	1.18	0.00	0.00
RIDA	10247	6.88	11.9	4.11	7.83	0.65	0.00	0.00
RIF1	55183	0.68	2.49	0.47	0.89	1.18	0.00	0.00
RILP	83547	10.95	3.81	2.54	4.65	1.04	0.00	0.00
RIMBP3	85376	0	0	0	0	1.85	0.00	0.00
RIMKLA	284716	0.59	0.67	0.31	0.25	1.12	0.00	0.00
RINT1	60561	2.5	5.63	1.95	2.23	0.91	0.00	0.00

RIOK2	55781	45.56	25.33	23.8	25.76	1.26	0.00	0.00
RIPOR3	140876	2.62	4.33	1.15	1.84	0.88	0.00	0.00
RIT1	6016	0.81	2.37	0.82	1.09	0.61	0.00	0.00
RITA1	84934	73.12	23.58	25.79	38.47	0.60	0.00	0.00
RLF	6018	0.38	2.01	0.52	0.6	1.24	0.00	0.00
RLIM	51132	0.39	2.38	0.74	0.85	0.71	0.00	0.00
RMDN2	151393	0.64	1.26	0.33	0.58	1.21	0.00	0.00
RNASEH2A	10535	102.38	73.91	64.23	53.79	0.58	0.00	0.00
RNASEH2C	84153	21.39	9.7	9.04	8.09	0.91	0.00	0.00
RNASEK	440400	579.41	211.13	248.31	250.41	0.68	0.00	0.00
RND1	27289	1.86	2.19	0.83	1.57	0.76	0.00	0.00
RNF13	11342	2.19	5.73	1.94	3.16	0.62	0.00	0.00
RNF138	51444	1.75	4.89	1.75	2.26	0.86	0.00	0.00
RNF167	26001	148.29	70.44	65.62	68.46	0.72	0.00	0.00
RNF168	165918	0.91	2.85	0.71	1.1	0.98	0.00	0.00
RNF169	254225	0.17	0.69	0.23	0.3	0.73	0.00	0.00
RNF170	81790	1.2	4.16	1.33	1.84	0.75	0.00	0.00
RNF19A	25897	1.08	5.83	1.69	2.33	0.79	0.00	0.00
RNF2	6045	4.59	9.94	3.64	4.29	0.68	0.00	0.00
RNF20	56254	1.66	7.93	2.13	2.57	1.00	0.00	0.00
RNF219	79596	0.92	4.59	0.91	1.3	1.25	0.00	0.00
RNF6	6049	0.92	3.81	0.91	2.07	0.66	0.00	0.00
RNGTT	8732	0.87	4.56	1.11	1.19	1.23	0.00	0.00
RNMT	8731	1.31	8.32	3.4	3.18	0.75	0.00	0.00
ROCK1	6093	0.77	3.14	0.75	1.5	0.87	0.00	0.00
ROCK2	9475	0.09	0.22	0.1	0.21	0.79	0.00	0.00
RPAIN	84268	2.03	2.1	1.34	3.01	1.06	0.00	0.00
RPAP2	79871	0.71	2.61	0.81	1.05	0.90	0.00	0.00
RPAP3	79657	2.61	4.31	1.15	0.88	1.38	0.00	0.00
RPF2	84154	11.89	12.59	4.7	6.02	1.18	0.00	0.00
RPGR	6103	0.55	1.66	0.34	0.72	0.84	0.00	0.00
RPGRIP1L	23322	1.22	2.6	0.73	1.44	1.29	0.00	0.00
RPL18A	6142	5024.16	1959.51	2533.54	2015.68	0.63	0.00	0.00

RPP21	79897	15.71	4.95	7.32	6.3	0.62	0.00	0.00
RPS19BP1	91582	112.99	41.75	46.04	53.36	0.65	0.00	0.00
RPS26	6231	1544.84	530.31	577.65	710.85	0.70	0.00	0.00
RPS6KA3	6197	9.88	60.85	14.94	23.87	0.93	0.00	0.00
RPS6KB1	6198	1.91	8.66	1.56	2.69	0.95	0.00	0.00
RPS6KB2	6199	129.27	39.61	50.58	60.52	0.59	0.00	0.00
RPS6KC1	26750	0.79	3.48	0.92	1.21	0.93	0.00	0.00
RPUSD1	113000	81.07	21.16	25.42	28.61	0.93	0.00	0.00
RRAGD	58528	0.69	0.48	0.07	0.28	1.63	0.00	0.00
RRM1	6240	7.79	33.07	7.83	10.55	1.14	0.00	0.00
RRM2B	50484	0.91	5.37	1.58	2.21	0.75	0.00	0.00
RRP15	51018	1.68	5.31	1.98	2.08	0.90	0.00	0.00
RRP7A	27341	49.3	28.92	24.34	22.49	0.68	0.00	0.00
RSBN1L	222194	0	0	0	0	0.92	0.00	0.00
RSL24D1	51187	35.65	39.94	22.25	20.26	0.83	0.00	0.00
RSPH14	27156	3.02	2.25	0.63	0.55	2.58	0.00	0.00
RSRC1	51319	1.37	5.12	1.69	2.42	0.66	0.00	0.00
RSRC2	65117	3.49	12.22	3.76	4.97	0.86	0.00	0.00
RSRP1	57035	1.67	4.53	0.8	1.56	1.02	0.00	0.00
RUFY2	55680	0.21	1.62	0.41	0.69	0.71	0.00	0.00
RUSC1-AS1	284618	2.09	0.73	0.51	0.51	1.05	0.00	0.00
RWDD1	51389	22.11	24.26	10.1	13.93	0.95	0.00	0.00
RWDD4	201965	7.57	13.66	5.24	7.91	0.68	0.00	0.00
RYBP	23429	1.46	5.82	1.91	2.23	0.80	0.00	0.00
S1PR5	53637	0.13	1.15	0.15	0.23	1.72	0.00	0.00
SAC3D1	29901	60.3	21.65	22.42	23.36	0.83	0.00	0.00
SACM1L	22908	0.56	4.75	1.17	2.05	0.74	0.00	0.00
SACS	26278	0.12	0.68	0.12	0.29	0.96	0.00	0.00
SAMD8	142891	0.33	1.69	0.66	0.74	0.66	0.00	0.00
SASS6	163786	0.27	1.21	0.38	0.4	0.89	0.00	0.00
SAXO1	158297	10.07	6.92	0.29	0.2	4.45	0.00	0.00
SBDSP1	155370	3	5.71	2.4	2.94	0.73	0.00	0.00
SBF1	6305	66.87	25.47	27.77	26.39	0.75	0.00	0.00

SCAF11	9169	1.57	5.17	1.61	2.09	0.91	0.00	0.00
SCAMP1	9522	0.62	4.39	0.79	1.39	0.89	0.00	0.00
SCAMP5	192683	98.72	36.78	33.73	36.18	0.91	0.00	0.00
SCAPER	49855	0.17	1.11	0.26	0.38	1.30	0.00	0.00
SCFD1	23256	5.8	16.11	3.05	7.05	0.94	0.00	0.00
SCN11A	11280	0.04	0.05	0	0	4.91	0.00	0.00
SCO2	9997	54.03	12.54	18.91	23.31	0.68	0.00	0.00
SCOC	60592	8.52	13.91	3.8	7.56	1.02	0.00	0.00
SCP2	6342	15.4	22.89	9.2	15.53	0.64	0.00	0.00
SCRN1	9805	7.9	30.94	13.93	14.06	0.59	0.00	0.00
SCYL2	55681	1.07	3.5	0.81	2.01	0.60	0.00	0.00
SDAD1	55153	2.59	8.73	2.57	4.48	0.71	0.00	0.00
SDF2L1	23753	93.32	38.97	40.3	42.85	0.68	0.00	0.00
SDHAF1	644096	46.19	14.09	19.16	17.45	0.73	0.00	0.00
SDHAF3	57001	5.11	5.94	3.32	3.68	0.66	0.00	0.00
SEC23A-AS1	105370458	0.46	0.5	0.2	0.35	1.09	0.00	0.00
SEC62	7095	0	0.04	0	0	0.84	0.00	0.00
SEC63	11231	3.17	12.38	3.1	6.07	0.77	0.00	0.00
SECISBP2	79048	2.87	9.7	4.11	4.31	0.64	0.00	0.00
SECISBP2L	9728	0.31	1.63	0	0.56	1.82	0.00	0.00
SEL1L	6400	2.1	15.25	3.27	4.99	0.97	0.00	0.00
SELENOI	85465	2.06	9.3	2.56	3.33	0.88	0.00	0.00
SELENOM	140606	42.08	12.31	18.25	13.24	0.81	0.00	0.00
SELENOP	6414	172.35	297.75	95.21	174.24	0.80	0.00	0.00
SEMA6B	10501	346.78	129.59	142.31	115.31	0.81	0.00	0.00
SENP1	29843	1.15	5.43	1.37	1.94	0.99	0.00	0.00
SENP6	26054	1.09	4.93	1.26	2.48	0.68	0.00	0.00
SEPSECS	51091	0.83	1.41	0.46	0.7	0.89	0.00	0.00
SEPT7P2	641977	0.78	2.18	0.96	0.85	0.78	0.00	0.00
SERF2	10169	10.76	4.05	4.01	4.72	0.83	0.00	0.00
SERINC1	57515	4.88	22.69	5.61	9.19	0.88	0.00	0.00
SERPINB9	5272	1.67	5.43	2.15	1.63	0.80	0.00	0.00
SERPINE3	647174	0.11	1.11	0.11	0.31	1.50	0.00	0.00

SETDB2-PHF11	107303344	1.4	3.42	1.39	1.49	0.66	0.00	0.00
SETX	23064	0.58	3.79	1.21	1.71	0.88	0.00	0.00
SF3B1	23451	8.72	59.94	12.26	17.51	1.17	0.00	0.00
SF3B5	83443	491.01	171.63	207.51	206.73	0.69	0.00	0.00
SGCB	6443	0.62	2.59	1.14	0.95	0.61	0.00	0.00
SGK1	6446	6.4	15.96	5.09	4.5	1.20	0.00	0.00
SGK3	23678	0.3	1.51	0.14	0.93	0.70	0.00	0.00
SGO1	151648	1.74	3.32	1.05	1.26	1.09	0.00	0.00
SGO2	151246	0.84	1.71	0.19	0.43	1.97	0.00	0.00
SGSM2	9905	14.17	11.77	9.34	7.46	0.67	0.00	0.00
SGTA	6449	252.86	122.1	114.37	105.04	0.78	0.00	0.00
SGTB	54557	0.94	2.12	0.82	0.98	0.77	0.00	0.00
SHC3	53358	0.02	0.05	0.04	0.03	0.79	0.00	0.00
SHCBP1	79801	3.99	11.71	3.12	4.71	0.90	0.00	0.00
SHLD2	54537	4.24	10.65	2.51	4.15	1.09	0.00	0.00
SHOC2	8036	0.11	3.38	0.32	0.61	1.89	0.00	0.00
SHPK	23729	37.7	16.27	11.76	17.14	0.91	0.00	0.00
SHPRH	257218	0.14	1.39	0.15	0.41	1.49	0.00	0.00
SIRT1	23411	0.47	3.23	0.77	0.96	1.15	0.00	0.00
SIT1	27240	6.54	6.16	4.94	3.4	0.60	0.00	0.00
SIVA1	10572	0.01	0.11	0.25	0.12	0.82	0.00	0.00
SIX4	51804	0.31	2.13	0.71	0.72	0.65	0.00	0.00
SKA1	220134	7.59	16.39	5.88	7.25	0.86	0.00	0.00
SKA2	348235	6.37	11.5	3.5	5.47	1.00	0.00	0.00
SKA3	221150	4.55	6.69	1.64	3.76	1.41	0.00	0.00
SKIL	6498	0.49	2.51	0.41	0.57	1.51	0.00	0.00
SLC10A7	84068	1.03	3.61	0.98	1.45	0.94	0.00	0.00
SLC16A1	6566	4.78	1.38	1.72	2.41	0.99	0.00	0.00
SLC16A6	9120	0.4	2.39	0.52	1.2	0.67	0.00	0.00
SLC1A2	6506	0.91	3.99	1.31	1.48	1.07	0.00	0.00
SLC22A11	55867	0.02	0	0	0	0.75	0.00	0.00
SLC22A18AS	5003	4.33	1.69	1.47	2.55	0.58	0.00	0.00
SLC25A10	1468	237.62	86.11	97.6	111.95	0.62	0.00	0.00

SLC25A11	8402	104.63	51.17	48.57	44.23	0.75	0.00	0.00
SLC25A18	83733	3.94	1.11	1.02	1.42	0.90	0.00	0.00
SLC25A24	29957	0	0	0	0	0.84	0.00	0.00
SLC25A36	55186	0.91	5.31	1.41	2.14	0.81	0.00	0.00
SLC25A46	91137	1.34	4.23	1.57	2.05	0.76	0.00	0.00
SLC26A2	1836	0.21	1.78	0.3	0.63	1.09	0.00	0.00
SLC2A13	114134	21.87	17.37	16.03	15.67	0.91	0.00	0.00
SLC2A2	6514	2.13	6.94	1.84	3.67	0.74	0.00	0.00
SLC2A3	6515	41.08	138.72	64.78	36.19	0.82	0.00	0.00
SLC2A4RG	56731	141.52	47.55	59.98	65.81	0.58	0.00	0.00
SLC2A6	11182	28.54	7.98	10.47	13.27	0.62	0.00	0.00
SLC30A3	7781	5.72	5.54	4.57	1.74	0.83	0.00	0.00
SLC30A9	10463	3.04	11.95	2.25	3.76	1.27	0.00	0.00
SLC35A1	10559	1.8	5.71	1.98	1.67	1.14	0.00	0.00
SLC35A3	23443	0.5	1.4	0.32	0.63	0.90	0.00	0.00
SLC35B3	51000	2.76	7.48	3.59	3.48	0.62	0.00	0.00
SLC38A1	81539	4.77	18	5.8	8.09	0.70	0.00	0.00
SLC38A2	54407	6.92	60.43	21.07	22.79	0.62	0.00	0.00
SLC38A4	55089	0.15	0.66	0.12	0.1	1.77	0.00	0.00
SLC39A10	57181	0.83	6.74	1.23	1.75	1.33	0.00	0.00
SLC39A3	29985	0.55	0.31	0.33	0.31	0.68	0.00	0.00
SLC39A6	25800	2.3	13.15	2.79	3.74	1.21	0.00	0.00
SLC39A8	64116	0.2	0.65	0.06	0.36	1.26	0.00	0.00
SLC44A2	57153	1.08	1.84	1.15	0.5	0.94	0.00	0.00
SLC44A5	204962	1.04	1.88	0.62	1.04	0.91	0.00	0.00
SLC46A1	113235	10.44	7.34	5.18	5.8	0.70	0.00	0.00
SLC4A1AP	22950	3.87	10.41	3.15	4.73	0.85	0.00	0.00
SLC4A7	9497	0.43	1.58	0.4	0.57	1.13	0.00	0.00
SLC5A3	6526	0.13	2.18	0.46	0.53	1.21	0.00	0.00
SLC6A13	6540	0.49	0.17	0	0.02	5.55	0.00	0.00
SLC7A10	56301	22.18	17.08	7.81	6.16	1.49	0.00	0.00
SLF1	84250	0.11	1.1	0.09	0.22	1.83	0.00	0.00
SLF2	55719	1.85	9.67	2.54	2.78	1.30	0.00	0.00

SLK	9748	0.81	2.66	0.88	1.03	1.30	0.00	0.00
SLMAP	7871	0.71	5.25	1.4	2.55	0.65	0.00	0.00
SLMO2-ATP5E	100533975	0.05	0.34	0	0	6.42	0.00	0.00
SLU7	10569	1.57	6.92	2.29	2.88	0.65	0.00	0.00
SMAD5	4090	1.67	4.98	1.7	2.3	0.93	0.00	0.00
SMARCA1	6594	21.62	15.24	21.79	18.12	0.96	0.00	0.00
SMARCA5	8467	1.41	13.8	2.06	4.06	1.31	0.00	0.00
SMARCA5-AS1	100128055	0.75	2.01	0.51	0.96	0.89	0.00	0.00
SMARCAD1	56916	15.76	26.73	23.4	21.81	0.81	0.00	0.00
SMC2	10592	0.5	2.66	0.46	1.26	0.89	0.00	0.00
SMC3	9126	0.88	6.15	0.99	2.31	1.08	0.00	0.00
SMC4	10051	1.93	6.57	0.99	2.19	1.43	0.00	0.00
SMC5	23137	0.38	2.38	0.5	1	0.82	0.00	0.00
SMC6	79677	2.18	4.4	1.03	1.91	1.18	0.00	0.00
SMCHD1	23347	0.35	3.97	0.7	1.54	0.85	0.00	0.00
SMG1	23049	0.43	6.26	1.21	1.78	1.17	0.00	0.00
SMIM10L1	100129361	6.28	4.09	2.4	2.7	0.66	0.00	0.00
SMIM13	221710	3.53	6.95	2.91	3.77	0.71	0.00	0.00
SMIM15	643155	6.45	11.54	3.55	6.32	0.89	0.00	0.00
SMIM30	401397	8.98	10.59	5.08	5.04	0.96	0.00	0.00
SMN1	6606	5.03	9.79	4.63	4.75	0.66	0.00	0.00
SMN2	6607	10.97	21.14	11.03	9.17	0.69	0.00	0.00
SMO	6608	0	0	0	0	0.94	0.00	0.00
SMPD2	6610	0	0	0	0.05	0.70	0.00	0.00
SMTNL1	219537	0	0	0	0	3.45	0.00	0.00
SMYD4	114826	2.48	7.01	3.01	2.91	0.60	0.00	0.00
SNAPC1	6617	0.62	2.64	0.89	1.07	0.83	0.00	0.00
SNHG9	735301	2.81	0.85	0.79	0.88	1.15	0.00	0.00
SNRNP48	154007	2.21	5.88	1.94	2.3	0.94	0.00	0.00
SNRPD2	6633	6.97	4.72	3.21	3.74	0.77	0.00	0.00
SNTB1	6641	2.59	7.58	3.5	4.56	1.26	0.00	0.00
SNW1	22938	9.38	25.58	9.38	10.38	0.85	0.00	0.00
SNX10	29887	3.2	9.07	1.74	3.8	1.13	0.00	0.00

SNX13	23161	0.69	2.5	0.61	1.19	0.91	0.00	0.00
SNX14	57231	9.37	15.14	5.73	9.4	0.69	0.00	0.00
SNX2	6643	1.01	3.98	0.03	2.51	1.59	0.00	0.00
SNX4	8723	3.72	8.14	1.72	3.25	1.24	0.00	0.00
SOCS4	122809	0.24	1.31	0.32	0.46	0.92	0.00	0.00
SOCS5	9655	0.93	3.88	0.9	1.81	0.84	0.00	0.00
SOCS6	9306	1.08	3.56	1.08	1.58	0.80	0.00	0.00
SORCS2	57537	0.17	0.03	0.04	0	3.04	0.00	0.00
SP3	6670	2.33	12.14	2.37	4.1	1.02	0.00	0.00
SP4	6671	0.16	0.78	0.13	0.18	1.37	0.00	0.00
SPAAR	158376	0.62	1.23	0.52	0.21	1.34	0.00	0.00
SPAG16	79582	5.15	7.86	3.01	3.12	0.85	0.00	0.00
SPAG9	9043	1.3	11.1	3.32	3.46	0.80	0.00	0.00
SPART	23111	2.48	8.04	1.76	2.72	1.25	0.00	0.00
SPAST	6683	1.16	3.69	0.95	2	0.73	0.00	0.00
SPATA2L	124044	43.28	13.3	13.67	15.9	0.95	0.00	0.00
SPATA33	124045	15.36	8.46	6.24	5.99	0.95	0.00	0.00
SPATA9	83890	0.15	0.77	0.18	0.16	1.31	0.00	0.00
SPC24	147841	23.5	16.06	12.06	9.38	0.80	0.00	0.00
SPC25	57405	6.65	11.53	4.29	5.1	0.93	0.00	0.00
SPCS3	60559	0	0	0	0.06	0.59	0.00	0.00
SPDL1	54908	1.32	6.08	1.08	1.49	1.45	0.00	0.00
SPECC1L	23384	0	0.03	0.01	0	1.05	0.00	0.00
SPICE1	152185	0.43	1.44	0.31	0.39	1.35	0.00	0.00
SPINDOC	144097	71.85	29.06	28.34	29.45	0.76	0.00	0.00
SPINK1	6690	12.27	12.8	5.73	6.79	0.95	0.00	0.00
SPOPL	339745	0.72	3.38	0.66	1.58	0.89	0.00	0.00
SPRTN	83932	1.13	2.57	0.69	1.25	0.96	0.00	0.00
SPTLC1	10558	10.98	21.44	8.23	11.32	0.72	0.00	0.00
SPTY2D1	144108	0.97	3.87	0.95	1.23	1.13	0.00	0.00
SPTY2D1OS	100506540	0.64	0.89	0.49	0.34	0.82	0.00	0.00
SRBD1	55133	0.7	5.48	1.18	1.27	1.37	0.00	0.00
SREK1	140890	0.61	3.72	0.65	1.47	0.89	0.00	0.00

SREK1IP1	285672	0.73	2.01	0.39	0.8	1.39	0.00	0.00
SRFBP1	153443	0.78	2.1	0.24	0.48	0.98	0.00	0.00
SRGAP2B	647135	0.33	1.23	0.58	0.38	0.59	0.00	0.00
SRM	6723	864.73	282.84	332.87	347.71	0.77	0.00	0.00
SRP19	6728	15.53	18.72	8.05	11.89	0.78	0.00	0.00
SRP54	6729	8.34	19.5	6.32	10.22	0.73	0.00	0.00
SRP72	6731	0	0	0.05	0.05	0.93	0.00	0.00
SRP9	6726	76.85	97.04	46.44	63.6	0.65	0.00	0.00
SRPK1	6732	0	0	0.02	0	0.88	0.00	0.00
SRR	63826	12.71	30.74	14.39	13.36	0.68	0.00	0.00
SRSF1	6426	28.94	76.94	24.3	33.68	0.91	0.00	0.00
SRSF10	10772	6.87	28.09	7.4	10.06	1.09	0.00	0.00
SRSF11	9295	4.1	17.09	3.57	6.17	1.09	0.00	0.00
SRSF7	6432	42.58	96.11	47.28	43.41	0.59	0.00	0.00
SS18	6760	3.97	12.89	5.15	6.08	0.61	0.00	0.00
SSB	6741	0.12	0	0	0	1.37	0.00	0.00
SSSCA1	10534	56.09	22.31	25.01	18	0.94	0.00	0.00
SSTR3	6753	5.86	1.86	0.65	1.85	1.68	0.00	0.00
SSTR5	6755	31.73	15.13	9.9	11.91	1.11	0.00	0.00
SSX2IP	117178	0.62	3.1	0.56	1.17	1.05	0.00	0.00
ST6GAL2	84620	0.23	0.09	0.05	0.02	2.08	0.00	0.00
STAG1	10274	0.12	2.49	0.23	0.54	1.88	0.00	0.00
STAG2	10735	1.18	5.68	0.99	1.55	1.37	0.00	0.00
STAM2	10254	1.15	4.31	1.08	1.52	0.83	0.00	0.00
STAU2	27067	1.64	5.14	2.02	2.79	0.60	0.00	0.00
STEAP1	26872	10.54	15.08	6.35	10.66	0.61	0.00	0.00
STIL	6491	15.4	8.84	10.68	11.42	0.97	0.00	0.00
STK17B	9262	1.16	1.45	0.29	0.4	1.61	0.00	0.00
STK26	51765	4.53	17.54	4.34	6.99	0.94	0.00	0.00
STK4	6789	2.75	14.21	5.66	6.92	0.64	0.00	0.00
STOML1	9399	5.49	2.53	3.24	2.18	0.63	0.00	0.00
STRBP	55342	0	0.01	0	0	0.66	0.00	0.00
STRIP2	57464	0.58	1.4	0.41	0.48	1.08	0.00	0.00

STRN	6801	0.66	3.15	0.51	1.07	1.02	0.00	0.00
STT3B	201595	11.23	48.31	15.09	19.13	0.81	0.00	0.00
STX5	6811	38.98	13.28	16.32	17.97	0.62	0.00	0.00
STXBP3	6814	1.43	2.66	0.73	1.9	0.64	0.00	0.00
STXBP5-AS1	729178	0.09	0.51	0.15	0.12	1.29	0.00	0.00
SUB1	10923	89.88	67.8	47.88	60.41	0.61	0.00	0.00
SUCLA2	8803	3.5	11.05	1.98	4.28	1.21	0.00	0.00
SUCO	51430	0.89	4.45	1.18	1.65	0.92	0.00	0.00
SUGT1	10910	5.19	11.87	2.65	4.23	1.19	0.00	0.00
SUPT16H	11198	7.36	31.41	9.2	9.99	1.00	0.00	0.00
SUV39H2	79723	2.83	6.19	1.76	2.59	1.07	0.00	0.00
SUZ12	23512	1.66	5.88	0.97	2.71	1.09	0.00	0.00
SVIP	258010	3.03	7.2	2.4	3.94	1.01	0.00	0.00
SWAP70	23075	0.54	2.96	0.93	1.12	0.63	0.00	0.00
SYCE1L	100130958	1.14	1.35	0.53	0.44	1.34	0.00	0.00
SYDE1	85360	11.6	9.13	5.75	3.69	1.10	0.00	0.00
SYNE1	23345	0.35	2.55	0.72	1.39	0.74	0.00	0.00
SYNJ1	8867	0.1	0.93	0.22	0.42	0.69	0.00	0.00
SYNJ2BP-COX16	100529257	0.76	0.98	0.61	0.19	1.44	0.00	0.00
SYNM	23336	0.43	1.56	0.57	0.58	0.76	0.00	0.00
SYNPO	11346	3.84	3.31	1.48	2.02	0.98	0.00	0.00
SYP	6855	10.72	5.68	3.93	3.61	1.06	0.00	0.00
SYTL1	84958	18.97	4.47	12.82	4.99	0.76	0.00	0.00
SYTL2	54843	1.17	9.57	1.89	2.18	1.45	0.00	0.00
SYTL5	94122	0.08	0.6	0.08	0.09	2.40	0.00	0.00
TACR2	6865	6.85	4.77	3.15	2.76	0.90	0.00	0.00
TAF13	6884	7.34	10.81	3.38	5.55	0.90	0.00	0.00
TAF1B	9014	0	0	0	0	0.97	0.00	0.00
TAF1D	79101	7	14.77	5.77	8.54	0.75	0.00	0.00
TAF2	6873	1.02	4.84	1.03	2.03	0.94	0.00	0.00
TAF5	6877	0.51	2.65	0.64	0.95	0.84	0.00	0.00
TAF9B	51616	2.43	4.25	1.51	2.39	0.61	0.00	0.00
TANK	10010	6.22	14.16	4.6	7.16	0.78	0.00	0.00

TAOK1	57551	0.15	3.41	0.73	1.03	1.01	0.00	0.00
TARBP2	6895	51.35	20.76	24.39	19.84	0.59	0.00	0.00
TARDBP	23435	15.55	38.89	13.47	16.32	0.76	0.00	0.00
TARS	6897	12.26	29.86	11.75	13.76	0.74	0.00	0.00
TARSL2	123283	0.74	3.04	0.82	1.34	0.76	0.00	0.00
TAS1R3	83756	3.2	1.1	1.06	1.33	0.86	0.00	0.00
TASP1	55617	2.44	8.36	3.08	3.65	0.58	0.00	0.00
TAX1BP1	8887	6.76	18.83	4.42	7.82	1.06	0.00	0.00
TBC1D12	23232	0.48	2.31	0.6	1.01	0.71	0.00	0.00
TBC1D13	54662	30.16	16.53	13.62	16.15	0.66	0.00	0.00
TBC1D15	64786	1.26	2.68	0.48	1.11	1.13	0.00	0.00
TBC1D19	55296	0.21	0.79	0.35	0.22	1.21	0.00	0.00
TBC1D23	55773	0.83	4.65	0.98	1.57	0.94	0.00	0.00
TBC1D31	93594	0.52	1.82	0.22	0.46	1.79	0.00	0.00
TBC1D3P1-DHX40P1	653645	0.89	2.29	0	1.48	0.93	0.00	0.00
TBC1D8B	54885	0.45	2.33	0.39	0.6	1.34	0.00	0.00
TBCEL	219899	0.17	0.58	0.22	0.24	0.96	0.00	0.00
TBK1	29110	0.67	1.3	0.29	0.58	1.16	0.00	0.00
TBL1XR1	79718	1.32	5.28	1.69	1.94	0.85	0.00	0.00
TBL1Y	90665	0.4	0.37	0.08	0.21	1.46	0.00	0.00
TBRG4	9238	0.05	0.16	0.03	0.04	0.72	0.00	0.00
TBX4	9496	2.29	6.36	1.96	1.73	1.08	0.00	0.00
TCEAL4	79921	6.73	10.61	4.94	5.51	0.73	0.00	0.00
TCEAL9	51186	4.51	8.41	1.15	2.03	1.98	0.00	0.00
TCERG1	10915	3.36	13.9	4.82	6.32	0.63	0.00	0.00
TCF12	6938	1.5	10.11	2.66	3.97	0.77	0.00	0.00
TCIM	56892	3.23	10.16	3.73	3.03	1.01	0.00	0.00
TCTE3	6991	2.7	2.36	1.73	1.46	0.67	0.00	0.00
TDG	6996	2.35	7.66	2.28	3.01	0.98	0.00	0.00
TDP2	51567	11.77	29.55	10.46	16.18	0.68	0.00	0.00
TDRD3	81550	0.46	1.82	0.41	1.25	0.85	0.00	0.00
TDRD7	23424	1.27	5.73	2.24	2.27	0.68	0.00	0.00
TEAD1	7003	0.53	5.4	0.87	1.64	1.23	0.00	0.00

TEDC1	283643	17.61	8.71	7.25	5.99	1.02	0.00	0.00
TEDC2	80178	2.91	7.69	1.06	1.97	1.02	0.00	0.00
TEFM	79736	0	0	0	0	0.96	0.00	0.00
TEKT4P2	100132288	20.31	11.04	10.94	9.08	0.67	0.00	0.00
TEN1	100134934	38.59	13.21	17.69	15.83	0.64	0.00	0.00
TENM3	55714	0.47	4.58	0.42	0.63	2.22	0.00	0.00
TENM4	26011	0	0.06	0	0	5.20	0.00	0.00
TENT4B	64282	1.72	5.91	1.42	3.27	0.71	0.00	0.00
TENT5A	55603	1.33	5.01	1.41	2.42	0.72	0.00	0.00
TENT5B	115572	0.4	0.38	0.06	0.13	2.04	0.00	0.00
TERF1	7013	2.1	3.6	1.16	1.33	1.00	0.00	0.00
TET1	80312	0.23	1.03	0.26	0.35	0.98	0.00	0.00
TET2	54790	0.21	1.15	0.19	0.4	1.18	0.00	0.00
TEX15	56154	0.04	0.15	0.02	0	4.35	0.00	0.00
TEX9	374618	0.44	0.75	0.11	0.18	1.53	0.00	0.00
TFAM	7019	7.38	15.6	2.63	4.34	1.17	0.00	0.00
TFPI	7035	70.69	152.74	62.68	72.34	0.61	0.00	0.00
TFRC	7037	15.53	69	23.64	25.86	0.80	0.00	0.00
TGDS	23483	3.48	5.69	2.59	2.95	0.71	0.00	0.00
TGFB3	7043	0.71	1.44	0.93	0.54	1.06	0.00	0.00
TGFBR1	7046	6.58	9.06	8.78	10.13	0.59	0.00	0.00
TGFBR3	7049	2.24	21.74	7.04	8.08	0.64	0.00	0.00
TGFBR3L	100507588	5.82	2.26	2.51	1.96	0.90	0.00	0.00
TGS1	96764	1.44	5.45	1.5	1.79	0.95	0.00	0.00
THAP1	55145	2.17	3.12	0.76	1.55	1.19	0.00	0.00
THAP12	5612	2.75	11.3	2.94	4.23	0.97	0.00	0.00
THAP5	168451	0.88	2.2	0.43	1.03	1.03	0.00	0.00
THAP9	79725	0.22	0.65	0.21	0.31	1.11	0.00	0.00
THBS1	7057	3.02	13.69	5.82	4.92	0.64	0.00	0.00
THEM4	117145	4.3	8.87	4.43	3.78	0.59	0.00	0.00
THEM6	51337	74.99	24.77	33.02	29.41	0.68	0.00	0.00
THNSL1	79896	0.33	2.42	0.76	0.87	0.75	0.00	0.00
THOC1	9984	0.63	2.62	0.6	0.61	1.10	0.00	0.00

THOC2	57187	0.92	4.88	1.03	1.86	0.97	0.00	0.00
THOP1	7064	130.76	70.7	58.93	57.92	0.79	0.00	0.00
THSD4	79875	8.04	13.69	4.99	6.31	0.91	0.00	0.00
THSD4-AS1	101929196	3.09	7.55	3.32	3.67	0.60	0.00	0.00
THUMPD1	55623	1.5	7.59	1.56	2.44	1.16	0.00	0.00
THUMPD2	80745	1.72	5.2	1.37	1.73	1.09	0.00	0.00
THUMPD3	25917	2.62	6.96	2.67	3.41	0.63	0.00	0.00
THUMPD3-AS1	440944	0.12	0.44	0.13	0.17	0.89	0.00	0.00
TIA1	7072	3.03	17.12	3.18	5.74	1.26	0.00	0.00
TIFA	92610	1.52	3.58	1.42	1.65	0.74	0.00	0.00
TIGD2	166815	0.98	4.4	1.12	2.31	0.67	0.00	0.00
TIGD5	84948	9.83	3.61	4.56	3.86	0.69	0.00	0.00
TIMM10	26519	88.73	38.53	40.46	43.45	0.60	0.00	0.00
TIMM13	26517	110.94	43.15	39.96	45.21	0.86	0.00	0.00
TIMM21	29090	4.07	12.88	4.7	5.97	0.63	0.00	0.00
TIMM8B	26521	60.9	25.76	21.89	28.93	0.70	0.00	0.00
TIMP4	7079	5.14	3.56	2.13	1.21	1.25	0.00	0.00
TIPIN	54962	6.57	9.55	4.65	4.73	0.87	0.00	0.00
TK1	7083	170.52	113.27	86.23	89.66	0.69	0.00	0.00
TLK1	9874	1.72	11.88	2.45	5.18	0.78	0.00	0.00
TM9SF3	56889	0	0.02	0	0	0.63	0.00	0.00
TMA16	55319	4.39	10.84	4.79	5.23	0.63	0.00	0.00
TMCC1	23023	2.15	9.47	4.03	3.3	0.73	0.00	0.00
TMED1	11018	66.84	34.06	38.55	32.77	0.62	0.00	0.00
TMED3	23423	192.05	87.88	93.56	87.97	0.63	0.00	0.00
TMED7	51014	9.03	19.43	5.86	9.33	0.90	0.00	0.00
TMED8	283578	0.96	4.19	1.32	1.85	0.66	0.00	0.00
TMEM117	84216	1.06	1.87	0.53	1.23	0.73	0.00	0.00
TMEM126A	84233	18.7	14.52	12.12	10.29	0.59	0.00	0.00
TMEM126B	55863	15.79	17.88	6.96	8.81	1.06	0.00	0.00
TMEM161B	153396	1.64	3.59	1.36	1.55	0.79	0.00	0.00
TMEM167A	153339	5.56	16.05	4.97	6.97	0.85	0.00	0.00
TMEM168	64418	0.5	1.62	0.65	0.71	0.65	0.00	0.00

TMEM170B	100113407	0.33	0.77	0.28	0.43	0.78	0.00	0.00
TMEM181	57583	1.79	12.97	4.06	4.86	0.75	0.00	0.00
TMEM19	55266	0	0.09	0.08	0	0.60	0.00	0.00
TMEM205	374882	192.13	68.31	85.04	73.12	0.72	0.00	0.00
TMEM222	84065	82.21	20.37	32.6	33.2	0.65	0.00	0.00
TMEM250	90120	47.68	16.3	21.3	21.08	0.60	0.00	0.00
TMEM263	90488	4.85	16.67	4.4	6.93	0.92	0.00	0.00
TMEM30A	55754	2.79	19.58	6.19	8.58	0.61	0.00	0.00
TMEM52	339456	51.56	18.92	24.95	21.36	0.60	0.00	0.00
TMEM87A	25963	4.02	6.8	2.43	2.92	0.81	0.00	0.00
TMF1	7110	0.88	2.57	1.75	0.84	0.76	0.00	0.00
TMOD1	7111	0.01	0.28	0.06	0.01	1.72	0.00	0.00
TMOD3	29766	1.39	5.61	1.88	2.69	0.58	0.00	0.00
TMPO	7112	16.12	40.62	14.34	18.15	0.86	0.00	0.00
TMPO-AS1	100128191	0	0.1	0	0	4.80	0.00	0.00
TMTC3	160418	0.58	1.35	0.36	0.55	1.08	0.00	0.00
TMX1	81542	2.82	9.3	1.89	1.83	1.52	0.00	0.00
TMX3	54495	0.46	2.27	0.55	0.88	1.14	0.00	0.00
TNFAIP8	25816	1.61	3.39	1.49	1.59	0.70	0.00	0.00
TNFRSF14-AS1	115110	23.05	8.41	7.5	10.26	0.64	0.00	0.00
TNKS2	80351	0.83	5.97	1.62	2.7	0.62	0.00	0.00
TNPO1	3842	6.36	15.74	6.33	7.04	0.86	0.00	0.00
TNRC6A	27327	1.09	6	1.42	1.94	1.06	0.00	0.00
TNS1	7145	0.59	6.19	3.41	0.55	0.76	0.00	0.00
TOGARAM1	23116	0.21	0.66	0.21	0.26	0.91	0.00	0.00
TOP1	7150	8.4	36.15	10.9	15.72	0.72	0.00	0.00
TOP2A	7153	3.61	27.75	4.46	6.97	1.44	0.00	0.00
TOP2B	7155	1.72	13.14	2.38	4.26	1.15	0.00	0.00
TOPBP1	11073	1.02	7.64	1.16	2.45	1.24	0.00	0.00
TOPORS	10210	0.96	2.31	0.39	0.55	1.78	0.00	0.00
TOR1AIP1	26092	2.45	9.81	3.69	4.47	0.60	0.00	0.00
TPI1	7167	1254.53	586	623.01	571.56	0.63	0.00	0.00
TPMT	7172	7.05	11.22	3.32	6.06	0.90	0.00	0.00

TPP2	7174	2.28	9.45	3.04	3.09	1.19	0.00	0.00
TPR	7175	0.6	3.57	0.84	1.79	0.66	0.00	0.00
TPRKB	51002	16.34	14.82	6.48	7.59	1.13	0.00	0.00
TRA2A	29896	7.56	20.34	6.6	7.91	0.95	0.00	0.00
TRA2B	6434	47.08	74.69	37.91	39.4	0.67	0.00	0.00
TRAF3IP1	26146	0.96	3.7	1.65	1.78	0.64	0.00	0.00
TRAF4	9618	106.17	30.37	40.64	39.76	0.76	0.00	0.00
TRAM1	23471	0	0.08	0.04	0.04	0.66	0.00	0.00
TRAPPC11	60684	1.31	5.38	1.19	2.33	0.90	0.00	0.00
TRAPPC2L	51693	64.96	22.62	23.06	30.19	0.76	0.00	0.00
TRAPPC5	126003	224.88	51.21	54.92	54.98	1.33	0.00	0.00
TRAPPC6B	122553	1.5	3.69	1.31	1.55	0.84	0.00	0.00
TRDMT1	1787	0.16	0.52	0.2	0.13	1.34	0.00	0.00
TRIM24	8805	11.17	32.88	8.05	15.26	0.92	0.00	0.00
TRIM37	4591	5.77	16.87	4.57	7.23	0.93	0.00	0.00
TRIM39-RPP21	202658	0.77	0.27	0	0	5.83	0.00	0.00
TRIM59-IFT80	100174949	0.02	0.21	0	0.03	2.61	0.00	0.00
TRIP12	9320	3.46	19.47	6.31	7.62	0.68	0.00	0.00
TRIQK	286144	2.92	5.76	1.75	3.72	0.78	0.00	0.00
TRMT10A	93587	0.42	1.7	0.23	0.4	0.96	0.00	0.00
TRMT10C	54931	3.63	9.61	1.85	2.97	1.45	0.00	0.00
TRMT11	60487	0.23	0.09	0.15	0.39	0.89	0.00	0.00
TRMT13	54482	0.5	1.71	0.23	0.93	1.21	0.00	0.00
TRMT1L	81627	0.73	3.09	0.61	1.07	1.15	0.00	0.00
TRMT2A	27037	29.99	12.9	12.78	12.18	0.76	0.00	0.00
TRMT5	57570	1.64	4.13	1.51	2.06	0.75	0.00	0.00
TRMT6	51605	52.97	39.47	45.02	42.72	0.62	0.00	0.00
TRMT61A	115708	37.62	11.1	12.41	13.71	0.97	0.00	0.00
TRNAU1AP	54952	3.87	0.06	0.02	0.49	2.56	0.00	0.00
TRNT1	51095	0.04	0	0	0	0.90	0.00	0.00
TRPC7	57113	0.05	0.63	0	0	7.43	0.00	0.00
TRPM2	7226	6.72	3.09	2.52	3.46	0.68	0.00	0.00
TRPM7	54822	0.52	2.57	0.46	1.06	0.93	0.00	0.00

TSNAX	7257	0.66	5.13	0.46	0.57	2.59	0.00	0.00
TSPAN17	26262	30.77	11.76	13.49	12.6	0.69	0.00	0.00
TSSC4	10078	34.95	12.64	15.15	15.04	0.68	0.00	0.00
TTC1	7265	16.52	27.7	13.1	14.75	0.67	0.00	0.00
TTC14	151613	2.29	5.79	1.23	2.37	1.23	0.00	0.00
TTC16	158248	11.08	3.65	1.73	0.85	2.58	0.00	0.00
TTC21B	79809	0.72	5.14	1.04	2.09	0.91	0.00	0.00
TTC23	64927	1.48	3.86	1.62	1.78	0.61	0.00	0.00
TTC28	23331	0.77	2.61	0.87	1.26	0.64	0.00	0.00
TTC3	7267	2.77	19.95	5.34	7.64	0.79	0.00	0.00
TTC30A	92104	0.55	1.44	0.44	0.31	1.41	0.00	0.00
TTC33	23548	1.1	3.79	0.77	1.22	1.15	0.00	0.00
TTC37	9652	0.72	5.55	0.97	1.73	1.20	0.00	0.00
TTC41P	253724	0.11	0.1	0.01	0.04	2.24	0.00	0.00
TTF1	7270	0.61	3.11	0.54	1.35	0.99	0.00	0.00
TTK	7272	2.64	4.96	0.57	1.18	2.00	0.00	0.00
TTLL10	254173	69.74	64.17	15.49	32.72	1.47	0.00	0.00
TTPA	7274	2.51	2.81	1.12	1.82	0.77	0.00	0.00
TTTY10	246119	0.01	0.05	0	0.02	1.73	0.00	0.00
TUBA3D	113457	0	0.45	0	0	5.25	0.00	0.00
TUBA4B	80086	2.67	1.91	1	1.58	0.83	0.00	0.00
TUBB8P12	260334	0.44	1.19	0.45	0.27	1.34	0.00	0.00
TUBD1	51174	1.02	5.23	1.42	1.56	1.13	0.00	0.00
TUT7	79670	0.31	1.8	0.33	0.7	1.01	0.00	0.00
TVP23B	51030	4.08	10.71	3.79	4.6	0.84	0.00	0.00
TWF1	5756	5.67	14.01	2.79	5.96	1.15	0.00	0.00
TWISTNB	221830	0.86	3.65	0.75	1.44	1.03	0.00	0.00
TWSG1	57045	0.56	1.73	0.54	0.64	0.95	0.00	0.00
TXLNG	55787	0.05	0	0.03	0.14	0.70	0.00	0.00
TXLNGY	246126	1.02	2.46	0.78	1.92	0.63	0.00	0.00
TXNDC16	57544	0.38	2.14	0.59	0.9	0.72	0.00	0.00
TXNDC9	10190	19.98	18.87	7.99	11.95	0.97	0.00	0.00
U2SURP	23350	2.95	10.67	2.29	4.05	1.12	0.00	0.00

UACA	55075	0	0.01	0	0	1.76	0.00	0.00
UAP1	6675	10.76	31.53	13.25	13.77	0.59	0.00	0.00
UBA3	9039	4.48	14.32	5.11	7	0.63	0.00	0.00
UBA5	79876	2.79	8.64	2.61	4.82	0.65	0.00	0.00
UBA52	7311	963.04	372.27	426.61	376.34	0.62	0.00	0.00
UBA6	55236	0.7	4.27	0.66	1.32	1.11	0.00	0.00
UBAC2-AS1	100289373	16.09	14.02	10.09	10.39	0.59	0.00	0.00
UBE2D1	7321	3.08	5	2.44	2.73	0.62	0.00	0.00
UBE2Q2L	100505679	0.09	1.08	0.83	0.77	1.53	0.00	0.00
UBE2V2	7336	8.58	19.13	6.79	9.61	0.79	0.00	0.00
UBE3A	7337	2.95	11.22	3.4	4.43	0.86	0.00	0.00
UBLCP1	134510	0.98	4.43	0.87	1.66	1.09	0.00	0.00
UBN2	254048	0.13	0.64	0.2	0.28	0.77	0.00	0.00
UBR1	197131	4.64	2.97	3.18	4.44	1.35	0.00	0.00
UBR2	23304	0.77	4.79	1.18	1.84	0.97	0.00	0.00
UBR3	130507	0.47	3.11	0.83	1.16	1.11	0.00	0.00
UBR5	51366	0.89	7.49	2.6	3.23	0.64	0.00	0.00
UBXN11	91544	22.34	11.47	9.51	8.97	0.95	0.00	0.00
UBXN2A	165324	2.35	9.85	3.36	3.85	0.62	0.00	0.00
UBXN2B	137886	0.88	2.56	1.17	1.39	0.59	0.00	0.00
UBXN7	26043	0.8	3.4	0.92	0.75	1.26	0.00	0.00
UBXN7-AS1	100874034	0	0	0	0	3.25	0.00	0.00
UCHL5	51377	7.83	14.21	4.62	6.32	0.94	0.00	0.00
UCP2	7351	119.31	50.6	44.12	34.04	1.12	0.00	0.00
UFL1	23376	1.2	4.31	0.81	1.78	1.08	0.00	0.00
UGGT2	55757	0.88	3.72	0.98	1.44	0.90	0.00	0.00
UGP2	7360	27.52	88.96	19.03	24.42	1.42	0.00	0.00
UGT1A4	54657	0.41	0.13	0	0.1	2.53	0.00	0.00
UGT2A3	79799	16.74	33.32	9.59	15.19	1.01	0.00	0.00
UGT2B10	7365	0.03	0	0	0	0.81	0.00	0.00
UHMK1	127933	0.58	3.28	0.84	1.6	0.67	0.00	0.00
UHRF1BP1L	23074	0.74	3.09	0.75	1.86	0.63	0.00	0.00
UHRF2	115426	15.98	7.78	8.95	7.44	0.88	0.00	0.00

UMAD1	729852	4.7	7.34	2.27	3.81	0.83	0.00	0.00
UNC13C	440279	0.16	0.63	0.21	0.29	0.61	0.00	0.00
UNC5B	219699	0.98	1.97	0.55	0.09	2.12	0.00	0.00
UNC5D	137970	2.8	8.33	3.27	3.83	0.72	0.00	0.00
UPF2	26019	0.79	4.08	0.68	1.29	1.29	0.00	0.00
UPF3A	65110	13.73	26.91	12.33	13.69	0.64	0.00	0.00
UPF3B	65109	1.08	1.86	0.54	0.82	1.01	0.00	0.00
UQCC3	790955	17.59	6.9	7.49	6.19	0.85	0.00	0.00
UQCRB	7381	6.35	4.24	2.78	1.99	0.73	0.00	0.00
URGCP-MRPS24	100534592	2.58	7.69	3.81	5.65	2.17	0.00	0.00
URI1	8725	0	0	0	0	0.74	0.00	0.00
UROD	7389	194.5	90.03	88.09	94.42	0.65	0.00	0.00
USO1	8615	3.18	15.24	4.53	7.06	0.75	0.00	0.00
USP1	7398	0	0	0.03	0	1.37	0.00	0.00
USP12	219333	0.46	3.08	0.75	1.13	0.86	0.00	0.00
USP12-AS1	100874070	0.64	3.48	0.95	0.72	1.29	0.00	0.00
USP15	9958	1.04	4.24	1.24	1.3	1.12	0.00	0.00
USP16	10600	25.06	71.46	26.96	24.56	0.92	0.00	0.00
USP24	23358	0.89	6.46	1.39	2.47	0.98	0.00	0.00
USP25	29761	0.75	5.18	1.4	2.29	0.70	0.00	0.00
USP33	23032	1.46	5.69	1.35	2.38	0.83	0.00	0.00
USP34	9736	0.84	7.41	1.68	2.81	1.06	0.00	0.00
USP3-AS1	100130855	1.67	3.2	0.51	1.49	1.16	0.00	0.00
USP45	85015	1.3	2.31	0.57	0.71	0.94	0.00	0.00
USP46	64854	0.68	2.46	0.36	0.77	1.25	0.00	0.00
USP47	55031	0.49	2.75	0.54	0.74	1.13	0.00	0.00
USP48	84196	4.11	28.03	7.68	10.43	0.90	0.00	0.00
USP8	9101	1.02	4.36	1	2.3	0.68	0.00	0.00
USP9X	8239	1.07	8.53	2.3	3.4	0.74	0.00	0.00
USP9Y	8287	0.06	0.81	0.11	0.18	1.54	0.00	0.00
USPL1	10208	0.27	2.73	0.72	0.81	1.02	0.00	0.00
UST-AS1	100128176	0.09	0.29	0	0	5.89	0.00	0.00
UTP14A	10813	1.01	0	0	0	2.32	0.00	0.00

UTP20	27340	0.83	5.03	1.05	1.66	1.06	0.00	0.00
UTP23	84294	0.62	1.71	0.56	0.55	1.04	0.00	0.00
UTP3	57050	3.34	8.19	3.21	4.26	0.62	0.00	0.00
UTRN	7402	0.58	6.2	1.89	3.05	0.75	0.00	0.00
VASH2	79805	0.11	0.73	0.15	0.11	1.63	0.00	0.00
VAX2	25806	7.67	3.68	2.42	3.01	1.00	0.00	0.00
VBP1	7411	8.51	15.08	5.65	7.59	0.82	0.00	0.00
VCAN	1462	0.32	4.34	0.58	0.18	3.17	0.00	0.00
VEZF1	7716	2.45	11.47	4.83	4.76	0.64	0.00	0.00
VEZT	55591	1.17	4.77	1.48	1.93	0.85	0.00	0.00
VIRMA	25962	1.38	6.98	1.94	2.74	0.81	0.00	0.00
VMO1	284013	9.7	6.14	8.65	5.99	0.65	0.00	0.00
VPS13A	23230	0.3	2.01	0.46	0.58	0.87	0.00	0.00
VPS13B	157680	0.67	1.71	0.69	0.89	0.72	0.00	0.00
VPS13C	54832	0.19	1.22	0.27	0.55	0.78	0.00	0.00
VPS26A	9559	4.74	10.94	4.25	6.27	0.63	0.00	0.00
VPS36	51028	1.45	3.84	1.35	1.79	0.72	0.00	0.00
VPS41	27072	145.78	99.31	134.06	127.33	0.78	0.00	0.00
VPS4B	9525	2.26	6.37	2.47	3.15	0.66	0.00	0.00
VPS50	55610	1.02	1.67	0.37	0.35	1.66	0.00	0.00
VPS54	51542	2.85	13.84	4.25	6.91	0.60	0.00	0.00
VRK1	7443	4.89	9.89	2.78	2.86	1.40	0.00	0.00
VRK2	7444	3.12	7.79	2.3	4.42	0.70	0.00	0.00
VTA1	51534	12.32	16.81	4.99	6.75	1.32	0.00	0.00
WAC	51322	4.65	25.69	7.41	10.02	0.81	0.00	0.00
WAC-AS1	220906	0.73	2.68	0.73	1.22	0.80	0.00	0.00
WAPL	23063	0.97	6.47	1.69	2.24	0.82	0.00	0.00
WASHC4	23325	0.71	2.48	0.57	1.04	0.96	0.00	0.00
WBP1	23559	103.53	29.54	46.64	39.99	0.63	0.00	0.00
WBP4	11193	1.66	3.62	1.17	1.43	0.63	0.00	0.00
WDFY1	57590	3.31	16.43	5.21	6.75	0.69	0.00	0.00
WDFY3	23001	0.16	1.56	0.51	0.61	0.66	0.00	0.00
WDHD1	11169	0.5	2.92	0.28	0.62	1.78	0.00	0.00

WDPCP	51057	0.48	1.15	0.41	0.47	0.72	0.00	0.00
WDR18	57418	219.07	98.21	102.22	81.54	0.80	0.00	0.00
WDR19	57728	0.4	2.33	0.95	0.75	0.68	0.00	0.00
WDR34	89891	0.07	0.14	0.08	0	1.09	0.00	0.00
WDR35	57539	0.94	4.77	1.11	1.49	1.11	0.00	0.00
WDR36	134430	1.27	4.54	1.24	2.07	0.70	0.00	0.00
WDR43	23160	10.27	37.21	8.99	14.74	0.97	0.00	0.00
WDR44	54521	0.46	1.42	0.53	0.49	1.03	0.00	0.00
WDR47	22911	0.11	1.31	1.1	0.45	0.95	0.00	0.00
WDR48	57599	1.46	7.46	2.85	2.62	0.68	0.00	0.00
WDR54	84058	2.91	1.41	1.23	0.64	2.02	0.00	0.00
WDR72	256764	0	0	0.08	0.06	2.02	0.00	0.00
WDR75	84128	2.83	14.84	3.87	5.46	0.92	0.00	0.00
WDR76	79968	1.19	2.89	1.01	1.2	0.62	0.00	0.00
WDR89	112840	0.8	1.58	0.42	0.53	1.30	0.00	0.00
WNT8A	7478	0.06	0.6	0.13	0.05	1.91	0.00	0.00
WNT9B	7484	7.47	9.99	5.11	3.92	0.95	0.00	0.00
WRN	7486	0.6	1.93	0.67	0.8	0.85	0.00	0.00
WTIP	126374	12.27	6.33	5.23	3.98	1.01	0.00	0.00
WWC2	80014	0.78	5.12	1.38	1.8	0.79	0.00	0.00
WWP1	11059	1.44	14.3	2.37	3.72	1.42	0.00	0.00
XIAP	331	0.83	3.28	1.15	1.09	0.88	0.00	0.00
XPNPEP2	7512	2.65	0.39	0.48	1.3	0.79	0.00	0.00
XPO1	7514	7.62	50.36	10.9	16.38	1.12	0.00	0.00
XPO4	64328	0.94	4.57	1.06	1.87	0.83	0.00	0.00
XPOT	11260	5.19	25.42	8.55	9.13	0.77	0.00	0.00
XRCC2	7516	0.71	1.77	0.57	0.77	1.08	0.00	0.00
XRCC3	7517	25.69	14.19	10.62	11.34	0.68	0.00	0.00
XRCC4	7518	3.52	4.1	1.33	1.43	1.28	0.00	0.00
XRCC5	7520	75.04	197.53	83.58	103.82	0.60	0.00	0.00
XRN1	54464	0.09	0.75	0.21	0.28	0.70	0.00	0.00
YAE1	57002	8.3	6.57	6.69	6.77	0.67	0.00	0.00
YEATS4	8089	11.91	13.32	5.68	7.17	0.98	0.00	0.00

YES1	7525	1.56	6.45	1.51	2.61	0.99	0.00	0.00
YJU2	55702	50.05	30.69	24.55	23.5	0.75	0.00	0.00
YME1L1	10730	0	0	0	0.02	1.09	0.00	0.00
YTHDC1	91746	2.3	11.76	4.17	4.62	0.72	0.00	0.00
YTHDC2	64848	0.86	4.01	1	1.12	1.22	0.00	0.00
YTHDF3	253943	2.39	9.32	2.71	4.33	0.70	0.00	0.00
ZBED5	58486	0.33	2.67	0.29	0.09	2.96	0.00	0.00
ZBED6	100381270	0.19	0.87	0.19	0.15	1.12	0.00	0.00
ZBTB1	22890	0.7	2.8	0.73	1.43	0.70	0.00	0.00
ZBTB11	27107	0.58	0.74	0.75	0.59	0.64	0.00	0.00
ZBTB2	57621	5.06	12.89	4.82	6.19	0.71	0.00	0.00
ZBTB20	26137	0.03	0.29	0.11	0.12	1.03	0.00	0.00
ZBTB24	9841	0	0	0	0	0.68	0.00	0.00
ZBTB33	10009	0.62	3.7	0.74	1.59	0.88	0.00	0.00
ZBTB34	403341	0.32	1.14	0.48	0.49	0.65	0.00	0.00
ZBTB38	253461	0.63	3.92	1.29	1.52	0.67	0.00	0.00
ZBTB41	360023	0.11	0.29	0.06	0.09	1.39	0.00	0.00
ZBTB6	10773	0.38	1.7	0.28	0.61	1.34	0.00	0.00
ZC3H11A	9877	1.87	11.69	3.82	5.08	0.63	0.00	0.00
ZC3H12C	85463	0.08	0.37	0.09	0.09	1.42	0.00	0.00
ZC3H13	23091	0.5	3.71	0.95	1.55	0.74	0.00	0.00
ZC3H14	79882	5.67	10.44	3.57	5.06	0.97	0.00	0.00
ZC3H15	55854	15.32	25.24	5.97	11.88	1.17	0.00	0.00
ZCCHC10	54819	2.76	5.68	2.15	2.2	0.95	0.00	0.00
ZCCHC9	84240	0.02	0	0	0	1.01	0.00	0.00
ZCRB1	85437	8.49	14.59	6.22	9.33	0.61	0.00	0.00
ZDHHC1	29800	1.84	1.52	1.41	0.6	1.08	0.00	0.00
ZDHHC11	79844	1.6	1.43	0.98	1.02	0.74	0.00	0.00
ZDHHC13	54503	0	0.01	0	0	0.59	0.00	0.00
ZDHHC17	23390	0.17	1.63	0.37	0.46	1.22	0.00	0.00
ZDHHC20	253832	2.7	8.86	1.51	3.3	1.21	0.00	0.00
ZDHHC6	64429	6.35	16.1	4.78	6.51	0.95	0.00	0.00
ZEB1	6935	0.19	1.04	0.27	0.26	1.32	0.00	0.00

ZFAS1	441951	1.72	6.23	2.92	2.78	0.68	0.00	0.00
ZFC3H1	196441	0.1	1.47	0.29	0.43	1.09	0.00	0.00
ZFP1	162239	13.68	23.11	15.3	16.34	0.82	0.00	0.00
ZFP69	339559	0.4	0.97	0.33	0.34	1.08	0.00	0.00
ZFP69B	65243	0.5	1.42	0.37	0.68	0.75	0.00	0.00
ZFP90	146198	1.19	3.68	1.54	1.12	0.91	0.00	0.00
ZFR	51663	5.13	19.6	5.29	7.17	0.98	0.00	0.00
ZFY	7544	0.34	0.99	0.19	0.44	1.04	0.00	0.00
ZFYVE16	9765	1.64	3.81	1.09	1.54	1.11	0.00	0.00
ZFYVE9	9372	28.46	36.92	44.21	36.91	0.81	0.00	0.00
ZGRF1	55345	0.12	0.57	0.02	0.3	1.15	0.00	0.00
ZHX1-C8orf76	100533106	1.09	3.36	1.47	1.87	0.86	0.00	0.00
ZIM2	23619	0.79	10.3	1.44	1.59	1.84	0.00	0.00
ZKSCAN5	23660	1.12	3.65	1.47	1.77	0.60	0.00	0.00
ZMPSTE24	10269	0.79	2.47	1.79	2	0.72	0.00	0.00
ZMYM2	7750	0.62	3.76	0.94	0.98	1.19	0.00	0.00
ZMYM4	9202	0	0	0	0	1.09	0.00	0.00
ZMYND11	10771	3.67	14.12	3.81	5.52	0.91	0.00	0.00
ZNF100	163227	0.46	0.99	0.17	0.36	1.55	0.00	0.00
ZNF106	64397	0.96	5.66	2.17	2.39	0.62	0.00	0.00
ZNF117	51351	0.23	0.81	0.4	0.3	0.62	0.00	0.00
ZNF12	7559	0.44	1.64	0.38	0.61	1.26	0.00	0.00
ZNF121	7675	0.43	2.87	0.75	0.86	0.93	0.00	0.00
ZNF131	7690	1.32	3.64	1.33	1.52	0.74	0.00	0.00
ZNF138	7697	0.94	0.85	0.15	0.31	1.85	0.00	0.00
ZNF14	7561	0.12	1.15	0.21	0.22	1.49	0.00	0.00
ZNF143	7702	105.72	111.88	109.11	121.61	0.98	0.00	0.00
ZNF146	7705	1.72	9.9	1.71	2.63	1.46	0.00	0.00
ZNF148	7707	0.47	1.7	0.34	0.71	0.86	0.00	0.00
ZNF17	7565	2.09	1.25	1.11	1.01	0.64	0.00	0.00
ZNF184	7738	0.57	1.95	0.43	0.57	1.36	0.00	0.00
ZNF189	7743	1.77	7.03	1.65	2.36	1.15	0.00	0.00
ZNF195	7748	1.5	2.95	0.85	1.39	0.71	0.00	0.00

ZNF219	51222	56.95	17.73	25.57	23.43	0.65	0.00	0.00
ZNF24	7572	1.91	5.94	1.79	1.79	1.04	0.00	0.00
ZNF252P	286101	0.46	1.67	0.45	0.42	1.24	0.00	0.00
ZNF253	56242	1.08	2.76	0.29	0.6	1.98	0.00	0.00
ZNF254	9534	0.75	1.73	0.35	0.52	0.59	0.00	0.00
ZNF26	7574	0.24	0.89	0.2	0.21	1.17	0.00	0.00
ZNF260	339324	0.26	0.52	0.07	0.25	1.31	0.00	0.00
ZNF267	10308	0.85	1.55	0.27	0.61	1.45	0.00	0.00
ZNF277	11179	1.06	5.09	1.59	2.67	0.58	0.00	0.00
ZNF280C	55609	0.2	0.61	0.2	0.15	1.59	0.00	0.00
ZNF281	23528	1.64	5.37	1.42	2.16	0.98	0.00	0.00
ZNF286A	57335	0.76	2.37	1.34	0.74	0.69	0.00	0.00
ZNF286B	729288	0.43	1	0.49	0.5	1.10	0.00	0.00
ZNF292	23036	0	0	0	0	0.92	0.00	0.00
ZNF322	79692	0.8	2.53	0.46	0.7	1.46	0.00	0.00
ZNF326	284695	1.39	5.31	0.58	1.79	1.22	0.00	0.00
ZNF358	140467	119.18	38.73	52.05	37.99	0.83	0.00	0.00
ZNF37A	7587	0.58	1.78	1.27	0.74	1.04	0.00	0.00
ZNF37BP	100129482	0.27	0.99	0.18	0.28	1.14	0.00	0.00
ZNF414	84330	36.92	14.67	17.9	15.73	0.62	0.00	0.00
ZNF417	147687	0.19	0.61	0.12	0.19	1.35	0.00	0.00
ZNF43	7594	0.59	0.93	0.2	0.25	1.53	0.00	0.00
ZNF430	80264	11.59	10.98	10.18	11.24	1.50	0.00	0.00
ZNF431	170959	0.71	1.2	0.28	0.69	0.82	0.00	0.00
ZNF439	90594	0.07	0.87	0.05	0.26	1.67	0.00	0.00
ZNF44	51710	0.49	2.38	0.69	0.96	0.76	0.00	0.00
ZNF440	126070	0.54	2.54	0.77	0.9	0.75	0.00	0.00
ZNF443	10224	0.56	1.33	0.42	0.47	1.05	0.00	0.00
ZNF451	26036	1.1	4.54	1.03	1.78	0.71	0.00	0.00
ZNF468	90333	1.03	1.32	1.1	0.58	0.79	0.00	0.00
ZNF484	83744	0.21	0.79	0.18	0.16	1.50	0.00	0.00
ZNF486	90649	1.7	2.62	1.92	3.26	1.30	0.00	0.00
ZNF493	284443	0.24	0.44	0.01	0.16	1.93	0.00	0.00

ZNF507	22847	0.25	1.79	0.41	0.6	0.99	0.00	0.00
ZNF511	118472	0	0.14	0	0	0.75	0.00	0.00
ZNF514	84874	0.73	2.24	2.5	1.58	0.89	0.00	0.00
ZNF518A	9849	1.23	3.05	0.78	1.42	0.80	0.00	0.00
ZNF519	162655	0.06	0.34	0.09	0.03	1.45	0.00	0.00
ZNF551	90233	0.32	0.86	0.29	0.28	1.05	0.00	0.00
ZNF555	148254	0.4	1.4	0.65	0.59	0.89	0.00	0.00
ZNF557	79230	0.33	1.26	0.33	0.56	0.83	0.00	0.00
ZNF562	54811	0.53	3.01	1.13	0.98	0.71	0.00	0.00
ZNF576	79177	14.88	8.75	6.93	6.44	0.68	0.00	0.00
ZNF587	84914	0.15	0.8	0.06	0.18	2.01	0.00	0.00
ZNF593	51042	308.72	115.43	140.62	124.78	0.69	0.00	0.00
ZNF600	162966	12.17	6.04	9.48	10.11	1.14	0.00	0.00
ZNF614	80110	0.58	1.83	0.44	1.06	0.62	0.00	0.00
ZNF620	253639	0.89	2.26	0.86	0.91	0.78	0.00	0.00
ZNF621	285268	0.76	2.06	0.94	0.95	0.70	0.00	0.00
ZNF626	199777	0.74	0.75	0.34	0.62	1.14	0.00	0.00
ZNF638	27332	0.64	3.13	0.63	1.1	1.08	0.00	0.00
ZNF644	84146	1.68	3.36	0.91	1.82	1.04	0.00	0.00
ZNF664	144348	4.23	24.88	7.84	10.89	0.61	0.00	0.00
ZNF668	79759	16.02	7.09	8.29	7.35	0.60	0.00	0.00
ZNF669	79862	0.69	2.67	0.67	1.09	1.05	0.00	0.00
ZNF670	93474	0.43	1.91	0.41	0.65	1.12	0.00	0.00
ZNF675	171392	0.86	1.38	0.43	0.99	0.94	0.00	0.00
ZNF678	339500	0.25	0.51	0.1	0.1	1.83	0.00	0.00
ZNF682	91120	1.08	0.82	0.25	0.39	1.32	0.00	0.00
ZNF69	7620	0.05	0	0.04	0.04	0.85	0.00	0.00
ZNF70	7621	0.46	1.81	0.33	0.35	1.92	0.00	0.00
ZNF700	90592	0.55	1.43	0.24	0.59	1.11	0.00	0.00
ZNF708	7562	0.6	0.92	0.14	0.3	1.34	0.00	0.00
ZNF714	148206	0.71	2.11	0.45	0.81	1.03	0.00	0.00
ZNF724	440519	0.53	1.48	0.12	0.35	2.24	0.00	0.00
ZNF761	388561	1.8	2.61	1.71	1.54	0.81	0.00	0.00

ZNF765	91661	0	0.3	0.02	0	3.67	0.00	0.00
ZNF766	90321	0.3	2.38	0.6	0.77	0.95	0.00	0.00
ZNF770	54989	0.99	3.77	0.87	1.69	0.91	0.00	0.00
ZNF771	51333	21.15	9.28	9.1	8.63	0.67	0.00	0.00
ZNF776	284309	0.67	1.84	0.54	0.69	0.80	0.00	0.00
ZNF787	126208	170.18	53.16	65.83	64.87	0.78	0.00	0.00
ZNF788P	388507	0.51	3.15	0.7	0.66	1.38	0.00	0.00
ZNF799	90576	0.7	2.03	0.29	0.73	1.38	0.00	0.00
ZNF813	126017	0.22	1.16	0.1	0.29	1.74	0.00	0.00
ZNF814	730051	0.12	0.42	0.18	0.04	1.38	0.00	0.00
ZNF823	55552	0.51	3.9	0.79	0.9	1.34	0.00	0.00
ZNF833P	401898	33.6	24.04	21.54	23.21	1.24	0.00	0.00
ZNF837	116412	7.41	1.55	2.98	2.2	0.73	0.00	0.00
ZNF841	284371	0.06	0.59	0.19	0.2	0.85	0.00	0.00
ZNF845	91664	0.16	0.42	0.06	0.15	1.20	0.00	0.00
ZNF888	388559	0.24	0.9	0.27	0.18	0.89	0.00	0.00
ZNF92	168374	1	1.11	0.23	0.65	1.26	0.00	0.00
ZNHIT6	54680	1.1	3.03	1.23	1.75	0.59	0.00	0.00
ZRANB2	9406	3.13	12.2	2.81	4.33	1.07	0.00	0.00
ZRANB3	84083	0.91	2.39	1.33	1.12	0.92	0.00	0.00
ZSCAN12	9753	2.06	11.49	4.33	5.39	1.17	0.00	0.00
ZSCAN23	222696	0.03	0.14	0.02	0.02	3.20	0.00	0.00
ZSCAN26	7741	0.45	1.76	0.63	0.65	0.83	0.00	0.00
ZSCAN30	100101467	0.28	1.03	0.55	0.34	0.75	0.00	0.00
ZUP1	221302	1.49	4.64	1.12	1.63	1.27	0.00	0.00
ZW10	9183	3.04	8.49	2.46	3.9	0.84	0.00	0.00
ZWILCH	55055	1.24	5.83	1.19	2.08	1.11	0.00	0.00
ZYG11B	79699	0.3	2.34	0.91	1.22	0.67	0.00	0.00
ZZZ3	26009	0.99	4.29	0.95	1.57	1.10	0.00	0.00

Table S1b. Genes down-regulated (>1.5-fold change, q-value < 0.001) after miR-23a knock out

Symobol	GeneID	23aKO1 FPKM	23aKO2 FPKM	Scr1 FPKM	Scr2 FPKM	log2Ratio 23aKO/Scr	q-value	p-value
A1BG	1	9.78	14.17	10.45	2.05	-0.91	0.00	0.00
A4GALT	53947	0.96	0.14	0	0.11	-3.47	0.00	0.00
AADAC	13	0.36	1.31	0.34	0.25	-1.51	0.00	0.00
ABCC3	8714	30.03	23.51	19.14	22.79	-0.63	0.00	0.00
ABCC4	10257	0.62	0.76	0.1	0.18	-2.25	0.00	0.00
ABCG2	9429	3.64	3.89	1.08	3.81	-0.61	0.00	0.00
ABRACL	58527	12.83	14.18	6.06	4.87	-1.29	0.00	0.00
ABTB2	25841	9.88	7.77	5.46	5.22	-0.72	0.00	0.00
ACKR2	1238	1.29	0.88	0.26	0.72	-1.46	0.00	0.00
ACOT11	26027	3.71	3.41	0.81	0.41	-2.62	0.00	0.00
ACP5	54	8.06	6.35	5.38	7.95	-1.03	0.00	0.00
ACSL5	51703	16.03	19.53	7.46	12.66	-0.74	0.00	0.00
ADAMTS2	9509	17.54	14.56	12.94	9.12	-1.05	0.00	0.00
ADAMTS9	56999	0.31	0.28	0.02	0.09	-2.63	0.00	0.00
ADAMTSL4	54507	13.24	10.49	5.78	5.21	-1.00	0.00	0.00
ADCY9	115	10.62	11.08	5.73	8.25	-0.69	0.00	0.00
ADGRA2	25960	0.49	0.12	0.06	0.16	-1.48	0.00	0.00
ADGRG1	9289	26.71	19.57	14.17	11.02	-0.87	0.00	0.00
ADIRF	10974	1.84	1.13	0.18	0	-3.85	0.00	0.00
ADSSL1	122622	9.15	4.64	3.16	2.94	-1.13	0.00	0.00
AFAP1-AS1	84740	0.71	0.02	0	0	-6.43	0.00	0.00
AGR2	10551	3.91	9.71	6.43	1.03	-0.92	0.00	0.00
AGTR1	185	1.03	1.01	0.14	0.31	-2.21	0.00	0.00
AHR	196	1.36	1.64	0.8	1.29	-0.62	0.00	0.00
AIFM3	150209	4.71	2.44	1.1	1.72	-1.34	0.00	0.00
AKR1C2	1646	35.63	18.92	0.53	1.31	-1.69	0.00	0.00
AKR7A3	22977	0	0	0.23	0	-2.39	0.00	0.00
ALDH2	217	97.67	104.4	65.99	65.52	-0.62	0.00	0.00
ALDH3A1	218	2.01	1.47	0.09	0.25	-3.29	0.00	0.00

ALDH3B1	221	14.83	13.46	10.48	7.63	-0.59	0.00	0.00
ALDH6A1	4329	5.39	7.91	1.94	5.26	-0.89	0.00	0.00
ALG1L2	644974	0.68	0.76	0.06	0.43	-0.99	0.00	0.00
ALOX12	239	1.28	0.96	0.53	0.33	-1.38	0.00	0.00
ALPK1	80216	1.02	0.59	0.27	0.65	-0.60	0.00	0.00
ALPL	249	6.58	2.13	0.71	2.44	-1.41	0.00	0.00
ANGPTL6	83854	1.22	0.77	0.5	0.52	-1.12	0.00	0.00
ANGPTL8	55908	273.45	270.13	182.04	55.17	-1.18	0.00	0.00
ANO9	338440	0.53	0.37	0.12	1.46	-1.09	0.00	0.00
ANXA13	312	38.83	34.85	39.6	35.67	-6.44	0.00	0.00
ANXA2	302	92.36	76.18	32.06	76.97	-0.64	0.00	0.00
ANXA3	306	1.6	1.87	0.57	0.29	-2.09	0.00	0.00
AOC3	8639	9.4	7.56	2.12	2.27	-1.91	0.00	0.00
APOL1	8542	14.76	10.82	2.45	4.5	-2.02	0.00	0.00
APOL2	23780	3.7	4.3	1.68	3	-0.78	0.00	0.00
AQP3	360	0	0.07	0	0	-1.28	0.00	0.00
AQP7P1	375719	0.64	0.28	0	0.04	-2.05	0.00	0.00
ARAP2	116984	0.04	0.08	0	0.01	-3.95	0.00	0.00
ARAP3	64411	0.24	0.08	0.01	0.06	-2.33	0.00	0.00
AREG	374	5.61	1.32	0.79	2.29	-1.08	0.00	0.00
ARHGAP40	343578	6.98	5.92	8.29	7.57	-1.39	0.00	0.00
ARHGAP45	23526	4.49	3.61	1.9	1.47	-1.13	0.00	0.00
ARHGDIB	397	2.36	5.67	3.44	1.49	-0.71	0.00	0.00
ARL8A	127829	15.85	12.95	9.81	8.13	-0.75	0.00	0.00
ARSD	414	4.3	4.05	1.41	2.89	-1.02	0.00	0.00
ARSI	340075	3.67	1.3	0.03	0.05	-5.88	0.00	0.00
ASNS	440	0.38	0.16	0.23	0.25	-1.27	0.00	0.00
ASPHD1	253982	15.48	14.9	9.49	6.27	-0.92	0.00	0.00
ASS1	445	200.01	187.4	169.37	74.14	-0.66	0.00	0.00
ATF3	467	4.55	1.23	1.1	2.71	-0.60	0.00	0.00
ATOH8	84913	0.55	0.2	0.09	0.33	-0.90	0.00	0.00
ATP1A1	476	403.25	397.44	185.07	306.97	-0.70	0.00	0.00
ATP1A3	478	0.05	0.14	0	0	-4.63	0.00	0.00

ATP1B4	23439	2.19	2.4	0.93	1.7	-0.81	0.00	0.00
ATP2B2	491	0.16	0.44	0.11	0.02	-2.31	0.00	0.00
ATP4A	495	29.62	50.83	45.98	5.53	-0.62	0.00	0.00
ATP6AP2	10159	11.46	11.49	7.61	7.32	-0.61	0.00	0.00
ATP8B3	148229	0.12	0.06	0	0	-0.61	0.00	0.00
ATRIP-TREX1	111822955	0.56	0.65	0.09	0.23	-1.53	0.00	0.00
ATXN2	6311	7.24	7.33	2.45	6.87	-0.59	0.00	0.00
AXL	558	4.06	1.94	0.65	2.74	-0.79	0.00	0.00
AZGP1	563	11.01	8.98	3.29	14.6	-0.73	0.00	0.00
AZGP1P1	646282	0.88	1.12	0.38	0.29	-1.58	0.00	0.00
B3GNT3	10331	33.37	31.43	13.25	5.12	-1.83	0.00	0.00
B4GALNT4	338707	0	0	0	0.05	-1.88	0.00	0.00
B4GALT1	2683	22.8	19.51	9.81	16.89	-0.66	0.00	0.00
B4GALT6	9331	0.12	0.15	0.03	0	-3.74	0.00	0.00
BATF	10538	1.55	0.48	0	0.06	-5.00	0.00	0.00
BDNF-AS	497258	0.02	0.08	0	0.01	-3.97	0.00	0.00
BEAN1	146227	1.33	0.67	0.19	0.06	-3.02	0.00	0.00
BGN	633	2.46	0.68	0.46	1.05	-1.01	0.00	0.00
BHLHE40	8553	18.84	22.13	21.38	23.19	-1.55	0.00	0.00
BICDL1	92558	6.13	8.81	1.99	1.52	-1.94	0.00	0.00
BIK	638	9.86	6.84	3.39	3.55	-1.17	0.00	0.00
BIN1	274	1.5	1.37	0.67	0.63	-1.28	0.00	0.00
BIRC7	79444	5.19	2.38	1.25	2	-1.21	0.00	0.00
BPIFB2	80341	133.73	134.47	52.3	11.01	-2.07	0.00	0.00
BSPRY	54836	2.07	0.92	0.27	0.22	-1.78	0.00	0.00
BTBD11	121551	2.65	3.53	0.23	0.44	-3.24	0.00	0.00
BUD23	114049	15.53	12.63	9.4	6.56	-0.80	0.00	0.00
C1RL	51279	0	0	0	0.01	-0.79	0.00	0.00
C2	717	105.86	96.81	71.13	51.66	-0.72	0.00	0.00
C22orf23	84645	3.7	3.08	2.33	1.22	-1.05	0.00	0.00
C3	718	723.37	431.11	298.58	408.98	-0.71	0.00	0.00
C3orf86	102724231	2.31	1	0.88	0	-1.63	0.00	0.00
C4A	720	31.62	18.59	10.36	13.21	-1.09	0.00	0.00

C4B	721	88.27	72.87	41.53	47.85	-0.85	0.00	0.00
C4B_2	100293534	35.05	27.59	14.63	20.76	-0.82	0.00	0.00
C5AR1	728	0	0	0	0	-1.54	0.00	0.00
C8A	731	0.56	0.35	0.16	0.12	-1.74	0.00	0.00
CADM1	23705	0.99	0.53	0.04	0.15	-2.86	0.00	0.00
CALCOCO1	57658	7.12	5.53	3.3	4.64	-0.66	0.00	0.00
CALY	50632	0.47	0.67	0.58	0.79	-2.00	0.00	0.00
CAMK2N2	94032	0.87	0.67	0	0.04	-5.30	0.00	0.00
CAPN2	824	8.38	8.76	1.55	6.49	-1.10	0.00	0.00
CARMIL1	55604	1.63	2.26	0.17	0.44	-2.70	0.00	0.00
CATSPERG	57828	0.35	0.23	0.04	0.17	-1.88	0.00	0.00
CBSL	102724560	14.92	10.47	7.22	4.74	-0.95	0.00	0.00
CCDC120	90060	3.84	4.67	2.89	1.92	-0.79	0.00	0.00
CCDC17	149483	0.39	0.16	0	0.11	-2.36	0.00	0.00
CCDC187	399693	0.44	0.34	0.07	0.12	-2.00	0.00	0.00
CCDC28A	25901	6.86	7.34	2.64	5.42	-0.83	0.00	0.00
CCL20	6364	45.13	43.09	35.6	53.78	-1.59	0.00	0.00
CCNB1IP1	57820	58.24	35.93	29.35	31.93	-0.64	0.00	0.00
CCNJL	79616	1.44	1.21	0.41	0.18	-1.96	0.00	0.00
CD101	9398	0.04	0.03	0	0	-4.87	0.00	0.00
CD14	929	1.13	0.86	0.59	0.75	-3.31	0.00	0.00
CD163L1	283316	0.25	0.22	0.01	0.03	-3.37	0.00	0.00
CD22	933	0	0	0.02	0	-2.33	0.00	0.00
CD24	100133941	13.26	9.29	0.37	1.2	-3.86	0.00	0.00
CD302	9936	1.21	2.64	0.83	0.9	-1.15	0.00	0.00
CD4	920	0.96	0.51	0.26	0.64	-0.98	0.00	0.00
CD68	968	46.71	41.98	48.32	41.67	-0.61	0.00	0.00
CD7	924	23.28	20.97	14.75	7.19	-0.98	0.00	0.00
CD9	928	8.4	6.19	1.56	1.18	-2.38	0.00	0.00
CDA	978	19.77	15.98	6.8	3.29	-1.82	0.00	0.00
CDC42BPB	9578	10.98	8.62	1.47	11.12	-0.66	0.00	0.00
CDC42EP5	148170	4.48	2.68	0.3	0.14	-3.93	0.00	0.00
CDCP1	64866	0.14	0.07	0.01	0	-4.57	0.00	0.00

CDH1	999	10.42	14.76	3.1	2.31	-2.22	0.00	0.00
CDH3	1001	0.1	0	0	0	-1.79	0.00	0.00
CDHR2	54825	24.56	25.38	14.91	12.25	-0.92	0.00	0.00
CDKN2A	1029	13.06	10.68	7.7	2.57	-1.14	0.00	0.00
CEBPB	1051	75.64	39.03	35.84	36.99	-0.65	0.00	0.00
CEBPD	1052	35.36	17.1	15.25	10.43	-1.02	0.00	0.00
CELF3	11189	0.13	0.06	0.06	0.15	-5.37	0.00	0.00
CERCAM	51148	10.1	7.57	6.16	4.46	-0.73	0.00	0.00
CES1	1066	0	0.04	0.16	0.09	-0.85	0.00	0.00
CFB	629	68.52	43.87	17.18	29.8	-1.26	0.00	0.00
CFD	1675	5.05	4.43	2.68	2.98	-0.71	0.00	0.00
CHAC1	79094	7.29	1.54	3.72	1.76	-0.70	0.00	0.00
CHADL	150356	4.93	3.42	2.92	2.12	-0.60	0.00	0.00
CHD5	26038	0.2	0.17	0.08	0.12	-1.06	0.00	0.00
CHKB-CPT1B	386593	2.3	1.8	0.91	1.54	-0.74	0.00	0.00
CHMP4C	92421	5.31	4.58	1.06	3.6	-1.10	0.00	0.00
CHP1	11261	44.69	52.21	31.26	35.72	-0.63	0.00	0.00
CHST6	4166	2.11	1.92	0.23	0.76	-2.18	0.00	0.00
CIZ1	25792	0.01	0	0.01	0	-0.58	0.00	0.00
CLDN1	9076	13.18	18.69	7.26	9.78	-0.91	0.00	0.00
CLDN11	5010	33.08	20.16	2.3	12.82	-1.87	0.00	0.00
CLDN3	1365	5.57	5.54	3.54	0.81	-1.33	0.00	0.00
CLDN6	9074	0.02	0.01	0	0.04	-0.74	0.00	0.00
CLEC2L	154790	1.04	0.59	0.11	0	-1.97	0.00	0.00
CLIC3	9022	21.01	9.88	10.94	5.35	-0.83	0.00	0.00
CLIP3	25999	0.72	0.43	0.35	0.36	-0.69	0.00	0.00
CLMN	79789	5.31	4.09	0.38	1.28	-2.91	0.00	0.00
CLU	1191	47.34	62.36	58.52	0.88	-0.85	0.00	0.00
CMTM8	152189	21.06	18.72	13.45	11.25	-0.59	0.00	0.00
CNTNAP1	8506	7.01	2.8	1.35	4.76	-0.70	0.00	0.00
CNTNAP3P2	643827	0.75	0.46	0.26	0.33	-1.32	0.00	0.00
COBL	23242	3.53	4.32	1.4	2.06	-1.15	0.00	0.00
COL26A1	136227	0.14	0.16	0	0.03	-2.88	0.00	0.00

COL9A3	1299	57.38	23.15	8.62	39.95	-0.71	0.00	0.00
COLGALT2	23127	17.86	12.68	5.61	14.45	-0.60	0.00	0.00
COMP	1311	3.71	1.72	0.28	0.18	-3.55	0.00	0.00
COPG2	26958	8.9	7.28	2.82	7.61	-0.64	0.00	0.00
CORO2B	10391	0.64	0.75	0.29	0.09	-2.04	0.00	0.00
CORO7	79585	4.79	3.63	0.44	3.67	-1.05	0.00	0.00
COX20	116228	10.17	13.09	7.28	9.34	-0.88	0.00	0.00
CPA5	93979	0.72	0.64	0.09	0.12	-2.76	0.00	0.00
CPLX1	10815	5.39	3.59	3.59	2.25	-0.62	0.00	0.00
CPN1	1369	2.89	4.71	2.84	0.83	-1.04	0.00	0.00
CPNE7	27132	0.31	0.18	0.01	0.05	-2.80	0.00	0.00
CPT1A	1374	9.92	10.82	3.43	10.69	-0.63	0.00	0.00
CRIP1	1396	71.3	61.36	42.02	28.97	-0.89	0.00	0.00
CRYAB	1410	18.05	13.54	7.84	8.13	-0.83	0.00	0.00
CRYBB2P1	1416	0	0	0.04	0	-0.71	0.00	0.00
CRYBB3	1417	1.41	1.41	0	0	-9.45	0.00	0.00
CSF1R	1436	0.04	0.02	0	0.01	-3.90	0.00	0.00
CSGALNACT1	55790	0.18	0.16	0	0.01	-4.83	0.00	0.00
CSTF2T	23283	0.21	0.17	0.14	0	-3.95	0.00	0.00
CTAG1B	1485	0	0	0	0.02	-8.78	0.00	0.00
CTDSPL	10217	3.83	3.87	0	0.05	-7.27	0.00	0.00
CTGF	1490	108.85	119.9	36.35	82.14	-0.95	0.00	0.00
CTH	1491	9	6.35	3.31	5.02	-0.83	0.00	0.00
CTSB	1508	125.48	124.11	16.02	100.08	-1.13	0.00	0.00
CTSS	1520	0.68	0.43	0.15	0.16	-1.67	0.00	0.00
CUBN	8029	0.04	0.32	0.06	0.04	-2.18	0.00	0.00
CYB5A	1528	137.77	155.69	92.84	101.64	-0.59	0.00	0.00
CYP17A1	1586	0.35	0.83	0.2	0	-2.52	0.00	0.00
CYP1A1	1543	6.65	1.5	1.03	0.87	-2.10	0.00	0.00
CYP21A2	1589	2.43	1.14	0.59	0.7	-1.46	0.00	0.00
CYP24A1	1591	18.31	14.33	1.66	17.73	-0.75	0.00	0.00
CYP2D6	1565	1.22	3.71	2.5	0.25	-0.85	0.00	0.00
CYP2S1	29785	0.76	1.77	0.88	2.39	-1.83	0.00	0.00

CYP2U1	113612	0.27	0.11	0	0.15	-1.95	0.00	0.00
CYP3A5	1577	2	4.54	1.89	2.73	-0.64	0.00	0.00
DBNDD2	55861	46.7	30.77	24.43	20.93	-0.78	0.00	0.00
DCBLD2	131566	1.55	1.78	1.72	2	-0.86	0.00	0.00
DCUN1D3	123879	2.43	2.44	1.12	2.03	-0.61	0.00	0.00
DDIT4	54541	56.66	28.49	27.63	15.26	-0.98	0.00	0.00
DDOST	1650	20.66	9.84	10.81	3.87	-1.04	0.00	0.00
DEFB1	1672	72.66	41.95	28.05	36.56	-0.83	0.00	0.00
DENND3	22898	0.29	0.2	0.02	0.02	-4.47	0.00	0.00
DENND6B	414918	3.99	2.64	2.42	1.86	-0.62	0.00	0.00
DEPP1	11067	23.06	8.11	5.47	11.34	-0.90	0.00	0.00
DGUOK-AS1	100874048	42.17	29.7	13.48	29.65	-0.73	0.00	0.00
DHRS1	115817	244.66	249.34	121.76	316.39	-2.94	0.00	0.00
DHRS11	79154	29.84	40.27	29.23	14.39	-0.69	0.00	0.00
DHRS12	79758	4.73	4.46	2.65	2.08	-0.75	0.00	0.00
DIO1	1733	21.23	28.95	40.6	18.25	-2.85	0.00	0.00
DKK1	22943	14.84	27.41	2.95	4.36	-2.52	0.00	0.00
DLK1	8788	55.6	46.42	41.42	18.58	-0.76	0.00	0.00
DLL4	54567	0	0	0	0	-1.71	0.00	0.00
DMKN	93099	9.09	4.88	0.57	0.55	-3.73	0.00	0.00
DNAH10	196385	0.11	0.28	0.02	0.06	-2.41	0.00	0.00
DNM1	1759	3.9	2.29	0.52	0.92	-2.09	0.00	0.00
DNMBP	23268	2.1	2.3	0.46	1.62	-0.88	0.00	0.00
DOP1B	9980	1.41	1.62	0.34	0.78	-1.39	0.00	0.00
DSG2	1829	2.56	3.06	0.08	0.33	-3.98	0.00	0.00
DUSP13	51207	14.77	9.31	8.6	6.61	-0.66	0.00	0.00
DUSP4	1846	1.09	0.93	0.58	0.68	-0.66	0.00	0.00
DUSP5	1847	3.01	0.42	0.34	0.45	-2.13	0.00	0.00
DYSF	8291	0.37	0.22	0.1	0.1	-1.54	0.00	0.00
EBI3	10148	6.62	1.09	1.68	3.07	-0.68	0.00	0.00
EFCAB8	388795	0.74	0.64	0.58	1.24	-1.51	0.00	0.00
EGFL7	51162	8.07	3.3	0.7	0.67	-3.03	0.00	0.00
EGFL8	80864	1.32	0.07	0	0.09	-3.95	0.00	0.00

EGLN3	112399	2.61	0.15	0	0.08	-4.15	0.00	0.00
ELF3	1999	9.44	4.33	3.96	4.05	-0.94	0.00	0.00
ELF5	2001	1.96	3.22	0.1	0.57	-2.98	0.00	0.00
EMC10	284361	52.72	44.58	62.21	58.49	-4.67	0.00	0.00
EME2	197342	0.72	0.55	0.78	0.58	-0.79	0.00	0.00
EML2	24139	7.61	3.41	1.71	3.04	-1.11	0.00	0.00
EMP2	2013	2.09	1.45	0.69	1.55	-0.76	0.00	0.00
ENC1	8507	8.65	10.64	2.39	7.06	-0.98	0.00	0.00
ENG	2022	12.02	13.93	6.66	6.34	-0.99	0.00	0.00
EOMES	8320	0.63	0.54	0.04	0.19	-2.23	0.00	0.00
EPB41L4A-AS1	114915	6.26	5.06	4.05	3.37	-0.61	0.00	0.00
EPS8L1	54869	2.92	1.7	0.76	0.45	-1.82	0.00	0.00
ERBB3	2065	49.64	42.48	17.11	31.08	-1.01	0.00	0.00
ERMARD	55780	2.21	1.3	0.82	1.07	-0.95	0.00	0.00
ESRP1	54845	0.37	0.55	0.01	0.01	-5.09	0.00	0.00
ETS2	2114	33.09	21.51	9.15	20.5	-0.92	0.00	0.00
ETV7	51513	0.15	0.12	0.01	0.02	-2.65	0.00	0.00
EVC	2121	0.15	0.16	0.01	0.04	-2.41	0.00	0.00
EVPLL	645027	0.23	0.2	0	0	-4.95	0.00	0.00
EZR-AS1	101409257	0.73	0.78	0.42	0	-1.82	0.00	0.00
FABP1	2168	0	0	0	0	-1.37	0.00	0.00
FAM102A	399665	13.33	11.54	5.83	4.77	-0.78	0.00	0.00
FAM110C	642273	1.12	0.85	0.12	0.52	-1.64	0.00	0.00
FAM131C	348487	2.42	0.33	0.48	0.83	-1.06	0.00	0.00
FAM181A	90050	1.44	1.17	0.18	0.23	-2.69	0.00	0.00
FAM19A5	25817	4.38	4.22	1.92	1.96	-1.06	0.00	0.00
FAM20A	54757	0.3	0.57	0.06	0.78	-2.54	0.00	0.00
FAM86B1	85002	8.34	5.16	4	4.95	-0.73	0.00	0.00
FAM86B3P	286042	1.79	1.48	0.45	1.71	-0.62	0.00	0.00
FAM86JP	100125556	4.23	2.63	2.11	1.94	-0.68	0.00	0.00
FAXDC2	10826	4.33	5.52	1.82	1.83	-1.25	0.00	0.00
FBXL8	55336	3.11	2.92	2.55	1.33	-0.63	0.00	0.00
FBXO10	26267	1.83	1.58	0.84	1.33	-0.67	0.00	0.00

FCHO1	23149	4.47	4.09	2.88	2.8	-0.58	0.00	0.00
FGA	2243	82.62	77.38	32.42	42.6	-1.09	0.00	0.00
FGB	2244	5.56	10.6	2.51	3.76	-1.37	0.00	0.00
FGF13	2258	0	0	0	0.04	-1.93	0.00	0.00
FGG	2266	75.1	70.39	28.65	53.32	-0.83	0.00	0.00
FIBCD1	84929	5.73	3.71	4.16	1.25	-0.62	0.00	0.00
FIGNL2	401720	0.34	0.29	0.01	0.01	-4.83	0.00	0.00
FMNL3	91010	0.19	0.13	0.07	0.06	-1.35	0.00	0.00
FOXQ1	94234	33.03	30.54	11.53	17.01	-1.16	0.00	0.00
FREM1	158326	0.15	0.19	0.01	0.07	-2.59	0.00	0.00
FRMPD2	143162	0.96	0.69	0.37	0.55	-0.90	0.00	0.00
FST	10468	5	4.79	1.24	2.75	-1.29	0.00	0.00
FUCA1	2517	46.69	42.83	20.93	34.48	-0.65	0.00	0.00
FUT1	2523	0.38	0.04	0.06	0.02	-2.36	0.00	0.00
FUT2	2524	1.19	1.11	0.27	0.37	-1.90	0.00	0.00
FUT3	2525	0.98	0.5	0.11	0.33	-1.77	0.00	0.00
FXYD1	5348	26.3	9.32	15.01	7.3	-0.64	0.00	0.00
FXYD3	5349	4.97	0.28	1.41	0.72	-1.39	0.00	0.00
G6PC	2538	0.36	0.41	0.03	0.09	-2.73	0.00	0.00
GABARAPL1	23710	14.42	7.28	3.12	10.01	-0.74	0.00	0.00
GADD45A	1647	34.59	24.98	12.09	23.81	-0.73	0.00	0.00
GADD45B	4616	11.56	5.52	3.82	6.71	-0.71	0.00	0.00
GALNT6	11226	1.85	1.28	0.66	1.13	-7.51	0.00	0.00
GARNL3	84253	0.8	0.67	0.17	0.54	-1.04	0.00	0.00
GAS7	8522	0.1	0.04	0.01	0.01	-2.85	0.00	0.00
GATAD2B	57459	2.99	2.46	1.1	2.43	-0.65	0.00	0.00
GC	2638	0.1	0.32	0	0	-4.74	0.00	0.00
GCKR	2646	10.66	6.78	3.71	4.59	-1.16	0.00	0.00
GCNT3	9245	9	7.29	4.31	6.53	-0.64	0.00	0.00
GDF15	9518	545.56	201.87	226.75	191.23	-0.79	0.00	0.00
GDNF	2668	2.08	1.27	0.01	0.36	-3.40	0.00	0.00
GDPD5	81544	6.37	4.74	2.13	1.07	-1.60	0.00	0.00
GFOD1	54438	0.87	1.36	0.78	0.62	-0.89	0.00	0.00

GHRL	51738	0.04	0.09	0.01	0.02	-2.05	0.00	0.00
GJC2	57165	22.49	8.83	28.68	13.52	-2.88	0.00	0.00
GLDC	2731	31.17	39.28	16.27	29.79	-0.61	0.00	0.00
GLE1	2733	7.7	9.64	3.09	7.74	-0.69	0.00	0.00
GLIS2	84662	0.71	0.92	0.26	0.21	-1.79	0.00	0.00
GLS2	27165	8.34	7.97	4.91	4.3	-0.72	0.00	0.00
GNAS	2778	685.7	522.61	351.63	364	-0.75	0.00	0.00
GNPTG	84572	30.63	33.78	20.72	20.96	-0.62	0.00	0.00
GNS	2799	29.78	34.14	10.63	29.3	-0.74	0.00	0.00
GOLGA2	2801	9.46	8.68	2.57	6.63	-0.96	0.00	0.00
GOLGA6C	653641	0.19	0.2	0.04	0.09	-1.56	0.00	0.00
GOT1	2805	3.65	1.78	2.08	1.19	-0.76	0.00	0.00
GPAT3	84803	6.03	3.82	1.49	4.43	-0.73	0.00	0.00
GPC1	2817	9.41	7.39	2.83	1.42	-1.97	0.00	0.00
GPC3	2719	1.27	2.08	0.92	2.78	-0.68	0.00	0.00
GPC6-AS2	100873973	0.26	0.21	0	0.18	-1.41	0.00	0.00
GPR135	64582	0.25	0.24	0.05	0.05	-1.64	0.00	0.00
GPR143	4935	1.24	1.05	0	0	-6.80	0.00	0.00
GPRC5D-AS1	100506314	0.06	0.06	0.02	0.04	-1.70	0.00	0.00
GPX3	2878	227.98	218.46	187.62	81.23	-0.74	0.00	0.00
GRAMD1A	57655	34.61	21.08	16.88	13.74	-0.88	0.00	0.00
GRAMD1B	57476	0.08	0.15	0	0	-3.85	0.00	0.00
GRAMD2A	196996	0.1	0.14	0	0	-4.85	0.00	0.00
GRB14	2888	0.34	0.6	0.04	0.07	-2.78	0.00	0.00
GRHL1	29841	0.78	0.79	0.15	0.66	-1.02	0.00	0.00
GRIP2	80852	2.16	0.78	0	0	-9.46	0.00	0.00
GSDME	1687	27.33	22.89	13.14	17.81	-0.69	0.00	0.00
GTF2IP20	441124	0.85	0.86	0.42	0.75	-0.67	0.00	0.00
GUCY2EP	390226	0.08	0.01	0	0.01	-3.13	0.00	0.00
H19	283120	1.22	0	0	0	-6.73	0.00	0.00
H1F0	3005	176.4	118.74	52.01	122.58	-0.77	0.00	0.00
HAMP	57817	199.1	86.56	18.8	44.64	-2.18	0.00	0.00
HBA1	3039	9.15	12.54	3.64	0.38	-2.23	0.00	0.00

HEG1	57493	1.14	0.9	0.37	0.86	-0.74	0.00	0.00
HEPN1	641654	16.38	15.3	8.76	12.03	-0.61	0.00	0.00
HES5	388585	0.42	0.16	0	0.04	-3.85	0.00	0.00
HEY1	23462	0.74	0.92	0.03	0.36	-2.15	0.00	0.00
HFE	3077	9.36	10.21	5.17	7.08	-0.61	0.00	0.00
HGFAC	3083	5.08	2.77	0.5	1.07	-2.22	0.00	0.00
HHAT	55733	0.97	0.93	0.39	0.3	-1.94	0.00	0.00
HIST1H2AC	8334	1.16	3.76	0.07	1.27	-2.31	0.00	0.00
HIST1H2BD	3017	3.23	3.4	3.67	0.61	-1.07	0.00	0.00
HIST2H2BE	8349	1.15	1.81	0.6	0	-2.28	0.00	0.00
HKDC1	80201	13.43	18.07	4.81	7.08	-1.40	0.00	0.00
HKR1	284459	4.17	2.64	1.01	2.76	-0.83	0.00	0.00
HLA-E	3133	1.12	1.55	0.56	0.45	-1.36	0.00	0.00
HMGCL	3155	61.76	47.55	36.17	37.27	-0.58	0.00	0.00
HNF1B	6928	1.41	1.52	0.07	0.08	-4.42	0.00	0.00
HOXD1	3231	5.99	5.55	2.7	4.64	-0.66	0.00	0.00
HOXD3	3232	0	0	0	0	-0.58	0.00	0.00
HOXD4	3233	0.04	0.03	0.03	0.05	-5.53	0.00	0.00
HP	3240	24.51	22.88	8.64	7.03	-1.60	0.00	0.00
HR	55806	0.96	0.19	0.11	0.35	-1.13	0.00	0.00
HSD17B2	3294	3.84	9.3	0.38	0.04	-4.94	0.00	0.00
HSD17B8	7923	5.57	3.44	3.05	1.13	-1.09	0.00	0.00
HSPA1A	3303	0.87	1.98	1.39	4.52	-1.84	0.00	0.00
HYI	81888	1.97	1.21	0.88	1.28	-0.59	0.00	0.00
ICAM2	3384	3.17	1.4	1.39	0.36	-1.82	0.00	0.00
ICAM5	7087	2.99	0.99	0.46	1.17	-0.93	0.00	0.00
IFITM1	8519	53.37	20.42	21.81	21.27	-0.77	0.00	0.00
IFNLR1	163702	1.5	1.36	0.14	0.18	-3.21	0.00	0.00
IGFBP2	3485	13.31	5.61	6.78	4.83	-0.68	0.00	0.00
IGFBP4	3487	28.02	21.87	16.21	12.74	-0.78	0.00	0.00
IGFL2	147920	0.19	0.09	0	0.01	-4.74	0.00	0.00
IL11	3589	2.53	0.14	0.17	1.22	-0.96	0.00	0.00
IL17D	53342	4.52	5.38	3.21	2.58	-0.79	0.00	0.00

IL23A	51561	1.13	0.84	0.58	0.54	-1.57	0.00	0.00
IL4I1	259307	2.38	3.59	1	1.47	-1.30	0.00	0.00
INKA1	389119	0.46	0.24	0.08	0.05	-2.09	0.00	0.00
INTS6-AS1	100507398	0.47	0.74	0.25	0.25	-1.10	0.00	0.00
IP6K3	117283	0.58	0.06	0.06	0.05	-2.50	0.00	0.00
IRF5	3663	2.56	2.46	0.98	3.24	-1.06	0.00	0.00
IRF9	10379	2.2	1.62	0.46	0.81	-1.62	0.00	0.00
ISM2	145501	12.05	7.23	2.95	7.1	-0.95	0.00	0.00
ITGA3	3675	1.01	0.55	0.24	0.4	-1.26	0.00	0.00
ITGAL	3683	0.84	0.95	0.4	0.36	-1.23	0.00	0.00
ITGB1	3688	21.01	12.64	4.7	7.16	-0.81	0.00	0.00
ITGB4	3691	5.9	4.27	1.5	1.35	-1.73	0.00	0.00
ITIH3	3699	7.85	7.78	5.81	8.22	-0.87	0.00	0.00
ITIH4	3700	0.56	0.48	0.14	0.27	-0.92	0.00	0.00
ITIH5	80760	2.56	1.37	0	0.06	-7.16	0.00	0.00
ITPR1	3708	0.96	1.28	0.18	1.35	-0.62	0.00	0.00
ITPR3	3710	45.81	37.27	15.83	22.83	-0.75	0.00	0.00
JAG2	3714	0.28	0.12	0.09	0.05	-2.47	0.00	0.00
JAK3	3718	4.19	2.49	1.34	1.56	-1.20	0.00	0.00
JDP2	122953	6.09	1.96	2.11	1.65	-1.16	0.00	0.00
JUP	3728	45.86	45.39	34.17	19.86	-0.73	0.00	0.00
KBTBD4	55709	2	1.92	0.32	1.36	-1.25	0.00	0.00
KCNC4	3749	0.78	0.69	0.45	0.38	-0.83	0.00	0.00
KCNK13	56659	1.96	3.72	2.06	1.22	-0.79	0.00	0.00
KCTD13	253980	11.3	7.36	5.65	4.45	-0.93	0.00	0.00
KDM4A	9682	8.57	8.59	4.01	7.24	-0.61	0.00	0.00
KIAA1211L	343990	0.5	1.27	0.29	0.29	-1.48	0.00	0.00
KIF1A	547	0.6	0.47	0.02	0.02	-4.54	0.00	0.00
KIF21B	23046	0.38	0.14	0.11	0.11	-1.49	0.00	0.00
KIF3C	3797	10.97	7.99	5.48	6.4	-0.67	0.00	0.00
KLC3	147700	0.75	0.35	0.08	0.18	-2.08	0.00	0.00
KLF13	51621	4.91	5.05	2.02	3.9	-0.65	0.00	0.00
KLHL14	57565	2.12	3.66	0.32	1	-2.14	0.00	0.00

KLHL23	151230	0.87	0.29	0	0	-7.47	0.00	0.00
KLK6	5653	1.44	0.46	0.24	0.26	-2.02	0.00	0.00
KNDC1	85442	0.73	0.12	0.01	0.1	-2.12	0.00	0.00
KNG1	3827	1.2	2.66	1.12	0.58	-1.20	0.00	0.00
KREMEN1	83999	10.54	7.86	3.2	7.59	-0.76	0.00	0.00
KREMEN2	79412	6.18	2.33	1.45	1.84	-1.27	0.00	0.00
KRT15	3866	0.5	0.41	0.07	0.19	-1.75	0.00	0.00
L1CAM	3897	1.98	0.48	0.04	0.02	-5.30	0.00	0.00
LAIR2	3904	2.64	2.52	1.03	0.39	-1.95	0.00	0.00
LAMA3	3909	1.54	1.91	0.43	1.67	-0.84	0.00	0.00
LAMB3	3914	0.9	0.65	0.21	0.23	-1.18	0.00	0.00
LAPTM5	7805	1.68	1.33	0.36	1.39	-0.88	0.00	0.00
LARGE2	120071	18.35	7.89	8.08	4.4	-1.07	0.00	0.00
LARP6	55323	3.12	0.88	0.73	1.6	-1.19	0.00	0.00
LCK	3932	6.04	5.38	3.59	3.24	-0.74	0.00	0.00
LCN10	414332	1.62	0.47	0.24	0.66	-1.38	0.00	0.00
LCN15	389812	0.11	0.15	0.14	0.22	-1.27	0.00	0.00
LCN2	3934	49.03	4.98	0.86	1.23	-4.67	0.00	0.00
LDLRAD1	388633	5.53	5.45	1.2	1.34	-2.14	0.00	0.00
LDLRAD2	401944	0.15	0.24	0	0.08	-2.33	0.00	0.00
LENG8	114823	0	0.34	0.13	1.19	-0.99	0.00	0.00
LENG8-AS1	104355426	3.29	2.65	2.26	8.19	-1.32	0.00	0.00
LETMD1	25875	33.82	28.71	17.42	20.25	-0.68	0.00	0.00
LGALS3	3958	55.35	80.92	53.96	27.87	-0.73	0.00	0.00
LGALS7B	653499	15	13.49	8.85	2.69	-1.28	0.00	0.00
LGALSL-DT	105374771	0.71	0.69	0.53	0.21	-0.82	0.00	0.00
LGI4	163175	0.29	0.15	0.08	0.09	-2.16	0.00	0.00
LGSN	51557	1.9	1.91	0.59	1.8	-0.68	0.00	0.00
LIF	3976	4.18	3.12	1.79	1.37	-1.20	0.00	0.00
LIM2	3982	10.04	4.92	0.88	1.49	-2.64	0.00	0.00
LIPH	200879	0.81	1.58	0.7	0.22	-1.46	0.00	0.00
LMTK3	114783	1.82	1.11	0.36	0.65	-1.46	0.00	0.00
LRP4	4038	4.67	4.81	1.59	4.47	-0.63	0.00	0.00

LRRC57	255252	3.26	4.76	2.15	4.04	-0.71	0.00	0.00
LRRK1	79705	0.67	0.4	0.3	0.21	-1.02	0.00	0.00
LTBP4	8425	0.52	0.16	0.05	0.13	-1.76	0.00	0.00
LY96	23643	3.69	4.11	2.48	1.84	-0.71	0.00	0.00
LYZ	4069	206.66	229.36	61.63	129.05	-1.22	0.00	0.00
MARCKS	4082	14.8	13.4	6.45	11.16	-0.68	0.00	0.00
MARVELD3	91862	7.97	10.39	3.5	5.41	-1.06	0.00	0.00
MBL2	4153	29.95	32.46	49.31	37.45	-1.58	0.00	0.00
MBP	4155	2.84	3.23	0.84	1.65	-1.31	0.00	0.00
MCHR1	2847	1.34	1.01	0.57	0.41	-1.26	0.00	0.00
MDK	4192	278.25	187.47	219.08	75.48	-0.64	0.00	0.00
MEGF6	1953	0.49	0.41	0.03	0.05	-3.25	0.00	0.00
MFAP2	4237	16.89	4.17	4.41	5.52	-1.08	0.00	0.00
MICAL2	9645	0.19	0.23	0.06	0.24	-1.67	0.00	0.00
MISP	126353	13.65	11.58	5.62	4.89	-1.26	0.00	0.00
MLXIPL	51085	17.08	22.42	15.72	8.95	-0.65	0.00	0.00
MMP7	4316	0	0.37	0	0	-6.44	0.00	0.00
MOAP1	64112	5.57	5.43	1.16	5.52	-0.73	0.00	0.00
MOGAT1	116255	0.04	0.16	0	0	-4.85	0.00	0.00
MPP1	4354	3.01	6.52	3.26	1.92	-0.87	0.00	0.00
MPP7	143098	0.15	0.45	0.08	0.14	-1.48	0.00	0.00
MPZ	4359	11.21	6.18	4.26	4.27	-1.04	0.00	0.00
MT1G	4495	75.79	56.45	67.03	8.26	-0.78	0.00	0.00
MTCL1	23255	1.41	2.41	0.63	1.63	-0.67	0.00	0.00
MTHFD2	10797	75.71	41.38	22.89	50.6	-0.68	0.00	0.00
MUC13	56667	1	1.09	0.04	0.26	-2.81	0.00	0.00
MUC20	200958	2.6	4.61	2.16	0.86	-1.14	0.00	0.00
MUC5AC	4586	0.31	0.12	0.11	0.04	-1.97	0.00	0.00
MUC5B	727897	0.06	0.09	0.03	0.01	-2.85	0.00	0.00
MVP	9961	45.33	44.91	28.79	24.31	-0.63	0.00	0.00
MYBPHL	343263	5.26	5.55	3.18	2.27	-1.03	0.00	0.00
MYC	4609	0	0	0	0	-0.60	0.00	0.00
MYH14	79784	8.65	6.51	4.16	4.19	-0.86	0.00	0.00

MYO10	4651	1.8	1.88	0.05	0.07	-4.68	0.00	0.00
MYO15B	80022	8.1	12.98	6.94	2.26	-1.17	0.00	0.00
MYO19	80179	14.55	15.82	8.39	9.77	-0.85	0.00	0.00
MYO1A	4640	2.52	4.37	2.63	1.77	-0.63	0.00	0.00
N4BP2L1	90634	5.13	4.91	1.24	4.12	-0.71	0.00	0.00
NAIF1	203245	3.68	3.39	2.87	2.32	-0.63	0.00	0.00
NANOS1	340719	0.4	0.35	0.18	0.21	-0.92	0.00	0.00
NCMAP	400746	5.14	4.23	1.48	2.63	-1.20	0.00	0.00
NDRG4	65009	1.45	0.86	0.18	0.9	-0.90	0.00	0.00
NDUFA4L2	56901	3.09	0.42	0.29	0.35	-2.43	0.00	0.00
NECTIN4	81607	2.42	1.36	1	0.98	-0.99	0.00	0.00
NEGR1	257194	0.03	0.1	0	0.05	-4.16	0.00	0.00
NEO1	4756	0.7	0.72	0.13	0.29	-1.94	0.00	0.00
NFE2L3	9603	2.84	1.96	0.08	0.88	-1.92	0.00	0.00
NGFR	4804	0.87	0.08	0.05	0.03	-3.61	0.00	0.00
NKG7	4818	0.96	0.54	0.74	0.41	-2.42	0.00	0.00
NKX6-3	157848	0.63	0.51	0.29	0.11	-1.49	0.00	0.00
NMNAT3	349565	12.71	12.39	7.3	8.82	-0.65	0.00	0.00
NOA1	84273	19.68	19.12	9.86	16.72	-0.59	0.00	0.00
NOL4	8715	0.17	0.15	0	0.05	-2.71	0.00	0.00
NOS2	4843	2.94	6.4	3.62	2.15	-0.66	0.00	0.00
NOTCH1	4851	0.8	0.59	0.36	0.43	-0.75	0.00	0.00
NOTUM	147111	201.09	145.41	102.1	105.97	-0.73	0.00	0.00
NOXA1	10811	1.93	1.11	0.79	1.09	-0.66	0.00	0.00
NPAS2	4862	26.71	25.64	14.8	17.9	-0.75	0.00	0.00
NPIPA1	9284	9.84	7.66	5.31	6.14	-0.61	0.00	0.00
NPIPA5	100288332	0	0	0	0	-0.63	0.00	0.00
NPIPA7	101059938	0	0	0	0.01	-1.24	0.00	0.00
NQO1	1728	0	0	0	0.02	-0.75	0.00	0.00
NR2F1-AS1	441094	0.89	0.89	0.47	0.31	-1.07	0.00	0.00
NRIP2	83714	0.14	0.03	0	0.01	-4.29	0.00	0.00
NT5DC3	51559	3.74	3.72	1.04	3.34	-0.59	0.00	0.00
NT5E	4907	2.14	4.19	0.73	0.38	-2.51	0.00	0.00

NT5M	56953	5.22	1.87	2.19	1.38	-0.97	0.00	0.00
NTSR1	4923	0.6	0.16	0	0.13	-2.51	0.00	0.00
NUPR1	26471	100.94	67.9	51.66	40.43	-0.87	0.00	0.00
OAS1	4938	11	13.83	7.9	8.7	-0.66	0.00	0.00
ODF2	4957	8.43	7.29	2.66	7.8	-0.66	0.00	0.00
OGDHL	55753	0.44	0.35	0.09	0.23	-1.31	0.00	0.00
OSBPL10	114884	8.64	7.32	1.67	5.68	-0.91	0.00	0.00
OSBPL2	9885	14.54	18.41	7.35	17.25	-0.72	0.00	0.00
OSCAR	126014	2.9	0.39	0.37	0.54	-1.79	0.00	0.00
OSER1-DT	100505783	3.65	1.62	0.08	1.4	-1.75	0.00	0.00
OTUB2	78990	2.03	0.99	0.54	1.43	-0.61	0.00	0.00
OXT	5020	1.73	0.67	0.13	0	-4.37	0.00	0.00
PACS2	23241	1.9	1.51	1.22	1.3	-0.63	0.00	0.00
PAK1	5058	0.9	0.72	0.39	0.23	-1.53	0.00	0.00
PALLD	23022	1.56	0.59	0.05	0.92	-1.02	0.00	0.00
PAX6	5080	3.77	3.17	0.25	0.47	-2.61	0.00	0.00
PBXIP1	57326	12.38	10.37	5.41	8.97	-0.64	0.00	0.00
PCOLCE	5118	5.68	5.14	3.81	2.26	-0.83	0.00	0.00
PCYOX1L	78991	3.4	0.81	0.09	1.23	-1.47	0.00	0.00
PDCD1	5133	0.77	0.08	0.08	0.08	-3.04	0.00	0.00
PDCD4-AS1	282997	0.2	0.3	0.44	0.32	-4.69	0.00	0.00
PDE9A	5152	6.17	2.26	0.89	2.92	-1.09	0.00	0.00
PDGFA	5154	35.49	37.48	8.56	39.49	-1.64	0.00	0.00
PDLIM4	8572	2.98	0.34	0.47	1.26	-1.04	0.00	0.00
PFKFB2	5208	1.57	2.78	0.73	1.26	-1.05	0.00	0.00
PGC	5225	32.87	30.34	4.47	8.89	-2.25	0.00	0.00
PHKA2	5256	7.9	7.34	3.4	5.6	-0.76	0.00	0.00
PHYKPL	85007	16.07	10.08	8.97	7.83	-0.68	0.00	0.00
PIEZO2	63895	0.71	0.77	0.01	0.04	-4.80	0.00	0.00
PIGW	284098	2.72	4.61	1.06	3.17	-0.73	0.00	0.00
PIP4P2	55529	2.39	4.02	1.91	2.36	-0.66	0.00	0.00
PIP5K1B	8395	0	0	0.04	0.03	-2.09	0.00	0.00
PITPNA-AS1	100306951	0.43	0.63	0	0.39	-1.47	0.00	0.00

PITPNM2-AS1	100507091	1.93	0.83	0.69	0.31	-1.42	0.00	0.00
PLA2G16	11145	2.28	1.92	0.17	0.2	-3.24	0.00	0.00
PLA2G2A	5320	361.08	239.25	9.64	109.29	-2.34	0.00	0.00
PLA2G4B	100137049	1.3	0	0	0	-7.04	0.00	0.00
PLPP1	8611	3.08	3.77	2.15	1.76	-0.64	0.00	0.00
PLSCR3	57048	7.39	4.34	4.7	3.1	-0.59	0.00	0.00
PLTP	5360	32.91	20.08	15.1	14.08	-0.71	0.00	0.00
PLXNB3	5365	3.78	2.97	2.13	1.77	-0.75	0.00	0.00
PNCK	139728	8.7	1.23	1.78	2.64	-1.21	0.00	0.00
PNPLA3	80339	4.25	6.21	5.04	2.06	-0.72	0.00	0.00
POC1B-GALNT4	100528030	0.28	0.29	0	0.19	-1.72	0.00	0.00
PPARGC1A	10891	0.14	0.37	0	0	-6.95	0.00	0.00
PPIP5K1P1-CATSPER2	110006325	0.37	0.34	0.03	0.19	-1.69	0.00	0.00
PPM1J	333926	1.07	0.23	0.19	0.04	-2.37	0.00	0.00
PPM1L	151742	0.24	0.39	0.18	0.26	-0.88	0.00	0.00
PPP1R14D	54866	2.57	1.32	0.15	0.2	-3.61	0.00	0.00
PPP1R3E	90673	1.18	1.09	0.37	0.99	-0.79	0.00	0.00
PPT1	5538	79.54	66.29	20.76	44.94	-1.15	0.00	0.00
PQLC3	130814	2.7	1.76	0.85	1.11	-1.27	0.00	0.00
PRF1	5551	2.64	0.92	0.86	1.28	-0.74	0.00	0.00
PRIMA1	145270	0	0	0	0	-4.13	0.00	0.00
PRKCA	5578	7.75	10.81	1.9	8.4	-0.60	0.00	0.00
PRPF40B	25766	3.22	2.21	1.25	2.25	-0.63	0.00	0.00
PRR29	92340	1.31	0.49	0.06	0.35	-2.01	0.00	0.00
PRSS22	64063	0.92	0.62	0.22	0.08	-2.50	0.00	0.00
PRSS30P	124221	0.61	0.33	0.2	0.1	-1.63	0.00	0.00
PRSS56	646960	0	0	0	0	-3.95	0.00	0.00
PRSS8	5652	25.03	11	10.01	3.77	-1.37	0.00	0.00
PSAT1	29968	152.99	114.11	71.47	101.11	-0.63	0.00	0.00
PTAFR	5724	2	0.85	0.19	0.47	-2.13	0.00	0.00
PTGDS	5730	10.32	5.95	1.46	2.81	-1.98	0.00	0.00
PTGES3L	100885848	0.42	1.11	0	0.42	-1.89	0.00	0.00
PTGFRN	5738	7.05	6.69	1.18	3.16	-1.66	0.00	0.00

PTK6	5753	1.63	1.33	0.85	0.48	-1.26	0.00	0.00
PTPN3	5774	4.56	4.07	3.68	8.52	-0.72	0.00	0.00
PTPRJ	5795	0.96	1.23	0.1	0.03	-3.75	0.00	0.00
RAB15	376267	9.01	9.98	4.36	3.67	-1.26	0.00	0.00
RAB25	57111	10.5	9.5	1.21	0.89	-3.25	0.00	0.00
RAB2B	84932	1.5	0.83	0.16	1.2	-0.81	0.00	0.00
RALGPS1	9649	1.34	1.33	0.69	0.95	-0.89	0.00	0.00
RAMP2	10266	7.58	4.32	4	2.72	-0.79	0.00	0.00
RAPGEF3	10411	2.46	1.91	1.44	0.88	-1.01	0.00	0.00
RARRES2	5919	101.2	63.17	50.4	53.22	-0.64	0.00	0.00
RASA4	10156	0.87	0.85	0.42	0.21	-1.44	0.00	0.00
RASAL1	8437	0.51	0.55	0.35	0.07	-1.28	0.00	0.00
RASD1	51655	12.64	6.35	4.42	5.2	-0.98	0.00	0.00
RASSF3	283349	3.71	4.34	1.27	4.01	-0.61	0.00	0.00
RBM47	54502	1.62	3.28	1.15	1.72	-0.81	0.00	0.00
RBMS2	5939	4.78	3.25	2.2	2.99	-0.76	0.00	0.00
RBP1	5947	21.17	8.29	3.6	7.23	-1.40	0.00	0.00
RBPMS2	348093	8.52	9.07	4.72	3.54	-1.06	0.00	0.00
RDH10	157506	8.8	10.5	3.2	7.84	-1.14	0.00	0.00
RENBP	5973	8.6	7.18	5.03	3.4	-0.85	0.00	0.00
RGMB	285704	3.53	2.75	1.24	2.34	-0.71	0.00	0.00
RGR	5995	0.65	0.28	0	0	-5.63	0.00	0.00
RGS10	6001	21.66	13.97	8.04	9.24	-1.03	0.00	0.00
RHPN1	114822	3.58	2.25	1.46	1.93	-0.77	0.00	0.00
RIMBP3C	150221	0.21	0.04	0	0.02	-3.92	0.00	0.00
RIPK4	54101	3.32	3.11	1.9	1.94	-0.74	0.00	0.00
RLBP1	6017	0.4	0.18	0.03	0.02	-3.21	0.00	0.00
RNF144A-AS1	386597	0.34	0.52	0.14	0.31	-0.94	0.00	0.00
RNF157	114804	22.54	19.28	15.12	13.62	-0.63	0.00	0.00
RORC	6097	9.1	9.29	5.53	4.27	-0.90	0.00	0.00
RPLP0P2	113157	0.34	0.21	0.14	0.09	-1.48	0.00	0.00
RSPH10B	222967	0.06	0.07	0	0.04	-1.71	0.00	0.00
RTBDN	83546	3.77	2.6	2.32	1.38	-0.78	0.00	0.00

RTL8C	8933	19.46	16.06	0.09	0.05	-8.01	0.00	0.00
RTN4RL2	349667	28.41	12.48	13.76	8.55	-0.87	0.00	0.00
RUNX1	861	4.57	4.21	2.55	2.71	-0.65	0.00	0.00
S100A14	57402	34.97	25.48	7.45	6.76	-2.08	0.00	0.00
S100A3	6274	0	0	0	0	-0.91	0.00	0.00
S100P	6286	236.76	161.91	151.07	109.14	-0.61	0.00	0.00
SALL1	6299	0.33	0.29	0	0.08	-2.98	0.00	0.00
SAMD11	148398	0	0	0	0.05	-1.69	0.00	0.00
SAT1	6303	11.62	11.68	10.46	14.87	-0.75	0.00	0.00
SBK2	646643	5.27	1.62	1.24	2.76	-0.86	0.00	0.00
SBK3	100130827	0.04	0	0	0	-1.10	0.00	0.00
SCN1A	6323	0.56	0.62	0.07	0.55	-0.99	0.00	0.00
SCN1B	6324	3.89	4.85	2.66	1.05	-1.26	0.00	0.00
SCNN1A	6337	20.33	15.46	6.21	5.5	-1.47	0.00	0.00
SDCBP2	27111	7.11	12.71	8.13	2.34	-0.81	0.00	0.00
SEC14L6	730005	1.24	0.45	0.08	0.48	-1.74	0.00	0.00
SEL1L3	23231	9.58	6.7	0.83	1.32	-2.93	0.00	0.00
SEMA6A	57556	1.33	1.84	0.03	0.16	-4.93	0.00	0.00
SERINC2	347735	56.8	31.72	28.19	13.97	-1.06	0.00	0.00
SERPINA11	256394	41.87	19.39	14.09	14.86	-1.09	0.00	0.00
SERPINA3	12	665.9	122.86	43.46	81.74	-2.66	0.00	0.00
SERPINB8	5271	1.48	2.9	1.71	0.98	-0.78	0.00	0.00
SERPINE2	5270	5.72	3.63	0.47	2.27	-1.73	0.00	0.00
SEZ6L2	26470	27.57	27.19	7.06	2.78	-2.46	0.00	0.00
SFRP5	6425	2.29	1.86	0	0.06	-6.15	0.00	0.00
SFT2D3	84826	10.71	9.42	7.15	5.68	-0.65	0.00	0.00
SGPL1	8879	8.71	8.54	3.92	7.35	-0.66	0.00	0.00
SH2D3A	10045	5.16	3.92	2.71	6.56	-1.04	0.00	0.00
SH2D4A	63898	0.33	0.35	0.02	0.03	-4.45	0.00	0.00
SH2D5	400745	5.06	5.12	4.13	1.35	-0.89	0.00	0.00
SH3BGRL	6451	2.38	2.92	0.28	0.77	-2.21	0.00	0.00
SH3RF1	57630	1.99	2.74	0.98	2.04	-0.70	0.00	0.00
SHC4	399694	0	0	0	0	-6.33	0.00	0.00

SHE	126669	0.05	0.04	0.01	0.01	-3.03	0.00	0.00
SHISA4	149345	2.45	0.51	0.3	0.34	-2.27	0.00	0.00
SHISA8	440829	0.34	0.03	0.12	0	-1.57	0.00	0.00
SHROOM2	357	0.7	0.63	0.04	0	-4.62	0.00	0.00
SIAE	54414	2.12	3.51	0.63	2.69	-0.85	0.00	0.00
SIM2	6493	1.11	1.42	0.81	0.67	-0.94	0.00	0.00
SIN3A	25942	2.26	1.06	0.76	1.08	-0.69	0.00	0.00
SLC12A3	6559	0.59	0.28	0.06	0.48	-1.02	0.00	0.00
SLC13A3	64849	21.21	12.35	9.26	13.57	-0.77	0.00	0.00
SLC17A2	10246	8.69	7.62	4.17	4.12	-0.93	0.00	0.00
SLC22A31	146429	22.37	27.6	18.58	2.49	-1.24	0.00	0.00
SLC25A25	114789	6.14	7.16	3.1	2.36	-1.27	0.00	0.00
SLC26A9	115019	0.36	0.87	0.16	0	-2.87	0.00	0.00
SLC27A1	376497	4.23	2.17	1.3	1.92	-1.01	0.00	0.00
SLC2A4	6517	4.74	3.09	1.76	2.75	-0.80	0.00	0.00
SLC30A1	7779	10.22	11.02	4.54	7.98	-0.73	0.00	0.00
SLC41A1	254428	0.1	0.06	0	0	-4.85	0.00	0.00
SLC44A3	126969	1.98	3.45	0.92	1.97	-1.11	0.00	0.00
SLC47A1	55244	30.51	32.7	14.14	19.17	-0.93	0.00	0.00
SLC4A11	83959	22.15	30.73	41.65	16.93	-2.01	0.00	0.00
SLC52A3	113278	0.77	0.48	0.4	0.1	-1.28	0.00	0.00
SLC6A14	11254	0.92	0.75	0.08	0.52	-2.04	0.00	0.00
SLC7A1	6541	22.96	15.38	7.63	16.7	-0.65	0.00	0.00
SMARCA2	6595	2.6	1.94	0.85	1.75	-0.61	0.00	0.00
SMIM11A	54065	2.42	2.49	0.27	1.2	-1.48	0.00	0.00
SMPDL3B	27293	3.22	2.78	0.47	0.21	-2.91	0.00	0.00
SNAI3	333929	1.41	1.26	0.74	0.45	-1.16	0.00	0.00
SNHG16	100507246	0.74	0.5	0.12	0.39	-1.30	0.00	0.00
SOCS3	9021	3.03	0.81	0.06	0.34	-3.28	0.00	0.00
SORBS1	10580	1.6	2.03	0.47	1.22	-1.13	0.00	0.00
SOWAHB	345079	0.14	0.37	0.06	0.1	-1.76	0.00	0.00
SOX9	6662	32.29	25.55	6.93	18.16	-1.21	0.00	0.00
SPATA21	374955	6.38	5.28	4.89	6.32	-0.75	0.00	0.00

SPECC1	92521	1.57	2.33	0.23	0.16	-3.36	0.00	0.00
SPINT1	6692	20.33	16.24	5.39	7.07	-2.11	0.00	0.00
SPN	6693	0.62	0.33	0.07	0.27	-1.49	0.00	0.00
SPOCK2	9806	28.91	46.98	23.77	7.06	-1.36	0.00	0.00
SPON2	10417	9.39	9.64	6.26	2.39	-1.10	0.00	0.00
SPTBN1	6711	0	0	0	0	-0.61	0.00	0.00
SPTSSB	165679	0.77	2.06	0.02	0.1	-5.38	0.00	0.00
SPX	80763	5.45	6.05	4.2	11.3	-4.16	0.00	0.00
SQOR	58472	13.66	14.67	4.38	5.28	-1.54	0.00	0.00
SRRM3	222183	0.47	0.44	0.15	0.19	-1.53	0.00	0.00
SSC4D	136853	14.47	5.29	5.6	5.02	-0.98	0.00	0.00
STAB1	23166	0.11	0.09	0.01	0.01	-3.26	0.00	0.00
STAG3	10734	3.39	3.1	1.08	1.4	-0.77	0.00	0.00
STAT3	6774	23.44	21.33	9.44	18.25	-0.82	0.00	0.00
STC2	8614	11.51	12.09	10.21	22.59	-0.65	0.00	0.00
STK4-AS1	100505826	0.2	0.2	0.05	0.08	-1.65	0.00	0.00
STN1	79991	11.58	7.88	5.08	7.3	-0.66	0.00	0.00
STOM	2040	0.55	0	0	0	-6.04	0.00	0.00
STPG3	441476	2.09	0.4	0.2	0.67	-1.45	0.00	0.00
STRA6	64220	3.03	1.31	0	0.1	-5.83	0.00	0.00
STXBP1	6812	7.58	7.07	5.43	4.06	-1.39	0.00	0.00
SULF2	55959	1.8	1.19	0.53	0.56	-1.46	0.00	0.00
SULT1C2	6819	1.58	2.58	1.19	0.28	-0.97	0.00	0.00
SULT2B1	6820	0.15	0.24	0.18	0.19	-2.74	0.00	0.00
SUSD3	203328	7.88	5.08	0.45	0.76	-3.47	0.00	0.00
SYNE2	23224	0.42	0.4	0.01	0.06	-2.69	0.00	0.00
SYNE3	161176	1.34	0.89	0.31	0.21	-2.83	0.00	0.00
SYNGR3	9143	4.63	2.14	1.09	1.56	-1.29	0.00	0.00
SYT5	6861	0.53	0.06	0.02	0.03	-3.46	0.00	0.00
TAGLN2	8407	13.67	6.22	1.64	5.64	-1.40	0.00	0.00
TANC1	85461	1.15	1.1	0.35	0.9	-1.98	0.00	0.00
TAPBP	6892	18.29	13.82	8.21	11.13	-0.73	0.00	0.00
TBC1D30	23329	2.21	2.16	1.07	0.9	-1.15	0.00	0.00

TBC1D3L	101060376	2.69	1.84	0.46	1.78	-1.06	0.00	0.00
TBX10	347853	6.75	9.63	7.61	2.28	-0.79	0.00	0.00
TCP10L	140290	0.41	0.41	0.21	0.08	-1.48	0.00	0.00
TCP11L2	255394	3.46	1.9	0.09	1.85	-1.35	0.00	0.00
TDRKH	11022	2.59	2.31	0.98	1.72	-0.88	0.00	0.00
TEP1	7011	1.6	1.71	0.31	1.3	-0.91	0.00	0.00
TEX30	93081	0.78	0.86	0.03	0.14	-2.74	0.00	0.00
TFEB	7942	3.32	2.89	2.05	0.62	-0.99	0.00	0.00
TGFA	7039	4.79	4.9	0.22	0.64	-3.48	0.00	0.00
TGM2	7052	30.67	14.89	8.75	11.25	-1.26	0.00	0.00
TIAF1	9220	6.19	3.33	0.12	0.34	-4.40	0.00	0.00
TIE1	7075	0.31	0.13	0.05	0.06	-2.19	0.00	0.00
TIMP2	7077	11.54	10.41	3.34	3.85	-1.60	0.00	0.00
TINAGL1	64129	20.08	9.13	6.93	6.43	-1.11	0.00	0.00
TJP3	27134	6.16	11.33	8.47	1.86	-0.74	0.00	0.00
TLDC2	140711	8.02	11.51	5.35	6.88	-0.67	0.00	0.00
TM4SF19	116211	5.31	2.63	2.38	1.53	-1.04	0.00	0.00
TM4SF19-TCTEX1D2	100534611	5.08	3.12	3.56	2.66	-0.66	0.00	0.00
TMC4	147798	4.31	2.56	1.63	0.43	-1.73	0.00	0.00
TMC7	79905	2.31	1.31	0.54	0.98	-1.19	0.00	0.00
TMCO6	55374	8.28	4.08	4.31	4.14	-0.58	0.00	0.00
TMEM125	128218	0.16	0.29	0	0.01	-4.04	0.00	0.00
TMEM140	55281	4.45	5.12	2.6	2.67	-0.86	0.00	0.00
TMEM151A	256472	1.79	1.91	0.61	0.37	-1.91	0.00	0.00
TMEM159	57146	1.1	1.31	0.43	0.15	-1.74	0.00	0.00
TMEM173	340061	1.17	1.17	0.27	0.93	-1.13	0.00	0.00
TMEM268	203197	4.34	2.72	1.64	2.67	-0.67	0.00	0.00
TMEM45B	120224	97.85	80.99	49.25	51.76	-0.82	0.00	0.00
TMEM56	148534	0.29	0.65	0.08	0.33	-1.22	0.00	0.00
TMEM59L	25789	47.34	13.41	8.16	6.74	-2.02	0.00	0.00
TMEM9B-AS1	493900	1.11	1.23	0.74	0.71	-0.71	0.00	0.00
TNFAIP1	7126	0.6	1.08	0.16	0.61	-0.82	0.00	0.00
TNFRSF11A	8792	0.91	0.86	0.56	1.26	-0.69	0.00	0.00

TNFRSF13C	115650	0.61	0.17	0.38	0.04	-2.16	0.00	0.00
TNFRSF21	27242	31.92	15.48	6.51	23.92	-0.66	0.00	0.00
TNFSF14	8740	0.39	0.16	0.05	0.25	-1.28	0.00	0.00
TNS4	84951	0.87	0.47	0.04	0.16	-3.38	0.00	0.00
TOB1-AS1	400604	0	0	0	0	-1.83	0.00	0.00
TOX3	27324	1.83	1.23	0	0.05	-5.19	0.00	0.00
TPPP	11076	0.18	0.15	0	0.06	-2.58	0.00	0.00
TPRXL	348825	0.04	0.04	0.02	0.01	-1.43	0.00	0.00
TRAPPC6A	79090	17.68	8.18	7.82	6.71	-0.76	0.00	0.00
TRIM29	23650	0.79	0.64	0.07	0.1	-2.63	0.00	0.00
TRIM50	135892	7.88	13.18	10.47	2.87	-0.65	0.00	0.00
TRPV3	162514	0.53	0.89	0.23	0.18	-1.75	0.00	0.00
TRPV4	59341	4.85	3.54	2.27	1.15	-1.26	0.00	0.00
TRUB2	26995	22.23	26.16	17.95	11.94	-0.65	0.00	0.00
TSPAN1	10103	7.31	5.81	2.63	2.45	-1.32	0.00	0.00
TSPAN31	6302	6.96	4.87	3.28	4.15	-0.62	0.00	0.00
TSPAN8	7103	2.69	3.92	0.68	0.36	-2.71	0.00	0.00
TSPOAP1-AS1	100506779	0.45	0.25	0.02	0.26	-1.36	0.00	0.00
TTC28-AS1	284900	2.61	2.91	1.3	2.89	-1.32	0.00	0.00
TTC39C	125488	11.51	14.71	9.49	7.79	-0.60	0.00	0.00
TTC7B	145567	1.09	0.52	0.45	0.52	-0.80	0.00	0.00
TTYH1	57348	2.93	1.24	0.31	0.24	-2.97	0.00	0.00
TUB	7275	0.1	0.08	0	0.03	-3.43	0.00	0.00
TUBB1	81027	0.88	0.67	0.18	0.85	-0.91	0.00	0.00
TUBB6	84617	17.36	9.47	5.77	9.09	-0.83	0.00	0.00
TUBBP5	643224	0.54	1.02	0.09	0.1	-3.09	0.00	0.00
TUSC8	400128	0.43	0.5	0.2	0.33	-0.83	0.00	0.00
TUT1	64852	6.7	1.36	0.9	3.3	-0.95	0.00	0.00
TXNIP	10628	38.65	22.44	7.59	30.68	-0.66	0.00	0.00
UBASH3B	84959	2.03	2.5	0.76	1.29	-1.14	0.00	0.00
UBXN10	127733	0.55	0.38	0.05	0.42	-1.09	0.00	0.00
UCA1	652995	2.37	2.08	1.01	1.44	-0.86	0.00	0.00
UGT1A6	54578	1.79	2.5	0.17	0.93	-1.74	0.00	0.00

UGT1A7	54577	0.3	0.14	0	0	-5.25	0.00	0.00
UNC5CL	222643	0.03	0.13	0.01	0.04	-0.65	0.00	0.00
UNC93A	54346	7.22	8.63	3.54	5.26	-0.86	0.00	0.00
URB1	9875	6.88	5.45	1.88	6.15	-0.68	0.00	0.00
UROC1	131669	1.74	2.13	1.54	0.67	-0.71	0.00	0.00
USHBP1	83878	1.32	0.6	0.25	0.49	-1.53	0.00	0.00
USP2	9099	6.29	7.04	3.87	4.13	-0.58	0.00	0.00
USP3	9960	6.89	5.55	1.58	5.74	-0.65	0.00	0.00
VAV3	10451	0.54	1.63	0.31	0.45	-1.49	0.00	0.00
VDR	7421	0.35	0.54	0.24	0.56	-0.99	0.00	0.00
VPS35L	57020	0.4	1.18	0.02	0.06	-4.48	0.00	0.00
VPS37B	79720	7.94	7.5	5.64	4.51	-0.64	0.00	0.00
WAS	7454	0.37	0.23	0.03	0.05	-2.79	0.00	0.00
WBP2	23558	1.77	4.17	1.45	0.3	-1.70	0.00	0.00
WDR97	340390	0.14	0.1	0	0.04	-3.04	0.00	0.00
WNT7B	7477	1.85	0.51	0.6	0.39	-1.99	0.00	0.00
WNT8B	7479	6.37	0	0.02	0.13	-3.83	0.00	0.00
XIRP2	129446	0.21	0.21	0.04	0.04	-2.37	0.00	0.00
YPEL5	51646	6.73	7.64	2.29	7.21	-0.59	0.00	0.00
YRDC	79693	18.74	16.88	9.42	10.05	-0.87	0.00	0.00
ZAP70	7535	3.25	2.09	1.94	0.87	-0.75	0.00	0.00
ZBED6CL	113763	0	0	0	0	-1.00	0.00	0.00
ZBTB25	7597	2.45	2.11	0.42	1.67	-1.10	0.00	0.00
ZFP36	7538	22.12	17.01	10.78	11.64	-0.80	0.00	0.00
ZHX1	11244	0.29	0.56	0.24	0.08	-2.10	0.00	0.00
ZMIZ1-AS1	283050	1.36	0.65	0.46	0.58	-0.95	0.00	0.00
ZMYND15	84225	2.16	1.9	1.09	0.8	-1.23	0.00	0.00
ZNF16	7564	3.61	3.23	1.51	2.87	-0.64	0.00	0.00
ZNF252P-AS1	286103	0.03	0.24	0	0.07	-1.84	0.00	0.00
ZNF419	79744	1.53	0.95	0.56	0.72	-0.93	0.00	0.00
ZNF425	155054	1.03	0.31	0.14	0.59	-0.82	0.00	0.00
ZNF460	10794	0.57	0.48	0.19	0.45	-0.60	0.00	0.00
ZNF529-AS1	101927599	0.38	0.07	0.01	0	-4.67	0.00	0.00

ZNF542P	147947	0.13	0.51	0	0.02	-5.33	0.00	0.00
ZNF703	80139	25.66	23.15	19.57	10.42	-0.69	0.00	0.00
ZNF8	7554	0.41	0.23	0	0.14	-2.24	0.00	0.00
ZNF862	643641	0.59	0.21	0.06	0.27	-0.97	0.00	0.00
ZNHIT3	9326	19.03	20.68	12.59	11.4	-0.75	0.00	0.00
ZSWIM6	57688	0.15	0.26	0.01	0.04	-2.34	0.00	0.00

Table S1c. Genes up-regulated (>1.5-fold change, q-value < 0.001) after miR-27a knock out

Symobol	GeneID	27aKO1 FPKM	27aKO2 FPKM	Scr1 FPKM	Scr2 FPKM	log2Ratio 23aKO/Scr	q-value	p-value
AADAC	13	4.9	5.03	0.36	1.31	2.57	0.00	0.00
AARS2	57505	7.23	7.27	5.33	4.11	0.59	0.00	0.00
AASDH	132949	1.53	1.9	1.23	1.25	0.76	0.00	0.00
ABAT	18	39.23	39.84	12.89	18.89	1.31	0.00	0.00
ABCB10	23456	3.71	3.51	1.5	2.89	0.74	0.00	0.00
ABCB7	22	6.72	6.68	4.4	4.25	0.66	0.00	0.00
ABHD1	84696	1.13	0.88	0.47	0.16	1.46	0.00	0.00
ABHD17B	51104	2.9	3.07	1.09	2.65	0.67	0.00	0.00
ABHD18	80167	1.32	1.86	0.9	1.47	0.66	0.00	0.00
ABLIM1	3983	26.84	24.35	14.15	15.07	0.81	0.00	0.00
ABRAXAS1	84142	2.21	2.11	1.21	1.76	0.63	0.00	0.00
ABRAXAS2	23172	2.04	1.81	0.87	1.53	0.68	0.00	0.00
ACAD10	80724	6.62	7.24	4.42	3.9	0.69	0.00	0.00
ACAT1	38	48.29	48.63	28.55	29.33	0.77	0.00	0.00
ACER3	55331	1.62	1.5	0.69	1.07	0.63	0.00	0.00
ACOT11	26027	10.38	9.66	3.71	3.41	1.51	0.00	0.00
ACSL4	2182	25.78	27.67	10.18	20.33	0.80	0.00	0.00
ACSM1	116285	1.45	1.71	0.05	0.29	3.88	0.00	0.00
ACSM2B	348158	3.58	3.87	2.02	2.63	0.77	0.00	0.00
ACSM3	6296	2.49	2.69	1.43	1.22	1.24	0.00	0.00
ACSM5	54988	4.97	5.02	2.29	2.15	0.98	0.00	0.00
ACSS1	84532	65.37	61.69	23.45	32.46	1.21	0.00	0.00
ACTN4	81	226.35	221.47	141.7	140.51	0.66	0.00	0.00
ACTR6	64431	1.58	1.49	0.66	0.9	0.99	0.00	0.00
ACTR8	93973	2.73	2.74	1.66	1.75	0.60	0.00	0.00
ACVR2A	92	1.03	1.46	0.31	0.88	1.14	0.00	0.00
ACVR2B	93	0.37	0.49	0.53	0.37	0.69	0.00	0.00
ADAL	161823	2.72	2.65	1.1	1.11	1.08	0.00	0.00
ADAM17	6868	5.74	5.13	2.96	2.93	0.80	0.00	0.00

ADAMTS2	9509	24.32	24.88	17.54	14.56	1.19	0.00	0.00
ADAP2	55803	0.87	1.6	0.13	0.04	3.66	0.00	0.00
ADGRA3	166647	17.06	19.24	11.79	11.91	0.63	0.00	0.00
ADH4	127	8.37	8.06	1.1	2.85	2.07	0.00	0.00
ADH6	130	37.23	38.61	13	19.88	1.24	0.00	0.00
ADM5	199800	1.79	2.66	1.5	1.37	0.61	0.00	0.00
ADNP	23394	12.75	12.53	8.32	8.61	0.60	0.00	0.00
ADNP2	22850	6.95	6.92	4.04	4.44	0.69	0.00	0.00
ADPRHL1	113622	3.32	3.76	1.86	2.03	0.82	0.00	0.00
ADSS	159	6.97	7.45	4.45	4.55	0.67	0.00	0.00
AEBP2	121536	2.78	2.88	1.36	2.17	0.64	0.00	0.00
AFDN	4301	16.58	15.92	6.64	6.35	1.31	0.00	0.00
AFG1L	246269	2.12	2.26	2	2.17	1.16	0.00	0.00
AFM	173	0.29	0.36	0.07	0.14	2.08	0.00	0.00
AGAP4	119016	1.43	1.06	0.25	0.77	1.20	0.00	0.00
AGBL3	340351	1.65	1.49	0.31	1.03	0.92	0.00	0.00
AGGF1	55109	1.74	1.61	0.55	1.19	0.95	0.00	0.00
AGTPBP1	23287	1.14	1.16	0.62	0.92	0.82	0.00	0.00
AHNAK	79026	9.11	9.53	6.32	7.09	0.61	0.00	0.00
AHR	196	3.36	2.46	1.36	1.64	0.86	0.00	0.00
AIFM3	150209	7.47	8.88	4.71	2.44	1.20	0.00	0.00
AKAP13	11214	2.05	1.82	1.44	1.26	0.59	0.00	0.00
AKAP2	11217	2.74	1.89	1.37	1.07	0.92	0.00	0.00
AKAP7	9465	2.76	2.57	1.28	2.36	0.59	0.00	0.00
AKR1D1	6718	3.21	3.21	1.17	2.03	1.00	0.00	0.00
AKR7A2	8574	78.12	83.21	52.15	51.83	0.76	0.00	0.00
ALDH1A1	216	244.15	241.16	79.15	89.15	1.53	0.00	0.00
ALDH3A2	224	46.09	47.27	20.33	28.48	0.93	0.00	0.00
ALDH5A1	7915	27.89	28.31	13.91	19.37	0.75	0.00	0.00
ALDH6A1	4329	10.17	10.16	5.39	7.91	0.62	0.00	0.00
ALG10	84920	0.82	0.78	0.23	0.57	0.94	0.00	0.00
ALG10B	144245	0.25	0.31	0.11	0.13	1.22	0.00	0.00
ALG11	440138	1.34	1.52	0.98	0.89	1.18	0.00	0.00

ALG6	29929	1.97	1.76	0.8	1.2	0.75	0.00	0.00
ALOX12P2	245	0.35	0.34	0.15	0.17	0.93	0.00	0.00
ALOX15	246	0	0	0	0.02	1.72	0.00	0.00
ALPK1	80216	1.64	1.62	1.02	0.59	0.75	0.00	0.00
ALPK2	115701	2.06	1.78	1.75	0.78	0.65	0.00	0.00
AMDHD1	144193	1.77	1.42	0.62	1.02	0.98	0.00	0.00
AMIGO1	57463	1.09	1.1	0.64	0.49	0.94	0.00	0.00
AMMECR1	9949	1.23	0.86	0.65	0.65	0.61	0.00	0.00
AMZ1	155185	0.07	1.13	0.02	0.43	1.25	0.00	0.00
ANAPC16	119504	29.25	32.4	20.41	19.27	0.64	0.00	0.00
ANGEL2	90806	2.03	1.98	1.13	1.17	0.77	0.00	0.00
ANGPT2	285	0.15	0.39	0	0	6.16	0.00	0.00
ANGPTL1	9068	1.05	0.95	0.23	0.18	2.26	0.00	0.00
ANKHD1-EIF4EBP3	404734	7.15	7.48	4.34	5.25	0.61	0.00	0.00
ANKRD11	29123	10.71	11.06	7.91	7.24	0.58	0.00	0.00
ANKRD13C	81573	3.48	2.72	1.64	2.39	0.58	0.00	0.00
ANKRD17	26057	11.42	10.91	6.07	8.25	0.68	0.00	0.00
ANKRD23	200539	0.82	1.03	0.44	0.71	1.01	0.00	0.00
ANKRD46	157567	3.78	4.15	2.19	2.49	0.73	0.00	0.00
ANO6	196527	17.63	17.71	8.67	11.15	0.84	0.00	0.00
AP1B1	162	38.18	34.86	35.02	36.89	0.77	0.00	0.00
APAF1	317	1.29	1.3	0.57	0.91	0.73	0.00	0.00
API5	8539	6.53	8.64	1.46	0.43	2.88	0.00	0.00
APOBEC3B	9582	15.71	14.8	12.5	7.61	0.60	0.00	0.00
APOBEC3G	60489	0.95	0.74	0.6	0.11	1.37	0.00	0.00
APOF	319	10.71	11.23	3.89	8.52	0.82	0.00	0.00
APOH	350	1698.25	1758.52	876.24	1232.32	0.71	0.00	0.00
APOL2	23780	11.83	11.35	3.7	4.3	1.52	0.00	0.00
APOL3	80833	2.12	2.55	0.65	0.37	2.24	0.00	0.00
APP	351	160.27	154.06	84.99	64.99	1.06	0.00	0.00
APPBP2	10513	3.32	3.58	2.33	2.83	0.66	0.00	0.00
APPL1	26060	2.17	2.32	1	2.02	0.75	0.00	0.00
ARAP1	116985	40.78	40.22	21.27	19.73	0.98	0.00	0.00

ARFIP1	27236	2.99	2.97	1.55	2.24	0.59	0.00	0.00
ARHGAP18	93663	12.05	13.45	6.1	10.83	0.58	0.00	0.00
ARHGAP19	84986	2.81	2.65	1.25	1.53	0.97	0.00	0.00
ARHGAP44	9912	2.91	3.15	1.69	1.5	0.88	0.00	0.00
ARHGEF34P	728377	0.14	0.19	0.04	0.06	1.21	0.00	0.00
ARHGEF35	445328	0.54	0.65	0	0.11	3.46	0.00	0.00
ARL6IP1	23204	95.41	97.45	47.15	60.53	0.84	0.00	0.00
ARL6IP6	151188	9.12	10.05	5.94	5.61	0.75	0.00	0.00
ARLNC1	100996425	0.19	0.17	0.01	0.01	4.20	0.00	0.00
ARMCX5	64860	2.68	2.4	1.28	1.81	0.72	0.00	0.00
ARMCX5-GPRASP2	100528062	0.9	0.52	0.34	0.44	0.81	0.00	0.00
ARMT1	79624	7.96	7.83	2.94	6.41	0.75	0.00	0.00
ARNT2	9915	0.02	0	0.02	0	0.76	0.00	0.00
ARNTL	406	2.55	2.68	0.98	1.75	0.90	0.00	0.00
ARNTL2-AS1	101928646	0.96	1.3	0.53	0.23	1.58	0.00	0.00
ARPIN	348110	2.58	2.75	1.58	1.24	0.92	0.00	0.00
ARSD	414	8.12	8.04	4.3	4.05	0.94	0.00	0.00
ARSD-AS1	100506356	0.14	0.35	0.06	0.09	1.70	0.00	0.00
ARSE	415	36.16	33.83	34.72	31.52	0.75	0.00	0.00
ARV1	64801	8.83	9.16	5.09	6.29	0.70	0.00	0.00
ASAH2	56624	3.72	3.97	0.56	1.34	2.39	0.00	0.00
ASB7	140460	2.09	1.84	1.39	1.77	0.83	0.00	0.00
ASH1L	55870	1.36	1.17	0.63	0.8	0.98	0.00	0.00
ASH1L-AS1	645676	0.66	0.41	0.24	0.37	0.80	0.00	0.00
ASNSD1	54529	9.27	8.7	4.62	6.48	0.70	0.00	0.00
ASPHD1	253982	28.18	27.21	15.48	14.9	0.81	0.00	0.00
ASXL2	55252	2.78	2.41	1.72	2.31	0.71	0.00	0.00
ATAD1	84896	4.2	3.82	1.99	3.1	0.65	0.00	0.00
ATE1	11101	4	4.49	1.82	3.51	0.64	0.00	0.00
ATL1	51062	0.86	0.72	0.33	0.34	0.73	0.00	0.00
ATP10D	57205	0.21	0.28	0.11	0.14	1.30	0.00	0.00
ATP13A3	79572	2.9	2.94	1.24	2.41	0.66	0.00	0.00
ATP1A1-AS1	84852	0.55	0.67	0.13	0.18	1.47	0.00	0.00

ATP2B4	493	2.96	2.92	1.07	0.94	1.57	0.00	0.00
ATP6V1FNB	100130705	0.29	0.29	0.13	0.05	1.76	0.00	0.00
ATP9A	10079	6.61	5.98	2.52	2.41	1.35	0.00	0.00
ATPAF1	64756	25.06	24.18	13.79	15.98	0.72	0.00	0.00
ATRX	546	0.77	0.81	0.24	0.7	0.60	0.00	0.00
ATXN7L3	56970	34.04	33.2	38.83	34.85	2.49	0.00	0.00
AUH	549	6.81	7.22	3.51	4.1	0.90	0.00	0.00
B3GNT10	100288842	0.38	0.78	0.25	0.2	1.39	0.00	0.00
B4GALNT2	124872	0	0.1	0	0	0.99	0.00	0.00
B4GALNT3	283358	8.59	8.54	4.7	4.8	0.81	0.00	0.00
B4GALT1	2683	31.94	31.93	22.8	19.51	0.60	0.00	0.00
B4GALT6	9331	0.37	0.57	0.12	0.15	1.32	0.00	0.00
BABAM1	29086	41.5	44.88	69.72	66.58	0.67	0.00	0.00
BAG5	9529	3.12	3.29	1.71	2.25	0.69	0.00	0.00
BAIAP3	8938	1.86	1.66	1.03	0.78	0.97	0.00	0.00
BARD1	580	1.52	1.36	0.57	0.64	1.54	0.00	0.00
BATF	10538	3.7	3.79	1.55	0.48	1.89	0.00	0.00
BAZ1B	9031	8.34	9.05	5.21	6.18	0.62	0.00	0.00
BBS1	582	4.47	4.98	3.98	2.47	0.62	0.00	0.00
BBS10	79738	0.95	1.01	0.18	0.46	1.60	0.00	0.00
BBS4	585	3.84	3.92	2.89	2.13	0.64	0.00	0.00
BCAM	4059	135.59	129.56	75.82	69.69	0.85	0.00	0.00
BCL10	8915	2.84	2.93	1.96	1.48	0.94	0.00	0.00
BCL11B	64919	0.19	0.09	0	0.05	2.90	0.00	0.00
BCL7C	9274	65.36	69.1	41.73	39.15	0.64	0.00	0.00
BHLHE40	8553	14.66	14.53	20.33	16.24	1.32	0.00	0.00
BICDL1	92558	13.15	12.32	6.13	8.81	0.75	0.00	0.00
BIRC3	330	0.26	0.44	0.06	0.07	1.96	0.00	0.00
BIRC6	57448	2.2	2.07	1.15	1.75	0.65	0.00	0.00
BLM	641	1.22	1.22	0.6	0.82	0.83	0.00	0.00
BLOC1S1-RDH5	100528022	1.86	2.14	1.84	1.3	0.61	0.00	0.00
BLOC1S5	63915	5.81	5.85	1.8	3.49	1.13	0.00	0.00
BMF	90427	5.13	5.02	5.09	1.65	0.60	0.00	0.00

BMP8B	656	1.67	1.71	1.06	0.8	3.30	0.00	0.00
BMPR2	659	1.75	1.87	1.05	1.12	0.88	0.00	0.00
BMS1	9790	6.3	6.69	3.46	4.19	0.88	0.00	0.00
BMS1P1	399761	0.23	0.59	0.14	0.26	1.07	0.00	0.00
BMS1P2	642826	0.85	1.11	0.71	0.51	0.68	0.00	0.00
BNIP3L	665	8.19	8.68	5.58	4.41	0.76	0.00	0.00
BORCS7	119032	5.82	6.58	3.9	4.52	0.61	0.00	0.00
BORCS7-ASMT	100528007	3.46	5.18	2.7	3.37	0.64	0.00	0.00
BPGM	669	2.05	1.93	1.36	0.72	0.96	0.00	0.00
BPHL	670	30.73	28.98	15.23	18.93	0.84	0.00	0.00
BPNT1	10380	14.98	14.86	17.15	16.44	0.67	0.00	0.00
BPTF	2186	3.02	3.19	1.48	2.2	0.73	0.00	0.00
BRD8	10902	11.97	10.72	8.03	7.16	0.58	0.00	0.00
BRIP1	83990	0.86	0.87	0.35	0.67	0.78	0.00	0.00
BRMS1	25855	20.92	20.74	16.07	10.6	0.65	0.00	0.00
BSPRY	54836	7.21	7.94	2.07	0.92	1.85	0.00	0.00
BST1	683	1.26	0.78	0.32	0.53	1.27	0.00	0.00
BTBD11	121551	5.58	5.74	2.65	3.53	0.93	0.00	0.00
BTBD9	114781	0.65	0.61	0.17	0.27	1.55	0.00	0.00
BTN2A2	10385	6.56	6.66	3.67	3.04	0.95	0.00	0.00
BTN3A1	11119	2.76	3.41	2.14	1.44	0.70	0.00	0.00
BTN3A3	10384	1.79	1.55	1.22	1.02	0.66	0.00	0.00
BUB1	699	9.8	9.47	5.18	6.79	0.69	0.00	0.00
BUB1B	701	7.79	8.04	4.28	5.77	0.64	0.00	0.00
C1R	715	5	5.21	3.08	1.96	0.88	0.00	0.00
C2CD5	9847	1.88	1.84	0.84	1.18	0.93	0.00	0.00
C3	718	1028.22	1021.88	723.37	431.11	0.83	0.00	0.00
C6	729	0.94	0.82	0.19	0.22	2.15	0.00	0.00
CABIN1	23523	29.98	28.1	19.48	15.76	0.77	0.00	0.00
CACNA1F	778	0.11	0.09	0.03	0.02	2.11	0.00	0.00
CALD1	800	14.77	14.93	7.4	11.75	0.63	0.00	0.00
CAMK2N2	94032	2.72	4.39	0.87	0.67	2.20	0.00	0.00
CAND1	55832	6.56	6.54	3.07	5.02	0.69	0.00	0.00

CANX	821	138.26	135.53	66.41	87.04	0.71	0.00	0.00
CAPG	822	24.7	25.91	16.74	15	0.73	0.00	0.00
CAPN12	147968	0.68	0.88	0.31	0.42	1.00	0.00	0.00
CAPN7	23473	3.05	3.04	1.63	2.11	0.65	0.00	0.00
CAPRIN1	4076	46.93	46.28	25.55	35.19	0.66	0.00	0.00
CARF	79800	0.29	0.34	0.13	0.26	1.15	0.00	0.00
CARMIL1	55604	5.82	5.11	1.63	2.26	1.48	0.00	0.00
CARMIL2	146206	3.58	3.33	3.37	2.2	0.67	0.00	0.00
CARNMT1	138199	1.22	1.23	0.42	1.15	0.75	0.00	0.00
CASP3	836	5.93	7.54	2.58	6.37	0.59	0.00	0.00
CASP6	839	9.36	11.34	5.55	7.44	0.69	0.00	0.00
CASTOR1	652968	1.71	1.86	1.24	0.75	0.86	0.00	0.00
CAT	847	94.18	93.62	38.59	48.66	1.10	0.00	0.00
CATIP-AS2	103689911	0.25	0.03	0	0.03	3.19	0.00	0.00
CATSPER2P1	440278	0.27	0.19	0.13	0.1	0.85	0.00	0.00
CATSPERG	57828	0.6	0.71	0.35	0.23	1.27	0.00	0.00
CAVIN2	8436	2.91	4.41	0	0.16	5.24	0.00	0.00
CBFA2T3	863	2.19	1.95	1	1.22	0.89	0.00	0.00
CBLC	23624	12.62	12	9.61	6.3	0.67	0.00	0.00
CBX1	10951	29.78	28.82	17.32	19.21	0.68	0.00	0.00
CBX5	23468	7.06	7.03	4.5	4.87	0.59	0.00	0.00
CBX6	23466	8.26	8.82	7.04	4.4	0.59	0.00	0.00
CBX7	23492	3.54	3.41	1.6	1.19	1.29	0.00	0.00
CCAR1	55749	5.72	5.82	1.82	5.23	0.63	0.00	0.00
CCDC115	84317	26.63	26.72	16.13	16.92	0.71	0.00	0.00
CCDC122	160857	1.09	1.38	0.41	0.33	1.51	0.00	0.00
CCDC142	84865	3.74	3.91	1.57	1.7	1.22	0.00	0.00
CCDC146	57639	1.34	1.6	1.1	1.17	0.60	0.00	0.00
CCDC170	80129	2.21	2.35	1.02	0.62	1.51	0.00	0.00
CCDC174	51244	2.03	2.4	1.37	1.42	0.65	0.00	0.00
CCDC183	84960	3.58	3.09	2.61	1.88	0.60	0.00	0.00
CCDC191	57577	0.24	0.19	0.14	0.04	2.09	0.00	0.00
CCDC25	55246	4.97	5.11	2.86	3.45	0.68	0.00	0.00

CCDC30	728621	0.46	1	0.28	0.12	1.73	0.00	0.00
CCDC43	124808	11.9	12.41	7.31	8.35	0.64	0.00	0.00
CCDC47	57003	13.83	14.65	5.97	10.62	0.62	0.00	0.00
CCDC93	54520	4.63	4.26	2.71	3.21	0.67	0.00	0.00
CCNG1	900	66.89	69.59	37.76	52.73	0.61	0.00	0.00
CCNI	10983	3.46	3.19	2.37	1.94	0.71	0.00	0.00
CCNJL	79616	1.95	2.19	1.44	1.21	0.63	0.00	0.00
CCNT1	904	2.06	1.68	0.96	1.21	0.80	0.00	0.00
CCT2	10576	57.94	64.92	39.14	39.4	0.64	0.00	0.00
CCZ1	51622	12.16	12.3	11.36	12.2	0.77	0.00	0.00
CD101	9398	0.32	0.38	0.04	0.03	2.53	0.00	0.00
CD164	8763	36.99	40.35	20.61	28.57	0.67	0.00	0.00
CD24	100133941	20.83	24.13	13.26	9.29	1.01	0.00	0.00
CD300A	11314	0.01	0.01	0	0	1.07	0.00	0.00
CD302	9936	3.35	2.87	1.21	2.64	0.69	0.00	0.00
CD46	4179	32.31	31.42	14.79	20.89	0.83	0.00	0.00
CD7	924	66.51	58.24	23.28	20.97	1.50	0.00	0.00
CD74	972	275.63	263.49	164.63	143.82	0.80	0.00	0.00
CD83	9308	0.79	1.02	0.23	0.1	2.49	0.00	0.00
CD8A	925	0.53	1.22	0.24	0.12	2.34	0.00	0.00
CD8B	926	0.85	0.79	0.38	0.16	1.62	0.00	0.00
CDC23	8697	13.08	13.38	8.49	8.93	0.60	0.00	0.00
CDC26	246184	20.82	20.09	10.17	13.87	0.77	0.00	0.00
CDC42EP4	23580	44.57	44.02	25.29	25.27	0.84	0.00	0.00
CDC42EP5	148170	7.99	6.27	4.48	2.68	0.98	0.00	0.00
CDCA2	157313	2.19	2.01	1	1.6	0.75	0.00	0.00
CDH1	999	25.23	24.64	10.42	14.76	0.98	0.00	0.00
CDH15	1013	3.41	3.57	1.69	2.01	0.92	0.00	0.00
CDK19	23097	2.31	2.23	1.08	1.48	0.99	0.00	0.00
CDK5RAP2	55755	10.04	9.7	6.59	6.17	0.97	0.00	0.00
CDK6	1021	2.95	2.96	0.93	2.43	0.77	0.00	0.00
CDK7	1022	8.26	9.49	7.58	9.77	0.60	0.00	0.00
CDKN2AIP	55602	2	2.64	1.13	1.81	0.70	0.00	0.00

CELSR1	9620	2.09	1.83	0.61	0.97	1.33	0.00	0.00
CENPF	1063	1.47	1.6	0.72	1.2	0.65	0.00	0.00
CENPH	64946	9.52	8.88	5.69	5.5	0.75	0.00	0.00
CENPI	2491	0.96	0.68	0.33	0.48	0.77	0.00	0.00
CENPK	64105	3.06	2.42	0.87	1.91	0.99	0.00	0.00
CENPL	91687	1.29	2.09	1.26	1.13	0.61	0.00	0.00
CENPN	55839	14.74	14.95	10.54	11.47	0.62	0.00	0.00
CENPS	378708	6.93	7.22	3.4	5.79	0.62	0.00	0.00
CENPS-CORT	100526739	6.53	6.61	4.32	4.34	0.69	0.00	0.00
CEP152	22995	0.23	0.34	0.02	0.23	1.20	0.00	0.00
CEP192	55125	1.57	1.48	0.5	0.7	1.37	0.00	0.00
CEP350	9857	0.33	0.35	0.11	0.26	0.85	0.00	0.00
CEP57L1	285753	0.41	0.75	0.16	0.61	0.92	0.00	0.00
CEP78	84131	2.68	2.5	1.14	1.95	0.66	0.00	0.00
CERCAM	51148	15.14	13.96	10.1	7.57	0.70	0.00	0.00
CFAP44	55779	0.32	0.25	0.08	0.21	1.47	0.00	0.00
CFAP65	255101	0.18	0.11	0.02	0.01	3.62	0.00	0.00
CFAP74	85452	2.36	2.67	1.91	1.39	0.61	0.00	0.00
CFAP97	57587	3.29	3.51	2.07	1.99	0.66	0.00	0.00
CFB	629	85.99	87.44	68.52	43.87	0.64	0.00	0.00
CGNL1	84952	2.88	2.88	1.03	0.91	1.60	0.00	0.00
CHCHD2	51142	323.51	325.6	357.98	339.85	0.68	0.00	0.00
CHD2	1106	5.88	6.01	3.3	3.91	0.72	0.00	0.00
CHD6	84181	2.77	3.11	2.15	1.72	0.69	0.00	0.00
CHD8	57680	7.43	7.49	5.1	5	0.61	0.00	0.00
CHDH	55349	38.23	38.11	20.44	23.2	0.83	0.00	0.00
CHN1	1123	2.02	2.26	1.33	1.28	0.75	0.00	0.00
CHRNA5	1138	1.6	1.73	1.17	0.95	0.67	0.00	0.00
CHST9	83539	2.5	2.38	0.61	1.67	0.91	0.00	0.00
CIAO1	9391	33	33.41	29.59	25.69	1.18	0.00	0.00
CIPC	85457	2.63	2.8	1.54	1.89	0.65	0.00	0.00
CIR1	9541	2.88	3.07	1.26	2.16	0.70	0.00	0.00
CITED2	10370	10.92	9.67	4.97	7.04	0.78	0.00	0.00

CKAP2	26586	2.93	2.67	0.44	3.08	0.71	0.00	0.00
CLDN12	9069	4.35	5.06	3.08	3.69	0.95	0.00	0.00
CLDN19	149461	8.55	8.25	5.3	5.75	0.62	0.00	0.00
CLDN3	1365	13.93	13.77	5.57	5.54	1.32	0.00	0.00
CLEC2L	154790	1.05	1.24	1.04	0.59	1.07	0.00	0.00
CLHC1	130162	1.03	1.55	0.44	0.72	1.09	0.00	0.00
CLINT1	9685	12.12	12.26	5.98	8.18	0.69	0.00	0.00
CLK1	1195	5.9	6.8	2.95	3.63	0.95	0.00	0.00
CLK4	57396	1.03	1.2	0.42	0.85	0.76	0.00	0.00
CLMN	79789	9.45	9.77	5.31	4.09	1.20	0.00	0.00
CLNS1A	1207	66.91	69.58	48.04	39.83	0.64	0.00	0.00
CLTC	1213	34.92	35.81	16.89	29.9	0.59	0.00	0.00
CLUHP3	100132341	4.59	4.5	2.73	3.89	0.75	0.00	0.00
CMBL	134147	9.48	9.96	7.37	5.44	0.60	0.00	0.00
CMC4	100272147	6.69	8.15	4.36	5.47	0.61	0.00	0.00
CMTM6	54918	11.34	12.47	6.22	8.41	0.70	0.00	0.00
CMTR2	55783	1.16	1.07	0.46	0.82	0.74	0.00	0.00
CNBD2	140894	31.96	29.93	17.45	21.98	0.69	0.00	0.00
CNDP1	84735	0.03	0.03	0	0	3.74	0.00	0.00
CNOT1	23019	71.78	71.4	46.38	52.32	0.71	0.00	0.00
CNOT10	25904	8.06	8.98	5.08	5.83	0.72	0.00	0.00
CNOT4	4850	2.06	2.06	1.24	1.6	0.60	0.00	0.00
CNOT6	57472	3.48	3.4	1.84	2.4	0.61	0.00	0.00
CNP	1267	92.53	95.02	47.08	49.02	0.95	0.00	0.00
CNPY3-GNMT	107080644	1.03	0	0	0	4.85	0.00	0.00
CNTF	1270	0	0	0.01	0	1.03	0.00	0.00
CNTLN	54875	0.39	0.35	0.08	0.29	1.36	0.00	0.00
CNTNAP3	79937	0.17	0.15	0.07	0.04	1.59	0.00	0.00
COA5	493753	6.27	7.58	4.54	5.13	0.67	0.00	0.00
COBL	23242	6.41	6.33	3.53	4.32	0.71	0.00	0.00
COG3	83548	5.78	5.87	4.26	5.94	0.77	0.00	0.00
COL16A1	1307	3.99	3.53	3.4	1.52	0.67	0.00	0.00
COL18A1	80781	43.58	44.63	35.18	22.66	0.61	0.00	0.00

COL9A3	1299	103.19	100.75	57.38	23.15	1.34	0.00	0.00
COMMD2	51122	9.03	8.66	6.95	6.18	0.62	0.00	0.00
COMMD4	54939	40.5	44.13	39.71	41.35	1.23	0.00	0.00
COMP	1311	7.77	7.91	3.71	1.72	1.53	0.00	0.00
COPB2	9276	36.02	35.71	23.03	26.41	3.49	0.00	0.00
COPS9	150678	30.47	28.8	29.09	31.96	1.48	0.00	0.00
COQ7	10229	7.29	8.71	6.16	5.94	1.18	0.00	0.00
CORT	1325	0.13	0.06	0.19	0.06	0.91	0.00	0.00
COX11	1353	32.8	35.03	18.02	23.9	0.76	0.00	0.00
CP	1356	15.65	15.43	4.16	2.19	2.30	0.00	0.00
CPLX2	10814	121.45	119.69	80.86	51.95	0.86	0.00	0.00
CPNE3	8895	7.01	6.99	2.92	5.18	0.79	0.00	0.00
CPSF2	53981	4.52	5.14	2.7	3.49	0.65	0.00	0.00
CPSF3	51692	26.72	25.43	15.73	17.79	0.64	0.00	0.00
CPSF4L	642843	1.69	0.99	0.8	0.13	2.38	0.00	0.00
CPT1A	1374	20.93	19.63	9.92	10.82	0.94	0.00	0.00
CPT1B	1375	0.6	0.68	0.01	0.12	3.32	0.00	0.00
CREB1	1385	2.91	2.62	2.06	1.83	0.65	0.00	0.00
CREBL2	1389	6	6.37	3.36	3.72	0.82	0.00	0.00
CREG1	8804	19.29	19.82	7.78	9.86	1.15	0.00	0.00
CRIM1	51232	15.76	15.27	8.51	9.76	0.76	0.00	0.00
CRIP1	1396	140.45	156.13	71.3	61.36	1.16	0.00	0.00
CRLF1	9244	666.74	675.5	1219.88	1010.7	0.74	0.00	0.00
CRNKL1	51340	5.74	5.95	2.79	4.45	0.62	0.00	0.00
CRYBB2	1415	0.2	0.17	0.06	0.03	2.57	0.00	0.00
CRYBG3	131544	0.2	0.19	0.03	0.14	1.01	0.00	0.00
CRYZL1	9946	4.04	3.63	2.83	2.96	0.63	0.00	0.00
CSDE1	7812	52.77	52.76	27.25	35.61	0.79	0.00	0.00
CSE1L	1434	38.74	37.87	14.84	28.86	0.80	0.00	0.00
CSGALNACT1	55790	0.5	0.61	0.18	0.16	1.41	0.00	0.00
CSTF2T	23283	0.63	0.65	0.21	0.17	1.70	0.00	0.00
CSTF3	1479	13.23	12.9	11.66	12.15	0.64	0.00	0.00
CTDSP2	10106	29.83	30.91	24.82	10.26	0.81	0.00	0.00

CTDSPL2	51496	1.91	1.99	0.99	1.35	0.68	0.00	0.00
CTNNB1	1499	166.04	168.81	96.52	84.28	0.91	0.00	0.00
CTNNBIP1	56998	113.26	114.1	76.8	86.32	0.59	0.00	0.00
CTR9	9646	3.19	3.16	1.52	2.39	0.64	0.00	0.00
CTSC	1075	295.14	300.78	135.61	114.41	1.28	0.00	0.00
CTSS	1520	1.32	1.42	0.68	0.43	1.22	0.00	0.00
CTSV	1515	4.11	3.35	1.21	0.54	1.99	0.00	0.00
CUL1	8454	14.14	13.55	7.44	10.11	0.70	0.00	0.00
CWF19L2	143884	0.69	0.54	0.55	0.71	0.61	0.00	0.00
CXCL2	2920	0.54	0.73	0.2	0	2.93	0.00	0.00
CXCL5	6374	0.35	0.46	0	0.02	5.19	0.00	0.00
CYB561	1534	6.19	6.64	4.33	3.66	0.67	0.00	0.00
CYP26B1	56603	1.35	1.1	0.55	0.22	1.78	0.00	0.00
CYP2J2	1573	1.75	1.69	0.21	0.15	3.30	0.00	0.00
CYP2R1	120227	1.72	1.23	0.51	0.69	0.98	0.00	0.00
CYP2S1	29785	3.44	3.11	7.99	7.53	0.72	0.00	0.00
CYP51A1-AS1	613126	1.05	1.39	0.57	0.76	0.72	0.00	0.00
DAAM1	23002	2.06	1.72	0.91	1.42	0.69	0.00	0.00
DARS	1615	41.16	44.41	22.31	24.93	0.85	0.00	0.00
DARS-AS1	101928243	0.79	0.86	0.44	0.4	0.94	0.00	0.00
DCAF16	54876	9.13	8.51	4.69	5.22	0.77	0.00	0.00
DCAF6	55827	12.94	11.95	5	8.33	0.91	0.00	0.00
DCDC2	51473	6.67	6.78	2.7	4.03	1.08	0.00	0.00
DCUN1D4	23142	1.63	1.95	0.93	1.33	0.64	0.00	0.00
DDAH1	23576	19.15	17.66	6.78	16.62	0.66	0.00	0.00
DDHD2	23259	10.53	9.97	6.48	6.47	0.66	0.00	0.00
DDI2	84301	8.02	8.82	3.38	5.15	1.11	0.00	0.00
DDO	8528	2.28	1.83	0.66	1.02	1.23	0.00	0.00
DDR1	780	26.58	25.3	18.18	15.17	0.65	0.00	0.00
DDX1	1653	24.23	25.13	11.96	16.41	0.80	0.00	0.00
DDX18	8886	10.63	10	5.1	7.78	0.66	0.00	0.00
DDX20	11218	4.13	3.61	2.25	2.48	0.84	0.00	0.00
DDX23	9416	30.1	31.17	24.35	28.96	4.61	0.00	0.00

DDX42	11325	29.99	31.21	19.68	18.2	0.69	0.00	0.00
DDX52	11056	0.63	0.47	0.33	0.29	0.81	0.00	0.00
DDX6	1656	7.89	8.08	3.66	4.75	0.90	0.00	0.00
DENND1B	163486	0.34	0.34	0.09	0.36	1.11	0.00	0.00
DENND6A	201627	3.33	3.11	1.65	2.29	0.66	0.00	0.00
DENR	8562	14.13	14.23	7.67	10	0.68	0.00	0.00
DEPDC1	55635	0.88	1.1	0.43	0.88	0.63	0.00	0.00
DEPDC1B	55789	12.4	11.87	6.11	9.4	0.69	0.00	0.00
DET1	55070	4.17	3.06	2.37	1.93	0.80	0.00	0.00
DGAT1	8694	114.75	110.8	56.64	82.89	0.73	0.00	0.00
DGCR6L	85359	36.13	37.2	26.87	22.74	0.59	0.00	0.00
DGLUCY	80017	7.47	7.92	4.96	5.21	0.76	0.00	0.00
DHFR	1719	19.53	20.93	8.06	10.86	1.10	0.00	0.00
DHFR2	200895	0.77	0.98	0.33	0.32	1.58	0.00	0.00
DHRS2	10202	203.37	209.46	108.57	133.36	0.74	0.00	0.00
DHX15	1665	25.89	25.78	14.51	17.86	0.67	0.00	0.00
DHX33	56919	18.66	19.03	10.37	12.51	0.72	0.00	0.00
DHX40	79665	4.43	4.8	1.87	2.17	1.19	0.00	0.00
DHX57	90957	4.37	4.43	2.06	2.84	0.85	0.00	0.00
DHX9	1660	29.65	27.89	15	19.05	0.76	0.00	0.00
DICER1	23405	2.12	2.29	1.09	1.6	0.68	0.00	0.00
DIS3L	115752	9.78	9.92	5.06	6.45	0.79	0.00	0.00
DKK1	22943	52.18	55.3	14.84	27.41	1.36	0.00	0.00
DLD	1738	4.68	4.77	2.09	3.16	0.75	0.00	0.00
DLG1	1739	5.08	4.53	2.31	3.26	0.78	0.00	0.00
DLG3	1741	2.88	2.99	1.38	0.91	1.35	0.00	0.00
DMD	1756	3.46	3.91	1.73	2.75	0.62	0.00	0.00
DMGDH	29958	2.87	2.3	1.23	1.18	1.14	0.00	0.00
DMKN	93099	26.3	22.53	9.09	4.88	1.84	0.00	0.00
DNAH3	55567	2.72	3.2	1.99	1.13	0.86	0.00	0.00
DNAJA1	3301	49.11	49.62	28.46	30.54	0.80	0.00	0.00
DNAJB13	374407	0.96	1.1	0.35	0.52	1.21	0.00	0.00
DNAJB6	10049	33.1	37.67	24.98	21.49	0.62	0.00	0.00

DNAJC13	23317	2.52	2.23	1.05	1.66	0.62	0.00	0.00
DNAJC22	79962	23.8	24.24	12.98	14.36	0.90	0.00	0.00
DNAJC25	548645	1.8	1.59	1.08	1.16	0.81	0.00	0.00
DNAJC5G	285126	0.08	0.14	0.01	0.04	3.25	0.00	0.00
DNHD1	144132	0.33	0.39	0.23	0.19	0.60	0.00	0.00
DNMT3B	1789	8.59	8.7	6.4	5.32	0.58	0.00	0.00
DOC2A	8448	0.32	0.32	0.03	0.03	3.10	0.00	0.00
DOCK1	1793	6.33	6.22	3.41	3.59	0.84	0.00	0.00
DOCK7	85440	6.75	6.59	2.72	4.57	0.71	0.00	0.00
DOCK9	23348	3.66	3.88	2.24	2.93	1.20	0.00	0.00
DOK2	9046	0.72	0.72	0.08	0.12	2.78	0.00	0.00
DOK4	55715	39.92	40.36	49.92	53.18	2.96	0.00	0.00
DOP1A	23033	0.69	0.61	0.31	0.49	0.64	0.00	0.00
DPEP1	1800	40.89	42.98	28.91	22.39	0.70	0.00	0.00
DPY19L4	286148	1.46	1.71	0.82	1.23	0.76	0.00	0.00
DQX1	165545	18.78	16.15	9.8	12.51	0.62	0.00	0.00
DRC7	84229	1.66	1.3	0.52	0.37	1.66	0.00	0.00
DSC2	1824	5	5.56	1.37	1.61	1.87	0.00	0.00
DSCC1	79075	2.23	3.05	1.16	1.69	0.90	0.00	0.00
DSG1	1828	0.01	0	0	0	0.98	0.00	0.00
DSG2	1829	11.06	11.28	2.56	3.06	2.00	0.00	0.00
DSN1	79980	12.52	14.28	4.96	6.24	1.19	0.00	0.00
DSTYK	25778	1.02	1.23	0.83	0.78	0.60	0.00	0.00
DTL	51514	7.58	7.58	4.28	6.13	0.62	0.00	0.00
DTX4	23220	5.47	5.28	2.33	4.19	0.72	0.00	0.00
DUOXA1	90527	0	0	0.04	0	0.72	0.00	0.00
DYNC1LI1	51143	8.55	8.44	5.74	5.67	0.59	0.00	0.00
DYNC2LI1	51626	3.23	3.28	1.92	2.74	0.63	0.00	0.00
DYSF	8291	1.12	1.27	0.37	0.22	2.05	0.00	0.00
DZIP3	9666	1.23	1.12	0.4	1.04	0.69	0.00	0.00
E2F7	144455	1.96	2.02	0.93	1.08	1.01	0.00	0.00
E2F8	79733	2.12	1.81	1.03	1.64	0.58	0.00	0.00
EBLN2	55096	0.64	0.3	0.07	0.14	2.20	0.00	0.00

ECPAS	23392	13.61	13.3	5.68	7.08	1.12	0.00	0.00
EDRF1	26098	1.06	1.25	0.53	0.81	0.64	0.00	0.00
EED	8726	5.58	5.06	3.63	3.29	0.65	0.00	0.00
EEF1B2	1933	834.08	841.28	570.38	487.72	0.66	0.00	0.00
EFCAB11	90141	2.27	2.52	1.24	1.4	0.75	0.00	0.00
EFCAB8	388795	0.71	0.6	0.67	0.63	0.86	0.00	0.00
EFL1	79631	2.61	2.11	0.95	1.5	1.05	0.00	0.00
EGF	1950	0.9	0.55	0.16	0.19	2.01	0.00	0.00
EGLN3	112399	3.03	1.84	2.61	0.15	0.76	0.00	0.00
EHBP1L1	254102	8.22	8.56	5.95	5.52	0.60	0.00	0.00
EHF	26298	0.11	0.32	0	0	5.56	0.00	0.00
EHHADH	1962	6	6.17	1.95	4.09	1.03	0.00	0.00
EID2B	126272	1.35	1.04	0.09	0.64	1.42	0.00	0.00
EIF2AK2	5610	2.82	2.91	1.48	2.25	0.75	0.00	0.00
EIF3A	8661	14.28	13.89	5.26	9.16	0.91	0.00	0.00
EIF3J	8669	15.63	15.39	8.1	11.63	0.65	0.00	0.00
EIF4B	1975	174.03	179.62	104.13	91.92	0.85	0.00	0.00
ELAC1	55520	1.81	1.97	1.16	0.87	0.93	0.00	0.00
ELF1	1997	12.51	12.46	5.85	6.97	1.01	0.00	0.00
ELF2	1998	2.69	2.4	0.99	1.48	0.90	0.00	0.00
ELF3	1999	14.77	16.88	9.44	4.33	0.91	0.00	0.00
ELF5	2001	7.76	6.82	1.96	3.22	1.50	0.00	0.00
ELK1	2002	1.44	1.79	1.11	0.76	0.73	0.00	0.00
ELK4	2005	1.83	2	1.01	1.73	0.71	0.00	0.00
ELL2	22936	12.08	14.16	8.19	9.33	0.66	0.00	0.00
ELMOD1	55531	0	0	0.06	0	0.63	0.00	0.00
EML5	161436	0.12	0.14	0.03	0.07	1.48	0.00	0.00
EMX1	2016	7.71	7.47	4.65	6.01	0.71	0.00	0.00
ENAH	55740	4.66	4.05	2.4	2.41	0.87	0.00	0.00
ENC1	8507	17.37	19	8.65	10.64	0.92	0.00	0.00
ENOX2	10495	2.56	2.2	1.25	2.09	1.71	0.00	0.00
ENY2	56943	15.04	15.84	9.73	12.81	0.59	0.00	0.00
EP300-AS1	101927279	0.37	0.27	0.15	0.15	1.00	0.00	0.00

EPB41	2035	7.85	8.53	4.98	5.34	0.64	0.00	0.00
EPHA10	284656	0.44	0.5	0.19	0.2	1.42	0.00	0.00
EPHX4	253152	0.95	1.26	0.06	0.06	4.41	0.00	0.00
EPM2AIP1	9852	1.55	1.61	0.69	1	0.99	0.00	0.00
EPS15	2060	3.71	3.17	2.03	2.15	0.70	0.00	0.00
EPS8L1	54869	4.76	4.21	2.92	1.7	0.91	0.00	0.00
ERAP2	64167	1.84	1.83	0.77	1.43	0.77	0.00	0.00
ERBB2	2064	17.17	13.75	7.69	6.23	1.18	0.00	0.00
ERBB3	2065	82.73	82.68	49.64	42.48	0.95	0.00	0.00
ERCC5	2073	2.01	2.41	0.89	1.94	0.64	0.00	0.00
ERCC6L	54821	0.52	0.6	0.21	0.38	0.93	0.00	0.00
ERI2	112479	1.51	1.46	1.15	1.45	0.59	0.00	0.00
ERVK13-1	100507321	3.82	3.9	2.44	2.3	0.94	0.00	0.00
ESCO2	157570	1.31	1.13	0.42	0.76	1.06	0.00	0.00
ESPL1	9700	5.59	4.96	3.51	3.02	0.70	0.00	0.00
ESPN	83715	129.37	124.86	76.79	58.8	0.93	0.00	0.00
ESRP1	54845	4.23	4.25	0.37	0.55	3.16	0.00	0.00
ETAA1	54465	1.56	1.14	0.55	1.3	0.64	0.00	0.00
ETF1	2107	39.6	39.29	22.41	29.18	0.63	0.00	0.00
ETFB	2109	491.63	486.21	349.34	260.76	0.68	0.00	0.00
EVC	2121	0.48	0.54	0.15	0.16	2.19	0.00	0.00
EVPL	2125	7.01	6.54	3.65	5.03	0.63	0.00	0.00
EVPLL	645027	1.03	0.91	0.23	0.2	2.09	0.00	0.00
EXO1	9156	3.49	3.84	1.55	2.48	0.88	0.00	0.00
EXOC3	11336	17.68	17.76	12.4	11.86	0.64	0.00	0.00
EXOC5	10640	1.09	1.15	0.43	0.75	0.67	0.00	0.00
EXOC6	54536	1.52	1.41	0.76	1.14	0.62	0.00	0.00
EXOC6B	23233	4.2	4.17	2.76	3.05	0.58	0.00	0.00
EXOSC9	5393	11.88	12.44	6.29	8.4	0.87	0.00	0.00
EZH2	2146	10.11	8.77	4.81	5.86	0.78	0.00	0.00
EZR	7430	69.95	69.71	43.11	48.72	0.61	0.00	0.00
EZR-AS1	101409257	2.29	2.19	0.73	0.78	1.56	0.00	0.00
F11	2160	6.39	5.65	2.71	5.65	0.68	0.00	0.00

FAAH2	158584	1.49	1.71	0.91	0.44	1.11	0.00	0.00
FADS2	9415	12.84	13.15	23.52	24.09	0.68	0.00	0.00
FAHD1	81889	27.88	29.18	16.24	22.14	0.58	0.00	0.00
FAIM	55179	6.94	5.07	2.58	4.81	0.61	0.00	0.00
FAM102A	399665	26.95	27.89	13.33	11.54	0.99	0.00	0.00
FAM107B	83641	16.01	16.57	8.75	12.67	0.60	0.00	0.00
FAM110C	642273	1.44	1.84	1.12	0.85	0.70	0.00	0.00
FAM111A	63901	1.6	1.54	0.42	1.33	0.85	0.00	0.00
FAM111A-DT	101927204	0.49	0.49	0.21	0.23	1.16	0.00	0.00
FAM133B	257415	7.3	7.9	3.71	4.07	0.92	0.00	0.00
FAM136A	84908	72.73	77.87	41.88	52.07	0.67	0.00	0.00
FAM13B	51306	1.25	1.05	0.55	0.84	0.71	0.00	0.00
FAM151B	167555	0.22	0.19	0.16	0.16	0.83	0.00	0.00
FAM160A1	729830	0.88	0.71	0.43	0.44	0.77	0.00	0.00
FAM160A1-DT	105377486	0.12	0.09	0.16	0.12	0.59	0.00	0.00
FAM168B	130074	29.02	29.92	16.69	18.15	0.76	0.00	0.00
FAM169A	26049	5.59	5.46	2.2	4.6	0.72	0.00	0.00
FAM182A	284800	4.86	4.8	2.66	1.99	1.19	0.00	0.00
FAM184A	79632	1.37	1.05	0.25	0.22	2.29	0.00	0.00
FAM19A5	25817	9.61	9.19	4.38	4.22	1.19	0.00	0.00
FAM200A	221786	1.57	1.73	0.48	1.06	1.09	0.00	0.00
FAM208A	23272	1.87	1.86	0.93	1.5	0.77	0.00	0.00
FAM210A	125228	2.62	3.38	1.47	2.26	0.84	0.00	0.00
FAM215B	644297	0.45	0.63	0.28	0.09	0.79	0.00	0.00
FAM217B	63939	2.45	2.65	1.17	1.44	0.91	0.00	0.00
FAM3B	54097	42.54	39.3	17.3	23.31	1.00	0.00	0.00
FAM49B	51571	16.03	16.62	9.53	10.29	0.74	0.00	0.00
FAM69A	388650	2.98	2.83	1.25	2.28	0.59	0.00	0.00
FAM74A6	653123	0.14	0	0	0.02	1.63	0.00	0.00
FAM81A	145773	0	0.24	0	0	5.70	0.00	0.00
FAM83A	84985	0.82	0.98	0.13	0.06	3.48	0.00	0.00
FAM83H-AS1	100128338	0.97	0.76	0.37	0.66	0.72	0.00	0.00
FAM84B	157638	0.21	0.3	0	0	6.68	0.00	0.00

FAM8A1	51439	17.65	17.16	5.39	11.19	1.07	0.00	0.00
FAN1	22909	2.78	2.46	1.36	1.38	0.72	0.00	0.00
FANCA	2175	17.41	15.28	13.39	9.94	0.66	0.00	0.00
FANCD2	2177	5.63	5.75	3.36	3.4	0.65	0.00	0.00
FANCI	55215	6.48	6.67	3.74	3.79	0.78	0.00	0.00
FARP2	9855	8.07	7.72	5.81	5.41	0.65	0.00	0.00
FASTKD1	79675	2.01	2.36	1.28	1.26	0.65	0.00	0.00
FASTKD2	22868	3.83	2.71	1.44	2.37	0.79	0.00	0.00
FASTKD3	79072	2.13	2.45	1.18	1.66	0.74	0.00	0.00
FBN3	84467	0.69	0.64	0.56	0.13	1.92	0.00	0.00
FBXL21	26223	0.66	0.56	0.12	0.26	1.64	0.00	0.00
FBXL3	26224	1.96	2.42	1.1	1.82	0.59	0.00	0.00
FBXL5	26234	7.18	7.42	5.39	6.19	0.68	0.00	0.00
FBXO21	23014	8.92	8.93	5.29	5.08	0.79	0.00	0.00
FBXO4	26272	6.94	7.63	4.92	3.71	0.68	0.00	0.00
FBXO45	200933	1.89	2.07	1.16	1.41	0.63	0.00	0.00
FCGRT	2217	173.17	177.17	109.09	110.63	0.67	0.00	0.00
FDXACB1	91893	1	0.78	0.8	0.44	0.83	0.00	0.00
FEM1B	10116	2.04	1.95	0.96	1.51	0.69	0.00	0.00
FERMT1	55612	16.38	17.77	10.23	11.5	0.65	0.00	0.00
FERMT2	10979	2.91	2.82	0.28	0.33	3.40	0.00	0.00
FEZ2	9637	28.24	27.62	16.51	17.6	0.73	0.00	0.00
FGD3	89846	0.98	0.77	0.09	0.5	1.62	0.00	0.00
FGL1	2267	24.1	23.5	14.17	16.2	0.63	0.00	0.00
FIGNL1	63979	0.63	0.67	0.33	0.44	0.90	0.00	0.00
FIGNL2	401720	1.14	0.52	0.34	0.29	1.11	0.00	0.00
FKBP1B	2281	21.24	20.35	25.98	21.33	1.03	0.00	0.00
FKBP3	2287	23.22	24.74	11.9	16.39	0.68	0.00	0.00
FKBP5	2289	15.65	17.03	10.15	9.86	0.71	0.00	0.00
FKTN	2218	0.8	0.73	0.41	0.63	0.65	0.00	0.00
FLRT2	23768	0	0	0	0.02	0.69	0.00	0.00
FLVCR1	28982	2.94	2.91	1.41	2.82	0.77	0.00	0.00
FMN1	342184	0.14	0.2	0.04	0.03	2.45	0.00	0.00

FMN2	56776	0	0.01	0.01	0	1.55	0.00	0.00
FMNL3	91010	0.3	0.31	0.19	0.13	0.89	0.00	0.00
FMR1	2332	2.47	1.79	1.01	1.76	0.63	0.00	0.00
FMR1-AS1	100126270	0.59	1.38	0.42	0.48	1.14	0.00	0.00
FNDC11	79025	0	0	0.05	0	1.41	0.00	0.00
FNTA	2339	22.54	25.26	14	13.91	0.77	0.00	0.00
FOPNL	123811	5.43	3.3	2.1	2.17	1.21	0.00	0.00
FOXO3	2309	2.69	2.57	1.44	1.45	0.87	0.00	0.00
FPGT-TNNI3K	100526835	1.02	0.99	0.23	0.72	1.40	0.00	0.00
FRAS1	80144	9.65	9.89	5.81	5.4	0.80	0.00	0.00
FREM1	158326	1.35	1.53	0.15	0.19	3.49	0.00	0.00
FRS2	10818	16.45	14.91	3.56	4.18	1.65	0.00	0.00
FRYL	285527	0.42	0.49	0.17	0.3	1.01	0.00	0.00
FRZB	2487	0.41	0.26	0.08	0	3.06	0.00	0.00
FSTL3	10272	81.56	77.26	19.16	4.58	2.75	0.00	0.00
FTCDNL1	348751	2.02	2.43	1.01	1.28	0.91	0.00	0.00
FUBP1	8880	28.35	28.58	13.41	17.21	0.90	0.00	0.00
FUT2	2524	2.38	2.6	1.19	1.11	1.13	0.00	0.00
FXN	2395	3.81	3.58	2.24	2.36	0.68	0.00	0.00
FXR1	8087	10.57	10.46	5.34	7.07	0.74	0.00	0.00
FXYD2	486	76.31	74.56	47.61	50.22	0.63	0.00	0.00
FXYD3	5349	4.28	5.36	4.97	0.28	1.13	0.00	0.00
FXYD4	53828	0.37	0.37	0.08	0.25	1.47	0.00	0.00
FYB2	199920	0.34	0.42	0.09	0.16	1.46	0.00	0.00
FZD4	8322	13.39	13.65	5.2	5.2	1.38	0.00	0.00
FZD6	8323	2.45	2.67	0.66	0.82	1.77	0.00	0.00
G3BP1	10146	44.24	45.71	23.58	26.69	0.77	0.00	0.00
GALNS	2588	17.86	18.95	25.42	24.32	1.79	0.00	0.00
GALNT11	63917	2.4	2.23	1.1	1.17	0.95	0.00	0.00
GALNT4	8693	0.85	1.09	0.36	0.35	1.44	0.00	0.00
GALNT6	11226	2.58	2.51	0.84	1.15	1.40	0.00	0.00
GARNL3	84253	1.56	1.56	0.8	0.67	0.90	0.00	0.00
GAS2	2620	4.72	4.5	1.5	3.45	0.80	0.00	0.00

GAS6	2621	1.79	1.87	1.35	0.79	0.73	0.00	0.00
GAS6-AS1	650669	0.06	0.1	0.05	0.03	1.16	0.00	0.00
GATA5	140628	0.3	0.29	0.02	0.09	2.46	0.00	0.00
GATA6	2627	3.18	3.54	1.02	0.69	1.97	0.00	0.00
GATAD1	57798	5.39	5.13	3.12	2.99	0.78	0.00	0.00
GATC	283459	15.34	14.14	8	9.79	0.74	0.00	0.00
GATM	2628	1.03	0.85	0.3	0.31	1.39	0.00	0.00
GBP1	2633	0.3	0.25	0.04	0.02	3.36	0.00	0.00
GCA	25801	3.83	5.04	0.67	0.41	2.98	0.00	0.00
GCC2	9648	0.61	1.1	0.43	0.53	0.65	0.00	0.00
GCM1	8521	0.12	0.22	0	0	5.06	0.00	0.00
GCSH	2653	37.79	37.82	19.8	22.15	0.86	0.00	0.00
GDAP1	54332	1.24	1.08	0.44	0.56	1.52	0.00	0.00
GDF9	2661	1.9	2.09	1.22	1.43	0.60	0.00	0.00
GDNF	2668	4.74	5.55	2.08	1.27	1.61	0.00	0.00
GDPD5	81544	30.65	27.69	6.37	4.74	2.45	0.00	0.00
GEN1	348654	0.75	0.67	0.18	0.7	0.91	0.00	0.00
GFM2	84340	7.59	6.54	4.94	4.87	0.59	0.00	0.00
GFOD1	54438	1.6	1.69	0.87	1.36	0.63	0.00	0.00
GGCT	79017	20.6	19.42	8.76	14.48	0.77	0.00	0.00
GGNBP2	79893	5.04	5.51	2.58	3.42	0.76	0.00	0.00
GGT6	124975	8.02	8.87	3.67	4.62	0.98	0.00	0.00
GID4	79018	2.79	2.41	2.29	1.78	0.68	0.00	0.00
GIGYF2	26058	7.39	6.39	4	4.62	0.61	0.00	0.00
GINS1	9837	15.74	14.75	8.85	9.73	0.71	0.00	0.00
GINS2	51659	49.27	46.05	29.69	33.47	0.59	0.00	0.00
GINS4	84296	10.09	9.81	5.58	6.76	0.67	0.00	0.00
GIPC2	54810	11.46	11.32	4.71	7.05	1.01	0.00	0.00
GJC3	349149	1.62	1.56	0.43	0.42	1.91	0.00	0.00
GLA	2717	19.9	21.73	21.39	18.79	1.94	0.00	0.00
GLCE	26035	1.81	1.86	0.9	1.26	0.75	0.00	0.00
GLE1	2733	18.15	15.95	7.7	9.64	0.97	0.00	0.00
GLO1	2739	61.67	61.17	38.24	35.42	0.74	0.00	0.00

GLOD5	392465	5.26	5.44	1.44	2.3	1.50	0.00	0.00
GLT1D1	144423	0.04	0.05	0.03	0	0.59	0.00	0.00
GLYATL2	219970	0	0	0	0	0.72	0.00	0.00
GNAI1	2770	2.78	2.46	1.16	1.24	1.12	0.00	0.00
GNG10	2790	13.9	14.43	6.01	2.29	1.77	0.00	0.00
GNL2	29889	7.16	7.15	3.64	4.99	0.65	0.00	0.00
GNMT	27232	8.76	8.1	0.87	4.1	1.76	0.00	0.00
GNPNAT1	64841	21.79	21.56	10.56	14.36	0.81	0.00	0.00
GOLGA1	2800	4.5	4.01	2.26	3.17	0.63	0.00	0.00
GOLGA2P5	55592	0.93	0.98	0.46	0.48	1.16	0.00	0.00
GOLGA5	9950	8.22	7.79	3.82	6.01	0.69	0.00	0.00
GOLGA6L22	440243	0.01	0	0	0	0.88	0.00	0.00
GOLGA8A	23015	0.81	0.99	0.41	0.45	1.14	0.00	0.00
GOLGA8B	440270	1.14	1.45	1	0.73	0.60	0.00	0.00
GOPC	57120	4.47	4.01	2.13	3.16	0.68	0.00	0.00
GORAB	92344	0.54	0.53	0.18	0.29	1.24	0.00	0.00
GPATCH1	55094	3.6	3.42	1.74	2.51	0.73	0.00	0.00
GPATCH2L	55668	1.29	1.16	1.07	0.99	0.61	0.00	0.00
GPATCH4	54865	9.43	8.74	4.12	5.64	0.85	0.00	0.00
GPNMB	10457	2.44	2.81	0.39	0.65	2.34	0.00	0.00
GPR132	29933	0.05	0.06	0.04	0.03	0.64	0.00	0.00
GPR143	4935	2.79	2.72	1.24	1.05	1.34	0.00	0.00
GPR153	387509	2.74	2.46	1.72	1.34	0.79	0.00	0.00
GPSM2	29899	2.42	2.54	1.31	1.58	0.84	0.00	0.00
GPX2	2877	1205.56	1218.56	815.28	645.16	0.73	0.00	0.00
GPX7	2882	13.31	11.38	6.41	5.69	1.09	0.00	0.00
GRAMD1B	57476	0.18	0.36	0.08	0.15	1.19	0.00	0.00
GRB14	2888	1.15	1.19	0.34	0.6	1.33	0.00	0.00
GREB1	9687	12.9	12.62	8.34	6.99	0.75	0.00	0.00
GRK3	157	3.44	3.21	1.38	1.21	1.23	0.00	0.00
GRPEL2	134266	1.84	1.64	0.94	0.96	0.88	0.00	0.00
GSAP	54103	6.76	7.59	3.38	4.74	0.84	0.00	0.00
GSDMD	79792	17.44	16.86	9.97	12.14	0.59	0.00	0.00

GSTA1	2938	17.43	19.69	7.88	18	0.62	0.00	0.00
GSTA2	2939	6.18	7.56	1.42	4.98	1.11	0.00	0.00
GSTA4	2941	24.89	24.3	17.08	12.54	0.73	0.00	0.00
GSTCD	79807	2.65	2.36	1.35	1.24	1.01	0.00	0.00
GSTM2	2946	6.05	4.99	2.22	1.17	1.70	0.00	0.00
GSTT1	2952	154.24	151.96	88.42	103.01	0.71	0.00	0.00
GTDC1	79712	1.96	1.86	0.94	1.12	0.86	0.00	0.00
GTF2H1	2965	8.52	8.07	4.47	6.43	0.68	0.00	0.00
GTF2H3	2967	6.91	8.19	5.06	4.93	0.64	0.00	0.00
GTF2I	2969	50.39	47.47	28.77	33.63	0.64	0.00	0.00
GTPBP10	85865	3.39	3.27	2.59	3.05	0.79	0.00	0.00
GUSBP1	728411	1.02	0.95	0.59	0.72	0.84	0.00	0.00
GXYLT2	727936	7.9	7.66	3.82	2.78	1.61	0.00	0.00
H2AFV	94239	31.63	31.81	24.59	23.73	0.60	0.00	0.00
H2AFY2	55506	35.18	34.03	26.67	17.58	0.64	0.00	0.00
H6PD	9563	39	39.21	30.24	21.32	0.62	0.00	0.00
HAO1	54363	0.45	0.64	0	0.26	2.06	0.00	0.00
HAUS1	115106	11.07	12.81	5.45	9.27	0.69	0.00	0.00
HAUS4	54930	19.9	20.5	12.33	9	0.92	0.00	0.00
HBP1	26959	4.36	4.55	2.45	2.15	0.69	0.00	0.00
HBS1L	10767	12.69	14	6.63	9.72	0.70	0.00	0.00
HCP5	10866	0.23	0.38	0.05	0	3.59	0.00	0.00
HDHD2	84064	9.44	10.67	5.39	5.55	0.82	0.00	0.00
HDLBP	3069	279.71	275.12	186.19	186.13	0.59	0.00	0.00
HEATR4	399671	0.95	1.05	0.51	0.71	0.73	0.00	0.00
HEATR5B	54497	3.33	3.62	1.48	2.83	0.71	0.00	0.00
HECTD1	25831	3.92	4.05	2.02	3.19	0.68	0.00	0.00
HEG1	57493	2.64	2.5	1.14	0.9	1.33	0.00	0.00
HELB	92797	0.09	0.19	0.09	0.03	1.29	0.00	0.00
HELLS	3070	2.06	2.05	0.97	1.8	0.62	0.00	0.00
HELZ	9931	2.08	2.34	0.89	1.72	0.70	0.00	0.00
HEPACAM	220296	20.47	20.55	10.79	11.91	0.83	0.00	0.00
HERC1	8925	1.19	1.14	0.7	0.84	0.58	0.00	0.00

HERC2P2	400322	0.68	0.51	0.25	0.37	0.95	0.00	0.00
HERC3	8916	1.48	1.34	0.82	0.93	0.72	0.00	0.00
HEY1	23462	5.68	5.33	0.74	0.92	2.73	0.00	0.00
HGFAC	3083	7.56	6.78	5.08	2.77	0.80	0.00	0.00
HHAT	55733	1.96	1.76	0.97	0.93	0.97	0.00	0.00
HHEX	3087	27.85	27.5	16.76	26.22	2.34	0.00	0.00
HINT3	135114	2.78	3.25	1.54	2.37	0.64	0.00	0.00
HIP1R	9026	11.77	12.06	7.23	7.72	0.66	0.00	0.00
HIPK3	10114	2.08	1.93	0.89	1.47	0.77	0.00	0.00
HIST1H2AI	8329	0.33	0.36	0.12	0.12	1.52	0.00	0.00
HIST1H2BK	85236	28.58	24.61	6.63	5.77	2.31	0.00	0.00
HIST2H2AA4	723790	17.56	16.65	0	20.06	0.77	0.00	0.00
HJURP	55355	14.98	14.52	10.08	9.92	0.59	0.00	0.00
HLF	3131	35.42	35.3	24.38	25.1	0.58	0.00	0.00
HLX-AS1	100873924	0.26	0.21	0.02	0.02	3.25	0.00	0.00
HMGB2	3148	59.96	62.24	33.78	30.79	0.90	0.00	0.00
HMGN2P46	283651	0	0.13	0.01	0	4.20	0.00	0.00
HMGN4	10473	26.33	27.08	24.26	21.81	1.09	0.00	0.00
HNF1A-AS1	283460	0.4	0.64	0.11	0.17	1.87	0.00	0.00
HNF1B	6928	5.43	4.24	1.41	1.52	1.72	0.00	0.00
HNMT	3176	29.94	30.1	14.54	19.12	1.08	0.00	0.00
HNRNPA1	3178	674.45	705.71	440.07	442.54	0.62	0.00	0.00
HNRNPH1	3187	91.64	90.38	49.67	64.44	0.68	0.00	0.00
HNRNPH2	3188	8.75	7.49	4.28	5.48	0.73	0.00	0.00
HNRNPR	10236	49.1	47.12	24.53	36.21	0.65	0.00	0.00
HNRNPU	3192	55.13	55.37	29.18	35.97	0.75	0.00	0.00
HNRNPUL1	11100	42.88	39.38	22.36	29.48	0.63	0.00	0.00
HOOK1	51361	1.57	1.43	0.69	1.19	0.68	0.00	0.00
HOOK2	29911	23.78	20.9	14.21	12.12	0.88	0.00	0.00
HOXA5	3202	3.66	3.82	2.07	2.44	0.84	0.00	0.00
HOXD1	3231	28.96	26.51	5.99	5.55	2.27	0.00	0.00
HOXD10	3236	0.16	0.36	0	0	5.09	0.00	0.00
HOXD9	3235	0.18	0.09	0	0	6.87	0.00	0.00

HS3ST4	9951	3.12	4.42	1.99	2.66	0.68	0.00	0.00
HSD17B2	3294	30.58	29.28	3.84	9.3	2.18	0.00	0.00
HSDL2	84263	13.05	13.81	5.72	10.77	0.71	0.00	0.00
HSP90AA1	3320	74.66	78.11	40.06	61.42	0.59	0.00	0.00
HSPA1B	3304	27.01	23.76	16.59	16.6	0.61	0.00	0.00
HSPA8	3312	513.2	520.87	256.18	359.05	0.76	0.00	0.00
HSPB11	51668	28.24	29.43	17.86	18.66	0.67	0.00	0.00
HSPB6	126393	0	0	0.02	0.12	1.81	0.00	0.00
HSPD1	3329	335.32	344.33	169.87	236.93	0.74	0.00	0.00
HTR1D	3352	0.38	0.46	0.02	0.02	4.44	0.00	0.00
HYAL1	3373	135.48	132.91	83.24	88.68	0.65	0.00	0.00
IARS2	55699	36.22	33.9	20.38	26.3	0.59	0.00	0.00
ICAM2	3384	4.16	3.89	3.17	1.4	0.78	0.00	0.00
ICK	22858	3.26	2.98	1.61	2.05	0.97	0.00	0.00
ICOSLG	23308	0.5	0.29	0	0.12	2.07	0.00	0.00
ID4	3400	1.57	1.73	0.8	0.85	1.03	0.00	0.00
IDNK	414328	4.59	4.77	2.36	2.26	1.10	0.00	0.00
IFIH1	64135	1.16	1.04	0.3	0.5	1.47	0.00	0.00
IFNLR1	163702	5.73	6.62	1.5	1.36	2.15	0.00	0.00
IFT46	56912	8.35	7.17	4.69	3.34	0.90	0.00	0.00
IGFALS	3483	3.69	3.07	1.14	2.2	1.01	0.00	0.00
IGFL2	147920	0.86	0.88	0.19	0.09	2.45	0.00	0.00
IGSF23	147710	5.17	4.51	2.3	4.12	0.62	0.00	0.00
IHH	3549	2.38	2.21	0.91	1.97	0.69	0.00	0.00
IK	3550	20.95	20.14	11.02	14.44	0.64	0.00	0.00
IKBKE	9641	4.58	4.47	2.42	2.46	0.86	0.00	0.00
IL12RB2	3595	32.16	34.95	20.73	20.64	0.70	0.00	0.00
IL18RAP	8807	0	0	0	0.01	0.67	0.00	0.00
IL1R1	3554	8.76	8.28	4.54	4.39	0.93	0.00	0.00
IL1R2	7850	5.95	5.23	3.83	2.97	0.68	0.00	0.00
IL22RA1	58985	22.9	23.1	9.15	13.31	1.03	0.00	0.00
IMPA2	3613	73.31	81.11	45.15	45.83	0.78	0.00	0.00
IMPACT	55364	1.17	0.87	0.5	0.48	1.23	0.00	0.00

IMPAD1	54928	8.8	8.67	7	7.97	0.58	0.00	0.00
IMPDH2	3615	299.55	296.82	201.02	172.95	0.67	0.00	0.00
INIP	58493	5.3	5.45	2.54	3.96	0.69	0.00	0.00
INO80B	83444	13.2	12.5	9.19	6.39	0.72	0.00	0.00
INSIG2	51141	10.47	11.18	6.06	5.55	0.96	0.00	0.00
IPO11	51194	3.26	3.54	1.84	2.31	0.64	0.00	0.00
IPP	3652	2.57	2.48	1.82	1.73	0.64	0.00	0.00
IQCK	124152	4.26	3.79	2.29	2.75	0.61	0.00	0.00
ISOC1	51015	26.54	27.09	14.47	19.57	0.66	0.00	0.00
ISX	91464	4.71	4.79	2.42	2.81	0.88	0.00	0.00
ISY1	57461	1.45	1.96	0.9	0.85	0.95	0.00	0.00
ITGA6	3655	11.81	11.12	6.18	6.61	0.84	0.00	0.00
ITGA6-AS1	101929947	0.33	1.25	0.07	0.15	3.43	0.00	0.00
ITGAL	3683	2.14	2.11	0.84	0.95	1.26	0.00	0.00
ITIH5	80760	4.09	4.53	2.56	1.37	1.26	0.00	0.00
ITPR2	3709	2.92	2.71	1.14	2.04	0.96	0.00	0.00
ITPRID2	6744	5.87	5.68	3.05	4.86	0.63	0.00	0.00
ITPRIPL2	162073	3.02	3.21	2.42	1.56	0.68	0.00	0.00
ITSN1	6453	4.7	4.33	2.26	3.18	0.74	0.00	0.00
JADE3	9767	5.13	4.79	2.85	3.6	0.61	0.00	0.00
JAK1	3716	12.26	12.35	7.83	8.41	0.60	0.00	0.00
JMJD7-PLA2G4B	8681	1.71	1.77	0.96	1.27	0.59	0.00	0.00
JRK	8629	2.74	3	1.45	1.07	1.22	0.00	0.00
KANK1	23189	28.28	29.7	16.36	11.57	1.05	0.00	0.00
KANK4	163782	1.48	1.18	0.28	0.24	2.47	0.00	0.00
KAT14	57325	8.88	10.26	5.39	6.19	0.72	0.00	0.00
KATNBL1	79768	2.08	2.72	1.63	1.67	0.67	0.00	0.00
KBTBD6	89890	1.63	1.72	0.86	0.87	0.96	0.00	0.00
KBTBD7	84078	1.37	1.2	0.55	0.87	0.85	0.00	0.00
KCTD1	284252	2.42	1.98	1.41	1.2	0.79	0.00	0.00
KCTD18	130535	3.23	3.02	2.02	2.1	0.71	0.00	0.00
KCTD20	222658	20.75	19.7	14.35	12.63	0.60	0.00	0.00
KCTD3	51133	8.97	9.41	3.35	5.53	1.05	0.00	0.00

KDELC2	143888	5.94	5.33	3.04	4.08	0.67	0.00	0.00
KDF1	126695	13.63	12.33	8.25	8.83	0.60	0.00	0.00
KDM3A	55818	5.42	5.15	3.3	3.4	0.66	0.00	0.00
KDM5A	5927	2.31	2.27	1.51	1.48	0.58	0.00	0.00
KIAA1147	57189	1.73	1.51	0.78	1.72	0.99	0.00	0.00
KIAA1211L	343990	2.71	2.21	0.5	1.27	1.50	0.00	0.00
KIAA1257	57501	4.64	4.18	2.71	2.92	0.72	0.00	0.00
KIAA1586	57691	1.88	1.35	0.46	1.51	0.81	0.00	0.00
KIF11	3832	2.36	2.31	0.97	1.98	0.66	0.00	0.00
KIF12	113220	2.12	1.77	0.83	0.69	1.42	0.00	0.00
KIF13A	63971	6.46	6.23	3.14	4.51	0.66	0.00	0.00
KIF15	56992	1.03	0.81	0.25	0.73	0.99	0.00	0.00
KIF1A	547	1.95	1.83	0.6	0.47	1.81	0.00	0.00
KIF1BP	26128	6.05	5.89	3.5	4.44	0.59	0.00	0.00
KIF4A	24137	8.65	7.85	4	5.05	0.86	0.00	0.00
KIFC1	3833	34.34	34.02	20.22	23.08	0.61	0.00	0.00
KITLG	4254	1.15	0.92	0.46	0.73	0.77	0.00	0.00
KIZ	55857	17.74	16.55	9.43	8.2	0.94	0.00	0.00
KLC3	147700	1.87	1.36	0.75	0.35	1.54	0.00	0.00
KLHL14	57565	8.87	8.99	2.12	3.66	1.63	0.00	0.00
KLHL31	401265	0.17	0.19	0.06	0.04	1.88	0.00	0.00
KLHL36	79786	6.75	6.62	4.41	3.31	0.78	0.00	0.00
KLHL9	55958	1.82	1.88	0.86	1.18	0.91	0.00	0.00
KLK6	5653	2.66	1.74	1.44	0.46	1.15	0.00	0.00
KNG1	3827	3.1	3.03	1.2	2.66	0.71	0.00	0.00
KNTC1	9735	1.79	1.65	0.88	0.94	0.99	0.00	0.00
KPNB1	3837	53.63	53.89	24.78	32.45	0.91	0.00	0.00
KRAS	3845	1.09	1.61	0.61	0.53	1.21	0.00	0.00
KRBA2	124751	2.66	1.84	1.92	1.1	1.09	0.00	0.00
KRCC1	51315	1.85	1.62	0.62	0.97	1.07	0.00	0.00
KREMEN1	83999	23.55	22.95	10.54	7.86	1.36	0.00	0.00
KRIT1	889	1.66	1.44	0.53	0.93	1.05	0.00	0.00
KRT17	3872	1.62	1.68	0.11	0.15	3.69	0.00	0.00

KRTAP5-AS1	338651	0.63	0.69	0.35	0.17	1.23	0.00	0.00
L2HGDH	79944	1.09	1.28	0.56	0.79	0.76	0.00	0.00
LACTB2	51110	7.48	7.52	3.41	5.31	0.80	0.00	0.00
LAMA3	3909	3.04	3.02	1.54	1.91	0.80	0.00	0.00
LAMB2	3913	44.57	43.08	28.87	27.17	0.65	0.00	0.00
LAMB4	22798	0	0	0.04	0.03	2.98	0.00	0.00
LAMC1	3915	31.39	31.23	21.38	15.84	0.76	0.00	0.00
LAMC1-AS1	110841583	3.73	1.97	0	0.58	3.30	0.00	0.00
LAMP2	3920	33.09	33.81	20.11	20.72	0.84	0.00	0.00
LAP3	51056	43.8	45.28	28.87	30.59	0.58	0.00	0.00
LARGE2	120071	30.58	29.77	18.35	7.89	1.22	0.00	0.00
LARS	51520	12.67	11.34	8.31	6.17	0.80	0.00	0.00
LATS1	9113	2.24	1.89	1.4	1.83	0.63	0.00	0.00
LBR	3930	14.72	12.63	6.97	8.47	0.83	0.00	0.00
LCA5	167691	0.17	0.18	0.08	0.09	1.62	0.00	0.00
LCN12	286256	1.36	1.74	0.55	1.21	0.86	0.00	0.00
LCN2	3934	333.13	332.37	49.03	4.98	3.62	0.00	0.00
LCP1	3936	1.5	1.87	0.81	1.15	0.78	0.00	0.00
LDB3	11155	0.48	0.46	0.2	0.33	0.93	0.00	0.00
LDLRAD1	388633	16	15.71	5.53	5.45	1.57	0.00	0.00
LDLRAD4	753	0.02	0	0	0.01	1.64	0.00	0.00
LEPROT	54741	5.29	5.46	1.88	2	1.47	0.00	0.00
LGR5	8549	10.53	11.63	5.89	6.41	0.94	0.00	0.00
LGSN	51557	3.63	3.18	1.9	1.91	0.84	0.00	0.00
LIAS	11019	4.41	5.49	3.58	2.78	0.70	0.00	0.00
LIG1	3978	24.92	23.76	14.98	16.78	0.62	0.00	0.00
LIMA1	51474	7.45	8.27	3.46	6.04	0.72	0.00	0.00
LIME1	54923	66.68	65.94	27.54	36.19	1.02	0.00	0.00
LIMK2	3985	13.15	13.74	9.12	8.49	0.61	0.00	0.00
LIMS1	3987	9.12	8.25	4.85	6.51	0.65	0.00	0.00
LIMS3-LOC440895	100271835	0.1	0.08	0.01	0	2.61	0.00	0.00
LIMS4	100288695	0.85	0.5	0.52	0.36	1.18	0.00	0.00
LIN7C	55327	1.36	1.61	0.6	0.96	0.93	0.00	0.00

LINC00216	55451	0.98	1.25	0.49	0.71	0.91	0.00	0.00
LINC00426	100188949	51.14	50.52	23.81	26.35	1.02	0.00	0.00
LINC00526	147525	1.01	1.18	0.64	0.74	0.67	0.00	0.00
LINC00639	283547	0.33	0.39	0.12	0.14	1.49	0.00	0.00
LINC00992	728342	0.84	1.18	0.2	0.17	2.03	0.00	0.00
LINC01003	100128822	2.74	2.94	1.53	2.13	0.63	0.00	0.00
LINC01099	101928656	0	0	0.1	0	0.89	0.00	0.00
LINC01106	151009	0.19	0.2	0.09	0.03	1.81	0.00	0.00
LINC01138	388685	0.12	0.16	0.06	0.12	0.83	0.00	0.00
LINC01145	103091866	0.02	0.02	0.02	0	2.14	0.00	0.00
LINC01238	102723927	0	0	0	0	1.49	0.00	0.00
LINC01260	79015	0.03	0.05	0.01	0	0.98	0.00	0.00
LINC01348	731656	1.65	1.39	1	0.7	0.84	0.00	0.00
LINC01473	101927217	1.22	0.65	0.47	0.44	0.99	0.00	0.00
LINC01605	100507420	3.32	2.45	1.76	1.97	0.64	0.00	0.00
LINC01637	101928891	1.28	1.2	1.06	0.65	0.92	0.00	0.00
LINC01770	102724312	1.94	1.57	1.46	0.21	1.26	0.00	0.00
LINC01814	101929567	0.27	0.26	0.1	0.06	1.67	0.00	0.00
LINC01881	728323	0.75	0.76	0.44	0.46	0.87	0.00	0.00
LINC01945	101928067	0.12	0.06	0	0.02	3.28	0.00	0.00
LINC01948	102467147	0.2	0.19	0.07	0.07	1.39	0.00	0.00
LINC01952	105375261	0.14	0.63	0.41	0.01	0.90	0.00	0.00
LINC02125	101928203	0	0	0	0	4.13	0.00	0.00
LINC02256	100996255	0.06	0.05	0	0.07	2.49	0.00	0.00
LINC02263	105377390	0	0	0	0	0.68	0.00	0.00
LINC02323	105370681	1.54	1.45	0.97	1	0.65	0.00	0.00
LINC02363	728175	0.28	0.3	0.09	0.19	1.39	0.00	0.00
LINC02365	105377586	1.9	1.94	0.6	1.1	1.13	0.00	0.00
LINC02408	100507175	0	0	0.02	0	0.72	0.00	0.00
LINC02487	441178	0.17	0.2	0.07	0.02	2.08	0.00	0.00
LINC02535	101928820	1.42	1.22	0.44	0.48	1.52	0.00	0.00
LINC02542	102724168	1.16	1.05	0.54	0.85	0.64	0.00	0.00
LIX1L-AS1	105371260	0.72	0.38	0.31	0.23	0.72	0.00	0.00

LMNB1	4001	39.37	44.25	20.71	23.88	0.85	0.00	0.00
LMTK3	114783	3.02	2.77	1.82	1.11	1.06	0.00	0.00
LNCOC1	100288181	0.03	0.04	0	0	4.68	0.00	0.00
LNPEP	4012	0.69	0.75	0.25	0.5	0.93	0.00	0.00
LNPK	80856	1.9	1.53	1.02	1.2	0.59	0.00	0.00
LOXL4	84171	10.38	10.94	8.03	4.66	0.75	0.00	0.00
LPGAT1	9926	1.46	0.69	0.84	0.43	0.90	0.00	0.00
LRBA	987	5.53	5.52	2.8	4.37	0.70	0.00	0.00
LRIF1	55791	3.14	3.45	1.64	2.53	0.71	0.00	0.00
LRIG2	9860	1.25	1.07	0.74	0.56	0.80	0.00	0.00
LRP6	4040	1.73	1.74	0.71	1.18	0.87	0.00	0.00
LRRC3	81543	6.36	6.41	3.21	2.54	1.11	0.00	0.00
LRRC37A2	474170	0.39	0.6	0.2	0.27	1.37	0.00	0.00
LRRC8B	23507	1.83	1.93	0.97	1.4	0.64	0.00	0.00
LRRFIP1	9208	4.04	4.16	2.62	3.13	0.62	0.00	0.00
LSM11	134353	1.15	1.03	0.72	0.69	0.63	0.00	0.00
LSM12	124801	41.51	41.39	45.13	43.09	3.85	0.00	0.00
LSM6	11157	19.38	18.97	11.95	13.37	0.61	0.00	0.00
LUC7L	55692	11.61	12.28	7.5	6.98	0.73	0.00	0.00
LYAR	55646	14.21	13.74	7.91	10.56	0.59	0.00	0.00
LYNX1	66004	4.15	4.41	2.41	2.12	0.92	0.00	0.00
LYRM7	90624	1.3	0.88	0.58	0.98	0.68	0.00	0.00
LYZ	4069	927.13	968.79	206.66	229.36	2.12	0.00	0.00
LZTFL1	54585	1.98	2.07	0.83	0.63	1.41	0.00	0.00
MAL2	114569	51.09	51.75	23.13	35.69	0.81	0.00	0.00
MAML3	55534	0.73	1	0.27	0.01	2.71	0.00	0.00
MAMSTR	284358	12.94	14.28	9.55	5.27	0.84	0.00	0.00
MAN1A1	4121	71.63	69.93	41.34	46.41	0.71	0.00	0.00
MAP2K6	5608	0.48	0.78	0.25	0.12	1.95	0.00	0.00
MAP3K1	4214	3.15	3.3	0.93	1.81	1.25	0.00	0.00
MAP3K2	10746	0.19	0.1	0.08	0.02	1.61	0.00	0.00
MAP3K4	4216	3.51	3.43	1.76	1.98	0.88	0.00	0.00
MAP3K8	1326	3.98	4.32	3.29	2.65	1.08	0.00	0.00

MAP4K4	9448	74.64	77.27	48.35	41.19	0.77	0.00	0.00
MARVELD3	91862	14.18	14.95	7.97	10.39	0.66	0.00	0.00
MAT2A	4144	35.85	39.17	20.44	13.25	1.12	0.00	0.00
MBLAC2	153364	1.14	1.06	0.43	0.76	0.88	0.00	0.00
MBNL3	55796	21.94	21.79	10.82	16.51	0.79	0.00	0.00
MBP	4155	4.84	4.92	2.84	3.23	0.77	0.00	0.00
MBTD1	54799	2.67	2.36	1.2	1.67	0.80	0.00	0.00
MBTPS1	8720	46.04	44.72	31.27	26.27	0.66	0.00	0.00
MCCC1	56922	10.82	9.76	5.25	7.91	0.66	0.00	0.00
MCHR1	2847	1.97	2.26	1.34	1.01	0.85	0.00	0.00
MCIDAS	345643	0	0	0.06	0.11	0.77	0.00	0.00
MCM3AP-AS1	114044	0.7	0.88	0.38	0.17	1.61	0.00	0.00
MCM4	4173	77.89	76.04	36.63	48.09	0.80	0.00	0.00
MCM5	4174	112.36	109.46	67.99	70.34	0.68	0.00	0.00
MDK	4192	447.79	449.2	278.25	187.47	0.96	0.00	0.00
MDM1	56890	0.61	0.86	0.2	0.34	1.31	0.00	0.00
MDM2	4193	9.16	10.02	4.87	7.77	0.60	0.00	0.00
MDN1	23195	1.67	1.61	0.43	0.93	1.60	0.00	0.00
MED1	5469	4.51	4.95	2.8	3.94	0.61	0.00	0.00
MED7	9443	3.33	2.43	1.22	1.77	1.09	0.00	0.00
MEGF6	1953	1.95	2.15	0.49	0.41	1.98	0.00	0.00
MEGF8	1954	8.8	8.1	5.94	5.04	0.62	0.00	0.00
MELK	9833	9.4	9.06	5.15	6.83	0.60	0.00	0.00
MELTF	4241	7.69	8.55	4.82	2.76	1.05	0.00	0.00
MERTK	10461	14.73	14.4	6.36	6.52	1.23	0.00	0.00
MEST	4232	28.71	30.06	16.58	13.26	0.94	0.00	0.00
METTL14	57721	1.51	1.41	0.52	1.02	0.87	0.00	0.00
MFAP1	4236	4.78	5.38	2.73	3.13	0.79	0.00	0.00
MFF	56947	75.83	73.94	56.13	56.97	0.70	0.00	0.00
MFSD8	256471	1.3	1.51	0.83	1.25	0.87	0.00	0.00
MGA	23269	1.3	1	0.73	0.8	0.65	0.00	0.00
MGAT5	4249	11.32	12	6.15	7.04	0.82	0.00	0.00
MGC32805	153163	0.75	0.7	0.01	0.39	2.21	0.00	0.00

MIER3	166968	1.12	0.93	0.45	0.78	0.68	0.00	0.00
MIOS	54468	1.77	1.67	0.75	1.3	0.72	0.00	0.00
MIR210HG	100506211	0.83	0.47	0.21	0.47	0.84	0.00	0.00
MIR34AHG	106614088	0.42	0.37	0.26	0.18	0.86	0.00	0.00
MIR4453HG	54553	0.84	0.38	0.2	0.28	1.54	0.00	0.00
MIR663AHG	284801	0	0	0	0	0.73	0.00	0.00
MIR762HG	101928736	0.18	0.76	0	0	4.89	0.00	0.00
MIS18A	54069	11.19	10.05	4.72	5.24	1.08	0.00	0.00
MIS18BP1	55320	0.75	0.82	0.3	0.64	0.75	0.00	0.00
MKNK1	8569	14.66	13.12	9.82	8.28	0.69	0.00	0.00
MLH3	27030	1.01	1.28	0.48	0.43	1.00	0.00	0.00
MLIP	90523	0.62	0.93	0.15	0.18	2.15	0.00	0.00
MLLT1	4298	24.16	23.37	27.4	27.69	0.71	0.00	0.00
MLLT3	4300	0.76	1.27	0.42	0.63	0.88	0.00	0.00
MLLT6	4302	33.05	28.9	12.48	17.47	1.04	0.00	0.00
MMACHC	25974	14.05	14.05	7.92	8.97	0.74	0.00	0.00
MMS22L	253714	1.19	0.93	0.52	0.48	1.12	0.00	0.00
MOB3C	148932	2.49	2.12	1.12	1.27	0.94	0.00	0.00
MORN2	729967	8.56	9.03	6.36	5.08	0.62	0.00	0.00
MORN3	283385	6.84	8.65	3.12	2.95	1.02	0.00	0.00
MPC2	25874	41.42	41.62	16.46	33.04	0.73	0.00	0.00
MPHOSPH6	10200	11.44	14.11	5.74	10.59	0.65	0.00	0.00
MPP5	64398	2.12	2.31	1.19	1.62	0.66	0.00	0.00
MPP7	143098	0.81	1.36	0.15	0.45	1.74	0.00	0.00
MPZ	4359	15.91	15.71	11.21	6.18	0.88	0.00	0.00
MRE11	4361	1.39	1.57	0.73	0.86	0.87	0.00	0.00
MREG	55686	7.58	6.97	4.03	4.2	1.58	0.00	0.00
MRFAP1L1	114932	24.33	25.42	15.75	16.75	0.61	0.00	0.00
MROH6	642475	4.89	4.54	1.83	1.45	1.53	0.00	0.00
MRPL1	65008	6.65	6.34	3	4.4	0.81	0.00	0.00
MRPL30	51263	26.05	24.05	15.23	15.35	0.65	0.00	0.00
MRPS31	10240	7.56	7.95	4.67	5.73	0.59	0.00	0.00
MRTFB	57496	2.29	2.44	1.14	1.75	0.70	0.00	0.00

MSH2	4436	6.53	6.75	2.38	4.15	1.02	0.00	0.00
MSH3	4437	1.67	1.81	0.8	1.43	0.66	0.00	0.00
MSH5-SAPCD1	100532732	0.68	0.98	0	0.17	3.28	0.00	0.00
MSH6	2956	15.38	15.54	7.16	9.09	0.97	0.00	0.00
MSL2	55167	3.49	2.82	1.86	1.98	0.69	0.00	0.00
MSLN	10232	9.18	8.57	5.1	3.39	1.10	0.00	0.00
MSRA	4482	9.88	10.87	5.73	6.76	0.78	0.00	0.00
MTERF1	7978	0.75	0.91	0.28	0.41	0.95	0.00	0.00
MTERF2	80298	2.8	2.24	0.94	1.38	0.86	0.00	0.00
MTF1	4520	0.56	0.62	0.13	0.23	1.69	0.00	0.00
MTIF2	4528	7.16	8.32	2.93	5.24	0.92	0.00	0.00
MTR	4548	1.9	1.66	0.97	1.25	0.80	0.00	0.00
MTSS1	9788	9.69	9.36	3.87	6.51	0.89	0.00	0.00
MTTP	4547	20.54	21.38	5.51	7.9	1.64	0.00	0.00
MTURN	222166	2.34	2.44	1.19	0.86	1.24	0.00	0.00
MTUS1	57509	6.45	7.01	3.41	5.35	0.71	0.00	0.00
MTX2	10651	52.29	51.38	26.34	22.94	1.07	0.00	0.00
MUC3A	4584	0.77	0.77	0.38	0.62	0.64	0.00	0.00
MX1	4599	3.76	3.87	0.89	0.95	2.01	0.00	0.00
MYBPC3	4607	0.03	0	0.06	0.03	0.70	0.00	0.00
MYBPHL	343263	12.35	10.57	5.26	5.55	1.00	0.00	0.00
MYH10	4628	14.99	14.15	6.99	7.2	1.03	0.00	0.00
MYH14	79784	15.99	16	8.65	6.51	1.08	0.00	0.00
MYH15	22989	0.06	0.62	0.03	0.12	1.37	0.00	0.00
MYH3	4621	0.2	0.54	0.07	0.32	1.19	0.00	0.00
MYLK4	340156	0.3	0.42	0.09	0.28	1.19	0.00	0.00
MYNN	55892	1.29	0.84	0.58	0.92	0.60	0.00	0.00
MYO10	4651	8.7	8.41	1.8	1.88	2.20	0.00	0.00
MYO18B	84700	0.28	0.23	0.05	0.02	2.91	0.00	0.00
MYO1B	4430	10.44	10.72	5.38	8.33	0.70	0.00	0.00
MYO5B	4645	8.29	7.74	3.57	5.12	0.89	0.00	0.00
MYO5C	55930	2.76	2.46	0.73	1.72	1.08	0.00	0.00
N4BP2	55728	0.4	0.22	0.09	0.12	1.51	0.00	0.00

N4BP2L2	10443	2.78	2.36	2.24	2.78	0.59	0.00	0.00
NANOS1	340719	0.42	0.48	0.4	0.35	0.75	0.00	0.00
NBEA	26960	0.21	0.14	0.08	0.05	1.70	0.00	0.00
NBN	4683	1.78	1.79	0.75	1.56	0.61	0.00	0.00
NBR1	4077	24.14	24.85	14.05	15.78	0.71	0.00	0.00
NCAPG2	54892	10.29	9.64	5.58	6.4	0.73	0.00	0.00
NCBP1	4686	7.42	8.41	3.41	5.71	0.71	0.00	0.00
NCBP2	22916	18.59	21.09	9.32	9.31	1.07	0.00	0.00
NCK1	4690	2.86	2.71	1.41	2.14	0.83	0.00	0.00
NCL	4691	215.39	211.04	100.32	136.16	0.81	0.00	0.00
NCMAP	400746	8.35	8.05	5.14	4.23	0.79	0.00	0.00
NCOA1	8648	2.06	2.13	1.1	1.51	0.63	0.00	0.00
NCOA2	10499	2	2.19	0.84	1.87	0.63	0.00	0.00
NCOA3	8202	3.99	4.43	1.41	2.32	1.11	0.00	0.00
NCOA4	8031	28.86	37.36	8.56	13.59	1.59	0.00	0.00
NCOA7	135112	2.01	2.08	0.88	1.67	0.77	0.00	0.00
NCR3LG1	374383	5.46	5.02	2.65	4.08	0.62	0.00	0.00
NDRG2	57447	23.13	21.51	21.63	26.57	0.80	0.00	0.00
NDUFB1	4707	54.02	51.53	30.27	33.69	0.77	0.00	0.00
NDUFB2	4708	63.97	67.21	62.27	62.83	0.80	0.00	0.00
NEB	4703	0.26	0.41	0.02	0.05	2.85	0.00	0.00
NEBL	10529	0	0	0	0.01	0.90	0.00	0.00
NEDD9	4739	88.57	87.03	50.33	27.97	1.21	0.00	0.00
NEK11	79858	0.74	0.98	0.49	0.4	0.95	0.00	0.00
NEK2	4751	5.65	6.01	3.23	4.25	0.66	0.00	0.00
NEK3	4752	8.72	9.66	5.75	6.12	0.65	0.00	0.00
NEK4	6787	2.26	1.85	1	1.35	0.64	0.00	0.00
NEMP2	100131211	2.08	1.69	1.49	1	0.74	0.00	0.00
NEO1	4756	3.39	3.34	0.7	0.72	2.27	0.00	0.00
NEURL3	93082	2.58	2.5	0.54	0.21	2.74	0.00	0.00
NFATC2	4773	5.6	4.98	0.94	1.73	1.98	0.00	0.00
NFATC2IP	84901	12.04	12.08	6.13	7.55	0.82	0.00	0.00
NFE2	4778	12.6	12.73	3.75	2.23	1.46	0.00	0.00

NFE2L3	9603	6.26	6.64	2.84	1.96	1.40	0.00	0.00
NFIC	4782	6.7	6.64	7.95	6.82	0.67	0.00	0.00
NFKB1	4790	19.56	19.49	9.9	10.61	0.94	0.00	0.00
NFKBID	84807	1.85	2.38	1.35	1.17	0.62	0.00	0.00
NFKBIZ	64332	5.48	5.6	3.32	1.17	1.28	0.00	0.00
NFU1	27247	18.33	19.93	10.56	14.01	0.64	0.00	0.00
NFXL1	152518	1.59	1.45	0.41	1.21	0.90	0.00	0.00
NGLY1	55768	6.91	8.26	4.03	5.14	0.66	0.00	0.00
NHLRC2	374354	0.66	1.26	0.84	0.39	0.98	0.00	0.00
NIFK	84365	13.2	14.47	7.03	10.12	0.69	0.00	0.00
NINL	22981	10.88	12.18	6.92	6.98	0.81	0.00	0.00
NIPAL1	152519	0.49	0.46	0.06	0.09	2.60	0.00	0.00
NIPAL2	79815	0.77	1.08	0.45	0.71	0.73	0.00	0.00
NIPSNAP3B	55335	0.57	0.36	0.21	0.11	1.68	0.00	0.00
NKD2	85409	1.8	2.45	1.33	0.95	0.90	0.00	0.00
NKG7	4818	1.11	0.7	1.73	0.09	1.16	0.00	0.00
NKRF	55922	2.47	2.85	1.57	1.71	0.74	0.00	0.00
NME7	29922	2.55	2.82	1.58	1.3	0.94	0.00	0.00
NME9	347736	0.12	0.46	0.03	0.19	1.72	0.00	0.00
NMI	9111	1.55	1.99	0.39	0.67	1.96	0.00	0.00
NMRAL1	57407	1.27	1.84	0.81	0.43	1.22	0.00	0.00
NMRK1	54981	5.36	6.04	4.08	4.03	0.68	0.00	0.00
NOC3L	64318	1.89	2.16	0.92	1.03	1.08	0.00	0.00
NOL11	25926	21.84	20.82	9.61	15.68	0.75	0.00	0.00
NOL7	51406	74.5	72.48	43.05	48.21	0.70	0.00	0.00
NOP14-AS1	317648	3.72	4.66	2.73	2.85	0.69	0.00	0.00
NORAD	647979	19.67	20.9	8.52	11.42	1.01	0.00	0.00
NOTCH1	4851	1.94	2.05	0.8	0.59	1.49	0.00	0.00
NPHP4	261734	13.17	13.33	8.64	6.69	0.82	0.00	0.00
NPM1	4869	453.51	474.5	269.62	309.05	0.68	0.00	0.00
NPR1	4881	0.08	0.04	0.06	0.04	0.82	0.00	0.00
NR1D2	9975	1.63	1.71	0.8	1	0.91	0.00	0.00
NR1H4	9971	9.21	10.83	2.53	6.02	1.23	0.00	0.00

NR2C2	7182	5.95	6.72	2.98	3.32	0.91	0.00	0.00
NRARP	441478	13.18	13.7	11.41	5.29	0.67	0.00	0.00
NRBF2	29982	9.79	10.99	7.04	6.88	0.58	0.00	0.00
NRG4	145957	1.51	1.58	0.68	0.9	0.88	0.00	0.00
NSA2	10412	24.15	28.76	15.77	16.51	0.69	0.00	0.00
NSL1	25936	15.34	15.46	6.46	7.83	0.98	0.00	0.00
NT5DC4	284958	1.3	2.13	0.51	0.37	1.54	0.00	0.00
NT5E	4907	11.59	12.16	2.14	4.19	1.91	0.00	0.00
NTAN1	123803	37.46	35.26	28.11	17.91	0.64	0.00	0.00
NUBPL	80224	2.1	2.08	1.12	1.59	0.83	0.00	0.00
NUCKS1	64710	23.47	24.66	12.25	14.9	0.83	0.00	0.00
NUDT21	11051	23.64	24.18	13.94	16.69	0.71	0.00	0.00
NUDT4B	440672	1.85	2.06	0.93	1.38	0.77	0.00	0.00
NUDT6	11162	3.58	3.62	2.27	2.04	0.73	0.00	0.00
NUF2	83540	1.8	1.76	1.18	1.66	0.78	0.00	0.00
NUFIP2	57532	2.94	3.35	1.21	1.73	0.84	0.00	0.00
NUP107	57122	3.95	4.19	1.56	2.51	0.90	0.00	0.00
NUP35	129401	8.7	9.74	5.36	6.87	0.62	0.00	0.00
NUP50	10762	6.07	5.7	2.85	4.19	0.79	0.00	0.00
NUP54	53371	8.54	8.3	3.85	5.44	0.86	0.00	0.00
NUSAP1	51203	17.97	16.82	8.94	11.19	0.80	0.00	0.00
NXT2	55916	2.37	2.29	0.86	1.99	0.71	0.00	0.00
NYX	60506	0.12	0.23	0.02	0	4.22	0.00	0.00
OAS3	4940	7.44	7.65	4.16	5.43	0.65	0.00	0.00
OBSL1	23363	73.65	72.59	44.79	32.06	0.96	0.00	0.00
OCLN	100506658	2.32	2.92	1.47	1.89	0.77	0.00	0.00
OFD1	8481	1.21	1.51	0.72	1.29	0.60	0.00	0.00
OGFRL1	79627	0.13	0.62	0.07	0.19	1.61	0.00	0.00
OIP5	11339	22.87	24.75	14.1	16.06	0.65	0.00	0.00
OMA1	115209	2.38	2.84	1.54	1.66	0.69	0.00	0.00
ORC2	4999	9.37	9.05	4.95	6.38	0.70	0.00	0.00
ORC3	23595	3.63	4.29	2.13	2.74	0.66	0.00	0.00
ORC5	5001	7.32	8.05	3.56	4.99	0.86	0.00	0.00

OSBPL9	114883	25.13	25.19	14.21	17.3	0.68	0.00	0.00
OSGEPL1	64172	2.26	1.89	1.19	1.15	0.86	0.00	0.00
OTUD3	23252	2.77	3.12	1.71	1.24	1.03	0.00	0.00
OTUD7B	56957	3.57	3.78	1.92	2.8	0.64	0.00	0.00
OXR1	55074	0.93	1.15	0.42	0.8	0.81	0.00	0.00
PAGE5	90737	0.08	0	0	0	0.66	0.00	0.00
PAK1	5058	2.23	1.63	0.9	0.72	1.29	0.00	0.00
PALB2	79728	3.61	3.17	1.72	2.35	0.65	0.00	0.00
PALM2	114299	0.08	0.05	0.02	0	2.40	0.00	0.00
PALM2-AKAP2	445815	0.26	0.4	0	0.14	2.13	0.00	0.00
PALMD	54873	8.34	8.32	3.48	7.01	0.68	0.00	0.00
PAXIP1	22976	9.32	8.62	5.28	6.15	0.65	0.00	0.00
PAXIP1-AS1	202781	3.28	3.95	2.02	2.12	0.81	0.00	0.00
PBK	55872	6.11	6.12	3.56	4.58	0.58	0.00	0.00
PBRM1	55193	2.32	2.15	0.93	1.76	0.75	0.00	0.00
PCDH9-AS2	100874064	0.26	0.05	0.03	0	3.82	0.00	0.00
PCLAF	9768	27.96	27.14	13.68	17.06	0.86	0.00	0.00
PCMTD2	55251	5.4	6.27	3.37	3.35	0.81	0.00	0.00
PCNT	5116	3.27	3.67	2.07	2.94	0.62	0.00	0.00
PCSK5	5125	0.89	1.2	0.19	0.07	3.12	0.00	0.00
PCTP	58488	18.42	18.65	8.53	8.07	1.12	0.00	0.00
PCYOX1	51449	18.45	19.31	11.25	13.67	0.59	0.00	0.00
PCYT1B	9468	3.73	4.28	1.87	2.04	1.28	0.00	0.00
PDCL	5082	3.87	3.89	2.66	2.06	0.75	0.00	0.00
PDK1	5163	7.37	6.5	4.26	3.78	0.80	0.00	0.00
PDS5A	23244	5.52	4.89	2.36	5.1	0.63	0.00	0.00
PDZK1	5174	13.47	13.28	5.61	10.89	0.70	0.00	0.00
PEG3	5178	24.88	23.25	13.38	15.81	0.72	0.00	0.00
PELI3	246330	1.91	2.97	1.61	1.01	0.90	0.00	0.00
PEX11A	8800	1.48	1.29	0.8	0.84	0.75	0.00	0.00
PEX2	5828	7.56	9.23	5.18	4.22	0.72	0.00	0.00
PFKL	5211	133.24	125.65	72.88	65.72	0.90	0.00	0.00
PGBD1	84547	1.39	1.28	0.78	0.76	0.80	0.00	0.00

PGBD2	267002	0.82	1.49	0.67	0.5	0.90	0.00	0.00
PGC	5225	326.4	311.76	32.87	30.34	3.34	0.00	0.00
PGM2	55276	5.84	6.15	3.37	4.35	0.69	0.00	0.00
PGM5P2	595135	0.02	0.02	0	0	1.91	0.00	0.00
PHAX	51808	1.73	2.29	0.9	1.29	0.76	0.00	0.00
PHF20	51230	5.18	5.11	2.69	2.42	0.97	0.00	0.00
PHF6	84295	1.39	1.26	0.63	0.93	0.60	0.00	0.00
PHKB	5257	13.04	15.2	7.98	9.79	0.71	0.00	0.00
PHLPP1	23239	7.68	8.05	4.46	6.15	0.60	0.00	0.00
PHOSPHO2-KLHL23	100526832	4.91	6.24	2.32	3.29	0.87	0.00	0.00
PHTF2	57157	1.05	0.89	0.57	0.83	0.75	0.00	0.00
PI4K2B	55300	5.39	4.96	2.24	3.51	0.79	0.00	0.00
PIEZO2	63895	1.69	1.75	0.71	0.77	1.23	0.00	0.00
PIGM	93183	2.39	2.45	1.67	1.55	0.59	0.00	0.00
PIGN	23556	3.74	4.43	1.86	2.46	0.91	0.00	0.00
PIGW	284098	7.09	6.7	2.72	4.61	0.95	0.00	0.00
PIGY	84992	21.1	21.99	12.59	13.68	0.71	0.00	0.00
PIGZ	80235	21.53	19.51	12.09	8.04	1.04	0.00	0.00
PIH1D2	120379	0.29	0.46	0.11	0.09	2.83	0.00	0.00
PIK3AP1	118788	2.07	2.3	0.62	0.86	1.60	0.00	0.00
PIK3R1	5295	1.96	2.2	0.48	1.24	1.27	0.00	0.00
PIK3R3	8503	1.05	1.1	0.46	0.77	0.87	0.00	0.00
PIK3R4	30849	2.74	3.1	1.53	2.2	0.68	0.00	0.00
PKD1P4-NPIPA8	110006323	2.3	2.28	1.19	1.17	0.99	0.00	0.00
PKD2	5311	0.76	0.63	0.35	0.41	0.78	0.00	0.00
PKN2	5586	1.84	2.28	1.03	1.38	0.72	0.00	0.00
PKNOX1	5316	2.71	2.65	1.22	1.61	0.89	0.00	0.00
PLAG1	5324	0.36	0.36	0.12	0.24	1.05	0.00	0.00
PLBD1	79887	0.15	0.06	0	0	8.53	0.00	0.00
PLCD1	5333	5.69	4.48	3.09	2.34	0.90	0.00	0.00
PLD1	5337	4.24	4.71	3.22	2.78	0.60	0.00	0.00
PLEKHA6	22874	5.86	6.2	4	5.22	0.97	0.00	0.00
PLEKHA7	144100	6.86	6.51	4.04	5.18	0.58	0.00	0.00

PLEKHB1	58473	106.81	105.34	64.5	46.38	0.94	0.00	0.00
PLEKHF2	79666	1.72	1.61	0.77	1.32	0.68	0.00	0.00
PLK4	10733	1.23	1.41	0.59	1.15	0.62	0.00	0.00
PLPP1	8611	10.38	9.56	3.08	3.77	1.57	0.00	0.00
PLXDC2	84898	0	0.07	0.04	0	1.69	0.00	0.00
PM20D2	135293	4.63	4.9	1.7	1.85	1.46	0.00	0.00
PMM1	5372	34.78	38.68	34.4	30.1	1.11	0.00	0.00
PNCK	139728	9.31	10.4	8.7	1.23	0.89	0.00	0.00
PNPLA3	80339	9.86	9.98	4.25	6.21	0.87	0.00	0.00
PNRC2	55629	34.39	34.26	14.04	26.31	0.76	0.00	0.00
POLA1	5422	3.12	3.58	1.46	2.5	0.86	0.00	0.00
POLD3	10714	3.53	3.68	2.22	2.08	0.73	0.00	0.00
POLE	5426	11.61	10.88	7.16	7.25	1.49	0.00	0.00
POLE3	54107	55.33	55.02	33.83	37	0.64	0.00	0.00
POLN	353497	1.44	1.88	1.48	0.87	0.79	0.00	0.00
POLR2B	5431	15.81	15.79	9.1	12.12	0.59	0.00	0.00
POLR2M	81488	3.74	3.4	1.68	2.09	0.92	0.00	0.00
POLR3B	55703	2.26	2.55	1.71	1.66	0.69	0.00	0.00
POMZP3	22932	3.51	3.9	2.18	2.14	0.79	0.00	0.00
POP1	10940	2.89	2.89	1.93	1.7	0.68	0.00	0.00
POU2F1	5451	1.63	1.19	0.88	0.93	0.71	0.00	0.00
PPARA	5465	4.05	3.27	2.35	2.49	0.59	0.00	0.00
PPARGC1A	10891	1.4	1.34	0.14	0.37	2.37	0.00	0.00
PPIL6	285755	1.15	0.82	0.5	0.19	1.47	0.00	0.00
PPIP5K2	23262	1.16	1.24	0.44	1.11	0.62	0.00	0.00
PPL	5493	3.79	3.05	1.63	1.63	1.06	0.00	0.00
PPP1CB	5500	9.92	10.49	5.14	7.66	0.66	0.00	0.00
PPP1R14D	54866	3.08	2.52	2.57	1.32	0.90	0.00	0.00
PPP1R2	5504	9.62	8.57	4.9	5.5	0.83	0.00	0.00
PPP1R21	129285	5.51	4.77	2.79	2.9	0.84	0.00	0.00
PPP1R3C	5507	2.56	2.53	0.94	1.44	1.10	0.00	0.00
PPP2CA	5515	37.97	37.8	30.45	37.07	1.09	0.00	0.00
PPP2R5D	5528	7.1	7.94	2.75	3.19	1.46	0.00	0.00

PPP4R2	151987	9.67	8.37	3.06	5.74	1.00	0.00	0.00
PPP4R3B	57223	5.81	6.11	2.66	4.24	0.78	0.00	0.00
PPP6R3	55291	7.72	8.41	3.65	5.42	0.84	0.00	0.00
PPT2-EGFL8	100532746	1.02	0.89	0.6	0.59	0.68	0.00	0.00
PRB3	5544	0	0.05	0	0	1.79	0.00	0.00
PRC1	9055	22.8	24.26	15.53	15.36	0.62	0.00	0.00
PRDM10	56980	1.19	1.2	0.45	0.9	0.94	0.00	0.00
PRDM15	63977	1.4	1.48	0.95	0.78	0.83	0.00	0.00
PRDM16	63976	0.16	0.18	0.09	0.08	1.17	0.00	0.00
PRELID3A	10650	1.53	2.19	0.53	0.66	1.35	0.00	0.00
PRELID3B	51012	15.24	14.75	7.79	10.23	0.74	0.00	0.00
PRF1	5551	6	5.59	2.64	0.92	1.71	0.00	0.00
PRIM1	5557	14.59	14.25	7.45	8.97	0.86	0.00	0.00
PRIM2	5558	8.55	9.27	5.2	6.02	0.67	0.00	0.00
PRKAB2	5565	6.28	6.23	4.2	3.71	0.64	0.00	0.00
PRKAG1	5571	16.26	18.01	7.93	9.41	0.97	0.00	0.00
PRKAR1B	5575	32.4	32.94	21.23	28.95	0.98	0.00	0.00
PRKCI	5584	3.1	3.46	1.66	2.91	0.64	0.00	0.00
PRKCQ	5588	0.96	0.9	0.14	0.27	1.77	0.00	0.00
PRKD3	23683	1.75	2.12	0.95	1.61	0.66	0.00	0.00
PRKDC	5591	31.45	31.48	17.3	18.72	0.92	0.00	0.00
PRMT3	10196	4.94	5.11	2.24	3.48	0.82	0.00	0.00
PRPF38A	84950	14.92	15.84	9.79	10.27	0.72	0.00	0.00
PRPF38B	55119	2.34	2.01	1.25	1.66	0.62	0.00	0.00
PRPF39	55015	1.69	2.21	0.79	1.01	0.99	0.00	0.00
PRPF6	24148	95.18	96.57	57.59	62.76	0.65	0.00	0.00
PRR14L	253143	1.71	1.75	1.02	0.96	0.80	0.00	0.00
PRRG1	5638	1.28	1.23	0.59	0.57	0.91	0.00	0.00
PRRG2	5639	5.3	4.84	3.09	3.07	0.71	0.00	0.00
PRRG4	79056	1.23	1.6	0.31	0.29	2.16	0.00	0.00
PRSS23	11098	0.1	0	0.16	0.1	3.98	0.00	0.00
PRSS36	146547	1.74	2.19	1.2	0.95	0.84	0.00	0.00
PRXL2C	195827	10.36	11.18	7.02	6.79	0.69	0.00	0.00

PSCA	8000	0	0	0.47	0.29	0.91	0.00	0.00
PSMA4	5685	54.64	57.02	35.38	35.62	0.66	0.00	0.00
PSMB9	5698	2.4	2.24	0.69	1.23	1.27	0.00	0.00
PSMC1	5700	42.87	41.69	26.04	24.38	0.74	0.00	0.00
PSMC2	5701	0.51	1.29	0.25	0.41	1.47	0.00	0.00
PSMC6	5706	18.86	18.97	8.6	13.69	0.76	0.00	0.00
PSMD1	5707	27.66	28.78	15.73	21.12	0.61	0.00	0.00
PSORS1C3	100130889	0.11	0.09	0.03	0.05	1.34	0.00	0.00
PSPC1	55269	30.74	29.64	20.08	19.62	0.59	0.00	0.00
PSRC1	84722	17.83	16.75	12.08	10.24	0.62	0.00	0.00
PSTPIP1	9051	3.44	3.27	0.3	0.74	2.37	0.00	0.00
PTBP2	58155	1.55	0.92	0.4	0.57	1.12	0.00	0.00
PTGFRN	5738	13.04	13.57	7.05	6.69	0.96	0.00	0.00
PTGIR	5739	1.19	3.69	0.71	1.07	1.39	0.00	0.00
PTGR1	22949	7.54	7.91	2.94	2.2	1.65	0.00	0.00
PTGR2	145482	1.14	1.26	0.33	0.47	1.59	0.00	0.00
PTK7	5754	70.87	71.88	52.49	36.34	0.69	0.00	0.00
PTN	5764	0	0.23	0	0	0.74	0.00	0.00
PTPN11	5781	6.69	6.78	3.67	5.08	0.61	0.00	0.00
PTPN12	5782	12.11	11.79	4.08	5.93	1.25	0.00	0.00
PTPN18	26469	25.43	23.43	15.58	16.09	0.67	0.00	0.00
PTPN2	5771	8.58	9.87	4.64	6	0.80	0.00	0.00
PTPN21	11099	0.82	0.82	0.5	0.42	0.92	0.00	0.00
PTPRG	5793	3.16	2.78	1.88	2.09	0.65	0.00	0.00
PTPRH	5794	12.13	11.87	7.8	5.52	0.86	0.00	0.00
PTPRJ	5795	1.68	1.58	0.96	1.23	1.00	0.00	0.00
PURB	5814	1.86	1.71	0.91	1.25	0.72	0.00	0.00
PUS3	83480	4.63	5.08	2.44	3.52	0.65	0.00	0.00
PVRIG	79037	0.16	0.18	0.06	0	2.38	0.00	0.00
PXMP2	5827	27.61	27.48	14.94	19.27	0.71	0.00	0.00
PYCARD	29108	34.17	35.51	17.38	13.21	1.18	0.00	0.00
PYURF	100996939	21.1	21.99	12.59	13.68	0.71	0.00	0.00
QDPR	5860	39.83	40.15	36.3	31.92	0.60	0.00	0.00

QRICH2	84074	0.61	0.79	0.38	0.4	0.92	0.00	0.00
QSER1	79832	1.79	1.67	0.71	1.48	0.67	0.00	0.00
R3HCC1L	27291	1.51	1.85	1.16	1.21	0.72	0.00	0.00
RAB13	5872	0.5	0.4	0	0	4.83	0.00	0.00
RAB15	376267	14	14.84	9.01	9.98	0.59	0.00	0.00
RAB19	401409	0.65	0.74	0.07	0.07	2.07	0.00	0.00
RAB28	9364	4.49	4.65	2.32	3.04	0.77	0.00	0.00
RAB30-AS1	100506233	0.75	0.74	0.47	0.34	1.05	0.00	0.00
RAB33B	83452	1.32	1.07	0.57	0.65	0.95	0.00	0.00
RAB3IP	117177	2.52	2.04	1.22	1.38	0.98	0.00	0.00
RAB4A	5867	26.46	30.44	18.37	18.01	0.72	0.00	0.00
RAB4B-EGLN2	100529264	1.47	0.63	0.48	0.59	0.78	0.00	0.00
RAB6A	5870	32.08	32.61	18.48	22.02	0.65	0.00	0.00
RAB8B	51762	0.86	0.9	0.6	0.51	0.65	0.00	0.00
RACGAP1	29127	17.84	16.41	10.23	11.93	0.63	0.00	0.00
RAD51AP1	10635	2.46	3.46	1.05	2.46	0.75	0.00	0.00
RAD51D	5892	3.91	3.77	2.66	2.36	0.64	0.00	0.00
RALGAPA2	57186	9.03	8.65	4.76	4.55	0.94	0.00	0.00
RALGPS1	9649	3.9	3.63	1.34	1.33	1.41	0.00	0.00
RALGPS2	55103	1.35	1.53	0.75	0.98	0.77	0.00	0.00
RAP1A	5906	13.25	13.11	6.93	10.71	0.60	0.00	0.00
RAPGEF5	9771	1.61	1.39	0.96	0.87	0.71	0.00	0.00
RAPGEF6	51735	0.66	0.68	0.2	0.65	0.71	0.00	0.00
RARRES2	5919	280.78	283.97	101.2	63.17	1.77	0.00	0.00
RARS	5917	24.12	24.42	14.61	17.16	0.61	0.00	0.00
RARS2	57038	20.9	20.72	13.03	14.41	0.61	0.00	0.00
RASA2	5922	0.58	0.52	0.32	0.28	0.96	0.00	0.00
RASSF6	166824	0.18	0.21	0.06	0	2.80	0.00	0.00
RASSF7	8045	20.19	20.07	17.08	18.14	0.63	0.00	0.00
RAVER2	55225	1.61	1.48	0.66	0.7	1.65	0.00	0.00
RBBP9	10741	14.09	14.24	5.87	8.56	0.97	0.00	0.00
RBL1	5933	3.08	3.1	1.38	2.23	0.75	0.00	0.00
RBL2	5934	9.53	8.98	5.08	5.85	0.79	0.00	0.00

RBM12	10137	9.12	8.89	5.18	6.22	0.66	0.00	0.00
RBM12B	389677	0.9	0.8	0.42	0.51	0.62	0.00	0.00
RBM27	54439	1.85	1.87	1.11	1.32	0.61	0.00	0.00
RBM43	375287	0.41	0.65	0.07	0	3.86	0.00	0.00
RBM47	54502	4.79	4.58	1.62	3.28	0.94	0.00	0.00
RBM48	84060	1.98	1.96	0.89	1.45	0.92	0.00	0.00
RBM7	10179	2.79	3.19	1.37	2.04	0.82	0.00	0.00
RBMXL1	494115	2.19	2.47	1.33	1.74	0.61	0.00	0.00
RBPJ	3516	2.97	2.96	1.54	2.18	0.65	0.00	0.00
RC3H1	149041	0.81	0.76	0.16	0.74	0.81	0.00	0.00
RCN1	5954	61.74	63.28	36.73	28.35	0.94	0.00	0.00
RCN2	5955	41.55	47.2	24.23	33.29	0.65	0.00	0.00
RCN3	57333	5.45	5.99	2.43	2.35	1.27	0.00	0.00
RDH14	57665	8.53	8.05	3.82	5.91	0.77	0.00	0.00
RECK	8434	1.33	1.73	0.32	0.21	2.68	0.00	0.00
REL	5966	0.27	0.28	0.06	0.17	1.17	0.00	0.00
RELCH	57614	2.25	2.31	0.96	1.84	0.67	0.00	0.00
RELL1	768211	6.28	6.18	1.83	2.48	1.50	0.00	0.00
REXO5	81691	3.66	3.67	2.59	1.95	0.71	0.00	0.00
RFC1	5981	4.87	5	2.09	3.52	0.80	0.00	0.00
RFC5	5985	17.11	18.03	11.82	11.91	0.63	0.00	0.00
RFK	55312	9.06	9.2	4.89	6.35	0.72	0.00	0.00
RFX7	64864	2.48	2.56	1.05	1.68	0.82	0.00	0.00
RFXAP	5994	2.19	1.66	0.89	1.34	0.81	0.00	0.00
RGN	9104	26.6	27.46	13.01	18.49	0.78	0.00	0.00
RGPD5	84220	0.82	0.69	0.5	0.23	0.92	0.00	0.00
RGPD6	729540	0.66	0.59	0.23	0.65	0.65	0.00	0.00
RGPD8	727851	0.5	0.61	0.2	0.28	1.25	0.00	0.00
RGR	5995	1.98	1.95	0.65	0.28	2.33	0.00	0.00
RHBG	57127	34.11	34.68	19.35	21.43	0.77	0.00	0.00
RHOU	58480	7.47	6.58	3.57	3.18	1.19	0.00	0.00
RIBC1	158787	2.1	1.16	0.74	0.93	1.30	0.00	0.00
RLIM	51132	1.19	1.23	0.74	0.85	0.60	0.00	0.00

RMI2	116028	32.82	31.34	21.26	16.98	0.75	0.00	0.00
RMND1	55005	22.23	21	11.44	11.38	0.90	0.00	0.00
RNASEH2B	79621	7.44	6.79	3.66	5.63	0.70	0.00	0.00
RND1	27289	2.91	1.85	0.83	1.57	0.99	0.00	0.00
RNF128	79589	5.14	5.81	2.51	3.34	0.90	0.00	0.00
RNF130	55819	29.45	29.11	19.02	16.98	0.71	0.00	0.00
RNF138	51444	3.2	2.86	1.75	2.26	0.75	0.00	0.00
RNF144A-AS1	386597	0.73	0.78	0.34	0.52	0.80	0.00	0.00
RNF146	81847	6.05	5.34	2.93	3.38	0.71	0.00	0.00
RNF169	254225	0.6	0.41	0.23	0.3	1.00	0.00	0.00
RNF19A	25897	3.21	3.67	1.69	2.33	0.76	0.00	0.00
RNF20	56254	4.06	4.34	2.13	2.57	0.81	0.00	0.00
RNF207	388591	4.11	3.65	2.76	2.27	0.68	0.00	0.00
RNF38	152006	3.49	3.52	2.28	2.76	0.75	0.00	0.00
RNF39	80352	1.46	1.4	0.56	0.52	1.41	0.00	0.00
RNF8	9025	11.11	13	9.32	10.77	0.74	0.00	0.00
RNGTT	8732	1.82	2.02	1.11	1.19	0.73	0.00	0.00
RP2	6102	1.93	2.44	0.81	1.57	0.75	0.00	0.00
RPAIN	84268	2.1	3.55	2.74	2.81	0.71	0.00	0.00
RPAP2	79871	1.7	1.48	0.81	1.05	0.86	0.00	0.00
RPL22	6146	1141.4	1181.55	849.22	669.13	0.60	0.00	0.00
RPL3	6122	1985.25	2040.18	1891.44	1499.7	0.99	0.00	0.00
RPL36A	6173	294.02	293.89	214.35	163.65	0.64	0.00	0.00
RPL36A-HNRNPH2	100529097	5.4	6.01	3.64	3.73	0.65	0.00	0.00
RPL7	6129	825.45	834.53	540.82	485.07	0.69	0.00	0.00
RPL7L1	285855	68.68	70.93	45.48	44.14	0.61	0.00	0.00
RPL9	6133	34.53	33.94	31.1	26.34	0.65	0.00	0.00
RPS10-NUDT3	100529239	5.93	6.08	4.26	3.94	0.59	0.00	0.00
RPS6KA5	9252	0.34	0.25	0.05	0.03	2.06	0.00	0.00
RPS6KC1	26750	2.04	1.93	0.92	1.21	0.83	0.00	0.00
RRM1	6240	15.93	17.46	7.83	10.55	0.89	0.00	0.00
RRN3	54700	11.02	11.16	5.13	5.77	1.03	0.00	0.00
RRN3P3	100131998	0.13	0.14	0.1	0.15	0.61	0.00	0.00

RSL1D1	26156	4.05	4.59	1.72	2.37	1.07	0.00	0.00
RSPH14	27156	2.15	1.74	0.63	0.55	2.20	0.00	0.00
RSRP1	57035	2.5	2.13	0.8	1.56	1.17	0.00	0.00
RTKN2	219790	0.94	0.98	0.42	0.82	1.23	0.00	0.00
RTL10	79680	5.9	5.89	3.71	3.59	0.69	0.00	0.00
RYBP	23429	2.94	3.27	1.91	2.23	0.59	0.00	0.00
RYK	6259	14.05	14	8.07	7.85	0.80	0.00	0.00
S100A14	57402	69.34	70.52	34.97	25.48	1.20	0.00	0.00
S1PR2	9294	1.27	1.16	0.98	0.56	0.66	0.00	0.00
S1PR5	53637	0.55	0.51	0.15	0.23	1.46	0.00	0.00
SALL1	6299	9.86	10.36	0.33	0.29	5.03	0.00	0.00
SALL2	6297	3.85	3.68	3.1	1.82	0.62	0.00	0.00
SAMD4A	23034	2.22	2.12	1.56	0.56	0.99	0.00	0.00
SAMHD1	25939	10.27	9.7	5	7.34	0.62	0.00	0.00
SAP30L	79685	4.33	4.45	3.52	4.03	0.64	0.00	0.00
SAR1A	56681	19.11	20.41	11.48	11.43	0.65	0.00	0.00
SBF2	81846	5.69	6.27	3.05	4.4	0.58	0.00	0.00
SBK2	646643	5.7	4.94	5.27	1.62	0.66	0.00	0.00
SBSPON	157869	0.02	0	0.01	0.01	0.68	0.00	0.00
SCAMP1-AS1	728769	0.88	0.96	0.63	0.41	0.83	0.00	0.00
SCN11A	11280	0.08	0.02	0	0	4.90	0.00	0.00
SCYL3	57147	1.07	1.16	0.73	0.81	0.66	0.00	0.00
SDCBP2	27111	19.42	18.35	7.11	12.71	0.97	0.00	0.00
SDHD	6392	40.05	41.76	24.5	28.44	0.63	0.00	0.00
SEC11C	90701	50.69	50.47	28.77	36.93	0.63	0.00	0.00
SEC14L2	23541	4	3.43	1.89	2.3	0.72	0.00	0.00
SEC14L4	284904	0.67	0.59	0.31	0.05	2.06	0.00	0.00
SEC14L6	730005	3.6	4.15	1.24	0.45	2.22	0.00	0.00
SECISBP2L	9728	0.64	0.52	0	0.56	1.11	0.00	0.00
SECTM1	6398	5.82	4.2	1.49	2.16	1.37	0.00	0.00
SEH1L	81929	14.29	14.9	5.06	8.79	1.09	0.00	0.00
SEL1L3	23231	16.34	17.05	9.58	6.7	1.04	0.00	0.00
SELENOI	85465	5.06	4.9	2.56	3.33	0.77	0.00	0.00

SEMA4A	64218	1.93	1.53	0.76	0.57	1.01	0.00	0.00
SEMA6A	57556	6.56	6.77	1.33	1.84	2.18	0.00	0.00
SEMA6C	10500	1.97	1.51	1.1	1.21	0.63	0.00	0.00
SENP1	29843	2.63	2.84	1.37	1.94	0.64	0.00	0.00
SENP2	59343	7.76	7.29	3.67	5.9	0.70	0.00	0.00
SENP5	205564	2.22	2.16	1.1	1.53	0.71	0.00	0.00
SEPSECS	51091	0.99	0.95	0.46	0.7	0.76	0.00	0.00
SERBP1	26135	41.22	37.04	21.91	27.7	0.69	0.00	0.00
SERINC1	57515	10.96	11.7	5.61	9.19	0.61	0.00	0.00
SERINC5	256987	7.8	7.18	3.37	4.52	0.96	0.00	0.00
SERPINA3	12	896.75	917.2	665.9	122.86	1.21	0.00	0.00
SERPINA4	5267	31.28	30.98	16.9	12.96	1.07	0.00	0.00
SERPIND1	3053	242.13	241.47	159.38	131.68	0.73	0.00	0.00
SESN1	27244	11.22	11.4	6.29	6.77	0.75	0.00	0.00
SETD6	79918	7.29	7.57	4.45	4.77	0.75	0.00	0.00
SETDB1	9869	9.32	9.4	7.41	5.5	0.59	0.00	0.00
SETDB2	83852	0.35	0.2	0.04	0	3.80	0.00	0.00
SEZ6L2	26470	66.25	65.95	27.57	27.19	1.27	0.00	0.00
SF3B1	23451	24.77	27.05	12.26	17.51	0.77	0.00	0.00
SFR1	119392	1.1	1.55	0.46	0.57	1.25	0.00	0.00
SFXN2	118980	10.79	11.67	5.82	5.86	0.97	0.00	0.00
SGK3	23678	0.39	1.46	0.14	0.93	0.75	0.00	0.00
SGMS1-AS1	104355295	0.3	0.25	0.06	0.12	1.63	0.00	0.00
SGPL1	8879	12.45	13.56	8.71	8.54	0.59	0.00	0.00
SH2D4A	63898	1.99	1.29	0.33	0.35	2.45	0.00	0.00
SH2D6	284948	0.23	0.39	0.02	0.12	2.01	0.00	0.00
SH3BGRL	6451	7.78	8.38	2.38	2.92	1.82	0.00	0.00
SH3BGRL2	83699	7.52	6.96	2.56	4.07	1.13	0.00	0.00
SH3BP4	23677	35.41	34.85	23.96	17.33	0.78	0.00	0.00
SHANK3	85358	5.75	5.39	6.53	5.96	0.76	0.00	0.00
SHLD2	54537	5.96	6.81	2.51	4.15	0.89	0.00	0.00
SHPRH	257218	0.48	0.49	0.15	0.41	0.77	0.00	0.00
SHROOM1	134549	16.5	16.66	7.63	8.11	1.06	0.00	0.00

SHROOM2	357	1.93	1.75	0.7	0.63	1.56	0.00	0.00
SHTN1	57698	7.84	7.59	4.11	5.5	0.68	0.00	0.00
SIAE	54414	4.77	4.1	2.12	3.51	0.66	0.00	0.00
SIGLEC1	6614	0.15	0.23	0.03	0.05	2.33	0.00	0.00
SIK2	23235	8.93	8.3	4.74	5.12	0.74	0.00	0.00
SIRT1	23411	1.64	1.32	0.77	0.96	0.83	0.00	0.00
SIRT5	23408	9.89	8.47	6.34	5.38	0.66	0.00	0.00
SKAP2	8935	6.91	7.17	3.81	5.81	0.64	0.00	0.00
SKIL	6498	0.8	0.8	0.41	0.57	0.71	0.00	0.00
SLC11A2	4891	18.67	19.8	10.94	11.53	0.76	0.00	0.00
SLC12A3	6559	0.84	0.95	0.59	0.28	0.99	0.00	0.00
SLC13A3	64849	40.78	39.67	21.21	12.35	1.32	0.00	0.00
SLC18B1	116843	6.7	6.77	3.48	3.61	0.94	0.00	0.00
SLC22A31	146429	75.73	69.74	22.37	27.6	1.56	0.00	0.00
SLC22A5	6584	5.42	3.86	3.16	3.46	0.60	0.00	0.00
SLC23A1	9963	10.44	8.97	5.84	6.39	0.68	0.00	0.00
SLC25A30	253512	5.95	6	2.76	2.75	1.11	0.00	0.00
SLC25A45	283130	3.41	2.91	2.07	2.03	0.72	0.00	0.00
SLC25A46	91137	2.4	2.5	1.57	2.05	0.62	0.00	0.00
SLC26A5	375611	0.01	0.01	0	0.01	0.69	0.00	0.00
SLC27A2	11001	9.96	11.38	4.63	7.79	0.78	0.00	0.00
SLC29A4	222962	22.83	21.53	16.41	11.39	0.67	0.00	0.00
SLC2A3	6515	76.39	76.12	64.78	36.19	0.60	0.00	0.00
SLC2A4RG	56731	105.31	101.67	59.98	65.81	0.74	0.00	0.00
SLC30A10	55532	1.78	1.83	0.73	1.35	0.71	0.00	0.00
SLC30A3	7781	8.79	8.12	4.57	1.74	1.42	0.00	0.00
SLC30A9	10463	4.75	4.79	2.25	3.76	0.61	0.00	0.00
SLC35A1	10559	2.48	2.97	1.98	1.67	0.65	0.00	0.00
SLC35A3	23443	0.91	0.83	0.32	0.63	0.89	0.00	0.00
SLC35B3	51000	6.23	4.99	3.59	3.48	0.66	0.00	0.00
SLC35E3	55508	7.99	7.08	4.25	6.29	0.64	0.00	0.00
SLC36A4	120103	1.42	1.11	0.57	0.21	1.37	0.00	0.00
SLC38A9	153129	5.02	5.99	2.96	3.75	0.71	0.00	0.00

SLC39A6	25800	4.87	5.07	2.79	3.74	0.59	0.00	0.00
SLC44A1	23446	17.31	17.41	9.02	12.63	0.74	0.00	0.00
SLC44A3	126969	8.56	9.42	1.98	3.45	1.84	0.00	0.00
SLC46A1	113235	9.8	10.02	5.18	5.8	0.86	0.00	0.00
SLC52A3	113278	2.49	1.78	0.77	0.48	1.80	0.00	0.00
SLC5A11	115584	6.69	6.85	2.38	3.05	1.45	0.00	0.00
SLC5A9	200010	16.23	17.1	5.83	9.91	1.09	0.00	0.00
SLC6A11	6538	14.02	14.31	9.07	9.7	0.60	0.00	0.00
SLC6A14	11254	1.64	1.59	0.92	0.75	0.95	0.00	0.00
SLC6A8	6535	91.53	94.58	75.67	45.16	0.67	0.00	0.00
SLC7A7	9056	1.53	2.46	0.79	0.71	1.20	0.00	0.00
SLCO4A1-AS1	100127888	0.91	0.82	0.17	0.12	2.58	0.00	0.00
SLFN13	146857	0	0	0	0.01	1.00	0.00	0.00
SLIRP	81892	94.67	94.39	94.01	105.01	0.72	0.00	0.00
SLMO2-ATP5E	100533975	0	0.41	0	0	5.79	0.00	0.00
SMAD9	4093	1.09	1.01	0.43	0.53	1.15	0.00	0.00
SMARCA5	8467	4.34	5.49	2.06	4.06	0.73	0.00	0.00
SMARCA5-AS1	100128055	1.43	0.83	0.51	0.96	0.62	0.00	0.00
SMARCE1	6605	26.49	25.21	12.98	17.71	0.76	0.00	0.00
SMC1A	8243	8.22	9.68	5.29	6.57	0.61	0.00	0.00
SMC2-AS1	101928550	0.02	0.05	0	0.01	3.60	0.00	0.00
SMCHD1	23347	1.68	1.82	0.7	1.54	0.58	0.00	0.00
SMDT1	91689	5.7	4.55	3.66	3.46	0.60	0.00	0.00
SMG1	23049	2.55	2.43	1.21	1.78	0.74	0.00	0.00
SMIM20	389203	22.02	22.44	13.92	14.11	0.66	0.00	0.00
SMIM5	643008	0	0.08	0	0.17	0.74	0.00	0.00
SMN1	6606	8.31	6.85	4.63	4.75	0.64	0.00	0.00
SMPDL3B	27293	6.65	6.47	3.22	2.78	1.11	0.00	0.00
SMTNL2	342527	1.13	1.44	0.52	0.31	1.59	0.00	0.00
SNAI3	333929	2.17	2.25	1.41	1.26	0.73	0.00	0.00
SNCAIP	9627	0.18	0.11	0	0.03	3.26	0.00	0.00
SNHG14	104472715	7.18	8.12	3.18	4.24	1.05	0.00	0.00
SNHG16	100507246	1.12	1.03	0.74	0.5	0.78	0.00	0.00

SNHG26	109729180	4.41	4.05	1.85	1.28	1.35	0.00	0.00
SNHG9	735301	2.52	1.43	0.79	0.88	1.25	0.00	0.00
SNRK	54861	1.58	1.72	1.05	1.15	0.65	0.00	0.00
SNRNP48	154007	3.97	3.09	1.94	2.3	0.72	0.00	0.00
SNRPB2	6629	34.66	34.45	21.5	22.64	0.65	0.00	0.00
SNRPD2	6633	6.32	4.6	3.21	3.74	0.73	0.00	0.00
SNRPN	6638	103.56	110.61	68.66	65.8	0.67	0.00	0.00
SNTB1	6641	8.5	5.87	3.5	4.56	1.85	0.00	0.00
SNURF	8926	65.71	60.19	40.04	34.18	0.76	0.00	0.00
SNW1	22938	15.93	17.53	9.38	10.38	0.78	0.00	0.00
SNX14	57231	11.36	12.22	5.73	9.4	0.60	0.00	0.00
SNX2	6643	1.3	0.9	0.03	2.51	0.80	0.00	0.00
SNX30	401548	3.34	2.82	1.19	1.87	1.00	0.00	0.00
SNX32	254122	0.81	0.26	0.28	0.16	1.26	0.00	0.00
SNX6	58533	14.13	13.55	7.16	8.51	0.81	0.00	0.00
SNX9	51429	8.61	7.72	3.52	3.8	1.09	0.00	0.00
SOCS5	9655	2.44	2.41	0.9	1.81	0.84	0.00	0.00
SOCS6	9306	2.1	2.2	1.08	1.58	0.70	0.00	0.00
SOD2	6648	147.78	147.31	62.25	52	1.73	0.00	0.00
SON	6651	24.51	23.34	13.68	19.48	0.61	0.00	0.00
SORBS1	10580	2.87	3.09	1.6	2.03	0.71	0.00	0.00
SORBS2	8470	5.67	5.67	1.58	1.83	1.76	0.00	0.00
SORD	6652	75.28	73.82	40	39.92	0.90	0.00	0.00
SORD2P	653381	1.4	1.62	0.93	0.55	1.03	0.00	0.00
SORL1	6653	15.76	15.36	9.78	7.16	0.90	0.00	0.00
SORT1	6272	19.61	19.43	13.2	11.04	0.70	0.00	0.00
SOWAHB	345079	1.42	1.44	0.14	0.37	2.48	0.00	0.00
SOX30	11063	0	0	0	0.05	0.67	0.00	0.00
SOX9-AS1	400618	0	0.02	0.06	0.04	0.66	0.00	0.00
SP110	3431	2.66	2.04	0.3	0.8	2.19	0.00	0.00
SP140L	93349	2.42	2.24	0.72	0.82	1.69	0.00	0.00
SP3	6670	5.7	6	2.37	4.1	0.80	0.00	0.00
SPAG16	79582	5.39	6.3	3.01	3.12	0.83	0.00	0.00

SPAG5	10615	17.49	18	9.42	12.41	0.70	0.00	0.00
SPATA6	54558	0.67	0.42	0.32	0.11	0.98	0.00	0.00
SPC25	57405	7.26	7.16	4.29	5.1	0.61	0.00	0.00
SPECC1	92521	4.26	4.05	1.57	2.33	1.05	0.00	0.00
SPG11	80208	3.39	3.36	2.04	2.42	0.62	0.00	0.00
SPIB	6689	1.31	1.34	0.33	0.73	1.32	0.00	0.00
SPIDR	23514	6.68	6.23	5.69	5.44	1.61	0.00	0.00
SPINT1-AS1	102724362	0.11	0.1	0.03	0	2.88	0.00	0.00
SPIRE1	56907	3.29	3.16	1.23	1.71	1.19	0.00	0.00
SPIRE2	84501	11.41	12.34	7.14	6.35	0.83	0.00	0.00
SPNS1	83985	47.34	46.09	53.26	58.53	1.01	0.00	0.00
SPP1	6696	729.2	744.34	241.64	97.1	2.12	0.00	0.00
SPR	6697	75.72	77.89	41.42	51.61	0.71	0.00	0.00
SPRTN	83932	1.48	1.5	0.69	1.25	0.63	0.00	0.00
SPRYD4	283377	9.38	9.44	4.52	6.03	0.82	0.00	0.00
SPTLC3	55304	9.76	10.08	1.71	5.01	1.53	0.00	0.00
SPTY2D1	144108	1.7	1.6	0.95	1.23	0.59	0.00	0.00
SPTY2D1OS	100506540	0.68	0.92	0.49	0.34	0.99	0.00	0.00
SRBD1	55133	2.7	2.84	1.18	1.27	1.21	0.00	0.00
SRRM3	222183	1.68	1.74	0.47	0.44	1.19	0.00	0.00
SRSF1	6426	53.63	53.96	24.3	33.68	0.90	0.00	0.00
SRSF11	9295	9.33	9.02	3.57	6.17	0.81	0.00	0.00
SRSF3	6428	72.4	77	50.91	48.79	0.59	0.00	0.00
SRSF7	6432	69.81	75.52	47.28	43.41	0.63	0.00	0.00
SS18L1	26039	3.63	4.14	1.97	2.13	0.91	0.00	0.00
SSC4D	136853	34.73	34.75	14.47	5.29	1.96	0.00	0.00
SSX2IP	117178	2	1.79	0.56	1.17	1.07	0.00	0.00
ST8SIA1	6489	0.01	0	0	0	0.62	0.00	0.00
STAG2	10735	2.42	1.94	0.99	1.55	0.75	0.00	0.00
STARD13	90627	2.13	2.61	1.52	1.41	0.65	0.00	0.00
STARD7-AS1	285033	1.91	1.86	1.1	1.22	0.63	0.00	0.00
STAT1	6772	8.75	8.99	3.79	7.06	0.73	0.00	0.00
STMN1	3925	391.31	401.84	258.87	240.12	0.69	0.00	0.00

STRA6	64220	7.91	7.62	3.03	1.31	1.85	0.00	0.00
STX17	55014	1.81	1.8	1.16	1.26	0.59	0.00	0.00
STX18-AS1	100507266	0.3	0.52	0.25	0.17	0.76	0.00	0.00
STX6	10228	9.79	6.74	6.68	5.09	0.66	0.00	0.00
SUB1	10923	84.04	91.67	47.88	60.41	0.79	0.00	0.00
SUDS3	64426	5.97	5.44	3.87	3.81	0.60	0.00	0.00
SULT1A2	6799	38.93	40.78	25.34	24.91	0.66	0.00	0.00
SULT2A1	6822	75.93	77.75	25.78	61.17	0.82	0.00	0.00
SUMF1	285362	16.64	14.98	13.21	11.6	0.71	0.00	0.00
SUMO1	7341	46.19	46.53	29.75	25.84	0.74	0.00	0.00
SUN2	25777	58.34	59.36	43.32	32.69	0.64	0.00	0.00
SUPT16H	11198	16.41	16.14	9.2	9.99	0.76	0.00	0.00
SUSD3	203328	23.27	23.86	7.88	5.08	1.86	0.00	0.00
SUZ12	23512	3.06	2.98	0.97	2.71	0.76	0.00	0.00
SVIP	258010	6.94	5.91	2.4	3.94	1.25	0.00	0.00
SYNC	81493	2.9	3.15	1.04	1.72	1.09	0.00	0.00
SYNE2	23224	0.73	0.84	0.42	0.4	1.19	0.00	0.00
SYNJ2	8871	11.15	12.15	7.41	7.14	0.74	0.00	0.00
SYNJ2BP	55333	1.13	1.39	0.5	0.92	0.83	0.00	0.00
SYNJ2BP-COX16	100529257	0.61	0.79	0.61	0.19	1.21	0.00	0.00
SYT1	6857	0.15	0.12	0	0	5.42	0.00	0.00
SZT2	23334	1.79	1.88	1.15	1.2	0.63	0.00	0.00
TAF1	6872	2.44	1.93	1.11	1.22	0.90	0.00	0.00
TAF1A	9015	0.31	0.37	0.06	0.15	1.57	0.00	0.00
TAF9B	51616	3.3	3.25	1.51	2.39	0.68	0.00	0.00
TAL2	6887	0	0.29	0	0	0.62	0.00	0.00
TANC1	85461	4.03	3.95	1.15	1.1	2.41	0.00	0.00
TARDBP	23435	26.56	25.06	13.47	16.32	0.77	0.00	0.00
TARSL2	123283	2.49	2.03	0.82	1.34	1.04	0.00	0.00
TASP1	55617	6.31	5.64	3.08	3.65	0.77	0.00	0.00
TBC1D1	23216	16.78	16.1	11.81	10.01	0.58	0.00	0.00
TBC1D24	57465	6.11	5.48	3.37	3.85	0.74	0.00	0.00
TBC1D31	93594	0.73	0.72	0.22	0.46	1.13	0.00	0.00

TBC1D4	9882	7.34	7.45	5.71	6.7	0.88	0.00	0.00
TBC1D5	9779	7.57	7.61	4.75	5.3	0.61	0.00	0.00
TBC1D9B	23061	31.75	28.04	18.36	19.34	0.66	0.00	0.00
TBL1XR1	79718	3.23	2.89	1.69	1.94	0.78	0.00	0.00
TCEA3	6920	195.42	195.24	117.92	131.45	0.63	0.00	0.00
TCEAL1	9338	9.29	8.91	5.02	5.13	0.84	0.00	0.00
TCEANC2	127428	2.46	2.54	1.93	2.06	0.64	0.00	0.00
TCERG1	10915	8.31	9.35	4.82	6.32	0.64	0.00	0.00
TCF12	6938	5.27	4.9	2.66	3.97	0.66	0.00	0.00
TCF25	22980	59.2	61.93	45.02	42.72	0.61	0.00	0.00
TCF7L2	6934	4.28	4.98	5.68	5.38	1.09	0.00	0.00
TCP10L2	401285	0.29	0.17	0	0.04	3.90	0.00	0.00
TCTE3	6991	3.78	2.86	1.73	1.46	1.06	0.00	0.00
TCTN2	79867	3.44	3.16	2.13	1.68	0.83	0.00	0.00
TDG	6996	4.24	4.3	2.28	3.01	0.77	0.00	0.00
TDP2	51567	18.59	18.64	10.46	16.18	0.61	0.00	0.00
TDRD7	23424	3.36	3.09	2.24	2.27	0.61	0.00	0.00
TEDC2	80178	12.12	12.37	10.82	11.45	0.99	0.00	0.00
TENT4A	11044	5.43	4.77	3.57	3.37	0.59	0.00	0.00
TERF1	7013	2.32	1.89	1.16	1.33	0.64	0.00	0.00
TESK2	10420	5.08	4.35	2.08	2.49	1.02	0.00	0.00
TET1	80312	0.51	0.49	0.26	0.35	0.69	0.00	0.00
TET3	200424	3.72	3.58	1.29	1.6	1.25	0.00	0.00
TEX10	54881	7.32	7.92	4.34	5.27	0.60	0.00	0.00
TEX2	55852	14.4	14.35	7.51	9.65	0.74	0.00	0.00
TF	7018	2151.58	2223.93	983.09	681.22	1.41	0.00	0.00
TFAM	7019	5.42	5.8	2.63	4.34	0.64	0.00	0.00
TFDP2	7029	7.27	8.24	4.64	4.39	1.06	0.00	0.00
TFEB	7942	9.57	9.83	3.32	2.89	0.98	0.00	0.00
TFRC	7037	38.07	40.03	23.64	25.86	0.69	0.00	0.00
TGFA	7039	10.5	10.49	4.79	4.9	1.12	0.00	0.00
TGFBR2	7048	38.37	41.32	24.98	26.39	0.65	0.00	0.00
TGM4	7047	0.62	0.94	0.31	0.17	1.70	0.00	0.00

TGOLN2	10618	70.14	70.73	44.78	48.86	0.59	0.00	0.00
TGS1	96764	2.52	2.62	1.5	1.79	0.66	0.00	0.00
THAP6	152815	1.21	1.33	0.88	0.69	0.95	0.00	0.00
THG1L	54974	7.07	6.98	4.55	4.77	0.59	0.00	0.00
THSD4	79875	10.8	13.03	4.99	6.31	1.04	0.00	0.00
THSD4-AS1	101929196	4.96	6.69	3.32	3.67	0.74	0.00	0.00
THUMPD3	25917	5.14	5.2	2.67	3.41	0.76	0.00	0.00
TICRR	90381	3.04	3.01	1.46	1.75	0.88	0.00	0.00
TIFA	92610	2.98	3.75	1.42	1.65	1.07	0.00	0.00
TIMM21	29090	7.81	8.37	4.7	5.97	0.58	0.00	0.00
TIMM8A	1678	9.45	8.88	5.62	8.13	0.64	0.00	0.00
TINAG	27283	1.04	1.27	0.1	0	4.60	0.00	0.00
TINAGL1	64129	29.52	28.64	20.08	9.13	1.00	0.00	0.00
TIPIN	54962	5.57	6.63	4.65	4.73	0.71	0.00	0.00
TJP3	27134	13.77	14.15	6.16	11.33	0.66	0.00	0.00
TM2D2	83877	11.51	12.19	13.22	13.68	1.01	0.00	0.00
TM4SF4	7104	3.19	2.66	0.82	2.45	0.84	0.00	0.00
TMA16	55319	7.84	7.82	4.79	5.23	0.64	0.00	0.00
TMC4	147798	9.54	9.45	4.31	2.56	1.42	0.00	0.00
TMCO4	255104	10.2	10.92	6.45	7.49	0.64	0.00	0.00
TMEM117	84216	1.33	1.53	0.53	1.23	0.78	0.00	0.00
TMEM121	80757	4.9	4.42	2.5	1.47	1.21	0.00	0.00
TMEM123	114908	58.62	59.45	31.44	36.91	0.79	0.00	0.00
TMEM135	65084	17.34	18.52	5.45	7.44	1.50	0.00	0.00
TMEM141	85014	101.54	94.87	70.8	49.65	0.69	0.00	0.00
TMEM14A	28978	26.31	26.78	9.4	14.21	1.20	0.00	0.00
TMEM161B-AS1	100505894	0.21	0.26	0.1	0.09	1.17	0.00	0.00
TMEM168	64418	1.14	1.23	0.65	0.71	0.79	0.00	0.00
TMEM17	200728	0.23	0.62	0.47	0.5	0.61	0.00	0.00
TMEM170B	100113407	0.6	0.62	0.28	0.43	0.94	0.00	0.00
TMEM181	57583	6.56	7.13	4.06	4.86	0.63	0.00	0.00
TMEM200A	114801	0.77	0.64	0.11	0.29	1.74	0.00	0.00
TMEM218	219854	7.01	7.8	5.66	4	0.75	0.00	0.00

TMEM260	54916	1.71	1.38	1.37	1.31	0.81	0.00	0.00
TMEM33	55161	8.3	8.97	8.78	10.13	1.10	0.00	0.00
TMEM51-AS1	200197	1.93	1.47	0.98	0.7	0.85	0.00	0.00
TMEM56	148534	0.91	0.8	0.29	0.65	0.85	0.00	0.00
TMEM56-RWDD3	100527978	0.68	0.86	0.3	0.29	1.18	0.00	0.00
TMEM64	169200	2.57	2.66	1.6	1.86	0.60	0.00	0.00
TMEM65	157378	1.18	1.11	1.22	0.61	0.72	0.00	0.00
TMEM87A	25963	4.57	4.55	2.43	2.92	0.77	0.00	0.00
TMLHE	55217	8.38	6.15	5.24	5.28	0.67	0.00	0.00
TMPO	7112	27.09	26.91	14.34	18.15	0.78	0.00	0.00
TMPRSS3	64699	1.09	0.86	0.26	0.1	2.32	0.00	0.00
TMTC1	83857	0.38	0.43	0.21	0.22	0.90	0.00	0.00
TMX1	81542	3.39	4.55	1.89	1.83	1.32	0.00	0.00
TMX3	54495	1.19	0.82	0.55	0.88	0.74	0.00	0.00
TMX4	56255	7.94	7.2	4.06	5.12	0.72	0.00	0.00
TNFAIP3	7128	17.38	17.58	14.17	7.68	0.67	0.00	0.00
TNFAIP8	25816	2.58	2.32	1.49	1.59	0.67	0.00	0.00
TNFRSF11A	8792	3.36	4.51	0.91	0.86	1.98	0.00	0.00
TNFRSF19	55504	25.47	25	12.68	10.86	1.11	0.00	0.00
TNFRSF21	27242	40.64	40.11	31.92	15.48	0.78	0.00	0.00
TNFSF10	8743	1.8	1.43	0.48	0.58	1.63	0.00	0.00
TNFSF14	8740	0.5	0.6	0.39	0.16	0.92	0.00	0.00
TNKS	8658	3.8	3.84	1.89	2.39	0.82	0.00	0.00
TNKS2	80351	3.36	3.74	1.62	2.7	0.71	0.00	0.00
TNNI1	7135	1.31	1.42	0.4	0.7	1.39	0.00	0.00
TNNT1	7138	1.03	1.19	1.21	0.43	1.04	0.00	0.00
TNRC18	84629	14.78	14.02	8.56	7.74	0.82	0.00	0.00
TNRC6A	27327	3.11	3.41	1.42	1.94	0.96	0.00	0.00
TOB1	10140	28.49	26.25	15.1	20.52	0.64	0.00	0.00
TOB2	10766	10.8	10.68	6.37	7.53	0.62	0.00	0.00
TOMM20L	387990	0.42	0.85	0.43	0	4.01	0.00	0.00
TOP1	7150	21.63	22.74	10.9	15.72	0.73	0.00	0.00
TOP2A	7153	9.91	9.95	4.46	6.97	0.80	0.00	0.00

TOP2B	7155	5.49	5.54	2.38	4.26	0.73	0.00	0.00
TOPORS	10210	1.21	1.36	0.39	0.55	1.43	0.00	0.00
TOX3	27324	6.24	6.78	1.83	1.23	1.97	0.00	0.00
TPH1	7166	0.05	0.08	0	0.01	3.67	0.00	0.00
TPMT	7172	8	8.79	3.32	6.06	0.93	0.00	0.00
TPR	7175	2.13	1.82	0.84	1.79	0.60	0.00	0.00
TPTE2P5	100616668	0.24	0.35	0.12	0.09	1.44	0.00	0.00
TPX2	22974	87.92	82.02	53.91	51.41	0.70	0.00	0.00
TRA2A	29896	13.24	12.71	6.6	7.91	0.87	0.00	0.00
TRAF2	7186	10.75	10.59	7.3	6.76	1.35	0.00	0.00
TRAF6	7189	1.67	1.74	1.09	1.24	0.65	0.00	0.00
TRAM2-AS1	401264	1.23	1.72	0.78	1.06	0.73	0.00	0.00
TRANK1	9881	0.1	0.11	0.02	0.05	1.53	0.00	0.00
TRAPPC2B	10597	5.2	5.59	3.2	3.47	0.69	0.00	0.00
TREH	11181	3.99	3.85	0.68	0.65	2.62	0.00	0.00
TRIL	9865	0.22	0.29	0.08	0.1	1.59	0.00	0.00
TRIM10	10107	3.38	3.39	1.14	1.61	1.33	0.00	0.00
TRIM13	10206	3.16	2.88	2.38	2.05	0.61	0.00	0.00
TRIM15	89870	23.21	25.12	15.51	14.6	0.71	0.00	0.00
TRIM2	23321	1.56	1.29	0.83	1.16	0.60	0.00	0.00
TRIM24	8805	19.36	19.4	8.05	15.26	0.74	0.00	0.00
TRIM56	81844	3.26	3.02	1.61	1.71	0.86	0.00	0.00
TRIM58	25893	0.14	0.21	0.09	0.08	1.13	0.00	0.00
TRIM59	286827	0.37	0.28	0.11	0	2.53	0.00	0.00
TRIP11	9321	0.69	0.41	0.31	0.35	0.87	0.00	0.00
TRIP12	9320	11.01	11.2	6.31	7.62	0.69	0.00	0.00
TRMO	51531	3.85	4.19	2.92	2.32	0.74	0.00	0.00
TRMT10A	93587	0.7	0.99	0.23	0.4	1.29	0.00	0.00
TRMT10C	54931	3.78	4.34	1.85	2.97	0.75	0.00	0.00
TRMT5	57570	2.49	3.45	1.51	2.06	0.99	0.00	0.00
TRNAU1AP	54952	1.33	3.33	0.02	0.49	2.81	0.00	0.00
TRPC7	57113	0.21	0	0	0	5.83	0.00	0.00
TRPM7	54822	1.22	1.33	0.46	1.06	0.72	0.00	0.00

TRPV4	59341	6	6.11	4.85	3.54	0.59	0.00	0.00
TSNAX	7257	3.37	4.73	0.46	0.57	3.08	0.00	0.00
TSNAX-DISC1	100303453	1.76	1.46	2.06	2.38	0.60	0.00	0.00
TSPAN8	7103	15.3	17.12	2.69	3.92	2.25	0.00	0.00
TSSK4	283629	0.89	0.73	0.4	0.32	0.70	0.00	0.00
TTC1	7265	21.58	22.31	13.1	14.75	0.66	0.00	0.00
TTC12	54970	3.68	3.81	1.9	2.26	0.89	0.00	0.00
TTC14	151613	2.66	2.78	1.23	2.37	0.60	0.00	0.00
TTC21B	79809	2.29	2.11	1.04	2.09	0.66	0.00	0.00
TTC22	55001	1.71	1.89	0.34	0.25	2.63	0.00	0.00
TTC27	55622	11.77	13.05	7.19	8.6	0.66	0.00	0.00
TTC30A	92104	0.83	0.92	0.44	0.31	1.22	0.00	0.00
TTC39C	125488	23.72	21.53	11.51	14.71	0.99	0.00	0.00
TTC7B	145567	1.07	2.25	1.09	0.52	1.01	0.00	0.00
TTLL10	254173	50.73	50.57	15.49	32.72	1.06	0.00	0.00
TTYH1	57348	6.36	5.65	2.93	1.24	1.53	0.00	0.00
TUBA3D	113457	0.37	0	0	0	5.00	0.00	0.00
TUBBP5	643224	3.97	3.59	0.54	1.02	2.30	0.00	0.00
TUG1	55000	6.19	6.55	2.78	4.88	0.72	0.00	0.00
TUSC3	7991	2.05	1.7	0.31	0.44	2.48	0.00	0.00
TUT4	23318	3.53	3.3	1.87	2.66	0.60	0.00	0.00
TUT7	79670	1.09	0.93	0.33	0.7	0.90	0.00	0.00
TXNRD3	114112	6.41	5.52	4.12	2.48	0.84	0.00	0.00
TYW1	55253	3.84	5.13	3.11	3.88	0.65	0.00	0.00
UBD	10537	125.87	123.82	13.19	2.42	3.96	0.00	0.00
UBE2Q2P2	100134869	74.74	65.61	34.33	36.83	0.98	0.00	0.00
UBE3A	7337	6.11	5.36	3.4	4.43	0.58	0.00	0.00
UBE3C	9690	20.09	21.02	13.82	13.62	0.60	0.00	0.00
UBE4A	9354	6.43	6.27	3.63	4.63	0.62	0.00	0.00
UBL7-AS1	440288	1.85	1.88	0.84	1.33	0.94	0.00	0.00
UBR1	197131	0.6	0.52	0.26	0.44	0.90	0.00	0.00
UBR2	23304	2.32	2.45	1.18	1.84	0.67	0.00	0.00
UBR3	130507	1.25	1.72	0.83	1.16	0.70	0.00	0.00

UBXN10	127733	1.3	1.36	0.55	0.38	1.51	0.00	0.00
UBXN2A	165324	7.28	6.47	3.36	3.85	0.82	0.00	0.00
UBXN2B	137886	1.91	1.68	1.17	1.39	0.64	0.00	0.00
UGCG	7357	1.9	1.79	1.2	0.97	1.03	0.00	0.00
UGDH-AS1	100885776	2.22	2.84	1.35	1.33	0.91	0.00	0.00
UGGT1	56886	22.39	23.24	12.62	13.74	0.81	0.00	0.00
UGT1A9	54600	2.6	2.38	0.9	1.52	1.04	0.00	0.00
UHMK1	127933	1.81	1.86	0.84	1.6	0.62	0.00	0.00
UNC119B	84747	12.27	12.54	7.55	8.31	0.65	0.00	0.00
UNC5C	8633	0	0	0	0	1.71	0.00	0.00
UNC93A	54346	20.01	18.45	7.22	8.63	1.28	0.00	0.00
UNKL	64718	4.76	4.4	3.26	1.93	0.72	0.00	0.00
UPF3B	65109	1.25	1.52	0.54	0.82	1.06	0.00	0.00
UPRT	139596	3.39	2.69	1.49	2.41	0.68	0.00	0.00
USP14	9097	13.83	13.53	9.03	9.18	0.62	0.00	0.00
USP16	10600	37.08	41.18	26.96	24.56	0.59	0.00	0.00
USP28	57646	3.28	3.23	2.04	2.06	0.68	0.00	0.00
USP33	23032	2.96	2.77	1.35	2.38	0.59	0.00	0.00
USP34	9736	3.05	3.6	1.68	2.81	0.62	0.00	0.00
USP40	55230	12.52	13.36	6.95	8.1	0.82	0.00	0.00
USP48	84196	12.7	14.36	7.68	10.43	0.67	0.00	0.00
USP49	25862	0.24	0.18	0.25	0.21	0.69	0.00	0.00
USP5	8078	37.36	38.55	37.69	41.43	0.65	0.00	0.00
USP53	54532	27.59	28.12	10.83	18.34	0.91	0.00	0.00
USP7	7874	13.05	13.86	7.85	8.55	0.72	0.00	0.00
USP8	9101	2.38	2.56	1	2.3	0.63	0.00	0.00
USPL1	10208	1.49	1.1	0.72	0.81	0.76	0.00	0.00
UST-AS1	100128176	0.09	0.08	0	0	4.73	0.00	0.00
UTP20	27340	2.19	2.36	1.05	1.66	0.73	0.00	0.00
UTP23	84294	1.19	0.82	0.56	0.55	0.85	0.00	0.00
UTP25	27042	2.23	2.63	1.66	1.06	0.76	0.00	0.00
VANGL1	81839	2.57	2.68	1.72	1.6	0.68	0.00	0.00
VCAN	1462	0.67	0.87	0.58	0.18	1.30	0.00	0.00

VCP	7415	131.07	129.62	109.11	121.61	0.90	0.00	0.00
VEZF1	7716	7.21	7.57	4.83	4.76	0.62	0.00	0.00
VIRMA	25962	3.5	3.93	1.94	2.74	0.66	0.00	0.00
VMO1	284013	2.05	1.38	1.72	2.41	0.83	0.00	0.00
VPS13B	157680	2.78	0.92	0.69	0.89	0.63	0.00	0.00
VPS13D	55187	2.56	2.57	1.58	1.5	0.72	0.00	0.00
VPS35	55737	35.77	37.47	20.18	24.86	0.71	0.00	0.00
VPS36	51028	3.2	2.78	1.35	1.79	0.97	0.00	0.00
VPS4B	9525	4.55	3.64	2.47	3.15	0.59	0.00	0.00
VPS8	23355	3.01	2.55	1.67	1.8	0.74	0.00	0.00
VRK1	7443	5.24	4.74	2.78	2.86	0.84	0.00	0.00
VTA1	51534	10.85	10.68	4.99	6.75	0.88	0.00	0.00
VWA1	64856	88.65	87.29	58.54	49.85	0.70	0.00	0.00
WAC-AS1	220906	1.77	1.6	0.73	1.22	0.79	0.00	0.00
WAPL	23063	3.29	3.25	1.69	2.24	0.73	0.00	0.00
WASF3	10810	3.36	3.21	1.92	2.11	0.70	0.00	0.00
WASHC2C	253725	4.26	4.28	2.06	3.31	0.73	0.00	0.00
WDCP	80304	5.37	6.02	3.07	3.53	0.77	0.00	0.00
WDHD1	11169	0.74	1.02	0.28	0.62	0.98	0.00	0.00
WDR31	114987	0.72	0.69	0.47	0.25	0.99	0.00	0.00
WDR44	54521	0.83	0.79	0.53	0.49	0.85	0.00	0.00
WDR48	57599	4.74	4.45	2.85	2.62	0.73	0.00	0.00
WDR75	84128	8.06	8.08	3.87	5.46	0.79	0.00	0.00
WNK1	65125	11.74	11.73	7.5	7.58	0.64	0.00	0.00
WRN	7486	1.04	1.1	0.67	0.8	0.67	0.00	0.00
WWC2	80014	2.55	2.73	1.38	1.8	0.72	0.00	0.00
WWC2-AS1	101928734	0.29	0	0	0.1	1.47	0.00	0.00
WWC3	55841	1.76	1.32	2.56	2.46	0.92	0.00	0.00
XAF1	54739	0.16	0.15	0.05	0.03	2.50	0.00	0.00
XPNPEP2	7512	4.76	5.31	0.48	1.3	2.50	0.00	0.00
XPO1	7514	22.19	22.29	10.9	16.38	0.71	0.00	0.00
XRCC2	7516	1.05	0.99	0.57	0.77	0.74	0.00	0.00
XXYLT1	152002	17.69	16.57	12.3	10.78	0.58	0.00	0.00

YAP1	10413	17.76	18.47	9.69	11.25	0.80	0.00	0.00
YEATS4	8089	10	10.62	5.68	7.17	0.70	0.00	0.00
YIPF3	25844	111.53	109.23	134.06	127.33	0.70	0.00	0.00
YIPF5	81555	11.97	13.48	6.04	7.91	0.61	0.00	0.00
YPEL2	388403	1.27	1.18	0.4	0.46	1.55	0.00	0.00
YTHDC2	64848	1.65	1.78	1	1.12	0.69	0.00	0.00
YY1	7528	19.02	19.98	11.42	13.88	0.60	0.00	0.00
ZACN	353174	0	0	0.04	0	4.64	0.00	0.00
ZBED5-AS1	729013	1.95	2.04	0.84	1.33	1.01	0.00	0.00
ZBED8	63920	0.71	0.69	0.19	0.17	1.97	0.00	0.00
ZBTB10	65986	0.86	0.83	0.43	0.63	0.73	0.00	0.00
ZBTB34	403341	0.83	0.87	0.48	0.49	0.92	0.00	0.00
ZBTB37	84614	0.35	0.21	0.19	0.17	0.74	0.00	0.00
ZBTB40	9923	5.18	4.81	3.12	3.35	0.60	0.00	0.00
ZBTB44	29068	1.82	2.58	1.41	1.86	0.74	0.00	0.00
ZBTB8A	653121	0.46	0.32	0.19	0.21	0.99	0.00	0.00
ZC3H12A	80149	18.59	17.79	12.35	7.04	0.87	0.00	0.00
ZC3H13	23091	2.06	1.93	0.95	1.55	0.63	0.00	0.00
ZC3H7A	29066	10.12	9.9	4.76	6.51	0.83	0.00	0.00
ZCCHC10	54819	3.93	3.24	2.15	2.2	0.73	0.00	0.00
ZCCHC14	23174	8.54	8.64	5.04	5.82	0.76	0.00	0.00
ZCCHC2	54877	7.63	7.05	3.12	5.08	0.82	0.00	0.00
ZCCHC4	29063	2.57	2.91	1.66	2.02	0.58	0.00	0.00
ZCCHC8	55596	4.72	4.85	2.47	3.16	0.73	0.00	0.00
ZDBF2	57683	0.14	0.06	0	0.03	2.24	0.00	0.00
ZDHHC2	51201	5.94	5.59	2.29	2.31	1.37	0.00	0.00
ZDHHC21	340481	0.55	0.5	0.43	0.42	0.86	0.00	0.00
ZDHHC23	254887	2.75	3.11	1.48	1.67	0.96	0.00	0.00
ZDHHC6	64429	9.92	8.46	4.78	6.51	0.75	0.00	0.00
ZFAND1	79752	4.68	3.91	3.04	2.99	0.61	0.00	0.00
ZFHX3	463	2.55	2.25	1.2	1.37	0.94	0.00	0.00
ZFP30	22835	0.47	0.62	0.18	0.27	1.18	0.00	0.00
ZFR	51663	10.45	9.32	5.29	7.17	0.67	0.00	0.00

ZFY	7544	0.8	0.55	0.19	0.44	1.03	0.00	0.00
ZG16B	124220	6.77	7.73	4.12	2.91	1.08	0.00	0.00
ZIM2	23619	3.11	4.25	1.44	1.59	1.43	0.00	0.00
ZKSCAN1	7586	5.14	5.24	2.46	3.04	0.98	0.00	0.00
ZKSCAN2	342357	2.46	2.39	1.29	1.72	0.71	0.00	0.00
ZKSCAN3	80317	0.93	0.99	0.61	0.35	0.83	0.00	0.00
ZKSCAN4	387032	2.02	2.17	1.12	1.01	0.88	0.00	0.00
ZKSCAN8	7745	3.54	2.88	1.6	1.73	0.97	0.00	0.00
ZNF101	94039	4.5	4.71	2.63	2.56	0.63	0.00	0.00
ZNF106	64397	3.92	3.31	2.17	2.39	0.69	0.00	0.00
ZNF12	7559	1.04	0.89	0.38	0.61	1.26	0.00	0.00
ZNF131	7690	2.19	1.91	1.33	1.52	0.60	0.00	0.00
ZNF134	7693	1.12	0.82	0.7	0.55	0.64	0.00	0.00
ZNF140	7699	2.02	2.11	1.29	1.31	0.68	0.00	0.00
ZNF146	7705	3.29	3.04	1.71	2.63	0.60	0.00	0.00
ZNF155	7711	0.35	0.61	0.16	0.23	1.70	0.00	0.00
ZNF169	169841	0.6	0.63	0.49	0.17	1.04	0.00	0.00
ZNF174	7727	7.78	7.67	6.17	4.58	0.59	0.00	0.00
ZNF192P1	651302	0.18	0.14	0.09	0.08	1.71	0.00	0.00
ZNF197	10168	0.07	0.24	0	0.04	2.25	0.00	0.00
ZNF211	10520	1.61	1.65	1.03	0.98	0.82	0.00	0.00
ZNF217	7764	9.72	9.38	5.85	6.47	0.63	0.00	0.00
ZNF22	7570	0.34	0.27	0	0.01	5.73	0.00	0.00
ZNF223	7766	0.43	0.27	0.07	0.09	1.88	0.00	0.00
ZNF224	7767	0.2	0.38	0.08	0.12	1.68	0.00	0.00
ZNF225	7768	0.31	0.29	0.11	0.04	2.11	0.00	0.00
ZNF227	7770	0.54	0.6	0.22	0.24	0.95	0.00	0.00
ZNF230	7773	0.18	0.24	0	0.02	4.24	0.00	0.00
ZNF236	7776	0.67	0.7	0.34	0.62	0.61	0.00	0.00
ZNF24	7572	3	3.04	1.79	1.79	0.80	0.00	0.00
ZNF252P	286101	0.95	0.96	0.45	0.42	1.12	0.00	0.00
ZNF264	9422	1.26	1.3	0.75	0.81	0.96	0.00	0.00
ZNF280D	54816	1.08	1.42	0.67	0.7	0.87	0.00	0.00

ZNF283	284349	0.26	0.26	0.14	0.07	1.26	0.00	0.00
ZNF286B	729288	0.66	0.9	0.49	0.5	0.97	0.00	0.00
ZNF304	57343	1.25	1.29	1.03	0.64	0.64	0.00	0.00
ZNF322	79692	1.56	1.76	0.46	0.7	1.52	0.00	0.00
ZNF326	284695	2.4	1.73	0.58	1.79	0.73	0.00	0.00
ZNF337	26152	3.13	3.87	2.86	1.8	0.72	0.00	0.00
ZNF33B	7582	2.73	2.18	0.98	0.88	1.32	0.00	0.00
ZNF343	79175	4.47	4.21	2.51	1.92	0.96	0.00	0.00
ZNF382	84911	0.13	0.05	0.04	0	2.59	0.00	0.00
ZNF385B	151126	15.49	14.11	3.54	3.55	2.05	0.00	0.00
ZNF395	55893	8.84	8.28	4.53	3.53	1.09	0.00	0.00
ZNF397	84307	1.6	1.77	1.17	0.52	0.89	0.00	0.00
ZNF41	7592	0.57	0.7	0.36	0.43	0.75	0.00	0.00
ZNF416	55659	1.44	1.87	0.95	1.03	0.73	0.00	0.00
ZNF417	147687	0.44	0.35	0.12	0.19	1.36	0.00	0.00
ZNF443	10224	1.02	0.74	0.42	0.47	0.92	0.00	0.00
ZNF45	7596	0.84	0.81	0.29	0.33	1.46	0.00	0.00
ZNF473	25888	4.02	3.4	2.69	1.99	0.64	0.00	0.00
ZNF507	22847	0.87	0.8	0.41	0.6	0.72	0.00	0.00
ZNF517	340385	3.2	3.21	1.97	1.73	0.98	0.00	0.00
ZNF530	348327	0.59	0.47	0.18	0.38	1.06	0.00	0.00
ZNF543	125919	1.01	1	0.63	0.48	0.85	0.00	0.00
ZNF544	27300	1.95	1.67	1.83	1.19	0.75	0.00	0.00
ZNF551	90233	0.84	0.82	0.29	0.28	1.54	0.00	0.00
ZNF556	80032	6.36	7.41	3.01	3.56	0.94	0.00	0.00
ZNF558	148156	1.16	1	0.4	0.39	1.32	0.00	0.00
ZNF572	137209	0.6	0.44	0.19	0.25	1.25	0.00	0.00
ZNF587	84914	0.49	0.39	0.06	0.18	1.90	0.00	0.00
ZNF587B	100293516	4.2	4.68	2.2	2.08	0.82	0.00	0.00
ZNF606	80095	0.5	0.43	0.15	0.24	1.09	0.00	0.00
ZNF619	285267	0.79	0.74	0.48	0.37	1.03	0.00	0.00
ZNF620	253639	1.43	1.48	0.86	0.91	0.94	0.00	0.00
ZNF621	285268	1.42	1.19	0.94	0.95	0.71	0.00	0.00

ZNF629	23361	13.45	13.51	8.05	7.75	0.77	0.00	0.00
ZNF638	27332	1.28	1.33	0.63	1.1	0.60	0.00	0.00
ZNF644	84146	2	2.37	0.91	1.82	0.62	0.00	0.00
ZNF652	22834	4.13	4.43	2.6	2.94	0.61	0.00	0.00
ZNF664	144348	18.85	18.45	7.84	10.89	0.99	0.00	0.00
ZNF691	51058	4.62	4.86	3.63	2.37	0.77	0.00	0.00
ZNF70	7621	0.6	0.79	0.33	0.35	1.22	0.00	0.00
ZNF71	58491	2.67	2.64	1.8	1.37	0.81	0.00	0.00
ZNF749	388567	0.95	1.23	0.32	0.66	1.13	0.00	0.00
ZNF75A	7627	0.32	0.46	0.08	0.13	1.71	0.00	0.00
ZNF765	91661	0.15	0.2	0.02	0	3.84	0.00	0.00
ZNF765-ZNF761	110116772	1.95	2.24	1.21	1.5	0.61	0.00	0.00
ZNF766	90321	1.29	1.28	0.6	0.77	0.92	0.00	0.00
ZNF772	400720	0.43	0.65	0.23	0.32	0.96	0.00	0.00
ZNF776	284309	1.12	1.2	0.54	0.69	0.65	0.00	0.00
ZNF786	136051	1.6	1.41	1.31	1.57	0.60	0.00	0.00
ZNF850	342892	0.15	0.1	0.04	0.06	1.81	0.00	0.00
ZNF852	285346	1.08	1.24	0.76	0.7	0.61	0.00	0.00
ZNRF2	223082	0.9	0.75	0.5	0.42	0.66	0.00	0.00
ZP3	7784	0.24	0.22	0.06	0.09	2.01	0.00	0.00
ZRANB3	84083	1.44	1.37	1.33	1.12	0.63	0.00	0.00
ZSCAN21	7589	3.41	3.22	2.31	1.31	0.87	0.00	0.00
ZSCAN22	342945	0.2	0.74	0.09	0.13	2.02	0.00	0.00
ZSCAN26	7741	1.4	1.67	0.63	0.65	1.36	0.00	0.00
ZSCAN30	100101467	0.52	0.8	0.55	0.34	0.71	0.00	0.00
ZSCAN31	64288	1.14	1.42	0.63	0.84	0.78	0.00	0.00
ZSWIM9	374920	3.24	2.64	2.43	1.48	0.66	0.00	0.00
ZW10	9183	6.35	5.81	2.46	3.9	0.93	0.00	0.00
ZYG11B	79699	1.47	1.56	0.91	1.22	0.85	0.00	0.00
ZZZ3	26009	1.69	2.2	0.95	1.57	0.77	0.00	0.00

Table S1d. Genes down-regulated (>1.5-fold change, q-value < 0.001) after miR-27a knock out

Symobol	GeneID	27aKO1 FPKM	27aKO2 FPKM	Scr1 FPKM	Scr2 FPKM	log2Ratio 23aKO/Scr	q-value	p-value
A1BG	1	5.59	5.23	9.78	14.17	-1.09	0.00	0.00
A4GALT	53947	0.11	0.19	0.96	0.14	-1.99	0.00	0.00
AARS	16	74.57	76.01	141.36	87.55	-0.58	0.00	0.00
ABCA17P	650655	0	0.02	0.1	0.05	-2.60	0.00	0.00
ABCA3	21	7.08	7.13	12.62	10.87	-0.72	0.00	0.00
ABCA4	24	0.27	0.26	0.96	0.48	-1.45	0.00	0.00
ABCC11	85320	3.58	4.01	5.5	6.94	-0.61	0.00	0.00
ABHD2	11057	3.91	3.75	5.74	7.23	-0.77	0.00	0.00
ACKR2	1238	0.25	0.5	1.29	0.88	-1.58	0.00	0.00
ACOX2	8309	11.61	10.62	15.97	31.04	-0.99	0.00	0.00
ACRBP	84519	0.03	0.03	0.36	0.3	-2.22	0.00	0.00
ADAM11	4185	1.48	1.69	2.85	4.2	-1.18	0.00	0.00
ADAM19	8728	0.01	0.03	0.47	0.33	-4.56	0.00	0.00
ADAMTS4	9507	3.69	3.91	6.49	4.88	-0.60	0.00	0.00
ADAMTS9	56999	0	0	0.31	0.28	-7.93	0.00	0.00
ADAT3	113179	4.79	4.53	6.61	7.62	-0.60	0.00	0.00
ADGRL2	23266	0.84	0.66	0.82	1.68	-0.72	0.00	0.00
ADM2	79924	2.5	2.22	4.87	3.05	-0.76	0.00	0.00
ADORA2A	135	3.66	3.44	6.31	5.11	-0.69	0.00	0.00
ADORA2B	136	2.93	3.46	10.39	10.82	-1.73	0.00	0.00
AFP	174	82.88	86.24	1030.99	1594.7	-3.96	0.00	0.00
AGAP2	116986	1.7	1.71	2.91	3.03	-0.67	0.00	0.00
AGAP2-AS1	100130776	0.24	0.06	1.07	0.33	-2.26	0.00	0.00
AGR2	10551	0.75	1.07	3.91	9.71	-2.88	0.00	0.00
AGT	183	453.16	446.99	767.21	869.21	-0.86	0.00	0.00
AGTR1	185	0.19	0.23	1.03	1.01	-2.26	0.00	0.00
AJM1	389813	0.92	0.29	0.33	2.11	-1.03	0.00	0.00
AKR1B1	231	82.47	88.13	175.49	132.39	-0.85	0.00	0.00
AKR1C2	1646	1.56	1.07	35.63	18.92	-2.22	0.00	0.00

ALB	213	812.27	838.14	3594.91	5334.22	-2.44	0.00	0.00
ALDOC	230	14.09	14.16	35.63	25.02	-1.10	0.00	0.00
ALG14	199857	2.64	3.45	4.13	7.19	-0.79	0.00	0.00
ALPL	249	0.15	0.13	6.58	2.13	-5.06	0.00	0.00
AMIGO3	386724	0	0	0.82	0.22	-6.84	0.00	0.00
AMPD3	272	0.21	0.23	1.24	0.91	-1.49	0.00	0.00
ANGPTL4	51129	0.84	0.66	2.65	0.93	-1.27	0.00	0.00
ANGPTL6	83854	0.36	0.79	1.22	0.77	-0.99	0.00	0.00
ANGPTL8	55908	154.74	153.56	273.45	270.13	-0.82	0.00	0.00
ANK2	287	0.86	0.95	1.86	2.31	-1.20	0.00	0.00
ANKRD1	27063	7.68	7.35	8.05	16.36	-0.70	0.00	0.00
ANKRD13B	124930	3.51	3	7.47	4.99	-0.92	0.00	0.00
ANKRD35	148741	0.05	0.05	0.2	0.3	-2.25	0.00	0.00
ANKRD9	122416	1.28	1.62	1.92	2.54	-0.64	0.00	0.00
ANTXR2	118429	0.32	0.34	1.43	2.44	-2.77	0.00	0.00
ANXA6	309	34.22	34.97	83.82	102.94	-1.39	0.00	0.00
AOC2	314	0	0	0.62	0	-5.91	0.00	0.00
AP1M1	8907	38.23	38.46	58.86	69.51	-0.73	0.00	0.00
APOA4	337	6.78	6.44	22.07	32.52	-2.05	0.00	0.00
AQP6	363	0.09	0.06	0.29	0.46	-2.02	0.00	0.00
AQP7	364	3.17	2.73	4.2	5.06	-0.67	0.00	0.00
AQP8	343	1.76	1.19	5.19	8.8	-2.13	0.00	0.00
ARAP3	64411	0.06	0.05	0.24	0.08	-1.62	0.00	0.00
AREG	374	1.26	1.09	5.61	1.32	-1.54	0.00	0.00
ARHGAP10	79658	0.04	0.03	0.6	0.27	-3.67	0.00	0.00
ARHGAP23	57636	0	0	0.09	0.13	-5.46	0.00	0.00
ARHGAP40	343578	0	0.02	1.08	0.59	-0.68	0.00	0.00
ARHGDIB	397	0.28	0.52	2.36	5.67	-3.38	0.00	0.00
ARID2	196528	0.53	0.57	0.66	0.88	-0.60	0.00	0.00
ARID4B	51742	0.24	0.24	0.25	0.44	-0.62	0.00	0.00
ARL17B	100506084	3.01	3.56	7.84	5.51	-0.86	0.00	0.00
ARL5B	221079	1.76	1.55	1.82	3.76	-0.76	0.00	0.00
ARL8A	127829	9.23	8.85	15.85	12.95	-0.74	0.00	0.00

ARMC6	93436	27.36	27.16	39.82	40.87	-0.59	0.00	0.00
ARRB1	408	13.41	13.41	20.61	17.68	-0.60	0.00	0.00
ARRDC4	91947	0.93	0.86	2.22	2.03	-1.26	0.00	0.00
ARSG	22901	3.25	3.23	8.26	4.62	-0.81	0.00	0.00
ASAP3	55616	11.71	12.14	23.31	17.69	-0.66	0.00	0.00
ASB13	79754	12.06	11.36	20.39	18.38	-0.72	0.00	0.00
ASB4	51666	0.04	0.04	3.77	2.51	-7.07	0.00	0.00
ASB9	140462	4.29	4.1	5.68	8.04	-0.70	0.00	0.00
ASCL5	647219	0	0	1.35	0.69	-6.67	0.00	0.00
ASNA1	439	46.11	46.42	85.23	85.45	-0.88	0.00	0.00
ASPH	444	20.07	19.73	33.14	44.92	-0.85	0.00	0.00
ATG4D	84971	13.4	12.3	21.14	21.06	-0.75	0.00	0.00
ATP2B2	491	0.01	0	0.16	0.44	-6.29	0.00	0.00
ATP6V0D1	9114	36.43	36.34	61.64	85.72	-0.97	0.00	0.00
ATP6V1B1	525	0.26	0.12	1.07	1.01	-2.55	0.00	0.00
ATP8A1	10396	0.02	0.04	0.22	0.4	-3.20	0.00	0.00
ATP8B2	57198	3.35	3.2	11.56	8.01	-1.54	0.00	0.00
AXL	558	0.7	0.91	4.06	1.94	-1.88	0.00	0.00
B3GNT8	374907	0.47	0.32	1.48	1.23	-1.67	0.00	0.00
B3GNT9	84752	2.48	2.67	6.32	5.57	-1.21	0.00	0.00
B3GNTL1	146712	5.43	3.81	9.5	9.18	-0.88	0.00	0.00
BAG3	9531	14.23	15.79	23.72	22.77	-0.60	0.00	0.00
BAHCC1	57597	0.78	0.86	3.46	2.89	-1.80	0.00	0.00
BAMBI	25805	38.47	43.15	102.82	64.61	-1.04	0.00	0.00
BBS5	129880	0.88	0.87	2.29	3.19	-1.68	0.00	0.00
BCAT1	586	0.01	0.05	2.47	2.93	-6.73	0.00	0.00
BCL6B	255877	0	0.02	0	0	-0.82	0.00	0.00
BEAN1	146227	0.04	0.04	1.33	0.67	-4.70	0.00	0.00
BEND7	222389	2.49	2.61	6.16	6.02	-0.99	0.00	0.00
BEST1	7439	1.93	3.17	5.08	3.21	-0.62	0.00	0.00
BFSP1	631	0.21	0.11	2.81	1.45	-3.59	0.00	0.00
BGN	633	0.1	0.1	2.46	0.68	-4.08	0.00	0.00
BIN1	274	0.7	0.7	1.5	1.37	-1.16	0.00	0.00

BIRC7	79444	0.32	0.58	5.19	2.38	-3.05	0.00	0.00
BIVM	54841	0.14	0.54	0.83	0.91	-1.36	0.00	0.00
BMP4	652	73.2	76.76	176.94	150.27	-1.13	0.00	0.00
BMPR1B	658	0.01	0.01	0	0.01	-1.29	0.00	0.00
BOC	91653	0.27	0.19	3.95	3.87	-3.97	0.00	0.00
BOLA2B	654483	22.66	31.48	54.43	41.59	-0.78	0.00	0.00
BORCS8-MEF2B	4207	1.76	1.49	4.2	2.72	-0.76	0.00	0.00
BRAT1	221927	14.93	14.13	13.79	12.66	-0.81	0.00	0.00
BRSK1	84446	0.1	0.11	1.37	0.62	-2.50	0.00	0.00
BRSK2	9024	0.81	0.57	1.93	1.38	-1.24	0.00	0.00
BSG	682	521.63	507.82	889.73	705.44	-0.63	0.00	0.00
BTBD16	118663	7.27	8.24	13.75	11.25	-0.77	0.00	0.00
C1QTNF6	114904	3.75	2.68	6.02	6.89	-0.92	0.00	0.00
C2CD2L	9854	2.15	1.98	4.43	3.73	-0.86	0.00	0.00
C8G	733	15.4	11.61	25.58	28.57	-0.99	0.00	0.00
CA14	23632	0.93	1.24	2.8	1.2	-0.95	0.00	0.00
CA5BP1	340591	4.13	4.07	6.09	8.83	-0.68	0.00	0.00
CACNA2D4	93589	5.42	5.33	36.09	29.32	-2.57	0.00	0.00
CACNG4	27092	17.2	16.78	21.73	30.18	-0.61	0.00	0.00
CADM1	23705	0.01	0.06	0.99	0.53	-4.35	0.00	0.00
CADM4	199731	17.8	18.87	31.47	23.81	-0.64	0.00	0.00
CALHM2	51063	0.11	0	0.47	0.17	-2.42	0.00	0.00
CAPN3	825	1.41	1.08	1.65	3.89	-1.10	0.00	0.00
CAPN5	726	0.09	0.28	1.42	0.99	-2.61	0.00	0.00
CAPN9	10753	0.09	0.18	0.48	0.51	-2.18	0.00	0.00
CAPRIN2	65981	1.8	2.25	4.02	4.2	-1.01	0.00	0.00
CAPZA2	830	12.29	12.02	5.92	8.91	-6.99	0.00	0.00
CASC10	399726	2.41	3.21	4.52	4.97	-0.81	0.00	0.00
CASP16P	197350	0.14	0.19	0.53	0.71	-1.91	0.00	0.00
CASZ1	54897	0.21	0.29	2.27	2.34	-2.95	0.00	0.00
CAVIN1	284119	3.17	2.77	8.26	10.08	-1.63	0.00	0.00
CBR1	873	1.39	1.33	15.83	14.85	-3.49	0.00	0.00
CCDC102A	92922	0.68	0.79	1.61	1.4	-1.05	0.00	0.00

CCDC113	29070	0.01	0.07	0.71	0.04	-1.91	0.00	0.00
CCDC187	399693	0.13	0.19	0.44	0.34	-1.32	0.00	0.00
CCDC74A	90557	0.08	0.17	2.09	0.56	-3.31	0.00	0.00
CCDC74B	91409	0.38	0.22	2.03	1.3	-2.31	0.00	0.00
CCNB1IP1	57820	31.86	28.46	58.24	35.93	-0.65	0.00	0.00
CCND2	894	0.44	0.29	1.02	1	-1.47	0.00	0.00
CCND3	896	9.41	8.77	14.25	15.02	-0.65	0.00	0.00
CCNE1	898	20.54	23.49	40.7	33.16	-0.72	0.00	0.00
CCNO	10309	0	0.04	1.02	1.25	-6.05	0.00	0.00
CCPG1	9236	0.26	0.51	0.66	1.3	-1.42	0.00	0.00
CD109	135228	0.66	0.64	0.71	1.14	-0.63	0.00	0.00
CD36	948	1.27	0.82	2.15	2.6	-1.41	0.00	0.00
CD3D	915	0.63	0.21	4.57	1.1	-2.96	0.00	0.00
CD3G	917	0.01	0	0.08	0.01	-3.72	0.00	0.00
CD4	920	0.2	0.12	0.96	0.51	-1.68	0.00	0.00
CD55	1604	6.25	5.98	13.88	10.35	-0.92	0.00	0.00
CD70	970	0.12	0.14	1.1	0.54	-1.87	0.00	0.00
CD9	928	2.57	1.91	8.4	6.19	-1.70	0.00	0.00
CDC34	997	81.71	76.8	147.01	140.56	-0.86	0.00	0.00
CDCP1	64866	0.01	0	0.14	0.07	-4.55	0.00	0.00
CDH16	1014	1.21	1.19	9.16	7.3	-2.78	0.00	0.00
CDH2	1000	0.96	0.95	2.98	2.25	-1.42	0.00	0.00
CDK14	5218	0.06	0	0.54	0.45	-4.69	0.00	0.00
CDKL1	8814	0.03	0	0.44	0.27	-4.47	0.00	0.00
CDKN1A	1026	58.21	56.1	143.83	123.5	-1.23	0.00	0.00
CDKN2A	1029	8.81	7.08	13.06	10.68	-0.75	0.00	0.00
CDKN2B	1030	0.03	0.01	0.38	0.38	-4.19	0.00	0.00
CDR2L	30850	4.83	4.29	9.73	7.38	-0.91	0.00	0.00
CDX2	1045	0.05	0.1	1.23	1.24	-4.15	0.00	0.00
CEBPB	1051	28.44	28.42	75.64	39.03	-1.01	0.00	0.00
CELF3	11189	0.04	0.01	0.08	0.06	-1.88	0.00	0.00
CELSR3	1951	1.99	2.16	4.42	2.79	-0.70	0.00	0.00
CERNA1	100129973	0.11	0.02	0.02	0.17	-1.53	0.00	0.00

CETP	1071	0.38	0.55	7.07	1.79	-3.23	0.00	0.00
CFAP58	159686	0.03	0.06	0.08	0.08	-1.62	0.00	0.00
CHAC1	79094	2.34	1.96	7.29	1.54	-1.05	0.00	0.00
CHCHD10	400916	90.41	81.92	203.42	155.39	-1.06	0.00	0.00
CHD3	1107	1.1	0.91	3.65	2.69	-1.66	0.00	0.00
CHI3L1	1116	0.25	0.19	2.01	1.44	-2.91	0.00	0.00
CHRND	1144	0.8	0.65	1.85	0.99	-0.96	0.00	0.00
CHST15	51363	4.26	4.07	13.79	5.29	-1.26	0.00	0.00
CHSY3	337876	0	0	0.34	0.16	-2.51	0.00	0.00
CIDEC	63924	15.91	13.88	22.4	31.41	-0.85	0.00	0.00
CILP2	148113	0.1	0.08	0.28	0.19	-1.61	0.00	0.00
CKB	1152	82.03	77.53	133.38	108.35	-0.60	0.00	0.00
CKLF	51192	23.25	22.82	49.5	41.98	-0.99	0.00	0.00
CKM	1158	1.74	2.23	5.93	5.34	-1.42	0.00	0.00
CKMT1B	1159	0	0.07	0.35	0.35	-3.44	0.00	0.00
CKMT2	1160	0.62	0.25	7.38	8.28	-2.50	0.00	0.00
CLCF1	23529	0.73	0.77	1.75	1.06	-0.97	0.00	0.00
CLCN4	1183	0.92	0.98	1.39	1.51	-0.60	0.00	0.00
CLDN2	9075	2.14	1.72	1.43	6.24	-0.97	0.00	0.00
CLEC18A	348174	3.1	2.62	6.64	5.02	-0.93	0.00	0.00
CLEC18B	497190	1.22	1.1	2.7	1.51	-0.65	0.00	0.00
CLEC18C	283971	1.39	1.65	3.73	2.71	-0.98	0.00	0.00
CLMAT3	101927096	0.36	0.46	2.39	2.1	-2.53	0.00	0.00
CLTB	1212	33.1	34.23	55.33	49	-0.59	0.00	0.00
CLTRN	57393	0.76	0.35	0.9	1.43	-1.15	0.00	0.00
CLU	1191	24.09	35.12	47.34	62.36	-0.87	0.00	0.00
CMTM3	123920	18.13	19.47	37.77	22.72	-0.69	0.00	0.00
COL2A1	1280	0.09	0.05	0.57	0.4	-2.82	0.00	0.00
COL4A2-AS2	100129836	0.19	0.12	1.84	0.73	-3.07	0.00	0.00
COL5A2	1290	0.07	0.06	0.36	0.18	-1.70	0.00	0.00
COL6A1	1291	0.38	0.28	1.7	1.15	-2.11	0.00	0.00
COLGALT2	23127	0.05	0.03	17.86	12.68	-8.64	0.00	0.00
CORO2B	10391	0.4	0.39	0.64	0.75	-1.31	0.00	0.00

CORO6	84940	0.95	0.39	1.83	0.96	-1.16	0.00	0.00
COTL1	23406	7.53	6.31	59.48	49.81	-2.98	0.00	0.00
COX20	116228	9.37	7.25	10.17	13.09	-0.90	0.00	0.00
CPA2	1358	0	0	0.13	0	-1.45	0.00	0.00
CPA5	93979	0.15	0	0.72	0.64	-3.23	0.00	0.00
CPB2-AS1	100509894	0.06	0.04	0.18	0.11	-1.91	0.00	0.00
CPEB2-DT	441009	0	0	0	0	-3.58	0.00	0.00
CPED1	79974	1.05	0.98	5.11	5.96	-2.40	0.00	0.00
CPLX1	10815	0.72	1.29	5.39	3.59	-2.23	0.00	0.00
CPNE7	27132	0.09	0.05	0.31	0.18	-1.97	0.00	0.00
CPZ	8532	0.13	0.27	1.34	0.69	-2.47	0.00	0.00
CREB3L3	84699	8.53	8.18	17.3	22.03	-1.23	0.00	0.00
CRIP3	401262	2.89	3.3	14.76	13.55	-2.10	0.00	0.00
CRYAB	1410	1.91	2.04	18.05	13.54	-3.06	0.00	0.00
CRYBB3	1417	0.63	0.72	1.41	1.41	-1.06	0.00	0.00
CSDC2	27254	0.02	0.04	0.65	0.24	-3.79	0.00	0.00
CSF1	1435	0.54	0.76	3.13	1.18	-1.75	0.00	0.00
CSF1R	1436	0	0	0.04	0.02	-4.88	0.00	0.00
CSNK2A3	283106	0	0	0.02	0	-0.70	0.00	0.00
CTBP1-DT	92070	5.18	3.74	3.45	4.09	-0.60	0.00	0.00
CTCF	10664	3.68	3.74	11.49	4.87	-1.00	0.00	0.00
CTDSPL	10217	0.02	0.01	3.83	3.87	-8.25	0.00	0.00
CTGF	1490	60.93	62.16	108.85	119.9	-0.89	0.00	0.00
CTH	1491	4.99	4.34	9	6.35	-0.62	0.00	0.00
CTRL	1506	0.44	0	0.86	0.81	-1.93	0.00	0.00
CYP17A1	1586	0.19	0.21	0.35	0.83	-1.55	0.00	0.00
CYP1A1	1543	0.47	0.47	6.65	1.5	-3.12	0.00	0.00
CYP21A2	1589	0.71	0.73	2.43	1.14	-1.27	0.00	0.00
CYP24A1	1591	0.8	0.96	18.31	14.33	-4.22	0.00	0.00
CYP27B1	1594	0.58	0.57	1.32	1.33	-0.72	0.00	0.00
CYP2B6	1555	0.02	0.05	7.85	4.27	-7.41	0.00	0.00
CYP2U1	113612	0.09	0	0.27	0.11	-4.93	0.00	0.00
CYP39A1	51302	0	0	0.99	1.17	-7.56	0.00	0.00

CYP3A5	1577	0.94	1.08	2	4.54	-1.73	0.00	0.00
CYP3A7-CYP3A51P	100861540	0	0.06	0.19	0.27	-2.89	0.00	0.00
CYTH1	9267	10.29	10.19	16.52	14.44	-0.59	0.00	0.00
DAB2	1601	0.02	0	0.22	0.68	-6.25	0.00	0.00
DAD1	1603	192.84	182.94	176.41	192.88	-1.07	0.00	0.00
DCAF15	90379	13.21	12.56	22.98	19.79	-0.68	0.00	0.00
DCDC1	341019	0.01	0.02	0.25	0.28	-4.08	0.00	0.00
DDA1	79016	12.7	12.15	24.35	21.63	-0.89	0.00	0.00
DDAH2	23564	4.41	5.87	18.21	14.2	-1.57	0.00	0.00
DDIT3	1649	9.43	7.96	18.61	11.04	-0.74	0.00	0.00
DDN	23109	0.28	0.35	0.29	1.61	-1.59	0.00	0.00
DDOST	1650	4.85	11.59	20.66	9.84	-0.89	0.00	0.00
DDR2	4921	0.01	0.01	0.46	0.37	-5.10	0.00	0.00
DDX28	55794	6.5	6.83	10.96	11.02	-0.72	0.00	0.00
DDX49	54555	26.25	25.1	37.17	41.18	-0.62	0.00	0.00
DEPP1	11067	8.96	7.45	23.06	8.11	-0.92	0.00	0.00
DGKH	160851	0	0	0.04	0.04	-4.03	0.00	0.00
DHPS	1725	35.46	34.07	61.5	47.11	-0.61	0.00	0.00
DIP2C	22982	0.01	0.01	0.09	0.2	-4.39	0.00	0.00
DIRAS1	148252	1.04	1.21	3.3	1.27	-1.03	0.00	0.00
DIXDC1	85458	1.38	1.48	3.03	3.33	-1.08	0.00	0.00
DKK4	27121	0.29	0.44	2.33	15.36	-4.86	0.00	0.00
DLG4	1742	0.8	0.79	2.56	1.67	-0.92	0.00	0.00
DLK1	8788	0.13	0.17	55.6	46.42	-8.42	0.00	0.00
DLX1	1745	0.64	0.44	10.97	11.13	-3.64	0.00	0.00
DNAAF3	352909	0.39	0.42	1.28	0.63	-1.23	0.00	0.00
DNAH10	196385	0.07	0.06	0.11	0.28	-1.37	0.00	0.00
DNAJC12	56521	0.24	0.22	18.89	14.24	-6.30	0.00	0.00
DNAJC17	55192	5.96	3.95	7.65	4.6	-0.66	0.00	0.00
DNALI1	7802	0.02	0.16	1.18	0	-3.86	0.00	0.00
DNASE1L2	1775	0	0	0.27	0.08	-4.63	0.00	0.00
DNM1	1759	1.26	1.23	3.9	2.29	-1.28	0.00	0.00
DOC2B	8447	0.53	0.52	1.74	1.66	-1.81	0.00	0.00

DOCK4	9732	1.24	1.41	2.07	2.28	-0.65	0.00	0.00
DOHH	83475	12.29	13.3	19.21	18.98	-0.58	0.00	0.00
DPP4	1803	12.36	12.96	14.5	24.96	-0.62	0.00	0.00
DTX3	196403	3.94	3.06	6.51	4.94	-0.68	0.00	0.00
DUS2	54920	6.68	5.98	11.81	9.3	-0.70	0.00	0.00
DUSP1	1843	5.68	5.71	16.5	9.19	-1.15	0.00	0.00
DUSP13	51207	4.97	5.98	14.77	9.31	-1.06	0.00	0.00
DUSP4	1846	0.07	0.1	1.09	0.93	-3.64	0.00	0.00
DUSP5	1847	0.13	0.11	3.01	0.42	-4.03	0.00	0.00
DUSP6	1848	7.27	8.19	15.91	19.5	-1.19	0.00	0.00
DUSP7	1849	4.75	3.88	7.08	6.54	-0.67	0.00	0.00
DZIP1	22873	0.02	0	0.95	0.95	-7.32	0.00	0.00
EAPP	55837	15.63	13.96	10.06	9.82	-0.61	0.00	0.00
EBF4	57593	0.46	0.31	1.05	0.72	-1.35	0.00	0.00
EBI3	10148	1.28	0.94	6.62	1.09	-1.88	0.00	0.00
ECM1	1893	0.28	0.28	0.65	1.12	-1.91	0.00	0.00
EEF1A2	1917	0.11	0.14	1.91	0.5	-3.28	0.00	0.00
EGFL7	51162	2.69	3.13	8.07	3.3	-0.93	0.00	0.00
EGR1	1958	2.56	2.88	5.84	10.02	-1.55	0.00	0.00
EHD2	30846	0	0.09	1.89	0.37	-4.58	0.00	0.00
EIF5A2	56648	0.75	0.67	1.06	2.12	-0.87	0.00	0.00
ELFN1	392617	1.64	1.29	2.9	2.72	-0.95	0.00	0.00
ELMO3	79767	0.76	0.83	1.63	1.15	-0.78	0.00	0.00
ELOF1	84337	37.28	35.92	57.35	52.21	-0.58	0.00	0.00
EMILIN1	11117	13.46	12.49	43.48	29.11	-1.51	0.00	0.00
EML2	24139	3.89	3.15	7.61	3.41	-0.60	0.00	0.00
EMP3	2014	2.52	2.69	13.11	6.86	-2.05	0.00	0.00
ENHO	375704	0.65	0.54	1.44	1.65	-1.37	0.00	0.00
ENKUR	219670	0.06	0.04	0.18	0.27	-2.09	0.00	0.00
EPB41L1	2036	12.32	12.18	19.44	18.61	-0.62	0.00	0.00
EPHA2	1969	37.88	38.76	81.27	62.11	-0.89	0.00	0.00
EPN3	55040	2.04	1.49	2.46	3.51	-0.84	0.00	0.00
EPPK1	83481	3.99	4.31	3.4	1.99	-4.72	0.00	0.00

EPS8L3	79574	15.05	14.07	26.5	18.83	-0.67	0.00	0.00
EREG	2069	0.19	0.21	0.69	0.28	-1.31	0.00	0.00
ESAM	90952	1.28	1.34	20.54	12.63	-3.18	0.00	0.00
ESYT3	83850	0.04	0.01	0.14	0.2	-2.93	0.00	0.00
EVA1C	59271	0.14	0.05	0.52	0.44	-1.06	0.00	0.00
EXOC3L1	283849	0.21	0.22	0.86	0.88	-1.65	0.00	0.00
EXOSC6	118460	12.05	10.7	16.17	19.26	-0.64	0.00	0.00
EXTL1	2134	0.09	0.01	0.57	0.2	-2.97	0.00	0.00
F12	2161	6.45	5.92	9.04	7.19	-0.80	0.00	0.00
F13B	2165	0	0	0.1	0.26	-4.93	0.00	0.00
F2R	2149	1.94	1.53	3.45	2.21	-0.72	0.00	0.00
F2RL1	2150	6.82	7.47	11.17	10.81	-0.63	0.00	0.00
FABP3	2170	0.11	0.38	1.48	1.68	-2.56	0.00	0.00
FADS6	283985	0.13	0.22	2.33	3.67	-5.05	0.00	0.00
FAH	2184	47.62	49.07	94.15	94.24	-0.93	0.00	0.00
FAIM2	23017	0	0	0.02	0	-0.67	0.00	0.00
FAM129C	199786	0.25	0.19	0.43	0.37	-0.79	0.00	0.00
FAM131C	348487	0.36	0.39	2.42	0.33	-1.82	0.00	0.00
FAM155B	27112	2.87	2.75	6.07	5.54	-1.05	0.00	0.00
FAM162B	221303	0	0	0.65	0.75	-5.83	0.00	0.00
FAM167B	84734	0.25	0.56	4.6	3.37	-3.30	0.00	0.00
FAM171A2	284069	2.78	2.56	10.16	3.2	-1.44	0.00	0.00
FAM181A	90050	0.6	0.61	1.44	1.17	-1.13	0.00	0.00
FAM20A	54757	0.75	0.5	0.3	0.57	-1.46	0.00	0.00
FAM214B	80256	1.53	1.52	3.01	2.54	-0.81	0.00	0.00
FAM222B	55731	7.4	7.3	11.92	10.82	-0.62	0.00	0.00
FAM234B	57613	1.26	1.4	2.78	1.62	-0.73	0.00	0.00
FAM241A	132720	1.84	1.66	2.34	3.07	-0.67	0.00	0.00
FAM53B	9679	3.11	3.52	5.53	5.5	-0.68	0.00	0.00
FAM78A	286336	0.1	0.02	1.24	0.93	-4.10	0.00	0.00
FAM86B1	85002	4.7	4.37	8.34	5.16	-0.61	0.00	0.00
FAM86B3P	286042	1	1.05	1.79	1.48	-0.58	0.00	0.00
FAM86DP	692099	6.86	6.87	12.61	14.1	-0.84	0.00	0.00

FAM86FP	653113	1.38	1.3	3.52	2.37	-1.12	0.00	0.00
FBLN1	2192	87.42	91.23	184.49	120.4	-0.77	0.00	0.00
FBLN5	10516	0.03	0.07	0.8	1.33	-4.94	0.00	0.00
FBXL22	283807	1.61	1.8	3.26	3.5	-0.95	0.00	0.00
FBXL8	55336	1.85	1.47	3.11	2.92	-0.86	0.00	0.00
FCHO1	23149	1.11	0.93	4.47	4.09	-2.08	0.00	0.00
FES	2242	3.33	2.72	5.92	6.03	-0.88	0.00	0.00
FETUB	26998	1.63	1.24	3.05	1.97	-0.66	0.00	0.00
FGB	2244	2.13	2.22	5.56	10.6	-1.89	0.00	0.00
FGD1	2245	9.37	9.08	7.11	6.76	-1.94	0.00	0.00
FGF17	8822	0.03	0.07	0.47	0.23	-2.69	0.00	0.00
FGFRL1	53834	40.75	40.43	64.87	59.25	-0.60	0.00	0.00
FHL2	2274	5.24	4.23	17.08	8.18	-1.45	0.00	0.00
FIBCD1	84929	0.73	0.79	5.73	3.71	-2.46	0.00	0.00
FILIP1L	11259	0.82	0.78	1.65	1.45	-1.07	0.00	0.00
FITM1	161247	1.42	1.76	15.56	15.91	-3.31	0.00	0.00
FJX1	24147	0.52	0.28	2.98	2.24	-0.95	0.00	0.00
FKBP10	60681	61.5	60.22	108.35	74.34	-0.58	0.00	0.00
FKBP7	51661	1.76	2.65	4.23	3.46	-0.82	0.00	0.00
FLJ31356	403150	0.03	0.17	0.21	0.25	-1.16	0.00	0.00
FLJ46906	441172	0.9	1.36	3.67	1.75	-1.25	0.00	0.00
FMNL1	752	0.57	0.47	1.32	0.7	-0.93	0.00	0.00
FNDC10	643988	1.19	0.84	2.54	2.5	-1.30	0.00	0.00
FNDC4	64838	14	14.87	27.02	29.26	-0.97	0.00	0.00
FOS	2353	0.1	0.51	1.18	1.24	-2.01	0.00	0.00
FOSL2	2355	3.13	2.78	6.53	4.98	-1.03	0.00	0.00
FOXC1	2296	0.49	0.55	1.4	1.44	-1.45	0.00	0.00
FPGT	8790	0	0	0.06	0.14	-5.16	0.00	0.00
FRAT1	10023	1.82	2.09	3.12	3.57	-0.78	0.00	0.00
FRG1EP	102723390	0.05	0.05	0.1	0.1	-0.85	0.00	0.00
FRMD3	257019	1.97	1.62	2.2	3.78	-0.78	0.00	0.00
FRMD5	84978	0	0.04	0.22	0.11	-2.93	0.00	0.00
FRY	10129	1.08	1.16	1.73	1.89	-0.68	0.00	0.00

FSD1	79187	0.09	0	1.09	0.28	-3.90	0.00	0.00
FST	10468	0.41	0.61	5	4.79	-3.16	0.00	0.00
FSTL4	23105	1.03	0.85	2.18	1.55	-1.08	0.00	0.00
FURIN	5045	28.79	29.26	59.42	66.18	-1.12	0.00	0.00
FUT1	2523	0.03	0.03	0.38	0.04	-2.94	0.00	0.00
FUT11	170384	0.19	0.08	2.3	1.57	-2.93	0.00	0.00
FUT8	2530	1.07	1.06	1.41	1.81	-0.70	0.00	0.00
FZD7	8324	0	0.06	0.11	0.18	-2.42	0.00	0.00
GADD45A	1647	15.37	16.08	34.59	24.98	-0.92	0.00	0.00
GADD45B	4616	1.65	2.53	11.56	5.52	-2.04	0.00	0.00
GALNT16	57452	0.28	0.16	1.63	1.1	-2.62	0.00	0.00
GATA2	2624	4	5.01	6.99	8.86	-0.84	0.00	0.00
GCKR	2646	5.27	6.41	10.66	6.78	-0.67	0.00	0.00
GDF11	10220	0.33	0.28	0.93	0.39	-1.07	0.00	0.00
GDF15	9518	123.02	121.09	545.56	201.87	-1.64	0.00	0.00
GDI1	2664	0	0	0.3	1.46	-7.79	0.00	0.00
GGA1	26088	22.22	21.58	59.05	50.24	-1.06	0.00	0.00
GGH	8836	18.92	19.08	32.42	38.44	-0.90	0.00	0.00
GGT5	2687	0.3	0.24	8.75	4.91	-3.62	0.00	0.00
GIP	2695	0.62	0.79	2.6	2.5	-1.86	0.00	0.00
GJC2	57165	0.41	0.22	0.66	0.36	-2.51	0.00	0.00
GLCCI1	113263	0.26	0.21	0.51	0.67	-1.11	0.00	0.00
GLIPR1	11010	1.11	0.53	1.24	1.16	-0.69	0.00	0.00
GLIS2	84662	0.12	0.55	0.71	0.92	-1.28	0.00	0.00
GLRX	2745	47.12	47.41	138.67	148.13	-1.60	0.00	0.00
GLYATL1	92292	0.04	0.11	0.24	0.48	-1.97	0.00	0.00
GNA11	2767	19.09	18.43	28.18	30.06	-0.63	0.00	0.00
GNG12	55970	0.05	0.03	0.43	0.68	-2.28	0.00	0.00
GOLGA8IP	283796	0	0	0	0	-0.86	0.00	0.00
GOT1	2805	0.61	1.84	3.65	1.78	-1.07	0.00	0.00
GP1BB	2812	0.1	0.19	0.65	0.95	-2.46	0.00	0.00
GPC1	2817	3.91	3.46	9.41	7.39	-1.18	0.00	0.00
GPC2	221914	0	0.04	0.26	0.1	-3.25	0.00	0.00

GPCPD1	56261	0.6	0.67	1.02	1.04	-0.70	0.00	0.00
GPR162	27239	0.02	0.02	0.09	0.07	-2.04	0.00	0.00
GPR3	2827	0.92	1.17	2.98	2.28	-1.33	0.00	0.00
GPR35	2859	15.11	16.68	32.84	31.33	-1.09	0.00	0.00
GPR39	2863	1.56	1.75	4.88	4.68	-1.50	0.00	0.00
GPRC5C	55890	31.65	32.52	67.54	73.36	-1.09	0.00	0.00
GPSM1	26086	1.36	1.02	5.66	2.79	-1.92	0.00	0.00
GPSM3	63940	0.76	0.69	2.26	1.24	-1.27	0.00	0.00
GRB10	2887	0.51	0.43	5	4.13	-3.24	0.00	0.00
GREB1L	80000	0.09	0.08	0.12	0.33	-1.45	0.00	0.00
GRIN2C	2905	0.7	0.63	1.81	1.02	-1.00	0.00	0.00
GRIP2	80852	0.02	1.09	2.16	0.78	-1.39	0.00	0.00
GRK5	2869	5.59	7.67	14.31	15.2	-1.15	0.00	0.00
GRPEL2-AS1	106144529	0	0	0.13	0.14	-5.57	0.00	0.00
GSDME	1687	13.57	15.84	27.33	22.89	-0.75	0.00	0.00
GUCY2EP	390226	0	0.01	0.08	0.01	-3.11	0.00	0.00
GYS2	2998	0	0	0.1	0.33	-5.46	0.00	0.00
H19	283120	0	0.04	1.22	0	-4.79	0.00	0.00
H3F3AP4	440926	1.27	0	1.38	1.65	-1.32	0.00	0.00
HAMP	57817	56.47	55.7	199.1	86.56	-1.34	0.00	0.00
HAS3	3038	0.36	0.4	1.3	1.31	-1.86	0.00	0.00
HBA1	3039	3.02	0.15	9.15	12.54	-2.78	0.00	0.00
HBG2	3048	0	0.22	0	0	-0.59	0.00	0.00
HCN2	610	0.11	0.16	0.4	0.39	-1.62	0.00	0.00
HES1	3280	0.31	0.05	6.46	5.42	-4.98	0.00	0.00
HES6	55502	25.46	26.86	44	45.03	-0.74	0.00	0.00
HHIPL2	79802	1.66	0.67	3.53	0.84	-0.72	0.00	0.00
HID1	283987	8.65	8.88	15	13.94	-0.72	0.00	0.00
HIST1H2AJ	8331	1.01	0.36	0.54	0.1	-0.91	0.00	0.00
HIST2H2AA3	8337	0	0	12.86	0	-7.68	0.00	0.00
HIST2H2BA	337875	0	0	0	0	-5.04	0.00	0.00
HLA-E	3133	0.36	0.44	1.12	1.55	-1.75	0.00	0.00
HLX	3142	0.11	0	0.32	0.27	-2.41	0.00	0.00

HMGA1	3159	615.63	597.8	1063.05	805.76	-0.62	0.00	0.00
HMX2	3167	0	0	0.7	0.15	-5.61	0.00	0.00
HOMER2	9455	5.86	6.49	12.42	7.54	-0.60	0.00	0.00
HOMER3	9454	20.21	19.84	40.69	36.91	-0.95	0.00	0.00
HPX	3263	1.42	2.01	4.59	6.71	-1.72	0.00	0.00
HR	55806	0.21	0.19	0.96	0.19	-1.49	0.00	0.00
HRC	3270	2.65	2.23	9.51	4.83	-1.55	0.00	0.00
HSD17B8	7923	0.4	0	5.57	3.44	-4.51	0.00	0.00
HSF4	3299	0.94	0.75	2.05	1.05	-0.60	0.00	0.00
HSPA12A	259217	2.64	2.17	6.1	4.66	-1.13	0.00	0.00
HSPB2	3316	0	0	1.64	1.12	-6.26	0.00	0.00
HSPB8	26353	0.39	0.5	2.06	1.6	-2.05	0.00	0.00
HSPG2	3339	13.27	12.42	26.89	14.67	-0.69	0.00	0.00
HTN3	3347	0	0	0.12	0	-0.62	0.00	0.00
ID1	3397	200.51	194.1	420.06	426.02	-1.09	0.00	0.00
ID3	3399	72.87	69.29	196.63	202.6	-1.49	0.00	0.00
IER2	9592	14.02	12.98	25.12	25.79	-0.91	0.00	0.00
IER5L	389792	7.84	7.7	15.43	13.85	-0.89	0.00	0.00
IFITM1	8519	18.46	21.93	53.37	20.42	-0.74	0.00	0.00
IFITM10	402778	0.75	0.62	1.99	1.27	-1.26	0.00	0.00
IGDCC3	9543	18.64	18.77	39.29	31.61	-0.89	0.00	0.00
IGF2	3481	36.29	30.56	197.35	76.84	-2.01	0.00	0.00
IGFBP1	3484	43.9	47.34	123.55	44.52	-0.88	0.00	0.00
IGFBP2	3485	4.15	4.26	13.31	5.61	-1.19	0.00	0.00
IGFBP7	3490	0.03	0.02	0.7	1.6	-2.95	0.00	0.00
IGFBPL1	347252	0.03	0.08	0.49	0.44	-3.03	0.00	0.00
IGSF1	3547	14.95	13.01	71.02	83.92	-2.47	0.00	0.00
IL11	3589	0.21	0.09	2.53	0.14	-3.14	0.00	0.00
IL17B	27190	0.15	0.09	1.37	4.41	-3.76	0.00	0.00
IL18	3606	0.7	1.37	0.89	3.57	-1.04	0.00	0.00
IL20RB-AS1	107986136	0.16	0	0.21	0.44	-2.04	0.00	0.00
IL23A	51561	0.55	0.82	1.13	0.84	-1.31	0.00	0.00
IL4R	3566	13.23	12.75	22.68	18.09	-0.60	0.00	0.00

IMPDH1	3614	22.74	21.77	35.74	35.97	-0.69	0.00	0.00
INHBE	83729	16.91	16.88	32.03	19.74	-0.61	0.00	0.00
INO80C	125476	15.48	13.94	22.6	24.72	-0.66	0.00	0.00
INPP1	3628	10.56	9.84	19	25.06	-1.11	0.00	0.00
INPP5J	27124	0.08	0.35	0.61	0.54	-1.36	0.00	0.00
INS-IGF2	723961	57.81	60.21	300.08	112.68	-1.80	0.00	0.00
IP6K3	117283	0.04	0.02	0.58	0.06	-3.48	0.00	0.00
IQCC	55721	1.88	1.87	1.55	1.78	-0.85	0.00	0.00
IRX3	79191	0.4	0.27	1.97	0.77	-2.06	0.00	0.00
IST1	9798	30.89	31.01	48.49	44.02	-0.58	0.00	0.00
ITGA10	8515	0.1	0.15	0.99	0.73	-2.68	0.00	0.00
ITGA3	3675	0.44	0.35	1.01	0.55	-0.93	0.00	0.00
ITGB2-AS1	100505746	0.26	0.22	0.82	0.53	-1.47	0.00	0.00
ITGB7	3695	1.5	1.05	1.09	1.3	-2.73	0.00	0.00
ITIH1	3697	0.05	0.08	0.54	0.37	-2.72	0.00	0.00
ITPR1	3708	0.5	0.62	0.96	1.28	-0.93	0.00	0.00
ITPRIP	85450	0.17	0.08	2.66	1.69	-4.23	0.00	0.00
JAG2	3714	0.06	0.05	0.28	0.12	-2.28	0.00	0.00
JAML	120425	1.36	1.72	6.55	5.84	-1.81	0.00	0.00
JSRP1	126306	16.4	13.88	23.95	23.3	-0.60	0.00	0.00
JUNB	3726	11.32	10.05	32.11	23.97	-1.39	0.00	0.00
JUND	3727	78.49	74.53	176.56	109.23	-0.90	0.00	0.00
KAZN	23254	23.12	23.27	32.75	42.09	-0.63	0.00	0.00
KCNC4	3749	0.22	0.44	0.78	0.69	-1.10	0.00	0.00
KCNK13	56659	0	0.02	1.96	3.72	-8.03	0.00	0.00
KCNK3	3777	0.01	0	0.54	0.27	-5.98	0.00	0.00
KCNK4-TEX40	106780802	0.12	0.13	0.2	0.36	-1.21	0.00	0.00
KCNN4	3783	0.07	0.04	0.58	0.12	-2.56	0.00	0.00
KCTD17	79734	4.19	3.55	9.06	6.3	-1.07	0.00	0.00
KDELC1	79070	4.32	5.16	8.88	6.99	-0.74	0.00	0.00
KDM2B	84678	28.49	34.61	44.54	54.69	-0.59	0.00	0.00
KIF21B	23046	0.14	0.08	0.38	0.14	-1.52	0.00	0.00
KIF3C	3797	3.14	3.09	10.97	7.99	-1.61	0.00	0.00

KLC2	64837	14.09	13.74	23.21	19.85	-0.64	0.00	0.00
KLF12	11278	0.27	0.41	0.71	0.67	-1.06	0.00	0.00
KLF16	83855	17.67	16.67	29.85	25	-0.69	0.00	0.00
KLHDC7B	113730	0	0.02	0.36	0.11	-4.56	0.00	0.00
KLHDC8B	200942	5.77	4.82	9.49	7.49	-0.68	0.00	0.00
KLHL13	90293	0	0	0	0	-4.00	0.00	0.00
KLHL23	151230	0.43	0	0.87	0.29	-1.44	0.00	0.00
KLHL26	55295	2.43	2.34	4.56	4.48	-0.78	0.00	0.00
KLRC3	3823	0.16	0.1	3.17	2.97	-4.79	0.00	0.00
KLRC4-KLRK1	100528032	0.12	0.08	0.38	0.14	-1.60	0.00	0.00
KNDC1	85442	0.04	0.09	0.73	0.12	-1.69	0.00	0.00
KPTN	11133	5.83	5.67	6.14	6.05	-5.15	0.00	0.00
KRTAP3-1	83896	0	0	0.56	1.22	-5.02	0.00	0.00
KYAT1	883	5.95	5.66	12.49	8.71	-0.80	0.00	0.00
LAIR2	3904	0.1	0.19	2.64	2.52	-4.25	0.00	0.00
LAMP1	3916	149.83	148.09	230.77	250.43	-0.69	0.00	0.00
LAPTM5	7805	0.52	0.8	1.68	1.33	-1.11	0.00	0.00
LARP6	55323	0.22	0.38	3.12	0.88	-3.41	0.00	0.00
LCAT	3931	10.24	8.51	19.68	39.6	-1.66	0.00	0.00
LCK	3932	2.22	1.75	6.04	5.38	-1.55	0.00	0.00
LCLAT1	253558	2.46	2.41	1.41	1.64	-0.73	0.00	0.00
LDLRAD2	401944	0.03	0.04	0.15	0.24	-2.35	0.00	0.00
LDLRAD3	143458	0.09	0.23	0.79	0.27	-0.58	0.00	0.00
LETM2	137994	0.28	0.22	1.66	1.51	-2.86	0.00	0.00
LGALS1	3956	22.46	23.77	197.82	108.86	-2.73	0.00	0.00
LGALS9	3965	0.89	0.7	2.02	1.59	-1.09	0.00	0.00
LGALSL-DT	105374771	0.21	0.14	0.71	0.69	-1.44	0.00	0.00
LGI1	9211	0	0	5.35	4.23	-9.13	0.00	0.00
LIN7A	8825	1.67	0.97	1	2.62	-0.73	0.00	0.00
LINC00345	105370208	0	0	0.23	0.27	-6.57	0.00	0.00
LINC00524	338002	0	0	0	0	-4.54	0.00	0.00
LINC00869	57234	0.57	0.41	2.2	1.97	-1.95	0.00	0.00
LINC00958	100506305	0.1	0.01	0.5	0.33	-3.66	0.00	0.00

LINC00987	100499405	0.02	0.03	0.06	0.07	-1.28	0.00	0.00
LINC01011	401232	0.1	0.08	0.27	1.08	-2.29	0.00	0.00
LINC01089	338799	3.8	3.56	6.06	8	-0.59	0.00	0.00
LINC01091	285419	0.57	0.21	0.69	1.09	-0.73	0.00	0.00
LINC01134	100133612	0.93	1.46	1.16	2.49	-0.62	0.00	0.00
LINC01239	441389	0	0	0.02	0.02	-3.87	0.00	0.00
LINC01269	103695436	0.31	0.56	1.47	1.03	-1.51	0.00	0.00
LINC01271	101927586	0.43	0.45	0.88	0.43	-0.59	0.00	0.00
LINC01285	101928287	0.02	0	0.11	0.09	-3.11	0.00	0.00
LINC01465	283416	0.07	0.12	0.11	0.12	-0.62	0.00	0.00
LINC01503	100506119	0.29	0.46	1.23	1.41	-2.05	0.00	0.00
LINC01669	102724354	0.22	0.12	0.64	0.31	-1.43	0.00	0.00
LINC01820	105374582	0.17	0.19	2.37	2.37	-3.65	0.00	0.00
LINC02435	107986311	0.03	0.03	0.09	0.05	-1.39	0.00	0.00
LINC02604	644794	1.84	1.49	0.87	1.25	-1.25	0.00	0.00
LIPH	200879	0.24	0.41	0.81	1.58	-2.07	0.00	0.00
LIX1L	128077	2.06	2.86	4.09	4.78	-0.85	0.00	0.00
LMBR1L	55716	2.78	2.33	5.33	2.92	-0.63	0.00	0.00
LMO2	4005	0	0	0.04	0	-1.39	0.00	0.00
LMO4	8543	7.25	9.42	18.41	15.62	-0.82	0.00	0.00
LONP1	9361	60.97	61.12	123.46	96.59	-0.78	0.00	0.00
LOXL2	4017	0.24	0.15	0.97	0.36	-1.11	0.00	0.00
LPCAT4	254531	5.62	5.9	18.21	8.58	-1.22	0.00	0.00
LRG1	116844	44.28	42.33	71.01	60.66	-0.61	0.00	0.00
LRRC25	126364	1.03	0.61	2.14	3.54	-1.72	0.00	0.00
LRRC27	80313	1.89	1.82	2.24	4.23	-0.66	0.00	0.00
LRRC37A3	374819	0.64	0.56	1.19	1.13	-0.67	0.00	0.00
LRRC73	221424	0.47	0.59	1.57	0.73	-1.15	0.00	0.00
LRRC8E	80131	3.83	4.1	6.98	5.92	-6.65	0.00	0.00
LRRN4	164312	3.18	3.21	6.73	5.66	-0.89	0.00	0.00
LSP1	4046	1.12	0.9	13.3	2.53	-3.10	0.00	0.00
LSP1P5	645166	10.21	9.82	27.5	17.51	-1.17	0.00	0.00
LTBP2	4053	0.93	0.8	2.37	1.8	-1.07	0.00	0.00

LTBP4	8425	0.14	0.12	0.52	0.16	-1.37	0.00	0.00
LY6G5B	58496	5.93	5.08	4.55	6.21	-0.85	0.00	0.00
LY96	23643	1.6	1.79	3.69	4.11	-1.15	0.00	0.00
LYL1	4066	1.78	2.01	3.39	3.61	-1.11	0.00	0.00
LYVE1	10894	0	0	3.12	4.18	-9.38	0.00	0.00
MACROD2	140733	0	0.01	0.04	0.16	-3.27	0.00	0.00
MAF	4094	0.02	0.07	0.25	0.78	-3.53	0.00	0.00
MAG	4099	0.11	0.02	0.61	0.55	-3.14	0.00	0.00
MAGI2	9863	0.04	0	1.8	2.39	-7.02	0.00	0.00
MAGI2-AS3	100505881	0	0	2.73	4.01	-7.85	0.00	0.00
MAP1B	4131	0.01	0	0.12	0.1	-3.67	0.00	0.00
MAP2K2	5605	169.9	165.51	269.72	253.12	-0.64	0.00	0.00
MAP3K9	4293	8.74	5.02	5.7	6.92	-0.67	0.00	0.00
MAP4K2	5871	7.68	6.91	13.89	10.33	-0.71	0.00	0.00
MAP7D2	256714	1.07	1.15	3.67	3.37	-1.67	0.00	0.00
MAPK11	5600	0.36	0.19	1.13	0.56	-1.63	0.00	0.00
MAPK8IP2	23542	1.62	1.66	2.24	2.97	-0.64	0.00	0.00
MARCKS	4082	6.73	6.98	14.8	13.4	-1.04	0.00	0.00
MASP1	5648	2.98	3.63	23.31	17.29	-2.62	0.00	0.00
MAST1	22983	2.64	2.97	4.86	3.57	-0.58	0.00	0.00
MAST3	23031	3.54	3.81	5.82	4.79	-0.62	0.00	0.00
MATN3	4148	1.59	1.48	20.82	9.78	-3.32	0.00	0.00
MBNL2	10150	1.8	1.61	2.89	4.61	-1.11	0.00	0.00
MBOAT4	619373	2.33	1.71	5.1	4.86	-1.27	0.00	0.00
MC1R	4157	6.45	5.89	12.61	8.71	-0.79	0.00	0.00
MCOLN1	57192	6.67	5.89	11.86	13.99	-1.03	0.00	0.00
MED28	80306	20.32	20.5	16.48	14.48	-1.30	0.00	0.00
MEF2B	100271849	1.13	1.1	9.41	3.04	-2.48	0.00	0.00
MEIS3	56917	0.11	0.21	0.83	0.23	-1.73	0.00	0.00
MEP1A	4224	0.09	0.06	25.06	39.07	-8.91	0.00	0.00
METRNL	284207	0.13	0.24	4.14	2.77	-4.07	0.00	0.00
MFAP4	4239	0.14	0.03	0.69	0.14	-2.57	0.00	0.00
MFGE8	4240	1.44	1.3	4.35	2.48	-1.34	0.00	0.00

MIA	8190	0.09	0.11	0.12	0.12	-0.62	0.00	0.00
MICAL1	64780	3.23	2.89	6.96	5.95	-1.08	0.00	0.00
MID1	4281	0.54	0.5	0.9	1.72	-1.38	0.00	0.00
MID1IP1	58526	0.09	0.09	33.14	31.16	-8.56	0.00	0.00
MIDN	90007	14.63	13.07	23	26.46	-0.85	0.00	0.00
MIER2	54531	7.47	7.62	17.01	11.44	-0.86	0.00	0.00
MILR1	284021	0.07	0	0.5	0.13	-3.02	0.00	0.00
MINOS1-NBL1	100532736	0.29	0.27	2	2.72	-2.96	0.00	0.00
MIOX	55586	5.65	5.6	10.62	9.68	-0.77	0.00	0.00
MIR22HG	84981	3.3	5.28	5.03	4.88	-0.62	0.00	0.00
MKNK2	2872	29.98	30.26	60.6	46.98	-0.72	0.00	0.00
MLLT11	10962	1.38	1.64	4.89	2.04	-0.79	0.00	0.00
MMP11	4320	0.6	0.55	4.28	2.31	-2.52	0.00	0.00
MORN4	118812	1.06	1.13	3.12	1.42	-1.04	0.00	0.00
MOSPD1	56180	1.57	1.48	4.09	5.77	-1.69	0.00	0.00
MPP1	4354	1.4	1.75	3.01	6.52	-1.57	0.00	0.00
MPV17L2	84769	13.83	13.95	30.29	33.93	-1.20	0.00	0.00
MRAS	22808	0.03	0.01	1.55	1.53	-6.29	0.00	0.00
MRPL34	64981	65.68	66.91	118.6	119.6	-0.85	0.00	0.00
MSANTD3-TMEFF1	100526694	0.98	1.33	3.09	3.2	-1.47	0.00	0.00
MSX1	4487	6.14	5.77	13.63	16.75	-1.35	0.00	0.00
MT1E	4493	5.39	5.5	150.77	38.73	-4.14	0.00	0.00
MT1F	4494	7.78	7.09	29.15	23.72	-1.82	0.00	0.00
MT1G	4495	11.31	9.99	75.79	56.45	-2.63	0.00	0.00
MT1X	4501	30.88	34.55	97.86	50.43	-1.18	0.00	0.00
MT2A	4502	88.42	96.59	963.4	263.33	-2.73	0.00	0.00
MTMR11	10903	0.5	1.05	1.48	1.28	-0.73	0.00	0.00
MTSS1L	92154	58.77	52.9	88.75	94.77	-0.72	0.00	0.00
MXD1	4084	1.3	1.51	1.92	2.73	-0.73	0.00	0.00
MXD4	10608	37.21	37.06	28.75	38.24	-1.29	0.00	0.00
MXRA7	439921	72.64	76.35	128.17	98.27	-0.58	0.00	0.00
MYEOV	26579	0.02	0.02	0.4	0	-3.19	0.00	0.00
MYLK2	85366	0.06	0.02	0.35	0.32	-3.07	0.00	0.00

MYMX	101929726	0.07	0.07	0.76	0.53	-2.69	0.00	0.00
MYO1A	4640	1.18	1.5	2.52	4.37	-1.35	0.00	0.00
MYOM1	8736	0.91	0.73	1.29	1.17	-0.60	0.00	0.00
NADK2	133686	2.51	2.3	3.57	5.1	-0.82	0.00	0.00
NAT14	57106	0.47	0.59	11.78	6.33	-4.09	0.00	0.00
NAV2	89797	1.65	1.81	5.45	6.38	-1.77	0.00	0.00
NBDY	550643	6.75	6.24	12.94	8.53	-0.73	0.00	0.00
NBL1	4681	3.41	4.26	43.89	26.48	-3.19	0.00	0.00
NBPF1	55672	3.83	3.36	4.51	5.51	-0.58	0.00	0.00
NBPF15	284565	1.99	2.13	3.82	2.68	-0.61	0.00	0.00
NBPF25P	101929780	0.91	1.11	1.51	1.62	-0.81	0.00	0.00
NCF2	4688	0.31	0.19	0.8	0.56	-1.56	0.00	0.00
NCOA7-AS1	104355145	0	0.03	0	0	-1.07	0.00	0.00
NCS1	23413	2.04	2.1	6.13	3.43	-1.21	0.00	0.00
NDUFA4L2	56901	0.07	0.37	3.09	0.42	-3.04	0.00	0.00
NDUFS7	374291	70.58	68.98	121.45	103.66	-0.69	0.00	0.00
NECTIN2	5819	53.82	51.91	95.84	90.43	-0.81	0.00	0.00
NECTIN4	81607	0.86	0.77	2.42	1.36	-1.21	0.00	0.00
NGFR	4804	0.02	0.05	0.87	0.08	-3.91	0.00	0.00
NID1	4811	1.16	1.04	27.58	18.48	-4.40	0.00	0.00
NIFK-AS1	254128	0.16	0.26	0.14	0.12	-0.59	0.00	0.00
NINJ2	4815	3.39	3.5	6.95	5.09	-0.66	0.00	0.00
NKX2-5	1482	0.64	0.37	2.75	1.5	-2.09	0.00	0.00
NKX6-3	157848	0.16	0.16	0.63	0.51	-1.83	0.00	0.00
NLRP12	91662	0.14	0.11	0.03	0.02	-1.05	0.00	0.00
NME3	4832	0.63	0.87	11.25	9.63	-3.21	0.00	0.00
NOMO2	283820	56.99	57.75	103.33	96.51	-0.80	0.00	0.00
NOP10	55505	53.92	78.15	75.65	127.17	-0.62	0.00	0.00
NOS2	4843	0.6	0.46	2.94	6.4	-3.12	0.00	0.00
NPAS1	4861	1.05	0.95	4.41	4.03	-1.95	0.00	0.00
NPDC1	56654	10.41	9.41	24.8	19.99	-1.15	0.00	0.00
NPEPPS	9520	27.42	26.92	16.14	19.39	-0.85	0.00	0.00
NPM2	10361	15.78	16.08	25.96	23.53	-0.65	0.00	0.00

NPPC	4880	0	0	0.04	0	-1.41	0.00	0.00
NR0B2	8431	81.91	81.89	132.76	159.98	-0.85	0.00	0.00
NR5A2	2494	0	0	0.26	0.73	-6.51	0.00	0.00
NR6A1	2649	4.36	4.65	10.49	10.48	-1.20	0.00	0.00
NRP1	8829	0.05	0.03	6.3	4.72	-7.47	0.00	0.00
NRP2	8828	0	0	5.19	2.73	-10.56	0.00	0.00
NRTN	4902	4.23	5.05	8.35	5.14	-0.59	0.00	0.00
NT5DC1	221294	10.03	8.8	5.45	6.05	-5.14	0.00	0.00
NTSR1	4923	0.09	0.03	0.6	0.16	-2.60	0.00	0.00
NUAK1	9891	0.09	0.06	0.74	0.78	-3.25	0.00	0.00
NUAK2	81788	1.28	1.4	2.05	2.5	-0.73	0.00	0.00
NUTM2F	54754	0.07	0.02	0.16	0.41	-2.62	0.00	0.00
NXF3	56000	0	0	0.07	0	-3.68	0.00	0.00
NXPE2	120406	0.03	0	0.03	0.03	-0.63	0.00	0.00
NXPH3	11248	0.05	0.11	0.75	0.98	-3.52	0.00	0.00
NXPH4	11247	0.22	0.49	1.39	0.76	-1.57	0.00	0.00
NYAP1	222950	1.17	1.16	2.68	1.87	-1.06	0.00	0.00
ODF3L2	284451	0.47	0.58	2.61	0.72	-1.86	0.00	0.00
OGDHL	55753	0.13	0.11	0.44	0.35	-1.70	0.00	0.00
ORAI2	80228	2.06	1.47	2.49	2.56	-0.59	0.00	0.00
OSCAR	126014	0.36	0.7	2.9	0.39	-1.57	0.00	0.00
OSER1-DT	100505783	1.37	0.89	3.65	1.62	-1.07	0.00	0.00
OTX1	5013	4.87	4.96	7.61	8.86	-0.75	0.00	0.00
OXCT2	64064	0.4	0.36	1.75	0.78	-1.73	0.00	0.00
P2RX5	5026	2.5	2.3	8.6	7.48	-1.74	0.00	0.00
PACSIN2	11252	34.17	34.38	34.16	29.58	-1.05	0.00	0.00
PAFAH1B1	5048	6.6	6.92	6.95	7.82	-1.07	0.00	0.00
PAGE1	8712	0.19	0.19	0.97	1.36	-2.61	0.00	0.00
PALM3	342979	1.81	2.23	2.99	4.75	-0.76	0.00	0.00
PAQR5	54852	0.11	0.06	0.63	0.32	-2.83	0.00	0.00
PAQR9	344838	0	0	5.75	8.78	-10.11	0.00	0.00
PARD6A	50855	2.38	1.85	4.46	3.42	-0.90	0.00	0.00
PBX3	5090	0.28	0.2	0.53	0.83	-1.56	0.00	0.00

PCDHB13	56123	0.26	0.09	0.48	0.39	-1.35	0.00	0.00
PCDHB8	56128	0.02	0.01	0.39	0.4	-4.64	0.00	0.00
PCDHGB1	56104	0.01	0.05	0.32	0.29	-3.44	0.00	0.00
PCDHGC3	5098	0	0.02	0.42	0	-4.32	0.00	0.00
PCED1B	91523	0.6	0.54	1.61	1.1	-1.13	0.00	0.00
PCK1	5105	0.02	0.02	1.32	1.12	-6.01	0.00	0.00
PCOLCE	5118	3.55	2.7	5.68	5.14	-0.79	0.00	0.00
PCOLCE2	26577	0	0	4.48	4.62	-9.33	0.00	0.00
PCSK9	255738	3.29	3.11	2.8	8.52	-0.77	0.00	0.00
PCYOX1L	78991	1.12	0.76	3.4	0.81	-1.09	0.00	0.00
PDCD1	5133	0.08	0.08	0.77	0.08	-2.44	0.00	0.00
PDE4C	5143	0.47	0.69	1.5	0.87	-1.32	0.00	0.00
PDE4D	5144	0.31	0.29	1.37	1.72	-1.93	0.00	0.00
PDE6D	5147	6.24	6.6	13.04	8.32	-0.70	0.00	0.00
PDGFB	5155	0.61	0.91	2.72	2.8	-1.68	0.00	0.00
PDGFRB	5159	1	0.97	2.21	3.8	-1.97	0.00	0.00
PDLIM7	9260	21.48	22.63	43.88	29.44	-0.78	0.00	0.00
PEAR1	375033	0	0.02	0.15	0.05	-3.93	0.00	0.00
PEG10	23089	38.79	38.46	102.31	188.35	-1.91	0.00	0.00
PFKFB1	5207	0.73	0.55	1.25	1.76	-1.17	0.00	0.00
PFKFB3	5209	2.6	2.8	5.51	4.21	-0.88	0.00	0.00
PGF	5228	4.61	4.64	14.08	13.78	-1.59	0.00	0.00
PHC1	1911	1.62	1.02	2.71	1.48	-0.65	0.00	0.00
PHLDA1	22822	7.66	8.97	21.81	14.57	-1.13	0.00	0.00
PHLDA2	7262	10.49	9.24	38.37	27.45	-1.74	0.00	0.00
PI4K2A	55361	7.94	8.03	12.04	12.62	-0.63	0.00	0.00
PIP4K2A	5305	6.96	6.89	9.59	14.05	-0.77	0.00	0.00
PIP4P2	55529	0.96	1.4	2.39	4.02	-1.31	0.00	0.00
PIP5K1C	23396	10.01	9.51	14.68	14.91	-0.60	0.00	0.00
PITPNM2	57605	2.04	1.49	3.18	4.2	-1.03	0.00	0.00
PKDCC	91461	5.62	5.96	11.59	9.25	-0.93	0.00	0.00
PLA2G15	23659	8.17	7.94	18.33	21.43	-1.30	0.00	0.00
PLA2G16	11145	0.42	0.18	2.28	1.92	-2.92	0.00	0.00

PLA2G3	50487	0	0	0.16	0.25	-5.42	0.00	0.00
PLA2G4B	100137049	0	0	1.3	0	-7.02	0.00	0.00
PLA2G4D	283748	0.06	0.09	0.52	0.12	-2.09	0.00	0.00
PLAC1	10761	1.03	0.66	2.12	1.65	-1.22	0.00	0.00
PLAC8	51316	9.71	11.99	21.44	21.36	-0.78	0.00	0.00
PLAT	5327	0.55	0.65	0.81	1.43	-0.92	0.00	0.00
PLAUR	5329	1.36	1.47	4.65	2.08	-1.11	0.00	0.00
PLCD3	113026	11.07	10.16	33.93	32.2	-1.61	0.00	0.00
PLCH2	9651	0.67	0.66	1.41	1.39	-1.10	0.00	0.00
PLCL2	23228	0.01	0	0	0	-2.53	0.00	0.00
PLCXD1	55344	31.79	29.97	44.3	50.97	-0.75	0.00	0.00
PLEKHG5	57449	3.34	3.55	7.51	3.95	-0.77	0.00	0.00
PLEKHM1	9842	4.29	3.93	6.29	6.25	-0.58	0.00	0.00
PLEKHO1	51177	4.36	3.81	8.64	6.31	-0.89	0.00	0.00
PLIN1	5346	0	0.06	0.04	0.3	-2.74	0.00	0.00
PLIN3	10226	62.6	63.46	103.72	93.85	-0.65	0.00	0.00
PLIN4	729359	0.93	1.24	1.89	1.53	-0.63	0.00	0.00
PLP1	5354	0	0	0.9	0.21	-6.93	0.00	0.00
PLPPR1	54886	0.07	0	0.46	0.23	-3.30	0.00	0.00
PLPPR2	64748	3.01	3.1	5.28	4.78	-0.71	0.00	0.00
PLXNA2	5362	2.03	2.09	5.94	6.59	-1.57	0.00	0.00
PLXNC1	10154	0.17	0.38	0.38	0.86	-1.46	0.00	0.00
PLXND1	23129	22.85	23.21	47.73	38.31	-0.85	0.00	0.00
PMAIP1	5366	0	0	0.35	0.29	-5.48	0.00	0.00
PNPLA6	10908	13.46	13.4	26.69	24.09	-0.92	0.00	0.00
PNRC1	10957	0.47	0.6	1.08	1.05	-1.03	0.00	0.00
PODN	127435	0.04	0.03	0.21	0.19	-2.46	0.00	0.00
PODXL	5420	0.11	0.14	0.53	0.27	-1.64	0.00	0.00
POM121L9P	29774	0.04	0.04	0.12	0.09	-1.41	0.00	0.00
PON1	5444	0.23	0.15	1.35	2.46	-3.29	0.00	0.00
PORCN	64840	2.47	2.76	7.59	5.55	-1.21	0.00	0.00
POU6F1	5463	0.41	0.32	0.62	0.62	-0.96	0.00	0.00
PPARGC1B	133522	0.06	0.03	0.35	0.23	-2.74	0.00	0.00

PPFIA3	8541	2.37	2.15	4.93	4.46	-1.04	0.00	0.00
PPFIA4	8497	0.01	0.02	0.26	0.04	-2.31	0.00	0.00
PPIP5K1P1-CATSPER2	110006325	0.18	0.19	0.37	0.34	-0.85	0.00	0.00
PPM1J	333926	0.08	0.2	1.07	0.23	-2.35	0.00	0.00
PPM1L	151742	0.05	0.02	0.24	0.39	-3.79	0.00	0.00
PPP1R15A	23645	9.24	10	17.32	11.57	-0.59	0.00	0.00
PPP1R18	170954	3.46	2.69	6.28	5.21	-0.90	0.00	0.00
PPP1R3G	648791	0.16	0.26	0.71	0.92	-1.97	0.00	0.00
PPP2R5B	5526	1.89	2.17	5.45	2.84	-0.85	0.00	0.00
PPT2	9374	15.12	17.23	24.84	24.39	-0.61	0.00	0.00
PRICKLE2	166336	0.22	0.33	0.62	0.9	-1.39	0.00	0.00
PRIMA1	145270	0.04	0.03	0.78	0.75	-1.93	0.00	0.00
PRKACA	5566	44.23	46.23	68.66	84.26	-0.75	0.00	0.00
PRKD1	5587	0.02	0	0.72	0.68	-6.48	0.00	0.00
PRLR	5618	0	0	0.06	0.22	-4.75	0.00	0.00
PRMT7	54496	10.97	11.44	19.13	17.79	-0.76	0.00	0.00
PROM1	8842	0	0	3.33	4.49	-10.14	0.00	0.00
PROSER2	254427	6.82	5.81	14.27	6.34	-0.63	0.00	0.00
PROSER3	148137	1.81	2.1	3.41	2.45	-0.67	0.00	0.00
PRR22	163154	1.05	0.25	1.72	1.36	-1.26	0.00	0.00
PRR26	414235	0.12	0.15	0.57	0.41	-1.67	0.00	0.00
PRR29	92340	0.05	0.09	1.31	0.49	-3.00	0.00	0.00
PSD2	84249	0.43	0.46	1.39	0.49	-0.89	0.00	0.00
PSKH1	5681	23.52	22.06	36.04	34.75	-0.64	0.00	0.00
PTAFR	5724	0.32	0.36	2	0.85	-2.06	0.00	0.00
PTER	9317	1.38	0.95	1.55	2.13	-0.61	0.00	0.00
PTGDR2	11251	1.48	1.19	2.04	3.05	-0.93	0.00	0.00
PTGER1	5731	0	0.06	0.76	0.34	-4.23	0.00	0.00
PTGER3	5733	0	0	0	0	-3.15	0.00	0.00
PTGES3L	100885848	0.14	0.56	0.42	1.11	-1.19	0.00	0.00
PTGES3L-AARSD1	100885850	2.56	2.26	3.96	3.42	-0.68	0.00	0.00
PTGS1	5742	0.01	0.01	0.02	0.03	-3.78	0.00	0.00
PTP4A3	11156	0	0	8.36	1.31	-9.96	0.00	0.00

PTPRN2	5799	0.16	0.18	2.93	3.76	-4.71	0.00	0.00
PTPRS	5802	7.22	6.34	17.22	11.2	-0.99	0.00	0.00
PTS	5805	6.12	6.18	12.22	12.2	-0.96	0.00	0.00
PYY	5697	0.06	0	0.68	0.16	-3.00	0.00	0.00
RAB25	57111	0.47	0.1	10.5	9.5	-5.13	0.00	0.00
RAB31	11031	0.43	0.14	3.06	2.74	-3.55	0.00	0.00
RAB34	83871	0.24	0.25	41.37	39.15	-6.09	0.00	0.00
RAB3A	5864	1.56	1.88	4.81	3.86	-1.33	0.00	0.00
RAB3B	5865	0.11	0.08	1.07	1.05	-3.31	0.00	0.00
RAB3IL1	5866	0.39	0.6	1.48	1.01	-1.35	0.00	0.00
RAC2	5880	0.19	0.09	0.95	0.63	-2.54	0.00	0.00
RAD21-AS1	644660	0	0.26	0	0.82	-1.74	0.00	0.00
RAMP2	10266	0.86	1.33	7.58	4.32	-2.57	0.00	0.00
RAMP2-AS1	100190938	0.04	0.1	0.54	0.29	-3.31	0.00	0.00
RANBP10	57610	5.03	5.27	7.31	8.06	-0.73	0.00	0.00
RANBP3L	202151	0	0	0.05	0.14	-5.12	0.00	0.00
RARG	5916	0.43	0.15	1.27	0.72	-1.73	0.00	0.00
RASA4B	100271927	0.92	0.5	0.77	1.03	-0.59	0.00	0.00
RASD1	51655	2.46	2.63	12.64	6.35	-1.90	0.00	0.00
RASGRF2	5924	2.13	1.64	2.54	2.52	-0.63	0.00	0.00
RASGRF2-AS1	102524628	1.05	1.61	3.16	2.64	-1.05	0.00	0.00
RASL10B	91608	7.52	7.64	16.72	16.37	-1.11	0.00	0.00
RBM39	9584	30.1	30.42	17.5	21.02	-0.67	0.00	0.00
RBMS2	5939	1.65	1.74	4.78	3.25	-1.22	0.00	0.00
RBP1	5947	1.88	2.03	21.17	8.29	-2.92	0.00	0.00
RBPMS	11030	34.64	34.38	63.54	52.8	-0.73	0.00	0.00
REEP2	51308	9.59	8.6	20.62	12.97	-0.88	0.00	0.00
RELL2	285613	3.38	3.24	10.25	6.65	-1.29	0.00	0.00
RENBP	5973	1.42	1.77	8.6	7.18	-2.37	0.00	0.00
REXO1	57455	9.43	9.36	15.22	13.72	-0.62	0.00	0.00
RGL1	23179	1.13	1.14	2.41	2.02	-1.08	0.00	0.00
RGL2	5863	134.27	154.2	129.17	120.84	-3.04	0.00	0.00
RGS10	6001	8.13	6.92	21.66	13.97	-1.23	0.00	0.00

RGS14	10636	1.77	2.08	3.07	3.5	-0.70	0.00	0.00
RHOC	389	78.14	77.32	164.36	142.14	-0.97	0.00	0.00
RHOG	391	16.35	17.9	41.77	40.08	-1.26	0.00	0.00
RIMBP3C	150221	0	0	0.21	0.04	-5.77	0.00	0.00
RIMKLA	284716	0.03	0.03	0.31	0.25	-3.25	0.00	0.00
RIN1	9610	14.07	12.85	22.03	27.57	-0.87	0.00	0.00
RIPOR3	140876	0.35	0.42	1.15	1.84	-1.75	0.00	0.00
RIPPLY3	53820	0.02	0.03	0.59	0.31	-4.11	0.00	0.00
RMC1	29919	4.75	4.12	7.6	7.44	-0.60	0.00	0.00
RMDN2-AS1	101410544	0	0.02	0	0	-6.31	0.00	0.00
RNASEK	440400	169.48	161.95	248.31	250.41	-0.59	0.00	0.00
RNF144A	9781	5.72	6.62	3.59	3.81	-0.62	0.00	0.00
RPH3AL	9501	4.21	4.52	10.62	8.43	-1.13	0.00	0.00
RPLP0P2	113157	0.11	0.08	0.34	0.21	-1.85	0.00	0.00
RRAD	6236	0.54	0.31	1.79	0.63	-1.52	0.00	0.00
RSPO4	343637	0	0	0.02	0	-1.42	0.00	0.00
RTEL1	51750	1.61	2.17	2.69	3.1	-0.59	0.00	0.00
RTL8C	8933	0	0	19.46	16.06	-10.57	0.00	0.00
RUNX3	864	3.97	0.76	3.06	6.46	-1.01	0.00	0.00
S100A10	6281	67.29	70.43	109.15	115.41	-0.70	0.00	0.00
S100A11	6282	43.93	37.39	164.23	122.09	-1.82	0.00	0.00
S100A2	6273	1.14	0.93	3.81	1.53	-1.50	0.00	0.00
S100A6	6277	65.37	67.62	148.14	134.82	-1.09	0.00	0.00
S100P	6286	81.95	93.18	236.76	161.91	-1.19	0.00	0.00
SAA2-SAA4	100528017	0.2	0.32	1.44	2.64	-3.13	0.00	0.00
SAA4	6291	27.13	29.8	121.26	284.03	-2.83	0.00	0.00
SASH3	54440	0.09	0.06	0.16	0.15	-1.17	0.00	0.00
SATB2-AS1	150538	0	0	0.11	0.5	-4.94	0.00	0.00
SCAMP4	113178	29.72	27.02	47.05	42.71	-0.66	0.00	0.00
SCARA3	51435	0.01	0	0.27	1.53	-6.89	0.00	0.00
SCML1	6322	1.07	1.37	2.77	4.06	-1.41	0.00	0.00
SCN1A	6323	0.17	0.16	0.56	0.62	-1.91	0.00	0.00
SCN2B	6327	0	0	0.12	0.18	-5.78	0.00	0.00

SCN9A	6335	0.27	0.28	0.63	0.71	-1.10	0.00	0.00
SCNN1A	6337	10.96	10.17	20.33	15.46	-0.73	0.00	0.00
SDC1	6382	64.23	65.02	111	96.88	-0.68	0.00	0.00
SDC3	9672	0.11	0.17	0.77	0.26	-1.89	0.00	0.00
SELENOM	140606	2.53	3.38	18.25	13.24	-2.39	0.00	0.00
SELENOW	6415	19.33	19.81	32.52	28.03	-0.63	0.00	0.00
SEMA3A	10371	0.25	0.22	0.68	1.11	-1.80	0.00	0.00
SEMA3F	6405	18.92	19.07	29.65	28.82	-0.63	0.00	0.00
SEMA6B	10501	88.32	83.62	142.31	115.31	-0.59	0.00	0.00
SERHL2	253190	0	0	0.35	0.36	-5.08	0.00	0.00
SERPINA2	390502	0.17	0.17	1	1.81	-3.05	0.00	0.00
SERPINB8	5271	1.12	1.51	1.48	2.9	-0.74	0.00	0.00
SERPINB9	5272	0.95	0.57	2.15	1.63	-1.29	0.00	0.00
SERPINE2	5270	0.4	0.42	5.72	3.63	-3.50	0.00	0.00
SERPINH1	871	39.94	40.9	138.68	72.89	-1.36	0.00	0.00
SERTAD1	29950	3.17	2.54	3.91	5.5	-0.72	0.00	0.00
SESN2	83667	8.63	8.54	15.86	11.29	-0.65	0.00	0.00
SETD7	80854	0.17	0.01	0.21	0.13	-1.97	0.00	0.00
SFI1	9814	4.74	4.98	9.86	6.4	-0.76	0.00	0.00
SGK1	6446	1.38	1.48	5.09	4.5	-1.75	0.00	0.00
SGK2	10110	17.83	16.3	21.15	34.66	-0.71	0.00	0.00
SGMS2	166929	0	0.02	0.04	0.11	-3.11	0.00	0.00
SH2D2A	9047	3.82	2.88	6.59	3.4	-0.72	0.00	0.00
SH2D5	400745	2.31	2.21	5.06	5.12	-1.17	0.00	0.00
SH3TC1	54436	2.64	2.59	10.5	8.34	-1.87	0.00	0.00
SHB	6461	10.55	10.4	18.52	16.76	-0.63	0.00	0.00
SHC2	25759	4.49	4.59	13.29	6.64	-1.14	0.00	0.00
SHISA4	149345	0.27	0.4	2.45	0.51	-2.20	0.00	0.00
SIGLEC5	8778	0	0	0	0	-4.49	0.00	0.00
SIK1	150094	0.65	0.62	1.47	1.16	-1.06	0.00	0.00
SIM2	6493	0.4	0.36	1.11	1.42	-1.36	0.00	0.00
SIMC1	375484	0.16	0.26	2.12	1.61	-2.65	0.00	0.00
SKA1	220134	3.93	4.48	5.88	7.25	-0.65	0.00	0.00

SLC12A4	6560	8.78	8.86	16.34	12.6	-0.76	0.00	0.00
SLC16A13	201232	15.98	15.37	24.08	24.55	-0.61	0.00	0.00
SLC16A5	9121	9.84	10.77	19.19	13.91	-0.65	0.00	0.00
SLC16A6	9120	0.12	0.27	0.52	1.2	-2.30	0.00	0.00
SLC17A2	10246	5.2	5.76	8.69	7.62	-0.59	0.00	0.00
SLC1A2	6506	0.64	0.66	1.31	1.48	-1.01	0.00	0.00
SLC1A7	6512	1.06	1.39	13.36	8.77	-3.14	0.00	0.00
SLC20A1	6574	26.47	29.61	39.53	46.02	-0.60	0.00	0.00
SLC22A9	114571	0.33	0.47	2.2	2.78	-2.64	0.00	0.00
SLC25A18	83733	0.65	0.31	1.02	1.42	-1.20	0.00	0.00
SLC25A27	9481	0.02	0.01	0.38	0.49	-5.45	0.00	0.00
SLC25A42	284439	9.06	9.25	12.89	18.73	-0.78	0.00	0.00
SLC25A47	283600	11.7	12.01	15.81	21.36	-0.65	0.00	0.00
SLC26A9	115019	0.33	0.17	0.36	0.87	-1.36	0.00	0.00
SLC29A3	55315	16.25	16.48	34.61	35.8	-1.07	0.00	0.00
SLC2A1	6513	49.68	50.31	103.28	80.23	-0.88	0.00	0.00
SLC2A11	66035	4.44	4.1	6.79	8.16	-0.72	0.00	0.00
SLC2A5	6518	0.01	0.05	0.14	0.47	-2.75	0.00	0.00
SLC35E1	79939	14.47	14.72	21.98	22.96	-0.59	0.00	0.00
SLC35G2	80723	0.79	1	2.41	1.76	-2.16	0.00	0.00
SLC37A2	219855	2.62	1.87	14.39	12.58	-2.57	0.00	0.00
SLC38A1	81539	4.07	3.84	5.8	8.09	-0.74	0.00	0.00
SLC38A2	54407	10.03	9.31	21.07	22.79	-1.18	0.00	0.00
SLC38A4	55089	0.01	0	0.12	0.1	-3.11	0.00	0.00
SLC38A7	55238	7.32	7.54	11.65	11.85	-0.65	0.00	0.00
SLC39A8	64116	0.04	0.04	0.06	0.36	-3.42	0.00	0.00
SLC40A1	30061	6.25	6.68	10.28	20.21	-1.24	0.00	0.00
SLC41A1	254428	0	0.01	0.1	0.06	-3.83	0.00	0.00
SLC43A2	124935	12.85	11.72	19.69	23.37	-0.86	0.00	0.00
SLC4A10	57282	0	0.03	0.04	0.09	-2.38	0.00	0.00
SLC51B	123264	4.58	4.11	5.04	10.28	-0.82	0.00	0.00
SLC52A2	79581	31.99	33.3	50.87	50.64	-0.60	0.00	0.00
SLC6A12	6539	4.18	3.64	4.36	10.3	-0.95	0.00	0.00

SLC6A4	6532	3.7	3.49	4.8	6.05	-0.59	0.00	0.00
SLC7A10	56301	2.28	1.89	7.81	6.16	-1.74	0.00	0.00
SLC9A5	6553	0.32	0.33	1.43	0.72	-1.58	0.00	0.00
SLC9B1	150159	0.29	0	0.87	0.33	-2.07	0.00	0.00
SLCO4A1	28231	7.36	8.44	31.2	27.18	-1.85	0.00	0.00
SLIT2	9353	0	0.01	0	0	-2.67	0.00	0.00
SMARCD3	6604	0.09	0	0.96	0.44	-4.48	0.00	0.00
SMIM10	644538	0.04	0.04	0.42	0.53	-3.67	0.00	0.00
SMIM10L2B	644596	0	0.02	0.18	0.16	-4.11	0.00	0.00
SMIM3	85027	1.06	0.73	6.62	4.68	-2.62	0.00	0.00
SMPD1	6609	4.05	3.92	9.29	9.69	-1.25	0.00	0.00
SMPD5	392275	0	0.02	0	0	-3.28	0.00	0.00
SNAI1	6615	1.42	1.54	7.23	4.16	-1.92	0.00	0.00
SNHG5	387066	0.34	0.44	0.33	0.31	-0.79	0.00	0.00
SNX29	92017	1.76	1.9	2.51	2.99	-0.62	0.00	0.00
SOBP	55084	0.23	0.2	1.53	0.94	-2.65	0.00	0.00
SOCS2	8835	0.45	0.81	1.14	1.25	-1.00	0.00	0.00
SOCS3	9021	0.06	0.12	3.03	0.81	-4.42	0.00	0.00
SOGA1	140710	3.45	3.13	5.83	6.24	-0.77	0.00	0.00
SORCS3	22986	0	0	0	0	-0.60	0.00	0.00
SOX4	6659	0.58	0.51	9.39	7.12	-3.92	0.00	0.00
SP6	80320	0.04	0.16	0.29	0.33	-1.64	0.00	0.00
SPARC	6678	0.58	0.83	5.67	1.96	-2.78	0.00	0.00
SPINK1	6690	0.22	0.11	5.73	6.79	-3.98	0.00	0.00
SPINT2	10653	0.85	1.18	4.9	3.55	-1.92	0.00	0.00
SPNS3	201305	0.04	0.03	0.39	0.36	-3.00	0.00	0.00
SPOCK2	9806	16.67	15.84	28.91	46.98	-1.23	0.00	0.00
SPRED3	399473	0.03	0.07	0.38	0.09	-2.29	0.00	0.00
SPRY1	10252	3.29	3.7	6.04	6.01	-0.87	0.00	0.00
SPRY2	10253	3.14	3.27	5.44	6.03	-0.81	0.00	0.00
SPRY4	81848	4.24	4.51	9.74	9.52	-1.14	0.00	0.00
SPTBN1	6711	48.8	50.93	35.49	37.48	-2.13	0.00	0.00
SPTBN2	6712	1.74	1.65	2.59	2.55	-2.33	0.00	0.00

SPTSSB	165679	0	0.07	0.77	2.06	-5.36	0.00	0.00
SRARP	149563	44.33	42.45	73.87	74.32	-0.77	0.00	0.00
SRC	6714	31.53	31.55	59.86	52.4	-0.83	0.00	0.00
SRPX	8406	0	0	0.25	0.18	-4.72	0.00	0.00
SRPX2	27286	0.05	0.25	8.06	6.35	-0.68	0.00	0.00
SSBP3	23648	11.79	13.15	11.62	11.68	-0.73	0.00	0.00
SSTR3	6753	0.4	0.46	0.65	1.85	-1.56	0.00	0.00
ST3GAL2	6483	7.01	6.17	14.17	10.12	-0.85	0.00	0.00
ST3GAL5	8869	0.05	0.06	0.68	0.57	-3.30	0.00	0.00
STAM	8027	10.37	10.67	15.53	22.28	-0.84	0.00	0.00
STAR	6770	5.91	6.28	10.98	8.62	-0.65	0.00	0.00
STARD3NL	83930	4.14	6.15	11.8	12.34	-1.24	0.00	0.00
STAT5A	6776	2.03	1.46	2.23	2.36	-0.65	0.00	0.00
STBD1	8987	2.5	2.06	4.68	4.1	-0.97	0.00	0.00
STEAP1	26872	3.55	3.28	6.35	10.66	-1.31	0.00	0.00
STEAP2	261729	0.34	0.37	1.16	0.9	-1.53	0.00	0.00
STEAP3-AS1	100874111	0	0.06	0	0	-0.61	0.00	0.00
STIM1	6786	10.01	9.7	19.22	18.92	-0.95	0.00	0.00
STK10	6793	3.02	2.92	4.82	4.95	-0.65	0.00	0.00
STK24	8428	46.09	39.6	74.68	70.62	-0.76	0.00	0.00
STK26	51765	3.28	4.46	4.34	6.99	-0.64	0.00	0.00
STK33	65975	0.09	0.09	0.65	0.67	-2.83	0.00	0.00
STK4-AS1	100505826	0.08	0.09	0.2	0.2	-1.22	0.00	0.00
STMN3	50861	3.99	3.35	21.19	14.16	-2.29	0.00	0.00
STN1	79991	5.08	6.1	11.58	7.88	-0.62	0.00	0.00
STOM	2040	0	0	0.55	0	-6.02	0.00	0.00
STON2	85439	0.05	0.09	0.3	0.43	-2.27	0.00	0.00
STPG3	441476	0.3	0.39	2.09	0.4	-1.71	0.00	0.00
SULF2	55959	0.42	0.55	1.8	1.19	-1.64	0.00	0.00
SULT1C2	6819	0.57	0.46	1.58	2.58	-2.10	0.00	0.00
SULT1C4	27233	0.02	0	1.28	1.01	-6.83	0.00	0.00
SV2A	9900	0.22	0.14	3.25	1.7	-3.66	0.00	0.00
SYBU	55638	3.76	3.5	4.83	6.6	-0.66	0.00	0.00

SYDE1	85360	1.35	1.3	5.75	3.69	-1.65	0.00	0.00
SYN3	8224	0.44	0.67	1.06	1.31	-0.83	0.00	0.00
SYNGR3	9143	0.95	1.2	4.63	2.14	-1.65	0.00	0.00
SYNGR4	23546	0.06	0.31	1.27	0.8	-2.39	0.00	0.00
SYNPO	11346	0.17	0.08	1.48	2.02	-3.87	0.00	0.00
SYP	6855	0.29	0.38	3.93	3.61	-3.48	0.00	0.00
SYPL2	284612	0.06	0.09	0.36	0.4	-2.29	0.00	0.00
SYT11	23208	0.03	0.01	0.25	0.04	-2.57	0.00	0.00
SYT7	9066	0.81	0.65	2.01	0.93	-1.09	0.00	0.00
SYTL2	54843	0.19	0.13	1.89	2.18	-3.74	0.00	0.00
SYTL4	94121	0.03	0.04	0.29	0.41	-3.48	0.00	0.00
TAC3	6866	0.19	0.08	0.24	0.46	-1.81	0.00	0.00
TACC2	10579	9.22	9.23	14.77	16.55	-0.84	0.00	0.00
TACR2	6865	0.14	0.16	3.15	2.76	-4.49	0.00	0.00
TAT	6898	1.03	1.48	3.02	1.86	-0.96	0.00	0.00
TBL1Y	90665	0	0	0.08	0.21	-5.88	0.00	0.00
TBX2	6909	4.73	3.81	6.7	10.82	-1.04	0.00	0.00
TBX3	6926	18.9	19.05	39.95	28.88	-0.85	0.00	0.00
TBXA2R	6915	0	0.06	0.34	0.03	-3.22	0.00	0.00
TCF3	6929	32.74	29.41	52.54	44.88	-0.63	0.00	0.00
TCP10L	140290	0	0.02	0.41	0.41	-5.58	0.00	0.00
TCP11L2	255394	1.48	1.75	3.46	1.9	-0.64	0.00	0.00
TECTB	6975	0.55	0.8	1.79	1.6	-1.33	0.00	0.00
TENM3	55714	0.1	0	0.42	0.63	-4.67	0.00	0.00
TEP1	7011	1.02	1.08	1.6	1.71	-0.59	0.00	0.00
TEX30	93081	0.16	0.09	0.78	0.86	-2.72	0.00	0.00
TEX50	730159	0	0	0	0	-2.83	0.00	0.00
TGFB3	7043	0.23	0.32	0.93	0.54	-1.32	0.00	0.00
TGFBR3L	100507588	1.11	0.72	2.51	1.96	-1.30	0.00	0.00
TGM2	7052	10.84	10.95	30.67	14.89	-1.06	0.00	0.00
TGM5	9333	0.03	0.06	0.49	0.23	-3.08	0.00	0.00
THBS1	7057	3.12	3.07	5.82	4.92	-0.80	0.00	0.00
THBS3	7059	0.37	0.63	2.06	0.85	-1.15	0.00	0.00

TIAF1	9220	0.62	1.22	6.19	3.33	-2.37	0.00	0.00
TIE1	7075	0.04	0.08	0.31	0.13	-1.81	0.00	0.00
TIGAR	57103	5.41	5.49	9.47	9.41	-0.72	0.00	0.00
TIMP1	7076	63.55	60.09	215.62	178.52	-1.67	0.00	0.00
TIMP3	7078	0.13	0.03	5.23	7.09	-6.07	0.00	0.00
TIMP4	7079	0.32	0.33	2.13	1.21	-2.67	0.00	0.00
TLE6	79816	0.39	0.41	1.7	0.47	-1.39	0.00	0.00
TLNRD1	59274	10.14	8.87	16.2	12.93	-0.58	0.00	0.00
TLX1	3195	0.16	0.26	0.4	0.77	-1.48	0.00	0.00
TM4SF19	116211	0.38	2.04	5.31	2.63	-1.75	0.00	0.00
TM4SF19-TCTEX1D2	100534611	2.19	2.07	5.08	3.12	-1.09	0.00	0.00
TMC6	11322	17.24	17.24	40.3	29.3	-0.88	0.00	0.00
TMC8	147138	0.99	1.32	4.49	3.97	-1.91	0.00	0.00
TMEM110-MUSTN1	100526772	0.47	0.34	1.18	1.85	-1.02	0.00	0.00
TMEM150B	284417	2.42	2.58	5.86	5.17	-1.10	0.00	0.00
TMEM151A	256472	0.72	0.6	1.79	1.91	-1.49	0.00	0.00
TMEM169	92691	0.79	0.86	1.55	1.24	-0.76	0.00	0.00
TMEM173	340061	0.42	0.67	1.17	1.17	-1.15	0.00	0.00
TMEM185B	79134	3.44	3.35	8.03	6.46	-1.09	0.00	0.00
TMEM198	130612	8.04	8.26	20.98	30.44	-1.28	0.00	0.00
TMEM231	79583	1.54	1.74	5.19	3.48	-1.24	0.00	0.00
TMEM268	203197	2.15	2.29	4.34	2.72	-0.67	0.00	0.00
TMEM54	113452	0.43	0.22	3.68	2.09	-3.27	0.00	0.00
TMEM59L	25789	2.54	2.88	47.34	13.41	-3.50	0.00	0.00
TMEM63C	57156	0.11	0.16	1.14	1.16	-3.05	0.00	0.00
TMEM9	252839	24.71	27.25	50.37	31.18	-0.65	0.00	0.00
TMEM91	641649	10.81	9.23	15.73	10.79	-0.58	0.00	0.00
TMIGD2	126259	0.73	1.49	5.07	2.98	-1.91	0.00	0.00
TMPRSS9	360200	3.97	4.16	11.71	6.33	-1.24	0.00	0.00
TMSB10	9168	600.38	604.03	1511.33	1177.55	-1.16	0.00	0.00
TNFRSF11B	4982	0	0	0	0	-5.68	0.00	0.00
TNFSF12	8742	3.28	4.05	9.39	4.87	-0.96	0.00	0.00
TNFSF4	7292	2.26	2.32	4.08	4.46	-0.91	0.00	0.00

TNFSF9	8744	1.66	1.88	5.81	3.18	-1.36	0.00	0.00
TNNC1	7134	11.63	13.44	23.21	14.56	-0.59	0.00	0.00
TNS4	84951	0.04	0.16	0.87	0.47	-3.68	0.00	0.00
TNXA	7146	0.35	0.26	0.38	0.16	-1.53	0.00	0.00
TNXB	7148	0.12	0.22	1.57	0.64	-2.57	0.00	0.00
TP53INP2	58476	12.78	12.1	20.16	31.66	-1.06	0.00	0.00
TPM2	7169	0.64	0.45	2.48	1.26	-1.72	0.00	0.00
TRIB1	10221	18.02	16.96	32.52	26.81	-0.73	0.00	0.00
TRIB2	28951	0	0	0.92	0.32	-7.05	0.00	0.00
TRIM29	23650	0.18	0.15	0.79	0.64	-1.51	0.00	0.00
TRIM54	57159	0.28	0.29	1.31	0.64	-1.28	0.00	0.00
TRIP10	9322	21.32	21.98	41.2	25.89	-0.65	0.00	0.00
TRIQK	286144	1.27	1.63	1.75	3.72	-0.85	0.00	0.00
TRMT1	55621	21.55	20.94	42.12	38.28	-0.90	0.00	0.00
TROVE2	6738	0.38	0.25	0.35	0.85	-0.90	0.00	0.00
TRPM4	54795	2.79	3.16	4.64	4.47	-0.70	0.00	0.00
TRPS1	7227	0	0	0	0	-0.60	0.00	0.00
TRPV1	7442	2.14	2.2	1.38	2.2	-6.23	0.00	0.00
TRPV2	51393	4.72	5.1	15.54	14.57	-1.61	0.00	0.00
TSC22D3	1831	0.06	0.16	1.87	0.6	-3.59	0.00	0.00
TSFM	10102	16.82	16.08	16.47	26.69	-0.87	0.00	0.00
TSKS	60385	0.5	0.16	1.27	1.04	-2.70	0.00	0.00
TSPAN14	81619	15.73	15.96	26.58	25.72	-0.73	0.00	0.00
TSPAN4	7106	36.96	34.19	57.31	53.82	-0.62	0.00	0.00
TSPAN7	7102	0.15	0.09	50.57	39.27	-8.40	0.00	0.00
TSPOAP1-AS1	100506779	0.04	0.19	0.45	0.25	-2.54	0.00	0.00
TSSK6	83983	1.39	1.67	2.21	3.05	-0.76	0.00	0.00
TTBK1	84630	1.72	1.64	3	2.84	-0.83	0.00	0.00
TTC25	83538	0.13	0.23	0.32	0.23	-1.10	0.00	0.00
TTC9	23508	0.55	0.39	1.64	1.97	-1.94	0.00	0.00
TTPA	7274	0.08	0.16	1.12	1.82	-3.72	0.00	0.00
TTR	7276	51.31	52.87	59.72	184.42	-1.23	0.00	0.00
TUB	7275	0	0	0.1	0.08	-5.40	0.00	0.00

TUBA4A	7277	6.17	6.37	13.85	6.85	-0.66	0.00	0.00
TUBA4B	80086	0.74	0.25	1	1.58	-1.39	0.00	0.00
TUBB2A	7280	2.98	5.16	19.62	24.47	-2.43	0.00	0.00
TUBB2B	347733	8.71	8.59	46.56	41.96	-2.30	0.00	0.00
TUBB3	10381	71.43	69.51	151.05	125.49	-0.97	0.00	0.00
TUBB4A	10382	0	0	0.28	0.05	-4.83	0.00	0.00
TUBB6	84617	2.87	2.34	17.36	9.47	-2.05	0.00	0.00
TUT1	64852	1.08	3.86	6.7	1.36	-0.70	0.00	0.00
TXNIP	10628	13.89	13.41	38.65	22.44	-1.14	0.00	0.00
U2AF1L4	199746	2.01	2.58	5.32	3.52	-1.01	0.00	0.00
UBALD2	283991	34.56	30.92	68.49	47.46	-0.84	0.00	0.00
UBASH3B	84959	1.38	1.56	2.03	2.5	-0.61	0.00	0.00
UBL5	59286	76.44	86.05	131.92	133.6	-0.72	0.00	0.00
UBTD1	80019	6.93	7.5	13.08	14.28	-0.92	0.00	0.00
UCA1	652995	0.16	0.08	2.37	2.08	-4.26	0.00	0.00
UGT1A3	54659	0.11	0.3	0.57	0.99	-1.94	0.00	0.00
UGT1A7	54577	0	0	0.3	0.14	-5.23	0.00	0.00
UGT2B11	10720	0.04	0	0.15	0.64	-4.52	0.00	0.00
UGT2B4	7363	0.16	0.09	3.2	9.54	-5.73	0.00	0.00
UGT2B7	7364	14.37	16.37	17.2	22.95	-1.00	0.00	0.00
ULK1	8408	3.29	2.23	5.73	4.58	-0.82	0.00	0.00
UNC5B	219699	0.03	0.03	0.55	0.09	-3.99	0.00	0.00
UPP1	7378	3.76	3.99	8.17	5.34	-0.75	0.00	0.00
UROD	7389	59.48	58.72	88.09	94.42	-0.62	0.00	0.00
USE1	55850	13.71	14.23	24.4	20.92	-0.70	0.00	0.00
USH1G	124590	0.11	0.09	0.62	0.43	-2.41	0.00	0.00
USHBP1	83878	0.4	0.45	1.32	0.6	-1.17	0.00	0.00
USP35	57558	1.32	1.4	2.37	2.38	-0.78	0.00	0.00
USP50	373509	0	0.01	0.26	0.09	-3.79	0.00	0.00
VAC14	55697	16.47	15.35	29.72	28.21	-0.77	0.00	0.00
VAT1	10493	108.12	102.11	200.13	193.58	-0.90	0.00	0.00
VAX2	25806	0.43	0.31	2.42	3.01	-3.09	0.00	0.00
VLDLR	7436	0.37	0.24	2.53	2.6	-3.26	0.00	0.00

VLDLR-AS1	401491	0	0	0	0	-0.77	0.00	0.00
VNN1	8876	0.09	0.06	0.95	1.37	-3.84	0.00	0.00
VPS35L	57020	0.03	0.09	0.4	1.18	-3.88	0.00	0.00
VSIR	64115	0.13	0.15	0.5	0.78	-2.37	0.00	0.00
VWA7	80737	4.37	3.59	4.43	5.31	-0.59	0.00	0.00
VWF	7450	0	0.01	0.04	0.08	-3.35	0.00	0.00
WARS	7453	35.82	33.99	54.08	51.09	-0.59	0.00	0.00
WDR35	57539	0.92	0.8	1.11	1.49	-0.58	0.00	0.00
WFIKKN1	117166	3.54	2.82	6.83	4.8	-0.74	0.00	0.00
WIPI1	55062	14.55	13.77	33.79	35.74	-1.21	0.00	0.00
WNT11	7481	0.21	0.14	1.31	0.75	-1.95	0.00	0.00
WNT3	7473	0.92	1	10.31	8.1	-3.27	0.00	0.00
WNT7B	7477	0.08	0.1	1.85	0.51	-3.83	0.00	0.00
WNT9B	7484	0.03	0.02	5.11	3.92	-7.40	0.00	0.00
WSCD1	23302	0.05	0	0.22	0.22	-3.06	0.00	0.00
XIRP2	129446	0.02	0.05	0.21	0.21	-2.67	0.00	0.00
YOD1	55432	0	0.35	0.22	0.37	-1.28	0.00	0.00
YPEL3	83719	18.91	15.9	31.48	21.69	-0.60	0.00	0.00
YRDC	79693	8.31	7.52	18.74	16.88	-1.17	0.00	0.00
ZAP70	7535	0.83	0.79	3.25	2.09	-1.91	0.00	0.00
ZAR1L	646799	0	0.11	0	0	-0.59	0.00	0.00
ZBTB46	140685	0.3	0.32	3.08	2.01	-3.11	0.00	0.00
ZC3H8	84524	1.97	1.54	0.76	1.77	-1.25	0.00	0.00
ZCCHC24	219654	0.11	0.04	0.29	0.35	-2.22	0.00	0.00
ZDHHC14	79683	2.2	1.82	3.83	3.32	-0.68	0.00	0.00
ZDHHC19	131540	0.26	0	1.17	0.32	-2.56	0.00	0.00
ZEB1	6935	0.11	0.08	0.27	0.26	-1.41	0.00	0.00
ZFP36	7538	9.15	8.27	22.12	17.01	-1.17	0.00	0.00
ZFP36L1	677	14.31	14.12	23.41	21.34	-0.63	0.00	0.00
ZHX1	11244	0.14	0	0.29	0.56	-1.56	0.00	0.00
ZIC2	7546	2.42	2.77	8.63	7.59	-1.64	0.00	0.00
ZMIZ1-AS1	283050	0.34	0.4	1.36	0.65	-1.28	0.00	0.00
ZNF234	10780	0.45	0.52	0.25	0.15	-3.54	0.00	0.00

ZNF497	162968	0.68	0.48	1.2	0.92	-0.88	0.00	0.00
ZNF66	7617	0.02	0	0.02	0.05	-3.04	0.00	0.00
ZNF704	619279	0.01	0.02	0.24	0.3	-4.05	0.00	0.00
ZNF737	100129842	0.37	0.24	0.42	0.51	-4.68	0.00	0.00
ZNF8	7554	0	0	0.41	0.23	-5.67	0.00	0.00
ZNF862	643641	0.06	0.22	0.59	0.21	-1.19	0.00	0.00
ZNHIT1	10467	61.92	63.28	71.96	64.87	-1.03	0.00	0.00
ZNRD1ASP	80862	0.06	0.05	0.21	0.19	-1.91	0.00	0.00
ZNRF1	84937	9.82	12.12	23.98	17.6	-0.81	0.00	0.00
ZP1	22917	0.04	0.25	1.08	0.39	-2.30	0.00	0.00
ZSCAN12	9753	0.64	0.63	0.55	0.42	-2.86	0.00	0.00
ZSWIM5	57643	5.64	4.56	8.24	8.54	-0.63	0.00	0.00

Table S2. Prediction of potential candidate target genes of miR-23a-3p and miR-27a-3p using TargetScan analysis

Target gene	Representative transcript	Gene name	3P-seq tags + 5	Total sites	8mer sites	7mer-m8 sites	7mer-A1 sites	6mer sites	Representative miRNA	Cumulative weighted context ++ score	Total context ++ score	Aggregate PCT
ZNF286B	ENST00000285274.5	zinc finger protein 286B	5	3	2	1	0	2	hsa-miR-23a-3p	-0.89	-0.89	N/A
KIAA1467	ENST00000197268.8	KIAA1467	509	3	3	0	0	0	hsa-miR-23a-3p	-0.83	-0.83	N/A
PKP4	ENST00000389757.3	plakophilin 4	373	2	2	0	0	0	hsa-miR-23a-3p	-0.79	-0.79	N/A
ZNF667	ENST00000591790.1	zinc finger protein 667	24	1	0	1	0	1	hsa-miR-23a-3p	-0.78	-0.78	N/A
ZNF287	ENST00000395824.1	zinc finger protein 287	14	1	1	0	0	2	hsa-miR-23a-3p	-0.68	-0.82	N/A
SNRPC	ENST00000244520.5	small nuclear ribonucleoprotein polypeptide C	60	1	1	0	0	0	hsa-miR-23a-3p	-0.68	-0.68	N/A
PDE4B	ENST00000371045.5	phosphodiesterase 4B, cAMP-specific	127	3	3	0	0	0	hsa-miR-23a-3p	-0.67	-0.69	N/A
PDE7A	ENST00000401827.3	phosphodiesterase 7A	84	4	2	1	1	0	hsa-miR-23a-3p	-0.65	-0.68	N/A
TOP1	ENST00000361337.2	topoisomerase (DNA) I	15	2	2	0	0	0	hsa-miR-23a-3p	-0.6	-0.6	N/A
RALYL	ENST00000517638.1	RALY RNA binding protein-like	7	2	2	0	0	1	hsa-miR-23a-3p	-0.6	-0.6	N/A
PROK2	ENST00000353065.3	prokineticin 2	7	1	1	0	0	0	hsa-miR-23a-3p	-0.58	-0.58	N/A
IGSF8	ENST00000368086.1	immunoglobulin superfamily, member 8	92	1	1	0	0	0	hsa-miR-23a-3p	-0.54	-0.55	N/A
BORA	ENST00000390667.5	bora, aurora kinase A activator	244	1	1	0	0	0	hsa-miR-23a-3p	-0.54	-0.7	N/A
NDUFC1	ENST00000394228.1	NADH dehydrogenase (ubiquinone) 1, subcomplex unknown, 1, 6kDa	84	1	0	0	1	1	hsa-miR-23a-3p	-0.54	-0.62	N/A
MYH1	ENST00000226207.5	myosin, heavy chain 1, skeletal muscle, adult	5	1	1	0	0	0	hsa-miR-23a-3p	-0.54	-0.54	N/A
CCL7	ENST00000378569.2	chemokine (C-C motif) ligand 7	5	1	1	0	0	0	hsa-miR-23a-3p	-0.53	-0.53	N/A
SATB1	ENST00000338745.6	SATB homeobox 1	312	2	2	0	0	0	hsa-miR-23a-3p	-0.51	-0.66	N/A
VRK3	ENST00000316763.3	vaccinia related kinase 3	366	2	1	1	0	0	hsa-miR-23a-3p	-0.51	-0.51	N/A
C6orf62	ENST00000378119.4	chromosome 6 open reading frame 62	300	1	1	0	0	0	hsa-miR-23a-3p	-0.51	-0.51	N/A
NAP1L5	ENST00000323061.5	nucleosome assembly protein 1-like 5	28	2	1	1	0	0	hsa-miR-23a-3p	-0.51	-0.55	N/A
MCFD2	ENST00000444761.2	multiple coagulation factor deficiency 2	1648	2	0	2	0	1	hsa-miR-23a-3p	-0.5	-0.5	N/A
SYT4	ENST00000255224.3	synaptotagmin IV	11	1	1	0	0	1	hsa-miR-23a-3p	-0.5	-0.5	N/A
PKIA	ENST00000396418.2	protein kinase (cAMP-dependent, catalytic) inhibitor alpha	542	1	1	0	0	2	hsa-miR-23a-3p	-0.5	-0.65	N/A
HMGB2	ENST00000296503.5	high mobility group box 2	8626	1	1	0	0	0	hsa-miR-23a-3p	-0.5	-0.71	N/A
C2orf69	ENST00000319974.5	chromosome 2 open reading frame 69	82	2	1	1	0	0	hsa-miR-23a-3p	-0.49	-0.52	N/A
STX12	ENST00000373943.4	syntaxin 12	1082	2	2	0	0	1	hsa-miR-23a-3p	-0.49	-0.52	N/A
SETD8	ENST00000402868.3	SET domain containing (lysine methyltransferase) 8	52	2	2	0	0	0	hsa-miR-23a-3p	-0.49	-0.49	N/A
MEIS1	ENST00000444274.2	Meis homeobox 1	533	1	1	0	0	1	hsa-miR-23a-3p	-0.49	-0.55	N/A
ZNF839	ENST00000262236.5	zinc finger protein 839	213	1	1	0	0	0	hsa-miR-23a-3p	-0.49	-0.49	N/A
B3GNT1	ENST00000311181.4	UDP-GlcNAc:betaGal beta-1,3-N-acetylglucosaminyltransferase 1	2688	1	1	0	0	0	hsa-miR-23a-3p	-0.49	-0.49	N/A
CCNG1	ENST00000340828.2	cyclin G1	1547	1	1	0	0	0	hsa-miR-23a-3p	-0.49	-0.49	N/A
ZNF23	ENST00000497160.1	zinc finger protein 23	5	1	1	0	0	0	hsa-miR-23a-3p	-0.49	-0.49	N/A

ZIC4	ENST00000383075.3	Zic family member 4	5	1	1	0	0	0hsa-miR-23a-3p	-0.48	-0.48	N/A
SIX1	ENST00000247182.6	SIX homeobox 1	1264	1	1	0	0	1hsa-miR-23a-3p	-0.48	-0.48	N/A
HEXIM1	ENST00000332499.2	hexamethylene bis-acetamide inducible 1	179	1	0	0	1	1hsa-miR-23a-3p	-0.48	-0.57	N/A
CTCF	ENST00000264010.4	CCCTC-binding factor (zinc finger protein)	1037	2	1	1	0	0hsa-miR-23a-3p	-0.47	-0.47	N/A
MAP7	ENST00000354570.3	microtubule-associated protein 7	444	2	1	1	0	1hsa-miR-23a-3p	-0.46	-0.46	N/A
RCHY1	ENST00000451788.1	protein ligase	821	1	1	0	0	0hsa-miR-23a-3p	-0.46	-0.5	N/A
SPTSSB	ENST00000359175.4	serine palmitoyltransferase, small subunit B	15	1	1	0	0	0hsa-miR-23a-3p	-0.46	-0.46	N/A
CCDC82	ENST00000278520.5	coiled-coil domain containing 82	224	1	1	0	0	1hsa-miR-23a-3p	-0.44	-0.44	N/A
FUT4	ENST00000358752.2	fucosyltransferase 4 (alpha (1,3) fucosyltransferase, myeloid-specific)	354	2	1	0	1	1hsa-miR-23a-3p	-0.44	-0.54	N/A
GLB1L3	ENST00000431683.2	galactosidase, beta 1-like 3	7	1	1	0	0	0hsa-miR-23a-3p	-0.44	-0.44	N/A
KIAA0922	ENST00000409663.3	KIAA0922	313	1	1	0	0	0hsa-miR-23a-3p	-0.44	-0.44	N/A
CLDN14	ENST00000399139.1	claudin 14	121	1	1	0	0	0hsa-miR-23a-3p	-0.44	-0.44	N/A
SYS1	ENST00000243918.5	SYS1 Golgi-localized integral membrane protein homolog (S. cerevisiae)	384	1	1	0	0	2hsa-miR-23a-3p	-0.44	-0.46	N/A
A1BG	ENST00000263100.3	alpha-1-B glycoprotein	74	1	1	0	0	0hsa-miR-23a-3p	-0.43	-0.43	N/A
FRA10AC1	ENST00000536233.1	fragile site, folic acid type, rare	289	1	1	0	0	1hsa-miR-23a-3p	-0.43	-0.48	N/A
PDCL3	ENST00000264254.6	phosducin-like 3	58	1	1	0	0	0hsa-miR-23a-3p	-0.43	-0.49	N/A
TNFAIP6	ENST00000243347.3	tumor necrosis factor, alpha-induced protein 6	14	1	1	0	0	0hsa-miR-23a-3p	-0.43	-0.43	N/A
TMEM87B	ENST00000283206.4	transmembrane protein 87B	219	1	1	0	0	0hsa-miR-23a-3p	-0.42	-0.51	N/A
DLGAP1	ENST00000400145.2	discs, large (Drosophila) homolog-associated protein 1	8	1	1	0	0	0hsa-miR-23a-3p	-0.42	-0.42	N/A
BLCAP	ENST00000397137.1	bladder cancer associated protein	38	1	1	0	0	0hsa-miR-23a-3p	-0.42	-0.62	N/A
SLC6A14	ENST00000371900.4	solute carrier family 6 (amino acid transporter), member 14	5	2	1	1	0	0hsa-miR-23a-3p	-0.42	-0.42	N/A
CXCL12	ENST00000343575.6	chemokine (C-X-C motif) ligand 12	719	1	0	1	0	0hsa-miR-23a-3p	-0.41	-0.41	N/A
MDFIC	ENST00000393486.1	MyoD family inhibitor domain containing	601	2	1	0	1	2hsa-miR-23a-3p	-0.41	-0.49	N/A
NRGN	ENST00000412681.2	neurogranin (protein kinase C substrate, RC3)	60	1	1	0	0	1hsa-miR-23a-3p	-0.41	-0.41	N/A
WTAP	ENST00000358372.4	Wilms tumor 1 associated protein	286	1	1	0	0	0hsa-miR-23a-3p	-0.41	-0.41	N/A
GALNT12	ENST00000375011.3	GalNAc-T12	73	1	1	0	0	1hsa-miR-23a-3p	-0.41	-0.42	N/A
CLDN12	ENST00000287916.4	claudin 12	267	1	1	0	0	0hsa-miR-23a-3p	-0.41	-0.41	N/A
PRKRIR	ENST00000260045.3	P58 repressor	13	1	1	0	0	0hsa-miR-23a-3p	-0.41	-0.41	N/A
MARCKS	ENST00000368635.4	myristoylated alanine-rich protein kinase C substrate	188	1	1	0	0	0hsa-miR-23a-3p	-0.4	-0.46	N/A
FUT9	ENST00000302103.5	fucosyltransferase 9 (alpha (1,3) fucosyltransferase)	5	2	2	0	0	2hsa-miR-23a-3p	-0.4	-0.4	N/A
KLF3	ENST00000261438.5	Kruppel-like factor 3 (basic)	515	4	1	1	2	1hsa-miR-23a-3p	-0.4	-0.67	N/A
TACR3	ENST00000304883.2	tachykinin receptor 3	5	1	1	0	0	1hsa-miR-23a-3p	-0.4	-0.4	N/A
WHAMM	ENST00000286760.4	WAS protein homolog associated with actin	834	1	1	0	0	1hsa-miR-23a-3p	-0.39	-0.39	N/A
IPPK	ENST00000287996.3	inositol 1,3,4,5,6-pentakisphosphate 2-kinase	211	1	1	0	0	1hsa-miR-23a-3p	-0.39	-0.39	N/A
CLEC1A	ENST00000315330.4	C-type lectin domain family 1, member A	5	1	1	0	0	0hsa-miR-23a-3p	-0.39	-0.39	N/A
ZNF292	ENST00000369577.3	zinc finger protein 292	230	2	1	1	0	1hsa-miR-23a-3p	-0.39	-0.43	N/A
ZNF793	ENST00000445217.1	zinc finger protein 793	22	1	1	0	0	0hsa-miR-23a-3p	-0.39	-0.4	N/A
ZNRF2	ENST00000323037.4	zinc and ring finger 2	403	2	2	0	0	1hsa-miR-23a-3p	-0.39	-0.75	N/A
ORMDL1	ENST00000392350.3	ORM1-like 1 (S. cerevisiae)	998	1	1	0	0	0hsa-miR-23a-3p	-0.39	-0.39	N/A

MAPRE1	ENST00000375571.5	microtubule-associated protein, RP/EB family, member 1	39	1	1	0	0	0hsa-miR-23a-3p	-0.35	-0.35	N/A
KCNIP4	ENST00000382148.3	Kv channel interacting protein 4	5	1	1	0	0	0hsa-miR-23a-3p	-0.35	-0.35	N/A
PLCXD3	ENST00000377801.3	phosphatidylinositol-specific phospholipase C, X domain containing 3	81	1	1	0	0	0hsa-miR-23a-3p	-0.35	-0.37	N/A
TMOD1	ENST00000259365.4	tropomodulin 1	115	1	1	0	0	1hsa-miR-23a-3p	-0.34	-0.4	N/A
PNMA2	ENST00000522362.2	paraneoplastic Ma antigen 2	362	1	1	0	0	0hsa-miR-23a-3p	-0.34	-0.35	N/A
RUFY2	ENST00000388768.2	RUN and FYVE domain containing 2	15	1	1	0	0	1hsa-miR-23a-3p	-0.34	-0.39	N/A
MARCKSL1	ENST00000329421.7	MARCKS-like 1	1382	1	1	0	0	0hsa-miR-23a-3p	-0.34	-0.4	N/A
RP11-210M15.2	ENST00000559008.1	Uncharacterized protein	5	1	0	1	0	2hsa-miR-23a-3p	-0.34	-0.34	N/A
FKBP5	ENST00000536438.1	FK506 binding protein 5	32	1	1	0	0	1hsa-miR-23a-3p	-0.34	-0.42	N/A
PRDX3	ENST00000356951.3	peroxiredoxin 3	141	1	1	0	0	1hsa-miR-23a-3p	-0.34	-0.37	N/A
PGRMC2	ENST00000296425.5	progesterone receptor membrane component 2	2089	1	1	0	0	0hsa-miR-23a-3p	-0.33	-0.38	N/A
LMNB1	ENST00000261366.5	lamin B1	369	1	0	0	1	1hsa-miR-23a-3p	-0.32	-0.34	N/A
CHST10	ENST00000264249.3	carbohydrate sulfotransferase 10	33	1	1	0	0	1hsa-miR-23a-3p	-0.32	-0.32	N/A
RPA3-AS1	ENST00000406829.1	RPA3 antisense RNA 1	22	1	1	0	0	0hsa-miR-23a-3p	-0.32	-0.5	N/A
ABCA1	ENST00000374736.3	ATP-binding cassette, sub-family A (ABC1), member 1	184	1	0	0	1	1hsa-miR-23a-3p	-0.32	-0.32	N/A
FBN1	ENST00000316623.5	fibrillin 1	798	1	0	1	0	1hsa-miR-23a-3p	-0.32	-0.5	N/A
MAPRE1	ENST00000375571.5	microtubule-associated protein, RP/EB family, member 1	39	1	1	0	0	0hsa-miR-23a-3p	-0.35	-0.35	N/A
KCNIP4	ENST00000382148.3	Kv channel interacting protein 4	5	1	1	0	0	0hsa-miR-23a-3p	-0.35	-0.35	N/A
PLCXD3	ENST00000377801.3	phosphatidylinositol-specific phospholipase C, X domain containing 3	81	1	1	0	0	0hsa-miR-23a-3p	-0.35	-0.37	N/A
TMOD1	ENST00000259365.4	tropomodulin 1	115	1	1	0	0	1hsa-miR-23a-3p	-0.34	-0.4	N/A
PNMA2	ENST00000522362.2	paraneoplastic Ma antigen 2	362	1	1	0	0	0hsa-miR-23a-3p	-0.34	-0.35	N/A
RUFY2	ENST00000388768.2	RUN and FYVE domain containing 2	15	1	1	0	0	1hsa-miR-23a-3p	-0.34	-0.39	N/A
MARCKSL1	ENST00000329421.7	MARCKS-like 1	1382	1	1	0	0	0hsa-miR-23a-3p	-0.34	-0.4	N/A
RP11-210M15.2	ENST00000559008.1	Uncharacterized protein	5	1	0	1	0	2hsa-miR-23a-3p	-0.34	-0.34	N/A
FKBP5	ENST00000536438.1	FK506 binding protein 5	32	1	1	0	0	1hsa-miR-23a-3p	-0.34	-0.42	N/A
PRDX3	ENST00000356951.3	peroxiredoxin 3	141	1	1	0	0	1hsa-miR-23a-3p	-0.34	-0.37	N/A
PGRMC2	ENST00000296425.5	progesterone receptor membrane component 2	2089	1	1	0	0	0hsa-miR-23a-3p	-0.33	-0.38	N/A
LMNB1	ENST00000261366.5	lamin B1	369	1	0	0	1	1hsa-miR-23a-3p	-0.32	-0.34	N/A
CHST10	ENST00000264249.3	carbohydrate sulfotransferase 10	33	1	1	0	0	1hsa-miR-23a-3p	-0.32	-0.32	N/A
RPA3-AS1	ENST00000406829.1	RPA3 antisense RNA 1	22	1	1	0	0	0hsa-miR-23a-3p	-0.32	-0.5	N/A
ABCA1	ENST00000374736.3	ATP-binding cassette, sub-family A (ABC1), member 1	184	1	0	0	1	1hsa-miR-23a-3p	-0.32	-0.32	N/A
FBN1	ENST00000316623.5	fibrillin 1	798	1	0	1	0	1hsa-miR-23a-3p	-0.32	-0.5	N/A
CUL3	ENST00000264414.4	cullin 3	1476	1	1	0	0	2hsa-miR-23a-3p	-0.32	-0.34	N/A
HOXB5	ENST00000239151.5	homeobox B5	55	1	1	0	0	0hsa-miR-23a-3p	-0.32	-0.32	N/A
TCF24	ENST00000563496.1	transcription factor 24	11	2	1	1	0	0hsa-miR-23a-3p	-0.32	-0.53	N/A
NRXN3	ENST00000281127.7	neurexin 3	14	1	1	0	0	3hsa-miR-23a-3p	-0.32	-0.34	N/A
ZC3H12C	ENST00000278590.3	zinc finger CCCH-type containing 12C	150	1	0	0	1	2hsa-miR-23a-3p	-0.31	-0.37	N/A
PPP2R5E	ENST00000337537.3	protein phosphatase 2, regulatory subunit B', epsilon isoform	23	2	1	1	0	2hsa-miR-23a-3p	-0.31	-0.33	N/A
CTNNBIP1	ENST00000377263.1	catenin, beta interacting protein 1	493	1	1	0	0	0hsa-miR-23a-3p	-0.31	-0.44	N/A

C6ORF50	ENST00000356722.3		5	1	0	1	0	1hsa-miR-23a-3p	-0.31	-0.31	N/A
BCL2	ENST00000398117.1	B-cell CLL/lymphoma 2	55	1	1	0	0	2hsa-miR-23a-3p	-0.31	-0.36	N/A
ZNF140	ENST00000440550.2	zinc finger protein 140	179	1	0	1	0	1hsa-miR-23a-3p	-0.31	-0.51	N/A
LRIG1	ENST00000273261.3	leucine-rich repeats and immunoglobulin-like domains 1	241	2	1	0	1	0hsa-miR-23a-3p	-0.3	-0.3	N/A
PPP1R12A	ENST00000261207.5	protein phosphatase 1, regulatory subunit 12A	276	2	1	0	1	0hsa-miR-23a-3p	-0.3	-0.31	N/A
HOXA3	ENST00000396352.4	homeobox A3	57	1	1	0	0	1hsa-miR-23a-3p	-0.3	-0.3	N/A
LYPLA1	ENST00000316963.3	lysophospholipase I	57	1	1	0	0	0hsa-miR-23a-3p	-0.3	-0.3	N/A
VPS26A	ENST00000395098.1	vacuolar protein sorting 26 homolog A (S. pombe)	127	1	1	0	0	1hsa-miR-23a-3p	-0.3	-0.3	N/A
PNRC1	ENST00000336032.3	proline-rich nuclear receptor coactivator 1	413	1	1	0	0	0hsa-miR-23a-3p	-0.3	-0.41	N/A
ATP6V1E1	ENST00000253413.5	ATPase	2139	1	0	1	0	0hsa-miR-23a-3p	-0.3	-0.46	N/A
SEMA6D	ENST00000355997.3	cytoplasmi (semaphorin) 6D	179	3	2	1	0	3hsa-miR-23a-3p	-0.3	-0.32	N/A
ZNF280C	ENST00000370978.4	zinc finger protein 280C	138	1	1	0	0	0hsa-miR-23a-3p	-0.29	-0.29	N/A
PRR13	ENST00000549068.1	proline rich 13	387	1	0	1	0	1hsa-miR-23a-3p	-0.29	-0.29	N/A
GHITM	ENST00000372134.3	growth hormone inducible transmembrane protein	2402	2	1	1	0	1hsa-miR-23a-3p	-0.29	-0.56	N/A
SLC25A36	ENST00000446041.2	solute carrier family 25 (pyrimidine nucleotide carrier), member 36	264	1	0	0	1	2hsa-miR-23a-3p	-0.29	-0.44	N/A
VSNL1	ENST00000406397.1	visinin-like 1	89	1	0	1	0	0hsa-miR-23a-3p	-0.29	-0.31	N/A
CNOT6L	ENST00000504123.1	CCR4-NOT transcription complex, subunit 6-like	23	3	1	2	0	0hsa-miR-23a-3p	-0.29	-0.39	N/A
TADA1	ENST00000367874.4	transcriptional adaptor 1	160	2	0	2	0	0hsa-miR-23a-3p	-0.29	-0.29	N/A
PJA1	ENST00000374583.1	praja ring finger 1, E3 ubiquitin protein ligase	765	1	1	0	0	0hsa-miR-23a-3p	-0.28	-0.28	N/A
TMEM2	ENST00000377044.4	transmembrane protein 2	130	1	1	0	0	0hsa-miR-23a-3p	-0.28	-0.28	N/A
KPNA5	ENST00000368564.1	karyopherin alpha 5 (importin alpha 6)	76	1	1	0	0	0hsa-miR-23a-3p	-0.28	-0.29	N/A
WNK1	ENST00000315939.6	WNK lysine deficient protein kinase 1	950	1	1	0	0	1hsa-miR-23a-3p	-0.28	-0.28	N/A
CALCR	ENST00000359558.2	calcitonin receptor	5	1	0	1	0	0hsa-miR-23a-3p	-0.28	-0.28	N/A
FAM134C	ENST00000309428.5	family with sequence similarity 134, member C	397	1	1	0	0	1hsa-miR-23a-3p	-0.28	-0.28	N/A
PTP4A2	ENST00000602725.1	protein tyrosine phosphatase type IVA, member 2	5	1	1	0	0	0hsa-miR-23a-3p	-0.28	-0.28	N/A
FBXO32	ENST00000517956.1	F-box protein 32	500	1	1	0	0	2hsa-miR-23a-3p	-0.27	-0.35	N/A
UBA6	ENST00000322244.5	ubiquitin-like modifier activating enzyme 6	169	1	1	0	0	1hsa-miR-23a-3p	-0.27	-0.31	N/A
DUSP5	ENST00000369583.3	dual specificity phosphatase 5	600	1	1	0	0	0hsa-miR-23a-3p	-0.27	-0.28	N/A
ACSS3	ENST00000548058.1	acyl-CoA synthetase short-chain family member 3	306	1	1	0	0	2hsa-miR-23a-3p	-0.27	-0.74	N/A
EBF3	ENST00000368648.3	early B-cell factor 3	101	1	1	0	0	0hsa-miR-23a-3p	-0.27	-0.27	N/A
NEU1	ENST00000375631.4	sialidase 1 (lysosomal sialidase)	6000	1	1	0	0	0hsa-miR-23a-3p	-0.27	-0.27	N/A
DEPDC1	ENST00000456315.2	DEP domain containing 1	1068	1	1	0	0	0hsa-miR-23a-3p	-0.27	-0.47	N/A
IL21R	ENST00000395755.1	interleukin 21 receptor	5	1	1	0	0	0hsa-miR-23a-3p	-0.27	-0.27	N/A
LPAR1	ENST00000374431.3	lysophosphatidic acid receptor 1	2111	1	1	0	0	1hsa-miR-23a-3p	-0.26	-0.29	N/A
PRRG1	ENST00000378628.4	proline rich Gla (G-carboxyglutamic acid) 1	1122	1	1	0	0	2hsa-miR-23a-3p	-0.26	-0.27	N/A
LBR	ENST00000272163.4	lamin B receptor	1217	1	0	1	0	1hsa-miR-23a-3p	-0.26	-0.27	N/A
GOLPH3L	ENST00000271732.3	golgi phosphoprotein 3-like	274	1	1	0	0	0hsa-miR-23a-3p	-0.26	-0.29	N/A
N4BP1	ENST00000262384.3	NEDD4 binding protein 1	105	1	1	0	0	1hsa-miR-23a-3p	-0.26	-0.26	N/A
PAX9	ENST00000361487.6	paired box 9	53	1	1	0	0	1hsa-miR-23a-3p	-0.26	-0.31	N/A

CREBZF	ENST00000398294.2	CREB/ATF bZIP transcription factor	1115	1	0	1	0	2hsa-miR-23a-3p	-0.26	-0.39	N/A
HS6ST3	ENST00000376705.2	heparan sulfate 6-O-sulfotransferase 3	598	1	0	0	1	0hsa-miR-23a-3p	-0.26	-0.26	N/A
MAF	ENST00000393350.1	v-maf avian musculoaponeurotic fibrosarcoma oncogene homolog	608	1	1	0	0	1hsa-miR-23a-3p	-0.26	-0.26	N/A
ZC4H2	ENST00000545618.1	zinc finger, C4H2 domain containing	432	1	0	1	0	0hsa-miR-23a-3p	-0.26	-0.26	N/A
ESRP1	ENST00000358397.5	epithelial splicing regulatory protein 1	74	1	1	0	0	0hsa-miR-23a-3p	-0.26	-0.26	N/A
B3GALT2	ENST00000367434.4	UDP-Gal:betaGlcNAc beta 1,3-galactosyltransferase, polypeptide 2	5	1	1	0	0	0hsa-miR-23a-3p	-0.26	-0.26	N/A
FKBP7	ENST00000424785.2	FK506 binding protein 7	645	1	0	1	0	0hsa-miR-23a-3p	-0.25	-0.26	N/A
HSP90B1	ENST00000299767.5	heat shock protein 90kDa beta (Grp94), member 1	42	1	1	0	0	0hsa-miR-23a-3p	-0.25	-0.31	N/A
NUS1	ENST00000368494.3	nuclear undecaprenyl pyrophosphate synthase 1 homolog (S. cerevisiae)	302	1	0	0	1	0hsa-miR-23a-3p	-0.25	-0.25	N/A
ACO1	ENST00000309951.6	aconitase 1, soluble	738	1	1	0	0	0hsa-miR-23a-3p	-0.25	-0.26	N/A
IDH1	ENST00000345146.2	isocitrate dehydrogenase 1 (NADP+), soluble	503	1	0	1	0	0hsa-miR-23a-3p	-0.25	-0.25	N/A
ZIC1	ENST00000282928.4	Zic family member 1	26	1	1	0	0	0hsa-miR-23a-3p	-0.25	-0.33	N/A
RXRG	ENST00000359842.5	retinoid X receptor, gamma	5	1	0	1	0	0hsa-miR-23a-3p	-0.25	-0.25	N/A
WBP4	ENST00000379487.3	WW domain binding protein 4	153	2	0	0	2	1hsa-miR-23a-3p	-0.24	-0.45	N/A
FGD4	ENST00000427716.2	FYVE, RhoGEF and PH domain containing 4	38	1	1	0	0	2hsa-miR-23a-3p	-0.24	-0.26	N/A
ELF4	ENST00000335997.7	E74-like factor 4 (ets domain transcription factor)	69	2	1	0	1	0hsa-miR-23a-3p	-0.24	-0.24	N/A
HAS2	ENST00000303924.4	hyaluronan synthase 2	398	1	1	0	0	0hsa-miR-23a-3p	-0.24	-0.27	N/A
SEMA4B	ENST00000411539.2	sema domain	3850	1	1	0	0	0hsa-miR-23a-3p	-0.24	-0.24	N/A
ATXN7L3B	ENST00000519948.2	ataxin 7-like 3B	55	1	1	0	0	0hsa-miR-23a-3p	-0.24	-0.24	N/A
ALAS1	ENST00000394965.2	aminolevulinate, delta-, synthase 1	440	1	0	0	1	0hsa-miR-23a-3p	-0.24	-0.24	N/A
SWT1	ENST00000367500.4	SWT1 RNA endoribonuclease homolog (S. cerevisiae)	104	1	1	0	0	1hsa-miR-23a-3p	-0.23	-0.23	N/A
TBR1	ENST00000389554.3	T-box, brain, 1	5	1	1	0	0	0hsa-miR-23a-3p	-0.23	-0.23	N/A
FAXDC2	ENST00000326080.5	fatty acid hydroxylase domain containing 2	43	1	1	0	0	0hsa-miR-23a-3p	-0.23	-0.23	N/A
ANKRD50	ENST00000504087.1	ankyrin repeat domain 50	152	2	1	1	0	2hsa-miR-23a-3p	-0.23	-0.28	N/A
MSMO1	ENST00000261507.6	methylsterol monooxygenase 1	1413	1	0	1	0	0hsa-miR-23a-3p	-0.23	-0.25	N/A
TGFBR2	ENST00000359013.4	transforming growth factor, beta receptor II (70/80kDa)	2168	1	1	0	0	4hsa-miR-23a-3p	-0.23	-0.3	N/A
CTXN2	ENST00000417307.2	cortexin 2	5	1	1	0	0	0hsa-miR-23a-3p	-0.23	-0.23	N/A
TFPI2	ENST00000222543.5	tissue factor pathway inhibitor 2	11151	1	0	1	0	0hsa-miR-23a-3p	-0.23	-0.23	N/A
STX17	ENST00000259400.6	syntaxin 17	510	1	1	0	0	1hsa-miR-23a-3p	-0.23	-0.23	N/A
GAP43	ENST00000305124.6	growth associated protein 43	5	1	0	1	0	0hsa-miR-23a-3p	-0.23	-0.23	N/A
ZNF579	ENST00000325421.4	zinc finger protein 579	1080	1	0	1	0	0hsa-miR-23a-3p	-0.23	-0.24	N/A
CASP7	ENST00000369331.4	caspase 7, apoptosis-related cysteine peptidase	277	1	1	0	0	0hsa-miR-23a-3p	-0.23	-0.23	N/A
TMEM229A	ENST00000455783.1	transmembrane protein 229A	5	1	1	0	0	0hsa-miR-23a-3p	-0.23	-0.23	N/A
RNF38	ENST00000259605.6	ring finger protein 38	348	1	1	0	0	0hsa-miR-23a-3p	-0.22	-0.24	N/A
ZCCHC2	ENST00000269499.5	zinc finger, CCHC domain containing 2	13	1	1	0	0	0hsa-miR-23a-3p	-0.22	-0.22	N/A
INTS6	ENST00000311234.4	integrator complex subunit 6	433	1	1	0	0	0hsa-miR-23a-3p	-0.22	-0.22	N/A
MPP2	ENST00000377184.3	membrane protein, palmitoylated 2 (MAGUK p55 subfamily member 2)	78	1	1	0	0	0hsa-miR-23a-3p	-0.22	-0.34	N/A
HOXD10	ENST00000249501.4	homeobox D10	182	2	0	2	0	0hsa-miR-23a-3p	-0.22	-0.22	N/A
BTAF1	ENST00000265990.6	BTAF1 RNA polymerase II, B-TFIID transcription factor-associated, 170kDa	917	1	1	0	0	1hsa-miR-23a-3p	-0.22	-0.22	N/A

LIPH	ENST00000296252.4	lipase, member H	18	1	1	0	0	0hsa-miR-23a-3p	-0.22	-0.22	N/A
NAA50	ENST00000240922.3	N(alpha)-acetyltransferase 50, NatE catalytic subunit	260	1	1	0	0	1hsa-miR-23a-3p	-0.22	-0.33	N/A
GNPDA1	ENST00000311337.6	glucosamine-6-phosphate deaminase 1	1130	1	0	1	0	1hsa-miR-23a-3p	-0.22	-0.22	N/A
SERINC5	ENST00000512721.1	serine incorporator 5	175	1	1	0	0	0hsa-miR-23a-3p	-0.22	-0.22	N/A
PAQR9	ENST00000340634.3	progestin and adipoQ receptor family member IX	233	1	1	0	0	0hsa-miR-23a-3p	-0.21	-0.21	N/A
DLX1	ENST00000341900.6	distal-less homeobox 1	312	1	0	1	0	1hsa-miR-23a-3p	-0.21	-0.21	N/A
HTR2C	ENST00000371950.3	5-hydroxytryptamine (serotonin) receptor 2C, G protein-coupled	5	1	1	0	0	1hsa-miR-23a-3p	-0.21	-0.21	N/A
ETNK1	ENST00000266517.4	ethanolamine kinase 1	1047	4	3	0	1	0hsa-miR-23a-3p	-0.21	-0.5	N/A
EIF1	ENST00000469257.1	eukaryotic translation initiation factor 1	415	2	0	1	1	0hsa-miR-23a-3p	-0.21	-0.21	N/A
CLK3	ENST00000345005.4	CDC-like kinase 3	127	1	1	0	0	0hsa-miR-23a-3p	-0.21	-0.21	N/A
PLEKHF2	ENST00000315367.3	pleckstrin homology domain containing	286	1	1	0	0	0hsa-miR-23a-3p	-0.21	-0.26	N/A
LPP	ENST00000312675.4	LIM domain containing preferred translocation partner in lipoma	1894	2	2	0	0	0hsa-miR-23a-3p	-0.21	-0.21	N/A
FUNDC2	ENST00000369498.3	FUN14 domain containing 2	130	1	1	0	0	0hsa-miR-23a-3p	-0.21	-0.3	N/A
HS6ST2	ENST00000370836.2	heparan sulfate 6-O-sulfotransferase 2	4566	1	1	0	0	0hsa-miR-23a-3p	-0.21	-0.21	N/A
C5orf42	ENST00000508244.1	chromosome 5 open reading frame 42	45	1	1	0	0	1hsa-miR-23a-3p	-0.21	-0.26	N/A
DHX15	ENST00000336812.4	DEAH (Asp-Glu-Ala-His) box helicase 15	1050	1	0	1	0	0hsa-miR-23a-3p	-0.21	-0.21	N/A
NUCKS1	ENST00000367142.4	nuclear casein kinase and cyclin-dependent kinase substrate 1	1692	1	1	0	0	0hsa-miR-23a-3p	-0.21	-0.27	N/A
HSPH1	ENST00000320027.5	heat shock 105kDa/110kDa protein 1	4109	1	0	0	1	1hsa-miR-23a-3p	-0.21	-0.21	N/A
CDKN2AIP	ENST00000302350.4	CDKN2A interacting protein	2313	1	1	0	0	0hsa-miR-23a-3p	-0.2	-0.2	N/A
IRF1	ENST00000245414.4	interferon regulatory factor 1	266	1	1	0	0	0hsa-miR-23a-3p	-0.2	-0.2	N/A
SREK1IP1	ENST00000513458.4	SREK1-interacting protein 1	145	1	1	0	0	2hsa-miR-23a-3p	-0.2	-0.71	N/A
ZNF654	ENST00000309495.5	zinc finger protein 654	456	1	1	0	0	0hsa-miR-23a-3p	-0.2	-0.21	N/A
RAB8B	ENST00000321437.4	RAB8B, member RAS oncogene family	45	2	2	0	0	0hsa-miR-23a-3p	-0.2	-0.34	N/A
PARD6B	ENST00000371610.2	par-6 family cell polarity regulator beta	277	1	1	0	0	1hsa-miR-23a-3p	-0.2	-0.2	N/A
MINPP1	ENST00000371994.4	multiple inositol-polyphosphate phosphatase 1	1079	1	1	0	0	2hsa-miR-23a-3p	-0.2	-0.2	N/A
TMEM144	ENST00000296529.6	transmembrane protein 144	131	1	0	1	0	0hsa-miR-23a-3p	-0.2	-0.53	N/A
PRDM10	ENST00000358825.5	PR domain containing 10	68	1	1	0	0	0hsa-miR-23a-3p	-0.2	-0.2	N/A
RBM12B	ENST00000399300.2	RNA binding motif protein 12B	143	1	1	0	0	0hsa-miR-23a-3p	-0.2	-0.33	N/A
NCOA6	ENST00000374796.2	nuclear receptor coactivator 6	984	1	1	0	0	0hsa-miR-23a-3p	-0.2	-0.23	N/A
SMS	ENST00000404933.2	spermine synthase	14	1	0	1	0	0hsa-miR-23a-3p	-0.2	-0.2	N/A
CSNK2A2	ENST00000262506.3	casein kinase 2, alpha prime polypeptide	99	1	1	0	0	0hsa-miR-23a-3p	-0.2	-0.2	N/A
SLC25A40	ENST00000341119.5	solute carrier family 25, member 40	405	1	1	0	0	0hsa-miR-23a-3p	-0.2	-0.21	N/A
MEF2C	ENST00000340208.5	myocyte enhancer factor 2C	70	2	2	0	0	1hsa-miR-23a-3p	-0.19	-0.25	N/A
HOOK2	ENST00000397668.3	hook microtubule-tethering protein 2	9	1	0	1	0	0hsa-miR-23a-3p	-0.19	-0.19	N/A
BAZ2B	ENST00000392782.1	bromodomain adjacent to zinc finger domain, 2B	25	1	1	0	0	1hsa-miR-23a-3p	-0.19	-0.21	N/A
PTGR2	ENST00000555661.1	prostaglandin reductase 2	244	1	0	1	0	0hsa-miR-23a-3p	-0.19	-0.19	N/A
TNKS2	ENST00000371627.4	tankyrase, TRF1-interacting ankyrin-related ADP-ribose polymerase 2	205	1	1	0	0	0hsa-miR-23a-3p	-0.19	-0.19	N/A
C8orf58	ENST00000289989.5	chromosome 8 open reading frame 58	619	1	1	0	0	0hsa-miR-23a-3p	-0.19	-0.19	N/A
SFRP4	ENST00000436072.2	secreted frizzled-related protein 4	5	1	0	1	0	2hsa-miR-23a-3p	-0.19	-0.19	N/A

SLC1A3	ENST00000265113.4	solute carrier family 1 (glial high affinity glutamate transporter)	34	1	0	1	0	0hsa-miR-23a-3p	-0.18	-0.18	N/A
RNF219	ENST00000282003.6	ring finger protein 219	680	1	0	0	1	0hsa-miR-23a-3p	-0.18	-0.18	N/A
KIAA1737	ENST00000361786.2	KIAA1737	57	1	1	0	0	0hsa-miR-23a-3p	-0.18	-0.21	N/A
MEF2A	ENST00000354410.5	myocyte enhancer factor 2A	654	1	1	0	0	0hsa-miR-23a-3p	-0.18	-0.21	N/A
BMP2K	ENST00000335016.5	BMP2 inducible kinase	164	2	2	0	0	0hsa-miR-23a-3p	-0.18	-0.19	N/A
MFHAS1	ENST00000276282.6	malignant fibrous histiocytoma amplified sequence 1	130	1	1	0	0	1hsa-miR-23a-3p	-0.18	-0.18	N/A
CHL1	ENST00000256509.2	cell adhesion molecule L1-like	24	1	1	0	0	0hsa-miR-23a-3p	-0.18	-0.18	N/A
KIAA1432	ENST00000414202.2	KIAA1432	1366	1	0	1	0	3hsa-miR-23a-3p	-0.18	-0.21	N/A
EGLN2	ENST00000593726.1	egl-9 family hypoxia-inducible factor 2	19	1	1	0	0	0hsa-miR-23a-3p	-0.18	-0.18	N/A
EOMES	ENST00000295743.4	eomesodermin	28	1	1	0	0	0hsa-miR-23a-3p	-0.18	-0.18	N/A
KIAA1958	ENST00000337530.6	KIAA1958	117	2	2	0	0	1hsa-miR-23a-3p	-0.18	-0.2	N/A
SNX27	ENST00000368843.3	sorting nexin family member 27	46	1	1	0	0	2hsa-miR-23a-3p	-0.17	-0.17	N/A
PROSC	ENST00000328195.3	proline synthetase co-transcribed homolog (bacterial)	414	1	0	1	0	0hsa-miR-23a-3p	-0.17	-0.18	N/A
LDB2	ENST00000441778.2	LIM domain binding 2	23	1	0	0	1	1hsa-miR-23a-3p	-0.17	-0.17	N/A
FOXB1	ENST00000396057.4	forkhead box B1	31	1	1	0	0	0hsa-miR-23a-3p	-0.17	-0.21	N/A
CEP350	ENST00000367607.3	centrosomal protein 350kDa	705	1	1	0	0	0hsa-miR-23a-3p	-0.17	-0.2	N/A
GPRC5B	ENST00000300571.2	G protein-coupled receptor, family C, group 5, member B	84	1	1	0	0	0hsa-miR-23a-3p	-0.17	-0.17	N/A
AUTS2	ENST00000342771.4	autism susceptibility candidate 2	198	1	1	0	0	2hsa-miR-23a-3p	-0.17	-0.34	N/A
FOPNL	ENST00000573396.1	FGFR1OP N-terminal like	29	1	1	0	0	0hsa-miR-23a-3p	-0.17	-0.26	N/A
ZBTB2	ENST00000325144.4	zinc finger and BTB domain containing 2	832	1	0	1	0	0hsa-miR-23a-3p	-0.17	-0.17	N/A
PPP1R16B	ENST00000299824.1	protein phosphatase 1, regulatory subunit 16B	16	1	0	0	1	0hsa-miR-23a-3p	-0.17	-0.17	N/A
OTUB1	ENST00000538426.1	OTU domain, ubiquitin aldehyde binding 1	2977	1	1	0	0	0hsa-miR-23a-3p	-0.17	-0.35	N/A
TMEM64	ENST00000458549.2	transmembrane protein 64	200	1	1	0	0	0hsa-miR-23a-3p	-0.17	-0.21	N/A
FOSB	ENST00000586615.1	FBJ murine osteosarcoma viral oncogene homolog B	11	1	1	0	0	0hsa-miR-23a-3p	-0.17	-0.17	N/A
GNG10	ENST00000374293.4	guanine nucleotide binding protein (G protein), gamma 10	407	1	0	1	0	0hsa-miR-23a-3p	-0.17	-0.17	N/A
PKDCC	ENST00000294964.5	protein kinase domain containing, cytoplasmic	123	1	0	1	0	1hsa-miR-23a-3p	-0.17	-0.17	N/A
DNAJC30	ENST00000395176.2	DnaJ (Hsp40) homolog, subfamily C, member 30	766	1	0	1	0	0hsa-miR-23a-3p	-0.16	-0.17	N/A
CHUK	ENST00000370397.7	conserved helix-loop-helix ubiquitous kinase	18	1	1	0	0	0hsa-miR-23a-3p	-0.16	-0.35	N/A
PDXDC1	ENST00000396410.4	pyridoxal-dependent decarboxylase domain containing 1	6417	1	1	0	0	0hsa-miR-23a-3p	-0.16	-0.16	N/A
FAM73A	ENST00000370791.3	family with sequence similarity 73, member A	837	1	1	0	0	0hsa-miR-23a-3p	-0.16	-0.16	N/A
FOXL2	ENST00000330315.3	forkhead box L2	20	1	1	0	0	0hsa-miR-23a-3p	-0.16	-0.16	N/A
RAI14	ENST00000265109.3	retinoic acid induced 14	2228	1	1	0	0	0hsa-miR-23a-3p	-0.16	-0.17	N/A
RNF43	ENST00000407977.2	ring finger protein 43	52	1	1	0	0	0hsa-miR-23a-3p	-0.16	-0.16	N/A
MPHOSPH9	ENST00000302349.5	M-phase phosphoprotein 9	180	1	0	1	0	0hsa-miR-23a-3p	-0.16	-0.16	N/A
SETD2	ENST00000409792.3	SET domain containing 2	196	1	0	0	1	0hsa-miR-23a-3p	-0.16	-0.16	N/A
RTCB	ENST00000451746.2	RNA 2',3'-cyclic phosphate and 5'-OH ligase	9513	1	0	1	0	0hsa-miR-23a-3p	-0.16	-0.16	N/A
DIP2C	ENST00000280886.6	DIP2 disco-interacting protein 2 homolog C (Drosophila)	359	1	1	0	0	0hsa-miR-23a-3p	-0.16	-0.16	N/A
TLK1	ENST00000431350.2	tousled-like kinase 1	200	3	2	1	0	0hsa-miR-23a-3p	-0.15	-0.32	N/A
LHX4	ENST00000263726.2	LIM homeobox 4	13	1	1	0	0	3hsa-miR-23a-3p	-0.15	-0.15	N/A

PIK3R3	ENST00000262741.5	phosphoinositide-3-kinase, regulatory subunit 3 (gamma)	335	1	1	0	0	1hsa-miR-23a-3p	-0.15	-0.17	N/A
PHF21B	ENST00000403565.1	PHD finger protein 21B	8	1	1	0	0	0hsa-miR-23a-3p	-0.15	-0.15	N/A
GGNBP2	ENST00000304718.4	gametogenetin binding protein 2	1011	1	0	1	0	0hsa-miR-23a-3p	-0.15	-0.21	N/A
PTGFR	ENST00000370756.3	prostaglandin F receptor (FP)	20	1	0	0	1	1hsa-miR-23a-3p	-0.15	-0.15	N/A
CIB2	ENST00000258930.3	calcium and integrin binding family member 2	119	1	1	0	0	0hsa-miR-23a-3p	-0.15	-0.15	N/A
STK35	ENST00000381482.3	serine/threonine kinase 35	263	1	1	0	0	2hsa-miR-23a-3p	-0.15	-0.24	N/A
EIF3M	ENST00000531120.1	eukaryotic translation initiation factor 3, subunit M	218	1	1	0	0	0hsa-miR-23a-3p	-0.15	-0.16	N/A
HNRNPU	ENST00000444376.2	heterogeneous nuclear ribonucleoprotein U (scaffold attachment factor A)	5343	1	0	1	0	0hsa-miR-23a-3p	-0.15	-0.19	N/A
MATN1	ENST00000373765.4	matrilin 1, cartilage matrix protein	5	1	0	1	0	0hsa-miR-23a-3p	-0.15	-0.15	N/A
ADRA2B	ENST00000409345.3	adrenoceptor alpha 2B	7	1	0	1	0	0hsa-miR-23a-3p	-0.15	-0.15	N/A
STAU2	ENST00000522695.1	staufen double-stranded RNA binding protein 2	306	2	0	1	1	0hsa-miR-23a-3p	-0.15	-0.15	N/A
DLD	ENST00000205402.5	dihydrolipoamide dehydrogenase	1623	1	0	0	1	0hsa-miR-23a-3p	-0.15	-0.47	N/A
CCAR1	ENST00000265872.6	cell division cycle and apoptosis regulator 1	1502	1	0	1	0	0hsa-miR-23a-3p	-0.15	-0.15	N/A
HOXC11	ENST00000546378.1	homeobox C11	80	1	0	1	0	0hsa-miR-23a-3p	-0.15	-0.15	N/A
PDPK1	ENST00000441549.3	3-phosphoinositide dependent protein kinase-1	104	1	1	0	0	1hsa-miR-23a-3p	-0.15	-0.15	N/A
CBLN1	ENST00000219197.6	cerebellin 1 precursor	12	1	1	0	0	0hsa-miR-23a-3p	-0.15	-0.39	N/A
STAT5A	ENST00000345506.4	signal transducer and activator of transcription 5A	221	1	1	0	0	0hsa-miR-23a-3p	-0.14	-0.14	N/A
TOPBP1	ENST00000260810.5	topoisomerase (DNA) II binding protein 1	102	1	0	1	0	0hsa-miR-23a-3p	-0.14	-0.17	N/A
TSPYL4	ENST00000420283.1	TSPY-like 4	5	1	0	1	0	2hsa-miR-23a-3p	-0.14	-0.14	N/A
RIPK4	ENST00000332512.3	receptor-interacting serine-threonine kinase 4	112	1	1	0	0	0hsa-miR-23a-3p	-0.14	-0.14	N/A
ACTN2	ENST00000366578.4	actinin, alpha 2	11	1	1	0	0	1hsa-miR-23a-3p	-0.14	-0.33	N/A
LRCH1	ENST00000311191.6	leucine-rich repeats and calponin homology (CH) domain containing 1	381	1	1	0	0	0hsa-miR-23a-3p	-0.14	-0.14	N/A
POM121C	ENST00000257665.5	POM121 transmembrane nucleoporin C	5	1	0	1	0	0hsa-miR-23a-3p	-0.14	-0.14	N/A
USP46	ENST00000441222.3	ubiquitin specific peptidase 46	180	1	1	0	0	1hsa-miR-23a-3p	-0.14	-0.15	N/A
USP5	ENST00000229268.8	ubiquitin specific peptidase 5 (isopeptidase T)	104	1	0	0	1	1hsa-miR-23a-3p	-0.14	-0.14	N/A
ELL2	ENST00000237853.4	elongation factor, RNA polymerase II, 2	397	1	0	0	1	1hsa-miR-23a-3p	-0.14	-0.14	N/A
KIAA1109	ENST00000388738.3	KIAA1109	591	1	0	1	0	0hsa-miR-23a-3p	-0.14	-0.14	N/A
SPOCK1	ENST00000394945.1	sparc/osteonectin, cwcv and kazal-like domains proteoglycan (testican) 1	935	1	1	0	0	1hsa-miR-23a-3p	-0.14	-0.14	N/A
MORC4	ENST00000535534.1	MORC family CW-type zinc finger 4	112	1	1	0	0	0hsa-miR-23a-3p	-0.14	-0.16	N/A
WASL	ENST00000223023.4	Wiskott-Aldrich syndrome-like	230	1	0	0	1	1hsa-miR-23a-3p	-0.14	-0.14	N/A
NLGN4X	ENST00000381095.3	neuroligin 4, X-linked	5	2	1	1	0	1hsa-miR-23a-3p	-0.14	-0.14	N/A
CASK	ENST00000421587.2	calcium/calmodulin-dependent serine protein kinase (MAGUK family)	892	1	0	1	0	1hsa-miR-23a-3p	-0.14	-0.16	N/A
ISL1	ENST00000230658.7	ISL LIM homeobox 1	67	1	0	0	1	0hsa-miR-23a-3p	-0.14	-0.15	N/A
PAPD5	ENST00000357464.3	PAP associated domain containing 5	152	1	0	1	0	1hsa-miR-23a-3p	-0.14	-0.15	N/A
EPAS1	ENST00000263734.3	endothelial PAS domain protein 1	3689	1	1	0	0	1hsa-miR-23a-3p	-0.14	-0.14	N/A
RCOR1	ENST00000262241.6	REST corepressor 1	403	1	1	0	0	2hsa-miR-23a-3p	-0.14	-0.18	N/A
ISM1	ENST00000262487.4	isthmin 1, angiogenesis inhibitor	9	1	0	1	0	0hsa-miR-23a-3p	-0.14	-0.14	N/A
TMEM38B	ENST00000374692.3	transmembrane protein 38B	755	1	0	0	1	1hsa-miR-23a-3p	-0.14	-0.23	N/A
VAPB	ENST00000475243.1	VAMP (vesicle-associated membrane protein)-associated protein B and C	391	1	1	0	0	0hsa-miR-23a-3p	-0.14	-0.15	N/A

FAM46A	ENST00000369754.3	family with sequence similarity 46, member A	899	1	1	0	0	0hsa-miR-23a-3p	-0.14	-0.14	N/A
FBXL14	ENST00000339235.3	F-box and leucine-rich repeat protein 14	76	1	0	0	1	0hsa-miR-23a-3p	-0.14	-0.14	N/A
STAT1	ENST00000540176.1	signal transducer and activator of transcription 1, 91kDa	326	1	0	0	1	0hsa-miR-23a-3p	-0.14	-0.14	N/A
PRDM1	ENST00000369089.3	PR domain containing 1, with ZNF domain	48	2	1	0	1	0hsa-miR-23a-3p	-0.14	-0.17	N/A
INO80D	ENST00000403263.1	INO80 complex subunit D	5	1	1	0	0	0hsa-miR-23a-3p	-0.14	-0.14	N/A
SRPK2	ENST00000393651.3	SRSF protein kinase 2	381	1	0	1	0	0hsa-miR-23a-3p	-0.13	-0.16	N/A
EIF4E3	ENST00000425534.3	eukaryotic translation initiation factor 4E family member 3	475	2	0	0	2	2hsa-miR-23a-3p	-0.13	-0.2	N/A
KCNK3	ENST00000302909.3	potassium channel, subfamily K, member 3	19	1	1	0	0	0hsa-miR-23a-3p	-0.13	-0.15	N/A
TIA1	ENST00000433529.2	TIA1 cytotoxic granule-associated RNA binding protein	1370	1	0	1	0	0hsa-miR-23a-3p	-0.13	-0.14	N/A
FAM126B	ENST00000418596.3	family with sequence similarity 126, member B	132	2	1	1	0	0hsa-miR-23a-3p	-0.13	-0.25	N/A
ACTBL2	ENST00000423391.1	actin, beta-like 2	9	1	1	0	0	0hsa-miR-23a-3p	-0.13	-0.13	N/A
DNAJC25-GNG10	ENST00000374294.3	DNAJC25-GNG10 readthrough	407	1	0	1	0	0hsa-miR-23a-3p	-0.13	-0.13	N/A
FAS	ENST00000352159.4	Fas cell surface death receptor	378	1	1	0	0	0hsa-miR-23a-3p	-0.13	-0.51	N/A
FRAT2	ENST00000371019.2	frequently rearranged in advanced T-cell lymphomas 2	120	1	0	1	0	0hsa-miR-23a-3p	-0.13	-0.21	N/A
GPSM1	ENST00000440944.1	G-protein signaling modulator 1	5243	1	1	0	0	0hsa-miR-23a-3p	-0.13	-0.13	N/A
PLEKHA3	ENST00000234453.5	pleckstrin homology domain containing, family A	767	1	0	0	1	3hsa-miR-23a-3p	-0.13	-0.19	N/A
MYCBP	ENST00000397572.2	MYC binding protein	85	1	0	0	1	0hsa-miR-23a-3p	-0.13	-0.19	N/A
TNRC6B	ENST00000335727.9	trinucleotide repeat containing 6B	69	2	2	0	0	0hsa-miR-23a-3p	-0.12	-0.15	N/A
ANKFY1	ENST00000341657.4	ankyrin repeat and FYVE domain containing 1	969	1	0	1	0	1hsa-miR-23a-3p	-0.12	-0.15	N/A
SLC12A2	ENST00000262461.2	solute carrier family 12 (sodium/potassium/chloride transporter), member 2	655	1	1	0	0	0hsa-miR-23a-3p	-0.12	-0.13	N/A
SOX11	ENST00000322002.3	SRY (sex determining region Y)-box 11	1100	1	0	1	0	0hsa-miR-23a-3p	-0.12	-0.13	N/A
PNMAL1	ENST00000313683.10	paraneoplastic Ma antigen family-like 1	313	1	1	0	0	0hsa-miR-23a-3p	-0.12	-0.12	N/A
RAD17	ENST00000361732.2	RAD17 homolog (S. pombe)	5	1	0	0	1	0hsa-miR-23a-3p	-0.12	-0.12	N/A
TTC33	ENST00000337702.4	tetratricopeptide repeat domain 33	676	1	0	1	0	0hsa-miR-23a-3p	-0.12	-0.25	N/A
C4orf29	ENST00000388795.5	chromosome 4 open reading frame 29	67	2	1	0	1	0hsa-miR-23a-3p	-0.12	-0.22	N/A
FOXO4	ENST00000374259.3	forkhead box O4	48	1	1	0	0	0hsa-miR-23a-3p	-0.12	-0.12	N/A
FAM222B	ENST00000582266.1	family with sequence similarity 222, member B	100	1	0	1	0	0hsa-miR-23a-3p	-0.12	-0.13	N/A
CAPN6	ENST00000324068.1	calpain 6	5	1	1	0	0	0hsa-miR-23a-3p	-0.12	-0.12	N/A
ZBTB43	ENST00000449886.1	zinc finger and BTB domain containing 43	396	2	0	2	0	0hsa-miR-23a-3p	-0.12	-0.12	N/A
CSNK2A1	ENST00000349736.5	casein kinase 2, alpha 1 polypeptide	1927	1	0	0	1	0hsa-miR-23a-3p	-0.12	-0.12	N/A
NACC1	ENST00000292431.4	nucleus accumbens associated 1, BEN and BTB (POZ) domain containing	337	1	1	0	0	0hsa-miR-23a-3p	-0.12	-0.13	N/A
DIO2	ENST00000438257.4	deiodinase, iodothyronine, type II	98	1	0	0	1	0hsa-miR-23a-3p	-0.12	-0.12	N/A
JHDM1D	ENST00000397560.2	jumonji C domain containing histone demethylase 1 homolog D	470	1	1	0	0	1hsa-miR-23a-3p	-0.12	-0.12	N/A
GLYCTK	ENST00000354773.4	glycerate kinase	3833	1	0	1	0	0hsa-miR-23a-3p	-0.12	-0.12	N/A
FAM178B	ENST00000417561.3	family with sequence similarity 178, member B	5	1	0	1	0	0hsa-miR-23a-3p	-0.12	-0.12	N/A
TADA2A	ENST00000394395.2	transcriptional adaptor 2A	278	1	0	1	0	0hsa-miR-23a-3p	-0.12	-0.12	N/A
ATMIN	ENST00000299575.4	ATM interactor	129	1	0	1	0	0hsa-miR-23a-3p	-0.12	-0.12	N/A
COL11A2	ENST00000374708.4	collagen, type XI, alpha 2	19	1	0	1	0	0hsa-miR-23a-3p	-0.12	-0.14	N/A
PLA2R1	ENST00000283243.7	phospholipase A2 receptor 1, 180kDa	41	1	1	0	0	0hsa-miR-23a-3p	-0.11	-0.13	N/A

FRK	ENST00000606080.1	fyn-related kinase	37	1	1	0	0	7	hsa-miR-23a-3p	-0.11	-0.27	N/A
STRIP2	ENST00000249344.2	striatin interacting protein 2	87	1	1	0	0	1	hsa-miR-23a-3p	-0.11	-0.14	N/A
MRC2	ENST00000303375.5	mannose receptor, C type 2	74	1	1	0	0	0	hsa-miR-23a-3p	-0.11	-0.11	N/A
AEBP2	ENST00000266508.9	AE binding protein 2	1228	1	0	1	0	2	hsa-miR-23a-3p	-0.11	-0.11	N/A
GRAP2	ENST00000344138.4	GRB2-related adaptor protein 2	5	1	1	0	0	2	hsa-miR-23a-3p	-0.11	-0.14	N/A
FRAT1	ENST00000371021.3	frequently rearranged in advanced T-cell lymphomas	631	1	0	1	0	1	hsa-miR-23a-3p	-0.11	-0.11	N/A
RIMKLA	ENST00000431473.3	ribosomal modification protein rimK-like family member A	100	1	0	1	0	1	hsa-miR-23a-3p	-0.11	-0.28	N/A
SLC36A2	ENST00000335244.4	solute carrier family 36 (proton/amino acid symporter), member 2	5	1	0	1	0	0	hsa-miR-23a-3p	-0.11	-0.11	N/A
SH2B3	ENST00000341259.2	SH2B adaptor protein 3	89	1	0	0	1	0	hsa-miR-23a-3p	-0.11	-0.13	N/A
MITF	ENST00000328528.6	microphthalmia-associated transcription factor	593	1	1	0	0	0	hsa-miR-23a-3p	-0.11	-0.12	N/A
WASF3	ENST00000335327.5	WAS protein family, member 3	24	1	0	1	0	0	hsa-miR-23a-3p	-0.11	-0.11	N/A
TBC1D15	ENST00000550746.1	TBC1 domain family, member 15	1164	1	0	1	0	0	hsa-miR-23a-3p	-0.11	-0.2	N/A
ANXA2	ENST00000396024.3	annexin A2	30	1	0	0	1	0	hsa-miR-23a-3p	-0.11	-0.12	N/A
TBC1D9	ENST00000442267.2	TBC1 domain family, member 9 (with GRAM domain)	200	1	1	0	0	0	hsa-miR-23a-3p	-0.11	-0.28	N/A
CD163	ENST00000541972.1	CD163 molecule	5	1	0	1	0	0	hsa-miR-23a-3p	-0.11	-0.11	N/A
EIF4ENIF1	ENST00000344710.5	eukaryotic translation initiation factor 4E nuclear import factor 1	1075	1	0	0	1	0	hsa-miR-23a-3p	-0.11	-0.11	N/A
RAD51L3-RFFL	ENST00000593039.1	Uncharacterized protein	670	1	0	0	1	1	hsa-miR-23a-3p	-0.11	-0.13	N/A
C11orf30	ENST00000529032.1	chromosome 11 open reading frame 30	230	1	1	0	0	0	hsa-miR-23a-3p	-0.1	-0.1	N/A
ABL2	ENST00000502732.1	c-abl oncogene 2, non-receptor tyrosine kinase	892	1	0	0	1	3	hsa-miR-23a-3p	-0.1	-0.11	N/A
TMEM178B	ENST00000565468.1	transmembrane protein 178B	129	1	0	0	1	3	hsa-miR-23a-3p	-0.1	-0.1	N/A
EIF3A	ENST00000369144.3	eukaryotic translation initiation factor 3, subunit A	638	1	0	1	0	0	hsa-miR-23a-3p	-0.1	-0.1	N/A
AKAP12	ENST00000402676.2	A kinase (PRKA) anchor protein 12	913	1	1	0	0	0	hsa-miR-23a-3p	-0.1	-0.1	N/A
PAK6	ENST00000260404.4	p21 protein (Cdc42/Rac)-activated kinase 6	70	1	1	0	0	0	hsa-miR-23a-3p	-0.1	-0.1	N/A
SIX2	ENST00000303077.6	SIX homeobox 2	75	1	0	0	1	0	hsa-miR-23a-3p	-0.1	-0.1	N/A
PPM1D	ENST00000305921.3	protein phosphatase, Mg2+/Mn2+ dependent, 1D	896	1	0	0	1	0	hsa-miR-23a-3p	-0.1	-0.11	N/A
TAB3	ENST00000378933.1	TGF-beta activated kinase 1/MAP3K7 binding protein 3	201	1	1	0	0	1	hsa-miR-23a-3p	-0.1	-0.11	N/A
PDE8B	ENST00000264917.5	phosphodiesterase 8B	186	1	0	1	0	0	hsa-miR-23a-3p	-0.1	-0.17	N/A
DCP2	ENST00000389063.2	decapping mRNA 2	155	1	0	1	0	2	hsa-miR-23a-3p	-0.1	-0.15	N/A
GABRB3	ENST00000311550.5	gamma-aminobutyric acid (GABA) A receptor, beta 3	267	1	0	0	1	1	hsa-miR-23a-3p	-0.1	-0.1	N/A
PHF17	ENST00000226319.6	PHD finger protein 17	1393	1	0	0	1	0	hsa-miR-23a-3p	-0.1	-0.1	N/A
CREG2	ENST00000324768.5	cellular repressor of E1A-stimulated genes 2	38	1	0	1	0	2	hsa-miR-23a-3p	-0.1	-0.12	N/A
RFFL	ENST00000315249.7	ring finger and FYVE-like domain containing E3 ubiquitin protein ligase	670	1	0	0	1	1	hsa-miR-23a-3p	-0.1	-0.12	N/A
PTPN4	ENST00000263708.2	protein tyrosine phosphatase, non-receptor type 4 (megakaryocyte)	215	2	1	0	1	3	hsa-miR-23a-3p	-0.1	-0.19	N/A
JARID2	ENST00000341776.2	jumonji, AT rich interactive domain 2	199	1	1	0	0	1	hsa-miR-23a-3p	-0.1	-0.1	N/A
PTK2B	ENST00000397501.1	protein tyrosine kinase 2 beta	163	1	0	1	0	0	hsa-miR-23a-3p	-0.1	-0.1	N/A
ZNF319	ENST00000299237.2	zinc finger protein 319	521	1	0	1	0	0	hsa-miR-23a-3p	-0.1	-0.1	N/A
NETO1	ENST00000327305.6	neuropilin (NRP) and tolloid (TLL)-like 1	5	1	0	0	1	0	hsa-miR-23a-3p	-0.09	-0.1	N/A
REPS2	ENST00000357277.3	RALBP1 associated Eps domain containing 2	36	2	1	0	1	1	hsa-miR-23a-3p	-0.09	-0.72	N/A
NUAK1	ENST00000261402.2	NUAK family, SNF1-like kinase, 1	489	1	1	0	0	0	hsa-miR-23a-3p	-0.09	-0.09	N/A

PBRM1	ENST00000356770.4	polybromo 1	689	1	1	0	0	1hsa-miR-23a-3p	-0.09	-0.09N/A
CADM3	ENST00000368125.4	cell adhesion molecule 3	5	1	1	0	0	1hsa-miR-23a-3p	-0.09	-0.09N/A
RBPMS2	ENST00000560606.1	RNA binding protein with multiple splicing 2	34	1	1	0	0	0hsa-miR-23a-3p	-0.09	-0.44N/A
CPEB3	ENST00000412050.4	cytoplasmic polyadenylation element binding protein 3	48	1	0	0	1	1hsa-miR-23a-3p	-0.09	-0.09N/A
YTHDF3	ENST00000539294.1	YTH domain family, member 3	444	1	0	0	1	0hsa-miR-23a-3p	-0.09	-0.09N/A
ZEB1	ENST00000361642.5	zinc finger E-box binding homeobox 1	59	1	0	1	0	0hsa-miR-23a-3p	-0.09	-0.09N/A
ZNF329	ENST00000598312.1	zinc finger protein 329	163	1	0	1	0	0hsa-miR-23a-3p	-0.09	-0.09N/A
EML4	ENST00000318522.5	echinoderm microtubule associated protein like 4	62	1	0	0	1	2hsa-miR-23a-3p	-0.09	-0.14N/A
SLC30A1	ENST00000367001.4	solute carrier family 30 (zinc transporter), member 1	831	1	1	0	0	0hsa-miR-23a-3p	-0.09	-0.13N/A
CPEB2	ENST00000538197.1	cytoplasmic polyadenylation element binding protein 2	367	1	1	0	0	1hsa-miR-23a-3p	-0.09	-0.09N/A
GCNT4	ENST00000322348.4	glucosaminyl (N-acetyl) transferase 4, core 2	19	1	1	0	0	0hsa-miR-23a-3p	-0.09	-0.12N/A
TET3	ENST00000409262.3	tet methylcytosine dioxygenase 3	1437	3	1	2	0	0hsa-miR-23a-3p	-0.09	-0.09N/A
PFKFB4	ENST00000232375.3	6-phosphofructo-2-kinase/fructose-2,6-biphosphatase 4	16	1	1	0	0	0hsa-miR-23a-3p	-0.09	-0.09N/A
PCDHA4	ENST00000530339.1	protocadherin alpha 4	86	1	0	0	1	0hsa-miR-23a-3p	-0.09	-0.1N/A
KSR1	ENST00000398988.3	kinase suppressor of ras 1	256	2	1	1	0	1hsa-miR-23a-3p	-0.09	-0.09N/A
WDFY1	ENST00000233055.4	WD repeat and FYVE domain containing 1	88	1	0	1	0	0hsa-miR-23a-3p	-0.09	-0.09N/A
EPN2	ENST00000314728.5	epsin 2	1609	1	0	1	0	1hsa-miR-23a-3p	-0.09	-0.09N/A
WISP1	ENST00000250160.6	WNT1 inducible signaling pathway protein 1	5	2	2	0	0	0hsa-miR-23a-3p	-0.09	-0.12N/A
CPEB4	ENST00000265085.5	cytoplasmic polyadenylation element binding protein 4	472	2	0	2	0	1hsa-miR-23a-3p	-0.09	-0.09N/A
PICALM	ENST00000532317.1	phosphatidylinositol binding clathrin assembly protein	1173	1	0	1	0	0hsa-miR-23a-3p	-0.09	-0.09N/A
MEFV	ENST00000541159.1	Mediterranean fever	5	1	0	1	0	0hsa-miR-23a-3p	-0.09	-0.09N/A
ADRBK1	ENST00000308595.5	adrenergic, beta, receptor kinase 1	1312	1	0	1	0	0hsa-miR-23a-3p	-0.08	-0.09N/A
FIGN	ENST00000333129.3	fidgetin	10	2	1	0	1	2hsa-miR-23a-3p	-0.08	-0.17N/A
KLHL5	ENST00000261425.3	kelch-like family member 5	366	1	0	0	1	1hsa-miR-23a-3p	-0.08	-0.14N/A
ARNT2	ENST00000303329.4	aryl-hydrocarbon receptor nuclear translocator 2	39	1	0	0	1	0hsa-miR-23a-3p	-0.08	-0.08N/A
PYGO1	ENST00000302000.6	pygopus homolog 1 (Drosophila)	104	1	0	0	1	2hsa-miR-23a-3p	-0.08	-0.12N/A
RYBP	ENST00000477973.2	RING1 and YY1 binding protein	342	1	0	1	0	0hsa-miR-23a-3p	-0.08	-0.12N/A
CELF1	ENST00000395290.2	CUGBP, Elav-like family member 1	153	3	1	2	0	1hsa-miR-23a-3p	-0.08	-0.12N/A
CTTNBP2NL	ENST00000271277.6	CTTNBP2 N-terminal like	182	1	0	1	0	0hsa-miR-23a-3p	-0.08	-0.08N/A
RP11-766F14.2	ENST00000511828.1	Protein LOC285556	5	2	2	0	0	0hsa-miR-23a-3p	-0.08	-0.08N/A
TCEB3	ENST00000418390.2	transcription elongation factor B (SIII), polypeptide 3 (110kDa, elongin A)	3007	1	0	0	1	2hsa-miR-23a-3p	-0.08	-0.08N/A
ELAVL4	ENST00000371824.1	ELAV like neuron-specific RNA binding protein 4	5	1	0	0	1	1hsa-miR-23a-3p	-0.08	-0.08N/A
MMS19	ENST00000327277.7	MMS19 nucleotide excision repair homolog (S. cerevisiae)	1062	1	0	0	1	1hsa-miR-23a-3p	-0.08	-0.09N/A
GJA9	ENST00000454994.2	gap junction protein, alpha 9, 59kDa	85	1	0	0	1	0hsa-miR-23a-3p	-0.08	-0.13N/A
RSBN1	ENST00000261441.5	round spermatid basic protein 1	54	2	0	1	1	1hsa-miR-23a-3p	-0.08	-0.08N/A
POP1	ENST00000401707.2	processing of precursor 1, ribonuclease P/MRP subunit (S. cerevisiae)	499	1	0	0	1	2hsa-miR-23a-3p	-0.08	-0.08N/A
CLK4	ENST00000316308.4	CDC-like kinase 4	218	1	0	1	0	0hsa-miR-23a-3p	-0.08	-0.2N/A
RUNX2	ENST00000371432.3	runt-related transcription factor 2	105	1	0	1	0	1hsa-miR-23a-3p	-0.08	-0.08N/A
ERBB4	ENST00000342788.4	v-erb-b2 avian erythroblastic leukemia viral oncogene homolog 4	27	1	1	0	0	0hsa-miR-23a-3p	-0.08	-0.09N/A
SLC25A53	ENST00000357421.4	solute carrier family 25, member 53	15	1	0	1	0	3hsa-miR-23a-3p	-0.08	-0.1N/A
SEPT3	ENST00000396425.3	septin 3	241	1	0	1	0	1hsa-miR-23a-3p	-0.08	-0.08N/A
MKX	ENST00000375790.5	mohawk homeobox	165	1	0	1	0	0hsa-miR-23a-3p	-0.07	-0.07N/A
FRMD5	ENST00000484674.1	FERM domain containing 5	161	1	1	0	0	0hsa-miR-23a-3p	-0.07	-0.21N/A
PPP6C	ENST00000373547.4	protein phosphatase 6, catalytic subunit	346	2	0	2	0	0hsa-miR-23a-3p	-0.07	-0.23N/A
MSI1	ENST00000257552.2	musashi RNA-binding protein 1	287	1	1	0	0	0hsa-miR-23a-3p	-0.07	-0.07N/A
CTNND2	ENST00000304623.8	catenin (cadherin-associated protein), delta 2	25	1	0	0	1	0hsa-miR-23a-3p	-0.07	-0.07N/A
TIGIT	ENST00000486257.1	T cell immunoreceptor with Ig and ITIM domains	5	1	1	0	0	0hsa-miR-23a-3p	-0.07	-0.07N/A
ATP11B	ENST00000323116.5	ATPase, class VI, type 11B	459	1	1	0	0	0hsa-miR-23a-3p	-0.07	-0.08N/A
SFXN5	ENST00000272433.2	sideroflexin 5	56	1	1	0	0	2hsa-miR-23a-3p	-0.07	-0.15N/A
ATXN1	ENST00000244769.4	ataxin 1	106	1	1	0	0	2hsa-miR-23a-3p	-0.07	-0.08N/A
EPS15	ENST00000371730.2	epidermal growth factor receptor pathway substrate 15	325	1	0	1	0	0hsa-miR-23a-3p	-0.07	-0.07N/A

NXF1	ENST00000531709.2	nuclear RNA export factor 1	1017	1	1	0	0	1	hsa-miR-23a-3p	-0.07	-0.07	N/A
HDAC7	ENST00000380610.4	histone deacetylase 7	56	1	1	0	0	0	hsa-miR-23a-3p	-0.07	-0.08	N/A
QSER1	ENST00000399302.2	glutamine and serine rich 1	34	1	1	0	0	0	hsa-miR-23a-3p	-0.07	-0.08	N/A
NFIA	ENST00000403491.3	nuclear factor I/A	5	3	1	2	0	0	hsa-miR-23a-3p	-0.07	-0.07	N/A
MUC19	ENST00000454784.4	mucin 19, oligomeric	5	1	0	1	0	0	hsa-miR-23a-3p	-0.07	-0.07	N/A
NDC1	ENST00000540001.1	NDC1 transmembrane nucleoporin	371	1	1	0	0	0	hsa-miR-23a-3p	-0.07	-0.24	N/A
UBN2	ENST00000473989.3	ubinuclein 2	238	2	1	1	0	4	hsa-miR-23a-3p	-0.07	-0.08	N/A
RAPGEF2	ENST00000264431.4	Rap guanine nucleotide exchange factor (GEF) 2	484	2	0	1	1	0	hsa-miR-23a-3p	-0.07	-0.07	N/A
SNX30	ENST00000374232.3	sorting nexin family member 30	33	1	0	0	1	1	hsa-miR-23a-3p	-0.07	-0.08	N/A
TXLNG	ENST00000380122.5	taxilin gamma	96	1	1	0	0	0	hsa-miR-23a-3p	-0.07	-0.27	N/A
ARL6IP1	ENST00000304414.7	ADP-ribosylation factor-like 6 interacting protein 1	25	1	0	1	0	0	hsa-miR-23a-3p	-0.07	-0.12	N/A
C1QL3	ENST00000298943.3	complement component 1, q subcomponent-like 3	77	1	0	0	1	0	hsa-miR-23a-3p	-0.07	-0.07	N/A
NKAP	ENST00000371410.3	NFKB activating protein	422	2	1	1	0	1	hsa-miR-23a-3p	-0.07	-0.13	N/A
HSPA12A	ENST00000369209.3	heat shock 70kDa protein 12A	37	1	1	0	0	2	hsa-miR-23a-3p	-0.07	-0.1	N/A
POU6F2	ENST00000518318.2	POU class 6 homeobox 2	5	1	1	0	0	4	hsa-miR-23a-3p	-0.07	-0.07	N/A
ATXN7	ENST00000295900.6	ataxin 7	264	1	1	0	0	2	hsa-miR-23a-3p	-0.07	-0.07	N/A
THSD7A	ENST00000423059.4	thrombospondin, type I, domain containing 7A	5	1	0	0	1	0	hsa-miR-23a-3p	-0.07	-0.07	N/A
CEBPA	ENST00000498907.2	CCAAT/enhancer binding protein (C/EBP), alpha	12449	1	1	0	0	0	hsa-miR-23a-3p	-0.07	-0.07	N/A
SPCS2	ENST00000263672.6	signal peptidase complex subunit 2 homolog (S. cerevisiae)	28	1	0	0	1	0	hsa-miR-23a-3p	-0.07	-0.07	N/A
CADM2	ENST00000383699.3	cell adhesion molecule 2	5	1	1	0	0	1	hsa-miR-23a-3p	-0.07	-0.07	N/A
IPO5	ENST00000261574.5	importin 5	1826	1	1	0	0	0	hsa-miR-23a-3p	-0.06	-0.07	N/A
GNG2	ENST00000556752.1	guanine nucleotide binding protein (G protein), gamma 2	199	1	0	1	0	0	hsa-miR-23a-3p	-0.06	-0.1	N/A
CCNL2	ENST00000408952.5	cyclin L2	699	1	0	1	0	0	hsa-miR-23a-3p	-0.06	-0.15	N/A
RAB35	ENST00000534951.1	RAB35, member RAS oncogene family	1051	1	0	0	1	0	hsa-miR-23a-3p	-0.06	-0.09	N/A
BCL9L	ENST00000334801.3	B-cell CLL/lymphoma 9-like	91	1	0	0	1	0	hsa-miR-23a-3p	-0.06	-0.12	N/A
TGFB2	ENST00000366930.4	transforming growth factor, beta 2	157	1	0	0	1	0	hsa-miR-23a-3p	-0.06	-0.06	N/A
PRR14L	ENST00000434485.1	proline rich 14-like	39	1	0	1	0	2	hsa-miR-23a-3p	-0.06	-0.06	N/A
KPNA3	ENST00000261667.3	karyopherin alpha 3 (importin alpha 4)	104	1	0	0	1	0	hsa-miR-23a-3p	-0.06	-0.25	N/A
PWWP2A	ENST00000456329.3	PWWP domain containing 2A	272	1	1	0	0	0	hsa-miR-23a-3p	-0.06	-0.06	N/A
PPP1R13B	ENST00000423488.2	protein phosphatase 1, regulatory subunit 13B	105	1	1	0	0	0	hsa-miR-23a-3p	-0.06	-0.06	N/A
MAT2A	ENST00000306434.3	methionine adenosyltransferase II, alpha	8	1	1	0	0	0	hsa-miR-23a-3p	-0.06	-0.1	N/A
GRM5	ENST00000418177.2	glutamate receptor, metabotropic 5	5	2	1	0	1	1	hsa-miR-23a-3p	-0.06	-0.06	N/A
SYNGAP1	ENST00000418600.2	synaptic Ras GTPase activating protein 1	15	1	0	0	1	0	hsa-miR-23a-3p	-0.06	-0.06	N/A
TARDBP	ENST00000240185.3	TAR DNA binding protein	410	1	1	0	0	1	hsa-miR-23a-3p	-0.06	-0.25	N/A
HELZ	ENST00000358691.5	helicase with zinc finger	571	1	0	1	0	2	hsa-miR-23a-3p	-0.06	-0.08	N/A
DAB2IP	ENST00000408936.3	DAB2 interacting protein	1787	1	0	0	1	0	hsa-miR-23a-3p	-0.06	-0.06	N/A
ZC3H6	ENST00000343936.4	zinc finger CCCH-type containing 6	10	1	1	0	0	0	hsa-miR-23a-3p	-0.06	-0.06	N/A
JAM3	ENST00000299106.4	junctional adhesion molecule 3	85	1	0	1	0	1	hsa-miR-23a-3p	-0.06	-0.06	N/A
PITPNA	ENST00000313486.7	phosphatidylinositol transfer protein, alpha	366	1	0	1	0	0	hsa-miR-23a-3p	-0.06	-0.14	N/A
EBF1	ENST00000313708.6	early B-cell factor 1	42	1	0	1	0	0	hsa-miR-23a-3p	-0.06	-0.07	N/A
NR2C1	ENST00000333003.5	nuclear receptor subfamily 2, group C, member 1	55	1	0	1	0	0	hsa-miR-23a-3p	-0.06	-0.18	N/A
LMOD3	ENST00000420581.2	leiomodin 3 (fetal)	5	1	1	0	0	0	hsa-miR-23a-3p	-0.06	-0.06	N/A
COX15	ENST00000016171.5	cytochrome c oxidase assembly homolog 15 (yeast)	984	2	1	1	0	1	hsa-miR-23a-3p	-0.06	-0.29	N/A
TNFSF13B	ENST00000375887.4	tumor necrosis factor (ligand) superfamily, member 13b	5	1	0	0	1	0	hsa-miR-23a-3p	-0.06	-0.06	N/A
MYO9A	ENST00000564571.1	myosin IXA	137	2	1	0	1	0	hsa-miR-23a-3p	-0.06	-0.06	N/A
RPTOR	ENST00000306801.3	regulatory associated protein of MTOR, complex 1	190	1	1	0	0	0	hsa-miR-23a-3p	-0.06	-0.06	N/A
EEA1	ENST00000322349.8	early endosome antigen 1	78	1	0	0	1	0	hsa-miR-23a-3p	-0.06	-0.07	N/A
RPRD2	ENST00000539519.1	regulation of nuclear pre-mRNA domain containing 2	280	2	2	0	0	2	hsa-miR-23a-3p	-0.06	-0.19	N/A
PDCD4	ENST00000280154.7	programmed cell death 4 (neoplastic transformation inhibitor)	612	1	0	0	1	0	hsa-miR-23a-3p	-0.05	-0.07	N/A
WAC	ENST00000375664.4	WW domain containing adaptor with coiled-coil	322	1	0	0	1	0	hsa-miR-23a-3p	-0.05	-0.05	N/A
PIP4K2B	ENST00000269554.3	phosphatidylinositol-5-phosphate 4-kinase, type II, beta	148	1	1	0	0	2	hsa-miR-23a-3p	-0.05	-0.33	N/A
STRN	ENST00000263918.4	striatin, calmodulin binding protein	476	2	2	0	0	2	hsa-miR-23a-3p	-0.05	-0.09	N/A
TET2	ENST00000545826.1	tet methylcytosine dioxygenase 2	558	1	0	1	0	1	hsa-miR-23a-3p	-0.05	-0.05	N/A
ZBTB44	ENST00000525842.1	zinc finger and BTB domain containing 44	236	2	2	0	0	0	hsa-miR-23a-3p	-0.05	-0.13	N/A
MED13L	ENST00000281928.3	mediator complex subunit 13-like	73	2	1	0	1	0	hsa-miR-23a-3p	-0.05	-0.1	N/A
GMPS	ENST00000496455.2	guanine monphosphate synthase	867	1	1	0	0	0	hsa-miR-23a-3p	-0.05	-0.14	N/A
HEPHL1	ENST00000315765.9	hephaestin-like 1	5	1	0	1	0	0	hsa-miR-23a-3p	-0.05	-0.05	N/A

TANGO2	ENST00000434570.2	transport and golgi organization 2 homolog (Drosophila)	119	1	0	1	0	1hsa-miR-23a-3p	-0.05	-0.07N/A
PRPF4B	ENST00000337659.6	pre-mRNA processing factor 4B	602	1	0	1	0	0hsa-miR-23a-3p	-0.05	-0.06N/A
DLG2	ENST00000398309.2	discs, large homolog 2 (Drosophila)	15	1	1	0	0	0hsa-miR-23a-3p	-0.05	-0.05N/A
FAM117B	ENST00000392238.2	family with sequence similarity 117, member B	245	1	1	0	0	4hsa-miR-23a-3p	-0.05	-0.05N/A
SLC4A4	ENST00000340595.3	solute carrier family 4 (sodium bicarbonate cotransporter), member 4	400	2	1	1	0	1hsa-miR-23a-3p	-0.05	-0.05N/A
XYLT1	ENST00000261381.6	xylosyltransferase I	159	1	0	1	0	2hsa-miR-23a-3p	-0.05	-0.06N/A
TENM1	ENST00000371130.3	teneurin transmembrane protein 1	5	2	0	2	0	0hsa-miR-23a-3p	-0.05	-0.05N/A
FZD4	ENST00000531380.1	frizzled family receptor 4	5	1	1	0	0	1hsa-miR-23a-3p	-0.05	-0.05N/A
EPN1	ENST00000270460.6	epsin 1	5464	1	0	0	1	0hsa-miR-23a-3p	-0.05	-0.05N/A
DNAH3	ENST00000415178.1	dynein, axonemal, heavy chain 3	5	1	0	1	0	1hsa-miR-23a-3p	-0.05	-0.05N/A
CLCN3	ENST00000513761.1	chloride channel, voltage-sensitive 3	1142	2	0	2	0	0hsa-miR-23a-3p	-0.05	-0.28N/A
NUDT3	ENST00000607016.1	nudix (nucleoside diphosphate linked moiety X)-type motif 3	190	1	0	0	1	2hsa-miR-23a-3p	-0.05	-0.12N/A
VCPIP1	ENST00000310421.4	valosin containing protein (p97)/p47 complex interacting protein 1	228	1	0	1	0	0hsa-miR-23a-3p	-0.05	-0.06N/A
BTBD7	ENST00000334746.5	BTB (POZ) domain containing 7	590	1	1	0	0	1hsa-miR-23a-3p	-0.05	-0.05N/A
TNRC6C	ENST00000335749.4	trinucleotide repeat containing 6C	248	2	1	1	0	0hsa-miR-23a-3p	-0.05	-0.05N/A
YIPF6	ENST00000462683.1	Yip1 domain family, member 6	888	1	0	1	0	1hsa-miR-23a-3p	-0.05	-0.18N/A
CNNM2	ENST00000369878.4	cyclin M2	517	1	0	1	0	1hsa-miR-23a-3p	-0.05	-0.08N/A
C20orf112	ENST00000359676.5	chromosome 20 open reading frame 112	148	1	1	0	0	1hsa-miR-23a-3p	-0.05	-0.11N/A
VMP1	ENST00000262291.4	vacuole membrane protein 1	2505	1	0	1	0	0hsa-miR-23a-3p	-0.05	-0.05N/A
ANTXR2	ENST00000403729.2	anthrax toxin receptor 2	51	1	0	0	1	1hsa-miR-23a-3p	-0.05	-0.11N/A
FAM134A	ENST00000430297.2	family with sequence similarity 134, member A	3080	1	1	0	0	0hsa-miR-23a-3p	-0.04	-0.16N/A
ARSB	ENST00000264914.4	arylsulfatase B	86	1	1	0	0	0hsa-miR-23a-3p	-0.04	-0.28N/A
ACVR2B	ENST00000352511.4	activin A receptor, type IIB	21	2	0	1	1	3hsa-miR-23a-3p	-0.04	-0.14N/A
PHACTR2	ENST00000427704.2	phosphatase and actin regulator 2	274	1	0	1	0	0hsa-miR-23a-3p	-0.04	-0.05N/A
CDKN2AIPNL	ENST00000458198.2	CDKN2A interacting protein N-terminal like	133	1	0	1	0	0hsa-miR-23a-3p	-0.04	-0.04N/A
ZNF469	ENST00000437464.1	zinc finger protein 469	139	1	1	0	0	0hsa-miR-23a-3p	-0.04	-0.04N/A
QKI	ENST00000392127.2	QKI, KH domain containing, RNA binding	323	1	0	1	0	5hsa-miR-23a-3p	-0.04	-0.09N/A
RNF7	ENST00000273480.3	ring finger protein 7	377	1	1	0	0	1hsa-miR-23a-3p	-0.04	-0.36N/A
SGK1	ENST00000367858.5	serum/glucocorticoid regulated kinase 1	369	1	0	1	0	0hsa-miR-23a-3p	-0.04	-0.04N/A
NT5DC3	ENST00000392876.3	5'-nucleotidase domain containing 3	235	1	0	0	1	2hsa-miR-23a-3p	-0.04	-0.04N/A
RUNX1T1	ENST00000523629.1	runt-related transcription factor 1; translocated to, 1 (cyclin D-related)	9	2	1	0	1	1hsa-miR-23a-3p	-0.04	-0.04N/A
AMER1	ENST00000330258.3	APC membrane recruitment protein 1	11	1	1	0	0	1hsa-miR-23a-3p	-0.04	-0.04N/A
UNC13A	ENST00000519716.2	unc-13 homolog A (C. elegans)	5	1	0	0	1	3hsa-miR-23a-3p	-0.04	-0.04N/A
ZKSCAN1	ENST00000324306.6	zinc finger with KRAB and SCAN domains 1	1314	1	0	1	0	2hsa-miR-23a-3p	-0.04	-0.04N/A
PCDH19	ENST00000420881.2	protocadherin 19	7	1	1	0	0	0hsa-miR-23a-3p	-0.04	-0.04N/A
ADAM23	ENST00000264377.3	ADAM metallopeptidase domain 23	113	1	1	0	0	1hsa-miR-23a-3p	-0.04	-0.09N/A
FREM1	ENST00000380881.4	FRAS1 related extracellular matrix 1	25	1	1	0	0	0hsa-miR-23a-3p	-0.04	-0.05N/A
DMBX1	ENST00000360032.3	diencephalon/mesencephalon homeobox 1	31	1	0	1	0	0hsa-miR-23a-3p	-0.04	-0.04N/A
CDK17	ENST00000543119.2	cyclin-dependent kinase 17	475	1	0	0	1	1hsa-miR-23a-3p	-0.04	-0.04N/A
STOX2	ENST00000308497.4	storkhead box 2	379	1	0	1	0	0hsa-miR-23a-3p	-0.04	-0.04N/A
ARID3B	ENST00000346246.5	AT rich interactive domain 3B (BRIGHT-like)	11	1	0	1	0	0hsa-miR-23a-3p	-0.04	-0.04N/A
PPP3CA	ENST00000512215.1	protein phosphatase 3, catalytic subunit, alpha isozyme	259	1	0	0	1	1hsa-miR-23a-3p	-0.04	-0.11N/A
CCDC6	ENST00000263102.6	coiled-coil domain containing 6	193	1	1	0	0	2hsa-miR-23a-3p	-0.04	-0.22N/A
EOGT	ENST00000383701.3	EGF domain-specific O-linked N-acetylglucosamine (GlcNAc) transferase	302	1	1	0	0	2hsa-miR-23a-3p	-0.04	-0.04N/A
EPG5	ENST00000282041.5	ectopic P-granules autophagy protein 5 homolog (C. elegans)	269	1	0	0	1	0hsa-miR-23a-3p	-0.04	-0.04N/A
PALD1	ENST00000263563.6	phosphatase domain containing, paladin 1	108	1	0	1	0	0hsa-miR-23a-3p	-0.04	-0.2N/A
SALL2	ENST00000327430.3	sal-like 2 (Drosophila)	233	1	1	0	0	1hsa-miR-23a-3p	-0.04	-0.04N/A
CYB561D1	ENST00000496961.1	cytochrome b561 family, member D1	105	1	0	1	0	0hsa-miR-23a-3p	-0.04	-0.04N/A
PTPN14	ENST00000366956.5	protein tyrosine phosphatase, non-receptor type 14	301	1	0	1	0	0hsa-miR-23a-3p	-0.04	-0.04N/A
PALLD	ENST00000261509.6	palladin, cytoskeletal associated protein	4423	1	0	1	0	0hsa-miR-23a-3p	-0.04	-0.04N/A
GBP1	ENST00000370473.4	guanylate binding protein 1, interferon-inducible	40	1	0	1	0	0hsa-miR-23a-3p	-0.04	-0.08N/A
IL17RD	ENST00000296318.7	interleukin 17 receptor D	108	1	1	0	0	0hsa-miR-23a-3p	-0.04	-0.04N/A
ADAM19	ENST00000257527.4	ADAM metallopeptidase domain 19	981	1	1	0	0	1hsa-miR-23a-3p	-0.04	-0.04N/A
CEP85L	ENST00000368491.3	centrosomal protein 85kDa-like	14	1	1	0	0	0hsa-miR-23a-3p	-0.04	-0.07N/A
PHYHIPL	ENST00000373880.4	phytanoyl-CoA 2-hydroxylase interacting protein-like	26	1	1	0	0	0hsa-miR-23a-3p	-0.04	-0.09N/A
NISCH	ENST00000345716.4	nischarin	120	1	1	0	0	0hsa-miR-23a-3p	-0.04	-0.04N/A
PARD6G	ENST00000353265.3	par-6 family cell polarity regulator gamma	172	1	0	1	0	0hsa-miR-23a-3p	-0.03	-0.04N/A

C3orf14	ENST00000494481.1	chromosome 3 open reading frame 14	67	1	1	0	0	0hsa-miR-23a-3p	-0.03	-0.17N/A
SHROOM2	ENST00000380913.3	shroom family member 2	121	1	1	0	0	1hsa-miR-23a-3p	-0.03	-0.17N/A
PIK3C2A	ENST00000265970.7	phosphatidylinositol-4-phosphate 3-kinase, catalytic subunit type 2 alpha	280	1	0	1	0	0hsa-miR-23a-3p	-0.03	-0.04N/A
FOXK1	ENST00000328914.4	forkhead box K1	368	1	0	1	0	0hsa-miR-23a-3p	-0.03	-0.05N/A
SLIT1	ENST00000266058.4	slit homolog 1 (Drosophila)	15	1	0	0	1	2hsa-miR-23a-3p	-0.03	-0.03N/A
CBFA2T2	ENST00000375279.2	core-binding factor, runt domain, alpha subunit 2; translocated to, 2	107	1	0	1	0	1hsa-miR-23a-3p	-0.03	-0.05N/A
THAP2	ENST00000308086.2	THAP domain containing, apoptosis associated protein 2	109	1	1	0	0	0hsa-miR-23a-3p	-0.03	-0.17N/A
KCNK1	ENST00000366621.3	potassium channel, subfamily K, member 1	410	1	1	0	0	3hsa-miR-23a-3p	-0.03	-0.08N/A
RUNX1	ENST00000344691.4	runt-related transcription factor 1	182	1	0	1	0	0hsa-miR-23a-3p	-0.03	-0.07N/A
LIMK2	ENST00000331728.4	LIM domain kinase 2	233	1	0	0	1	0hsa-miR-23a-3p	-0.03	-0.04N/A
SLC9A7	ENST00000328306.4	solute carrier family 9, subfamily A (NHE7, cation proton antiporter 7), member 7	147	1	1	0	0	1hsa-miR-23a-3p	-0.03	-0.07N/A
PCDHA5	ENST00000529859.1	protocadherin alpha 5	86	1	0	0	1	0hsa-miR-23a-3p	-0.03	-0.04N/A
PCDHA8	ENST00000531613.1	protocadherin alpha 8	86	1	0	0	1	0hsa-miR-23a-3p	-0.03	-0.04N/A
PCDHA10	ENST00000307360.5	protocadherin alpha 10	86	1	0	0	1	0hsa-miR-23a-3p	-0.03	-0.04N/A
PCDHA11	ENST00000398640.2	protocadherin alpha 11	86	1	0	0	1	0hsa-miR-23a-3p	-0.03	-0.04N/A
PCDHA1	ENST00000504120.2	protocadherin alpha 1	86	1	0	0	1	0hsa-miR-23a-3p	-0.03	-0.04N/A
PCDHA3	ENST00000522353.2	protocadherin alpha 3	86	1	0	0	1	0hsa-miR-23a-3p	-0.03	-0.04N/A
PCDHA2	ENST00000526136.1	protocadherin alpha 2	86	1	0	0	1	0hsa-miR-23a-3p	-0.03	-0.04N/A
PCDHAC1	ENST00000253807.2	protocadherin alpha subfamily C, 1	86	1	0	0	1	0hsa-miR-23a-3p	-0.03	-0.04N/A
PCDHA9	ENST00000532602.1	protocadherin alpha 9	86	1	0	0	1	0hsa-miR-23a-3p	-0.03	-0.04N/A
PCDHA13	ENST00000289272.2	protocadherin alpha 13	86	1	0	0	1	0hsa-miR-23a-3p	-0.03	-0.04N/A
PCDHA12	ENST00000398631.2	protocadherin alpha 12	86	1	0	0	1	0hsa-miR-23a-3p	-0.03	-0.04N/A
PCDHA7	ENST00000525929.1	protocadherin alpha 7	86	1	0	0	1	0hsa-miR-23a-3p	-0.03	-0.04N/A
PCDHA6	ENST00000529310.1	protocadherin alpha 6	86	1	0	0	1	0hsa-miR-23a-3p	-0.03	-0.04N/A
CHD7	ENST00000423902.2	chromodomain helicase DNA binding protein 7	925	1	1	0	0	0hsa-miR-23a-3p	-0.03	-0.05N/A
TGFBR3	ENST00000212355.4	transforming growth factor, beta receptor III	245	1	1	0	0	2hsa-miR-23a-3p	-0.03	-0.03N/A
NDST1	ENST00000261797.6	N-deacetylase/N-sulfotransferase (heparan glucosaminyl) 1	13660	1	1	0	0	1hsa-miR-23a-3p	-0.03	-0.03N/A
KCNB1	ENST00000371741.4	potassium voltage-gated channel, Shab-related subfamily, member 1	5	1	1	0	0	1hsa-miR-23a-3p	-0.03	-0.03N/A
SETD1B	ENST00000267197.5	SET domain containing 1B	703	1	1	0	0	0hsa-miR-23a-3p	-0.03	-0.03N/A
ZFHX3	ENST00000268489.5	zinc finger homeobox 3	57	1	1	0	0	3hsa-miR-23a-3p	-0.03	-0.03N/A
TECPR1	ENST00000447648.2	tectonin beta-propeller repeat containing 1	493	1	1	0	0	0hsa-miR-23a-3p	-0.03	-0.03N/A
ATXN7L3	ENST00000454077.2	ataxin 7-like 3	43	1	1	0	0	0hsa-miR-23a-3p	-0.03	-0.03N/A
NAA15	ENST00000296543.5	N(alpha)-acetyltransferase 15, NatA auxiliary subunit	546	1	1	0	0	0hsa-miR-23a-3p	-0.03	-0.03N/A
CCL22	ENST00000219235.4	chemokine (C-C motif) ligand 22	5	1	1	0	0	0hsa-miR-23a-3p	-0.03	-0.03N/A
TRRAP	ENST00000359863.4	transformation/transcription domain-associated protein	351	1	1	0	0	0hsa-miR-23a-3p	-0.03	-0.03N/A
HOXA11	ENST00000006015.3	homeobox A11	133	1	1	0	0	0hsa-miR-23a-3p	-0.03	-0.03N/A
ICMT	ENST00000343813.5	isoprenylcysteine carboxyl methyltransferase	558	1	1	0	0	0hsa-miR-23a-3p	-0.03	-0.03N/A
WDR31	ENST00000341761.4	WD repeat domain 31	23	1	1	0	0	0hsa-miR-23a-3p	-0.03	-0.03N/A
TRERF1	ENST00000541110.1	transcriptional regulating factor 1	52	1	1	0	0	1hsa-miR-23a-3p	-0.03	-0.03N/A
VCAN	ENST00000265077.3	versican	542	2	1	1	0	0hsa-miR-23a-3p	-0.03	-0.2N/A
VDR	ENST00000395324.2	vitamin D (1,25- dihydroxyvitamin D3) receptor	416	1	1	0	0	0hsa-miR-23a-3p	-0.03	-0.03N/A
SMAD3	ENST00000327367.4	SMAD family member 3	1140	1	0	1	0	0hsa-miR-23a-3p	-0.03	-0.04N/A
WASF2	ENST00000536657.1	WAS protein family, member 2	311	1	1	0	0	0hsa-miR-23a-3p	-0.03	-0.03N/A
MOGS	ENST00000409065.1	mannosyl-oligosaccharide glucosidase	184	1	1	0	0	0hsa-miR-23a-3p	-0.03	-0.03N/A
CKAP4	ENST00000378026.4	cytoskeleton-associated protein 4	1923	1	1	0	0	0hsa-miR-23a-3p	-0.03	-0.03N/A
DCUN1D3	ENST00000324344.4	DCN1, defective in cullin neddylation 1, domain containing 3	53	1	0	1	0	1hsa-miR-23a-3p	-0.03	-0.05N/A
SLC8A1	ENST00000406785.2	solute carrier family 8 (sodium/calcium exchanger), member 1	61	1	0	1	0	5hsa-miR-23a-3p	-0.03	-0.13N/A
COL4A1	ENST00000375820.4	collagen, type IV, alpha 1	6549	1	1	0	0	0hsa-miR-23a-3p	-0.03	-0.03N/A
TAOK1	ENST00000261716.3	TAO kinase 1	164	1	1	0	0	3hsa-miR-23a-3p	-0.03	-0.03N/A
PDE3A	ENST00000359062.3	phosphodiesterase 3A, cGMP-inhibited	1182	3	2	0	1	1hsa-miR-23a-3p	-0.03	-0.07N/A
PCDHAC2	ENST00000289269.5	protocadherin alpha subfamily C, 2	86	1	0	0	1	0hsa-miR-23a-3p	-0.03	-0.04N/A
COL27A1	ENST00000356083.3	collagen, type XXVII, alpha 1	620	1	0	0	1	1hsa-miR-23a-3p	-0.03	-0.03N/A
FZD5	ENST00000295417.3	frizzled family receptor 5	122	1	1	0	0	1hsa-miR-23a-3p	-0.03	-0.22N/A
PSD3	ENST00000327040.8	pleckstrin and Sec7 domain containing 3	133	1	0	1	0	0hsa-miR-23a-3p	-0.03	-0.05N/A
SH3PXD2A	ENST00000369774.4	SH3 and PX domains 2A	152	1	1	0	0	1hsa-miR-23a-3p	-0.03	-0.03N/A
SLC25A16	ENST00000609923.1	solute carrier family 25 (mitochondrial carrier; Graves disease autoantigen),	206	1	0	0	1	2hsa-miR-23a-3p	-0.03	-0.07N/A

		member 16								
MAP3K9	ENST00000554752.2	mitogen-activated protein kinase kinase kinase 9	44	1	1	0	0	0hsa-miR-23a-3p	-0.03	-0.03N/A
BBX	ENST00000415149.2	bobby sox homolog (Drosophila)	141	1	1	0	0	1hsa-miR-23a-3p	-0.02	-0.03N/A
FRMPD4	ENST00000380682.1	FERM and PDZ domain containing 4	25	1	0	0	1	0hsa-miR-23a-3p	-0.02	-0.02N/A
KCTD16	ENST00000507359.3	potassium channel tetramerization domain containing 16	36	1	0	1	0	1hsa-miR-23a-3p	-0.02	-0.14N/A
NLK	ENST00000407008.3	nemo-like kinase	61	1	0	1	0	0hsa-miR-23a-3p	-0.02	-0.2N/A
RAD9B	ENST00000409425.1	RAD9 homolog B (S. pombe)	10	1	0	1	0	1hsa-miR-23a-3p	-0.02	-0.15N/A
ZNF226	ENST00000588883.1	zinc finger protein 226	327	7	2	5	0	0hsa-miR-23a-3p	-0.02	-1.17N/A
IPMK	ENST00000373935.3	inositol polyphosphate multikinase	196	2	0	1	1	1hsa-miR-23a-3p	-0.02	-0.38N/A
SIM2	ENST00000290399.6	single-minded homolog 2 (Drosophila)	108	1	1	0	0	0hsa-miR-23a-3p	-0.02	-0.1N/A
USP6NL	ENST00000609104.1	USP6 N-terminal like	183	1	0	1	0	3hsa-miR-23a-3p	-0.02	-0.08N/A
NFIX	ENST00000360105.4	nuclear factor I/X (CCAAT-binding transcription factor)	24	1	0	0	1	0hsa-miR-23a-3p	-0.02	-0.11N/A
ASAP1	ENST00000357668.1	ArfGAP with SH3 domain, ankyrin repeat and PH domain 1	185	1	0	1	0	0hsa-miR-23a-3p	-0.02	-0.03N/A
GGCX	ENST00000233838.4	gamma-glutamyl carboxylase	254	1	1	0	0	0hsa-miR-23a-3p	-0.02	-0.03N/A
SOGA3	ENST00000556132.1	SOGA family member 3	95	1	0	1	0	2hsa-miR-23a-3p	-0.02	-0.04N/A
TMEM120B	ENST00000449592.2	transmembrane protein 120B	60	1	0	1	0	0hsa-miR-23a-3p	-0.02	-0.03N/A
FTO	ENST00000471389.1	fat mass and obesity associated	240	1	0	0	1	0hsa-miR-23a-3p	-0.02	-0.03N/A
PAPOLG	ENST00000238714.3	poly(A) polymerase gamma	49	1	0	1	0	0hsa-miR-23a-3p	-0.02	-0.02N/A
KMT2D	ENST00000301067.7	lysine (K)-specific methyltransferase 2D	78	1	0	1	0	0hsa-miR-23a-3p	-0.02	-0.02N/A
ZNRF3	ENST00000544604.2	zinc and ring finger 3	179	1	0	1	0	2hsa-miR-23a-3p	-0.02	-0.02N/A
SNX22	ENST00000325881.4	sorting nexin 22	5	1	0	1	0	0hsa-miR-23a-3p	-0.02	-0.02N/A
ZC3H12B	ENST00000338957.4	zinc finger CCCH-type containing 12B	29	1	0	1	0	0hsa-miR-23a-3p	-0.02	-0.02N/A
GABRB2	ENST00000393959.1	gamma-aminobutyric acid (GABA) A receptor, beta 2	5	1	0	1	0	0hsa-miR-23a-3p	-0.02	-0.02N/A
MAP3K3	ENST00000361357.3	mitogen-activated protein kinase kinase kinase 3	144	1	0	1	0	0hsa-miR-23a-3p	-0.02	-0.02N/A
KCNJ15	ENST00000328656.4	potassium inwardly-rectifying channel, subfamily J, member 15	5	1	0	1	0	0hsa-miR-23a-3p	-0.02	-0.02N/A
MGAT3	ENST00000341184.6	mannosyl (beta-1,4-)-glycoprotein beta-1,4-N-acetylglucosaminyltransferase	9	1	0	1	0	0hsa-miR-23a-3p	-0.02	-0.02N/A
FBXO10	ENST00000432825.2	F-box protein 10	14	1	0	1	0	1hsa-miR-23a-3p	-0.02	-0.02N/A
SRRM4	ENST00000267260.4	serine/arginine repetitive matrix 4	22	1	0	1	0	0hsa-miR-23a-3p	-0.02	-0.02N/A
GCC1	ENST00000321407.2	GRIP and coiled-coil domain containing 1	26	1	0	1	0	1hsa-miR-23a-3p	-0.02	-0.02N/A
ANKRD52	ENST00000267116.7	ankyrin repeat domain 52	2913	1	0	1	0	0hsa-miR-23a-3p	-0.02	-0.02N/A
PRPF40A	ENST00000410080.1	PRP40 pre-mRNA processing factor 40 homolog A (S. cerevisiae)	459	1	1	0	0	0hsa-miR-23a-3p	-0.02	-0.05N/A
JAG1	ENST00000254958.5	jagged 1	421	1	0	1	0	0hsa-miR-23a-3p	-0.02	-0.02N/A
ALDH1L2	ENST00000258494.9	aldehyde dehydrogenase 1 family, member L2	22	1	1	0	0	1hsa-miR-23a-3p	-0.02	-0.03N/A
TRIM44	ENST00000299413.5	tripartite motif containing 44	4203	1	0	1	0	4hsa-miR-23a-3p	-0.02	-0.07N/A
USP24	ENST00000294383.6	ubiquitin specific peptidase 24	306	1	0	1	0	1hsa-miR-23a-3p	-0.02	-0.07N/A
FNIP2	ENST00000264433.6	folliculin interacting protein 2	266	1	1	0	0	0hsa-miR-23a-3p	-0.02	-0.03N/A
PGR	ENST00000325455.5	progesterone receptor	8	1	1	0	0	1hsa-miR-23a-3p	-0.02	-0.03N/A
IKZF3	ENST00000346872.3	IKAROS family zinc finger 3 (Aiolos)	7	2	1	1	0	2hsa-miR-23a-3p	-0.02	-0.06N/A
ARHGEF5	ENST00000056217.5	Rho guanine nucleotide exchange factor (GEF) 5	200	1	0	1	0	1hsa-miR-23a-3p	-0.02	-0.02N/A
MARCH6	ENST00000274140.5	membrane-associated ring finger (C3HC4) 6, E3 ubiquitin protein ligase	14086	1	0	1	0	0hsa-miR-23a-3p	-0.02	-0.04N/A
LGALS8	ENST00000526589.1	lectin, galactoside-binding, soluble, 8	188	1	1	0	0	0hsa-miR-23a-3p	-0.02	-0.16N/A
ITGA1	ENST00000282588.6	integrin, alpha 1	400	1	0	1	0	2hsa-miR-23a-3p	-0.02	-0.1N/A
MARCH9	ENST00000266643.5	membrane-associated ring finger (C3HC4) 9	328	1	1	0	0	0hsa-miR-23a-3p	-0.02	-0.17N/A
TRIM24	ENST00000343526.4	tripartite motif containing 24	182	1	1	0	0	2hsa-miR-23a-3p	-0.02	-0.08N/A
TNIK	ENST00000436636.2	TRAF2 and NCK interacting kinase	106	1	0	1	0	0hsa-miR-23a-3p	-0.02	-0.04N/A
STK38L	ENST00000389032.3	serine/threonine kinase 38 like	705	1	0	1	0	0hsa-miR-23a-3p	-0.02	-0.07N/A
AFF1	ENST00000395146.4	AF4/FMR2 family, member 1	28	1	0	1	0	1hsa-miR-23a-3p	-0.02	-0.04N/A
BICD2	ENST00000356884.6	bicaudal D homolog 2 (Drosophila)	38	1	1	0	0	0hsa-miR-23a-3p	-0.02	-0.04N/A
TRABD2B	ENST00000606738.2	TraB domain containing 2B	244	1	0	1	0	1hsa-miR-23a-3p	-0.02	-0.02N/A
GCA	ENST00000437150.2	grancalcin, EF-hand calcium binding protein	177	1	0	1	0	1hsa-miR-23a-3p	-0.01	-0.27N/A
TRPS1	ENST00000395715.3	trichorhinophalangeal syndrome I	94	1	1	0	0	1hsa-miR-23a-3p	-0.01	-0.03N/A
TENM4	ENST00000278550.7	teneurin transmembrane protein 4	34	1	0	1	0	2hsa-miR-23a-3p	-0.01	-0.08N/A
OSBPL8	ENST00000393249.2	oxysterol binding protein-like 8	141	2	1	0	1	0hsa-miR-23a-3p	-0.01	-0.15N/A
CBLB	ENST00000264122.4	Cbl proto-oncogene B, E3 ubiquitin protein ligase	469	1	0	1	0	0hsa-miR-23a-3p	-0.01	-0.08N/A
KIAA1468	ENST00000398130.2	KIAA1468	756	1	0	1	0	0hsa-miR-23a-3p	-0.01	-0.02N/A
SETX	ENST00000224140.5	senataxin	270	1	0	0	1	0hsa-miR-23a-3p	-0.01	-0.04N/A
RBM27	ENST00000265271.5	RNA binding motif protein 27	130	1	0	0	1	0hsa-miR-23a-3p	-0.01	-0.05N/A

SP1	ENST00000426431.2	Sp1 transcription factor	402	1	1	0	0	0hsa-miR-23a-3p	-0.01	-0.03N/A
EML5	ENST00000554922.1	echinoderm microtubule associated protein like 5	60	1	0	1	0	0hsa-miR-23a-3p	-0.01	-0.02N/A
EPC1	ENST00000319778.6	enhancer of polycomb homolog 1 (Drosophila)	102	1	0	0	1	1hsa-miR-23a-3p	-0.01	-0.05N/A
PDE3B	ENST00000282096.4	phosphodiesterase 3B, cGMP-inhibited	258	1	0	1	0	1hsa-miR-23a-3p	-0.01	-0.02N/A
PKNOX1	ENST00000291547.5	PBX/knotted 1 homeobox 1	297	1	1	0	0	0hsa-miR-23a-3p	-0.01	-0.4N/A
PBX1	ENST00000420696.2	pre-B-cell leukemia homeobox 1	18	1	0	0	1	0hsa-miR-23a-3p	-0.01	-0.02N/A
KIF1C	ENST00000320785.5	kinesin family member 1C	149	1	0	1	0	1hsa-miR-23a-3p	-0.01	-0.02N/A
COX16	ENST00000389912.6	COX16 cytochrome c oxidase assembly homolog (S. cerevisiae)	4087	1	1	0	0	0hsa-miR-23a-3p	-0.01	-0.6N/A
MYO6	ENST00000369981.3	myosin VI	297	1	0	1	0	0hsa-miR-23a-3p	-0.01	-0.05N/A
FAM46C	ENST00000369448.3	family with sequence similarity 46, member C	74	1	0	1	0	0hsa-miR-23a-3p	-0.01	-0.04N/A
PURA	ENST00000331327.3	purine-rich element binding protein A	367	1	0	1	0	0hsa-miR-23a-3p	-0.01	-0.04N/A
CASD1	ENST00000297273.4	CAS1 domain containing 1	270	1	0	0	1	0hsa-miR-23a-3p	-0.01	-0.01N/A
CHST15	ENST00000346248.5	carbohydrate (N-acetylgalactosamine 4-sulfate 6-O) sulfotransferase 15	59	1	0	0	1	0hsa-miR-23a-3p	-0.01	-0.01N/A
SIK2	ENST00000304987.3	salt-inducible kinase 2	378	2	1	0	1	0hsa-miR-23a-3p	-0.01	-0.06N/A
FAM114A2	ENST00000351797.4	family with sequence similarity 114, member A2	385	1	1	0	0	0hsa-miR-23a-3p	-0.01	-0.26N/A
MAGI1	ENST00000402939.2	membrane associated guanylate kinase, WW and PDZ domain containing 1	253	1	0	1	0	0hsa-miR-23a-3p	-0.01	-0.02N/A
USP30	ENST00000257548.5	ubiquitin specific peptidase 30	76	1	1	0	0	1hsa-miR-23a-3p	-0.01	-0.07N/A
TMEM200C	ENST00000581347.2	transmembrane protein 200C	54	1	0	1	0	0hsa-miR-23a-3p	-0.01	-0.09N/A
SHB	ENST00000377707.3	Src homology 2 domain containing adaptor protein B	1436	1	1	0	0	2hsa-miR-23a-3p	-0.01	-0.24N/A
SEPSECS	ENST00000302922.3	Sep (O-phosphoserine) tRNA:Sec (selenocysteine) tRNA synthase	279	1	0	0	1	2hsa-miR-23a-3p	-0.01	-0.01N/A
BMPR2	ENST00000374574.2	bone morphogenetic protein receptor, type II (serine/threonine kinase)	287	1	1	0	0	1hsa-miR-23a-3p	-0.01	-0.03N/A
THRB	ENST00000396671.2	thyroid hormone receptor, beta	55	1	0	0	1	1hsa-miR-23a-3p	-0.01	-0.01N/A
WIPF2	ENST00000323571.4	WAS/WASL interacting protein family, member 2	214	1	0	1	0	1hsa-miR-23a-3p	-0.01	-0.02N/A
TANC2	ENST00000424789.2	tetratricopeptide repeat, ankyrin repeat and coiled-coil containing 2	155	1	0	0	1	1hsa-miR-23a-3p	-0.01	-0.01N/A
SRGAP1	ENST00000355086.3	SLIT-ROBO Rho GTPase activating protein 1	64	1	0	0	1	1hsa-miR-23a-3p	-0.01	-0.01N/A
ZNHIT6	ENST00000431532.2	zinc finger, HIT-type containing 6	201	1	0	1	0	1hsa-miR-23a-3p	-0.01	-0.02N/A
PLCG2	ENST00000359376.3	phospholipase C, gamma 2 (phosphatidylinositol-specific)	23	1	1	0	0	2hsa-miR-23a-3p	-0.01	-0.07N/A
STXBP5	ENST00000367481.3	syntaxin binding protein 5 (tomosyn)	555	1	1	0	0	2hsa-miR-23a-3p	-0.01	-0.04N/A
SCAI	ENST00000336505.6	suppressor of cancer cell invasion	74	1	0	0	1	1hsa-miR-23a-3p	-0.01	-0.03N/A
DNMT3A	ENST00000380746.4	DNA (cytosine-5-)-methyltransferase 3 alpha	221	1	0	1	0	0hsa-miR-23a-3p	-0.01	-0.02N/A
POLR1E	ENST00000377798.4	polymerase (RNA) I polypeptide E, 53kDa	540	1	1	0	0	1hsa-miR-23a-3p	0	-0.13N/A
SLC5A3	ENST00000608209.1	sodium/myo-inositol cotransporter	24	1	0	1	0	3hsa-miR-23a-3p	0	-0.02N/A
SLC5A3	ENST00000381151.3	solute carrier family 5 (sodium/myo-inositol cotransporter), member 3	24	1	0	1	0	3hsa-miR-23a-3p	0	-0.02N/A
SNX6	ENST00000396526.3	sorting nexin 6	254	1	1	0	0	1hsa-miR-23a-3p	0	-0.23N/A
CRTAP	ENST00000320954.6	cartilage associated protein	3154	1	0	1	0	0hsa-miR-23a-3p	0	-0.04N/A
CCDC104	ENST00000349456.4	coiled-coil domain containing 104	112	1	0	1	0	0hsa-miR-23a-3p	0	-0.08N/A
RPS6KA3	ENST00000379565.3	ribosomal protein S6 kinase, 90kDa, polypeptide 3	769	1	0	1	0	1hsa-miR-23a-3p	0	-0.02N/A
MAPRE2	ENST00000436190.2	microtubule-associated protein, RP/EB family, member 2	361	1	0	0	1	0hsa-miR-23a-3p	0	-0.1N/A
ADCYAP1	ENST00000579794.1	adenylate cyclase activating polypeptide 1 (pituitary)	35	1	0	1	0	0hsa-miR-23a-3p	0	-0.02N/A
GAPVD1	ENST00000470056.1	GTPase activating protein and VPS9 domains 1	1805	1	0	1	0	2hsa-miR-23a-3p	0	-0.05N/A
PRRC2B	ENST00000372249.1	proline-rich coiled-coil 2B	97	1	0	0	1	2hsa-miR-23a-3p	0	-0.09N/A
C9orf91	ENST00000374049.4	chromosome 9 open reading frame 91	151	1	0	1	0	0hsa-miR-23a-3p	0	-0.02N/A
TK2	ENST00000299697.7	thymidine kinase 2, mitochondrial	439	1	0	1	0	0hsa-miR-23a-3p	0	-0.02N/A
NCKAP1	ENST00000361354.4	NCK-associated protein 1	700	1	0	0	1	3hsa-miR-23a-3p	0	-0.18N/A
DZIP1	ENST00000347108.3	DAZ interacting zinc finger protein 1	360	1	0	1	0	0hsa-miR-23a-3p	0	-0.03N/A
USP31	ENST00000219689.7	ubiquitin specific peptidase 31	214	1	1	0	0	2hsa-miR-23a-3p	0	-0.03N/A
MED28	ENST00000237380.7	mediator complex subunit 28	1483	1	1	0	0	0hsa-miR-23a-3p	0	-0.09N/A
BNC2	ENST00000380672.4	basonuclin 2	770	1	1	0	0	1hsa-miR-23a-3p	0	-0.07N/A
SNAP29	ENST00000215730.7	synaptosomal-associated protein, 29kDa	766	1	1	0	0	1hsa-miR-23a-3p	0	-0.31N/A
NPC2	ENST00000541064.1	Niemann-Pick disease, type C2	1152	1	1	0	0	0hsa-miR-23a-3p	0	-0.35N/A
CDK13	ENST00000181839.4	cyclin-dependent kinase 13	32	1	1	0	0	1hsa-miR-23a-3p	0	-0.03N/A
SLC30A9	ENST00000264451.7	solute carrier family 30 (zinc transporter), member 9	396	1	0	1	0	0hsa-miR-23a-3p	0	-0.1N/A
NAP1L1	ENST00000261182.8	nucleosome assembly protein 1-like 1	9072	1	1	0	0	0hsa-miR-23a-3p	0	-0.04N/A
EXOC3L4	ENST00000380069.3	exocyst complex component 3-like 4	5	1	1	0	0	0hsa-miR-23a-3p	0	-0.53N/A
COLEC10	ENST00000332843.2	collectin sub-family member 10 (C-type lectin)	5	1	0	0	1	1hsa-miR-23a-3p	0	-0.4N/A
GTPBP10	ENST00000222511.6	GTP-binding protein 10 (putative)	489	1	0	0	1	1hsa-miR-23a-3p	0	-0.01N/A
XRCC5	ENST00000392133.3	X-ray repair complementing defective repair in Chinese hamster cells 5	14802	1	1	0	0	3hsa-miR-23a-3p	0	-0.05N/A

		(double-strand-break rejoining)								
PCF11	ENST00000298281.4	PCF11 cleavage and polyadenylation factor subunit	218	1	0	1	0	0hsa-miR-23a-3p	0	-0.02N/A
VCP	ENST00000358901.6	valosin containing protein	5387	1	1	0	0	0hsa-miR-23a-3p	0	-0.16N/A
HM13	ENST00000335574.5	histocompatibility (minor) 13	4848	1	0	0	1	0hsa-miR-23a-3p	0	-0.02N/A
SEC31B	ENST00000370345.3	SEC31 homolog B (S. cerevisiae)	43	1	0	0	1	1hsa-miR-23a-3p	0	-0.01N/A
IL1RAPL1	ENST00000378993.1	interleukin 1 receptor accessory protein-like 1	5	1	0	1	0	2hsa-miR-23a-3p	0	-0.17N/A
CEP128	ENST00000281129.3	centrosomal protein 128kDa	280	1	1	0	0	1hsa-miR-23a-3p	0	-0.04N/A
ANKRD32	ENST00000265140.5	ankyrin repeat domain 32	2982	1	1	0	0	1hsa-miR-23a-3p	0	-0.06N/A
DDX5	ENST00000225792.5	DEAD (Asp-Glu-Ala-Asp) box helicase 5	5537	1	0	1	0	0hsa-miR-23a-3p	0	-0.2N/A
SLC2A4RG	ENST00000266077.2	SLC2A4 regulator	2555	1	1	0	0	0hsa-miR-23a-3p	0	-0.33N/A
ZBED3	ENST00000255198.2	zinc finger, BED-type containing 3	399	1	0	0	1	0hsa-miR-23a-3p	0	-0.01N/A
GABRB1	ENST00000295454.3	gamma-aminobutyric acid (GABA) A receptor, beta 1	5	1	1	0	0	1hsa-miR-23a-3p	0	-0.08N/A
KCNH1	ENST00000271751.4	potassium voltage-gated channel, subfamily H (eag-related), member 1	5	1	1	0	0	0hsa-miR-23a-3p	0	-0.03N/A
SLFN12L	ENST00000260908.7	schlafen family member 12-like	5	1	0	1	0	1hsa-miR-23a-3p	0	-0.06N/A
CYB5R1	ENST00000367249.4	cytochrome b5 reductase 1	545	1	0	1	0	0hsa-miR-23a-3p	0	-0.02N/A
PPL	ENST00000345988.2	periplakin	849	1	1	0	0	0hsa-miR-23a-3p	0	-0.03N/A
DNAJC15	ENST00000379221.2	DnaJ (Hsp40) homolog, subfamily C, member 15	3290	1	1	0	0	3hsa-miR-23a-3p	0	-0.64N/A
SGIP1	ENST00000371036.3	SH3-domain GRB2-like (endophilin) interacting protein 1	180	1	0	1	0	0hsa-miR-23a-3p	0	-0.06N/A
TRPM3	ENST00000377111.2	transient receptor potential cation channel, subfamily M, member 3	11	1	0	1	0	0hsa-miR-23a-3p	0	-0.02N/A
CIRH1A	ENST00000314423.7	cirrhosis, autosomal recessive 1A (cirhin)	1015	1	0	1	0	1hsa-miR-23a-3p	0	-0.08N/A
GHR	ENST00000230882.4	growth hormone receptor	102	1	1	0	0	1hsa-miR-23a-3p	0	-0.03N/A
GCC2	ENST00000309863.6	GRIP and coiled-coil domain containing 2	5	2	2	0	0	1hsa-miR-27a-3p	-1.08	-1.08N/A
PLK2	ENST00000274289.3	polo-like kinase 2	689	3	2	1	0	0hsa-miR-27a-3p	-1.01	-1.01N/A
AKIRIN1	ENST00000432648.3	akirin 1	798	4	0	3	1	0hsa-miR-27a-3p	-0.88	-0.97N/A
NOVA1	ENST00000465357.2	neuro-oncological ventral antigen 1	5	2	1	1	0	1hsa-miR-27a-3p	-0.87	-0.87N/A
SZRD1	ENST00000401089.3	SUZ RNA binding domain containing 1	137	4	0	4	0	0hsa-miR-27a-3p	-0.84	-0.84N/A
ROR1	ENST00000371079.1	receptor tyrosine kinase-like orphan receptor 1	80	2	1	0	1	0hsa-miR-27a-3p	-0.84	-0.84N/A
ONECUT2	ENST00000491143.2	one cut homeobox 2	847	4	1	2	1	1hsa-miR-27a-3p	-0.81	-0.85N/A
HOXA5	ENST00000222726.3	homeobox A5	63	3	0	2	1	1hsa-miR-27a-3p	-0.79	-0.79N/A
CCNC	ENST00000520371.1	cyclin C	524	1	1	0	0	0hsa-miR-27a-3p	-0.78	-0.79N/A
SNAP25	ENST00000254976.2	synaptosomal-associated protein, 25kDa	54	3	0	2	1	0hsa-miR-27a-3p	-0.77	-0.86N/A
PXN	ENST00000458477.2	paxillin	484	1	0	1	0	3hsa-miR-27a-3p	-0.72	-0.74N/A
NGFRAP1	ENST00000361298.4	nerve growth factor receptor (TNFRSF16) associated protein 1	114	2	0	1	1	0hsa-miR-27a-3p	-0.7	-0.83N/A
CSRP2	ENST00000311083.5	cysteine and glycine-rich protein 2	38	1	1	0	0	0hsa-miR-27a-3p	-0.69	-0.69N/A
FBXW7	ENST00000281708.4	F-box and WD repeat domain containing 7, E3 ubiquitin protein ligase	12	2	2	0	0	2hsa-miR-27a-3p	-0.67	-0.67N/A
NDUFS4	ENST00000296684.5	NADH dehydrogenase (ubiquinone) Fe-S protein 4, 18kDa (NADH-coenzyme Q reductase)	125	1	1	0	0	0hsa-miR-27a-3p	-0.67	-0.67N/A
TNPO1	ENST00000337273.5	transportin 1	2041	3	1	1	1	1hsa-miR-27a-3p	-0.67	-0.82N/A
STAB2	ENST00000388887.2	stabilin 2	5	1	1	0	0	0hsa-miR-27a-3p	-0.67	-0.67N/A
AQP11	ENST00000313578.3	aquaporin 11	75	1	1	0	0	0hsa-miR-27a-3p	-0.67	-0.67N/A
SLC6A1	ENST00000287766.4	solute carrier family 6 (neurotransmitter transporter), member 1	5	3	1	2	0	0hsa-miR-27a-3p	-0.66	-0.66N/A
DCUN1D4	ENST00000334635.5	DCN1, defective in cullin neddylation 1, domain containing 4	1118	2	1	1	0	1hsa-miR-27a-3p	-0.65	-0.7N/A
PKIA	ENST00000396418.2	protein kinase (cAMP-dependent, catalytic) inhibitor alpha	542	1	1	0	0	0hsa-miR-27a-3p	-0.64	-0.85N/A
ADORA2B	ENST00000304222.2	adenosine A2b receptor	145	1	1	0	0	0hsa-miR-27a-3p	-0.64	-0.64N/A
PDIA5	ENST00000316218.7	protein disulfide isomerase family A, member 5	10871	1	1	0	0	0hsa-miR-27a-3p	-0.63	-0.63N/A
TRAF3IP3	ENST00000367023.1	TRAF3 interacting protein 3	5	1	1	0	0	0hsa-miR-27a-3p	-0.62	-0.62N/A
NABP1	ENST00000410026.2	nucleic acid binding protein 1	938	1	1	0	0	1hsa-miR-27a-3p	-0.62	-0.67N/A
C1orf52	ENST00000471115.1	chromosome 1 open reading frame 52	1516	1	1	0	0	1hsa-miR-27a-3p	-0.61	-0.63N/A
ZIC5	ENST00000267294.4	Zic family member 5	263	2	1	1	0	1hsa-miR-27a-3p	-0.61	-0.66N/A
GOLM1	ENST00000388712.3	golgi membrane protein 1	4212	1	1	0	0	0hsa-miR-27a-3p	-0.6	-0.6N/A
C8orf4	ENST00000315792.3	chromosome 8 open reading frame 4	3820	1	1	0	0	1hsa-miR-27a-3p	-0.59	-0.64N/A
TMSB10	ENST00000233143.4	thymosin beta 10	49918	1	1	0	0	0hsa-miR-27a-3p	-0.59	-0.59N/A
FLRT3	ENST00000378053.3	fibronectin leucine rich transmembrane protein 3	11	1	1	0	0	0hsa-miR-27a-3p	-0.59	-0.72N/A
FAM222B	ENST00000582266.1	family with sequence similarity 222, member B	100	1	1	0	0	0hsa-miR-27a-3p	-0.59	-0.67N/A
GATC	ENST00000551765.1	glutamyl-tRNA(Gln) amidotransferase, subunit C	781	1	1	0	0	0hsa-miR-27a-3p	-0.58	-0.67N/A
SFXN2	ENST00000369893.5	sideroflexin 2	547	2	1	1	0	0hsa-miR-27a-3p	-0.58	-0.58N/A

ACTA2	ENST00000224784.6	actin, alpha 2, smooth muscle, aorta	8771	1	1	0	0	0	hsa-miR-27a-3p	-0.58	-0.58	N/A
TRIM23	ENST00000231524.9	tripartite motif containing 23	247	1	1	0	0	0	hsa-miR-27a-3p	-0.58	-0.58	N/A
MMD	ENST00000262065.3	monocyte to macrophage differentiation-associated	558	1	1	0	0	0	hsa-miR-27a-3p	-0.57	-0.57	N/A
NR2F6	ENST00000291442.3	nuclear receptor subfamily 2, group F, member 6	814	1	1	0	0	1	hsa-miR-27a-3p	-0.57	-0.6	N/A
KATNBL1	ENST00000256544.3	katanin p80 subunit B-like 1	105	1	1	0	0	1	hsa-miR-27a-3p	-0.57	-0.85	N/A
B4GALT3	ENST00000319769.5	UDP-Gal:betaGlcNAc beta 1,4- galactosyltransferase, polypeptide 3	46	1	1	0	0	0	hsa-miR-27a-3p	-0.56	-0.56	N/A
REPS1	ENST00000450536.2	RALBP1 associated Eps domain containing 1	725	1	1	0	0	0	hsa-miR-27a-3p	-0.56	-0.56	N/A
CACNA2D3	ENST00000474759.1	calcium channel, voltage-dependent, alpha 2/delta subunit 3	8	1	1	0	0	0	hsa-miR-27a-3p	-0.56	-0.56	N/A
TMUB1	ENST00000297533.4	transmembrane and ubiquitin-like domain containing 1	79	1	1	0	0	0	hsa-miR-27a-3p	-0.56	-0.56	N/A
RREB1	ENST00000379938.2	ras responsive element binding protein 1	340	2	1	0	1	3	hsa-miR-27a-3p	-0.56	-0.56	N/A
GCA	ENST00000437150.2	grancalcin, EF-hand calcium binding protein	177	1	1	0	0	0	hsa-miR-27a-3p	-0.56	-0.65	N/A
FAM193B	ENST00000514747.1	family with sequence similarity 193, member B	77	1	1	0	0	0	hsa-miR-27a-3p	-0.56	-0.56	N/A
GPAM	ENST00000348367.4	glycerol-3-phosphate acyltransferase, mitochondrial	153	2	0	2	0	2	hsa-miR-27a-3p	-0.56	-0.57	N/A
AL021546.6	ENST00000551806.1	Glutamyl-tRNA(Gln) amidotransferase subunit C, mitochondrial	781	1	1	0	0	0	hsa-miR-27a-3p	-0.56	-0.65	N/A
CDR2	ENST00000268383.2	cerebellar degeneration-related protein 2, 62kDa	70	1	1	0	0	0	hsa-miR-27a-3p	-0.55	-0.55	N/A
CDS1	ENST00000295887.5	CDP-diacylglycerol synthase (phosphatidate cytidylyltransferase) 1	720	2	2	0	0	0	hsa-miR-27a-3p	-0.55	-0.57	N/A
SRP19	ENST00000282999.3	signal recognition particle 19kDa	243	1	1	0	0	0	hsa-miR-27a-3p	-0.55	-0.64	N/A
RCAN2	ENST00000330430.6	regulator of calcineurin 2	32	2	2	0	0	0	hsa-miR-27a-3p	-0.54	-0.59	N/A
FAM133B	ENST00000438306.1	family with sequence similarity 133, member B	32	1	1	0	0	0	hsa-miR-27a-3p	-0.54	-0.54	N/A
FBXO10	ENST00000432825.2	F-box protein 10	14	2	1	0	1	0	hsa-miR-27a-3p	-0.54	-0.54	N/A
PAIP2	ENST00000394795.2	poly(A) binding protein interacting protein 2	2034	1	1	0	0	0	hsa-miR-27a-3p	-0.54	-0.54	N/A
PLEKHH1	ENST00000329153.5	pleckstrin homology domain containing, family H (with MyTH4 domain) member 1	125	1	0	1	0	0	hsa-miR-27a-3p	-0.53	-0.53	N/A
HOXB8	ENST00000239144.4	homeobox B8	9	1	1	0	0	1	hsa-miR-27a-3p	-0.53	-0.53	N/A
SLC39A11	ENST00000255559.3	solute carrier family 39, member 11	544	1	0	1	0	0	hsa-miR-27a-3p	-0.53	-0.55	N/A
PPP4R2	ENST00000356692.5	protein phosphatase 4, regulatory subunit 2	310	1	1	0	0	1	hsa-miR-27a-3p	-0.53	-0.53	N/A
INPP1	ENST00000322522.4	inositol polyphosphate-1-phosphatase	525	1	1	0	0	0	hsa-miR-27a-3p	-0.53	-0.53	N/A
SLITRK1	ENST00000377084.2	SLIT and NTRK-like family, member 1	5	1	1	0	0	0	hsa-miR-27a-3p	-0.52	-0.52	N/A
GRB2	ENST00000392563.1	growth factor receptor-bound protein 2	2660	1	1	0	0	2	hsa-miR-27a-3p	-0.52	-0.55	N/A
SFXN4	ENST00000355697.2	sideroflexin 4	304	1	1	0	0	0	hsa-miR-27a-3p	-0.52	-0.52	N/A
BTG2	ENST00000290551.4	BTG family, member 2	758	2	1	0	1	1	hsa-miR-27a-3p	-0.52	-0.52	N/A
USP46	ENST00000441222.3	ubiquitin specific peptidase 46	180	1	1	0	0	1	hsa-miR-27a-3p	-0.52	-0.63	N/A
GALNT5	ENST00000259056.4	UDP-N-acetyl-alpha-D-galactosamine:polypeptide N-acetylgalactosaminyltransferase 5 (GalNAc-T5)	462	1	1	0	0	0	hsa-miR-27a-3p	-0.51	-0.52	N/A
AL355390.1	ENST00000325811.1	Uncharacterized protein	5	1	1	0	0	1	hsa-miR-27a-3p	-0.51	-0.51	N/A
C10orf137	ENST00000337623.3	chromosome 10 open reading frame 137	157	1	1	0	0	1	hsa-miR-27a-3p	-0.51	-0.51	N/A
C6orf120	ENST00000332290.2	chromosome 6 open reading frame 120	1415	1	0	1	0	1	hsa-miR-27a-3p	-0.5	-0.57	N/A
SPRY2	ENST00000377102.1	sprouty homolog 2 (Drosophila)	139	1	1	0	0	0	hsa-miR-27a-3p	-0.5	-0.5	N/A
LPAR6	ENST00000378434.4	lysophosphatidic acid receptor 6	5	1	1	0	0	0	hsa-miR-27a-3p	-0.5	-0.5	N/A
RUNX1	ENST00000344691.4	runt-related transcription factor 1	182	2	1	0	1	0	hsa-miR-27a-3p	-0.5	-0.5	N/A
PDS5B	ENST00000315596.10	PDS5, regulator of cohesion maintenance, homolog B (S. cerevisiae)	1493	1	1	0	0	0	hsa-miR-27a-3p	-0.5	-0.52	N/A
PRR3	ENST00000376560.3	proline rich 3	1145	3	0	1	2	1	hsa-miR-27a-3p	-0.5	-0.52	N/A
EYA4	ENST00000367895.5	eyes absent homolog 4 (Drosophila)	242	2	1	1	0	0	hsa-miR-27a-3p	-0.5	-0.54	N/A
NEDD4	ENST00000338963.2	neural precursor cell expressed, developmentally down-regulated 4, E3 ubiquitin protein ligase	289	1	1	0	0	2	hsa-miR-27a-3p	-0.5	-0.5	N/A
GRIN2D	ENST00000263269.3	glutamate receptor, ionotropic, N-methyl D-aspartate 2D	5	4	0	4	0	0	hsa-miR-27a-3p	-0.49	-0.49	N/A
SNX12	ENST00000374274.3	sorting nexin 12	849	1	1	0	0	0	hsa-miR-27a-3p	-0.49	-0.49	N/A
FAM184A	ENST00000338891.7	family with sequence similarity 184, member A	167	1	1	0	0	1	hsa-miR-27a-3p	-0.49	-0.49	N/A
RPA3-AS1	ENST00000406829.1	RPA3 antisense RNA 1	22	1	1	0	0	0	hsa-miR-27a-3p	-0.49	-0.49	N/A
HBEGF	ENST00000230990.6	heparin-binding EGF-like growth factor	28	1	1	0	0	1	hsa-miR-27a-3p	-0.49	-0.49	N/A
CREBRF	ENST00000540014.1	CREB3 regulatory factor	24	1	1	0	0	0	hsa-miR-27a-3p	-0.49	-0.49	N/A
SMAD9	ENST00000399275.2	SMAD family member 9	62	2	1	1	0	1	hsa-miR-27a-3p	-0.49	-0.52	N/A
MIER3	ENST00000381226.3	mesoderm induction early response 1, family member 3	112	2	1	1	0	2	hsa-miR-27a-3p	-0.49	-0.54	N/A
VSTM5	ENST00000409977.1	V-set and transmembrane domain containing 5	5	1	1	0	0	0	hsa-miR-27a-3p	-0.48	-0.48	N/A
NR2F2	ENST00000394166.3	nuclear receptor subfamily 2, group F, member 2	4428	1	1	0	0	3	hsa-miR-27a-3p	-0.48	-0.53	N/A
CA10	ENST00000570565.1	carbonic anhydrase X	5	1	1	0	0	1	hsa-miR-27a-3p	-0.48	-0.48	N/A
MAP2K4	ENST00000415385.3	mitogen-activated protein kinase kinase 4	80	1	1	0	0	0	hsa-miR-27a-3p	-0.48	-0.48	N/A
PANK1	ENST00000322191.6	pantothenate kinase 1	435	1	1	0	0	2	hsa-miR-27a-3p	-0.47	-0.47	N/A

UBE2D1	ENST00000373910.4	ubiquitin-conjugating enzyme E2D 1	200	1	0	1	0	0hsa-miR-27a-3p	-0.47	-0.47N/A
THOC1	ENST00000261600.6	THO complex 1	384	1	0	1	0	0hsa-miR-27a-3p	-0.47	-0.47N/A
ZBTB37	ENST00000367701.5	zinc finger and BTB domain containing 37	213	3	3	0	0	3hsa-miR-27a-3p	-0.47	-0.51N/A
AFF4	ENST00000265343.5	AF4/FMR2 family, member 4	2755	2	1	1	0	0hsa-miR-27a-3p	-0.46	-0.48N/A
ABHD17C	ENST00000258884.4	abhydrolase domain containing 17C	406	1	1	0	0	1hsa-miR-27a-3p	-0.46	-0.46N/A
UNKL	ENST00000389221.4	unkempt homolog (Drosophila)-like	308	1	1	0	0	2hsa-miR-27a-3p	-0.46	-0.48N/A
MATN3	ENST00000407540.3	matrilin 3	113	1	1	0	0	0hsa-miR-27a-3p	-0.46	-0.52N/A
RGPD6	ENST00000329516.3	RANBP2-like and GRIP domain containing 6	5	2	2	0	0	1hsa-miR-27a-3p	-0.46	-0.46N/A
PSPC1	ENST00000338910.4	paraspeckle component 1	14	1	1	0	0	0hsa-miR-27a-3p	-0.46	-0.49N/A
CKAP4	ENST00000378026.4	cytoskeleton-associated protein 4	1923	1	1	0	0	0hsa-miR-27a-3p	-0.46	-0.48N/A
POGLUT1	ENST00000295588.4	protein O-glucosyltransferase 1	262	1	1	0	0	0hsa-miR-27a-3p	-0.46	-0.48N/A
ID3	ENST00000374561.5	inhibitor of DNA binding 3, dominant negative helix-loop-helix protein	1295	1	1	0	0	0hsa-miR-27a-3p	-0.46	-0.46N/A
LETMD1	ENST00000380123.2	LETM1 domain containing 1	1619	1	0	1	0	1hsa-miR-27a-3p	-0.46	-0.49N/A
GAB1	ENST00000262995.4	GRB2-associated binding protein 1	26	3	0	3	0	1hsa-miR-27a-3p	-0.46	-0.97N/A
UBE2V1	ENST00000371657.5	ubiquitin-conjugating enzyme E2 variant 1	1621	1	1	0	0	0hsa-miR-27a-3p	-0.46	-0.46N/A
PLCL2	ENST00000418129.2	phospholipase C-like 2	122	1	1	0	0	0hsa-miR-27a-3p	-0.45	-0.49N/A
EPB41L4A	ENST00000261486.5	erythrocyte membrane protein band 4.1 like 4A	103	1	1	0	0	0hsa-miR-27a-3p	-0.45	-0.45N/A
FAM65B	ENST00000259698.4	family with sequence similarity 65, member B	7	1	1	0	0	1hsa-miR-27a-3p	-0.45	-0.45N/A
CLPP	ENST00000596605.1	caseinolytic mitochondrial matrix peptidase proteolytic subunit	4038	1	0	1	0	1hsa-miR-27a-3p	-0.45	-0.45N/A
MFSD2A	ENST00000372811.5	major facilitator superfamily domain containing 2A	113	1	1	0	0	1hsa-miR-27a-3p	-0.45	-0.46N/A
GPATCH2L	ENST00000553588.1	G patch domain containing 2-like	96	1	0	1	0	0hsa-miR-27a-3p	-0.45	-0.52N/A
C20orf194	ENST00000453730.2	chromosome 20 open reading frame 194	1083	1	0	1	0	0hsa-miR-27a-3p	-0.45	-0.45N/A
KCNA4	ENST00000328224.6	potassium voltage-gated channel, shaker-related subfamily, member 4	5	1	1	0	0	0hsa-miR-27a-3p	-0.45	-0.45N/A
GOSR2	ENST00000576910.2	golgi SNAP receptor complex member 2	1099	1	1	0	0	4hsa-miR-27a-3p	-0.45	-0.48N/A
FASTKD5	ENST00000380266.3	FAST kinase domains 5	53	1	1	0	0	0hsa-miR-27a-3p	-0.44	-0.44N/A
YWHAQ	ENST00000381844.4	tyrosine 3-monooxygenase/tryptophan 5-monooxygenase activation protein, theta polypeptide	473	1	1	0	0	0hsa-miR-27a-3p	-0.44	-0.44N/A
UAP1	ENST00000367926.4	UDP-N-acteylglucosamine pyrophosphorylase 1	1738	1	1	0	0	0hsa-miR-27a-3p	-0.44	-0.44N/A
ITSN2	ENST00000361999.3	intersectin 2	63	1	1	0	0	0hsa-miR-27a-3p	-0.44	-0.44N/A
RBPMS2	ENST00000560606.1	RNA binding protein with multiple splicing 2	34	1	1	0	0	2hsa-miR-27a-3p	-0.44	-0.49N/A
ZBTB34	ENST00000319119.4	zinc finger and BTB domain containing 34	726	2	1	1	0	0hsa-miR-27a-3p	-0.44	-0.45N/A
GNS	ENST00000418919.2	glucosamine (N-acetyl)-6-sulfatase	2435	2	1	1	0	1hsa-miR-27a-3p	-0.44	-0.46N/A
STYK1	ENST00000075503.3	serine/threonine/tyrosine kinase 1	13	1	1	0	0	0hsa-miR-27a-3p	-0.44	-0.44N/A
UBE2NL	ENST00000370494.1	ubiquitin-conjugating enzyme E2N-like	5	1	1	0	0	0hsa-miR-27a-3p	-0.43	-0.43N/A
CNN3	ENST00000370206.4	calponin 3, acidic	4490	1	1	0	0	0hsa-miR-27a-3p	-0.43	-0.43N/A
ING5	ENST00000313552.6	inhibitor of growth family, member 5	529	1	1	0	0	0hsa-miR-27a-3p	-0.43	-0.43N/A
B3GNT7	ENST00000287590.5	UDP-GlcNAc:betaGal beta-1,3-N-acetylglucosaminyltransferase 7	5	1	1	0	0	0hsa-miR-27a-3p	-0.43	-0.43N/A
ZNF385D	ENST00000281523.2	zinc finger protein 385D	5	1	1	0	0	1hsa-miR-27a-3p	-0.43	-0.43N/A
ZNF268	ENST00000542986.2	zinc finger protein 268	156	1	1	0	0	0hsa-miR-27a-3p	-0.43	-0.46N/A
PRKX	ENST00000262848.5	protein kinase, X-linked	12	2	1	1	0	1hsa-miR-27a-3p	-0.43	-0.43N/A
GALNT7	ENST00000265000.4	UDP-N-acetyl-alpha-D-galactosamine:polypeptide N-acetylgalactosaminyltransferase 7 (GalNAc-T7)	953	1	0	1	0	1hsa-miR-27a-3p	-0.43	-0.43N/A
NETO1	ENST00000327305.6	neuropilin (NRP) and tolloid (TLL)-like 1	5	1	1	0	0	0hsa-miR-27a-3p	-0.42	-0.43N/A
GABRP	ENST00000518525.1	gamma-aminobutyric acid (GABA) A receptor, pi	5	1	1	0	0	0hsa-miR-27a-3p	-0.42	-0.42N/A
SLC16A10	ENST00000368850.3	solute carrier family 16 (aromatic amino acid transporter), member 10	233	1	1	0	0	1hsa-miR-27a-3p	-0.42	-0.53N/A
PDK4	ENST00000005178.5	pyruvate dehydrogenase kinase, isozyme 4	5549	1	1	0	0	1hsa-miR-27a-3p	-0.42	-0.42N/A
LIMK1	ENST00000418310.1	LIM domain kinase 1	464	1	1	0	0	0hsa-miR-27a-3p	-0.42	-0.42N/A
PPIF	ENST00000225174.3	peptidylprolyl isomerase F	9304	1	1	0	0	2hsa-miR-27a-3p	-0.42	-0.42N/A
ATP5G3	ENST00000284727.4	ATP synthase, H+ transporting, mitochondrial Fo complex, subunit C3 (subunit 9)	7944	1	0	1	0	2hsa-miR-27a-3p	-0.42	-0.52N/A
NCOA7	ENST00000392477.2	nuclear receptor coactivator 7	2967	1	1	0	0	0hsa-miR-27a-3p	-0.42	-0.46N/A
CPEB3	ENST00000412050.4	cytoplasmic polyadenylation element binding protein 3	48	2	0	2	0	2hsa-miR-27a-3p	-0.42	-0.49N/A
PCDH9	ENST00000377861.3	protocadherin 9	102	1	1	0	0	0hsa-miR-27a-3p	-0.42	-0.45N/A
ARF3	ENST00000256682.4	ADP-ribosylation factor 3	1093	3	1	1	1	0hsa-miR-27a-3p	-0.42	-0.56N/A
CDIP1	ENST00000564828.1	cell death-inducing p53 target 1	1857	1	1	0	0	1hsa-miR-27a-3p	-0.42	-0.42N/A
BCL3	ENST00000164227.5	B-cell CLL/lymphoma 3	2099	1	1	0	0	0hsa-miR-27a-3p	-0.41	-0.41N/A
TSLP	ENST00000379706.4	thymic stromal lymphopoietin	62	1	0	1	0	0hsa-miR-27a-3p	-0.41	-0.41N/A

TMCC1	ENST00000432054.2	transmembrane and coiled-coil domain family 1	97	1	1	0	0	0hsa-miR-27a-3p	-0.41	-0.41N/A
SHE	ENST00000304760.2	Src homology 2 domain containing E	9	2	1	1	0	2hsa-miR-27a-3p	-0.41	-0.44N/A
RNF139	ENST00000303545.3	ring finger protein 139	237	2	0	1	1	0hsa-miR-27a-3p	-0.4	-0.4N/A
PDE6D	ENST00000409772.1	phosphodiesterase 6D, cGMP-specific, rod, delta	1265	1	0	1	0	0hsa-miR-27a-3p	-0.4	-0.4N/A
RASAL2	ENST00000448150.3	RAS protein activator like 2	950	1	0	0	1	6hsa-miR-27a-3p	-0.4	-0.64N/A
PDE7B	ENST00000308191.6	phosphodiesterase 7B	148	1	1	0	0	1hsa-miR-27a-3p	-0.4	-0.62N/A
GFPT2	ENST00000253778.8	glutamine-fructose-6-phosphate transaminase 2	70	1	1	0	0	0hsa-miR-27a-3p	-0.4	-0.4N/A
RELN	ENST00000428762.1	reelin	252	1	1	0	0	1hsa-miR-27a-3p	-0.4	-0.4N/A
GXYLT1	ENST00000398675.3	glucoside xylosyltransferase 1	126	2	1	1	0	1hsa-miR-27a-3p	-0.4	-0.62N/A
HOXA10	ENST00000283921.4	homeobox A10	1910	1	1	0	0	0hsa-miR-27a-3p	-0.4	-0.4N/A
TNRC18	ENST00000399537.4	trinucleotide repeat containing 18	5	1	1	0	0	0hsa-miR-27a-3p	-0.4	-0.4N/A
CNOT7	ENST00000361272.4	CCR4-NOT transcription complex, subunit 7	415	1	1	0	0	1hsa-miR-27a-3p	-0.4	-0.4N/A
NMB	ENST00000360476.3	neuromedin B	529	1	0	1	0	0hsa-miR-27a-3p	-0.4	-0.4N/A
B3GAT2	ENST00000230053.6	beta-1,3-glucuronyltransferase 2 (glucuronosyltransferase S)	39	1	0	0	1	2hsa-miR-27a-3p	-0.4	-0.41N/A
RGS17	ENST00000367225.2	regulator of G-protein signaling 17	11	1	1	0	0	2hsa-miR-27a-3p	-0.4	-0.45N/A
RPGRIP1L	ENST00000262135.4	RPGRIP1-like	55	1	1	0	0	1hsa-miR-27a-3p	-0.39	-0.42N/A
NXT2	ENST00000372106.1	nuclear transport factor 2-like export factor 2	390	1	1	0	0	0hsa-miR-27a-3p	-0.39	-0.39N/A
YWHAB	ENST00000353703.4	tyrosine 3-monooxygenase/tryptophan 5-monooxygenase activation protein, beta polypeptide	272	1	0	1	0	1hsa-miR-27a-3p	-0.39	-0.59N/A
PSMA1	ENST00000396394.2	proteasome (prosome, macropain) subunit, alpha type, 1	124	1	1	0	0	0hsa-miR-27a-3p	-0.39	-0.46N/A
COLQ	ENST00000383785.2	collagen-like tail subunit (single strand of homotrimer) of asymmetric acetylcholinesterase	8	2	1	1	0	2hsa-miR-27a-3p	-0.39	-0.39N/A
AK4	ENST00000545314.1	adenylate kinase 4	116	1	0	1	0	0hsa-miR-27a-3p	-0.39	-0.39N/A
NUDT13	ENST00000372997.3	nudix (nucleoside diphosphate linked moiety X)-type motif 13	10	1	1	0	0	0hsa-miR-27a-3p	-0.39	-0.39N/A
DLL4	ENST00000249749.5	delta-like 4 (Drosophila)	605	1	1	0	0	0hsa-miR-27a-3p	-0.38	-0.38N/A
NPEPPS	ENST00000322157.4	aminopeptidase puromycin sensitive	2194	1	1	0	0	0hsa-miR-27a-3p	-0.38	-0.39N/A
ZNF800	ENST00000393313.1	zinc finger protein 800	239	1	1	0	0	1hsa-miR-27a-3p	-0.38	-0.39N/A
IER3	ENST00000376377.2	immediate early response 3	768	1	0	0	1	0hsa-miR-27a-3p	-0.38	-0.4N/A
PARD6B	ENST00000371610.2	par-6 family cell polarity regulator beta	277	1	1	0	0	2hsa-miR-27a-3p	-0.38	-0.39N/A
PDE8B	ENST00000264917.5	phosphodiesterase 8B	186	1	1	0	0	4hsa-miR-27a-3p	-0.38	-0.42N/A
KCNK2	ENST00000444842.2	potassium channel, subfamily K, member 2	258	2	1	1	0	1hsa-miR-27a-3p	-0.38	-0.46N/A
APPBP2	ENST00000083182.3	amyloid beta precursor protein (cytoplasmic tail) binding protein 2	907	1	1	0	0	0hsa-miR-27a-3p	-0.38	-0.38N/A
NRK	ENST00000243300.9	Nik related kinase	5	2	1	1	0	0hsa-miR-27a-3p	-0.38	-0.38N/A
LPIN2	ENST00000261596.4	lipin 2	376	1	0	0	1	5hsa-miR-27a-3p	-0.38	-0.38N/A
SLIT2	ENST00000504154.1	slit homolog 2 (Drosophila)	579	1	0	1	0	1hsa-miR-27a-3p	-0.38	-0.48N/A
SLC25A25	ENST00000373069.5	solute carrier family 25 (mitochondrial carrier; phosphate carrier), member 25	3454	2	0	0	2	0hsa-miR-27a-3p	-0.37	-0.38N/A
ARHGEF26	ENST00000356448.4	Rho guanine nucleotide exchange factor (GEF) 26	164	1	0	0	1	0hsa-miR-27a-3p	-0.37	-0.38N/A
CDH11	ENST00000394156.3	cadherin 11, type 2, OB-cadherin (osteoblast)	448	1	1	0	0	0hsa-miR-27a-3p	-0.37	-0.37N/A
AK2	ENST00000467905.1	adenylate kinase 2	3125	1	0	1	0	1hsa-miR-27a-3p	-0.37	-0.37N/A
EDEM3	ENST00000318130.8	ER degradation enhancer, mannosidase alpha-like 3	535	2	0	1	1	1hsa-miR-27a-3p	-0.37	-0.38N/A
BRSK1	ENST00000590333.1	BR serine/threonine kinase 1	117	1	1	0	0	0hsa-miR-27a-3p	-0.37	-0.37N/A
RNF141	ENST00000265981.2	ring finger protein 141	345	1	1	0	0	0hsa-miR-27a-3p	-0.37	-0.4N/A
NLN	ENST00000380985.5	neurolysin (metallopeptidase M3 family)	885	1	0	1	0	0hsa-miR-27a-3p	-0.37	-0.37N/A
SLC35F1	ENST00000360388.4	solute carrier family 35, member F1	56	1	1	0	0	2hsa-miR-27a-3p	-0.37	-0.37N/A
RSPO3	ENST00000356698.4	R-spondin 3	118	1	1	0	0	0hsa-miR-27a-3p	-0.37	-0.39N/A
RGPD5	ENST00000016946.3	RANBP2-like and GRIP domain containing 5	5	1	1	0	0	1hsa-miR-27a-3p	-0.37	-0.37N/A
MMP16	ENST00000286614.6	matrix metallopeptidase 16 (membrane-inserted)	12	3	1	0	2	1hsa-miR-27a-3p	-0.37	-0.49N/A
FOXN4	ENST00000355216.1	forkhead box N4	16	1	1	0	0	0hsa-miR-27a-3p	-0.37	-0.37N/A
HORMAD2	ENST00000336726.6	HORMA domain containing 2	5	1	1	0	0	0hsa-miR-27a-3p	-0.37	-0.37N/A
RSBN1L	ENST00000334955.8	round spermatid basic protein 1-like	640	1	1	0	0	0hsa-miR-27a-3p	-0.37	-0.4N/A
E2F7	ENST00000322886.7	E2F transcription factor 7	308	1	1	0	0	0hsa-miR-27a-3p	-0.37	-0.37N/A
PTGER3	ENST00000370924.4	prostaglandin E receptor 3 (subtype EP3)	14	1	1	0	0	3hsa-miR-27a-3p	-0.37	-0.37N/A
LIN28B	ENST00000345080.4	lin-28 homolog B (C. elegans)	118	1	1	0	0	1hsa-miR-27a-3p	-0.37	-0.37N/A
CACYBP	ENST00000367681.2	calcyclin binding protein	374	1	0	1	0	1hsa-miR-27a-3p	-0.37	-0.38N/A
GSPT1	ENST00000434724.2	G1 to S phase transition 1	1119	1	0	1	0	0hsa-miR-27a-3p	-0.37	-0.54N/A
FRS3	ENST00000373018.3	fibroblast growth factor receptor substrate 3	41	1	1	0	0	1hsa-miR-27a-3p	-0.37	-0.37N/A
PPP1CC	ENST00000335007.5	protein phosphatase 1, catalytic subunit, gamma isozyme	3739	1	1	0	0	0hsa-miR-27a-3p	-0.37	-0.37N/A

ANK3	ENST00000280772.2	ankyrin 3, node of Ranvier (ankyrin G)	74	1	0	0	1	1	hsa-miR-27a-3p	-0.36	-0.5	N/A
NRXN1	ENST00000342183.5	neurexin 1	5	1	0	1	0	1	hsa-miR-27a-3p	-0.36	-0.36	N/A
RCBTB1	ENST00000258646.3	regulator of chromosome condensation (RCC1) and BTB (POZ) domain containing protein 1	9	1	1	0	0	0	hsa-miR-27a-3p	-0.36	-0.36	N/A
TMEM167A	ENST00000502346.1	transmembrane protein 167A	1029	1	0	1	0	1	hsa-miR-27a-3p	-0.36	-0.42	N/A
GLTP	ENST00000318348.4	glycolipid transfer protein	5	1	1	0	0	0	hsa-miR-27a-3p	-0.36	-0.36	N/A
IFITM10	ENST00000340134.4	interferon induced transmembrane protein 10	7	1	0	0	1	0	hsa-miR-27a-3p	-0.36	-0.41	N/A
YPEL3	ENST00000398841.1	yippee-like 3 (Drosophila)	330	1	0	1	0	0	hsa-miR-27a-3p	-0.36	-0.36	N/A
WNK3	ENST00000375169.3	WNK lysine deficient protein kinase 3	55	2	0	1	1	1	hsa-miR-27a-3p	-0.36	-0.36	N/A
BRWD3	ENST00000373275.4	bromodomain and WD repeat domain containing 3	506	1	1	0	0	5	hsa-miR-27a-3p	-0.36	-0.5	N/A
MARCKS	ENST00000368635.4	myristoylated alanine-rich protein kinase C substrate	188	1	0	1	0	2	hsa-miR-27a-3p	-0.36	-0.37	N/A
CREB1	ENST00000432329.2	cAMP responsive element binding protein 1	295	1	1	0	0	1	hsa-miR-27a-3p	-0.36	-0.38	N/A
JHDM1D	ENST00000397560.2	jumonji C domain containing histone demethylase 1 homolog D (S. cerevisiae)	470	2	1	0	1	1	hsa-miR-27a-3p	-0.36	-0.4	N/A
TLK2	ENST00000582809.1	tousled-like kinase 2	5	1	1	0	0	1	hsa-miR-27a-3p	-0.35	-0.35	N/A
ADAMTS10	ENST00000595838.1	ADAM metallopeptidase with thrombospondin type 1 motif, 10	7	1	1	0	0	0	hsa-miR-27a-3p	-0.35	-0.35	N/A
ZHX1	ENST00000395571.3	zinc fingers and homeoboxes 1	88	1	1	0	0	0	hsa-miR-27a-3p	-0.35	-0.35	N/A
UBE2N	ENST00000550657.1	ubiquitin-conjugating enzyme E2N	321	1	1	0	0	0	hsa-miR-27a-3p	-0.35	-0.35	N/A
KIAA1211L	ENST00000397899.2	KIAA1211-like	7	1	1	0	0	0	hsa-miR-27a-3p	-0.35	-0.35	N/A
ASB11	ENST00000537676.1	ankyrin repeat and SOCS box containing 11	5	1	1	0	0	0	hsa-miR-27a-3p	-0.35	-0.35	N/A
OTX2	ENST00000339475.5	orthodenticle homeobox 2	5	1	0	1	0	1	hsa-miR-27a-3p	-0.35	-0.35	N/A
TTC39A	ENST00000530004.1	tetratricopeptide repeat domain 39A	174	1	0	1	0	0	hsa-miR-27a-3p	-0.35	-0.38	N/A
WBP1L	ENST00000369889.4	WW domain binding protein 1-like	5	2	0	1	1	3	hsa-miR-27a-3p	-0.35	-0.35	N/A
NPAS3	ENST00000346562.2	neuronal PAS domain protein 3	9	1	0	0	1	1	hsa-miR-27a-3p	-0.35	-0.35	N/A
GLRA2	ENST00000218075.4	glycine receptor, alpha 2	5	1	0	1	0	0	hsa-miR-27a-3p	-0.35	-0.35	N/A
METTL21A	ENST00000448823.2	methyltransferase like 21A	224	1	0	1	0	2	hsa-miR-27a-3p	-0.35	-0.35	N/A
UGCG	ENST00000374279.3	UDP-glucose ceramide glucosyltransferase	1200	1	0	0	1	2	hsa-miR-27a-3p	-0.35	-0.35	N/A
CCNJ	ENST00000265992.5	cyclin J	5	1	1	0	0	1	hsa-miR-27a-3p	-0.35	-0.35	N/A
FOSB	ENST00000586615.1	FBJ murine osteosarcoma viral oncogene homolog B	11	1	1	0	0	2	hsa-miR-27a-3p	-0.35	-0.35	N/A
YPEL2	ENST00000312655.4	yippee-like 2 (Drosophila)	62	1	1	0	0	1	hsa-miR-27a-3p	-0.35	-0.35	N/A
KCNJ6	ENST00000609713.1	potassium inwardly-rectifying channel, subfamily J, member 6	5	2	1	1	0	6	hsa-miR-27a-3p	-0.35	-0.35	N/A
POU2F3	ENST00000260264.4	POU class 2 homeobox 3	5	1	0	1	0	0	hsa-miR-27a-3p	-0.35	-0.35	N/A
ENDOU	ENST00000229003.3	endonuclease, polyU-specific	9	1	1	0	0	0	hsa-miR-27a-3p	-0.35	-0.35	N/A
ISL1	ENST00000230658.7	ISL LIM homeobox 1	67	1	1	0	0	0	hsa-miR-27a-3p	-0.34	-0.34	N/A
TMEM189-UBE2V1	ENST00000341698.2	TMEM189-UBE2V1 readthrough	1621	1	1	0	0	0	hsa-miR-27a-3p	-0.34	-0.34	N/A
CTD-2140B24.4	ENST00000540096.2	Zinc finger protein 268	156	1	1	0	0	0	hsa-miR-27a-3p	-0.34	-0.38	N/A
TMEM189	ENST00000557021.1	transmembrane protein 189	1621	1	1	0	0	0	hsa-miR-27a-3p	-0.34	-0.34	N/A
CABP1	ENST00000453000.1	calcium binding protein 1	13	1	1	0	0	0	hsa-miR-27a-3p	-0.34	-0.34	N/A
FAM217B	ENST00000358293.3	family with sequence similarity 217, member B	175	1	1	0	0	5	hsa-miR-27a-3p	-0.34	-0.36	N/A
CSDC2	ENST00000306149.7	cold shock domain containing C2, RNA binding	31	1	0	1	0	1	hsa-miR-27a-3p	-0.34	-0.34	N/A
GDF6	ENST00000287020.5	growth differentiation factor 6	14	1	1	0	0	1	hsa-miR-27a-3p	-0.34	-0.35	N/A
PAQR9	ENST00000340634.3	progestin and adipoQ receptor family member IX	233	1	1	0	0	1	hsa-miR-27a-3p	-0.34	-0.36	N/A
TROVE2	ENST00000432079.1	TROVE domain family, member 2	380	1	0	1	0	0	hsa-miR-27a-3p	-0.34	-0.34	N/A
APEX1	ENST00000557054.1	APEX nuclease (multifunctional DNA repair enzyme) 1	15798	1	0	1	0	1	hsa-miR-27a-3p	-0.34	-0.34	N/A
SLC16A13	ENST00000308027.6	solute carrier family 16, member 13	137	1	1	0	0	1	hsa-miR-27a-3p	-0.34	-0.44	N/A
SYNRG	ENST00000339208.6	synergin, gamma	106	2	1	0	1	2	hsa-miR-27a-3p	-0.34	-0.34	N/A
CCM2	ENST00000541586.1	cerebral cavernous malformation 2	1046	1	1	0	0	0	hsa-miR-27a-3p	-0.34	-0.34	N/A
ST3GAL6	ENST00000265261.6	ST3 beta-galactoside alpha-2,3-sialyltransferase 6	150	1	0	0	1	0	hsa-miR-27a-3p	-0.33	-0.33	N/A
NEUROD6	ENST00000297142.3	neuronal differentiation 6	5	1	0	1	0	0	hsa-miR-27a-3p	-0.33	-0.33	N/A
TRPV3	ENST00000301365.4	transient receptor potential cation channel, subfamily V, member 3	13	3	2	1	0	2	hsa-miR-27a-3p	-0.33	-0.58	N/A
GORASP1	ENST00000319283.3	golgi reassembly stacking protein 1, 65kDa	102	1	1	0	0	0	hsa-miR-27a-3p	-0.33	-0.5	N/A
PELI2	ENST00000267460.4	pellino E3 ubiquitin protein ligase family member 2	229	1	1	0	0	0	hsa-miR-27a-3p	-0.33	-0.33	N/A
RP11-122A3.2	ENST00000517562.2	uncharacterized protein LOC100127983	579	1	0	1	0	0	hsa-miR-27a-3p	-0.33	-0.33	N/A
SEMA6A	ENST00000343348.6	sema domain, transmembrane domain (TM), and cytoplasmic domain, (semaphorin) 6A	106	2	1	0	1	1	hsa-miR-27a-3p	-0.33	-0.43	N/A
ASAH1	ENST00000262097.6	N-acylsphingosine amidohydrolase (acid ceramidase) 1	445	1	1	0	0	0	hsa-miR-27a-3p	-0.33	-0.4	N/A

BMPR2	ENST00000374574.2	bone morphogenetic protein receptor, type II (serine/threonine kinase)	287	1	0	0	1	1hsa-miR-27a-3p	-0.33	-0.45N/A
TRAPPC8	ENST00000283351.4	trafficking protein particle complex 8	502	1	1	0	0	0hsa-miR-27a-3p	-0.33	-0.33N/A
DKK2	ENST00000285311.3	dickkopf WNT signaling pathway inhibitor 2	5	1	1	0	0	0hsa-miR-27a-3p	-0.33	-0.33N/A
SRSF9	ENST00000229390.3	serine/arginine-rich splicing factor 9	946	1	0	1	0	1hsa-miR-27a-3p	-0.33	-0.33N/A
PIP5K1B	ENST00000265382.3	phosphatidylinositol-4-phosphate 5-kinase, type I, beta	69	1	1	0	0	0hsa-miR-27a-3p	-0.33	-0.33N/A
PDPK1	ENST00000441549.3	3-phosphoinositide dependent protein kinase-1	104	2	0	2	0	4hsa-miR-27a-3p	-0.32	-0.34N/A
ORC5	ENST00000297431.4	origin recognition complex, subunit 5	407	1	0	1	0	0hsa-miR-27a-3p	-0.32	-0.32N/A
UBR1	ENST00000382177.2	ubiquitin protein ligase E3 component n-recognin 1	11	2	0	2	0	2hsa-miR-27a-3p	-0.32	-0.38N/A
OAF	ENST00000328965.4	OAF homolog (Drosophila)	1447	1	1	0	0	0hsa-miR-27a-3p	-0.32	-0.32N/A
DNAJC13	ENST00000260818.6	DnaJ (Hsp40) homolog, subfamily C, member 13	256	1	1	0	0	0hsa-miR-27a-3p	-0.32	-0.32N/A
NRBF2	ENST00000277746.6	nuclear receptor binding factor 2	11	1	1	0	0	0hsa-miR-27a-3p	-0.32	-0.32N/A
PITHD1	ENST00000246151.4	PITH (C-terminal proteasome-interacting domain of thioredoxin-like) domain containing 1	718	1	0	1	0	0hsa-miR-27a-3p	-0.32	-0.32N/A
PKIB	ENST00000258014.3	protein kinase (cAMP-dependent, catalytic) inhibitor beta	545	1	0	1	0	0hsa-miR-27a-3p	-0.32	-0.32N/A
SUCO	ENST00000367723.4	SUN domain containing ossification factor	86	1	1	0	0	0hsa-miR-27a-3p	-0.32	-0.38N/A
CSRNP1	ENST00000273153.5	cysteine-serine-rich nuclear protein 1	544	1	1	0	0	2hsa-miR-27a-3p	-0.32	-0.32N/A
SLC10A7	ENST00000264986.3	solute carrier family 10, member 7	77	1	0	1	0	2hsa-miR-27a-3p	-0.32	-0.35N/A
ERG	ENST00000398905.1	v-ets avian erythroblastosis virus E26 oncogene homolog	184	1	1	0	0	0hsa-miR-27a-3p	-0.32	-0.32N/A
EYA1	ENST00000388742.4	eyes absent homolog 1 (Drosophila)	222	2	1	0	1	1hsa-miR-27a-3p	-0.32	-0.32N/A
DROSHA	ENST00000511367.2	drosha, ribonuclease type III	3272	1	0	1	0	0hsa-miR-27a-3p	-0.32	-0.32N/A
C19orf81	ENST00000425202.1	chromosome 19 open reading frame 81	63	1	0	1	0	0hsa-miR-27a-3p	-0.32	-0.32N/A
LIN54	ENST00000395282.2	lin-54 homolog (C. elegans)	228	1	1	0	0	0hsa-miR-27a-3p	-0.32	-0.32N/A
GMPS	ENST00000496455.2	guanine monphosphate synthase	867	1	1	0	0	2hsa-miR-27a-3p	-0.32	-0.34N/A
ATP6V1A	ENST00000273398.3	ATPase, H+ transporting, lysosomal 70kDa, V1 subunit A	180	1	0	1	0	0hsa-miR-27a-3p	-0.32	-0.34N/A
TAB3	ENST00000378933.1	TGF-beta activated kinase 1/MAP3K7 binding protein 3	201	2	2	0	0	0hsa-miR-27a-3p	-0.32	-0.45N/A
PRPF19	ENST00000227524.4	pre-mRNA processing factor 19	1583	1	1	0	0	1hsa-miR-27a-3p	-0.32	-0.32N/A
COL21A1	ENST00000244728.5	collagen, type XXI, alpha 1	9	1	1	0	0	0hsa-miR-27a-3p	-0.32	-0.32N/A
NIPAL4	ENST00000311946.7	NIPA-like domain containing 4	17	1	1	0	0	0hsa-miR-27a-3p	-0.32	-0.32N/A
EFNB2	ENST00000245323.4	ephrin-B2	25	1	1	0	0	1hsa-miR-27a-3p	-0.32	-0.32N/A
PEG10	ENST00000482108.1	paternally expressed 10	8778	2	1	0	1	1hsa-miR-27a-3p	-0.31	-0.31N/A
CCDC28B	ENST00000421922.2	coiled-coil domain containing 28B	106	1	0	1	0	0hsa-miR-27a-3p	-0.31	-0.31N/A
NEURL4	ENST00000315614.7	neuralized homolog 4 (Drosophila)	469	1	1	0	0	0hsa-miR-27a-3p	-0.31	-0.31N/A
MBTD1	ENST00000586178.1	mbt domain containing 1	113	1	0	1	0	0hsa-miR-27a-3p	-0.31	-0.31N/A
SMIM14	ENST00000295958.5	small integral membrane protein 14	601	1	0	1	0	1hsa-miR-27a-3p	-0.31	-0.31N/A
NECAP2	ENST00000337132.5	NECAP endocytosis associated 2	1011	1	1	0	0	0hsa-miR-27a-3p	-0.31	-0.32N/A
RASSF3	ENST00000542104.1	Ras association (RalGDS/AF-6) domain family member 3	461	2	0	0	2	0hsa-miR-27a-3p	-0.31	-0.32N/A
NR5A2	ENST00000367362.3	nuclear receptor subfamily 5, group A, member 2	121	1	1	0	0	1hsa-miR-27a-3p	-0.31	-0.31N/A
C12orf73	ENST00000547975.1	chromosome 12 open reading frame 73	1060	1	0	0	1	0hsa-miR-27a-3p	-0.31	-0.31N/A
C2CD2	ENST00000329623.7	C2 calcium-dependent domain containing 2	106	2	2	0	0	1hsa-miR-27a-3p	-0.31	-0.31N/A
BMI1	ENST00000376663.3	BMI1 polycomb ring finger oncogene	200	1	1	0	0	0hsa-miR-27a-3p	-0.31	-0.32N/A
CCNK	ENST00000389879.5	cyclin K	1777	1	1	0	0	1hsa-miR-27a-3p	-0.3	-0.54N/A
KCNJ1	ENST00000392665.2	potassium inwardly-rectifying channel, subfamily J, member 1	9	1	1	0	0	1hsa-miR-27a-3p	-0.3	-0.34N/A
BEND7	ENST00000396898.2	BEN domain containing 7	68	1	0	1	0	0hsa-miR-27a-3p	-0.3	-0.3N/A
GNG12	ENST00000370982.3	guanine nucleotide binding protein (G protein), gamma 12	1362	1	0	1	0	0hsa-miR-27a-3p	-0.3	-0.32N/A
SOX11	ENST00000322002.3	SRY (sex determining region Y)-box 11	1100	1	1	0	0	3hsa-miR-27a-3p	-0.3	-0.37N/A
PURA	ENST00000331327.3	purine-rich element binding protein A	367	2	0	1	1	1hsa-miR-27a-3p	-0.3	-0.43N/A
RAP1B	ENST00000250559.9	RAP1B, member of RAS oncogene family	3599	1	1	0	0	0hsa-miR-27a-3p	-0.3	-0.3N/A
BEND4	ENST00000504360.1	BEN domain containing 4	187	2	0	2	0	1hsa-miR-27a-3p	-0.3	-0.3N/A
VANGL2	ENST00000368061.2	VANGL planar cell polarity protein 2	560	2	0	1	1	1hsa-miR-27a-3p	-0.3	-0.3N/A
SATB2	ENST00000417098.1	SATB homeobox 2	820	1	1	0	0	0hsa-miR-27a-3p	-0.3	-0.3N/A
VAT1L	ENST00000302536.2	vesicle amine transport 1-like	123	1	1	0	0	0hsa-miR-27a-3p	-0.3	-0.3N/A
KBTBD8	ENST00000295568.4	kelch repeat and BTB (POZ) domain containing 8	22	1	1	0	0	1hsa-miR-27a-3p	-0.3	-0.3N/A
CCNY	ENST00000374706.1	cyclin Y	672	1	1	0	0	1hsa-miR-27a-3p	-0.3	-0.33N/A
LYSMD3	ENST00000509384.1	LysM, putative peptidoglycan-binding, domain containing 3	92	1	0	1	0	0hsa-miR-27a-3p	-0.3	-0.32N/A
MEPCE	ENST00000310512.2	methylphosphate capping enzyme	1024	1	1	0	0	0hsa-miR-27a-3p	-0.3	-0.31N/A
CYP39A1	ENST00000275016.2	cytochrome P450, family 39, subfamily A, polypeptide 1	152	1	1	0	0	1hsa-miR-27a-3p	-0.3	-0.32N/A
TFPI	ENST00000392365.1	tissue factor pathway inhibitor (lipoprotein-associated coagulation inhibitor)	10747	1	1	0	0	0hsa-miR-27a-3p	-0.3	-0.34N/A

TSC1	ENST00000298552.3	tuberous sclerosis 1	838	1	1	0	0	3hsa-miR-27a-3p	-0.3	-0.32N/A
HSPD1	ENST00000388968.3	heat shock 60kDa protein 1 (chaperonin)	42	1	0	1	0	0hsa-miR-27a-3p	-0.29	-0.29N/A
VGLL2	ENST00000352536.3	vestigial like 2 (Drosophila)	5	1	1	0	0	1hsa-miR-27a-3p	-0.29	-0.32N/A
WSB1	ENST00000262394.2	WD repeat and SOCS box containing 1	175	1	1	0	0	1hsa-miR-27a-3p	-0.29	-0.48N/A
VAV2	ENST00000406606.3	vav 2 guanine nucleotide exchange factor	477	2	0	0	2	1hsa-miR-27a-3p	-0.29	-0.39N/A
CBFB	ENST00000290858.6	core-binding factor, beta subunit	3904	1	0	1	0	0hsa-miR-27a-3p	-0.29	-0.3N/A
EIF5	ENST00000216554.3	eukaryotic translation initiation factor 5	970	1	0	1	0	1hsa-miR-27a-3p	-0.29	-0.46N/A
MDM4	ENST00000391947.2	Mdm4 p53 binding protein homolog (mouse)	443	1	1	0	0	1hsa-miR-27a-3p	-0.29	-0.32N/A
KRAS	ENST00000256078.4	Kirsten rat sarcoma viral oncogene homolog	40	2	0	0	2	0hsa-miR-27a-3p	-0.29	-0.47N/A
GATA3	ENST00000379328.3	GATA binding protein 3	136	1	0	0	1	0hsa-miR-27a-3p	-0.29	-0.29N/A
ZBTB18	ENST00000358704.4	zinc finger and BTB domain containing 18	935	1	0	0	1	1hsa-miR-27a-3p	-0.29	-0.3N/A
ATXN10	ENST00000252934.5	ataxin 10	4734	1	0	1	0	0hsa-miR-27a-3p	-0.29	-0.57N/A
AHSG	ENST00000411641.2	alpha-2-HS-glycoprotein	144558	1	0	1	0	0hsa-miR-27a-3p	-0.29	-0.29N/A
TEAD1	ENST00000361905.4	TEA domain family member 1 (SV40 transcriptional enhancer factor)	740	3	0	2	1	0hsa-miR-27a-3p	-0.29	-0.3N/A
LBH	ENST00000395323.3	limb bud and heart development	1956	1	0	1	0	0hsa-miR-27a-3p	-0.29	-0.3N/A
PDHX	ENST00000448838.3	pyruvate dehydrogenase complex, component X	40	1	1	0	0	0hsa-miR-27a-3p	-0.29	-0.38N/A
MAP3K4	ENST00000366919.2	mitogen-activated protein kinase kinase kinase 4	241	1	1	0	0	0hsa-miR-27a-3p	-0.29	-0.29N/A
WEE1	ENST00000299613.6	WEE1 homolog (S. pombe)	331	1	0	1	0	1hsa-miR-27a-3p	-0.29	-0.32N/A
HOXA13	ENST00000222753.4	homeobox A13	328	1	1	0	0	0hsa-miR-27a-3p	-0.29	-0.31N/A
IRF4	ENST00000380956.4	interferon regulatory factor 4	10	1	1	0	0	1hsa-miR-27a-3p	-0.28	-0.28N/A
PALM2	ENST00000448454.2	paralemmin 2	35	1	1	0	0	2hsa-miR-27a-3p	-0.28	-0.29N/A
SOWAHA	ENST00000378693.2	sosondowah ankyrin repeat domain family member A	138	1	1	0	0	0hsa-miR-27a-3p	-0.28	-0.28N/A
ZMYM4	ENST00000314607.6	zinc finger, MYM-type 4	280	1	1	0	0	1hsa-miR-27a-3p	-0.28	-0.28N/A
H3F3C	ENST00000340398.3	H3 histone, family 3C	7	1	0	1	0	0hsa-miR-27a-3p	-0.28	-0.28N/A
MTMR4	ENST00000579925.1	myotubularin related protein 4	150	1	1	0	0	0hsa-miR-27a-3p	-0.28	-0.28N/A
EFNA2	ENST00000215368.2	ephrin-A2	83	1	0	1	0	1hsa-miR-27a-3p	-0.28	-0.28N/A
PDZK1IP1	ENST00000294338.2	PDZK1 interacting protein 1	37	1	0	0	1	0hsa-miR-27a-3p	-0.28	-0.28N/A
TMEM233	ENST00000426426.1	transmembrane protein 233	5	1	0	1	0	1hsa-miR-27a-3p	-0.28	-0.28N/A
RAB20	ENST00000267328.3	RAB20, member RAS oncogene family	119	1	0	1	0	1hsa-miR-27a-3p	-0.28	-0.28N/A
KDM3A	ENST00000409556.1	lysine (K)-specific demethylase 3A	2300	1	0	1	0	0hsa-miR-27a-3p	-0.28	-0.33N/A
LAPTM4B	ENST00000445593.2	lysosomal protein transmembrane 4 beta	11776	1	0	1	0	0hsa-miR-27a-3p	-0.28	-0.28N/A
USP51	ENST00000500968.3	ubiquitin specific peptidase 51	26	1	0	1	0	0hsa-miR-27a-3p	-0.28	-0.28N/A
PNISR	ENST00000369239.5	PNN-interacting serine/arginine-rich protein	600	2	1	1	0	0hsa-miR-27a-3p	-0.28	-0.5N/A
CEP135	ENST00000257287.4	centrosomal protein 135kDa	19	1	1	0	0	0hsa-miR-27a-3p	-0.28	-0.41N/A
ARRDC4	ENST00000268042.6	arrestin domain containing 4	10	1	0	1	0	1hsa-miR-27a-3p	-0.28	-0.28N/A
UBXN2A	ENST00000309033.4	UBX domain protein 2A	45	2	1	0	1	1hsa-miR-27a-3p	-0.28	-0.39N/A
C17orf85	ENST00000158149.3	chromosome 17 open reading frame 85	718	1	0	1	0	1hsa-miR-27a-3p	-0.28	-0.45N/A
C6orf211	ENST00000367294.3	chromosome 6 open reading frame 211	65	1	0	1	0	0hsa-miR-27a-3p	-0.28	-0.28N/A
COMMD3-BMI1	ENST00000602390.1	COMMD3-BMI1 readthrough	195	1	1	0	0	0hsa-miR-27a-3p	-0.27	-0.29N/A
RALGAPA2	ENST00000202677.7	Ral GTPase activating protein, alpha subunit 2 (catalytic)	24	2	0	2	0	0hsa-miR-27a-3p	-0.27	-0.35N/A
H2AFZ	ENST00000296417.5	H2A histone family, member Z	927	1	0	0	1	0hsa-miR-27a-3p	-0.27	-0.27N/A
THAP1	ENST00000345117.2	THAP domain containing, apoptosis associated protein 1	196	1	0	1	0	1hsa-miR-27a-3p	-0.27	-0.52N/A
CDH5	ENST00000341529.3	cadherin 5, type 2 (vascular endothelium)	8	1	1	0	0	0hsa-miR-27a-3p	-0.27	-0.27N/A
CASC10	ENST00000377113.5	cancer susceptibility candidate 10	116	1	1	0	0	0hsa-miR-27a-3p	-0.27	-0.31N/A
DNAJC27	ENST00000264711.2	DnaJ (Hsp40) homolog, subfamily C, member 27	41	1	1	0	0	0hsa-miR-27a-3p	-0.27	-0.33N/A
SERBP1	ENST00000370994.4	SERPINE1 mRNA binding protein 1	926	1	1	0	0	1hsa-miR-27a-3p	-0.27	-0.28N/A
CHIC1	ENST00000373504.6	cysteine-rich hydrophobic domain 1	16	1	0	1	0	2hsa-miR-27a-3p	-0.27	-0.3N/A
TSC22D2	ENST00000361875.3	TSC22 domain family, member 2	1062	2	0	0	2	1hsa-miR-27a-3p	-0.27	-0.3N/A
EML1	ENST00000262233.6	echinoderm microtubule associated protein like 1	371	1	0	1	0	1hsa-miR-27a-3p	-0.27	-0.27N/A
ZNF597	ENST00000301744.4	zinc finger protein 597	159	1	1	0	0	1hsa-miR-27a-3p	-0.27	-0.32N/A
UBE2F	ENST00000272930.4	ubiquitin-conjugating enzyme E2F (putative)	905	1	0	1	0	2hsa-miR-27a-3p	-0.27	-0.39N/A
GALNT3	ENST00000392701.3	UDP-N-acetyl-alpha-D-galactosamine:polypeptide N-acetylgalactosaminyltransferase 3 (GalNAc-T3)	40	1	0	1	0	0hsa-miR-27a-3p	-0.27	-0.27N/A
RSF1	ENST00000308488.6	remodeling and spacing factor 1	216	1	0	0	1	1hsa-miR-27a-3p	-0.27	-0.27N/A
SRL	ENST00000399609.3	sarcalumenin	5	1	1	0	0	3hsa-miR-27a-3p	-0.27	-0.27N/A
HAPLN1	ENST00000274341.4	hyaluronan and proteoglycan link protein 1	5	1	0	1	0	0hsa-miR-27a-3p	-0.27	-0.27N/A

VEGFB	ENST00000309422.2	vascular endothelial growth factor B	4432	1	0	1	0	0hsa-miR-27a-3p	-0.27	-0.27N/A
MED14	ENST00000324817.1	mediator complex subunit 14	1038	1	0	1	0	1hsa-miR-27a-3p	-0.27	-0.37N/A
RNGTT	ENST00000369485.4	RNA guanylyltransferase and 5'-phosphatase	1384	1	0	1	0	0hsa-miR-27a-3p	-0.27	-0.27N/A
ALDH4A1	ENST00000375341.3	aldehyde dehydrogenase 4 family, member A1	520	1	0	1	0	1hsa-miR-27a-3p	-0.27	-0.27N/A
MYH10	ENST00000360416.3	myosin, heavy chain 10, non-muscle	134	1	1	0	0	0hsa-miR-27a-3p	-0.27	-0.28N/A
ELL2	ENST00000237853.4	elongation factor, RNA polymerase II, 2	397	1	0	1	0	1hsa-miR-27a-3p	-0.27	-0.27N/A
NFE2L2	ENST00000397063.4	nuclear factor, erythroid 2-like 2	1964	1	0	1	0	0hsa-miR-27a-3p	-0.27	-0.27N/A
EBI3	ENST00000221847.5	Epstein-Barr virus induced 3	5	1	0	1	0	0hsa-miR-27a-3p	-0.27	-0.27N/A
ABHD6	ENST00000295962.4	abhydrolase domain containing 6	289	2	0	0	2	0hsa-miR-27a-3p	-0.26	-0.35N/A
ACVR1	ENST00000263640.3	activin A receptor, type I	1523	1	1	0	0	1hsa-miR-27a-3p	-0.26	-0.41N/A
NKAIN1	ENST00000373736.2	Na+/K+ transporting ATPase interacting 1	10	1	1	0	0	0hsa-miR-27a-3p	-0.26	-0.26N/A
RELT	ENST00000064780.2	RELT tumor necrosis factor receptor	412	1	1	0	0	0hsa-miR-27a-3p	-0.26	-0.26N/A
ZNF462	ENST00000277225.5	zinc finger protein 462	214	1	0	1	0	0hsa-miR-27a-3p	-0.26	-0.3N/A
EEPD1	ENST00000242108.4	endonuclease/exonuclease/phosphatase family domain containing 1	39	1	0	1	0	0hsa-miR-27a-3p	-0.26	-0.27N/A
CFDP1	ENST00000283882.3	craniofacial development protein 1	482	1	0	0	1	1hsa-miR-27a-3p	-0.26	-0.27N/A
BAZ2B	ENST00000392782.1	bromodomain adjacent to zinc finger domain, 2B	25	1	0	0	1	0hsa-miR-27a-3p	-0.26	-0.4N/A
AP2B1	ENST00000262325.7	adaptor-related protein complex 2, beta 1 subunit	353	1	0	0	1	1hsa-miR-27a-3p	-0.26	-0.26N/A
NFKBID	ENST00000396901.1	nuclear factor of kappa light polypeptide gene enhancer in B-cells inhibitor, delta	40	2	0	0	2	2hsa-miR-27a-3p	-0.26	-0.41N/A
GPR75	ENST00000394705.2	G protein-coupled receptor 75	35	2	1	1	0	0hsa-miR-27a-3p	-0.26	-0.31N/A
SMAD5	ENST00000545279.1	SMAD family member 5	225	1	0	1	0	1hsa-miR-27a-3p	-0.26	-0.27N/A
KHSRP	ENST00000398148.3	KH-type splicing regulatory protein	4469	1	0	0	1	0hsa-miR-27a-3p	-0.26	-0.26N/A
LPPR1	ENST00000374874.3	Lipid phosphate phosphatase-related protein type 1	19	1	0	1	0	0hsa-miR-27a-3p	-0.26	-0.26N/A
PRKCD	ENST00000394729.2	protein kinase C, delta	2225	1	0	0	1	0hsa-miR-27a-3p	-0.26	-0.26N/A
SOCS6	ENST00000397942.3	suppressor of cytokine signaling 6	140	1	0	1	0	2hsa-miR-27a-3p	-0.26	-0.27N/A
CYSLTR2	ENST00000282018.3	cysteinyl leukotriene receptor 2	5	1	1	0	0	0hsa-miR-27a-3p	-0.26	-0.45N/A
NGFR	ENST00000172229.3	nerve growth factor receptor	5	1	1	0	0	1hsa-miR-27a-3p	-0.26	-0.26N/A
CACNB2	ENST00000396576.2	calcium channel, voltage-dependent, beta 2 subunit	52	1	0	1	0	0hsa-miR-27a-3p	-0.26	-0.26N/A
PRKCB	ENST00000303531.7	protein kinase C, beta	81	1	1	0	0	0hsa-miR-27a-3p	-0.26	-0.26N/A
LITAF	ENST00000413364.2	lipopolysaccharide-induced TNF factor	11008	1	0	1	0	1hsa-miR-27a-3p	-0.26	-0.26N/A
AMD1	ENST00000368885.3	adenosylmethionine decarboxylase 1	725	1	0	0	1	3hsa-miR-27a-3p	-0.26	-0.31N/A
SMOC2	ENST00000356284.2	SPARC related modular calcium binding 2	5	1	0	0	1	1hsa-miR-27a-3p	-0.26	-0.26N/A
PRMT8	ENST00000382622.3	protein arginine methyltransferase 8	5	1	0	1	0	0hsa-miR-27a-3p	-0.26	-0.26N/A
SS18L1	ENST00000331758.3	synovial sarcoma translocation gene on chromosome 18-like 1	274	1	1	0	0	0hsa-miR-27a-3p	-0.26	-0.32N/A
SIK1	ENST00000270162.6	salt-inducible kinase 1	2163	1	1	0	0	0hsa-miR-27a-3p	-0.26	-0.26N/A
ZCCHC24	ENST00000372336.3	zinc finger, CCHC domain containing 24	359	1	1	0	0	0hsa-miR-27a-3p	-0.26	-0.26N/A
AIDA	ENST00000340020.6	axin interactor, dorsalization associated	48	1	0	0	1	1hsa-miR-27a-3p	-0.26	-0.26N/A
ARID2	ENST00000457135.1	AT rich interactive domain 2 (ARID, RFX-like)	891	1	0	1	0	0hsa-miR-27a-3p	-0.26	-0.26N/A
FBLN5	ENST00000267620.10	fibulin 5	146	1	1	0	0	0hsa-miR-27a-3p	-0.26	-0.26N/A
RNF146	ENST00000356799.2	ring finger protein 146	1504	1	0	1	0	1hsa-miR-27a-3p	-0.25	-0.26N/A
SCN1A	ENST00000423058.2	sodium channel, voltage-gated, type I, alpha subunit	21	2	0	1	1	1hsa-miR-27a-3p	-0.25	-0.25N/A
TOMM40L	ENST00000367987.1	translocase of outer mitochondrial membrane 40 homolog (yeast)-like	13	1	1	0	0	0hsa-miR-27a-3p	-0.25	-0.25N/A
CABLES2	ENST00000279101.5	Cdk5 and Abl enzyme substrate 2	450	1	1	0	0	1hsa-miR-27a-3p	-0.25	-0.26N/A
EFHD2	ENST00000375980.4	EF-hand domain family, member D2	1411	1	0	0	1	0hsa-miR-27a-3p	-0.25	-0.25N/A
TAPT1	ENST00000405303.2	transmembrane anterior posterior transformation 1	105	1	1	0	0	0hsa-miR-27a-3p	-0.25	-0.25N/A
STK39	ENST00000355999.4	serine threonine kinase 39	266	1	1	0	0	0hsa-miR-27a-3p	-0.25	-0.34N/A
ARX	ENST00000379044.4	aristaless related homeobox	5	1	0	1	0	0hsa-miR-27a-3p	-0.25	-0.25N/A
RNF182	ENST00000488300.1	ring finger protein 182	1122	1	0	1	0	0hsa-miR-27a-3p	-0.25	-0.3N/A
MOSPD3	ENST00000393950.2	motile sperm domain containing 3	106	1	0	1	0	0hsa-miR-27a-3p	-0.25	-0.25N/A
GCNT2	ENST00000316170.3	glucosaminyl (N-acetyl) transferase 2, I-branching enzyme (I blood group)	651	1	1	0	0	1hsa-miR-27a-3p	-0.25	-0.25N/A
ABCA1	ENST00000374736.3	ATP-binding cassette, sub-family A (ABC1), member 1	184	2	2	0	0	1hsa-miR-27a-3p	-0.25	-0.25N/A
STX16	ENST00000355957.5	syntaxin 16	756	1	1	0	0	0hsa-miR-27a-3p	-0.25	-0.45N/A
H3F3B	ENST00000591890.1	H3 histone, family 3B (H3.3B)	336	1	0	1	0	1hsa-miR-27a-3p	-0.25	-0.32N/A
ZNF579	ENST00000325421.4	zinc finger protein 579	1080	1	0	0	1	0hsa-miR-27a-3p	-0.25	-0.25N/A

TMEM123	ENST00000361236.3	transmembrane protein 123	2135	1	0	1	0	0hsa-miR-27a-3p	-0.25	-0.25N/A
MTSS1L	ENST00000338779.6	metastasis suppressor 1-like	2982	1	1	0	0	2hsa-miR-27a-3p	-0.25	-0.25N/A
GRM5	ENST00000418177.2	glutamate receptor, metabotropic 5	5	1	1	0	0	2hsa-miR-27a-3p	-0.25	-0.25N/A
VASH2	ENST00000366968.4	vasohibin 2	70	1	0	1	0	0hsa-miR-27a-3p	-0.25	-0.26N/A
TMEM170B	ENST00000379426.1	transmembrane protein 170B	489	2	1	0	1	2hsa-miR-27a-3p	-0.25	-0.39N/A
DEPDC1B	ENST00000265036.5	DEP domain containing 1B	449	1	0	1	0	0hsa-miR-27a-3p	-0.25	-0.28N/A
FOXP4	ENST00000373063.3	forkhead box P4	228	1	1	0	0	1hsa-miR-27a-3p	-0.25	-0.25N/A
MSL1	ENST00000398532.4	male-specific lethal 1 homolog (Drosophila)	4715	1	1	0	0	2hsa-miR-27a-3p	-0.25	-0.25N/A
HOXC6	ENST00000243108.4	homeobox C6	63	1	0	1	0	0hsa-miR-27a-3p	-0.25	-0.25N/A
DTNA	ENST00000283365.9	dystrobrevin, alpha	144	1	1	0	0	3hsa-miR-27a-3p	-0.25	-0.25N/A
RS1	ENST00000379984.3	retinoschisin 1	5	1	1	0	0	0hsa-miR-27a-3p	-0.25	-0.25N/A
SH3BGRL2	ENST00000369838.4	SH3 domain binding glutamic acid-rich protein like 2	32	1	0	1	0	2hsa-miR-27a-3p	-0.25	-0.26N/A
GATA2	ENST00000341105.2	GATA binding protein 2	231	1	1	0	0	2hsa-miR-27a-3p	-0.24	-0.24N/A
TRIM44	ENST00000299413.5	tripartite motif containing 44	4203	1	0	0	1	4hsa-miR-27a-3p	-0.24	-0.32N/A
SUMO2	ENST00000420826.2	small ubiquitin-like modifier 2	75	1	0	0	1	0hsa-miR-27a-3p	-0.24	-0.24N/A
TOPBP1	ENST00000260810.5	topoisomerase (DNA) II binding protein 1	102	1	0	1	0	0hsa-miR-27a-3p	-0.24	-0.24N/A
RYBP	ENST00000477973.2	RING1 and YY1 binding protein	342	1	1	0	0	1hsa-miR-27a-3p	-0.24	-0.28N/A
KPNA5	ENST00000368564.1	karyopherin alpha 5 (importin alpha 6)	76	1	1	0	0	0hsa-miR-27a-3p	-0.24	-0.32N/A
GPR133	ENST00000376682.4	G protein-coupled receptor 133	57	1	0	1	0	4hsa-miR-27a-3p	-0.24	-0.24N/A
HUNK	ENST00000270112.2	hormonally up-regulated Neu-associated kinase	85	1	1	0	0	2hsa-miR-27a-3p	-0.24	-0.26N/A
NFIB	ENST00000397575.3	nuclear factor I/B	1149	3	0	1	2	4hsa-miR-27a-3p	-0.24	-0.46N/A
SH3RF1	ENST00000284637.9	SH3 domain containing ring finger 1	2747	1	1	0	0	1hsa-miR-27a-3p	-0.24	-0.24N/A
RGS1	ENST00000367459.3	regulator of G-protein signaling 1	5	1	0	1	0	0hsa-miR-27a-3p	-0.24	-0.24N/A
GATA6	ENST00000269216.3	GATA binding protein 6	691	1	0	1	0	2hsa-miR-27a-3p	-0.24	-0.24N/A
HAT1	ENST00000392584.1	histone acetyltransferase 1	3017	1	0	0	1	0hsa-miR-27a-3p	-0.24	-0.4N/A
ZBTB42	ENST00000342537.7	zinc finger and BTB domain containing 42	182	1	1	0	0	2hsa-miR-27a-3p	-0.24	-0.26N/A
GPR135	ENST00000395116.1	G protein-coupled receptor 135	40	1	1	0	0	0hsa-miR-27a-3p	-0.24	-0.24N/A
MDFI	ENST00000373050.4	MyoD family inhibitor	14	1	1	0	0	0hsa-miR-27a-3p	-0.24	-0.39N/A
PPAP2B	ENST00000371250.3	phosphatidic acid phosphatase type 2B	81	1	0	1	0	4hsa-miR-27a-3p	-0.24	-0.25N/A
TMEM170A	ENST00000357613.4	transmembrane protein 170A	70	1	0	1	0	3hsa-miR-27a-3p	-0.24	-0.36N/A
SLC35F4	ENST00000556826.1	solute carrier family 35, member F4	5	1	0	0	1	0hsa-miR-27a-3p	-0.24	-0.24N/A
SRSF1	ENST00000258962.4	serine/arginine-rich splicing factor 1	1430	1	0	1	0	1hsa-miR-27a-3p	-0.23	-0.27N/A
BTG1	ENST00000256015.3	B-cell translocation gene 1, anti-proliferative	1677	1	0	0	1	2hsa-miR-27a-3p	-0.23	-0.35N/A
C1orf173	ENST00000326665.5	chromosome 1 open reading frame 173	5	1	1	0	0	0hsa-miR-27a-3p	-0.23	-0.23N/A
LIN7C	ENST00000278193.2	lin-7 homolog C (C. elegans)	1701	1	0	1	0	0hsa-miR-27a-3p	-0.23	-0.23N/A
ARHGAP21	ENST00000396432.2	Rho GTPase activating protein 21	10	1	1	0	0	0hsa-miR-27a-3p	-0.23	-0.23N/A
ITGA8	ENST00000378076.3	integrin, alpha 8	7	1	1	0	0	1hsa-miR-27a-3p	-0.23	-0.25N/A
HMGCR	ENST00000287936.4	3-hydroxy-3-methylglutaryl-CoA reductase	1385	1	0	0	1	0hsa-miR-27a-3p	-0.23	-0.29N/A
SP1	ENST00000426431.2	Sp1 transcription factor	402	1	0	1	0	2hsa-miR-27a-3p	-0.23	-0.23N/A
STAG1	ENST00000383202.2	stromal antigen 1	94	1	0	1	0	0hsa-miR-27a-3p	-0.23	-0.23N/A
KLF3	ENST00000261438.5	Kruppel-like factor 3 (basic)	515	1	1	0	0	1hsa-miR-27a-3p	-0.23	-0.5N/A
CDK18	ENST00000360066.2	cyclin-dependent kinase 18	262	1	1	0	0	1hsa-miR-27a-3p	-0.23	-0.23N/A
MIER2	ENST00000264819.4	mesoderm induction early response 1, family member 2	137	1	1	0	0	0hsa-miR-27a-3p	-0.23	-0.23N/A
KLHL31	ENST00000370905.3	kelch-like family member 31	16	1	1	0	0	2hsa-miR-27a-3p	-0.23	-0.23N/A
NEURL1B	ENST00000369800.5	neuralized homolog 1B (Drosophila)	5	1	0	1	0	4hsa-miR-27a-3p	-0.23	-0.23N/A
DUSP5	ENST00000369583.3	dual specificity phosphatase 5	600	1	0	1	0	0hsa-miR-27a-3p	-0.23	-0.23N/A
RNF144A	ENST00000320892.6	ring finger protein 144A	124	1	0	1	0	4hsa-miR-27a-3p	-0.23	-0.24N/A
NRG1	ENST00000341377.5	neuregulin 1	113	1	0	1	0	1hsa-miR-27a-3p	-0.23	-0.23N/A
FBXO33	ENST00000298097.7	F-box protein 33	306	1	1	0	0	1hsa-miR-27a-3p	-0.23	-0.23N/A
ICOS	ENST00000435193.1	inducible T-cell co-stimulator	5	1	0	0	1	0hsa-miR-27a-3p	-0.23	-0.23N/A
SREK1	ENST00000334121.6	splicing regulatory glutamine/lysine-rich protein 1	316	1	0	1	0	0hsa-miR-27a-3p	-0.23	-0.26N/A
SFSWAP	ENST00000261674.4	splicing factor, suppressor of white-apricot homolog (Drosophila)	134	1	0	0	1	0hsa-miR-27a-3p	-0.23	-0.23N/A
MICU3	ENST00000318063.5	mitochondrial calcium uptake family, member 3	9	1	1	0	0	0hsa-miR-27a-3p	-0.23	-0.23N/A
LPIN1	ENST00000256720.2	lipin 1	357	1	0	0	1	0hsa-miR-27a-3p	-0.23	-0.23N/A
NUP153	ENST00000262077.2	nucleoporin 153kDa	231	1	0	0	1	0hsa-miR-27a-3p	-0.23	-0.23N/A

GRIK3	ENST00000373091.3	glutamate receptor, ionotropic, kainate 3	7	1	0	1	0	3hsa-miR-27a-3p	-0.23	-0.23N/A
CALD1	ENST00000361388.2	caldesmon 1	1013	2	0	0	2	0hsa-miR-27a-3p	-0.22	-0.25N/A
INSM2	ENST00000307169.3	insulinoma-associated 2	5	1	0	0	1	0hsa-miR-27a-3p	-0.22	-0.22N/A
BBC3	ENST00000341983.4	BCL2 binding component 3	87	1	1	0	0	0hsa-miR-27a-3p	-0.22	-0.22N/A
FAM78A	ENST00000372269.3	family with sequence similarity 78, member A	7	1	1	0	0	2hsa-miR-27a-3p	-0.22	-0.34N/A
ZFP36L1	ENST00000555997.1	ZFP36 ring finger protein-like 1	231	1	0	1	0	1hsa-miR-27a-3p	-0.22	-0.26N/A
TMX1	ENST00000457354.2	thioredoxin-related transmembrane protein 1	3176	1	0	1	0	0hsa-miR-27a-3p	-0.22	-0.43N/A
EN2	ENST00000297375.4	engrailed homeobox 2	86	1	1	0	0	1hsa-miR-27a-3p	-0.22	-0.31N/A
UBR5	ENST00000520539.1	ubiquitin protein ligase E3 component n-recognin 5	733	1	0	1	0	0hsa-miR-27a-3p	-0.22	-0.22N/A
RPN2	ENST00000237530.6	ribophorin II	14435	1	0	0	1	1hsa-miR-27a-3p	-0.22	-0.24N/A
ENAH	ENST00000366844.3	enabled homolog (Drosophila)	415	1	0	1	0	4hsa-miR-27a-3p	-0.22	-0.3N/A
SORL1	ENST00000260197.7	sortilin-related receptor, L(DLR class) A repeats containing	40	2	1	1	0	1hsa-miR-27a-3p	-0.22	-0.23N/A
SNN	ENST00000329565.5	stannin	663	1	0	0	1	0hsa-miR-27a-3p	-0.22	-0.22N/A
BLOC1S5	ENST00000397457.2	biogenesis of lysosomal organelles complex-1, subunit 5, muted	187	1	0	1	0	0hsa-miR-27a-3p	-0.22	-0.22N/A
SMURF2	ENST00000262435.9	SMAD specific E3 ubiquitin protein ligase 2	978	1	0	1	0	0hsa-miR-27a-3p	-0.22	-0.22N/A
RAP2B	ENST00000323534.2	RAP2B, member of RAS oncogene family	594	2	0	0	2	1hsa-miR-27a-3p	-0.22	-0.41N/A
CPEB4	ENST00000265085.5	cytoplasmic polyadenylation element binding protein 4	472	1	0	1	0	0hsa-miR-27a-3p	-0.22	-0.35N/A
KLHL29	ENST00000486442.1	kelch-like family member 29	414	1	0	0	1	0hsa-miR-27a-3p	-0.22	-0.22N/A
PDK1	ENST00000282077.3	pyruvate dehydrogenase kinase, isozyme 1	637	1	0	1	0	1hsa-miR-27a-3p	-0.22	-0.53N/A
STIM2	ENST00000467011.1	stromal interaction molecule 2	380	1	0	1	0	0hsa-miR-27a-3p	-0.22	-0.22N/A
HNRNPF	ENST00000357065.4	heterogeneous nuclear ribonucleoprotein F	240	1	0	1	0	0hsa-miR-27a-3p	-0.22	-0.34N/A
ATP8B2	ENST00000368489.3	ATPase, aminophospholipid transporter, class I, type 8B, member 2	244	1	0	1	0	0hsa-miR-27a-3p	-0.22	-0.22N/A
MRPS14	ENST00000476371.1	mitochondrial ribosomal protein S14	2140	1	0	0	1	0hsa-miR-27a-3p	-0.22	-0.23N/A
AGO2	ENST00000220592.5	argonaute RISC catalytic component 2	350	3	0	2	1	7hsa-miR-27a-3p	-0.22	-4.19N/A
SGPP1	ENST00000247225.6	sphingosine-1-phosphate phosphatase 1	1340	1	0	1	0	0hsa-miR-27a-3p	-0.22	-0.22N/A
KIF3A	ENST00000378746.4	kinesin family member 3A	33	2	1	0	1	0hsa-miR-27a-3p	-0.22	-0.26N/A
BRPF3	ENST00000357641.6	bromodomain and PHD finger containing, 3	7032	2	1	0	1	1hsa-miR-27a-3p	-0.21	-0.21N/A
SRPK2	ENST00000393651.3	SRSF protein kinase 2	381	1	0	1	0	2hsa-miR-27a-3p	-0.21	-0.22N/A
RBBP5	ENST00000264515.6	retinoblastoma binding protein 5	166	1	0	0	1	0hsa-miR-27a-3p	-0.21	-0.46N/A
NOL4	ENST00000261592.5	nucleolar protein 4	12	1	0	1	0	0hsa-miR-27a-3p	-0.21	-0.21N/A
PNRC2	ENST00000334351.7	proline-rich nuclear receptor coactivator 2	100	1	0	1	0	0hsa-miR-27a-3p	-0.21	-0.21N/A
KSR1	ENST00000398988.3	kinase suppressor of ras 1	256	3	3	0	0	1hsa-miR-27a-3p	-0.21	-0.22N/A
HMBOX1	ENST00000397358.3	homeobox containing 1	27	1	0	0	1	2hsa-miR-27a-3p	-0.21	-0.37N/A
PDGFRA	ENST00000257290.5	platelet-derived growth factor receptor, alpha polypeptide	39	1	0	1	0	2hsa-miR-27a-3p	-0.21	-0.21N/A
RUNX1T1	ENST00000523629.1	runt-related transcription factor 1; translocated to, 1 (cyclin D-related)	9	1	0	0	1	3hsa-miR-27a-3p	-0.21	-0.21N/A
BMP2K	ENST00000335016.5	BMP2 inducible kinase	164	2	0	2	0	0hsa-miR-27a-3p	-0.21	-0.22N/A
PRELID2	ENST00000334744.4	PRELI domain containing 2	63	1	0	1	0	2hsa-miR-27a-3p	-0.21	-0.29N/A
SHB	ENST00000377707.3	Src homology 2 domain containing adaptor protein B	1436	1	0	0	1	1hsa-miR-27a-3p	-0.21	-0.21N/A
POU3F2	ENST00000328345.5	POU class 3 homeobox 2	274	1	0	1	0	1hsa-miR-27a-3p	-0.21	-0.21N/A
CLCN3	ENST00000513761.1	chloride channel, voltage-sensitive 3	1142	2	0	2	0	1hsa-miR-27a-3p	-0.21	-0.37N/A
TMEM110	ENST00000355083.5	transmembrane protein 110	306	1	0	1	0	1hsa-miR-27a-3p	-0.21	-0.23N/A
EPB41	ENST00000373798.1	erythrocyte membrane protein band 4.1 (elliptocytosis 1, RH-linked)	53	1	1	0	0	0hsa-miR-27a-3p	-0.21	-0.21N/A
FGF14	ENST00000376131.4	fibroblast growth factor 14	5	1	0	1	0	1hsa-miR-27a-3p	-0.21	-0.21N/A
INPP5J	ENST00000331075.5	inositol polyphosphate-5-phosphatase J	70	1	0	1	0	0hsa-miR-27a-3p	-0.21	-0.21N/A
CASP8	ENST00000392259.2	caspase 8, apoptosis-related cysteine peptidase	64	1	1	0	0	0hsa-miR-27a-3p	-0.21	-0.21N/A
LPL	ENST00000311322.8	lipoprotein lipase	7658	1	1	0	0	0hsa-miR-27a-3p	-0.21	-0.21N/A
FOXO1	ENST00000379561.5	forkhead box O1	480	1	0	1	0	0hsa-miR-27a-3p	-0.21	-0.21N/A
CHST1	ENST00000308064.2	carbohydrate (keratan sulfate Gal-6) sulfotransferase 1	5	1	0	1	0	1hsa-miR-27a-3p	-0.21	-0.21N/A
HIC1	ENST00000399849.3	hypermethylated in cancer 1	76	1	1	0	0	0hsa-miR-27a-3p	-0.21	-0.21N/A
FNDC4	ENST00000264703.3	fibronectin type III domain containing 4	418	1	0	1	0	0hsa-miR-27a-3p	-0.21	-0.21N/A
ZMAT3	ENST00000311417.2	zinc finger, matrin-type 3	459	1	1	0	0	1hsa-miR-27a-3p	-0.21	-0.37N/A
ACVR1C	ENST00000243349.8	activin A receptor, type IC	27	2	1	0	1	0hsa-miR-27a-3p	-0.21	-0.41N/A
EMCN	ENST00000296420.4	endomucin	5	1	1	0	0	0hsa-miR-27a-3p	-0.21	-0.21N/A
UGT8	ENST00000310836.6	UDP glycosyltransferase 8	682	1	0	1	0	0hsa-miR-27a-3p	-0.21	-0.24N/A

AGFG1	ENST00000310078.8	ArfGAP with FG repeats 1	2019	2	0	1	1	2hsa-miR-27a-3p	-0.21	-0.21N/A
SOX7	ENST00000304501.1	SRY (sex determining region Y)-box 7	47	1	0	1	0	1hsa-miR-27a-3p	-0.21	-0.21N/A
FCRL1	ENST00000368176.3	Fc receptor-like 1	5	1	1	0	0	1hsa-miR-27a-3p	-0.21	-0.21N/A
DIRAS1	ENST00000323469.4	DIRAS family, GTP-binding RAS-like 1	49	1	0	1	0	1hsa-miR-27a-3p	-0.21	-0.21N/A
FOXP2	ENST00000408937.3	forkhead box P2	169	1	0	1	0	0hsa-miR-27a-3p	-0.21	-0.21N/A
LYPD3	ENST00000244333.3	LY6/PLAUR domain containing 3	13	1	0	1	0	0hsa-miR-27a-3p	-0.21	-0.21N/A
CBLB	ENST00000264122.4	Cbl proto-oncogene B, E3 ubiquitin protein ligase	469	1	0	0	1	0hsa-miR-27a-3p	-0.21	-0.29N/A
SLC7A11	ENST00000280612.5	solute carrier family 7 (anionic amino acid transporter light chain, xc- system), member 11	61	1	1	0	0	2hsa-miR-27a-3p	-0.21	-0.35N/A
FAM222A	ENST00000538780.1	family with sequence similarity 222, member A	23	1	0	1	0	0hsa-miR-27a-3p	-0.2	-0.3N/A
LDLR	ENST00000558518.1	low density lipoprotein receptor	1462	1	0	1	0	1hsa-miR-27a-3p	-0.2	-0.27N/A
MITF	ENST00000328528.6	microphthalmia-associated transcription factor	593	1	0	0	1	1hsa-miR-27a-3p	-0.2	-0.2N/A
EHD3	ENST00000322054.5	EH-domain containing 3	206	1	0	1	0	1hsa-miR-27a-3p	-0.2	-0.2N/A
KIAA0247	ENST00000342745.4	KIAA0247	161	1	1	0	0	2hsa-miR-27a-3p	-0.2	-0.2N/A
MESDC1	ENST00000267984.2	mesoderm development candidate 1	84	1	0	1	0	0hsa-miR-27a-3p	-0.2	-0.2N/A
PLEKHH2	ENST00000282406.4	pleckstrin homology domain containing, family H (with MyTH4 domain) member 2	9	1	0	1	0	0hsa-miR-27a-3p	-0.2	-0.2N/A
ANKRD40	ENST00000285243.6	ankyrin repeat domain 40	409	1	1	0	0	0hsa-miR-27a-3p	-0.2	-0.49N/A
GSPT2	ENST00000340438.4	G1 to S phase transition 2	97	1	0	1	0	0hsa-miR-27a-3p	-0.2	-0.2N/A
PAQR7	ENST00000374296.3	progestin and adipoQ receptor family member VII	106	1	0	1	0	0hsa-miR-27a-3p	-0.2	-0.2N/A
PDE3B	ENST00000282096.4	phosphodiesterase 3B, cGMP-inhibited	258	1	0	1	0	0hsa-miR-27a-3p	-0.2	-0.2N/A
RPS6KB1	ENST00000225577.4	ribosomal protein S6 kinase, 70kDa, polypeptide 1	16	1	1	0	0	1hsa-miR-27a-3p	-0.2	-0.2N/A
F3	ENST00000334047.7	coagulation factor III (thromboplastin, tissue factor)	13568	1	0	1	0	0hsa-miR-27a-3p	-0.2	-0.2N/A
SEC24A	ENST00000398844.2	SEC24 family, member A (S. cerevisiae)	679	1	0	1	0	1hsa-miR-27a-3p	-0.2	-0.2N/A
KCNK5	ENST00000359534.3	potassium channel, subfamily K, member 5	143	1	0	0	1	1hsa-miR-27a-3p	-0.2	-0.44N/A
WNK1	ENST00000315939.6	WNK lysine deficient protein kinase 1	950	1	0	1	0	0hsa-miR-27a-3p	-0.2	-0.2N/A
PHF13	ENST00000377648.4	PHD finger protein 13	1207	1	0	0	1	1hsa-miR-27a-3p	-0.2	-0.2N/A
ID4	ENST00000378700.3	inhibitor of DNA binding 4, dominant negative helix-loop-helix protein	3400	1	0	1	0	0hsa-miR-27a-3p	-0.2	-0.62N/A
CAMK2D	ENST00000296402.5	calcium/calmodulin-dependent protein kinase II delta	938	1	0	0	1	4hsa-miR-27a-3p	-0.2	-0.2N/A
LHX8	ENST00000294638.5	LIM homeobox 8	18	1	0	0	1	0hsa-miR-27a-3p	-0.2	-0.2N/A
NAA50	ENST00000240922.3	N(alpha)-acetyltransferase 50, NatE catalytic subunit	260	1	0	1	0	5hsa-miR-27a-3p	-0.2	-0.27N/A
NPTN	ENST00000345330.4	neuroplastin	2918	1	0	0	1	1hsa-miR-27a-3p	-0.2	-0.2N/A
KIAA1644	ENST00000381176.4	KIAA1644	10	1	0	1	0	2hsa-miR-27a-3p	-0.2	-0.21N/A
CTDSP2	ENST00000398073.2	CTD (carboxy-terminal domain, RNA polymerase II, polypeptide A) small phosphatase 2	991	1	0	1	0	0hsa-miR-27a-3p	-0.2	-0.2N/A
MEF2C	ENST00000340208.5	myocyte enhancer factor 2C	70	1	0	0	1	0hsa-miR-27a-3p	-0.2	-0.23N/A
STAT1	ENST00000540176.1	signal transducer and activator of transcription 1, 91kDa	326	1	1	0	0	1hsa-miR-27a-3p	-0.19	-0.2N/A
HCN4	ENST00000261917.3	hyperpolarization activated cyclic nucleotide-gated potassium channel 4	19	1	0	1	0	0hsa-miR-27a-3p	-0.19	-0.19N/A
SURF4	ENST00000545297.1	surfeit 4	3667	1	0	1	0	1hsa-miR-27a-3p	-0.19	-0.21N/A
CCDC92	ENST00000238156.3	coiled-coil domain containing 92	105	1	0	1	0	0hsa-miR-27a-3p	-0.19	-0.19N/A
SKOR1	ENST00000341418.5	SKI family transcriptional corepressor 1	22	1	0	1	0	0hsa-miR-27a-3p	-0.19	-0.19N/A
RBM12B	ENST00000399300.2	RNA binding motif protein 12B	143	1	0	0	1	1hsa-miR-27a-3p	-0.19	-0.19N/A
UBE2W	ENST00000517608.1	ubiquitin-conjugating enzyme E2W (putative)	102	1	0	1	0	0hsa-miR-27a-3p	-0.19	-0.2N/A
STRBP	ENST00000447404.2	spermatid perinuclear RNA binding protein	575	1	0	0	1	1hsa-miR-27a-3p	-0.19	-0.2N/A
HEG1	ENST00000311127.4	heart development protein with EGF-like domains 1	380	1	1	0	0	3hsa-miR-27a-3p	-0.19	-0.19N/A
B4GALNT4	ENST00000329962.6	beta-1,4-N-acetyl-galactosaminyl transferase 4	3266	1	0	0	1	0hsa-miR-27a-3p	-0.19	-0.19N/A
FBLN2	ENST00000404922.3	fibulin 2	59	1	0	1	0	0hsa-miR-27a-3p	-0.19	-0.19N/A
CCDC120	ENST00000376396.3	coiled-coil domain containing 120	170	1	0	1	0	2hsa-miR-27a-3p	-0.19	-0.19N/A
FAM13A	ENST00000395002.2	family with sequence similarity 13, member A	441	1	0	1	0	0hsa-miR-27a-3p	-0.19	-0.19N/A
YES1	ENST00000577961.1	v-yes-1 Yamaguchi sarcoma viral oncogene homolog 1	168	1	0	1	0	0hsa-miR-27a-3p	-0.19	-0.2N/A
ZKSCAN2	ENST00000328086.7	zinc finger with KRAB and SCAN domains 2	81	1	0	1	0	2hsa-miR-27a-3p	-0.19	-0.2N/A
LPHN2	ENST00000370715.1	latrophilin 2	407	1	0	0	1	1hsa-miR-27a-3p	-0.19	-0.25N/A
FAM69A	ENST00000370310.4	family with sequence similarity 69, member A	171	1	0	0	1	0hsa-miR-27a-3p	-0.19	-0.19N/A
G6PC3	ENST00000269097.4	glucose 6 phosphatase, catalytic, 3	429	1	0	1	0	3hsa-miR-27a-3p	-0.19	-0.19N/A
IRS1	ENST00000305123.5	insulin receptor substrate 1	804	1	0	1	0	0hsa-miR-27a-3p	-0.19	-0.23N/A
GRSF1	ENST00000254799.6	G-rich RNA sequence binding factor 1	4414	1	1	0	0	1hsa-miR-27a-3p	-0.19	-0.35N/A
LSM12	ENST00000585388.1	LSM12 homolog (S. cerevisiae)	20	1	0	1	0	0hsa-miR-27a-3p	-0.19	-0.26N/A
PPP4C	ENST00000279387.7	protein phosphatase 4, catalytic subunit	59	1	0	1	0	0hsa-miR-27a-3p	-0.19	-0.19N/A

SH2D3C	ENST00000373277.4	SH2 domain containing 3C	5	1	0	1	0	0hsa-miR-27a-3p		-0.19	-0.19N/A
GSE1	ENST00000253458.7	Gse1 coiled-coil protein	133	1	0	0	1	2hsa-miR-27a-3p		-0.19	-0.19N/A
HIVEP3	ENST00000372583.1	human immunodeficiency virus type I enhancer binding protein 3	84	2	1	0	1	2hsa-miR-27a-3p		-0.19	-0.19N/A
ATF3	ENST00000366983.1	activating transcription factor 3	2704	1	0	0	1	1hsa-miR-27a-3p		-0.19	-0.19N/A
POLR3G	ENST00000504930.1	polymerase (RNA) III (DNA directed) polypeptide G (32kD)	67	1	1	0	0	0hsa-miR-27a-3p		-0.19	-0.19N/A
STK32B	ENST00000282908.5	serine/threonine kinase 32B	196	1	0	1	0	0hsa-miR-27a-3p		-0.19	-0.19N/A
FEM1B	ENST00000306917.4	fem-1 homolog b (C. elegans)	656	1	0	1	0	2hsa-miR-27a-3p		-0.19	-0.35N/A
TBC1D4	ENST00000377636.3	TBC1 domain family, member 4	96	1	1	0	0	1hsa-miR-27a-3p		-0.18	-0.18N/A
MFHAS1	ENST00000276282.6	malignant fibrous histiocytoma amplified sequence 1	130	1	0	1	0	0hsa-miR-27a-3p		-0.18	-0.18N/A
TRABD2B	ENST00000606738.2	TraB domain containing 2B	244	1	0	0	1	1hsa-miR-27a-3p		-0.18	-0.24N/A
AICDA	ENST00000229335.6	activation-induced cytidine deaminase	5	1	1	0	0	0hsa-miR-27a-3p		-0.18	-0.18N/A
SLC6A6	ENST00000454876.2	solute carrier family 6 (neurotransmitter transporter), member 6	280	1	0	1	0	1hsa-miR-27a-3p		-0.18	-0.2N/A
LRRC8B	ENST00000330947.2	leucine rich repeat containing 8 family, member B	326	1	0	1	0	1hsa-miR-27a-3p		-0.18	-0.18N/A
DCX	ENST00000356915.2	doublecortin	5	2	0	2	0	2hsa-miR-27a-3p		-0.18	-0.18N/A
OSBPL10	ENST00000396556.2	oxysterol binding protein-like 10	10	1	0	1	0	0hsa-miR-27a-3p		-0.18	-0.21N/A
TET1	ENST00000373644.4	tet methylcytosine dioxygenase 1	5	1	0	1	0	1hsa-miR-27a-3p		-0.18	-0.18N/A
KCNA6	ENST00000433855.1	potassium voltage-gated channel, shaker-related subfamily, member 6	5	1	0	1	0	1hsa-miR-27a-3p		-0.18	-0.18N/A
PCNX	ENST00000304743.2	pecanex homolog (Drosophila)	458	1	1	0	0	1hsa-miR-27a-3p		-0.18	-0.26N/A
LPPR5	ENST00000370188.3	Lipid phosphate phosphatase-related protein type 5	23	1	0	1	0	1hsa-miR-27a-3p		-0.18	-0.21N/A
FAM102A	ENST00000373095.1	family with sequence similarity 102, member A	85	1	1	0	0	1hsa-miR-27a-3p		-0.18	-0.18N/A
CD2AP	ENST00000359314.5	CD2-associated protein	145	1	0	1	0	0hsa-miR-27a-3p		-0.18	-0.19N/A
CASC3	ENST00000264645.7	cancer susceptibility candidate 3	493	1	0	1	0	0hsa-miR-27a-3p		-0.18	-0.18N/A
ITGA2	ENST00000296585.5	integrin, alpha 2 (CD49B, alpha 2 subunit of VLA-2 receptor)	50	1	1	0	0	1hsa-miR-27a-3p		-0.18	-0.18N/A
TBR1	ENST00000389554.3	T-box, brain, 1	5	1	0	0	1	0hsa-miR-27a-3p		-0.18	-0.18N/A
IKZF2	ENST00000457361.1	IKAROS family zinc finger 2 (Helios)	32	1	0	1	0	2hsa-miR-27a-3p		-0.18	-0.2N/A
LHX4	ENST00000263726.2	LIM homeobox 4	13	1	0	1	0	1hsa-miR-27a-3p		-0.18	-0.28N/A
TNIK	ENST00000436636.2	TRAF2 and NCK interacting kinase	106	1	0	1	0	0hsa-miR-27a-3p		-0.18	-0.18N/A
C1GALT1	ENST00000223122.3	core 1 synthase, glycoprotein-N-acetylgalactosamine 3-beta-galactosyltransferase, 1	44	1	0	0	1	1hsa-miR-27a-3p		-0.18	-0.18N/A
C17orf96	ENST00000325814.5	chromosome 17 open reading frame 96	19	1	0	0	1	0hsa-miR-27a-3p		-0.18	-0.18N/A
SOX7	ENST00000554914.1	Transcription factor SOX-7; Uncharacterized protein; cDNA FLJ58508, highly similar to Transcription factor SOX-7	47	1	0	1	0	1hsa-miR-27a-3p		-0.18	-0.2N/A
CDK14	ENST00000380050.3	cyclin-dependent kinase 14	226	1	0	0	1	1hsa-miR-27a-3p		-0.18	-0.18N/A
EDNRA	ENST00000339690.5	endothelin receptor type A	35	1	0	1	0	0hsa-miR-27a-3p		-0.18	-0.18N/A
ABCB9	ENST00000280560.8	ATP-binding cassette, sub-family B (MDR/TAP), member 9	13	1	1	0	0	1hsa-miR-27a-3p		-0.18	-0.18N/A
EDAR	ENST00000409271.1	ectodysplasin A receptor	7	1	0	1	0	1hsa-miR-27a-3p		-0.18	-0.18N/A
HMGCS1	ENST00000325110.6	3-hydroxy-3-methylglutaryl-CoA synthase 1 (soluble)	3730	1	0	0	1	1hsa-miR-27a-3p		-0.18	-0.19N/A
MICALCL	ENST00000256186.2	MICAL C-terminal like	62	1	1	0	0	0hsa-miR-27a-3p		-0.18	-0.25N/A
TMEM194B	ENST00000409150.3	transmembrane protein 194B	271	2	0	2	0	4hsa-miR-27a-3p		-0.18	-0.48N/A
DGKG	ENST00000265022.3	diacylglycerol kinase, gamma 90kDa	5	1	0	0	1	0hsa-miR-27a-3p		-0.18	-0.18N/A
TMEM248	ENST00000341567.4	transmembrane protein 248	3207	1	0	0	1	0hsa-miR-27a-3p		-0.17	-0.17N/A
GOLGA1	ENST00000373555.4	golgin A1	334	1	0	0	1	0hsa-miR-27a-3p		-0.17	-0.17N/A
SESN2	ENST00000253063.3	sestrin 2	854	1	1	0	0	0hsa-miR-27a-3p		-0.17	-0.17N/A
ANKRD12	ENST00000262126.4	ankyrin repeat domain 12	11	1	0	0	1	1hsa-miR-27a-3p		-0.17	-0.19N/A
SGMS1	ENST00000361781.2	sphingomyelin synthase 1	100	1	0	1	0	0hsa-miR-27a-3p		-0.17	-0.17N/A
LPCAT1	ENST00000283415.3	lysophosphatidylcholine acyltransferase 1	298	1	1	0	0	0hsa-miR-27a-3p		-0.17	-0.27N/A
FAM104A	ENST00000581110.1	family with sequence similarity 104, member A	2724	1	0	0	1	0hsa-miR-27a-3p		-0.17	-0.17N/A
THRB	ENST00000396671.2	thyroid hormone receptor, beta	55	1	0	1	0	2hsa-miR-27a-3p		-0.17	-0.26N/A
KIAA0319L	ENST00000325722.3	KIAA0319-like	2090	1	0	0	1	0hsa-miR-27a-3p		-0.17	-0.17N/A
LEP	ENST00000308868.4	leptin	5	1	0	1	0	0hsa-miR-27a-3p		-0.17	-0.17N/A
RGL2	ENST00000497454.1	ral guanine nucleotide dissociation stimulator-like 2	150	1	0	1	0	0hsa-miR-27a-3p		-0.17	-0.17N/A
KPNA3	ENST00000261667.3	karyopherin alpha 3 (importin alpha 4)	104	1	0	1	0	2hsa-miR-27a-3p		-0.17	-0.33N/A
LRP6	ENST00000261349.4	low density lipoprotein receptor-related protein 6	157	1	1	0	0	1hsa-miR-27a-3p		-0.17	-0.18N/A
TAOK1	ENST00000261716.3	TAO kinase 1	164	2	0	1	1	3hsa-miR-27a-3p		-0.17	-0.19N/A
AEBP2	ENST00000266508.9	AE binding protein 2	1228	1	0	0	1	0hsa-miR-27a-3p		-0.17	-0.17N/A
ZBTB39	ENST00000300101.2	zinc finger and BTB domain containing 39	7	1	1	0	0	2hsa-miR-27a-3p		-0.17	-0.17N/A

ELFN2	ENST00000402918.2	extracellular leucine-rich repeat and fibronectin type III domain containing 2	22	1	1	0	0	1hsa-miR-27a-3p	-0.17	-0.2N/A
WNT3A	ENST00000284523.1	wingless-type MMTV integration site family, member 3A	7	1	1	0	0	0hsa-miR-27a-3p	-0.17	-0.17N/A
PPP3R1	ENST00000234310.3	protein phosphatase 3, regulatory subunit B, alpha	117	1	0	0	1	1hsa-miR-27a-3p	-0.17	-0.17N/A
ARHGAP19-SLIT1	ENST00000453547.2	ARHGAP19-SLIT1 readthrough (NMD candidate)	15	2	0	2	0	7hsa-miR-27a-3p	-0.17	-0.17N/A
ADCY6	ENST00000357869.3	adenylate cyclase 6	6	1	1	0	0	0hsa-miR-27a-3p	-0.17	-0.17N/A
ANKIB1	ENST00000265742.3	ankyrin repeat and IBR domain containing 1	127	1	1	0	0	0hsa-miR-27a-3p	-0.17	-0.17N/A
IKZF5	ENST00000368886.5	IKAROS family zinc finger 5 (Pegasus)	83	1	1	0	0	2hsa-miR-27a-3p	-0.17	-0.39N/A
GNA13	ENST00000439174.2	guanine nucleotide binding protein (G protein), alpha 13	190	1	0	1	0	1hsa-miR-27a-3p	-0.17	-0.2N/A
CEP128	ENST00000281129.3	centrosomal protein 128kDa	280	1	0	1	0	1hsa-miR-27a-3p	-0.17	-0.17N/A
E2F6	ENST00000362009.4	E2F transcription factor 6	70	1	0	1	0	0hsa-miR-27a-3p	-0.17	-0.18N/A
UNC13C	ENST00000545554.1	unc-13 homolog C (C. elegans)	14	1	1	0	0	0hsa-miR-27a-3p	-0.17	-0.17N/A
VPS26B	ENST00000281187.5	vacuolar protein sorting 26 homolog B (S. pombe)	1236	1	1	0	0	0hsa-miR-27a-3p	-0.17	-0.18N/A
RGS6	ENST00000553530.1	regulator of G-protein signaling 6	5	1	1	0	0	1hsa-miR-27a-3p	-0.17	-0.17N/A
NRIP2	ENST00000337508.4	nuclear receptor interacting protein 2	5	1	1	0	0	2hsa-miR-27a-3p	-0.17	-0.17N/A
YAP1	ENST00000282441.5	Yes-associated protein 1	1175	1	0	0	1	0hsa-miR-27a-3p	-0.17	-0.18N/A
MAGI3	ENST00000307546.9	membrane associated guanylate kinase, WW and PDZ domain containing 3	70	2	1	1	0	0hsa-miR-27a-3p	-0.17	-0.44N/A
LCOR	ENST00000371103.3	ligand dependent nuclear receptor corepressor	94	2	0	2	0	0hsa-miR-27a-3p	-0.17	-0.17N/A
ANKRD17	ENST00000358602.4	ankyrin repeat domain 17	1334	1	0	0	1	0hsa-miR-27a-3p	-0.17	-0.17N/A
ZNF385A	ENST00000551109.1	zinc finger protein 385A	94	1	0	1	0	0hsa-miR-27a-3p	-0.17	-0.17N/A
RC3H1	ENST00000367696.2	ring finger and CCCH-type domains 1	165	2	0	0	2	1hsa-miR-27a-3p	-0.16	-0.18N/A
PTGFRN	ENST00000393203.2	prostaglandin F2 receptor inhibitor	591	1	0	1	0	2hsa-miR-27a-3p	-0.16	-0.35N/A
CAMK2A	ENST00000348628.6	calcium/calmodulin-dependent protein kinase II alpha	5	1	0	0	1	0hsa-miR-27a-3p	-0.16	-0.16N/A
KIAA1147	ENST00000536163.1	KIAA1147	73	1	1	0	0	1hsa-miR-27a-3p	-0.16	-0.25N/A
HNF4G	ENST00000396423.2	hepatocyte nuclear factor 4, gamma	5	1	0	1	0	0hsa-miR-27a-3p	-0.16	-0.16N/A
HLX	ENST00000366903.6	H2.0-like homeobox	28	1	0	1	0	0hsa-miR-27a-3p	-0.16	-0.46N/A
GPD1L	ENST00000282541.5	glycerol-3-phosphate dehydrogenase 1-like	130	1	0	0	1	0hsa-miR-27a-3p	-0.16	-0.18N/A
TBC1D8B	ENST00000357242.5	TBC1 domain family, member 8B (with GRAM domain)	69	1	1	0	0	1hsa-miR-27a-3p	-0.16	-0.17N/A
SDC2	ENST00000302190.4	syndecan 2	774	1	0	0	1	0hsa-miR-27a-3p	-0.16	-0.43N/A
LRBA	ENST00000535741.1	LPS-responsive vesicle trafficking, beach and anchor containing	603	1	0	0	1	0hsa-miR-27a-3p	-0.16	-0.16N/A
NT5C1A	ENST00000235628.1	5'-nucleotidase, cytosolic IA	4	2	0	2	0	0hsa-miR-27a-3p	-0.16	-0.16N/A
KPNB1	ENST00000290158.4	karyopherin (importin) beta 1	465	1	1	0	0	1hsa-miR-27a-3p	-0.16	-0.16N/A
KMT2C	ENST00000262189.6	lysine (K)-specific methyltransferase 2C	417	1	1	0	0	0hsa-miR-27a-3p	-0.16	-0.16N/A
SNAI1	ENST00000244050.2	snail family zinc finger 1	486	1	0	1	0	0hsa-miR-27a-3p	-0.16	-0.16N/A
FLRT2	ENST00000330753.4	fibronectin leucine rich transmembrane protein 2	73	1	0	1	0	3hsa-miR-27a-3p	-0.16	-0.19N/A
MVB12B	ENST00000361171.3	multivesicular body subunit 12B	29	1	1	0	0	0hsa-miR-27a-3p	-0.16	-0.16N/A
PDLIM2	ENST00000409141.1	PDZ and LIM domain 2 (mystique)	426	1	0	1	0	0hsa-miR-27a-3p	-0.16	-0.16N/A
NECAP1	ENST00000339754.5	NECAP endocytosis associated 1	13	1	0	0	1	1hsa-miR-27a-3p	-0.16	-0.16N/A
ATP2B1	ENST00000261173.2	ATPase, Ca++ transporting, plasma membrane 1	699	1	0	1	0	0hsa-miR-27a-3p	-0.16	-0.2N/A
RMI2	ENST00000576027.1	RecQ mediated genome instability 2	17	1	0	0	1	1hsa-miR-27a-3p	-0.16	-0.19N/A
CSF1	ENST00000329608.6	colony stimulating factor 1 (macrophage)	290	2	1	1	0	0hsa-miR-27a-3p	-0.16	-0.16N/A
CCDC71	ENST00000321895.6	coiled-coil domain containing 71	64	1	0	1	0	0hsa-miR-27a-3p	-0.16	-0.25N/A
GPR126	ENST00000230173.6	G protein-coupled receptor 126	604	1	0	1	0	0hsa-miR-27a-3p	-0.16	-0.16N/A
TM2D3	ENST00000347970.3	TM2 domain containing 3	458	1	0	0	1	2hsa-miR-27a-3p	-0.16	-0.29N/A
CDC42EP3	ENST00000295324.3	CDC42 effector protein (Rho GTPase binding) 3	1749	1	1	0	0	2hsa-miR-27a-3p	-0.16	-0.29N/A
ZZZ3	ENST00000370801.3	zinc finger, ZZ-type containing 3	338	1	0	1	0	0hsa-miR-27a-3p	-0.16	-0.18N/A
ANK2	ENST00000357077.4	ankyrin 2, neuronal	133	1	0	1	0	0hsa-miR-27a-3p	-0.16	-0.16N/A
UQCC1	ENST00000349714.5	ubiquinol-cytochrome c reductase complex assembly factor 1	500	1	0	0	1	0hsa-miR-27a-3p	-0.16	-0.21N/A
DLGAP3	ENST00000373347.1	discs, large (Drosophila) homolog-associated protein 3	8	1	1	0	0	1hsa-miR-27a-3p	-0.16	-0.16N/A
ENPEP	ENST00000265162.5	glutamyl aminopeptidase (aminopeptidase A)	10	1	0	0	1	0hsa-miR-27a-3p	-0.16	-0.18N/A
ARFGEF1	ENST00000262215.3	ADP-ribosylation factor guanine nucleotide-exchange factor 1 (brefeldin A-inhibited)	70	2	1	0	1	0hsa-miR-27a-3p	-0.16	-0.38N/A
ALDH1L2	ENST00000258494.9	aldehyde dehydrogenase 1 family, member L2	22	1	0	1	0	1hsa-miR-27a-3p	-0.16	-0.28N/A
FKBP14	ENST00000222803.5	FK506 binding protein 14, 22 kDa	939	1	0	1	0	0hsa-miR-27a-3p	-0.16	-0.2N/A
APC	ENST00000457016.1	adenomatous polyposis coli	794	1	0	0	1	0hsa-miR-27a-3p	-0.15	-0.15N/A
GRAMD1C	ENST00000358160.4	GRAM domain containing 1C	12	1	0	0	1	0hsa-miR-27a-3p	-0.15	-0.15N/A
WNT2B	ENST00000369686.5	wingless-type MMTV integration site family, member 2B	5	1	0	1	0	1hsa-miR-27a-3p	-0.15	-0.15N/A

ZC3H12B	ENST00000338957.4	zinc finger CCCH-type containing 12B	29	1	1	0	0	1hsa-miR-27a-3p	-0.15	-0.15N/A
CAPN15	ENST00000219611.2	calpain 15	5	1	0	0	1	0hsa-miR-27a-3p	-0.15	-0.15N/A
LIMK2	ENST00000331728.4	LIM domain kinase 2	233	1	0	0	1	1hsa-miR-27a-3p	-0.15	-0.15N/A
PDXK	ENST00000468090.1	pyridoxal (pyridoxine, vitamin B6) kinase	5653	1	0	0	1	2hsa-miR-27a-3p	-0.15	-0.15N/A
EPS8	ENST00000543523.1	epidermal growth factor receptor pathway substrate 8	2643	1	0	0	1	0hsa-miR-27a-3p	-0.15	-0.18N/A
GAREM	ENST00000399218.4	GRB2 associated, regulator of MAPK1	536	1	1	0	0	0hsa-miR-27a-3p	-0.15	-0.22N/A
PAPPA	ENST00000328252.3	pregnancy-associated plasma protein A, pappalysin 1	350	1	0	1	0	3hsa-miR-27a-3p	-0.15	-0.17N/A
ARF6	ENST00000298316.5	ADP-ribosylation factor 6	31	1	0	1	0	0hsa-miR-27a-3p	-0.15	-0.18N/A
RASGRF1	ENST00000558480.2	Ras protein-specific guanine nucleotide-releasing factor 1	5	1	0	0	1	0hsa-miR-27a-3p	-0.15	-0.15N/A
CYTH1	ENST00000585509.1	cytohesin 1	553	1	0	1	0	0hsa-miR-27a-3p	-0.15	-0.15N/A
TGFBR1	ENST00000374994.4	transforming growth factor, beta receptor 1	249	1	0	1	0	1hsa-miR-27a-3p	-0.15	-0.17N/A
GZF1	ENST00000338121.5	GDNF-inducible zinc finger protein 1	252	1	0	0	1	0hsa-miR-27a-3p	-0.15	-0.22N/A
WIPF2	ENST00000323571.4	WAS/WASL interacting protein family, member 2	214	1	1	0	0	0hsa-miR-27a-3p	-0.15	-0.19N/A
FAM134C	ENST00000309428.5	family with sequence similarity 134, member C	397	1	1	0	0	0hsa-miR-27a-3p	-0.15	-0.15N/A
TMEM91	ENST00000539627.1	transmembrane protein 91	231	1	1	0	0	0hsa-miR-27a-3p	-0.15	-0.15N/A
WWC1	ENST00000265293.4	WW and C2 domain containing 1	43	1	0	0	1	1hsa-miR-27a-3p	-0.15	-0.15N/A
AGRN	ENST00000379370.2	agrin	11	1	0	1	0	0hsa-miR-27a-3p	-0.15	-0.15N/A
CDH24	ENST00000397359.3	cadherin 24, type 2	44	1	0	1	0	2hsa-miR-27a-3p	-0.15	-0.15N/A
MANEAL	ENST00000397631.3	mannosidase, endo-alpha-like	381	1	0	1	0	1hsa-miR-27a-3p	-0.15	-0.16N/A
NR6A1	ENST00000487099.2	nuclear receptor subfamily 6, group A, member 1	1082	2	0	0	2	0hsa-miR-27a-3p	-0.15	-0.15N/A
CNOT1	ENST00000317147.5	CCR4-NOT transcription complex, subunit 1	294	1	0	0	1	0hsa-miR-27a-3p	-0.15	-0.15N/A
SOX8	ENST00000293894.3	SRY (sex determining region Y)-box 8	5	1	0	0	1	2hsa-miR-27a-3p	-0.15	-0.15N/A
ZNF783	ENST00000434415.1	zinc finger family member 783	88	1	1	0	0	0hsa-miR-27a-3p	-0.15	-0.15N/A
SENP1	ENST00000004980.5	SUMO1/sentrin specific peptidase 1	361	1	0	0	1	0hsa-miR-27a-3p	-0.15	-0.15N/A
NCS1	ENST00000372398.3	neuronal calcium sensor 1	1083	1	0	1	0	1hsa-miR-27a-3p	-0.15	-0.2N/A
SULT4A1	ENST00000330884.4	sulfotransferase family 4A, member 1	25	1	0	0	1	1hsa-miR-27a-3p	-0.15	-0.17N/A
MATN1	ENST00000373765.4	matrilin 1, cartilage matrix protein	5	1	0	0	1	0hsa-miR-27a-3p	-0.15	-0.15N/A
GPD2	ENST00000310454.6	glycerol-3-phosphate dehydrogenase 2 (mitochondrial)	196	2	2	0	0	0hsa-miR-27a-3p	-0.15	-0.15N/A
RAB3D	ENST00000222120.3	RAB3D, member RAS oncogene family	111	1	0	1	0	0hsa-miR-27a-3p	-0.15	-0.22N/A
GABRB3	ENST00000311550.5	gamma-aminobutyric acid (GABA) A receptor, beta 3	267	1	0	1	0	0hsa-miR-27a-3p	-0.15	-0.18N/A
SEC62	ENST00000337002.4	SEC62 homolog (S. cerevisiae)	746	1	0	0	1	2hsa-miR-27a-3p	-0.15	-0.46N/A
HAS2	ENST00000303924.4	hyaluronan synthase 2	398	1	0	0	1	0hsa-miR-27a-3p	-0.15	-0.18N/A
LSAMP	ENST00000490035.2	limbic system-associated membrane protein	19	1	0	0	1	2hsa-miR-27a-3p	-0.15	-0.66N/A
VANGL1	ENST00000355485.2	VANGL planar cell polarity protein 1	721	2	1	1	0	0hsa-miR-27a-3p	-0.15	-0.34N/A
C2CD4A	ENST00000355522.5	C2 calcium-dependent domain containing 4A	23	1	1	0	0	0hsa-miR-27a-3p	-0.15	-0.28N/A
PDE3A	ENST00000359062.3	phosphodiesterase 3A, cGMP-inhibited	1182	1	0	1	0	1hsa-miR-27a-3p	-0.15	-0.15N/A
FZD3	ENST00000240093.3	frizzled family receptor 3	192	1	0	1	0	2hsa-miR-27a-3p	-0.15	-0.17N/A
FGF1	ENST00000360966.5	fibroblast growth factor 1 (acidic)	88	1	0	1	0	2hsa-miR-27a-3p	-0.15	-0.37N/A
TMEM9B	ENST00000309134.5	TMEM9 domain family, member B	195	1	0	1	0	0hsa-miR-27a-3p	-0.15	-0.3N/A
RND3	ENST00000375734.2	Rho family GTPase 3	574	1	0	1	0	0hsa-miR-27a-3p	-0.15	-0.15N/A
PSEN1	ENST00000324501.5	presenilin 1	1340	1	1	0	0	1hsa-miR-27a-3p	-0.15	-0.22N/A
WDR37	ENST00000358220.1	WD repeat domain 37	107	1	0	0	1	1hsa-miR-27a-3p	-0.15	-0.24N/A
MAPK10	ENST00000395169.3	mitogen-activated protein kinase 10	5	1	1	0	0	1hsa-miR-27a-3p	-0.14	-0.14N/A
RAB33B	ENST00000305626.5	RAB33B, member RAS oncogene family	270	2	0	1	1	0hsa-miR-27a-3p	-0.14	-0.23N/A
DOT1L	ENST00000398665.3	DOT1-like histone H3K79 methyltransferase	439	2	2	0	0	2hsa-miR-27a-3p	-0.14	-0.15N/A
CAB39	ENST00000258418.5	calcium binding protein 39	27	1	0	1	0	0hsa-miR-27a-3p	-0.14	-0.25N/A
TGFBR3	ENST00000212355.4	transforming growth factor, beta receptor III	245	1	1	0	0	0hsa-miR-27a-3p	-0.14	-0.14N/A
IL10	ENST00000423557.1	interleukin 10	5	1	0	0	1	0hsa-miR-27a-3p	-0.14	-0.14N/A
PDPN	ENST00000294489.6	podoplanin	264	1	1	0	0	0hsa-miR-27a-3p	-0.14	-0.28N/A
ELAVL2	ENST00000380110.4	ELAV like neuron-specific RNA binding protein 2	78	1	0	1	0	1hsa-miR-27a-3p	-0.14	-0.15N/A
EIF5A2	ENST00000474096.1	eukaryotic translation initiation factor 5A2	443	1	1	0	0	0hsa-miR-27a-3p	-0.14	-0.56N/A
ZFHX4	ENST00000521891.2	zinc finger homeobox 4	194	1	0	1	0	0hsa-miR-27a-3p	-0.14	-0.14N/A
SLC39A13	ENST00000524928.1	solute carrier family 39 (zinc transporter), member 13	845	1	0	1	0	4hsa-miR-27a-3p	-0.14	-0.14N/A
GATAD2A	ENST00000360315.3	GATA zinc finger domain containing 2A	986	2	0	2	0	1hsa-miR-27a-3p	-0.14	-0.22N/A
GNB4	ENST00000232564.3	guanine nucleotide binding protein (G protein), beta polypeptide 4	343	1	0	0	1	0hsa-miR-27a-3p	-0.14	-0.14N/A
SLC35A3	ENST00000465289.1	solute carrier family 35 (UDP-N-acetylglucosamine (UDP-GlcNAc)	191	1	0	0	1	0hsa-miR-27a-3p	-0.14	-0.14N/A

		transporter), member A3								
RP11-192H23.4	ENST00000534850.1	Uncharacterized protein	22	1	0	1	0	0hsa-miR-27a-3p	-0.14	-0.14N/A
SCN3B	ENST00000392770.2	sodium channel, voltage-gated, type III, beta subunit	10	1	0	1	0	3hsa-miR-27a-3p	-0.14	-0.14N/A
MDN1	ENST00000369393.3	MDN1, midasin homolog (yeast)	42	1	1	0	0	0hsa-miR-27a-3p	-0.14	-0.14N/A
HGF	ENST00000222390.5	hepatocyte growth factor (hepapoietin A; scatter factor)	18	1	0	0	1	1hsa-miR-27a-3p	-0.14	-0.14N/A
FAM98A	ENST00000403368.1	family with sequence similarity 98, member A	89	1	0	0	1	0hsa-miR-27a-3p	-0.14	-0.14N/A
DCAF12	ENST00000361264.4	DDB1 and CUL4 associated factor 12	2075	1	0	0	1	0hsa-miR-27a-3p	-0.14	-0.14N/A
MEIS2	ENST00000397624.3	Meis homeobox 2	63	1	0	1	0	1hsa-miR-27a-3p	-0.14	-0.17N/A
SERP1	ENST00000239944.2	stress-associated endoplasmic reticulum protein 1	3242	1	0	0	1	0hsa-miR-27a-3p	-0.14	-0.46N/A
SRGAP2	ENST00000414007.1	SLIT-ROBO Rho GTPase activating protein 2	98	2	1	1	0	1hsa-miR-27a-3p	-0.14	-0.16N/A
ZNF280C	ENST00000370978.4	zinc finger protein 280C	138	1	0	1	0	0hsa-miR-27a-3p	-0.14	-0.14N/A
CDK8	ENST00000381527.3	cyclin-dependent kinase 8	280	1	0	0	1	0hsa-miR-27a-3p	-0.14	-0.14N/A
CA7	ENST00000394069.3	carbonic anhydrase VII	5	1	0	1	0	0hsa-miR-27a-3p	-0.14	-0.14N/A
PMF1-BGLAP	ENST00000490491.1	PMF1-BGLAP readthrough	82	1	0	1	0	1hsa-miR-27a-3p	-0.14	-0.2N/A
MAP1B	ENST00000296755.7	microtubule-associated protein 1B	259	1	0	0	1	1hsa-miR-27a-3p	-0.14	-0.15N/A
EBF3	ENST00000368648.3	early B-cell factor 3	101	2	0	1	1	0hsa-miR-27a-3p	-0.14	-0.34N/A
SALL4	ENST00000217086.4	sal-like 4 (Drosophila)	155	1	1	0	0	0hsa-miR-27a-3p	-0.14	-0.14N/A
SNX18	ENST00000343017.6	sorting nexin 18	174	1	0	1	0	0hsa-miR-27a-3p	-0.14	-0.14N/A
RET	ENST00000355710.3	ret proto-oncogene	5	1	0	1	0	0hsa-miR-27a-3p	-0.14	-0.14N/A
SLC25A16	ENST00000609923.1	solute carrier family 25 (mitochondrial carrier; Graves disease autoantigen), member 16	206	1	1	0	0	0hsa-miR-27a-3p	-0.14	-0.41N/A
OPHN1	ENST00000355520.5	oligophrenin 1	54	1	1	0	0	3hsa-miR-27a-3p	-0.14	-0.14N/A
CTCF	ENST00000264010.4	CCCTC-binding factor (zinc finger protein)	1037	1	1	0	0	0hsa-miR-27a-3p	-0.14	-0.14N/A
KIAA1033	ENST00000332180.5	KIAA1033	238	1	0	1	0	0hsa-miR-27a-3p	-0.14	-0.14N/A
SBF2	ENST00000256190.8	SET binding factor 2	65	2	1	0	1	1hsa-miR-27a-3p	-0.14	-0.32N/A
PPFIA2	ENST00000549396.1	protein tyrosine phosphatase, receptor type, f polypeptide (PTPRF), interacting protein (liprin), alpha 2	5	1	0	1	0	0hsa-miR-27a-3p	-0.14	-0.14N/A
CBFA2T3	ENST00000327483.5	core-binding factor, runt domain, alpha subunit 2; translocated to, 3	7	1	0	1	0	1hsa-miR-27a-3p	-0.14	-0.2N/A
TNPO3	ENST00000393245.1	transportin 3	30	1	0	0	1	0hsa-miR-27a-3p	-0.14	-0.15N/A
HIP1	ENST00000336926.6	huntingtin interacting protein 1	37	1	1	0	0	0hsa-miR-27a-3p	-0.14	-0.17N/A
PLAA	ENST00000397292.3	phospholipase A2-activating protein	416	1	0	1	0	0hsa-miR-27a-3p	-0.14	-0.21N/A
SYDE1	ENST00000342784.2	synapse defective 1, Rho GTPase, homolog 1 (C. elegans)	249	1	0	1	0	1hsa-miR-27a-3p	-0.14	-0.14N/A
KBTBD11	ENST00000320248.3	kelch repeat and BTB (POZ) domain containing 11	173	1	0	1	0	0hsa-miR-27a-3p	-0.14	-0.14N/A
PAN3	ENST00000282391.5	PAN3 poly(A) specific ribonuclease subunit homolog (S. cerevisiae)	361	1	0	0	1	1hsa-miR-27a-3p	-0.14	-0.14N/A
NFAT5	ENST00000354436.2	nuclear factor of activated T-cells 5, tonicity-responsive	128	1	0	1	0	3hsa-miR-27a-3p	-0.14	-0.32N/A
DGKE	ENST00000284061.3	diacylglycerol kinase, epsilon 64kDa	470	2	0	1	1	0hsa-miR-27a-3p	-0.14	-0.16N/A
CYP1B1	ENST00000260630.3	cytochrome P450, family 1, subfamily B, polypeptide 1	111	1	0	0	1	1hsa-miR-27a-3p	-0.14	-0.15N/A
GALNT1	ENST00000269195.5	UDP-N-acetyl-alpha-D-galactosamine:polypeptide N-acetylgalactosaminyltransferase 1 (GalNAc-T1)	2466	1	0	0	1	1hsa-miR-27a-3p	-0.14	-0.16N/A
MAP2K7	ENST00000397981.3	mitogen-activated protein kinase kinase 7	164	1	1	0	0	0hsa-miR-27a-3p	-0.14	-0.14N/A
MOB1B	ENST00000309395.2	MOB kinase activator 1B	54	1	0	1	0	1hsa-miR-27a-3p	-0.14	-0.14N/A
ERI3	ENST00000372259.5	ERI1 exoribonuclease family member 3	540	1	0	0	1	0hsa-miR-27a-3p	-0.14	-0.14N/A
ALG8	ENST00000376156.3	ALG8, alpha-1,3-glucosyltransferase	2908	1	0	0	1	0hsa-miR-27a-3p	-0.14	-0.14N/A
BBX	ENST00000415149.2	bobby sox homolog (Drosophila)	141	1	0	1	0	0hsa-miR-27a-3p	-0.13	-0.26N/A
CELF2	ENST00000315874.4	CUGBP, Elav-like family member 2	56	3	1	0	2	1hsa-miR-27a-3p	-0.13	-0.21N/A
PLAG1	ENST00000316981.3	pleiomorphic adenoma gene 1	58	1	0	1	0	0hsa-miR-27a-3p	-0.13	-0.16N/A
KCNA7	ENST00000221444.1	potassium voltage-gated channel, shaker-related subfamily, member 7	11	1	0	0	1	2hsa-miR-27a-3p	-0.13	-0.13N/A
MSTN	ENST00000260950.4	myostatin	6	1	0	0	1	0hsa-miR-27a-3p	-0.13	-0.13N/A
BCOR	ENST00000397354.3	BCL6 corepressor	355	1	0	0	1	0hsa-miR-27a-3p	-0.13	-0.13N/A
EHF	ENST00000257831.3	ets homologous factor	5	3	0	2	1	1hsa-miR-27a-3p	-0.13	-0.55N/A
LOX	ENST00000231004.4	lysyl oxidase	3441	1	1	0	0	1hsa-miR-27a-3p	-0.13	-0.3N/A
MPP6	ENST00000222644.5	membrane protein, palmitoylated 6 (MAGUK p55 subfamily member 6)	389	1	0	1	0	4hsa-miR-27a-3p	-0.13	-0.24N/A
ARNTL	ENST00000389707.4	aryl hydrocarbon receptor nuclear translocator-like	456	1	0	0	1	0hsa-miR-27a-3p	-0.13	-0.13N/A
CAND1	ENST00000545606.1	cullin-associated and neddylation-dissociated 1	1568	1	1	0	0	1hsa-miR-27a-3p	-0.13	-0.35N/A
CBX5	ENST00000209875.4	chromobox homolog 5	360	1	0	0	1	1hsa-miR-27a-3p	-0.13	-0.19N/A
MYT1	ENST00000328439.1	myelin transcription factor 1	12	1	0	1	0	1hsa-miR-27a-3p	-0.13	-0.31N/A
INO80D	ENST00000403263.1	INO80 complex subunit D	5	2	0	1	1	4hsa-miR-27a-3p	-0.13	-0.13N/A
KIAA1199	ENST00000220244.3	KIAA1199	660	1	1	0	0	0hsa-miR-27a-3p	-0.13	-0.13N/A

MAPK8IP3	ENST00000250894.4	mitogen-activated protein kinase 8 interacting protein 3	100	1	0	1	0	0hsa-miR-27a-3p	-0.13	-0.13N/A
MMP13	ENST00000260302.3	matrix metallopeptidase 13 (collagenase 3)	5	1	0	0	1	0hsa-miR-27a-3p	-0.13	-0.13N/A
TSPYL5	ENST00000322128.3	TSPY-like 5	20	1	0	0	1	1hsa-miR-27a-3p	-0.13	-0.13N/A
CHERP	ENST00000546361.2	calcium homeostasis endoplasmic reticulum protein	614	1	0	0	1	0hsa-miR-27a-3p	-0.13	-0.14N/A
TRAF3	ENST00000560371.1	TNF receptor-associated factor 3	833	1	0	1	0	2hsa-miR-27a-3p	-0.13	-0.18N/A
CAPN7	ENST00000253693.2	calpain 7	76	1	0	1	0	1hsa-miR-27a-3p	-0.13	-0.15N/A
PHLPP2	ENST00000568954.1	PH domain and leucine rich repeat protein phosphatase 2	172	1	1	0	0	2hsa-miR-27a-3p	-0.13	-0.13N/A
LIN7A	ENST00000552864.1	lin-7 homolog A (C. elegans)	86	1	0	0	1	2hsa-miR-27a-3p	-0.13	-0.18N/A
CECR6	ENST00000399875.1	cat eye syndrome chromosome region, candidate 6	5	1	1	0	0	1hsa-miR-27a-3p	-0.13	-0.13N/A
CALM3	ENST00000291295.9	calmodulin 3 (phosphorylase kinase, delta)	3982	1	0	0	1	1hsa-miR-27a-3p	-0.13	-0.2N/A
BEND3	ENST00000369042.1	BEN domain containing 3	13	1	0	1	0	1hsa-miR-27a-3p	-0.13	-0.28N/A
CCNYL1	ENST00000295414.3	cyclin Y-like 1	111	1	0	0	1	0hsa-miR-27a-3p	-0.13	-0.13N/A
NXF1	ENST00000531709.2	nuclear RNA export factor 1	1017	1	0	1	0	0hsa-miR-27a-3p	-0.13	-0.13N/A
MAPT	ENST00000344290.5	microtubule-associated protein tau	11	1	0	0	1	2hsa-miR-27a-3p	-0.13	-0.13N/A
RASA2	ENST00000286364.3	RAS p21 protein activator 2	170	1	0	1	0	0hsa-miR-27a-3p	-0.13	-0.13N/A
MSI1	ENST00000257552.2	musashi RNA-binding protein 1	287	1	0	1	0	0hsa-miR-27a-3p	-0.13	-0.13N/A
FAIM3	ENST00000420007.2	Fas apoptotic inhibitory molecule 3	311	1	0	0	1	0hsa-miR-27a-3p	-0.13	-0.13N/A
SEPT4	ENST00000426861.1	septin 4	40	1	0	1	0	0hsa-miR-27a-3p	-0.13	-0.13N/A
ACLY	ENST00000352035.2	ATP citrate lyase	5900	1	0	0	1	0hsa-miR-27a-3p	-0.13	-0.14N/A
FAM105B	ENST00000284274.4	family with sequence similarity 105, member B	247	1	1	0	0	2hsa-miR-27a-3p	-0.13	-0.26N/A
SLC20A1	ENST00000272542.3	solute carrier family 20 (phosphate transporter), member 1	205	1	0	0	1	0hsa-miR-27a-3p	-0.13	-0.13N/A
ARID5B	ENST00000279873.7	AT rich interactive domain 5B (MRF1-like)	397	1	0	0	1	1hsa-miR-27a-3p	-0.13	-0.13N/A
CRTC1	ENST00000338797.6	CREB regulated transcription coactivator 1	322	1	0	1	0	1hsa-miR-27a-3p	-0.13	-0.13N/A
CPD	ENST00000225719.4	carboxypeptidase D	1766	1	0	0	1	2hsa-miR-27a-3p	-0.13	-0.13N/A
STOX2	ENST00000308497.4	storkhead box 2	379	1	0	1	0	1hsa-miR-27a-3p	-0.12	-0.12N/A
GOLGA6C	ENST00000300576.5	golgin A6 family, member C	0	1	0	1	0	0hsa-miR-27a-3p	-0.12	-0.12N/A
FN1	ENST00000357009.2	fibronectin 1	1064	1	0	1	0	1hsa-miR-27a-3p	-0.12	-0.17N/A
HIPK3	ENST00000303296.4	homeodomain interacting protein kinase 3	933	1	0	0	1	0hsa-miR-27a-3p	-0.12	-0.13N/A
SERTAD3	ENST00000392028.4	SERTA domain containing 3	765	1	0	0	1	1hsa-miR-27a-3p	-0.12	-0.12N/A
C15orf27	ENST00000388942.3	chromosome 15 open reading frame 27	17	1	0	1	0	0hsa-miR-27a-3p	-0.12	-0.12N/A
PIGM	ENST00000368090.2	phosphatidylinositol glycan anchor biosynthesis, class M	179	1	0	0	1	1hsa-miR-27a-3p	-0.12	-0.17N/A
CHST2	ENST00000309575.3	carbohydrate (N-acetylglucosamine-6-O) sulfotransferase 2	154	1	0	0	1	0hsa-miR-27a-3p	-0.12	-0.15N/A
SERINC3	ENST00000342374.4	serine incorporator 3	1218	1	0	0	1	0hsa-miR-27a-3p	-0.12	-0.13N/A
PPARGC1B	ENST00000309241.5	peroxisome proliferator-activated receptor gamma, coactivator 1 beta	12	1	1	0	0	4hsa-miR-27a-3p	-0.12	-0.15N/A
NEUROD4	ENST00000242994.3	neuronal differentiation 4	5	1	1	0	0	1hsa-miR-27a-3p	-0.12	-0.12N/A
MAL2	ENST00000276681.6	mal, T-cell differentiation protein 2 (gene/pseudogene)	2056	1	0	0	1	0hsa-miR-27a-3p	-0.12	-0.14N/A
MAPK14	ENST00000229795.3	mitogen-activated protein kinase 14	1191	1	0	1	0	4hsa-miR-27a-3p	-0.12	-0.24N/A
XIAP	ENST00000371199.3	X-linked inhibitor of apoptosis	153	1	0	0	1	0hsa-miR-27a-3p	-0.12	-0.2N/A
ADAM19	ENST00000257527.4	ADAM metallopeptidase domain 19	981	1	1	0	0	1hsa-miR-27a-3p	-0.12	-0.12N/A
RAB14	ENST00000373840.4	RAB14, member RAS oncogene family	586	1	0	0	1	1hsa-miR-27a-3p	-0.12	-0.17N/A
LASP1	ENST00000318008.6	LIM and SH3 protein 1	552	1	0	1	0	1hsa-miR-27a-3p	-0.12	-0.12N/A
SIPA1L3	ENST00000222345.6	signal-induced proliferation-associated 1 like 3	1578	1	0	1	0	3hsa-miR-27a-3p	-0.12	-0.12N/A
TBP	ENST00000230354.6	TATA box binding protein	1997	1	0	1	0	0hsa-miR-27a-3p	-0.12	-0.12N/A
ATP1A2	ENST00000361216.3	ATPase, Na+/K+ transporting, alpha 2 polypeptide	5	1	1	0	0	2hsa-miR-27a-3p	-0.12	-0.12N/A
MET	ENST00000397752.3	met proto-oncogene	1155	1	1	0	0	0hsa-miR-27a-3p	-0.12	-0.12N/A
NCOA1	ENST00000405141.1	nuclear receptor coactivator 1	200	1	0	1	0	0hsa-miR-27a-3p	-0.12	-0.23N/A
KLF7	ENST00000309446.6	Kruppel-like factor 7 (ubiquitous)	737	1	0	1	0	1hsa-miR-27a-3p	-0.12	-0.13N/A
ZNF516	ENST00000443185.2	zinc finger protein 516	311	1	0	0	1	1hsa-miR-27a-3p	-0.12	-0.12N/A
NBEAL1	ENST00000449802.1	neurobeachin-like 1	42	1	0	1	0	1hsa-miR-27a-3p	-0.12	-0.14N/A
MN1	ENST00000302326.4	meningioma (disrupted in balanced translocation) 1	15	1	0	1	0	0hsa-miR-27a-3p	-0.12	-0.12N/A
RIMS4	ENST00000372851.3	regulating synaptic membrane exocytosis 4	30	1	0	0	1	3hsa-miR-27a-3p	-0.12	-0.12N/A
ARL4C	ENST00000390645.2	ADP-ribosylation factor-like 4C	84	1	0	0	1	0hsa-miR-27a-3p	-0.12	-0.15N/A
C12orf49	ENST00000261318.3	chromosome 12 open reading frame 49	673	1	0	0	1	3hsa-miR-27a-3p	-0.12	-0.24N/A
DUSP18	ENST00000403268.1	dual specificity phosphatase 18	491	1	0	1	0	1hsa-miR-27a-3p	-0.12	-0.12N/A
MAP3K12	ENST00000267079.2	mitogen-activated protein kinase kinase kinase 12	61	1	0	0	1	0hsa-miR-27a-3p	-0.12	-0.14N/A
FBXO45	ENST00000311630.6	F-box protein 45	72	1	1	0	0	0hsa-miR-27a-3p	-0.12	-0.28N/A

AMMECR1L	ENST00000272647.5	AMMECR1-like	21	1	0	1	0	1hsa-miR-27a-3p	-0.12	-0.13N/A
WDR7	ENST00000254442.3	WD repeat domain 7	365	1	0	1	0	0hsa-miR-27a-3p	-0.12	-0.12N/A
FRMD6	ENST00000395718.2	FERM domain containing 6	508	1	0	0	1	2hsa-miR-27a-3p	-0.12	-0.12N/A
NR1D2	ENST00000312521.4	nuclear receptor subfamily 1, group D, member 2	154	1	1	0	0	0hsa-miR-27a-3p	-0.12	-0.27N/A
PTCHD1	ENST00000379361.4	patched domain containing 1	59	2	1	1	0	1hsa-miR-27a-3p	-0.12	-0.3N/A
HYOU1	ENST00000404233.3	hypoxia up-regulated 1	29	1	0	1	0	1hsa-miR-27a-3p	-0.12	-0.12N/A
PTGDR	ENST00000553372.1	prostaglandin D2 receptor (DP)	5	1	1	0	0	2hsa-miR-27a-3p	-0.11	-0.55N/A
LGR4	ENST00000379214.4	leucine-rich repeat containing G protein-coupled receptor 4	1387	1	0	1	0	1hsa-miR-27a-3p	-0.11	-0.12N/A
PRKAA2	ENST00000371244.4	protein kinase, AMP-activated, alpha 2 catalytic subunit	561	1	1	0	0	1hsa-miR-27a-3p	-0.11	-0.14N/A
USP42	ENST00000306177.5	ubiquitin specific peptidase 42	86	2	0	2	0	0hsa-miR-27a-3p	-0.11	-0.48N/A
SIX5	ENST00000560168.1	SIX homeobox 5	47	1	0	1	0	0hsa-miR-27a-3p	-0.11	-0.11N/A
BCORL1	ENST00000540052.1	BCL6 corepressor-like 1	209	1	0	1	0	0hsa-miR-27a-3p	-0.11	-0.14N/A
NRIP1	ENST00000400199.1	nuclear receptor interacting protein 1	342	1	0	1	0	1hsa-miR-27a-3p	-0.11	-0.11N/A
ITGA1	ENST00000282588.6	integrin, alpha 1	400	1	0	1	0	5hsa-miR-27a-3p	-0.11	-0.27N/A
SLC25A44	ENST00000359511.4	solute carrier family 25, member 44	760	1	0	0	1	0hsa-miR-27a-3p	-0.11	-0.11N/A
SP6	ENST00000342234.2	Sp6 transcription factor	5	1	0	0	1	0hsa-miR-27a-3p	-0.11	-0.11N/A
PHF7	ENST00000327906.3	PHD finger protein 7	38	1	0	1	0	1hsa-miR-27a-3p	-0.11	-0.11N/A
KMT2A	ENST00000534358.1	lysine (K)-specific methyltransferase 2A	1092	1	0	1	0	3hsa-miR-27a-3p	-0.11	-0.11N/A
MAN2A2	ENST00000360468.3	mannosidase, alpha, class 2A, member 2	340	1	0	1	0	1hsa-miR-27a-3p	-0.11	-0.17N/A
NCALD	ENST00000395923.1	neurocalcin delta	49	1	0	0	1	2hsa-miR-27a-3p	-0.11	-0.11N/A
DYRK1A	ENST00000339659.4	dual-specificity tyrosine-(Y)-phosphorylation regulated kinase 1A	448	1	0	0	1	0hsa-miR-27a-3p	-0.11	-0.16N/A
TMEM25	ENST00000411589.2	transmembrane protein 25	767	1	0	1	0	0hsa-miR-27a-3p	-0.11	-0.11N/A
MSL2	ENST00000309993.2	male-specific lethal 2 homolog (Drosophila)	175	1	0	0	1	1hsa-miR-27a-3p	-0.11	-0.12N/A
CAMK4	ENST00000282356.4	calcium/calmodulin-dependent protein kinase IV	73	2	0	1	1	2hsa-miR-27a-3p	-0.11	-0.16N/A
BNIP3L	ENST00000380629.2	BCL2/adenovirus E1B 19kDa interacting protein 3-like	191	1	0	0	1	0hsa-miR-27a-3p	-0.11	-0.11N/A
ERC2	ENST00000288221.6	ELKS/RAB6-interacting/CAST family member 2	11	1	0	1	0	1hsa-miR-27a-3p	-0.11	-0.11N/A
AMOTL2	ENST00000249883.5	angiomotin like 2	78	1	0	0	1	2hsa-miR-27a-3p	-0.11	-0.12N/A
RIOK3	ENST00000339486.3	RIO kinase 3	757	1	0	0	1	0hsa-miR-27a-3p	-0.11	-0.13N/A
TBX4	ENST00000393853.4	T-box 4	21	1	1	0	0	1hsa-miR-27a-3p	-0.11	-0.51N/A
APAF1	ENST00000333991.1	apoptotic peptidase activating factor 1	23	1	0	1	0	0hsa-miR-27a-3p	-0.11	-0.13N/A
NEO1	ENST00000261908.6	neogenin 1	261	1	0	1	0	2hsa-miR-27a-3p	-0.11	-0.28N/A
TOX	ENST00000361421.1	thymocyte selection-associated high mobility group box	139	1	0	0	1	0hsa-miR-27a-3p	-0.11	-0.11N/A
KIAA1045	ENST00000242315.3	KIAA1045	42	1	0	1	0	1hsa-miR-27a-3p	-0.11	-0.11N/A
MBNL2	ENST00000345429.6	muscleblind-like splicing regulator 2	34	1	0	1	0	1hsa-miR-27a-3p	-0.11	-0.13N/A
NEK6	ENST00000373603.1	NIMA-related kinase 6	3449	1	1	0	0	1hsa-miR-27a-3p	-0.11	-0.58N/A
AGGF1	ENST00000312916.7	angiogenic factor with G patch and FHA domains 1	890	1	0	0	1	0hsa-miR-27a-3p	-0.11	-0.11N/A
SAMD10	ENST00000369886.3	sterile alpha motif domain containing 10	45	1	0	1	0	0hsa-miR-27a-3p	-0.1	-0.1N/A
SLCO5A1	ENST00000260126.4	solute carrier organic anion transporter family, member 5A1	9	1	0	1	0	1hsa-miR-27a-3p	-0.1	-0.1N/A
SEPN1	ENST00000361547.2	selenoprotein N, 1	104	1	1	0	0	1hsa-miR-27a-3p	-0.1	-0.1N/A
TTC9	ENST00000256367.2	tetratricopeptide repeat domain 9	39	1	0	1	0	1hsa-miR-27a-3p	-0.1	-0.29N/A
CDC25B	ENST00000245960.5	cell division cycle 25B	3358	1	0	0	1	0hsa-miR-27a-3p	-0.1	-0.11N/A
ZBTB10	ENST00000430430.1	zinc finger and BTB domain containing 10	113	1	0	0	1	1hsa-miR-27a-3p	-0.1	-0.1N/A
SBK1	ENST00000341901.4	SH3 domain binding kinase 1	9	1	0	0	1	0hsa-miR-27a-3p	-0.1	-0.1N/A
DCP1A	ENST00000607628.1	decapping mRNA 1A	87	1	1	0	0	2hsa-miR-27a-3p	-0.1	-0.11N/A
ASH2L	ENST00000343823.6	ash2 (absent, small, or homeotic)-like (Drosophila)	140	1	0	0	1	1hsa-miR-27a-3p	-0.1	-0.14N/A
COBLL1	ENST00000375458.2	cordon-bleu WH2 repeat protein-like 1	70	1	0	0	1	2hsa-miR-27a-3p	-0.1	-0.12N/A
ZFP1	ENST00000464850.1	ZFP1 zinc finger protein	32	1	0	1	0	1hsa-miR-27a-3p	-0.1	-0.2N/A
PPM1H	ENST00000228705.6	protein phosphatase, Mg2+/Mn2+ dependent, 1H	45	1	0	1	0	4hsa-miR-27a-3p	-0.1	-0.38N/A
FZD4	ENST00000531380.1	frizzled family receptor 4	5	1	0	0	1	2hsa-miR-27a-3p	-0.1	-0.1N/A
SMCR8	ENST00000406438.3	Smith-Magenis syndrome chromosome region, candidate 8	219	2	0	2	0	2hsa-miR-27a-3p	-0.1	-0.1N/A
CDHR2	ENST00000510636.1	cadherin-related family member 2	84	1	0	1	0	4hsa-miR-27a-3p	-0.1	-0.27N/A
CSNK1G1	ENST00000303052.7	casein kinase 1, gamma 1	149	1	0	0	1	2hsa-miR-27a-3p	-0.1	-0.12N/A
SEPT11	ENST00000264893.6	septin 11	515	1	0	0	1	0hsa-miR-27a-3p	-0.1	-0.1N/A
DPY19L3	ENST00000586987.1	dpy-19-like 3 (C. elegans)	146	1	0	1	0	2hsa-miR-27a-3p	-0.1	-0.11N/A
ASB7	ENST00000332783.7	ankyrin repeat and SOCS box containing 7	95	1	0	0	1	1hsa-miR-27a-3p	-0.1	-0.1N/A
METTL8	ENST00000375258.4	methyltransferase like 8	124	1	0	1	0	0hsa-miR-27a-3p	-0.1	-0.13N/A
LONRF2	ENST00000393437.3	LON peptidase N-terminal domain and ring finger 2	394	1	0	0	1	3hsa-miR-27a-3p	-0.1	-0.1N/A

GPRIN3	ENST00000609438.1	GPRIN family member 3	213	1	1	0	0	5hsa-miR-27a-3p	-0.1	-0.1N/A
ATF2	ENST00000487334.2	activating transcription factor 2	123	1	0	1	0	0hsa-miR-27a-3p	-0.1	-0.16N/A
RAB11FIP1	ENST00000287263.4	RAB11 family interacting protein 1 (class I)	106	1	0	1	0	1hsa-miR-27a-3p	-0.1	-0.28N/A
NAP1L5	ENST00000323061.5	nucleosome assembly protein 1-like 5	28	1	0	0	1	0hsa-miR-27a-3p	-0.1	-0.14N/A
ATP10B	ENST00000327245.5	ATPase, class V, type 10B	5	1	0	1	0	0hsa-miR-27a-3p	-0.1	-0.1N/A
CHD7	ENST00000423902.2	chromodomain helicase DNA binding protein 7	925	1	0	0	1	1hsa-miR-27a-3p	-0.1	-0.12N/A
BCLAF1	ENST00000531224.1	BCL2-associated transcription factor 1	1732	1	0	0	1	1hsa-miR-27a-3p	-0.1	-0.13N/A
ZNF106	ENST00000263805.4	zinc finger protein 106	406	1	0	0	1	1hsa-miR-27a-3p	-0.1	-0.11N/A
ELMO1	ENST00000341056.3	engulfment and cell motility 1	118	1	0	1	0	2hsa-miR-27a-3p	-0.1	-0.1N/A
C11orf87	ENST00000327419.6	chromosome 11 open reading frame 87	5	1	1	0	0	0hsa-miR-27a-3p	-0.1	-0.1N/A
CDK6	ENST00000265734.4	cyclin-dependent kinase 6	1116	1	0	0	1	2hsa-miR-27a-3p	-0.1	-0.11N/A
TARDBP	ENST00000240185.3	TAR DNA binding protein	410	1	0	0	1	2hsa-miR-27a-3p	-0.1	-0.11N/A
RPP25	ENST00000322177.5	ribonuclease P/MRP 25kDa subunit	546	1	0	0	1	0hsa-miR-27a-3p	-0.1	-0.1N/A
NRARP	ENST00000356628.2	NOTCH-regulated ankyrin repeat protein	56	1	1	0	0	0hsa-miR-27a-3p	-0.1	-0.43N/A
MYCBP	ENST00000397572.2	MYC binding protein	85	1	0	0	1	0hsa-miR-27a-3p	-0.1	-0.15N/A
BACE1	ENST00000313005.6	beta-site APP-cleaving enzyme 1	486	1	1	0	0	1hsa-miR-27a-3p	-0.1	-0.2N/A
GOLGA6A	ENST00000290438.3	golgin A6 family, member A	5	1	0	1	0	0hsa-miR-27a-3p	-0.1	-0.16N/A
GOLGA6D	ENST00000434739.3	golgin A6 family, member D	0	1	0	1	0	0hsa-miR-27a-3p	-0.1	-0.1N/A
SPATA13	ENST00000382108.3	spermatogenesis associated 13	900	1	0	0	1	2hsa-miR-27a-3p	-0.09	-0.1N/A
IL6ST	ENST00000381287.4	interleukin 6 signal transducer (gp130, oncostatin M receptor)	213	1	0	0	1	2hsa-miR-27a-3p	-0.09	-0.11N/A
ZFP36L2	ENST00000282388.3	ZFP36 ring finger protein-like 2	317	1	0	1	0	1hsa-miR-27a-3p	-0.09	-0.16N/A
GIPC1	ENST00000393029.3	GIPC PDZ domain containing family, member 1	2934	1	0	0	1	0hsa-miR-27a-3p	-0.09	-0.09N/A
TBKBP1	ENST00000361722.3	TBK1 binding protein 1	173	1	0	0	1	0hsa-miR-27a-3p	-0.09	-0.09N/A
TMTC2	ENST00000321196.3	transmembrane and tetratricopeptide repeat containing 2	173	1	0	1	0	0hsa-miR-27a-3p	-0.09	-0.09N/A
FRYL	ENST00000503238.1	FRY-like	276	1	0	1	0	1hsa-miR-27a-3p	-0.09	-0.17N/A
RAB39B	ENST00000369454.3	RAB39B, member RAS oncogene family	10	1	0	1	0	1hsa-miR-27a-3p	-0.09	-0.27N/A
PCDH10	ENST00000264360.5	protocadherin 10	9	1	1	0	0	1hsa-miR-27a-3p	-0.09	-0.12N/A
PAK6	ENST00000260404.4	p21 protein (Cdc42/Rac)-activated kinase 6	70	1	1	0	0	1hsa-miR-27a-3p	-0.09	-0.09N/A
WISP1	ENST00000250160.6	WNT1 inducible signaling pathway protein 1	5	1	0	1	0	0hsa-miR-27a-3p	-0.09	-0.38N/A
JAZF1	ENST00000283928.5	JAZF zinc finger 1	869	1	0	0	1	0hsa-miR-27a-3p	-0.09	-0.24N/A
NF1	ENST00000358273.4	neurofibromin 1	161	1	1	0	0	0hsa-miR-27a-3p	-0.09	-0.09N/A
GOLGA8A	ENST00000360553.3	golgin A8 family, member A	2264	1	0	1	0	1hsa-miR-27a-3p	-0.09	-0.09N/A
RARA	ENST00000425707.3	retinoic acid receptor, alpha	560	2	2	0	0	0hsa-miR-27a-3p	-0.09	-0.34N/A
SEMA4F	ENST00000357877.2	sema domain, immunoglobulin domain (Ig), transmembrane domain (TM) and short cytoplasmic domain, (semaphorin) 4F	32	2	0	0	2	1hsa-miR-27a-3p	-0.09	-0.21N/A
NR2C2	ENST00000425241.1	nuclear receptor subfamily 2, group C, member 2	227	1	0	0	1	0hsa-miR-27a-3p	-0.09	-0.14N/A
PPIG	ENST00000260970.3	peptidylprolyl isomerase G (cyclophilin G)	470	1	0	1	0	1hsa-miR-27a-3p	-0.09	-0.21N/A
DMRT3	ENST00000190165.2	doublesex and mab-3 related transcription factor 3	21	1	0	0	1	0hsa-miR-27a-3p	-0.09	-0.15N/A
MYOCD	ENST00000425538.1	myocardin	62	1	0	0	1	0hsa-miR-27a-3p	-0.09	-0.11N/A
UBE2Q1	ENST00000292211.4	ubiquitin-conjugating enzyme E2Q family member 1	3046	1	0	1	0	0hsa-miR-27a-3p	-0.09	-0.14N/A
SAP30BP	ENST00000584667.1	SAP30 binding protein	867	1	0	0	1	1hsa-miR-27a-3p	-0.09	-0.13N/A
PLEKHA2	ENST00000420274.1	pleckstrin homology domain containing, family A (phosphoinositide binding specific) member 2	3303	1	0	0	1	2hsa-miR-27a-3p	-0.09	-0.09N/A
UHRF1BP1	ENST00000192788.5	UHRF1 binding protein 1	360	1	0	0	1	4hsa-miR-27a-3p	-0.09	-0.12N/A
CDYL	ENST00000343762.5	chromodomain protein, Y-like	24	1	0	0	1	0hsa-miR-27a-3p	-0.09	-0.16N/A
STXBP6	ENST00000396700.1	syntaxin binding protein 6 (amisyn)	40	1	1	0	0	1hsa-miR-27a-3p	-0.09	-0.33N/A
ECE1	ENST00000415912.2	endothelin converting enzyme 1	1166	1	0	0	1	1hsa-miR-27a-3p	-0.09	-0.09N/A
RALGPS1	ENST00000259351.5	Ral GEF with PH domain and SH3 binding motif 1	34	1	0	1	0	4hsa-miR-27a-3p	-0.09	-0.1N/A
KLF12	ENST00000377669.2	Kruppel-like factor 12	251	1	0	0	1	1hsa-miR-27a-3p	-0.09	-0.1N/A
FGD6	ENST00000343958.4	FYVE, RhoGEF and PH domain containing 6	21	1	1	0	0	2hsa-miR-27a-3p	-0.09	-0.13N/A
SMCHD1	ENST00000320876.6	structural maintenance of chromosomes flexible hinge domain containing 1	270	1	0	0	1	0hsa-miR-27a-3p	-0.09	-0.1N/A
COLGALT2	ENST00000367521.1	collagen beta(1-O)galactosyltransferase 2	70	1	1	0	0	2hsa-miR-27a-3p	-0.09	-0.34N/A
CLSTN2	ENST00000458420.3	calsyntenin 2	119	2	0	1	1	7hsa-miR-27a-3p	-0.09	-0.14N/A
FNDC3A	ENST00000492622.2	fibronectin type III domain containing 3A	122	1	0	0	1	0hsa-miR-27a-3p	-0.09	-0.13N/A
SIX1	ENST00000247182.6	SIX homeobox 1	1264	1	1	0	0	0hsa-miR-27a-3p	-0.09	-0.39N/A
CMTM4	ENST00000394106.2	CKLF-like MARVEL transmembrane domain containing 4	22	1	0	0	1	1hsa-miR-27a-3p	-0.09	-0.3N/A

SYT1	ENST00000457153.2	synaptotagmin I	13	1	0	1	0	1hsa-miR-27a-3p	-0.09	-0.09N/A
RORA	ENST00000335670.6	RAR-related orphan receptor A	102	1	1	0	0	1hsa-miR-27a-3p	-0.09	-0.1N/A
FBXO46	ENST00000317683.3	F-box protein 46	197	1	0	0	1	0hsa-miR-27a-3p	-0.08	-0.09N/A
COL5A1	ENST00000371817.3	collagen, type V, alpha 1	3111	1	1	0	0	0hsa-miR-27a-3p	-0.08	-0.18N/A
EPAS1	ENST00000263734.3	endothelial PAS domain protein 1	3689	1	0	0	1	1hsa-miR-27a-3p	-0.08	-0.08N/A
PPME1	ENST00000328257.8	protein phosphatase methylesterase 1	343	1	0	1	0	1hsa-miR-27a-3p	-0.08	-0.08N/A
EGR3	ENST00000317216.2	early growth response 3	21	1	0	1	0	0hsa-miR-27a-3p	-0.08	-0.08N/A
PRRC2C	ENST00000367742.3	proline-rich coiled-coil 2C	5	1	0	0	1	1hsa-miR-27a-3p	-0.08	-0.08N/A
RLIM	ENST00000332687.6	ring finger protein, LIM domain interacting	662	1	0	1	0	0hsa-miR-27a-3p	-0.08	-0.1N/A
URGCP	ENST00000223341.7	upregulator of cell proliferation	180	1	0	1	0	0hsa-miR-27a-3p	-0.08	-0.09N/A
FAM126B	ENST00000418596.3	family with sequence similarity 126, member B	132	1	0	1	0	0hsa-miR-27a-3p	-0.08	-0.08N/A
MSI2	ENST00000284073.2	musashi RNA-binding protein 2	283	1	0	1	0	1hsa-miR-27a-3p	-0.08	-0.12N/A
CAPRIN2	ENST00000395805.2	caprin family member 2	17	1	0	0	1	0hsa-miR-27a-3p	-0.08	-0.08N/A
C6orf223	ENST00000439969.2	chromosome 6 open reading frame 223	17	1	0	1	0	4hsa-miR-27a-3p	-0.08	-0.12N/A
ZNF618	ENST00000288466.7	zinc finger protein 618	778	1	0	0	1	3hsa-miR-27a-3p	-0.08	-0.08N/A
CNNM2	ENST00000369878.4	cyclin M2	517	2	1	1	0	7hsa-miR-27a-3p	-0.08	-0.28N/A
PPP1R12A	ENST00000261207.5	protein phosphatase 1, regulatory subunit 12A	276	1	0	0	1	1hsa-miR-27a-3p	-0.08	-0.09N/A
PRKG1	ENST00000373985.1	protein kinase, cGMP-dependent, type I	12	1	0	0	1	0hsa-miR-27a-3p	-0.08	-0.08N/A
SRRM2	ENST00000301740.8	serine/arginine repetitive matrix 2	5111	1	0	0	1	0hsa-miR-27a-3p	-0.08	-0.08N/A
LANCL3	ENST00000378621.3	LanC lantibiotic synthetase component C-like 3 (bacterial)	15	1	0	1	0	4hsa-miR-27a-3p	-0.08	-0.25N/A
KIAA1551	ENST00000312561.4	KIAA1551	15	1	0	1	0	2hsa-miR-27a-3p	-0.08	-0.18N/A
USP31	ENST00000219689.7	ubiquitin specific peptidase 31	214	1	0	0	1	1hsa-miR-27a-3p	-0.08	-0.15N/A
PPP6R3	ENST00000393800.2	protein phosphatase 6, regulatory subunit 3	400	1	0	0	1	0hsa-miR-27a-3p	-0.08	-0.08N/A
SLC1A2	ENST00000278379.3	solute carrier family 1 (glial high affinity glutamate transporter), member 2	5	1	1	0	0	2hsa-miR-27a-3p	-0.08	-0.08N/A
CCDC85C	ENST00000380243.4	coiled-coil domain containing 85C	86	1	0	1	0	3hsa-miR-27a-3p	-0.08	-0.09N/A
SV2A	ENST00000369146.3	synaptic vesicle glycoprotein 2A	10	1	0	1	0	1hsa-miR-27a-3p	-0.08	-0.08N/A
ABAT	ENST00000268251.8	4-aminobutyrate aminotransferase	66	1	1	0	0	1hsa-miR-27a-3p	-0.08	-0.13N/A
ATAD2B	ENST00000238789.5	ATPase family, AAA domain containing 2B	11	1	0	0	1	1hsa-miR-27a-3p	-0.08	-0.12N/A
N4BP1	ENST00000262384.3	NEDD4 binding protein 1	105	1	0	1	0	1hsa-miR-27a-3p	-0.08	-0.1N/A
MAPKAPK3	ENST00000446044.1	mitogen-activated protein kinase-activated protein kinase 3	155	1	0	1	0	0hsa-miR-27a-3p	-0.08	-0.19N/A
BMPR1A	ENST00000372037.3	bone morphogenetic protein receptor, type IA	125	1	0	0	1	1hsa-miR-27a-3p	-0.08	-0.08N/A
CERS6	ENST00000305747.6	ceramide synthase 6	1932	1	0	0	1	1hsa-miR-27a-3p	-0.08	-0.08N/A
NTN1	ENST00000173229.2	netrin 1	12	1	1	0	0	1hsa-miR-27a-3p	-0.07	-0.07N/A
SPTLC2	ENST00000216484.2	serine palmitoyltransferase, long chain base subunit 2	95	2	1	1	0	3hsa-miR-27a-3p	-0.07	-0.3N/A
NRAS	ENST00000369535.4	neuroblastoma RAS viral (v-ras) oncogene homolog	551	1	0	0	1	3hsa-miR-27a-3p	-0.07	-0.2N/A
PEAK1	ENST00000312493.4	pseudopodium-enriched atypical kinase 1	209	1	0	1	0	2hsa-miR-27a-3p	-0.07	-0.09N/A
SFRP1	ENST00000220772.3	secreted frizzled-related protein 1	148	1	1	0	0	1hsa-miR-27a-3p	-0.07	-0.45N/A
ANO6	ENST00000320560.8	anoctamin 6	2262	1	0	0	1	0hsa-miR-27a-3p	-0.07	-0.07N/A
ABL2	ENST00000502732.1	c-abl oncogene 2, non-receptor tyrosine kinase	892	1	1	0	0	1hsa-miR-27a-3p	-0.07	-0.08N/A
SLC27A4	ENST00000300456.4	solute carrier family 27 (fatty acid transporter), member 4	523	1	0	0	1	2hsa-miR-27a-3p	-0.07	-0.1N/A
JMJD1C	ENST00000399251.1	jumonji domain containing 1C	60	1	0	1	0	2hsa-miR-27a-3p	-0.07	-0.09N/A
LAYN	ENST00000525126.1	layilin	175	1	0	0	1	0hsa-miR-27a-3p	-0.07	-0.19N/A
SLC24A2	ENST00000341998.2	solute carrier family 24 (sodium/potassium/calcium exchanger), member 2	5	1	0	0	1	3hsa-miR-27a-3p	-0.07	-0.07N/A
CTH	ENST00000411986.2	cystathionase (cystathionine gamma-lyase)	430	1	1	0	0	1hsa-miR-27a-3p	-0.07	-0.35N/A
SOS1	ENST00000426016.1	son of sevenless homolog 1 (Drosophila)	189	3	0	3	0	0hsa-miR-27a-3p	-0.07	-0.17N/A
DVL2	ENST00000005340.5	dishevelled segment polarity protein 2	864	1	0	1	0	0hsa-miR-27a-3p	-0.07	-0.07N/A
HECW2	ENST00000260983.3	HECT, C2 and WW domain containing E3 ubiquitin protein ligase 2	28	1	1	0	0	2hsa-miR-27a-3p	-0.07	-0.1N/A
SGPL1	ENST00000373202.3	sphingosine-1-phosphate lyase 1	105	1	0	1	0	1hsa-miR-27a-3p	-0.07	-0.09N/A
GFPT1	ENST00000357308.4	glutamine--fructose-6-phosphate transaminase 1	1313	1	1	0	0	3hsa-miR-27a-3p	-0.07	-0.48N/A
CCNT1	ENST00000261900.3	cyclin T1	86	1	0	0	1	0hsa-miR-27a-3p	-0.07	-0.08N/A
SLC5A3	ENST00000381151.3	solute carrier family 5 (sodium/myo-inositol cotransporter), member 3	24	2	0	1	1	1hsa-miR-27a-3p	-0.07	-0.32N/A
SLC5A3	ENST00000608209.1	sodium/myo-inositol cotransporter	24	2	0	1	1	1hsa-miR-27a-3p	-0.07	-0.32N/A
NKTR	ENST00000232978.8	natural killer-tumor recognition sequence	798	1	1	0	0	0hsa-miR-27a-3p	-0.07	-0.07N/A
NCAM1	ENST00000316851.7	neural cell adhesion molecule 1	19	1	1	0	0	0hsa-miR-27a-3p	-0.07	-0.23N/A
KIAA2018	ENST00000316407.4	KIAA2018	23	1	1	0	0	2hsa-miR-27a-3p	-0.07	-0.07N/A
TAB2	ENST00000367456.1	TGF-beta activated kinase 1/MAP3K7 binding protein 2	1129	1	0	0	1	0hsa-miR-27a-3p	-0.07	-0.07N/A
SEPT8	ENST00000378706.1	septin 8	199	1	1	0	0	1hsa-miR-27a-3p	-0.07	-0.16N/A

NUP43	ENST00000367403.3	nucleoporin 43kDa	113	1	0	0	1	2hsa-miR-27a-3p	-0.07	-0.08N/A
SHH	ENST00000297261.2	sonic hedgehog	32	1	1	0	0	0hsa-miR-27a-3p	-0.07	-0.25N/A
BHLHE41	ENST00000242728.4	basic helix-loop-helix family, member e41	50	1	0	1	0	4hsa-miR-27a-3p	-0.07	-0.09N/A
MYCBP2	ENST00000544440.2	MYC binding protein 2, E3 ubiquitin protein ligase	343	1	0	0	1	0hsa-miR-27a-3p	-0.07	-0.07N/A
CNTNAP2	ENST00000361727.3	contactin associated protein-like 2	29	1	0	0	1	2hsa-miR-27a-3p	-0.07	-0.08N/A
MSN	ENST00000360270.5	moesin	5947	1	0	0	1	0hsa-miR-27a-3p	-0.07	-0.07N/A
DUSP16	ENST00000298573.4	dual specificity phosphatase 16	444	1	0	0	1	0hsa-miR-27a-3p	-0.07	-0.1N/A
IKZF1	ENST00000331340.3	IKAROS family zinc finger 1 (Ikaros)	11	2	1	0	1	1hsa-miR-27a-3p	-0.07	-0.15N/A
RAB11FIP2	ENST00000355624.3	RAB11 family interacting protein 2 (class I)	449	1	0	0	1	0hsa-miR-27a-3p	-0.07	-0.07N/A
MARCH6	ENST00000274140.5	membrane-associated ring finger (C3HC4) 6, E3 ubiquitin protein ligase	14086	1	0	1	0	1hsa-miR-27a-3p	-0.07	-0.23N/A
ADD1	ENST00000398125.1	adducin 1 (alpha)	460	1	1	0	0	0hsa-miR-27a-3p	-0.07	-0.07N/A
ABCA12	ENST00000272895.7	ATP-binding cassette, sub-family A (ABC1), member 12	5	1	0	1	0	1hsa-miR-27a-3p	-0.07	-0.07N/A
NRCAM	ENST00000379028.3	neuronal cell adhesion molecule	152	1	0	0	1	0hsa-miR-27a-3p	-0.07	-0.14N/A
FAM81A	ENST00000288228.5	family with sequence similarity 81, member A	53	1	0	1	0	0hsa-miR-27a-3p	-0.07	-0.1N/A
UBE2K	ENST00000261427.5	ubiquitin-conjugating enzyme E2K	762	1	0	0	1	0hsa-miR-27a-3p	-0.07	-0.08N/A
ATPAF1	ENST00000576409.1	ATP synthase mitochondrial F1 complex assembly factor 1	1637	1	0	1	0	0hsa-miR-27a-3p	-0.07	-0.07N/A
SOCS4	ENST00000395472.2	suppressor of cytokine signaling 4	336	1	0	0	1	0hsa-miR-27a-3p	-0.07	-0.09N/A
FBXO48	ENST00000377957.3	F-box protein 48	13	1	1	0	0	0hsa-miR-27a-3p	-0.07	-0.17N/A
BRSK2	ENST00000382179.1	BR serine/threonine kinase 2	15	1	0	1	0	1hsa-miR-27a-3p	-0.07	-0.17N/A
ADAMTSL1	ENST00000380548.4	ADAMTS-like 1	38	1	0	0	1	0hsa-miR-27a-3p	-0.07	-0.1N/A
ST3GAL2	ENST00000342907.2	ST3 beta-galactoside alpha-2,3-sialyltransferase 2	102	1	0	0	1	1hsa-miR-27a-3p	-0.06	-0.11N/A
UNC80	ENST00000439458.1	unc-80 homolog (C. elegans)	5	1	0	1	0	1hsa-miR-27a-3p	-0.06	-0.06N/A
KIRREL	ENST00000368172.1	kin of IRRE like (Drosophila)	493	1	0	1	0	0hsa-miR-27a-3p	-0.06	-0.07N/A
SETD5	ENST00000402198.1	SET domain containing 5	48	1	0	1	0	0hsa-miR-27a-3p	-0.06	-0.11N/A
FBXO32	ENST00000517956.1	F-box protein 32	500	1	0	0	1	0hsa-miR-27a-3p	-0.06	-0.14N/A
PPM1E	ENST00000308249.2	protein phosphatase, Mg2+/Mn2+ dependent, 1E	178	1	0	1	0	2hsa-miR-27a-3p	-0.06	-0.09N/A
BMP3	ENST00000282701.2	bone morphogenetic protein 3	36	1	1	0	0	1hsa-miR-27a-3p	-0.06	-0.15N/A
ATRX	ENST00000373344.5	alpha thalassemia/mental retardation syndrome X-linked	77	1	0	0	1	1hsa-miR-27a-3p	-0.06	-0.09N/A
ABHD13	ENST00000375898.3	abhydrolase domain containing 13	479	1	0	1	0	0hsa-miR-27a-3p	-0.06	-0.22N/A
PDP2	ENST00000311765.2	pyruvate dehyrogenase phosphatase catalytic subunit 2	102	1	1	0	0	4hsa-miR-27a-3p	-0.06	-0.15N/A
RNF111	ENST00000348370.4	ring finger protein 111	395	2	0	1	1	0hsa-miR-27a-3p	-0.06	-0.07N/A
KDM4B	ENST00000159111.4	lysine (K)-specific demethylase 4B	547	1	0	0	1	0hsa-miR-27a-3p	-0.06	-0.06N/A
SLC30A7	ENST00000370112.4	solute carrier family 30 (zinc transporter), member 7	204	1	1	0	0	2hsa-miR-27a-3p	-0.06	-0.34N/A
CLCN5	ENST00000376088.3	chloride channel, voltage-sensitive 5	154	1	1	0	0	2hsa-miR-27a-3p	-0.06	-0.12N/A
LDLRAD4	ENST00000399848.3	low density lipoprotein receptor class A domain containing 4	314	1	0	0	1	1hsa-miR-27a-3p	-0.06	-0.15N/A
MIDN	ENST00000591446.2	midnolin	621	1	0	0	1	0hsa-miR-27a-3p	-0.06	-0.06N/A
FOXO3	ENST00000343882.6	forkhead box O3	32	1	1	0	0	1hsa-miR-27a-3p	-0.06	-0.06N/A
ANK1	ENST00000289734.7	ankyrin 1, erythrocytic	5	1	0	1	0	1hsa-miR-27a-3p	-0.06	-0.06N/A
FAM126A	ENST00000409923.1	family with sequence similarity 126, member A	241	1	1	0	0	0hsa-miR-27a-3p	-0.06	-0.21N/A
RAB4A	ENST00000366690.4	RAB4A, member RAS oncogene family	850	1	0	1	0	1hsa-miR-27a-3p	-0.06	-0.46N/A
KDELC2	ENST00000323468.5	KDEL (Lys-Asp-Glu-Leu) containing 2	667	1	0	0	1	0hsa-miR-27a-3p	-0.06	-0.06N/A
CEP85L	ENST00000368491.3	centrosomal protein 85kDa-like	14	1	0	0	1	0hsa-miR-27a-3p	-0.06	-0.06N/A
STX6	ENST00000258301.5	syntaxin 6	348	1	0	0	1	0hsa-miR-27a-3p	-0.06	-0.12N/A
PCYOX1	ENST00000264441.5	prenylcysteine oxidase 1	407	1	0	0	1	0hsa-miR-27a-3p	-0.06	-0.06N/A
HS2ST1	ENST00000370550.5	heparan sulfate 2-O-sulfotransferase 1	787	2	0	0	2	0hsa-miR-27a-3p	-0.06	-0.06N/A
UBN2	ENST00000473989.3	ubinuclein 2	238	1	1	0	0	1hsa-miR-27a-3p	-0.06	-0.06N/A
PRDM16	ENST00000270722.5	PR domain containing 16	64	1	0	1	0	0hsa-miR-27a-3p	-0.06	-0.11N/A
ADAMTSL3	ENST00000286744.5	ADAMTS-like 3	9	1	0	0	1	1hsa-miR-27a-3p	-0.06	-0.06N/A
EIF2S2	ENST00000374980.2	eukaryotic translation initiation factor 2, subunit 2 beta, 38kDa	575	1	0	1	0	1hsa-miR-27a-3p	-0.06	-0.24N/A
DAAM1	ENST00000395125.1	dishevelled associated activator of morphogenesis 1	167	1	0	0	1	1hsa-miR-27a-3p	-0.06	-0.07N/A
TGIF2	ENST00000373874.2	TGFB-induced factor homeobox 2	192	1	0	1	0	1hsa-miR-27a-3p	-0.06	-0.06N/A
TFAP2B	ENST00000393655.3	transcription factor AP-2 beta (activating enhancer binding protein 2 beta)	23	2	0	0	2	0hsa-miR-27a-3p	-0.05	-0.27N/A
SERTAD2	ENST00000313349.3	SERTA domain containing 2	119	1	0	1	0	0hsa-miR-27a-3p	-0.05	-0.07N/A
KIAA1549	ENST00000440172.1	KIAA1549	508	1	1	0	0	1hsa-miR-27a-3p	-0.05	-0.06N/A
SLC9A7	ENST00000328306.4	solute carrier family 9, subfamily A (NHE7, cation proton antiporter 7), member 7	147	1	1	0	0	1hsa-miR-27a-3p	-0.05	-0.27N/A
KIF21B	ENST00000332129.2	kinesin family member 21B	12	1	0	0	1	1hsa-miR-27a-3p	-0.05	-0.05N/A

TOR1AIP1	ENST00000606911.2	torsin A interacting protein 1	3069	1	1	0	0	2hsa-miR-27a-3p	-0.05	-0.17N/A
ITGA5	ENST00000293379.4	integrin, alpha 5 (fibronectin receptor, alpha polypeptide)	5599	1	1	0	0	0hsa-miR-27a-3p	-0.05	-0.05N/A
KCNJ3	ENST00000295101.2	potassium inwardly-rectifying channel, subfamily J, member 3	21	1	1	0	0	0hsa-miR-27a-3p	-0.05	-0.05N/A
WDFY2	ENST00000298125.5	WD repeat and FYVE domain containing 2	85	1	1	0	0	4hsa-miR-27a-3p	-0.05	-0.4N/A
LIFR	ENST00000263409.4	leukemia inhibitory factor receptor alpha	44	1	1	0	0	1hsa-miR-27a-3p	-0.05	-0.19N/A
SEMA6D	ENST00000355997.3	sema domain, transmembrane domain (TM), and cytoplasmic domain, (semaphorin) 6D	179	1	1	0	0	0hsa-miR-27a-3p	-0.05	-0.06N/A
TRERF1	ENST00000541110.1	transcriptional regulating factor 1	52	1	1	0	0	2hsa-miR-27a-3p	-0.05	-0.05N/A
CDON	ENST00000392693.3	cell adhesion associated, oncogene regulated	173	1	0	1	0	3hsa-miR-27a-3p	-0.05	-0.14N/A
RASSF5	ENST00000367117.3	Ras association (RalGDS/AF-6) domain family member 5	27	1	0	0	1	0hsa-miR-27a-3p	-0.05	-0.06N/A
PRICKLE2	ENST00000295902.6	prickle homolog 2 (Drosophila)	20	1	0	1	0	3hsa-miR-27a-3p	-0.05	-0.05N/A
ATL3	ENST00000398868.3	atlastin GTPase 3	734	1	0	1	0	0hsa-miR-27a-3p	-0.05	-0.17N/A
SHROOM2	ENST00000380913.3	shroom family member 2	121	1	1	0	0	0hsa-miR-27a-3p	-0.05	-0.05N/A
C1orf21	ENST00000235307.6	chromosome 1 open reading frame 21	935	1	1	0	0	3hsa-miR-27a-3p	-0.05	-0.17N/A
NPTXR	ENST00000333039.2	neuronal pentraxin receptor	578	1	0	1	0	1hsa-miR-27a-3p	-0.05	-0.05N/A
RAD54L2	ENST00000409535.2	RAD54-like 2 (S. cerevisiae)	215	1	0	1	0	1hsa-miR-27a-3p	-0.05	-0.05N/A
PTGFR	ENST00000370756.3	prostaglandin F receptor (FP)	20	1	0	0	1	1hsa-miR-27a-3p	-0.05	-0.05N/A
RFX3	ENST00000382004.3	regulatory factor X, 3 (influences HLA class II expression)	37	1	0	0	1	0hsa-miR-27a-3p	-0.05	-0.07N/A
VCAN	ENST00000265077.3	versican	542	2	0	1	1	0hsa-miR-27a-3p	-0.05	-0.07N/A
ZFHX3	ENST00000268489.5	zinc finger homeobox 3	57	2	2	0	0	1hsa-miR-27a-3p	-0.05	-0.06N/A
CELF1	ENST00000395290.2	CUGBP, Elav-like family member 1	153	2	0	0	2	1hsa-miR-27a-3p	-0.05	-0.06N/A
NSD1	ENST00000439151.2	nuclear receptor binding SET domain protein 1	685	1	1	0	0	1hsa-miR-27a-3p	-0.05	-0.17N/A
ARMC8	ENST00000469044.1	armadillo repeat containing 8	398	1	1	0	0	0hsa-miR-27a-3p	-0.05	-0.32N/A
CHD2	ENST00000394196.4	chromodomain helicase DNA binding protein 2	1237	1	0	1	0	2hsa-miR-27a-3p	-0.05	-0.05N/A
SUB1	ENST00000265073.4	SUB1 homolog (S. cerevisiae)	242	1	0	0	1	1hsa-miR-27a-3p	-0.05	-0.23N/A
MBOAT2	ENST00000305997.3	membrane bound O-acyltransferase domain containing 2	1145	1	0	1	0	1hsa-miR-27a-3p	-0.05	-0.15N/A
HIVEP2	ENST00000367604.1	human immunodeficiency virus type I enhancer binding protein 2	137	1	0	0	1	0hsa-miR-27a-3p	-0.05	-0.15N/A
C11orf84	ENST00000294244.4	chromosome 11 open reading frame 84	232	1	0	1	0	1hsa-miR-27a-3p	-0.04	-0.19N/A
MED12L	ENST00000474524.1	mediator complex subunit 12-like	30	2	2	0	0	1hsa-miR-27a-3p	-0.04	-0.12N/A
TXLNG	ENST00000380122.5	taxilin gamma	96	1	1	0	0	0hsa-miR-27a-3p	-0.04	-0.41N/A
GTF2IRD2	ENST00000453619.2	GTF2I repeat domain containing 2	5	1	0	0	1	0hsa-miR-27a-3p	-0.04	-0.04N/A
SETD7	ENST00000274031.3	SET domain containing (lysine methyltransferase) 7	76	1	0	1	0	5hsa-miR-27a-3p	-0.04	-0.06N/A
PGM2L1	ENST00000298198.4	phosphoglucomutase 2-like 1	183	1	1	0	0	1hsa-miR-27a-3p	-0.04	-0.09N/A
REPS2	ENST00000357277.3	RALBP1 associated Eps domain containing 2	36	1	0	0	1	3hsa-miR-27a-3p	-0.04	-0.14N/A
ZBTB8B	ENST00000609129.1	zinc finger and BTB domain containing 8B	18	1	0	1	0	2hsa-miR-27a-3p	-0.04	-0.07N/A
ZNF148	ENST00000360647.4	zinc finger protein 148	230	1	1	0	0	0hsa-miR-27a-3p	-0.04	-0.06N/A
LZTS3	ENST00000329152.3	Homo sapiens leucine zipper, putative tumor suppressor family member 3 (LZTS3), transcript variant 2, mRNA.	78	1	0	1	0	1hsa-miR-27a-3p	-0.04	-0.04N/A
PKNOX2	ENST00000298282.9	PBX/knotted 1 homeobox 2	46	1	0	0	1	0hsa-miR-27a-3p	-0.04	-0.04N/A
GNAQ	ENST00000286548.4	guanine nucleotide binding protein (G protein), q polypeptide	47	1	1	0	0	0hsa-miR-27a-3p	-0.04	-0.04N/A
RNF152	ENST00000312828.3	ring finger protein 152	57	1	1	0	0	5hsa-miR-27a-3p	-0.04	-0.09N/A
KAT2B	ENST00000263754.4	K(lysine) acetyltransferase 2B	117	1	0	0	1	0hsa-miR-27a-3p	-0.04	-0.04N/A
SPTY2D1	ENST00000336349.5	SPT2, Suppressor of Ty, domain containing 1 (S. cerevisiae)	241	1	1	0	0	2hsa-miR-27a-3p	-0.04	-0.14N/A
MAGI2	ENST00000419488.1	membrane associated guanylate kinase, WW and PDZ domain containing 2	43	2	0	0	2	1hsa-miR-27a-3p	-0.04	-0.05N/A
PDLIM4	ENST00000379018.3	PDZ and LIM domain 4	417	1	0	1	0	0hsa-miR-27a-3p	-0.04	-0.09N/A
WDTC1	ENST00000319394.3	WD and tetratricopeptide repeats 1	137	1	0	1	0	1hsa-miR-27a-3p	-0.04	-0.04N/A
SORBS1	ENST00000371227.4	sorbin and SH3 domain containing 1	22	1	0	1	0	1hsa-miR-27a-3p	-0.04	-0.04N/A
GJA9	ENST00000454994.2	gap junction protein, alpha 9, 59kDa	85	1	0	0	1	0hsa-miR-27a-3p	-0.04	-0.07N/A
CECR2	ENST00000400573.5	cat eye syndrome chromosome region, candidate 2	46	1	1	0	0	2hsa-miR-27a-3p	-0.04	-0.07N/A
KCTD16	ENST00000507359.3	potassium channel tetramerization domain containing 16	36	2	0	1	1	0hsa-miR-27a-3p	-0.04	-0.24N/A
MUC19	ENST00000454784.4	mucin 19, oligomeric	5	2	1	0	1	0hsa-miR-27a-3p	-0.04	-0.04N/A
ANKS1A	ENST00000360359.3	ankyrin repeat and sterile alpha motif domain containing 1A	220	1	1	0	0	1hsa-miR-27a-3p	-0.04	-0.04N/A
ATP11C	ENST00000327569.3	ATPase, class VI, type 11C	590	1	1	0	0	0hsa-miR-27a-3p	-0.04	-0.04N/A
AP5M1	ENST00000261558.3	adaptor-related protein complex 5, mu 1 subunit	198	1	0	1	0	3hsa-miR-27a-3p	-0.04	-0.07N/A
PTPRT	ENST00000373187.1	protein tyrosine phosphatase, receptor type, T	5	1	1	0	0	4hsa-miR-27a-3p	-0.04	-0.04N/A
NEBL	ENST00000377122.4	nebulette	181	1	1	0	0	3hsa-miR-27a-3p	-0.04	-0.14N/A
TMED5	ENST00000479918.1	transmembrane emp24 protein transport domain containing 5	612	1	0	1	0	0hsa-miR-27a-3p	-0.04	-0.31N/A

MARCH5	ENST00000358935.2	membrane-associated ring finger (C3HC4) 5	881	1	1	0	0	0hsa-miR-27a-3p	-0.04	-0.4N/A
OPA1	ENST00000392438.3	optic atrophy 1 (autosomal dominant)	962	1	1	0	0	0hsa-miR-27a-3p	-0.04	-0.12N/A
ANKFY1	ENST00000341657.4	ankyrin repeat and FYVE domain containing 1	969	1	0	1	0	2hsa-miR-27a-3p	-0.04	-0.06N/A
FASN	ENST00000306749.2	fatty acid synthase	37	1	0	0	1	0hsa-miR-27a-3p	-0.04	-0.04N/A
FUT9	ENST00000302103.5	fucosyltransferase 9 (alpha (1,3) fucosyltransferase)	5	1	1	0	0	2hsa-miR-27a-3p	-0.04	-0.04N/A
USP49	ENST00000394253.3	ubiquitin specific peptidase 49	44	1	0	1	0	0hsa-miR-27a-3p	-0.04	-0.16N/A
RXRA	ENST00000481739.1	retinoid X receptor, alpha	609	1	1	0	0	3hsa-miR-27a-3p	-0.04	-0.12N/A
PGAP1	ENST00000354764.4	post-GPI attachment to proteins 1	106	1	0	1	0	1hsa-miR-27a-3p	-0.04	-0.14N/A
NCOA5	ENST00000290231.6	nuclear receptor coactivator 5	231	1	1	0	0	0hsa-miR-27a-3p	-0.04	-0.12N/A
GOLGA6L10	ENST00000439287.4	Putative golgin subfamily A member 6-like protein 10	0	1	0	1	0	1hsa-miR-27a-3p	-0.04	-0.04N/A
GOLGA6L4	ENST00000510439.2	golgin A6 family-like 4	5	1	0	1	0	1hsa-miR-27a-3p	-0.04	-0.04N/A
ABHD10	ENST00000494817.1	abhydrolase domain containing 10	929	1	0	0	1	1hsa-miR-27a-3p	-0.04	-0.04N/A
ERMP1	ENST00000381506.3	endoplasmic reticulum metallopeptidase 1	53	1	0	1	0	1hsa-miR-27a-3p	-0.03	-0.04N/A
KLF8	ENST00000468660.1	Kruppel-like factor 8	8	1	0	0	1	1hsa-miR-27a-3p	-0.03	-0.09N/A
DTX4	ENST00000227451.3	deltex homolog 4 (Drosophila)	5	1	0	1	0	2hsa-miR-27a-3p	-0.03	-0.03N/A
THAP2	ENST00000308086.2	THAP domain containing, apoptosis associated protein 2	109	1	0	1	0	3hsa-miR-27a-3p	-0.03	-0.18N/A
ARID1B	ENST00000346085.5	AT rich interactive domain 1B (SWI1-like)	220	1	0	1	0	1hsa-miR-27a-3p	-0.03	-0.13N/A
TMEM33	ENST00000504986.1	transmembrane protein 33	1470	1	0	0	1	0hsa-miR-27a-3p	-0.03	-0.07N/A
RP13-996F3.5	ENST00000559949.1	Uncharacterized protein	0	1	0	1	0	1hsa-miR-27a-3p	-0.03	-0.03N/A
FAM192A	ENST00000309137.8	family with sequence similarity 192, member A	502	1	0	1	0	1hsa-miR-27a-3p	-0.03	-0.18N/A
FUBP1	ENST00000370767.1	far upstream element (FUSE) binding protein 1	2021	1	0	1	0	1hsa-miR-27a-3p	-0.03	-0.09N/A
NLK	ENST00000407008.3	nemo-like kinase	61	2	0	0	2	0hsa-miR-27a-3p	-0.03	-0.27N/A
ZADH2	ENST00000322342.3	zinc binding alcohol dehydrogenase domain containing 2	207	1	0	1	0	0hsa-miR-27a-3p	-0.03	-0.06N/A
IGF1	ENST00000337514.6	insulin-like growth factor 1 (somatomedin C)	19	1	0	0	1	0hsa-miR-27a-3p	-0.03	-0.42N/A
C22orf29	ENST00000407472.1	chromosome 22 open reading frame 29	351	1	0	1	0	6hsa-miR-27a-3p	-0.03	-0.04N/A
CDS2	ENST00000460006.1	CDP-diacylglycerol synthase (phosphatidate cytidylyltransferase) 2	782	1	0	1	0	4hsa-miR-27a-3p	-0.03	-0.37N/A
C14orf93	ENST00000299088.6	chromosome 14 open reading frame 93	212	1	1	0	0	1hsa-miR-27a-3p	-0.03	-0.3N/A
NCOA3	ENST00000341724.6	nuclear receptor coactivator 3	282	1	0	0	1	2hsa-miR-27a-3p	-0.03	-0.04N/A
AFF3	ENST00000409236.2	AF4/FMR2 family, member 3	296	1	0	1	0	1hsa-miR-27a-3p	-0.03	-0.04N/A
TRIM71	ENST00000383763.5	tripartite motif containing 71, E3 ubiquitin protein ligase	67	1	0	0	1	0hsa-miR-27a-3p	-0.03	-0.04N/A
COL27A1	ENST00000356083.3	collagen, type XXVII, alpha 1	620	1	1	0	0	0hsa-miR-27a-3p	-0.03	-0.03N/A
NFASC	ENST00000401399.1	neurofascin	10	1	1	0	0	5hsa-miR-27a-3p	-0.03	-0.03N/A
COL11A2	ENST00000374708.4	collagen, type XI, alpha 2	19	1	1	0	0	0hsa-miR-27a-3p	-0.03	-0.03N/A
CDC42BPB	ENST00000361246.2	CDC42 binding protein kinase beta (DMPK-like)	615	1	1	0	0	1hsa-miR-27a-3p	-0.03	-0.03N/A
ST8SIA4	ENST00000231461.5	ST8 alpha-N-acetyl-neuraminide alpha-2,8-sialyltransferase 4	5	1	1	0	0	0hsa-miR-27a-3p	-0.03	-0.03N/A
BZW1	ENST00000409600.1	basic leucine zipper and W2 domains 1	470	1	1	0	0	1hsa-miR-27a-3p	-0.03	-0.36N/A
MDGA1	ENST00000297153.7	MAM domain containing glycosylphosphatidylinositol anchor 1	240	1	0	1	0	5hsa-miR-27a-3p	-0.03	-0.03N/A
DIDO1	ENST00000266070.4	death inducer-obliterator 1	1279	1	1	0	0	0hsa-miR-27a-3p	-0.03	-0.03N/A
APBA2	ENST00000558402.1	amyloid beta (A4) precursor protein-binding, family A, member 2	388	1	0	1	0	0hsa-miR-27a-3p	-0.03	-0.19N/A
OSBPL11	ENST00000296220.5	oxysterol binding protein-like 11	101	1	0	0	1	0hsa-miR-27a-3p	-0.03	-0.08N/A
SLC27A1	ENST00000252595.7	solute carrier family 27 (fatty acid transporter), member 1	33	1	0	1	0	0hsa-miR-27a-3p	-0.03	-0.03N/A
CDR2L	ENST00000337231.5	cerebellar degeneration-related protein 2-like	28	1	0	1	0	0hsa-miR-27a-3p	-0.03	-0.03N/A
PHF15	ENST00000395003.1	PHD finger protein 15	387	1	0	1	0	0hsa-miR-27a-3p	-0.03	-0.04N/A
TIMM10	ENST00000257245.4	translocase of inner mitochondrial membrane 10 homolog (yeast)	2518	1	0	0	1	0hsa-miR-27a-3p	-0.03	-0.41N/A
PTPN9	ENST00000306726.2	protein tyrosine phosphatase, non-receptor type 9	644	1	0	1	0	1hsa-miR-27a-3p	-0.03	-0.18N/A
FBRSL1	ENST00000434748.2	fibrosin-like 1	778	1	0	1	0	0hsa-miR-27a-3p	-0.03	-0.03N/A
CASD1	ENST00000297273.4	CAS1 domain containing 1	270	1	0	1	0	1hsa-miR-27a-3p	-0.03	-0.15N/A
SNX11	ENST00000393405.2	sorting nexin 11	20	1	0	0	1	1hsa-miR-27a-3p	-0.03	-0.09N/A
ASIC1	ENST00000228468.4	acid-sensing (proton-gated) ion channel 1	111	1	0	0	1	1hsa-miR-27a-3p	-0.03	-0.05N/A
ARPP19	ENST00000566423.1	cAMP-regulated phosphoprotein, 19kDa	1316	1	0	0	1	0hsa-miR-27a-3p	-0.03	-0.08N/A
SUZ12	ENST00000322652.5	SUZ12 polycomb repressive complex 2 subunit	104	1	0	1	0	0hsa-miR-27a-3p	-0.03	-0.17N/A
CCNA2	ENST00000274026.5	cyclin A2	1162	1	0	0	1	0hsa-miR-27a-3p	-0.03	-0.18N/A
CHKA	ENST00000265689.4	choline kinase alpha	352	1	0	0	1	1hsa-miR-27a-3p	-0.03	-0.15N/A
ACOT11	ENST00000343744.2	acyl-CoA thioesterase 11	36	1	0	1	0	1hsa-miR-27a-3p	-0.03	-0.03N/A
FAM168A	ENST00000064778.4	family with sequence similarity 168, member A	68	1	0	0	1	1hsa-miR-27a-3p	-0.02	-0.04N/A
ZNF704	ENST00000327835.3	zinc finger protein 704	124	1	0	1	0	2hsa-miR-27a-3p	-0.02	-0.05N/A

KRR1	ENST00000229214.4	KRR1, small subunit (SSU) processome component, homolog (yeast)	380	1	0	0	1	1hsa-miR-27a-3p	-0.02	-0.12N/A
MBD5	ENST00000407073.1	methyl-CpG binding domain protein 5	7	1	0	1	0	0hsa-miR-27a-3p	-0.02	-0.1N/A
FAM120A	ENST00000333936.5	family with sequence similarity 120A	57	1	0	0	1	1hsa-miR-27a-3p	-0.02	-0.12N/A
SPATA2	ENST00000289431.5	spermatogenesis associated 2	358	1	1	0	0	1hsa-miR-27a-3p	-0.02	-0.16N/A
DGKH	ENST00000261491.5	diacylglycerol kinase, eta	46	1	0	0	1	0hsa-miR-27a-3p	-0.02	-0.04N/A
EPHB2	ENST00000374632.3	EPH receptor B2	297	1	0	1	0	0hsa-miR-27a-3p	-0.02	-0.09N/A
POU4F1	ENST00000377208.5	POU class 4 homeobox 1	61	1	0	0	1	1hsa-miR-27a-3p	-0.02	-0.11N/A
RCOR3	ENST00000367005.4	REST corepressor 3	374	1	1	0	0	0hsa-miR-27a-3p	-0.02	-0.28N/A
HTT	ENST00000355072.5	huntingtin	731	1	1	0	0	4hsa-miR-27a-3p	-0.02	-0.05N/A
ACVR2A	ENST00000241416.7	activin A receptor, type IIA	109	1	1	0	0	2hsa-miR-27a-3p	-0.02	-0.13N/A
A1CF	ENST00000374001.2	APOBEC1 complementation factor	80	1	1	0	0	2hsa-miR-27a-3p	-0.02	-0.11N/A
PITPNM2	ENST00000280562.5	phosphatidylinositol transfer protein, membrane-associated 2	66	1	0	1	0	0hsa-miR-27a-3p	-0.02	-0.02N/A
PAPPA2	ENST00000367662.3	pappalysin 2	5	1	0	0	1	2hsa-miR-27a-3p	-0.02	-0.02N/A
TET3	ENST00000409262.3	tet methylcytosine dioxygenase 3	1437	1	0	0	1	1hsa-miR-27a-3p	-0.02	-0.02N/A
SHANK3	ENST00000414786.2	SH3 and multiple ankyrin repeat domains 3	5	1	0	1	0	1hsa-miR-27a-3p	-0.02	-0.02N/A
ITPKC	ENST00000263370.2	inositol-trisphosphate 3-kinase C	2682	1	0	1	0	0hsa-miR-27a-3p	-0.02	-0.02N/A
BAHD1	ENST00000416165.1	bromo adjacent homology domain containing 1	76	1	0	1	0	1hsa-miR-27a-3p	-0.02	-0.02N/A
FRMPD3	ENST00000276185.4	FERM and PDZ domain containing 3	0	1	0	1	0	0hsa-miR-27a-3p	-0.02	-0.02N/A
ADCY3	ENST00000260600.5	adenylate cyclase 3	479	1	0	1	0	0hsa-miR-27a-3p	-0.02	-0.02N/A
ZNF827	ENST00000379448.4	zinc finger protein 827	17	1	0	1	0	0hsa-miR-27a-3p	-0.02	-0.02N/A
IPMK	ENST00000373935.3	inositol polyphosphate multikinase	196	1	1	0	0	0hsa-miR-27a-3p	-0.02	-0.34N/A
SUV420H1	ENST00000304363.4	suppressor of variegation 4-20 homolog 1 (Drosophila)	62	1	0	0	1	0hsa-miR-27a-3p	-0.02	-0.05N/A
NCAM2	ENST00000400546.1	neural cell adhesion molecule 2	64	1	0	1	0	0hsa-miR-27a-3p	-0.02	-0.09N/A
MED13	ENST00000397786.2	mediator complex subunit 13	561	1	1	0	0	0hsa-miR-27a-3p	-0.02	-0.03N/A
ALG9	ENST00000531154.1	ALG9, alpha-1,2-mannosyltransferase	586	3	0	2	1	2hsa-miR-27a-3p	-0.02	-0.42N/A
ZRANB1	ENST00000359653.4	zinc finger, RAN-binding domain containing 1	724	1	0	0	1	2hsa-miR-27a-3p	-0.02	-0.05N/A
EPC1	ENST00000319778.6	enhancer of polycomb homolog 1 (Drosophila)	102	1	0	0	1	0hsa-miR-27a-3p	-0.02	-0.11N/A
STK40	ENST00000359297.2	serine/threonine kinase 40	214	1	0	1	0	2hsa-miR-27a-3p	-0.02	-0.02N/A
PLXND1	ENST00000393239.1	plexin D1	211	1	1	0	0	1hsa-miR-27a-3p	-0.02	-0.06N/A
CDH6	ENST00000265071.2	cadherin 6, type 2, K-cadherin (fetal kidney)	213	1	0	1	0	0hsa-miR-27a-3p	-0.02	-0.11N/A
FOXN3	ENST00000345097.4	forkhead box N3	42	1	0	1	0	1hsa-miR-27a-3p	-0.02	-0.15N/A
KCMF1	ENST00000409785.4	potassium channel modulatory factor 1	1285	1	1	0	0	0hsa-miR-27a-3p	-0.02	-0.23N/A
MAP2K6	ENST00000590474.1	mitogen-activated protein kinase kinase 6	235	1	1	0	0	1hsa-miR-27a-3p	-0.02	-0.16N/A
PLEKHA6	ENST00000272203.3	pleckstrin homology domain containing, family A member 6	45	1	0	0	1	0hsa-miR-27a-3p	-0.02	-0.04N/A
SLC22A23	ENST00000436008.2	solute carrier family 22, member 23	353	1	0	1	0	2hsa-miR-27a-3p	-0.02	-0.25N/A
SLC24A4	ENST00000393265.2	solute carrier family 24 (sodium/potassium/calcium exchanger), member 4	5	2	1	1	0	6hsa-miR-27a-3p	-0.02	-0.37N/A
PISD	ENST00000382151.2	phosphatidylserine decarboxylase	61	1	0	0	1	0hsa-miR-27a-3p	-0.01	-0.14N/A
NACC2	ENST00000371753.1	NACC family member 2, BEN and BTB (POZ) domain containing	172	1	0	1	0	1hsa-miR-27a-3p	-0.01	-0.02N/A
P2RY2	ENST00000311131.2	purinergic receptor P2Y, G-protein coupled, 2	66	1	1	0	0	2hsa-miR-27a-3p	-0.01	-0.05N/A
AAK1	ENST00000409085.4	AP2 associated kinase 1	27	1	0	0	1	4hsa-miR-27a-3p	-0.01	-0.04N/A
SMAD2	ENST00000262160.6	SMAD family member 2	1196	2	0	2	0	2hsa-miR-27a-3p	-0.01	-0.15N/A
SP7	ENST00000536324.2	Sp7 transcription factor	5	1	0	0	1	0hsa-miR-27a-3p	-0.01	-0.01N/A
STRN	ENST00000263918.4	striatin, calmodulin binding protein	476	1	0	0	1	3hsa-miR-27a-3p	-0.01	-0.02N/A
KIAA0513	ENST00000566428.1	KIAA0513	5	1	0	0	1	4hsa-miR-27a-3p	-0.01	-0.01N/A
PTCHD4	ENST00000339488.4	patched domain containing 4	19	1	1	0	0	0hsa-miR-27a-3p	-0.01	-0.03N/A
RIMS3	ENST00000372684.3	regulating synaptic membrane exocytosis 3	24	1	0	1	0	1hsa-miR-27a-3p	-0.01	-0.03N/A
INO80	ENST00000361937.3	INO80 complex subunit	279	1	0	0	1	4hsa-miR-27a-3p	-0.01	-0.02N/A
SPHAR	ENST00000366688.3	S-phase response (cyclin related)	202	1	0	1	0	0hsa-miR-27a-3p	-0.01	-0.4N/A
SRGAP3	ENST00000383836.3	SLIT-ROBO Rho GTPase activating protein 3	38	1	0	0	1	2hsa-miR-27a-3p	-0.01	-0.05N/A
ZNRF2	ENST00000323037.4	zinc and ring finger 2	403	1	0	0	1	0hsa-miR-27a-3p	-0.01	-0.12N/A
KIAA2022	ENST00000055682.6	KIAA2022	5	1	0	0	1	2hsa-miR-27a-3p	-0.01	-0.01N/A
ACSBG1	ENST00000258873.4	acyl-CoA synthetase bubblegum family member 1	5	1	0	0	1	1hsa-miR-27a-3p	-0.01	-0.01N/A
NAV1	ENST00000295624.6	neuron navigator 1	702	1	0	0	1	2hsa-miR-27a-3p	-0.01	-0.01N/A
PLAGL2	ENST00000246229.4	pleiomorphic adenoma gene-like 2	254	1	1	0	0	0hsa-miR-27a-3p	-0.01	-0.09N/A
DYRK3	ENST00000367108.3	dual-specificity tyrosine-(Y)-phosphorylation regulated kinase 3	173	1	1	0	0	0hsa-miR-27a-3p	-0.01	-0.22N/A
FSTL4	ENST00000265342.7	follistatin-like 4	60	1	0	0	1	0hsa-miR-27a-3p	-0.01	-0.01N/A
ZBTB3	ENST00000394807.3	zinc finger and BTB domain containing 3	96	1	1	0	0	0hsa-miR-27a-3p	-0.01	-0.03N/A

RPN1	ENST00000296255.3	ribophorin I	9235	1	1	0	0	0hsa-miR-27a-3p	-0.01	-0.49N/A
ACVR2B	ENST00000352511.4	activin A receptor, type IIB	21	1	0	0	1	2hsa-miR-27a-3p	-0.01	-0.02N/A
CA12	ENST00000178638.3	carbonic anhydrase XII	42	1	0	1	0	0hsa-miR-27a-3p	-0.01	-0.02N/A
PAQR3	ENST00000512733.1	progestin and adipoQ receptor family member III	217	1	1	0	0	0hsa-miR-27a-3p	-0.01	-0.16N/A
PIK3CA	ENST00000263967.3	phosphatidylinositol-4,5-bisphosphate 3-kinase, catalytic subunit alpha	112	1	1	0	0	2hsa-miR-27a-3p	-0.01	-0.19N/A
TRIM13	ENST00000378182.3	tripartite motif containing 13	139	1	1	0	0	1hsa-miR-27a-3p	-0.01	-0.11N/A
SESN3	ENST00000536441.1	sestrin 3	260	1	0	0	1	1hsa-miR-27a-3p	-0.01	-0.1N/A
KCNMB4	ENST00000258111.4	potassium large conductance calcium-activated channel, subfamily M, beta member 4	486	1	1	0	0	0hsa-miR-27a-3p	-0.01	-0.26N/A
ASF1A	ENST00000229595.5	anti-silencing function 1A histone chaperone	790	1	0	0	1	0hsa-miR-27a-3p	0	-0.07N/A
TRIP4	ENST00000261884.3	thyroid hormone receptor interactor 4	143	2	2	0	0	2hsa-miR-27a-3p	0	-0.2N/A
HIC2	ENST00000407464.2	hypermethylated in cancer 2	28	1	0	0	1	1hsa-miR-27a-3p	0	-0.02N/A
ADAP1	ENST00000265846.5	ArfGAP with dual PH domains 1	328	1	1	0	0	1hsa-miR-27a-3p	0	-0.3N/A
FDX1	ENST00000260270.2	ferredoxin 1	510	1	1	0	0	1hsa-miR-27a-3p	0	-0.46N/A
SSBP2	ENST00000320672.4	single-stranded DNA binding protein 2	1996	1	0	0	1	0hsa-miR-27a-3p	0	-0.09N/A
SLC16A7	ENST00000261187.4	solute carrier family 16 (monocarboxylate transporter), member 7	73	1	0	1	0	1hsa-miR-27a-3p	0	-0.02N/A
CADM1	ENST00000452722.3	cell adhesion molecule 1	676	1	0	1	0	0hsa-miR-27a-3p	0	-0.21N/A
CPNE2	ENST00000290776.8	copine II	1469	1	0	1	0	0hsa-miR-27a-3p	0	-0.31N/A
DIRC2	ENST00000261038.5	disrupted in renal carcinoma 2	1041	1	0	0	1	0hsa-miR-27a-3p	0	-0.21N/A
CACNG2	ENST00000300105.6	calcium channel, voltage-dependent, gamma subunit 2	5	2	1	1	0	2hsa-miR-27a-3p	0	-0.52N/A
GPR174	ENST00000276077.1	G protein-coupled receptor 174	5	1	1	0	0	2hsa-miR-27a-3p	0	-0.54N/A
PAX6	ENST00000419022.1	paired box 6	191	1	0	1	0	0hsa-miR-27a-3p	0	-0.03N/A
ARIH1	ENST00000379887.4	ariadne RBR E3 ubiquitin protein ligase 1	671	1	1	0	0	1hsa-miR-27a-3p	0	-0.03N/A
ZBTB20	ENST00000462705.1	zinc finger and BTB domain containing 20	5	4	2	2	0	6hsa-miR-27a-3p	0	-0.43N/A
RGPD4	ENST00000408999.3	RANBP2-like and GRIP domain containing 4	5	2	2	0	0	1hsa-miR-27a-3p	0	-0.42N/A
GABRB1	ENST00000295454.3	gamma-aminobutyric acid (GABA) A receptor, beta 1	5	1	0	1	0	1hsa-miR-27a-3p	0	-0.26N/A
SEC31B	ENST00000370345.3	SEC31 homolog B (S. cerevisiae)	43	1	0	0	1	3hsa-miR-27a-3p	0	-0.01N/A
G6PC2	ENST00000421979.1	glucose-6-phosphatase, catalytic, 2	5	1	0	1	0	0hsa-miR-27a-3p	0	-0.3N/A
RGPD3	ENST00000409886.3	RANBP2-like and GRIP domain containing 3	5	1	1	0	0	1hsa-miR-27a-3p	0	-0.26N/A
GRIN2A	ENST00000562109.1	glutamate receptor, ionotropic, N-methyl D-aspartate 2A	5	1	0	0	1	2hsa-miR-27a-3p	0	-0.22N/A
RGPD8	ENST00000302558.3	RANBP2-like and GRIP domain containing 8	5	1	1	0	0	1hsa-miR-27a-3p	0	-0.22N/A
SMIM17	ENST00000598409.1	small integral membrane protein 17	5	1	1	0	0	0hsa-miR-27a-3p	0	-0.24N/A
CACNA1A	ENST00000573710.2	calcium channel, voltage-dependent, P/Q type, alpha 1A subunit	5	1	1	0	0	1hsa-miR-27a-3p	0	-0.15N/A
SLITRK3	ENST00000475390.1	SLIT and NTRK-like family, member 3	5	1	0	1	0	0hsa-miR-27a-3p	0	-0.18N/A
ORC6	ENST00000568364.2	origin recognition complex, subunit 6	855	1	0	0	1	0hsa-miR-27a-3p	0	-0.12N/A
DCC	ENST00000442544.2	deleted in colorectal carcinoma	5	1	0	1	0	0hsa-miR-27a-3p	0	-0.08N/A
GOLGA6B	ENST00000421285.3	golgin A6 family, member B	5	1	0	1	0	0hsa-miR-27a-3p	0	-0.13N/A
ROBO1	ENST00000436010.2	roundabout, axon guidance receptor, homolog 1 (Drosophila)	546	1	1	0	0	0hsa-miR-27a-3p	0	-0.03N/A
SHC3	ENST00000375835.4	SHC (Src homology 2 domain containing) transforming protein 3	18	1	0	0	1	4hsa-miR-27a-3p	0	-0.12N/A
DCUN1D5	ENST00000260247.5	DCN1, defective in cullin neddylation 1, domain containing 5	1486	1	0	1	0	3hsa-miR-27a-3p	0	-0.15N/A
TGFBR2	ENST00000359013.4	transforming growth factor, beta receptor II (70/80kDa)	2168	1	1	0	0	2hsa-miR-27a-3p	0	-0.03N/A
SFPQ	ENST00000357214.5	splicing factor proline/glutamine-rich	4735	1	0	1	0	0hsa-miR-27a-3p	0	-0.02N/A

Table S3a. Identification of potential candidate target genes of miR-23a&miR-27 by a combined RNA-seq and miR-23a Targetscan data

Symobol	GeneID	23aKO1 FPKM	23AKO2 FPKM	27aKO1 FPKM	27aKO2 FPKM	Scr1 FPKM	Scr2 FPKM	log2Ratio 23aKO/Scr	q-value	p-value	log2Ratio 27aKO/Scr	q-value	p-value
ANKRD17	26057	3.92	19.61	11.42	10.91	6.07	8.25	0.69	0	0	0.68	0	0
CCAR1	55749	2.19	10.61	5.72	5.82	1.82	5.23	0.71975	0	0	0.62719	0	0
CDKN2AIP	55602	1.54	4.34	2	2.64	1.13	1.81	0.99058	0	0	0.69721	0	0
CEP350	9857	0.14	0.88	0.33	0.35	0.11	0.26	1.39761	0	0	0.84816	0.00001	0
CLK4	57396	0.3	1.64	1.03	1.2	0.42	0.85	0.90385	0.00003	0.00001	0.75691	0.00082	0.00034
CREBZF	58487	1.3	3.74	2.51	2.67	1.33	1.84	0.76578	0	0	0.74396	0	0
DEPDC1	55635	0.84	2.86	0.88	1.1	0.43	0.88	1.49499	0	0	0.63244	0.0004	0.00016
DHX15	1665	12.8	41.11	25.89	25.78	14.51	17.86	0.72886	0	0	0.66565	0	0
DLD	1738	2.35	9.79	4.68	4.77	2.09	3.16	1.13725	0	0	0.7461	0	0
EIF3A	8661	5.71	27.95	14.28	13.89	5.26	9.16	1.11167	0	0	0.90628	0	0
EML5	161436	0.12	0.26	0.12	0.14	0.03	0.07	2.00981	0	0	1.47622	0.00047	0.00019
EPS15	2060	1.02	5.53	3.71	3.17	2.03	2.15	0.5881	0	0	0.69717	0	0
GNG10	2790	8.49	13.88	13.9	14.43	6.01	2.29	1.42794	0	0	1.77291	0	0
GTPBP10	85865	3.46	6.95	3.39	3.27	2.59	3.05	1.18679	0	0	0.79039	0	0
HELZ	85441	2.88	3.68	3.81	3.31	4.44	2.53	0.05368	0.48358	0.37914	0.12431	0.06663	0.03992
HMGB2	3148	37.85	74.14	59.96	62.24	33.78	30.79	0.78683	0	0	0.90216	0	0
HNRNPU	3192	23.93	78.81	55.13	55.37	29.18	35.97	0.6127	0	0	0.7481	0	0
LBR	3930	6.16	19.51	14.72	12.63	6.97	8.47	0.72753	0	0	0.82872	0	0
NUCKS1	64710	9.93	40.81	23.47	24.66	12.25	14.9	0.87508	0	0	0.82619	0	0
PBRM1	55193	0.71	3.88	2.32	2.15	0.93	1.76	0.62526	0	0	0.75236	0	0
PIK3R3	8503	0.79	1.3	1.05	1.1	0.46	0.77	0.84097	0	0	0.86795	0	0
PLEKHF2	79666	1.4	3.13	1.72	1.61	0.77	1.32	1.06295	0	0	0.67838	0.00021	0.00008
POP1	10940	1.71	4.95	2.89	2.89	1.93	1.7	0.63237	0	0	0.6758	0	0
PRRG1	5638	0.39	1.5	1.28	1.23	0.59	0.57	0.71406	0.00012	0.00005	0.90683	0	0
PTGR2	145482	0.69	1.39	1.14	1.26	0.33	0.47	1.35894	0	0	1.58694	0	0
QSER1	79832	0.49	3.83	1.79	1.67	0.71	1.48	0.95368	0	0	0.66619	0	0
RAB8B	51762	0.48	2.02	0.86	0.9	0.6	0.51	1.1572	0	0	0.65399	0.00075	0.00031
RBM27	54439	0.82	3.88	1.85	1.87	1.11	1.32	0.90598	0	0	0.60567	0	0
SEPSECS	51091	0.83	1.41	0.99	0.95	0.46	0.7	0.89191	0	0	0.75961	0	0

SLC2A4RG	56731	141.52	47.55	105.31	101.67	59.98	65.81	0.58283	0	0	0.73748	0	0
SLC30A9	10463	3.04	11.95	4.75	4.79	2.25	3.76	1.27246	0	0	0.61064	0	0
TNKS2	80351	0.83	5.97	3.36	3.74	1.62	2.7	0.6231	0	0	0.71026	0	0
TOP1	7150	8.4	36.15	21.63	22.74	10.9	15.72	0.72181	0	0	0.73314	0	0
TRIM24	8805	11.17	32.88	19.36	19.4	8.05	15.26	0.91554	0	0	0.74429	0	0
ZNF286B	729288	0.43	1	0.66	0.9	0.49	0.5	1.0968	0	0	0.96591	0.00001	0
BMPR2	659	0.63	2.76	1.75	1.87	1.05	1.12	0.59852	0	0	0.8831	0	0
NAA50	80218	2.02	10.36	2.33	4.19	1.52	2	1.49185	0	0	0.7637	0	0
PURA	5813	0.94	1.88	1.57	1.53	0.76	1.03	0.64857	0	0	0.77741	0	0
RBM12B	389677	0.41	1.9	0.9	0.8	0.42	0.51	0.94912	0	0	0.62167	0.00007	0.00002
RYBP	23429	1.46	5.82	2.94	3.27	1.91	2.23	0.80395	0	0	0.58562	0	0
TARDBP	23435	15.55	38.89	26.56	25.06	13.47	16.32	0.76445	0	0	0.77022	0	0
VCAN	1462	0.32	4.34	0.67	0.87	0.58	0.18	3.16955	0	0	1.29785	0	0

Table S3b. Identification of potential candidate target genes of miR-23a&miR-27 by a combined RNA-seq and miR-27a Targetscan data

Symobol	GeneID	23aKO1 FPKM	23aKO2 FPKM	27aKO1 FPKM	27aKO2 FPKM	Scr1 FPKM	Scr2 FPKM	log2Ratio 23aKO/Scr	q-value	p-value	log2Ratio 27aKO/Scr	q-value	p-value
ANKRD17	26057	3.92	19.61	11.42	10.91	6.07	8.25	0.69	0	0	0.68	0	0
AGGF1	55109	0.69	2.84	1.74	1.61	0.55	1.19	1.01	0	0	0.95	0	0
ANKRD17	26057	3.92	19.61	11.42	10.91	6.07	8.25	0.69	0	0	0.68	0	0
APAF1	317	0.66	2.91	1.29	1.3	0.57	0.91	1.28	0	0	0.73	0	0
BLOC1S5	63915	2.79	8.49	5.81	5.85	1.8	3.49	1.08	0	0	1.13	0	0
BNIP3L	665	2.82	14.01	8.19	8.68	5.58	4.41	0.74	0	0	0.76	0	0
C1GALT1	56913	2.67	6.75	5.2	5.98	2.12	3.47	0.63	0	0	1.16	0	0
CALD1	800	5.75	24.26	14.77	14.93	7.4	11.75	0.64	0	0	0.63	0	0
CAND1	55832	2.82	13.12	6.56	6.54	3.07	5.02	0.95	0	0	0.69	0	0
CAPN7	23473	2.01	6.66	3.05	3.04	1.63	2.11	1.16	0	0	0.65	0	0
CCNT1	904	0.56	3.22	2.06	1.68	0.96	1.21	0.83	0	0	0.80	0	0
CDK6	1021	0.31	7.29	2.95	2.96	0.93	2.43	1.22	0	0	0.77	0	0
COMMD3	100532731	0.59	3.96	0.17	2.46	1.43	0.32	0.46	0	0	-0.07	0.34	0.25
CREB1	1385	1.18	5.11	2.91	2.62	2.06	1.83	1.01	0	0	0.65	0	0
DCUN1D4	23142	0.98	3.92	1.63	1.95	0.93	1.33	1.15	0	0	0.64	0	0
DDI2	84301	1.83	12.36	8.02	8.82	3.38	5.15	0.66	0	0	1.11	0	0
DEPDC1B	55789	9.58	17.78	12.4	11.87	6.11	9.4	0.82	0	0	0.69	0	0
DNAJC13	23317	1.14	3.79	2.52	2.23	1.05	1.66	1.11	0	0	0.62	0	0
DTX4	23220	5.02	4.83	5.47	5.28	2.33	4.19	0.61	0	0	0.72	0	0
E2F7	144455	0.58	2.85	1.96	2.02	0.93	1.08	0.76	0	0	1.01	0	0
ENAH	55740	0.76	7.36	4.66	4.05	2.4	2.41	0.72	0	0	0.87	0	0
FAM133B	257415	3.69	8.23	7.3	7.9	3.71	4.07	0.58	0	0	0.92	0	0
FAM217B	63939	1.26	3.44	2.45	2.65	1.17	1.44	1.00	0	0	0.91	0	0
FAM69A	388650	1.04	4.46	2.98	2.83	1.25	2.28	0.60	0	0	0.59	0	0
FEM1B	10116	1.07	3.19	2.04	1.95	0.96	1.51	0.72	0	0	0.69	0	0
FRYL	285527	0.08	1.23	0.42	0.49	0.17	0.3	1.60	0	0	1.01	0	0
GATC	283459	16.46	11.05	15.34	14.14	8	9.79	0.62	0	0	0.74	0	0
GCC2	9648	0.24	2.26	0.61	1.1	0.43	0.53	1.00	0	0	0.65	0	0
GOLGA8A	23015	0.11	1.92	0.81	0.99	0.41	0.45	1.45	0	0	1.14	0	0

GPATCH2L	55668	0.6	1.9	1.29	1.16	1.07	0.99	0.75	0	0	0.61	0	0
HAUS1	115106	11.42	18.19	11.07	12.81	5.45	9.27	1.00	0	0	0.69	0	0
HIPK3	10114	0.66	3.58	2.08	1.93	0.89	1.47	0.85	0	0	0.77	0	0
HSPD1	3329	155.96	512.5	335.32	344.33	169.87	236.93	0.71	0	0	0.74	0	0
KATNBL1	79768	2.68	3.07	2.08	2.72	1.63	1.67	0.75	0	0	0.67	0	0
KDELC2	143888	2.26	12.05	5.94	5.33	3.04	4.08	1.00	0	0	0.67	0	0
KDM3A	55818	1.87	11.19	5.42	5.15	3.3	3.4	1.02	0	0	0.66	0	0
KITLG	4254	0.35	2.2	1.15	0.92	0.46	0.73	1.11	0	0	0.77	0	0
KPNB1	3837	20.18	79.74	53.63	53.89	24.78	32.45	0.81	0	0	0.91	0	0
KRAS	3845	0.17	2.39	1.09	1.61	0.61	0.53	1.14	0	0	1.21	0	0
LIN7C	55327	0.55	2.56	1.36	1.61	0.6	0.96	0.97	0	0	0.93	0	0
LRBA	987	0.95	7.27	5.53	5.52	2.8	4.37	0.61	0	0	0.70	0	0
LRP6	4040	0.52	2.71	1.73	1.74	0.71	1.18	0.73	0	0	0.87	0	0
MAP2K6	5608	0.26	0.84	0.48	0.78	0.25	0.12	1.63	0	0	1.95	0	0
MAP3K4	4216	0.94	6.54	3.51	3.43	1.76	1.98	0.96	0	0	0.88	0	0
MBTD1	54799	0.57	3.87	2.67	2.36	1.2	1.67	0.59	0	0	0.80	0	0
MDN1	23195	0.27	2.94	1.67	1.61	0.43	0.93	1.59	0	0	1.60	0	0
MIER3	166968	0.46	2.07	1.12	0.93	0.45	0.78	0.94	0	0	0.68	0	0
MYH10	4628	3.87	20.79	14.99	14.15	6.99	7.2	0.78	0	0	1.03	0	0
NGLY1	55768	4.16	10.51	6.91	8.26	4.03	5.14	0.61	0	0	0.66	0	0
NRBF2	29982	5.91	14.76	9.79	10.99	7.04	6.88	0.59	0	0	0.58	0	0
NXT2	55916	2.35	3.17	2.37	2.29	0.86	1.99	0.96	0	0	0.71	0	0
ORC5	5001	3.63	10.18	7.32	8.05	3.56	4.99	0.69	0	0	0.86	0	0
PDK1	5163	3.33	10.76	7.37	6.5	4.26	3.78	0.83	0	0	0.80	0	0
PLAG1	5324	0.07	0.6	0.36	0.36	0.12	0.24	0.89	0	0	1.05	0	0
PNRC2	55629	19.19	58.71	34.39	34.26	14.04	26.31	0.94	0	0	0.76	0	0
POU4F1	5457	0.3	1.48	0.9	1.1	0.51	0.52	0.23	0.13	0.08	0.43	0	0
PPP4R2	151987	4.43	13.16	9.67	8.37	3.06	5.74	0.90	0	0	1.00	0	0
PPP6R3	55291	3.6	13.82	7.72	8.41	3.65	5.42	0.89	0	0	0.84	0	0
RAB33B	83452	0.47	1.54	1.32	1.07	0.57	0.65	0.69	0	0	0.95	0	0
RASA2	5922	0.19	1.14	0.58	0.52	0.32	0.28	0.98	0	0	0.96	0	0
RC3H1	149041	0.2	1.22	0.81	0.76	0.16	0.74	0.63	0	0	0.81	0	0
RGPD5	84220	0.09	1.37	0.82	0.69	0.5	0.23	0.88	0	0	0.92	0	0

RGPD6	729540	0.24	2.15	0.66	0.59	0.23	0.65	1.43	0	0	0.65	0	0
RGPD8	727851	0.1	0.81	0.5	0.61	0.2	0.28	0.86	0	0	1.25	0	0
RLIM	51132	0.39	2.38	1.19	1.23	0.74	0.85	0.71	0	0	0.60	0	0
RNGTT	8732	0.87	4.56	1.82	2.02	1.11	1.19	1.23	0	0	0.73	0	0
SENP1	29843	1.15	5.43	2.63	2.84	1.37	1.94	0.99	0	0	0.64	0	0
SHC3	53358	1.02	2.28	1.5	1.25	0.7	0.92	-0.17	0.79	0.72	0.46	0.37	0.28
SLC35A3	23443	0.5	1.4	0.91	0.83	0.32	0.63	0.90	0	0	0.89	0	0
SMCHD1	23347	0.35	3.97	1.68	1.82	0.7	1.54	0.85	0	0	0.58	0	0
SOCS6	9306	1.08	3.56	2.1	2.2	1.08	1.58	0.80	0	0	0.70	0	0
SPTY2D1	144108	0.97	3.87	1.7	1.6	0.95	1.23	1.13	0	0	0.59	0	0
SRSF1	6426	28.94	76.94	53.63	53.96	24.3	33.68	0.91	0	0	0.90	0	0
SSX2IP	117178	0.62	3.1	2	1.79	0.56	1.17	1.05	0	0	1.07	0	0
SUB1	10923	89.88	67.8	84.04	91.67	47.88	60.41	0.61	0	0	0.79	0	0
SUZ12	23512	1.66	5.88	3.06	2.98	0.97	2.71	1.09	0	0	0.76	0	0
TET1	80312	0.23	1.03	0.51	0.49	0.26	0.35	0.98	0	0	0.69	0	0
TGFBR1	7046	0.36	1.7	1.43	1.48	0.53	0.82	0.59	0	0	1.10	0	0
TMEM170B	1E+08	0.33	0.77	0.6	0.62	0.28	0.43	0.78	0	0	0.94	0	0
TMX1	81542	2.82	9.3	3.39	4.55	1.89	1.83	1.52	0	0	1.32	0	0
UBR1	197131	0.29	1.41	0.6	0.52	0.26	0.44	0.04	0.74	0.65	0.90	0	0
UBXN2A	165324	2.35	9.85	7.28	6.47	3.36	3.85	0.62	0	0	0.82	0	0
XPO1	7514	7.62	50.36	22.19	22.29	10.9	16.38	1.12	0	0	0.71	0	0
ZBTB34	403341	0.32	1.14	0.83	0.87	0.48	0.49	0.65	0	0	0.92	0	0
ZMYM4	9202	0.67	4.9	2.87	2.26	0.83	1.54	-1.94	0.11	0.07	-1.21	0.27	0.2
ZNF106	64397	0.96	5.66	3.92	3.31	2.17	2.39	0.62	0	0	0.69	0	0
ZZZ3	26009	0.99	4.29	1.69	2.2	0.95	1.57	1.10	0	0	0.77	0	0
BMPR2	659	0.63	2.76	1.75	1.87	1.05	1.12	0.60	0	0	0.88	0	0
NAA50	80218	2.02	10.36	2.33	4.19	1.52	2	1.49	0	0	0.76	0	0
PURA	5813	0.94	1.88	1.57	1.53	0.76	1.03	0.65	0	0	0.78	0	0
RBM12B	389677	0.41	1.9	0.9	0.8	0.42	0.51	0.95	0	0	0.62	0	0
RYBP	23429	1.46	5.82	2.94	3.27	1.91	2.23	0.80	0	0	0.59	0	0
TARDBP	23435	15.55	38.89	26.56	25.06	13.47	16.32	0.76	0	0	0.77	0	0
VCAN	1462	0.32	4.34	0.67	0.87	0.58	0.18	3.17	0	0	1.30	0	0

Table S3c. Identification of potential candidate target genes of miR-23a&miR-27 by a combined RNA-seq and Targetscan data

Symobol	GeneID	23aKO1 FPKM	23aKO2 FPKM	27aKO1 FPKM	27aKO2 FPKM	Scr1 FPKM	Scr2 FPKM	log2Ratio 23aKO/Scr	q-value	p-value	log2Ratio 27aKO/Scr	q-value	p-value
BMPR2	659	0.63	2.76	1.75	1.87	1.05	1.12	0.59852	0	0	0.8831	0	0
NAA50	80218	2.02	10.36	2.33	4.19	1.52	2	1.49185	0	0	0.7637	0	0
PURA	5813	0.94	1.88	1.57	1.53	0.76	1.03	0.64857	0	0	0.77741	0	0
RBM12B	389677	0.41	1.9	0.9	0.8	0.42	0.51	0.94912	0	0	0.62167	0.00007	0.00002
RYBP	23429	1.46	5.82	2.94	3.27	1.91	2.23	0.80395	0	0	0.58562	0	0
TARDBP	23435	15.55	38.89	26.56	25.06	13.47	16.32	0.76445	0	0	0.77022	0	0
VCAN	1462	0.32	4.34	0.67	0.87	0.58	0.18	3.16955	0	0	1.29785	0	0

Table S4. Identification of potential target signaling pathways of miR-23a&miR-27a by GSEA analysis using RNA-seq data

No	GS follow link to MSigDB	SIZE	ES	NES	NOM p-val	FDR q-val	FWER p-val	RANK AT MAX	LEADING EDGE
**Up-regulation**									
1	**HALLMARK_E2F_TARGETS**	198	0.61	2.59	0	0	0	4315	tags=64%, list=22%, signal=81%
2	HALLMARK_G2M_CHECKPOINT	195	0.58	2.45	0	0	0	4315	tags=58%, list=22%, signal=74%
3	HALLMARK_MYC_TARGETS_V1	196	0.53	2.25	0	0	0	6402	tags=60%, list=32%, signal=87%
4	HALLMARK_MITOTIC_SPINDLE	198	0.38	1.63	0	0.01	0.067	6196	tags=53%, list=31%, signal=75%
5	HALLMARK_DNA_REPAIR	150	0.4	1.63	0.001	0.01	0.072	7005	tags=51%, list=35%, signal=78%
6	HALLMARK_MYC_TARGETS_V2	57	0.46	1.61	0.007	0.01	0.081	3971	tags=37%, list=20%, signal=46%
7	HALLMARK_PROTEIN_SECRETION	95	0.35	1.33	0.0575	0.15	0.792	5737	tags=40%, list=29%, signal=56%
8	HALLMARK_OXIDATIVE_PHOSPHORYLATION	200	0.31	1.29	0.0341	0.19	0.917	8476	tags=49%, list=42%, signal=84%
9	HALLMARK_PI3K_AKT_MTOR_SIGNALING	99	0.31	1.19	0.1531	0.36	0.995	5851	tags=40%, list=29%, signal=57%
10	HALLMARK_ANDROGEN_RESPONSE	96	0.31	1.19	0.1633	0.34	0.996	5659	tags=41%, list=28%, signal=56%
11	HALLMARK_MTORC1_SIGNALING	195	0.28	1.15	0.1739	0.41	1	5659	tags=36%, list=28%, signal=50%
12	HALLMARK_ADIPOGENESIS	191	0.23	0.95	0.6091	1	1	8176	tags=45%, list=41%, signal=75%
13	HALLMARK_WNT_BETA_CATENIN_SIGNALING	42	0.29	0.94	0.5327	1	1	4674	tags=33%, list=23%, signal=43%
14	HALLMARK_FATTY_ACID_METABOLISM	151	0.23	0.92	0.6629	1	1	7485	tags=44%, list=37%, signal=69%
15	HALLMARK_UV_RESPONSE_DN	132	0.23	0.88	0.7329	1	1	5333	tags=33%, list=27%, signal=44%
16	HALLMARK_HEME_METABOLISM	180	0.21	0.87	0.7778	1	1	5652	tags=32%, list=28%, signal=44%
17	HALLMARK_SPERMATOGENESIS	105	0.22	0.84	0.7932	1	1	2526	tags=20%, list=13%, signal=23%
18	HALLMARK_UNFOLDED_PROTEIN_RESPONSE	109	0.22	0.84	0.7856	1	1	7801	tags=50%, list=39%, signal=81%
19	HALLMARK_PEROXISOME	102	0.22	0.84	0.7948	0.99	1	5788	tags=31%, list=29%, signal=44%
20	HALLMARK_INTERFERON_GAMMA_RESPONSE	42	0.29	0.94	0.5327	1	1	4674	tags=33%, list=23%, signal=43%
**Down-regulation**									
1	HALLMARK_MYOGENESIS	175	-0.44	-2.1	0	0	0	3727	tags=36%, list=19%, signal=44%
2	HALLMARK_COAGULATION	125	-0.46	-1.99	0	0	0	3949	tags=39%, list=20%, signal=49%
3	HALLMARK_XENOBIOTIC_METABOLISM	192	-0.44	-1.99	0	0	0	3326	tags=32%, list=17%, signal=38%
4	HALLMARK_TNFA_SIGNALING_VIA_NFKB	183	-0.41	-1.83	0	0.003	0.007	3148	tags=33%, list=16%, signal=39%
5	HALLMARK_INFLAMMATORY_RESPONSE	171	-0.39	-1.76	0	0.007	0.018	3213	tags=30%, list=16%, signal=36%
6	HALLMARK_KRAS_SIGNALING_UP	163	-0.39	-1.75	0	0.006	0.02	3166	tags=34%, list=16%, signal=40%
7	HALLMARK_EPITHELIAL_MESENCHYMAL_TRANSITION	164	-0.38	-1.68	0	0.008	0.031	2684	tags=27%, list=13%, signal=31%
8	HALLMARK_HYPOXIA	188	-0.35	-1.62	0	0.012	0.05	3727	tags=34%, list=19%, signal=41%
9	HALLMARK_COMPLEMENT	177	-0.35	-1.59	0	0.013	0.062	1990	tags=20%, list=10%, signal=22%
10	HALLMARK_ALLOGRAFT_REJECTION	164	-0.33	-1.48	0.004	0.036	0.179	2021	tags=21%, list=10%, signal=23%
11	HALLMARK_IL2_STAT5_SIGNALING	189	-0.31	-1.4	0	0.059	0.301	3638	tags=31%, list=18%, signal=38%
12	HALLMARK_TGF_BETA_SIGNALING	52	-0.38	-1.4	0.044	0.054	0.301	2541	tags=31%, list=13%, signal=35%
13	HALLMARK_ESTROGEN_RESPONSE_EARLY	186	-0.3	-1.39	0.007	0.057	0.334	3317	tags=30%, list=17%, signal=35%
14	HALLMARK_P53_PATHWAY	189	-0.3	-1.37	0.011	0.062	0.384	3720	tags=31%, list=19%, signal=37%
15	HALLMARK_KRAS_SIGNALING_DN	149	-0.3	-1.32	0.02	0.081	0.494	2733	tags=26%, list=14%, signal=29%
16	HALLMARK_ANGIOGENESIS	26	-0.42	-1.31	0.115	0.084	0.532	2473	tags=27%, list=12%, signal=31%
17	HALLMARK_APICAL_SURFACE	41	-0.37	-1.29	0.125	0.09	0.577	3864	tags=37%, list=19%, signal=45%
18	HALLMARK_ESTROGEN_RESPONSE_LATE	181	-0.28	-1.26	0.043	0.11	0.664	3293	tags=25%, list=16%, signal=29%
19	HALLMARK_HEDGEHOG_SIGNALING	31	-0.35	-1.17	0.221	0.206	0.893	3624	tags=29%, list=18%, signal=35%
20	HALLMARK_IL6_JAK_STAT3_SIGNALING	77	-0.28	-1.1	0.239	0.316	0.977	2966	tags=25%, list=15%, signal=29%

Table S5. The core enrichment genes with a high "Rank Metric Score" in G2/M gene set

Name	Gene Symbol	Rank in gene list	Rank in metric score	Running ES	Core enrichment
row_0	RPS6KA5	77	1.379729986	0.010678338	Yes
row_1	GINS2	135	1.218190432	0.02066177	Yes
row_2	EGF	163	1.170769691	0.03166101	Yes
row_3	MNAT1	190	1.135299444	0.042336214	Yes
row_4	HSPA8	215	1.102523088	0.052766383	Yes
row_5	EZH2	313	1.01214242	0.058551654	Yes
row_6	MAD2L1	315	1.011860609	0.06918709	Yes
row_7	PURA	328	1.002687454	0.079169564	Yes
row_8	MCM5	376	0.965291023	0.086987734	Yes
row_9	BARD1	407	0.949721456	0.09550088	Yes
row_10	SRSF1	442	0.92918843	0.10359497	Yes
row_11	TRA2B	444	0.928266823	0.1133476	Yes
row_12	CDC45	515	0.89015156	0.119209506	Yes
row_13	POLA2	525	0.888076127	0.12813325	Yes
row_14	CDK1	539	0.882238567	0.13679315	Yes
row_15	ESPL1	548	0.879973531	0.14568189	Yes
row_16	TMPO	595	0.861278594	0.15245216	Yes
row_17	NCL	597	0.860379219	0.16148785	Yes
row_18	HMGB3	604	0.857618272	0.1702416	Yes
row_19	PRIM2	610	0.855857849	0.17902732	Yes
row_20	TROAP	636	0.84679085	0.18670622	Yes
row_21	KIF4A	683	0.828502119	0.19313034	Yes
row_22	MCM2	694	0.825292706	0.2013405	Yes
row_23	LMNB1	702	0.823160231	0.2096798	Yes
row_24	ORC5	751	0.807665586	0.21578278	Yes
row_25	CDKN3	788	0.796515524	0.22237463	Yes
row_26	CCNF	809	0.791342378	0.22972073	Yes
row_27	G3BP1	819	0.787813604	0.23758563	Yes
row_28	NUSAP1	825	0.786351204	0.24563731	Yes
row_29	HMGN2	826	0.786219895	0.25394037	Yes

row_30	LBR	842	0.781583548	0.26143616	Yes
row_31	TTK	864	0.776032984	0.26857	Yes
row_32	KPNB1	889	0.771303952	0.27550226	Yes
row_33	EXO1	964	0.753738284	0.27972132	Yes
row_34	FBXO5	1095	0.727086604	0.28082794	Yes
row_35	HNRNPU	1167	0.714221954	0.28478134	Yes
row_36	SYNCRIP	1285	0.69420433	0.28619787	Yes
row_37	BUB1	1334	0.686437547	0.2910206	Yes
row_38	NASP	1346	0.684869707	0.29769725	Yes
row_39	SNRPD1	1397	0.677902341	0.3023287	Yes
row_40	POLE	1401	0.677272916	0.30932954	Yes
row_41	CUL1	1466	0.666981578	0.31313792	Yes
row_42	PBK	1501	0.661893606	0.31840917	Yes
row_43	KIF2C	1515	0.660019398	0.3247223	Yes
row_44	TRAIP	1517	0.659789801	0.3316396	Yes
row_45	NUP50	1545	0.654974401	0.33719167	Yes
row_46	NEK2	1578	0.649476826	0.3424329	Yes
row_47	CUL5	1685	0.63387841	0.34376845	Yes
row_48	TACC3	1702	0.631777048	0.3496316	Yes
row_49	MCM3	1736	0.627577424	0.354591	Yes
row_50	HMMR	1777	0.62270087	0.35914508	Yes
row_51	AURKA	1831	0.616383612	0.36297518	Yes
row_52	WRN	1832	0.616044164	0.36948106	Yes
row_53	SRSF2	1849	0.614277959	0.37515944	Yes
row_54	RBL1	1877	0.610192358	0.38023856	Yes
row_55	CCNT1	1921	0.605010688	0.38445413	Yes
row_56	TOP1	1923	0.604896486	0.3907917	Yes
row_57	STMN1	1926	0.604631603	0.39707595	Yes
row_58	TPX2	1960	0.599942863	0.40174353	Yes
row_59	AMD1	2054	0.590317011	0.40327623	Yes
row_60	RACGAP1	2083	0.587880671	0.4080692	Yes
row_61	BIRC5	2096	0.586398721	0.41365534	Yes
row_62	CHEK1	2139	0.582724035	0.41768607	Yes

row_63	BRCA2	2141	0.582619011	0.42378843	Yes
row_64	TOP2A	2143	0.582384586	0.42988828	Yes
row_65	RBM14	2155	0.580483079	0.4354625	Yes
row_66	KIF15	2160	0.579920411	0.44138467	Yes
row_67	SRSF10	2232	0.57265228	0.443843	Yes
row_68	CASP8AP2	2263	0.568865716	0.44833404	Yes
row_69	PRC1	2268	0.568314552	0.45413366	Yes
row_70	KNL1	2385	0.556620836	0.45414776	Yes
row_71	KIF11	2389	0.556485415	0.459873	Yes
row_72	DBF4	2438	0.551995456	0.4632759	Yes
row_73	NDC80	2449	0.551088989	0.46859026	Yes
row_74	XPO1	2455	0.550487816	0.47415105	Yes
row_75	CDC20	2459	0.55020839	0.47981	Yes
row_76	RAD21	2472	0.549335778	0.48500475	Yes
row_77	ATF5	2475	0.548852742	0.49069992	Yes
row_78	DMD	2559	0.540790498	0.49221513	Yes
row_79	MYBL2	2562	0.540708005	0.49782428	Yes
row_80	SMC4	2581	0.538571179	0.50260204	Yes
row_81	CHMP1A	2629	0.533614397	0.5058614	Yes
row_82	KIF22	2665	0.530330002	0.50969267	Yes
row_83	CCNA2	2679	0.528812289	0.5146201	Yes
row_84	PLK4	2744	0.523772418	0.5169161	Yes
row_85	KIF23	2817	0.517840266	0.51874506	Yes
row_86	STIL	2843	0.516160905	0.52293223	Yes
row_87	CBX1	2876	0.513114512	0.5267334	Yes
row_88	RPA2	3078	0.495505244	0.52180505	Yes
row_89	PLK1	3121	0.492740273	0.5248855	Yes
row_90	CENPF	3159	0.489643574	0.52818596	Yes
row_91	MTF2	3182	0.488390774	0.53223157	Yes
row_92	KATNA1	3236	0.483574659	0.53465915	Yes
row_93	YTHDC1	3261	0.481196791	0.53852767	Yes
row_94	PRPF4B	3343	0.476132154	0.53946114	Yes
row_95	DKC1	3403	0.472215891	0.5414654	Yes

row_96	AURKB	3406	0.47170642	0.5463459	Yes
row_97	MKI67	3452	0.468179047	0.5490153	Yes
row_98	RAD23B	3580	0.45904687	0.54744285	Yes
row_99	SS18	3602	0.457433671	0.5512121	Yes
row_100	MAP3K20	3684	0.45111084	0.55188125	Yes
row_101	CDC27	3700	0.450641662	0.5558821	Yes
row_102	E2F1	3783	0.445088685	0.55643713	Yes
row_103	CDC7	3817	0.442413658	0.5594411	Yes
row_104	CUL3	3919	0.435992211	0.5589396	Yes
row_105	DTYMK	3926	0.435177773	0.56323206	Yes
row_106	SMC1A	3954	0.432978541	0.5664397	Yes
row_107	PDS5B	4027	0.427746534	0.5673171	Yes
row_108	SLC12A2	4068	0.424899369	0.56978226	Yes
row_109	STAG1	4135	0.420216531	0.5708835	Yes
row_110	CDC6	4145	0.419474453	0.5748585	Yes
row_111	KIF5B	4172	0.417884886	0.5779573	Yes
row_112	CKS2	4239	0.413489938	0.5789875	Yes

Table S6a. The potential off-targets accessed using the Cas-OFFinder off-target searching tool

sgRNA	Target Sequence	Bulge Type	Bulge Size	Mismatch	Number of Found Targets
23a-sgRNA-1	CACCGACCCCTGTGCCACGGCCGGCNGG	X	0	3	1
23a-sgRNA-1	CACCGACCCCTGTGCCACGGCCGGCNGG	X	0	5	4
23a-sgRNA-1	CACCGACCCCTGTGCCACGGCCGGCNGG	X	0	6	40
23a-sgRNA-1	CACCGACCCCTGTGCCACGGCCGGCNGG	X	0	7	318
23a-sgRNA-1	CACCGACCCCTGTGCCACGGCCGGCNGG	X	0	8	2145
23a-sgRNA-1	CACCGACCCCTGTGCCACGGCCGGCNGG	X	0	9	11390
23a-sgRNA-2	CACCGCAGGGTCGGTTGGAAATCCCNGG	X	0	3	1
23a-sgRNA-2	CACCGCAGGGTCGGTTGGAAATCCCNGG	X	0	6	12
23a-sgRNA-2	CACCGCAGGGTCGGTTGGAAATCCCNGG	X	0	7	155
23a-sgRNA-2	CACCGCAGGGTCGGTTGGAAATCCCNGG	X	0	8	1223
23a-sgRNA-2	CACCGCAGGGTCGGTTGGAAATCCCNGG	X	0	9	7843
27a-sgRNA-1	CACCGTAAGCCCTGCTCCTCAGGCCNGG	X	0	4	1
27a-sgRNA-1	CACCGTAAGCCCTGCTCCTCAGGCCNGG	X	0	5	5
27a-sgRNA-1	CACCGTAAGCCCTGCTCCTCAGGCCNGG	X	0	6	69
27a-sgRNA-1	CACCGTAAGCCCTGCTCCTCAGGCCNGG	X	0	7	438
27a-sgRNA-1	CACCGTAAGCCCTGCTCCTCAGGCCNGG	X	0	8	2605
27a-sgRNA-1	CACCGTAAGCCCTGCTCCTCAGGCCNGG	X	0	9	13051
27a-sgRNA-2	CACCGACACCAAGTCGTGTTCACAGNGG	X	0	4	2
27a-sgRNA-2	CACCGACACCAAGTCGTGTTCACAGNGG	X	0	5	1
27a-sgRNA-2	CACCGACACCAAGTCGTGTTCACAGNGG	X	0	6	11
27a-sgRNA-2	CACCGACACCAAGTCGTGTTCACAGNGG	X	0	7	154
27a-sgRNA-2	CACCGACACCAAGTCGTGTTCACAGNGG	X	0	8	1053
27a-sgRNA-2	CACCGACACCAAGTCGTGTTCACAGNGG	X	0	9	8136

Table S6b. The potential mismatch ≤5 off-target regions accessed using the Cas-OFFinder off-target searching tool

sgRNA	Target DNA sequence	Chromosome	Position	Mismatch	Nearby Gene	Position	Direction
23a-sgRNA-1	CcCtcACCCCTGTGCCACGGCCGGCTGG	chr19	13836649	3	MIR23AHG	Exon1	-
23a-sgRNA-1	CACaGcCCCCTGgGtCACGGCCGGaAGG	chr1	22380454	5	/	/	-
23a-sgRNA-1	CACCcACCCCaGaGCCAtGGgCGGCAGG	chr2	64942875	5	LINC02245	Intron 2	-
23a-sgRNA-1	CACaGAggCCTGTGCCACtGCgGGCCGG	chr17	75820690	5	UNK	Intron13	+
23a-sgRNA-1	CAgCGACCCtTGTGgCAgGGCgGGCTGG	chr19	56840441	5	ZIM2 and PEG3	Intron 1/ Intron 1	-
23a-sgRNA-2	gAgCtCAGGGTCGGTTGGAAATCCCTGG	chr8	6611506	3	MIR23AHG	Exon1	+
27a-sgRNA-1	CtCCGTAtGCCCTGCTCCcCgGcCCCGG	chr20	41344813	5	MCPH1	Intron 12	-
27a-sgRNA-1	aACCcTAAcCCCaGCTCCTCAGaCCAGG	chr17	81806093	5	GCGR	Intron 1	-
27a-sgRNA-1	CAgCcTcAGCCCTGCTCtcCAGGCCAGG	chr6	2745083	5	MYLK4	Intron 2	+
27a-sgRNA-1	CACCcccAtCCCTGCTCCTgAGGCCCGG	chr19	13836495	5	MIR23AHG	Exon 1	-

27a-sgRNA-1	gcagcTAAGCCCTGCTCCTCAGGCCAGG	chr19	58354810	5	ZNF497	Exon 3	+
27a-sgRNA-1	CACCcTgAGCtCTGCTCCTCtGGCCTGG	chr9	3390577	4	RFX3	Intron 3	-
27a-sgRNA-2	CACaGACACaAAGTCcTGTTCAgAGAGG	chr12	57797962	4	TSFM	Intron 5	+
27a-sgRNA-2	CACacACACagAGTCtTGTTCACAGTGG	chr17	54181621	5	RP11-213B3.1	Intron 2	-
27a-sgRNA-2	ggtCcACACCAAGTCGTGTTCACAGTGG	chr19	13836457	4	MIR23AHG	Exon 1	-

Table S7 Primer, siRNA, and miRNA mimic and inhibitor sequence used in this study

Primer Name	Sequence
RNU6B qPCR	CCCGCCCCTGCGCAAGGATGAC
miR-16-5p qPCR	CCCTGACACGCAAATTCGTGAAG
miR-23a-3p qPCR	CCCATCACATTGCCAGGGATTTCC
miR-23a-5p qPCR	CCCGGGTTCCTGGGGATGGGATTT
miR-27a-3p qPCR	CCCTTCACAGTGGCTAAGTTCCGC
miR-27a-5p qPCR	CCCAGGGCTTAGCTGCTTGTGAGCA
miR-24-3p qPCR	CCCTGGCTCAGTTCAGCAGGAACAG
miR-24-2-5p qPCR	CCCTGCCTACTGAGCTGAAACACAG
miR-23a-3p mimics	AUCACAUUGCCAGGGAUUUCC
miR-23a-3p inhibitor	GGAAAUCCCUGGCAAUGUGAU
miR-27a-3p mimics	UUCACAGUGGCUAAGUUCCGC
miR-27a-3p inhibitor	GCGGAACUUAGCCACUGUGAA
miR-24-3p mimics	UGGCUCAGUUCAGCAGGAACAG
miR-24-3p inhibitor	CUGUUCCUGCUGAACUGAGCCA
Negtive control	UUCUCCGAACGUGUCACGUTT
GAPDH-qPCR-F	CCCCTTCATTGACCTCAACTAC
GAPDH-qPCR-R	CGCTCCTGGAAGATGGTGA
PURA-qPCR-F	GGCGCTCAAAAGCGAGTTC
PURA-qPCR-R	CTCCTCCACTCCGTAGTCGT
SMC1A-qPCR-F	CATCAAAGCTCGTAACTTCCTCG
SMC1A-qPCR-R	CCCCAGAACGACTAATCTCTTCA
PLK1-qPCR-F	CACCAGCACGTCGTAGGATTC
PLK1-qPCR-R	CCGTAGGTAGTATCGGGCCTC
STAG1-qPCR-F	AAAGTGCAATGCAGTCCGTG
STAG1-qPCR-R	GGCATGGTAAGAGGATAATCACC
CDK1-qPCR-F	AAACTACAGGTCAAGTGGTAGCC
CDK1-qPCR-R	TCCTGCATAAGCACATCCTGA
TTK-qPCR-F	GTGGAGCAGTACCACTAGAAATG
TTK-qPCR-R	CCCAAGTGAACCGGAAAATGA
CDC27-qPCR-F	CCCGTCCAGGCTGCTATATG
CDC27-qPCR-R	AAAGGCGTTCTGCGAGGAAAA
YWHAE-qPCR-F	GATTCGGGAATATCGGCAAATGG
YWHAE-qPCR-R	GCTGGAATGAGGTGTTTGTCC
MAD2L1-qPCR-F	GTTCTTCTCATTCGGCATCAACA

MAD2L1-qPCR-R	GAGTCCGTATTTCTGCACTCG
BMPR2-qPCR-F	CGGCTGCTTCGCAGAATCA
BMPR2-qPCR-R	TCTTGGGGATCTCCAATGTGAG
miR23a-sgRNA1-F	caccgacccctgtgccacggccggc
miR23a-sgRNA1-R	aaacgccggccgtggcacaggggtc
miR23a-sgRNA2-F	caccgcagggtcggttggaaatccc
miR23a-sgRNA2-R	aaacgggatttccaaccgaccctgc
miR27a-sgRNA1-F	caccgtaagccctgctcctcaggcc
miR27a-sgRNA1-F	aaacggcctgaggagcagggcttac
miR27a-sgRNA2-F	caccgacaccaagtcgtgttcacag
miR27a-sgRNA2-F	aaacctgtgaacacgacttggtgtc
CRISPRi dCas9 sgRNA 1	GCTAGGCTCCAGCTCCCCGTG
CRISPRi dCas9 sgRNA 2	GCCTGCCCAGGGCTCAATGA
CRISPRi dCas9 sgRNA 3	GACCCCTGTGCCACGGCCGGC
CRISPRi dCas9 sgRNA 4	GCAGGGTCGGTTGGAAATCCC
CRISPRa dCas9 sgRNA 1	GACACCAAGTCGTGTTCACAG
CRISPRa dCas9 sgRNA 2	GTAGAGGGCCCATGCGGGACA
CRISPRa dCas9 sgRNA 3	GTGTAGCCTCCTTGTCCCGCA
CRISPRa dCas9 sgRNA 4	GTCTAGGAAGTGGCGCCAGCT
pmriGLO miR-23a Forward Primer	AAACTAGCGGCCGCTAGTGCTTTTACAGTGCATAATGTGACT
pmriGLO miR-23a Reverse Primer	CTAGAGTCACATTATGCACTGTAAAAGCACTAGCGGCCGCTAGTTT
pmriGLO miR-27a Forward Primer	AAACTAGCGGCCGCTAGTGTTCACTGTACTTTAACTGTGATT
pmriGLO miR-27a Reverse Primer	CTAGAATCACAGTTAAAGTACAGTGAACACTAGCGGCCGCTAGTTT
pmriGLO miR-23a Mut Forward Primer	AAACTAGCGGCCGCTAGTGCTTTTACAGTGCATCT
pmriGLO miR-23a Mut Reverse Primer	CTAGAGATGCACTGTAAAAGCACTAGCGGCCGCTAGTTT
pmriGLO miR-27a Mut Forward Primer	AAACTAGCGGCCGCTAGTGTTCACTGTACTTTATT
pmriGLO miR-27a Mut Reverse Primer	CTAGAATAAAGTACAGTGAACACTAGCGGCCGCTAGTTT
PURA siRNA1	GUUUAAUUCAUGCAAGGUAAACAAU
PURA siRNA2	GUACACGUUUAAGCUAUUAUU
BMPR2 siRNA1	GCCUAUGGAGUGAAAUUAUUU
BMPR2 siRNA2	GAACGGCUAUGUGCGUUUAAA
TMEM170B siRNA1	ACAGACUGUACUGACAUUAAU
TMEM170B siRNA2	GAUAUGAGCAACGGGAUAAUU

Table S8. Establishment of CRISPR cell models used in this study

Name	Stably express transfected DNA constructs
HepG2+1922	KRAB-Spdcas9, PiggyBac-Tre3g-KRAB-dcas9(Bstx1)-2NLS-p2A-sfGFP-SV40polyA/R （EF1a-Zeocin-f2A-rtTA-1)
HEK293T+1922	KRAB-Spdcas9, PiggyBac-Tre3g-KRAB-dcas9(Bstx1)-2NLS-p2A-sfGFP-SV40polyA/R （EF1a-Zeocin-f2A-rtTA-1)
HepG2+1932	VPR-Spdcas9, PiggyBac-Tre3g-dcas9-VRP- sfGFP- SV40polyA/R （EF1a-Zeocin-f2A-rtTA-1）
HEK293T+1932	VPR-Spdcas9,PiggyBac-Tre3g-dcas9-VRP- sfGFP- SV40polyA/R （EF1a-Zeocin-f2A-rtTA-1）
HEK293T+1922-gRNA	pSLQ1922+pSLQ1651-CRISPRi gRNA
HEK293T+1932-gRNA	pSLQ1932+pSLQ1651-CRISPRa gRNA
HepG2+1922-gRNA	pSLQ1922+pSLQ1651-CRISPRi gRNA
HepG2+1932-gRNA	pSLQ1932+pSLQ1651-CRISPRa gRNA
